# Supplementary material for: Expedient access to bora-butenolide bioisosteres by counteranion-mediated trans-hydroboration of alkynes
Source: Nat Commun. 2025 May 27;16:4897. doi: 10.1038/s41467-025-60052-5 (PMC12116781; doi:10.1038/s41467-025-60052-5)
Supplement: Supplementary file 1 — Supplementary Information [file 41467_2025_60052_MOESM1_ESM.pdf]

Supplementary Information

Expedient access to bora-butenolide bioisosteres by counteranion-mediated *trans*-hydroboration of alkynes

Contents

1. General Methods S1
2. Experimental Procedures S2
3. Mechanistic Experiments S8
4. Computational Studies S11
5. Characterizations S14
6. NMR Spectra S47
7. Supporting Crystallographic Data S141
8. References S142

1. General Methods

Chemicals were purchased from commercial suppliers and used as delivered. *n*-BuLi (2.5 M in hexane), *t*-BuOLi, NaH, $NaNH_2$, NaHMDS (2M in THF), LiHMDS (1M in THF), KHMDS (1M in THF) and various boranes are bought from Adamas, TCI and Sigma-Aldrich. Propargyl alcohol derivatives were prepared according to related literatures[1-7]. Deuterated solvents were bought from Adamas. NMR spectra were, if not mentioned otherwise, recorded at room temperature on the following spectrometers: Bruker Avance-III-500. Chemical shifts are given in ppm and coupling constants in Hz. The following abbreviations were used for ^1H NMR spectra to indicate the signal multiplicity: s (singlet), brs (broad singlet), d (doublet), t (triplet), q (quartet), quint (quintet), sext (sextet), sept (septet) and m (multiplet) as well as combinations of them. When combinations of multiplicities are given the first character noted refers to the biggest coupling constant. All ^{13}C NMR spectra were measured with ^1H-decoupling. Mass spectra (MS and HRMS) were measured on an Agilent 6546 TOF LC-MS spectrometer. Infrared X-ray crystal structure analyses were measured on a Bruker D8 Quest instrument using Mo-K_α-radiation. Diffraction intensities were corrected for Lorentz and polarization effects. An empirical absorption correction was applied using SADABS based on the Laue symmetry of reciprocal space. Heavy atom diffractions were solved by direct methods and refined against F2 with full matrix least square algorithm. Hydrogen atoms were either isotropically refined or calculated. The structures were solved and refined using the SHELXTL software package. Melting Points were measured in open glass capillaries in a Büchi melting point apparatus. Flash Column Chromatography was accomplished using Silica gel 60 (0.04 - 0.063 mm / 230 - 400 mesh ASTM) purchased from Santai Science Inc. or Aluminium oxide (neutral or basic) purchased from Santai Science Inc.. As eluents, mixtures of petroleum ether (PE), ethyl acetate (EA), dichloromethane (DCM) and methanol (MeOH) were used. Analytical Thin Layer Chromatography (TLC) was carried out on precoated Yantai POLYGRAM® SIL G/UV254 or POLYGRAM® ALOX N/UV254 plastic sheets. Detection was accomplished using UV-light (254 nm), $KMnO_4$ (in 1.5M Na_2CO_3 (aq.)), molybdatophosphoric acid (5 % in ethanol), vanillin/H_2SO_4 (in ethanol) or anisaldehyde/HOAc (in ethanol). IUPAC names of the compounds described in the experimental section were determined with the program ACDLabs 12.0®.

2. Experimental Procedures

2.1 General procedure for the synthesis of propargyl alcohol[1-7]:

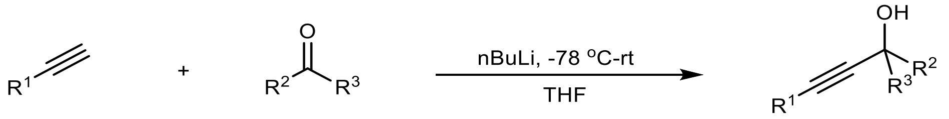

General procedure A: Under an argon atmosphere, an oven-dried Schlenk tube with a magnetic stir bar was charged with alkyne (1.3 mmol) and anhydrous THF. n-BuLi (2.50 M in THF, 1.1 mmol) was then added to this mixture at –78 °C, then stirring for 30 minutes at the same temperature. Subsequently, the corresponding ketone (1.0 mmol) was added. The reaction vessel was sealed with a Teflon-lined screw cap and warmed to room temperature. After continuously stirring for 4 hours, the reaction was quenched with aqueous NH_4Cl solution, followed by extraction with EtOAc for three times (3×20 mL). The combined organic layer was dried over anhydrous Na_2SO_4, filtered, and concentrated in vacuo. The residue was purified by flash column chromatography on silica gel to afford the desired product. The characterizations of unreported products were listed in Chapter 5.

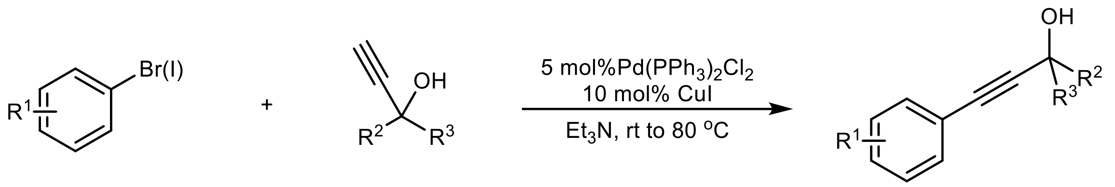

General procedure B: Under N_2 atmosphere, to a mixture of $Pd(PPh_3)_2Cl_2$ (5 mol %) and CuI (10 mol %) was added aryl halide (1.1 mmol), corresponding propargyl alcohol (1.3 mmol) and Et_3N (10 ml). The reaction vessel was sealed with a Teflon-lined screw cap and stirred at 80 °C for 12 h. Subsequently, the reaction was quenched with aqueous NH_4Cl solution (100 mL), followed by extraction with EtOAc (3×20 mL). The combined organic layer was dried over anhydrous Na_2SO_4, filtered, and concentrated in vacuo. The residue was purified by flash column chromatography on silica gel to afford the desired coupling product. The characterizations of unreported products were listed in Chapter 5.

2.2 General procedure for NaHMDS-promoted *trans*-hydroboration of alkynes:

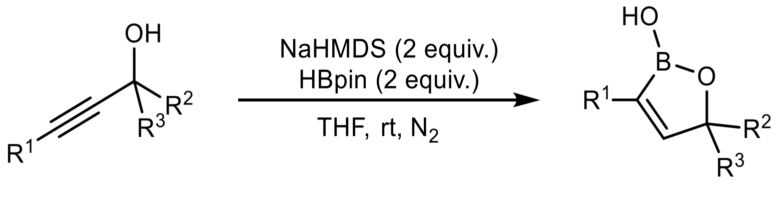

General procedure C: An oven-dried Schlenk tube equipped with a magnetic stir bar was charged with propargyl alcohol (0.2 mmol), anhydrous THF (2 mL) and NaHMDS (2.0 M THF solution, 0.4 mmol) at 0°C under N_2 atmosphere. The mixture was stirred for 10 min at 0 °C, then HBpin (0.4 mmol, 58 μL) was introduced. Subsequently, the reaction vessel was sealed with a Teflon-lined screw cap and removed to room temperature. After continuously stirring for 6 h, the reaction was quenched with aqueous NH_4Cl solution (10 mL), followed by extraction with EtOAc (3×10 mL). The combined organic layer was dried over anhydrous

Na_2SO_4, filtered, and concentrated in vacuo. The residue was purified by flash column chromatography on silica gel to afford the desired product.

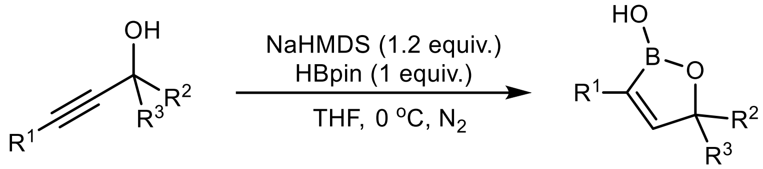

General procedure D: An oven-dried Schlenk tube equipped with a magnetic stir bar was charged with propargyl alcohol (0.2 mmol), anhydrous THF (2 mL) and NaHMDS (2.0 M THF solution, 0.24 mmol) at 0 °C under N_2 atmosphere. The mixture was stirred for 10 min at 0 °C, then HBpin (0.2 mmol, 29 μL) was introduced. Subsequently, the reaction vessel was sealed with a Teflon-lined and kept in ice baths. After continuously stirring for 12 h at 0 °C, the reaction was quenched with aqueous NH_4Cl solution (10 mL), followed by extraction with EtOAc (3×10 mL). The combined organic layer was dried over anhydrous Na_2SO_4, filtered, and concentrated in vacuo. The residue was purified by flash column chromatography on silica gel to afford the desired product.

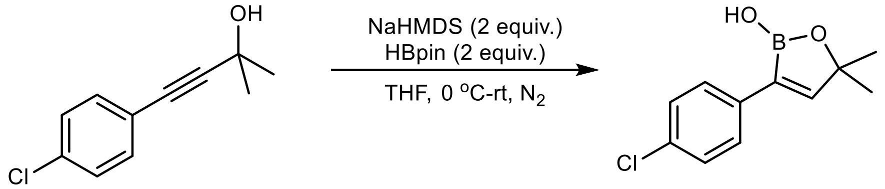

Representative procedure for the scale-up reaction: An oven-dried Schlenk tube equipped with a magnetic stir bar was charged under N_2 atmosphere with propargyl alcohol (1.03 g, 5.3 mmol), anhydrous THF (10 mL) and NaHMDS (2.0 M THF solution, 5.3 mL) at 0 °C. The mixture was stirred for 10 min at 0 °C, then HBpin (1.54 mL, 10.6 mmol) was added slowly. Subsequently, the reaction vessel was sealed with a Teflon-lined screw cap and removed to room temperature. After continuously stirring for 6 h, the reaction was quenched with aqueous NH_4Cl (100 mL), followed by extraction with EtOAc (3×50 mL). The combined organic layer was dried over anhydrous Na_2SO_4, filtered, and concentrated in vacuo. The residue was purified by flash column chromatography on silica gel (PE/ EA = 10/1) to afford the desired product (1.10 g, 93% yield).

2.3 Representative procedure for downstream derivatizations:

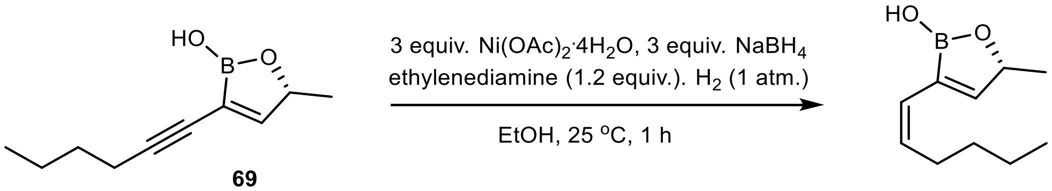

On account of this literature[8], an Schlenk tube equipped with a magnetic stir bar was charged with $Ni(OAc)_2 \cdot 4H_2O$ (0.3 mmol, 74.7 mg), $NaBH_4$ (0.3 mmol, 11.4 mg) and EtOH (2.5 mL). The mixture was stirred for 30 mins at room temperature. Then, ethylenediamine (0.12 mmol, 9.8 μL) and the compound **69** (0.1 mmol, 17.8 mg) was added in sequence. The reaction mixture was stirred under 1 atm of hydrogen for 1 hour, and quenched with aqueous NH_4Cl

solution (10 mL), followed by extraction with EtOAc (3×10 mL). The combined organic layer was dried over anhydrous Na_2SO_4, filtered, and concentrated in vacuo. The residue was purified by flash column chromatography on silica gel to afford the desired product **70** in 55% yield (9.9 mg).

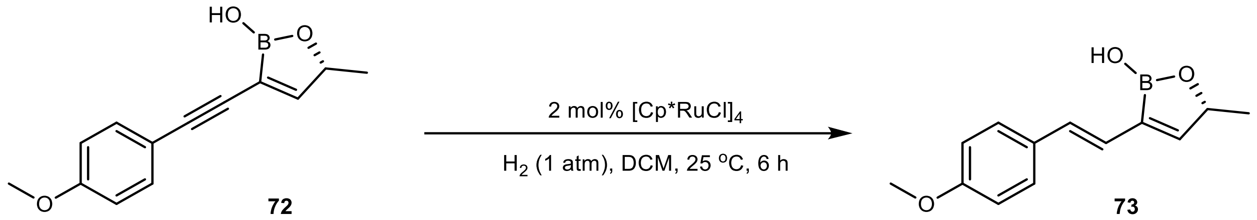

An Schlenk tube equipped with a magnetic stir bar was charged with $[Cp^*RuCl]_4$ (4,35 mg), the compound **72** (0.2 mmol, 45.6 mg) and DCM (2 mL). The reaction mixture was stirred under 1 atm of hydrogen for 6 hours, and then concentrated in vacuo. The residue was purified by flash column chromatography on silica gel to afford the desired product **73** in 55% yield (9.9 mg).

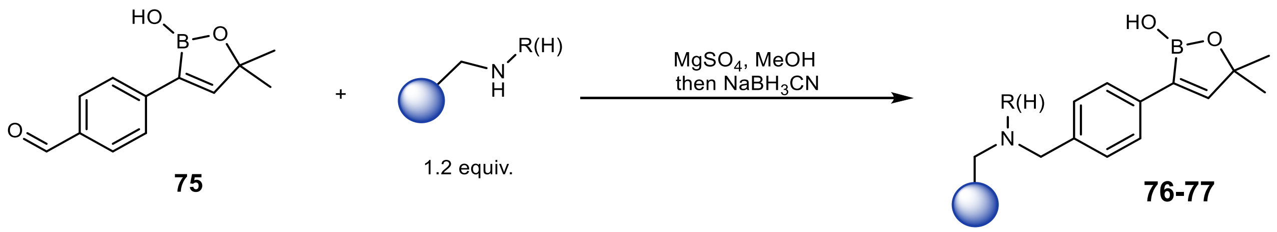

An oven-dried Schlenk tube equipped with a magnetic stir bar was charged with the oxaborole **75** (0.2 mmol, 40.4 mg), the corresponding amine (1.2 equiv.), anhydrous MeOH (2 mL) and anhydrous $MgSO_4$ (0.8 mmol, 96 mg). The mixture was stirred for 12 hours at room temperature. Then, $NaBH_3CN$ (0.6 mmol, 37.8 mg) was added. After continuously stirring for 2 h, the reaction was quenched with aqueous NH_4Cl solution (10 mL), followed by extraction with EtOAc (3×10 mL). The combined organic layer was dried over anhydrous Na_2SO_4, filtered, and concentrated in vacuo. The residue was purified by flash column chromatography on silica gel to afford the desired product **76** in 81% yield (96.4 mg), and **77** in 71% yield (70.0 mg), respectively.

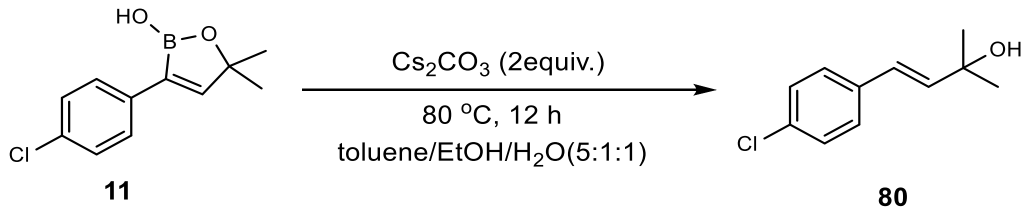

A Schlenk tube equipped with a magnetic stir bar was charged with the oxaborole **11** (44.5 mg, 0.2 mmol), Cs_2CO_3 (130.3 mg, 0.4 mmol) and toluene/EtOH/H_2O (2 mL/0.4 mL/0.4 mL). The resultant suspension was stirred at 80 °C for 12 hours. To the mixture was added brine and extracted with EtOAc for three times. The combined organic layer was dried over anhydrous Na_2SO_4, filtered, and concentrated on a rotary evaporator. The crude product was purified by flash column chromatography on silica gel (PE/ EA = 10/1). The product **80** was obtained as a colorless solid in 80% yield (30.6 mg).

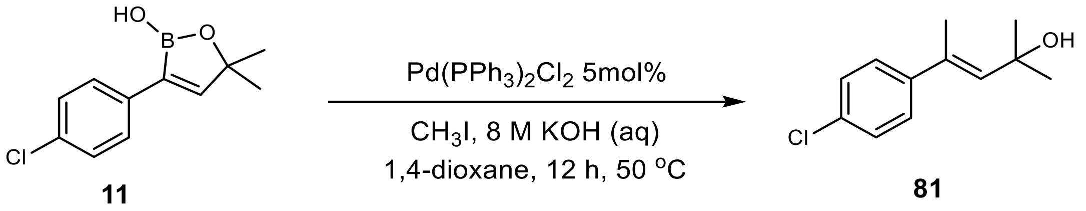

A Schlenk tube equipped with a magnetic stir bar was charged under N_2 atmosphere with the oxaborole **11** (44.5 mg, 0.2 mmol), $Pd(PPh_3)_2Cl_2$ (7.0 mg, 0.01 mmol) and 1,4-dioxane (2 mL). To the mixture was added iodomethane (24.9 µL, 0.4 mmol) and 8 M aqueous KOH solution (75 µL, 0.6 mmol) in sequence. The resultant suspension was stirred at 50 ºC for 12 hours. The reaction was quenched with brine and extracted with EtOAc for three times. The combined organic layer was dried over anhydrous Na_2SO_4, filtered, and concentrated on a rotary evaporator. The crude product by flash column chromatography on silica gel (PE/ EA = 10/1) to afford the desired product **81** in 64% yield (26.9 mg).

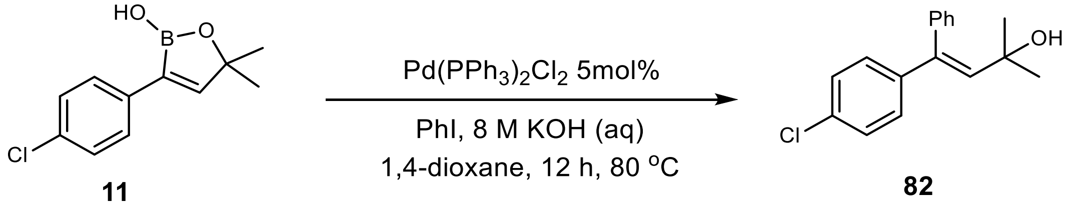

A Schlenk tube equipped with a magnetic stir bar was charged under N_2 atmosphere with the oxaborole **11** (44.5 mg, 0.2 mmol), $Pd(PPh_3)_2Cl_2$ (7.0 mg, 0.01 mmol) and 1,4-dioxane (2 mL). To the mixture was added iodobenzene (44.7 µL, 0.4 mmol) and 8 M aqueous KOH solution (75 µL, 0.6 mmol) in sequence. The resultant suspension was stirred at 80 ºC for 12 hours. The reaction was quenched with brine and extracted with EtOAc for three times. The combined organic layer was dried over anhydrous Na_2SO_4, filtered, and concentrated on a rotary evaporator. The crude product by flash column chromatography on silica gel (PE/ EA = 10/1) to afford the desired product **82** in 65% yield (35.4 mg).

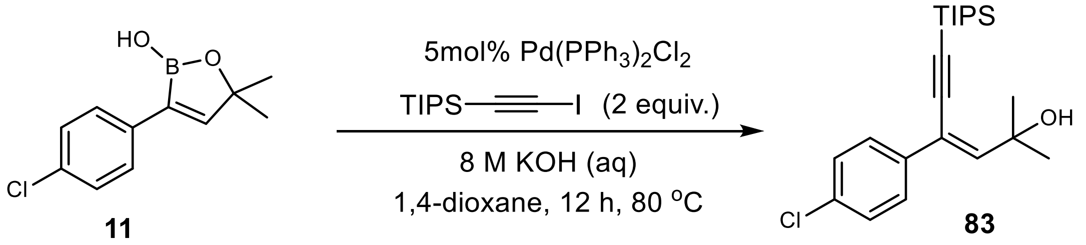

A Schlenk tube equipped with a magnetic stir bar was charged under N_2 atmosphere with the oxaborole **11** (44.5 mg, 0.2 mmol), $Pd(PPh_3)_2Cl_2$ (7.0 mg, 0.01 mmol) and 1,4-dioxane (2 mL). To the mixture was added (2-iodoethynyl)tris(1-methylethyl)silane (99.6 µL, 0.4 mmol) and 8 M aqueous KOH solution (75 µL, 0.6 mmol) in sequence. The resultant suspension was stirred at 80 ºC for 12 hours. The reaction was quenched with brine and extracted with EtOAc for three times. The combined organic layer was dried over anhydrous Na_2SO_4, filtered, and concentrated on a rotary evaporator. The crude product by flash column chromatography on silica gel (PE/ EA = 10/1) to afford the desired product **83** in 75% yield (54.0 mg).

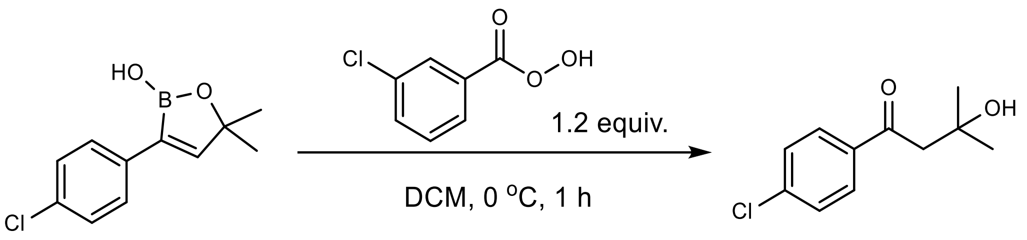

11 **84**

A Schlenk tube equipped with a magnetic stir bar was charged with the oxaborole **11** (44.5 mg, 0.2 mmol), *m*-CPBA (41.4 mg, 0.24 mmol) and dichloromethane (2 mL). The reaction was stirred at 0 °C for 1 hour. To the reaction was added brine and extracted with EtOAc for three times. The combined organic layer was dried over anhydrous Na_2SO_4, filtered, and concentrated on a rotary evaporator. The crude product by flash column chromatography on silica gel (PE/ EA = 10/1) to afford the desired product **84** in 85% yield (36.2 mg).

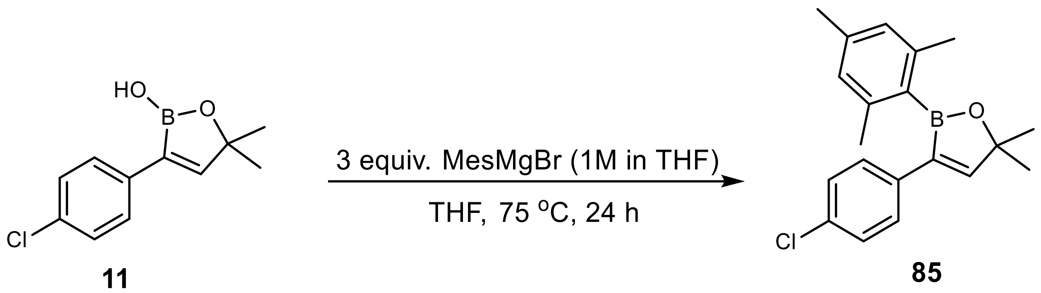

11 **85**

An oven-dried Schlenk tube equipped with a magnetic stir bar was charged with the oxaborole **11** (44.5mg, 0.2 mmol), anhydrous THF (3 mL) and MesMgBr (0.3 mmol, 1 M THF solution, 0.3 mL) at 0°C under N_2 atmosphere. The reaction vessel was sealed with a Teflon-lined and stirred at 75 °C for 24 h. Subsequently, the reaction was quenched with aqueous NH_4Cl solution (10 mL), followed by extraction with EtOAc for three times (3×10 mL). The combined organic layer was dried over anhydrous Na_2SO_4, filtered, and concentrated in vacuo. The residue was purified by flash column chromatography on silica gel (PE) to afford the desired product **85** in 82 % yield (53.2 mg).

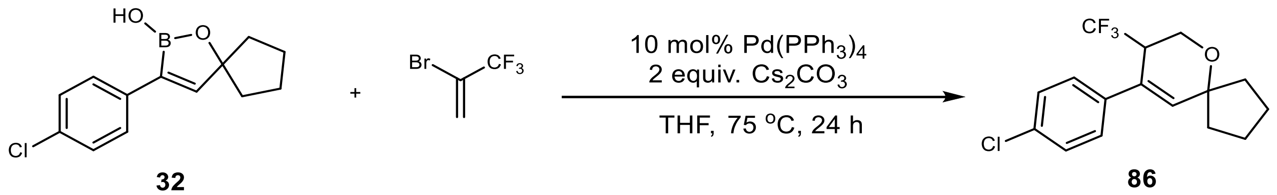

32 **86**

A Schlenk tube equipped with a magnetic stir bar was charged under N_2 atmosphere with the oxaborole **32** (49.7 mg, 0.2 mmol), $Pd(PPh_3)_4$ (23.1 mg, 0.02 mmol), Cs_2CO_3 (130.2 mg, 0.4 mmol), DMF/H_2O (2 mL/0.2 mL) and 2-bromo-3,3,3-trifluoroprop-1-ene (22.8 µL, 0.22 mmol). The resultant suspension was stirred at 100 °C for 24 hours. The reaction was quenched with brine and extracted with EtOAc for three times. The combined organic layer was dried over anhydrous Na_2SO_4, filtered, and concentrated on a rotary evaporator. The crude product by flash column chromatography on silica gel (PE/ EA = 10/1) to afford the desired product **86** in 47% yield (29.7 mg).

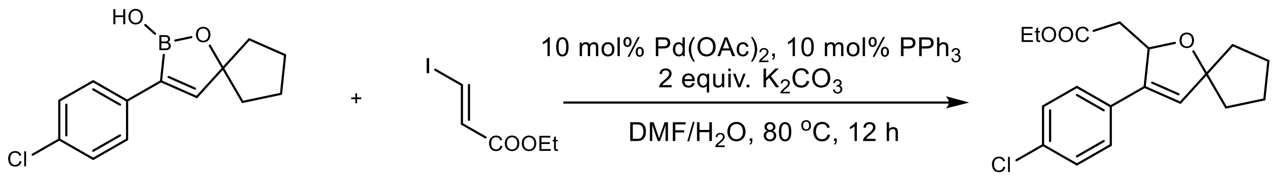

32 **87**

A Schlenk tube equipped with a magnetic stir bar was charged under N_2 atmosphere with the oxaborole **32** (49.7 mg, 0.2 mmol), $Pd(OAc)_2$ (4.5 mg, 0.02 mmol), PPh_3 (5.2 mg, 0.02 mmol), K_2CO_3 (55.2 mg, 0.4 mmol), DMF/H_2O (2 mL/0.2 mL) and (*E*)-Ethyl 3-iodoacrylate (42.3 mg, 0.22 mmol). The resultant suspension was stirred at 80 °C for 12 hours. The reaction was quenched with brine and extracted with EtOAc for three times. The combined organic layer was dried over anhydrous Na_2SO_4, filtered, and concentrated on a rotary evaporator. The crude product by flash column chromatography on silica gel (PE/ EA = 10/1) to afford the desired product **87** in 64% yield (39.4 mg).

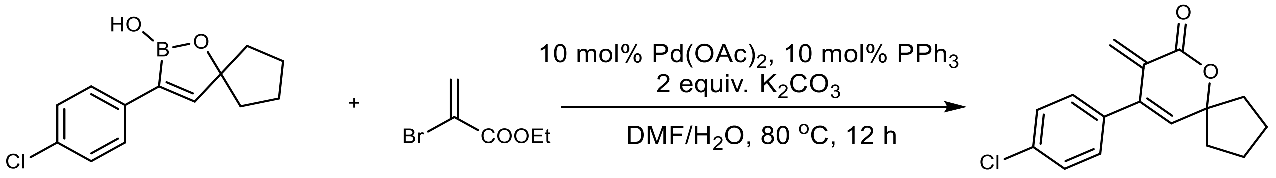

32 **88**

A Schlenk tube equipped with a magnetic stir bar was charged under N_2 atmosphere with the oxaborole **32** (49.7 mg, 0.2 mmol), $Pd(OAc)_2$ (4.5 mg, 0.02 mmol), PPh_3 (5.2 mg, 0.02 mmol), K_2CO_3 (55.2 mg, 0.4 mmol), DMF/H_2O (2 mL/0.2 mL) and (*E*)-Ethyl 3-iodoacrylate (42.3 mg, 0.22 mmol). The resultant suspension was stirred at 80 °C for 12 hours. The reaction was quenched with brine and extracted with EtOAc for three times. The combined organic layer was dried over anhydrous Na_2SO_4, filtered, and concentrated on a rotary evaporator. The crude product by flash column chromatography on silica gel (PE/ EA = 10/1) to afford the desired product **88** in 53% yield (29.1 mg).

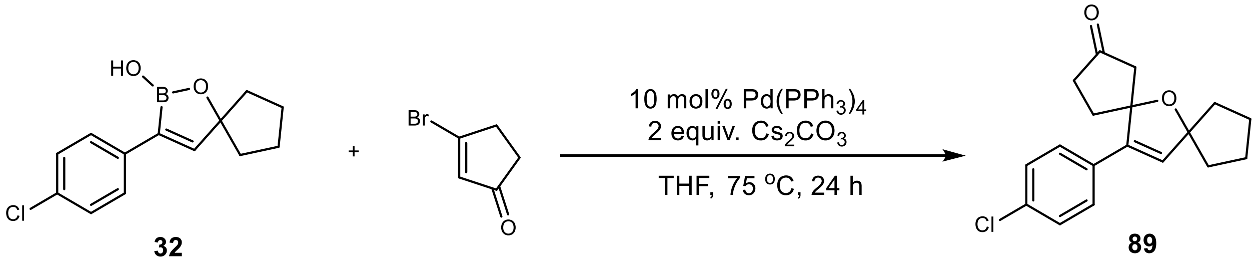

32 **89**

A Schlenk tube equipped with a magnetic stir bar was charged under N_2 atmosphere with the oxaborole **32** (49.7 mg, 0.2 mmol), $Pd(PPh_3)_4$ (23.1 mg, 0.02 mmol), Cs_2CO_3 (130.2 mg, 0.4 mmol), DMF/H_2O (2 mL/0.2 mL) and methyl 2-bromoacrylate (21.2 μL, 0.22 mmol). The resultant suspension was stirred at 75 °C for 24 hours. The reaction was quenched with brine and extracted with EtOAc for three times. The combined organic layer was dried over anhydrous Na_2SO_4, filtered, and concentrated on a rotary evaporator. The crude product by flash column chromatography on silica gel (PE/ EA = 10/1) to afford the desired product **89** in 61% yield (38.5 mg).

3. Mechanistic Experiments

Representative procedure for Fig. 4, equation (1): An oven-dried Schlenk tube equipped with a magnetic stir bar was charged under N_2 atmosphere with propargyl alcohol **S11** (39 mg, 0.2 mmol), anhydrous THF (2 mL) and NaHMDS (2.0 M THF solution, 0.2 mL, 0.4 mmol) at 0°C. The mixture was stirred for 10 min at 0 °C, then DBpin[9] (0.06 mL, 0.4 mmol) was added. Subsequently, the reaction vessel was sealed with a Teflon-lined screw cap and removed to room temperature. After continuously stirring for 6 h, the reaction was quenched with aqueous NH_4Cl solution (10 mL), followed by extraction with EtOAc (3×10 mL). The combined organic layer was dried over anhydrous Na_2SO_4, filtered, and concentrated in vacuo. The residue was purified by flash column chromatography on silica gel (PE/ EA = 10/1) to afford the deuterated product (41.6 mg, 93% yield).

Representative procedure for Fig. 4, equation (2): An oven-dried Schlenk tube equipped with a magnetic stir bar was charged under N_2 atmosphere with propargyl alcohol **S11** (39 mg, 0.2 mmol), anhydrous THF (2 mL), 15-crown-5 ether (0.5 mmol, 110 mg) and NaHMDS (2.0 M THF solution, 0.2 mL, 0.4 mmol) at 0°C. The mixture was stirred for 10 min at 0 °C, and then HBpin (0.058 mL, 0.4mmol) was added. Subsequently, the reaction vessel was sealed with a Teflon-lined screw cap and removed to room temperature. After continuously stirring for 6 h, the reaction was quenched with aqueous NH_4Cl solution (10 mL), followed by extraction with EtOAc (3×10 mL). The combined organic layer was dried over anhydrous Na_2SO_4, filtered, and concentrated in vacuo. The residue was purified by flash column chromatography on silica gel (PE/ EA = 10/1) to afford the product (37.8 mg, 85% yield).

Representative procedure for Fig. 4, equation (3): An oven-dried Schlenk tube equipped with a magnetic stir bar was charged under N_2 atmosphere with propargyl alcohol **S11** (39 mg, 0.2 mmol), anhydrous THF (2 mL) and NaH (60%, dispersion in paraffin liquid, 16 mg, 0.4 mmol) at 0°C. The mixture was stirred for 10 min at 0 °C, and then HBpin (0.058 mL, 0.4mmol) was added. Subsequently, the reaction vessel was sealed with a Teflon-lined screw cap and removed to room temperature. After continuously stirring for 6 h, the reaction was detected by thin layer chromatography.

Representative procedure for Fig. 4, equation (4): An oven-dried Schlenk tube equipped with a magnetic stir bar was charged under N_2 atmosphere with propargyl alcohol **S11** (39 mg, 0.2 mmol), anhydrous THF (2 mL), NaH (60%, dispersion in paraffin liquid, 16 mg, 0.4 mmol) and NaHMDS (2.0 M THF solution, 0.02 mL, 0.04 mmol) at 0°C. The mixture was stirred for 10 min at 0 °C, and then HBpin (0.058 mL, 0.4mmol) was added. Subsequently, the reaction vessel was sealed with a Teflon-lined screw cap and removed to room temperature. After continuously stirring for 6 h, the reaction was quenched with aqueous NH_4Cl solution (10 mL), followed by extraction with EtOAc (3×10 mL). The combined organic layer was dried over anhydrous Na_2SO_4, filtered, and concentrated in vacuo. The residue was purified by flash column chromatography on silica gel (PE/ EA = 10/1) to afford the product (21.8 mg, 49% yield).

Representative procedure for Fig. 4, equation (5): An oven-dried Schlenk tube equipped with a magnetic stir bar was charged under N_2 atmosphere with HBpin (0.058 mL, 0.4 mmol),

anhydrous THF (2 mL) and NaHMDS (2.0 M THF solution, 0.02 mL, 0.4 mmol). The mixture was stirred for 30 min at 25 °C, and then propargyl alcohol **S11** (39 mg, 0.2 mmol) was added. After continuously stirring for 6 h, the reaction was quenched with aqueous NH_4Cl solution (10 mL), followed by extraction with EtOAc (3×10 mL). The combined organic layer was dried over anhydrous Na_2SO_4, filtered, and concentrated in vacuo. The residue was purified by flash column chromatography on silica gel (PE/ EA = 10/1) to afford the product (31.1 mg, 70% yield).

Representative procedure for Fig. 4, equation (6): An oven-dried Schlenk tube equipped with a magnetic stir bar was charged under N_2 atmosphere with HBpin (0.058 mL, 0.4 mmol), anhydrous THF (2 mL) and NaHMDS (2.0 M THF solution, 0.02 mL, 0.4 mmol). The mixture was stirred for 30 min at 25 °C, and then propargyl alcohol **S11** (39 mg, 0.2 mmol) was added. After continuously stirring for 6 h, to the mixture was supplied 1 equiv. HBpin (0.029 mL, 0.2 mmol). The reaction was further stirred for 6 h and quenched with aqueous NH_4Cl solution (10 mL), followed by extraction with EtOAc (3×10 mL). The combined organic layer was dried over anhydrous Na_2SO_4, filtered, and concentrated in vacuo. The residue was purified by flash column chromatography on silica gel (PE/ EA = 10/1) to afford the product (42.3 mg, 95% yield).

Representative procedure for Fig. 4, equation (7): An oven-dried Schlenk tube equipped with a magnetic stir bar was charged under N_2 atmosphere with HBpin (0.058 mL, 0.4 mmol), anhydrous THF (2 mL) and NaHMDS (2.0 M THF solution, 0.02 mL, 0.4 mmol). The mixture was stirred for 30 min at 25 °C, and then propargyl alcohol **S11** (39 mg, 0.2 mmol) was added. After continuously stirring for 6 h, to the mixture was supplied 1 equiv. $LiBH_4$ (4.3 mg, 0.2 mmol). The reaction was further stirred for 6 h and quenched with aqueous NH_4Cl solution (10 mL), followed by extraction with EtOAc (3×10 mL). The combined organic layer was dried over anhydrous Na_2SO_4, filtered, and concentrated in vacuo. The residue was purified by flash column chromatography on silica gel (PE/ EA = 10/1) to afford the product (31.1 mg, 70% yield).

Representative procedure for the NMR titration experiment, Fig. 4a-c: In a nitrogen-filled glovebox, an oven-dried NMR tube was charged with NaHMDS (2.0 M THF solution, 50 μL, 0.1 mmol) and THF-d_8 (0.5 mL) and sealed with a rubber stopper. To the mixture was introduced HBpin (29 μL, 0.2 mmol, 25.6 mg), and the ^{11}B-NMR spectrum was examined immediately. Subsequently, an additional 12 μL of HBpin (0.08 mmol, 10.2 mg) was added to the NMR tube, and the ^{11}B-NMR spectrum was rapidly acquired. Finally, the ^{11}B-NMR spectrum was measured again after the addition of an extra 3 μL of HBpin (0.02 mmol, 2.6 mg).

Measurement of the ^{11}B NMR spectrum of K[HMDS-HBpin]: The synthesis is on account of this report[10]. In a nitrogen-filled glovebox, an oven-dried NMR tube was charged with freshly distilled HMDS-Bpin (17μL, 0.1 mmol), THF-d_8 (0.5 mL) and KH (30 wt% in mineral oil, 133.4 mg, 1 mmol). After being removed from the glovebox, the tube was sealed and the mixture was refluxed for 12 hours in oil bath. The ^{11}B-NMR spectrum of the in-situ generated K[HMDS-Bpin] was shown below.

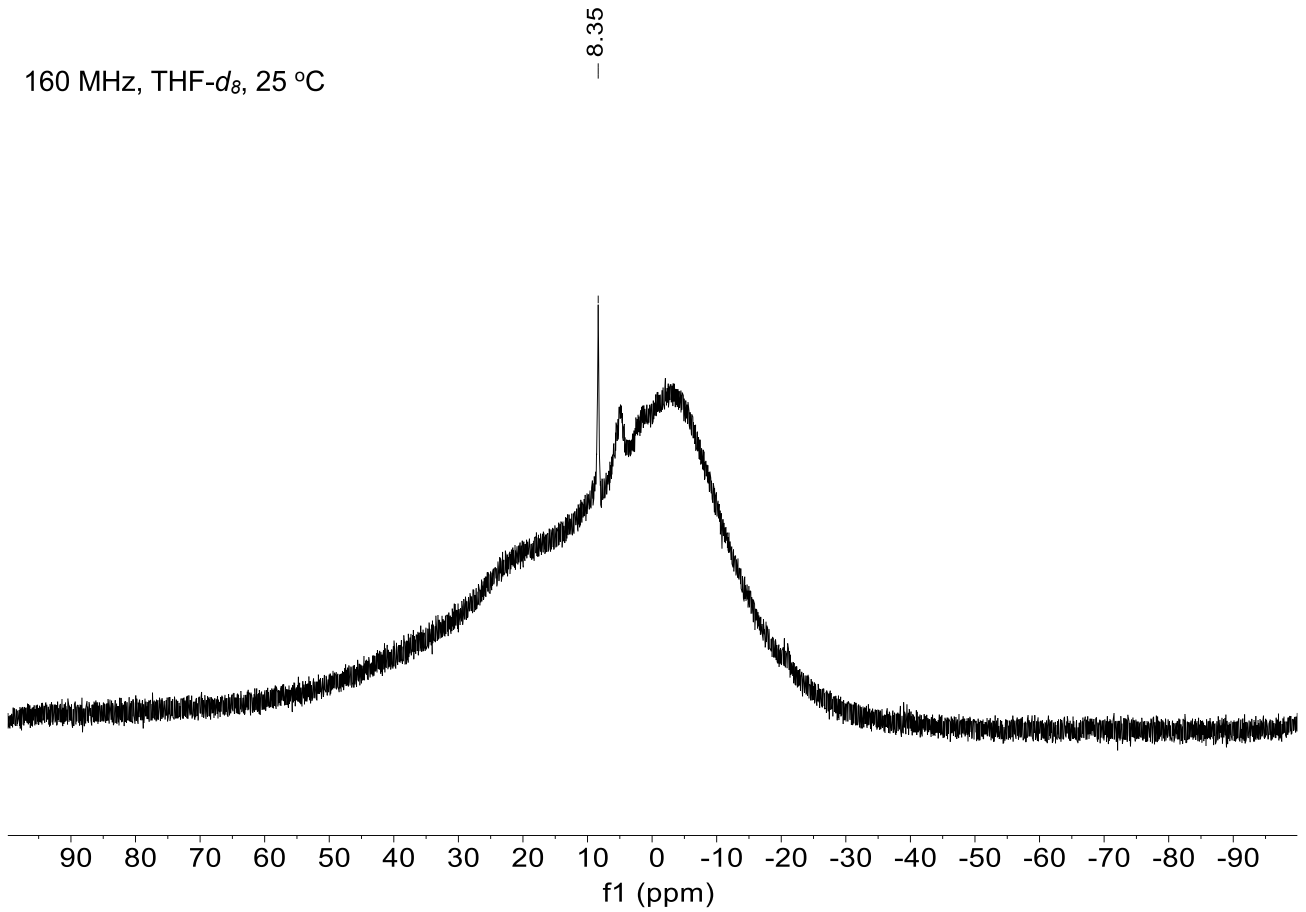

Figure S1. The ^{11}B NMR spectrum of in-situ generated K[HMDS-HBpin] (THF-*d*8).

Representative procedure for Fig. 4d: In a nitrogen-filled glovebox, an oven-dried NMR tube was charged with the propargyl alcohol **S11** (0.1 mmol, 19.4 mg), NaH (60%, dispersion in paraffin liquid, 4 mg, 0.1 mmol) and THF-*d*8 (0.5 mL), then sealed with a rubber stopper. The NMR tube was subjected to ultrasonic conditions for 10 minutes. Subsequently, HMDS-Bpin (0.1 mmol, 17 μL) was added. The ^{11}B-NMR spectrum was examined immediately.

The synthesis of HMDS-Bpin: An oven-dried Schlenk tube equipped with a magnetic stir bar was charged under N_2 atmosphere with HBpin (1.43 mL, 10 mmol), followed by the slow addition of NaHMDS (2.0 M THF solution, 5 mL) at 0°C. The reaction mixture was allowed to warm up slowly to room temperature, and stirred 1 h. The resulting white precipitate was filtered through celite with N_2 protection. The filtrate was evacuated at reduced pressure, and the residue was purified by vacuum distillation (bp: 50-60 °C/10 mbar) to offer HMDS-Bpin (1.46 g) as a colorless liquid. The ^{1}H-NMR data is in agreement with prior reports[11,12].

Representative procedure for Fig. 4e: In a nitrogen-filled glovebox, an oven-dried NMR tube was charged with the propargyl alcohol **S11** (0.1 mmol, 19.4 mg), NaH (60%, dispersion in paraffin liquid, 4 mg, 0.1 mmol) and THF-*d*8 (0.5 mL), then sealed with a rubber stopper. The NMR tube was subjected to ultrasonic conditions for 10 minutes. Subsequently, a mixture of NaHMDS (2 M in THF, 0.1 mmol, 50 μL) and HBpin (0.2 mmol, 29 μL) stirring for 30 mins in advance was added. The ^{11}B NMR spectrum was examined immediately.

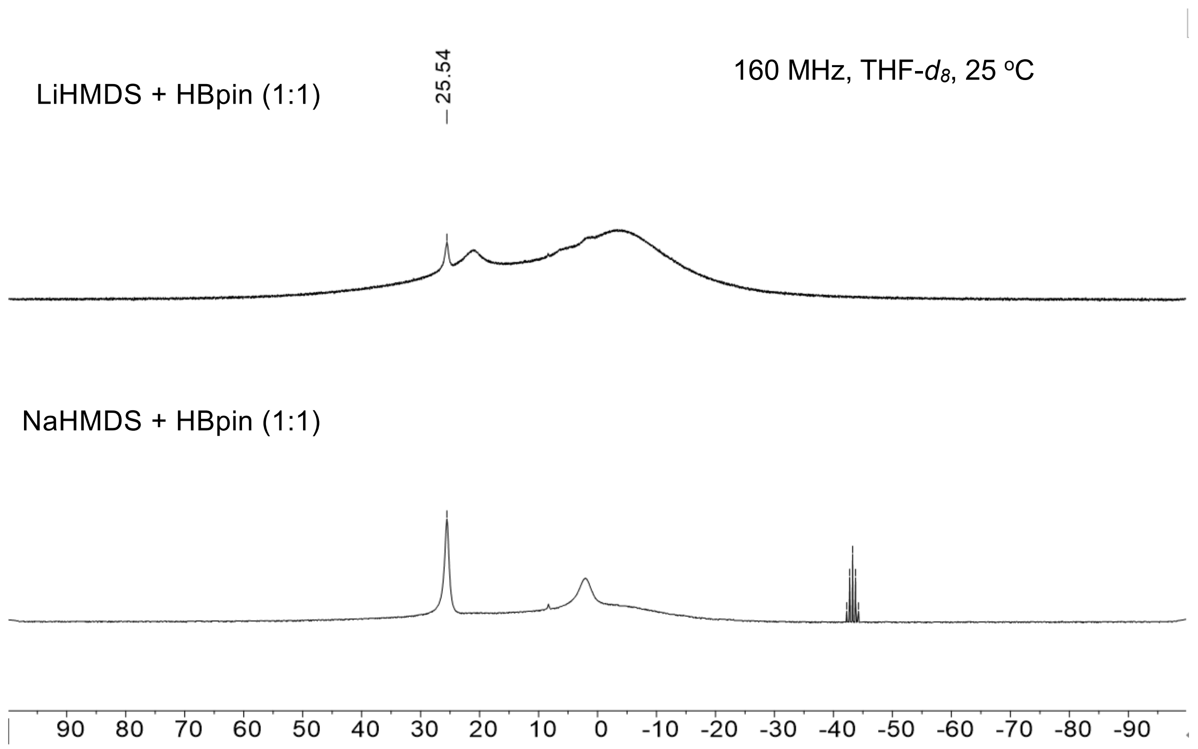

Figure S2. The ^{11}B NMR spectrum of LiHMDS/HBpin (1:1) compared with NaHMDS/HBpin (1:1) (THF-d_8).

4. Computational Studies

All the Density Functional Theory (DFT) calculations were conducted using the Gaussian program[15]. Geometric optimizations of reactants, products, intermediates and transition states were performed in the gas phase, employing B3LYP functional[16] with Grimme's dispersion correction, incorporating Becke–Johnson damping (GD3BJ)[17] of theory. All the atoms were treated with the def2-SVP basis set[18]. Vibrational frequency analyses were performed at the same theoretical level to validate the optimized structures as either local minima (zero imaginary frequencies) or transition states (one imaginary frequency). Thermodynamics corrections were derived from these vibrational frequency analyses. Intrinsic reaction coordinate calculations[19,20] were employed to verify that the transition state structures connect the corresponding reactants and products. To account the solvent (THF) effects and refine the electronic energies, the single-point energy calculations based on the optimized geometries were performed with the Solvent Model Density (SMD) method[21], at the M06-2X[22]/def2-TZVPP[23-27] level of theory. The Gibbs free energy reported is the sum of the electronic energy in solution and the thermal correction in the gas phase at the reaction temperature, expressed in kcal/mol at 1 atm. Structural visualizations were generated using CYLview[28]. Charge population analyses were performed using the Atomic Dipole Moment Corrected Hirshfeld (ADCH) method through the Multiwfn program[29].

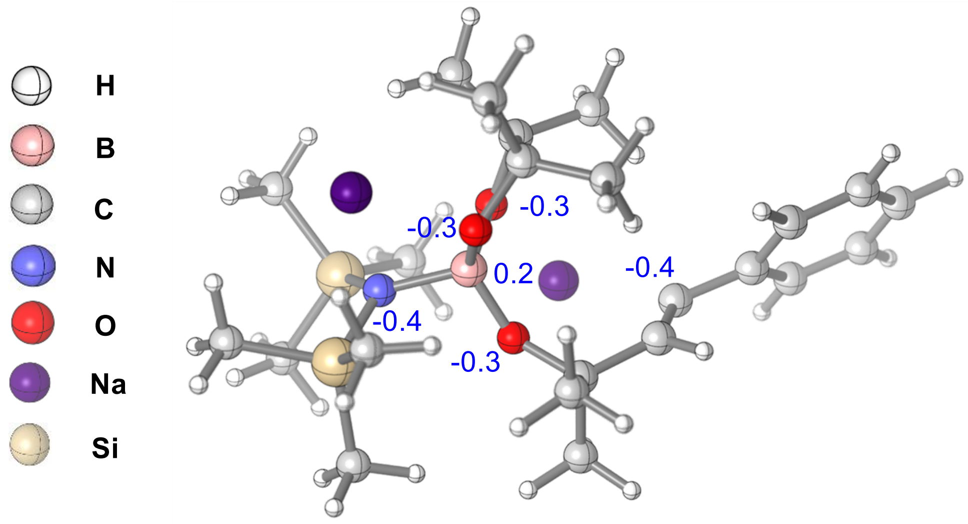

Figure S3. Charge population analyses of intermediate **C**, in which the boron atom shows a positive charge due to the adjacent electronegative N- and O- ligands.

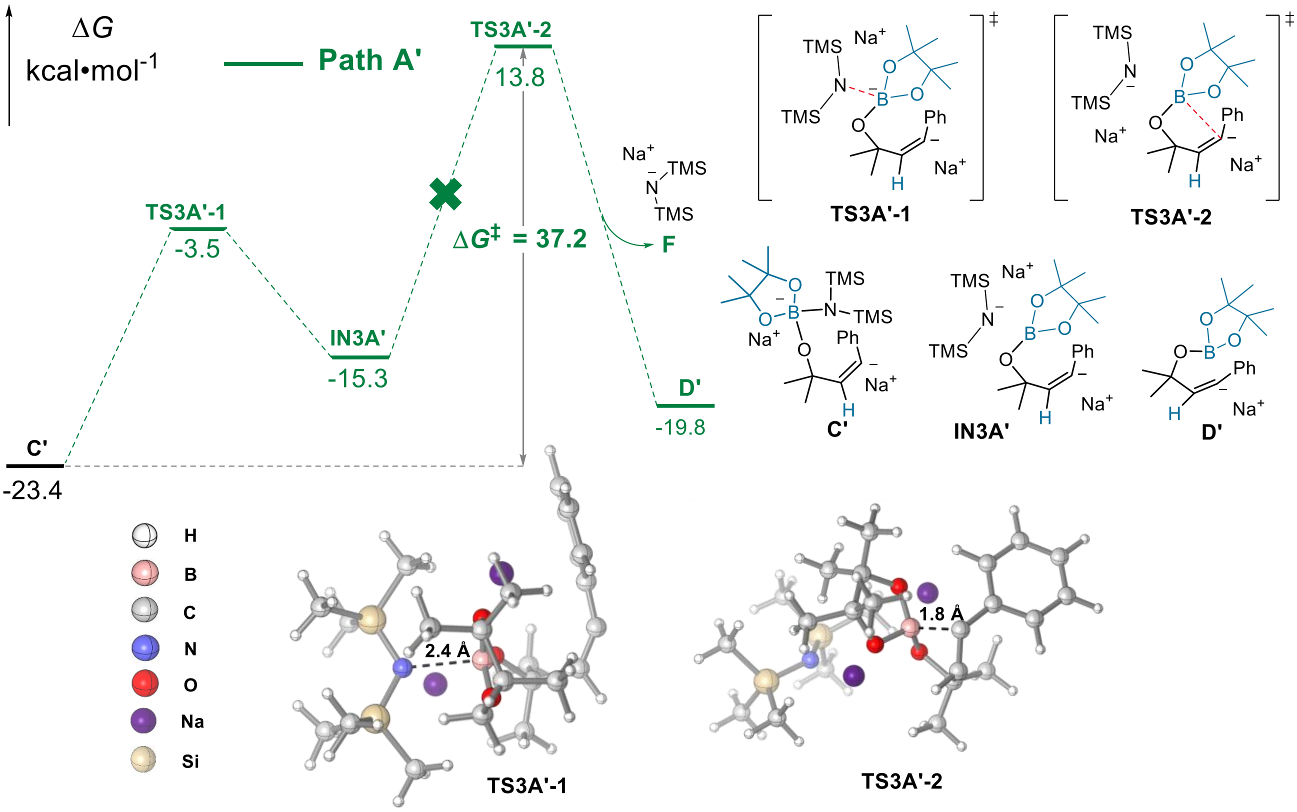

Figure S4. Free-energy profiles of reaction pathways starting from *cis*-configured isomer **C'** computed at the DFT/B3LYP(D3BJ)/def2-SVP//M06-2X/def2-TZVPP/SMD (THF) level of theory. The geometries of the transition states are illustrated below, with the key bond lengths indicated in Angstrom (Å).

We examined two mechanistic pathways for the direct hydroboration reaction between sodium alkoxide **A** and Na[HBpin-HMDS] (Na[B–H]) (Figure S5). In path 1, Na[B-H] initially

attacks the alkyne moiety of **A**, initiating intermolecular hydride transfer via transition state **TSA'**. This step requires a high free activation energy ($\Delta G^{\ddagger}$ = 32.1 kcal/mol), leading to the formation of intermediate **INTA'**. In the subsequent step, **INTA'** couples with the [HMDS-Bpin] fragment to form the boronate complex **C'** through transition state **TSB'** ($\Delta G^{\ddagger}$ = 9.1 kcal/mol). While the second step is energetically accessible, the overall transformation is rendered unfavorable due to the prohibitively high barrier of the initial hydride transfer. In path 2, Na[B-H] first reacts with **A** via transition state **TSA"**, involving B–O bond cleavage and generating intermediate **INTA"**. This step also requires a high activation energy ($\Delta G^{\ddagger}$ = 30.7 kcal/mol). Subsequently, an intramolecular hydride transfer from boron to the alkyne moiety occurs through transition state **TSB"**, forming intermediate **C"**. The high barrier of the initial step again makes this pathway unlikely under the reaction conditions. In summary, both mechanistic possibilities entail free energy barriers exceeding 30 kcal/mol, suggesting that direct hydroboration is not a viable pathway under the reaction conditions employed.

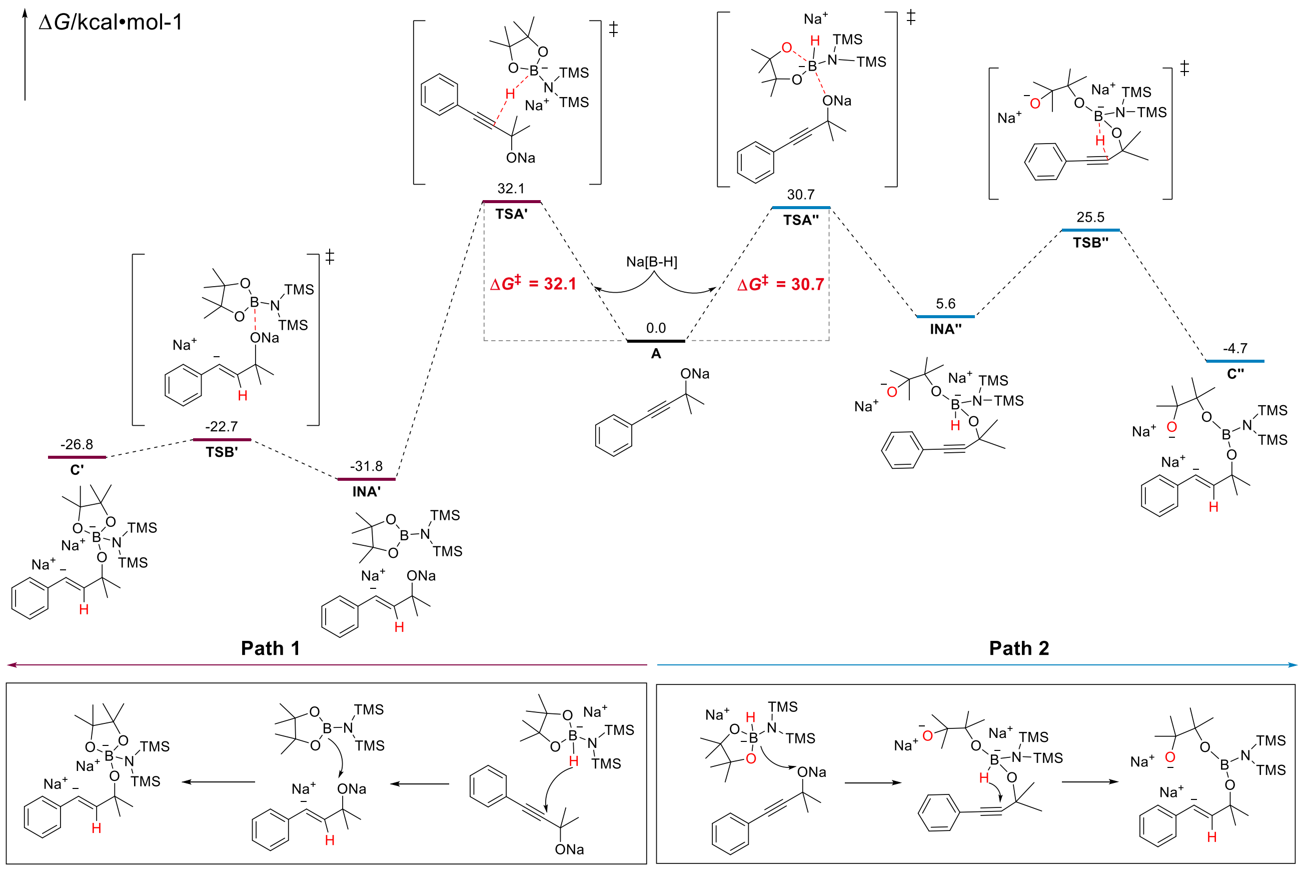

Figure S5. Free energy profiles of direct hydroboration reaction between sodium alkoxide A and Na[HBpin-HMDS] (Na[B–H]), computed at the DFT/B3LYP(D3BJ)/def2-SVP//M06-2X/def2-TZVPP/SMD(THF) level of theory).

5. Characterization

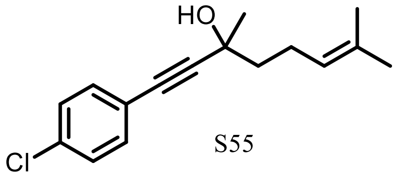

S55

1-(4-chlorophenyl)-3,7-dimethyloct-6-en-1-yn-3-ol

Prepared according to general procedure A: Yield 84%, colorless solid, mp: 67-68 ^{o}C;

^{1}H NMR (500 MHz, $CDCl_3$) δ = 7.49 – 7.42 (m, 2H), 7.36 – 7.29 (m, 3H), 5.24 (t, *J* = 7.5 Hz, 1H), 2.42 – 2.36 (m, 1H), 2.31 – 2.26 (m, 1H), 1.85 – 1.79 (m, 2H), 1.73 (s, 3H), 1.70 (s, 3H), 1.61 (s, 3H) ppm;

^{13}C NMR (126 MHz, $CDCl_3$) δ = 132.5, 131.7, 128.3, 128.2, 123.9, 122.9, 92.8, 83.6, 68.8, 43.5, 30.0, 25.7, 23.9, 17.8 ppm;

HRMS (ESI): *m*/*z* calculated for $C_{16}H_{18}ClO^-$, $[M–H]^-$ = 261.1052, found 261.1051.

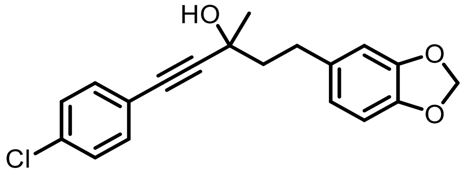

S56

1-(4-chlorophenyl)-5-(6-methoxynaphthalen-2-yl)-3-methylpent-1-yn-3-ol

Prepared according to general procedure A: Yield 87%, colorless solid, mp: 116-117 ^{o}C;

^{1}H NMR (500 MHz, $CDCl_3$) δ = 7.35 – 7.32 (m, 2H), 7.29 – 7.25 (m, 2H), 6.74 – 6.71 (m, 2H), 6.69 – 6.66 (m, 1H), 5.90 (s, 2H), 2.86 – 2.78 (m, 2H), 2.29 (s, 1H), 2.04 – 1.99 (m, 2H), 1.61 (s, 3H) ppm;

^{13}C NMR (126 MHz, $CDCl_3$) δ = 147.7, 145.7, 135.6, 134.4, 132.9, 128.6, 121.1, 108.9, 108.3, 100.8, 93.5, 82.8, 68.4, 60.4, 45.6, 31.1, 30.1 ppm;

HRMS (ESI): *m*/*z* calculated for $C_{19}H_{16}ClO_3^-$, $[M–H]^-$ = 327.0793, found 327.0793.

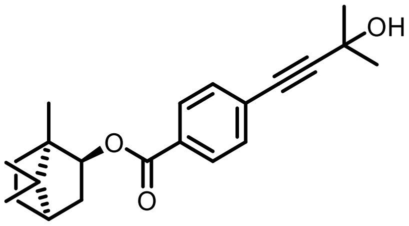

S57

(1R,4R)-1,7,7-trimethylbicyclo[2.2.1]heptan-2-yl 4-(3-hydroxy-3-methylbut-1-yn-1-yl)benzoate

Prepared according to general procedure B: Yield 70%, yellow oil;

^{1}H NMR (500 MHz, $CDCl_3$) δ = 8.02 – 7.98 (m, 2H), 7.51 – 7.48 (m, 2H), 5.15 – 5.11 (m, 1H), 2.53 – 2.46 (m, 1H), 2.16 – 2.11 (m, 2H), 1.85 – 1.81 (m, 1H), 1.78-1.74 (m, 1H), 1.66 (s, 6H), 1.62 (s, 1H), 1.47 – 1.43 (m, 1H), 1.15 – 1.13 (m, 1H), 0.99 (s, 3H), 0.94 (s, 3H), 0.93 (s, 3H) ppm;

^{13}C NMR (126 MHz, $CDCl_3$) δ = 166.3, 131.5, 130.3, 129.3, 127.3, 96.6, 81.5, 80.8, 65.6, 49.1, 47.9, 45.0, 36.9, 31.4, 28.1, 27.4, 19.7, 18.9, 13.6 ppm;
HRMS (EI): *m*/*z* calculated for $C_{22}H_{29}O_3^+$, $[M+H]^+$ = 341.2111, found 341.2111.

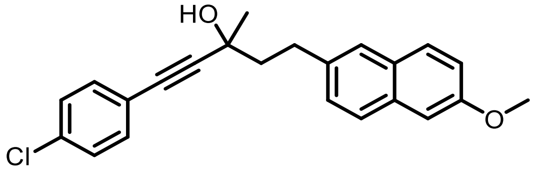

S59

1-(4-chlorophenyl)-5-(6-methoxynaphthalen-2-yl)-3-methylpent-1-yn-3-ol
Prepared according to general procedure A: Yield 91%, yellow solid, mp: 124-125 °C;
^{1}H NMR (500 MHz, $CDCl_3$) δ = 7.69 – 7.64 (m, 2H), 7.60 (s, 1H), 7.38 – 7.26 (m, 5H), 7.14 – 7.10 (m, 2H), 3.91 (s, 3H), 3.09 – 2.98 (m, 2H), 2.17 – 2,11 (m, 2H), 1.65 (s, 3H), 1.57 (s, 1H) ppm;
^{13}C NMR (126 MHz, $CDCl_3$) δ = 157.3, 136.9, 134.4, 133.1, 132.9, 129.2, 128.9, 128.6, 127.8, 126.9, 126.3, 121.2, 118.8, 105.7, 93.5, 82.9, 68.6, 55.3, 45.3, 31.2, 30.1 ppm;
HRMS (EI): *m*/*z* calculated for $C_{23}H_{21}ClO_2$+Na^+, $[M+Na]^+$ = 387.1122, found 387.1122.

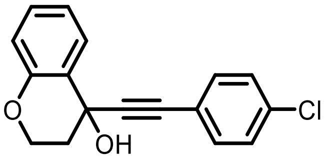

S60

4-((4-chlorophenyl)ethynyl)chroman-4-ol
Prepared according to general procedure A: Yield 85%, colorless solid, mp: 111-112 °C;
^{1}H NMR (500 MHz, $CDCl_3$) δ = 7.83 – 7.76 (m, 1H), 7.50 – 7.42 (m, 2H), 7.40 – 7.35 (m, 2H), 7.33 – 7.29 (m, 1H), 7.08 – 7.03 (m, 1H), 6.97 – 6.91 (m, 1H), 4.49– 4.42 (m, 2H), 2.59 (s, 1H), 2.56 – 2.50 (m, 1H), 2.48 – 2.43 (m, 1H) ppm;
^{13}C NMR (126 MHz, $CDCl_3$) δ = 153.5, 134.7, 133.0, 130.3, 128.7, 128.2, 125.0, 120.9, 120.8, 117.4, 92.4, 83.7, 64.2, 62.4, 37.1 ppm;
HRMS (EI): *m*/*z* calculated for $C_{17}H_{14}ClO_2^+$, $[M+H]^+$ = 285.0677, found 285.0676.

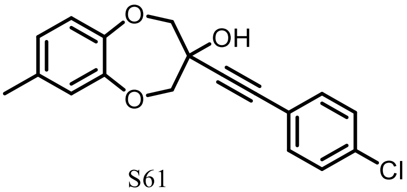

S61

3-((4-chlorophenyl)ethynyl)-7-methyl-3,4-dihydro-2H-benzo[b][1,4]dioxepin-3-ol
Prepared according to general procedure A: Yield 86%, yellow solid, mp: 103-104 °C;
^{1}H NMR (500 MHz, $CDCl_3$) δ = 7.38 – 7.34 (m, 2H), 7.30 – 7.27 (m, 2H), 6.95 – 6.91 (m, 1H), 6.87 (s, 1H), 6.81 – 6.78 (m, 1H), 4.38 – 4.33 (m, 2H), 4.09 – 4.06 (m, 2H), 3.26 (s, 1H), 2.28 (s, 3H) ppm;
^{13}C NMR (126 MHz, $CDCl_3$) δ = 150.8, 148.9, 135.2, 134.4, 133.2, 128.7, 124.9, 122.0, 121.3, 120.1, 86.5, 86.1, 77.8, 77.7, 70.5, 20.6 ppm;
HRMS (ESI): *m*/*z* calculated for $C_{18}H_{14}ClO_3^-$, $[M-H]^-$ = 313.0637, found 313.0638.

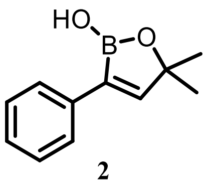

2

5,5-dimethyl-3-phenyl-1,2-oxaborol-2(5*H*)-ol

Prepared according to general procedure C: Yield 86 %, colorless solid, mp: 89-90 °C;

^{1}H NMR (500 MHz, CD_3OD) δ = 7.52 – 7.48 (m, 2H), 7.28 (s, 1H), 7.19 – 7.14 (m, 2H), 7.10 – 7.06 (m, 1H), 1.29 (s, 6H) ppm;

^{13}C NMR (126 MHz, $CDCl_3$) δ = 156.7, 135.9, 128.5, 127.4, 126.9, 83.8, 27.4 ppm;

^{11}B NMR (160 MHz, CD_3OD) δ = 32.3 ppm;

HRMS (ESI): *m*/*z* calculated for $C_{11}H_{12}BO_2^-$, $[M–H]^-$ = 187.0936, found 187.0935.

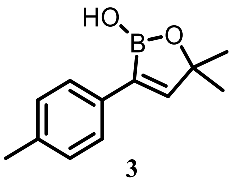

3

5,5-dimethyl-3-(*p*-tolyl)-1,2-oxaborol-2(5*H*)-ol

Prepared according to general procedure C: Yield 71 %, colorless solid, mp:136-137 °C;

^{1}H NMR (500 MHz, CD_3OD) δ = 7.53 – 7.50 (m, 2H), 7.35 (s, 1H), 7.12 – 7.09 (m, 2H), 2.32 (s, 3H), 1.41 (s, 6H) ppm;

^{13}C NMR (126 MHz, CD_3OD) δ = 155.3, 136.6, 133.1, 128.5, 126.6, 83.2, 26.5, 19.8 ppm;

^{11}B NMR (160 MHz, CD_3OD) δ = 31.6 ppm;

HRMS (ESI): *m*/*z* calculated for $C_{12}H_{14}BO_2^-$, $[M–H]^-$ = 201.1092, found 201.1093.

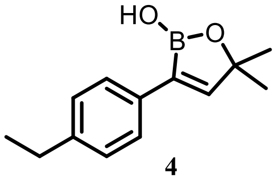

4

3-(4-ethylphenyl)-5,5-dimethyl-1,2-oxaborol-2(5*H*)-ol

Prepared according to general procedure C: Yield 86 %, colorless solid, mp:132-134 °C;

^{1}H NMR (500 MHz, CD_3OD) δ = 7.43 – 7.41 (m, 2H), 7.23 (s, 1H), 7.02 – 7.00 (m, 2H), 2.50 (q, *J* = 7.5 Hz, 2H), 1.29 (s, 6H), 1.11 (t, *J* = 7.5 Hz, 3H) ppm;

^{13}C NMR (126 MHz, CD_3OD) δ = 155.4, 143.2, 133.4, 127.3, 126.7, 83.2, 28.2, 26.5, 14.8 ppm;

^{11}B NMR (160 MHz, CD_3OD) δ = 31.5 ppm;

HRMS (ESI): *m*/*z* calculated for $C_{13}H_{16}BO_2^-$, $[M–H]^-$ = 215.1249, found 215.1249.

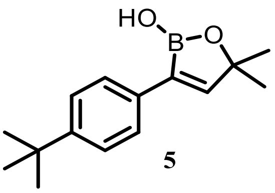

5

3-(4-(*tert*-butyl)phenyl)-5,5-dimethyl-1,2-oxaborol-2(5*H*)-ol

Prepared according to general procedure C: Yield 85%, colorless solid, mp:153-154 ^{o}C;

^{1}H NMR (500 MHz, CD_3OD) δ = 7.56 – 7.52 (m, 2H), 7.33 (s, 1H), 7.31 – 7.29 (m, 2H), 1.38 (s, 6H), 1.29 (s, 9H) ppm;

^{13}C NMR (126 MHz, CD_3OD) δ = 155.5, 149.9, 133.1, 126.4, 124.7, 83.3, 33.9, 30.4, 26.6 ppm;

^{11}B NMR (160 MHz, CD_3OD) δ = 31.5 ppm;

HRMS (ESI): *m*/*z* calculated for $C_{15}H_{20}BO_2^-$, $[M–H]^-$ = 243.1562, found 243.1560.

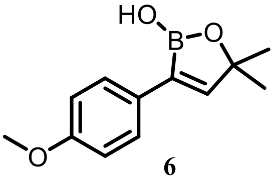

6

3-(4-methoxyphenyl)-5,5-dimethyl-1,2-oxaborol-2(5*H*)-ol

Prepared according to general procedure C: Yield 57%, colorless solid, mp:102-103 ^{o}C;

^{1}H NMR (500 MHz, CD_3OD) δ = 7.59 – 7.56 (m, 2H), 7.27 (s, 1H), 6.86 – 6.83 (m, 2H), 3.79 (s, 3H), 1.40 (s, 6H) ppm;

^{13}C NMR (126 MHz, CD_3OD) δ = 159.1, 154.1, 128.6, 127.8, 113.3, 83.2, 54.2, 26.6 ppm;

^{11}B NMR (160 MHz, CD_3OD) δ = 35.5 ppm;

HRMS (ESI): *m*/*z* calculated for $C_{12}H_{14}BO_3^-$, $[M–H]^-$ = 217.1041, found 217.1041.

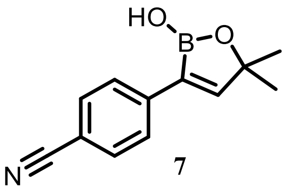

7

4-(2-hydroxy-5,5-dimethyl-2,5-dihydro-1,2-oxaborol-3-yl)benzonitrile

Prepared according to general procedure C: Yield 66%, colorless solid, mp:142-143 ^{o}C;

^{1}H NMR (500 MHz, CD_3OD) δ = 7.81 – 7.79 (m, 2H), 7.66 – 7.63 (m, 2H), 7.62 (s, 1H), 1.43 (s, 6H) ppm;

^{13}C NMR (126 MHz, CD_3OD) δ = 160.0, 140.9, 131.9, 127.5, 118.6, 110.0, 83.7, 26.2 ppm;

^{11}B NMR (160 MHz, CD_3OD) δ= 31.2 ppm;

HRMS (ESI): *m*/*z* calculated for $C_{12}H_{11}BNO_2^-$, $[M–H]^-$ = 212.0888, found 212.0888.

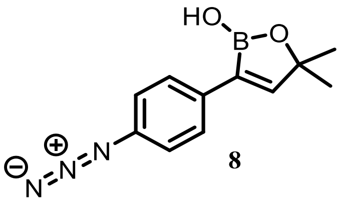

8

3-(4-azidophenyl)-5,5-dimethyl-1,2-oxaborol-2(5*H*)-ol
Prepared according to general procedure C: Yield 40%, yellow solid, mp:122-123 ^oC;
^{1}H NMR (500 MHz, CD_3OD) δ = 7.68 – 7.62 (m, 2H), 7.38 (s, 1H), 7.02 – 6.94 (m, 2H), 1.39 (s, 6H) ppm;
^{13}C NMR (126 MHz, CD_3OD) δ = 156.1, 138.7, 133.1, 128.2, 118.5, 83.4, 26.4 ppm;
^{11}B NMR (160 MHz, CD_3OD) δ = 30.6 ppm;
HRMS (ESI): *m*/*z* calculated for $C_{11}H_{11}BN_3O_2^-$, $[M–H]^-$ = 228.0950, found 228.0952.

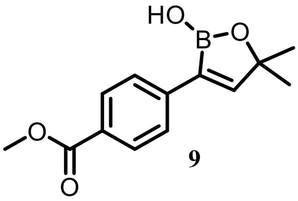

9

Methyl 4-(2-hydroxy-5,5-dimethyl-2,5-dihydro-1,2-oxaborol-3-yl)benzoate
Prepared according to general procedure C: Yield 79%, colorless solid, mp:146-147 ^oC;
^{1}H NMR (500 MHz, CD_3OD) δ = 7.98 – 7.94 (m, 2H), 7.76 – 7.73 (m, 2H), 7.59 (s, 1H), 3.91 (s, 3H), 1.43 (s, 6H) ppm;
^{13}C NMR (126 MHz, CD_3OD) δ = 167.1, 159.0, 129.1, 128.3, 126.6, 83.5, 51.1, 26.2 ppm;
^{11}B NMR (160 MHz, CD_3OD) δ = 31.4 ppm;
HRMS (ESI): *m*/*z* calculated for $C_{13}H_{14}BO_4^-$, $[M–H]^-$ = 245.0991, found 245.0991.

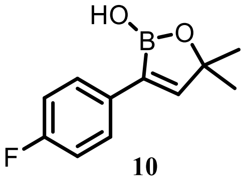

10

3-(4-fluorophenyl)-5,5-dimethyl-1,2-oxaborol-2(5*H*)-ol
Prepared according to general procedure C: Yield 76%, light yellow solid, mp: 121-122 ^oC;
^{1}H NMR (500 MHz, CD_3OD) δ = 7.55 – 7.50 (m, 2H), 7.25 (s, 1H), 6.92 – 6.87 (m, 2H), 1.29 (s, 6H) ppm;
^{13}C NMR (126 MHz, CD_3OD) δ = 162.1 (d, *J* = 243.8 Hz), 156.0 (d, *J* = 2.5 Hz), 132.2 (d, *J* = 3.7 Hz), 128.4 (d, *J* = 7.5 Hz), 114.5 (d, *J* = 21.2 Hz), 83.3, 26.4 ppm;
^{11}B NMR (160 MHz, CD_3OD) δ = 31.4 ppm;
HRMS (ESI): *m*/*z* calculated for $C_{11}H_{11}BFO_2^-$, $[M–H]^-$ = 205.0842, found 205.0844.

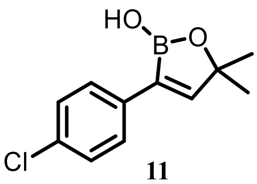

11

3-(4-chlorophenyl)-5,5-dimethyl-1,2-oxaborol-2(5*H*)-ol
Prepared according to general procedure C: Yield 95%, yellow solid, mp: 130-132 ºC;
^{1}H NMR (500 MHz, CD_3OD) δ= 7.52 – 7.48 (m, 2H), 7.33 (s, 1H), 7.19 – 7.15 (m, 2H), 1.30 (s, 6H) ppm;
^{13}C NMR (126 MHz, CD_3OD) δ =156.9, 134.6, 132.5, 128.1, 127.9, 83.4, 26.3 ppm;
^{11}B NMR (160 MHz, CD_3OD) δ =29.9 ppm;
HRMS (ESI): *m*/*z* calculated for $C_{11}H_{11}ClO_2^-$, $[M–H]^-$ = 221.0546, found 221.0546.

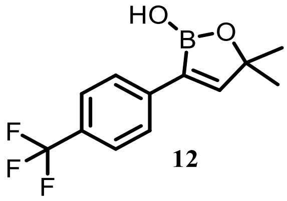

12

5,5-dimethyl-3-(4-(trifluoromethyl)phenyl)-1,2-oxaborol-2(5*H*)-ol
Prepared according to general procedure C: Yield 99%, colorless solid, mp: 172-173 ºC;
^{1}H NMR (500 MHz, CD_3OD) δ = 7.82 – 7.79 (m, 2H), 7.61 (s, 1H), 7.59 (s, 2H), 1.44 (s, 6H) ppm;
^{13}C NMR (126 MHz, CD_3OD) δ = 158.9, 139.8, 128.5 (q, *J* = 32.5 Hz), 127.0, 124.7 (q, *J* = 3.8 Hz), 124.4 (q, *J* = 270 Hz), 83.5, 26.2 ppm;
^{11}B NMR (160 MHz, CD_3OD) δ = 31.3 ppm;
HRMS (ESI): *m*/*z* calculated for $C_{12}H_{11}BF_3O_2^-$, $[M–H]^-$ = 255.0810, found 255.0812.

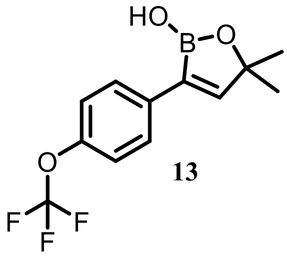

13

5,5-dimethyl-3-(4-(trifluoromethoxy)phenyl)-1,2-oxaborol-2(5*H*)-ol
Prepared according to general procedure C: Yield 78%, colorless solid, mp: 116-117 ºC;
^{1}H NMR (500 MHz, CD_3OD) δ = 7.62 – 7.54 (m, 2H), 7.33 (s, 1H), 7.12 – 7.01 (m, 2H), 1.30 (s, 6H) ppm;
^{13}C NMR (126 MHz, CD_3OD) δ = 157.4, 148.1 (d, *J* = 1.0 Hz), 135.1, 128.2, 120.4, 120.5 (q, *J* = 253.7 Hz), 83.4, 26.4 ppm;
^{11}B NMR (160 MHz, CD_3OD) δ = 31.4 ppm;
HRMS (ESI): *m*/*z* calculated for $C_{12}H_{11}BF_3O_3^-$, $[M–H]^-$ = 271.0759, found 271.0759.

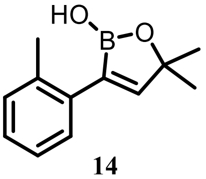

14

5,5-dimethyl-3-(*o*-tolyl)-1,2-oxaborol-2(5*H*)-ol

Prepared according to general procedure C: Yield 51%, colorless solid, mp: 108-109 ºC;
^{1}H NMR (500 MHz, CD_3OD) δ = 7.04 – 6.96 (m, 4H), 6.94 (s, 1H), 2.18 (s, 3H), 1.32 (s, 6H) ppm;
^{13}C NMR (126 MHz, CD_3OD) δ = 160.3, 136.8, 135.0, 129.5, 128.1, 126.3, 125.1, 83.6, 26.4, 19.3 ppm;
^{11}B NMR (160 MHz, CD_3OD) δ = 31.2 ppm;
HRMS (ESI): *m/z* calculated for $C_{12}H_{14}BO_2^-$, $[M–H]^-$ = 201.1092, found 201.1094.

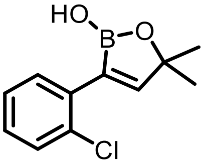

15

3-(2-chlorophenyl)-5,5-dimethyl-1,2-oxaborol-2(5*H*)-ol

Prepared according to general procedure C: Yield 96%, colorless solid, mp: 96-97 ºC;
^{1}H NMR (500 MHz, CD_3OD) δ = 7.28 – 7.20 (m, 3H), 7.13 – 7.04 (m, 2H), 1.32 (s, 6H) ppm;
^{13}C NMR (126 MHz, CD_3OD) δ = 161.8, 135.6, 132.1, 130.1, 129.2, 127.6, 126.3, 83.7, 26.2.ppm
^{11}B NMR (160 MHz, CD_3OD) δ = 34.9 ppm;
HRMS (ESI): *m/z* calculated for $C_{11}H_{11}BClO_2^-$, $[M–H]^-$ = 221.0546, found 221.0546.

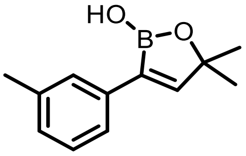

16

5,5-dimethyl-3-(*m*-tolyl)-1,2-oxaborol-2(5*H*)-ol

Prepared according to general procedure C: Yield 84%, colorless solid, mp: 114-115 ºC;
^{1}H NMR (500 MHz, CD_3OD) δ = 7.43 (s, 1H), 7.40 – 7.37 (m, 1H), 7.35 (s, 1H), 7.17 (t, *J* = 7.5 Hz, 1H), 7.01 (d, *J* = 6.5 Hz, 1H), 2.31 (s, 3H), 1.39 (s, 6H) ppm;
^{13}C NMR (126 MHz, CD_3OD) δ = 156.1, 137.3, 135.8, 127.7, 127.5, 127.2, 123.7, 83.2, 26.4, 20.1 ppm;
^{11}B NMR (160 MHz, CD_3OD) δ = 31.5 ppm;
HRMS (ESI): *m/z* calculated for $C_{12}H_{14}BO_2^-$, $[M–H]^-$ = 210.1092, found 210.1091.

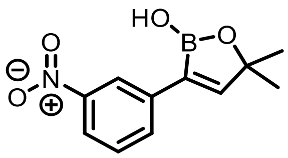

17

5,5-dimethyl-3-(3-nitrophenyl)-1,2-oxaborol-2(5*H*)-ol

Prepared according to general procedure C: Yield 68%, light yellow solid, mp: 149-150 ^{o}C;

^{1}H NMR (500 MHz, CD_2Cl_2) δ = 8.38 (s, 1H), 8.03 – 7.97 (m, 1H), 7.91 – 7.87 (m, 1H), 7.47 – 7.44 (m, 1H), 7.43 (s, 1H), 5.17 (s, 1H), 1.36 (s, 6H) ppm;

^{13}C NMR (126 MHz, CD_2Cl_2) δ = 159.7, 148.5, 137.7, 132.9, 129.4, 121.8, 121.5, 83.9, 26.8 ppm;

^{11}B NMR (160 MHz, CD_2Cl_2) δ = 32.0 ppm;

HRMS (ESI): *m*/*z* calculated for $C_{11}H_{11}BNO_4^-$, $[M–H]^-$ = 232.0787, found 232.0787.

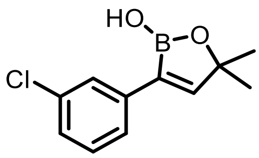

18

3-(3-chlorophenyl)-5,5-dimethyl-1,2-oxaborol-2(5*H*)-ol

Prepared according to general procedure C: Yield 95%, colorless solid, mp: 116-117 ^{o}C;

^{1}H NMR (500 MHz, CD_3OD) δ = 7.64 (s, 1H), 7.57 – 7.54 (m, 1H), 7.48 (s, 1H), 7.29 – 7.26 (m, 1H), 7.23 – 7.20 (m, 1H), 1.42 (s, 6H) ppm;

^{13}C NMR (126 MHz, CD_3OD) δ = 157.8, 138.0, 133.9, 129.4, 126.7, 126.6, 124.8, 83.5, 26.3 ppm;

^{11}B NMR (160 MHz, CD_3OD) δ = 31.2 ppm;

HRMS (ESI): *m*/*z* calculated for $C_{11}H_{11}BClO_2^-$, $[M–H]^-$ = 221.0546, found 221.0546.

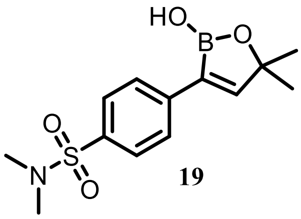

19

4-(2-hydroxy-5,5-dimethyl-2,5-dihydro-1,2-oxaborol-3-yl)-*N*,*N*-dimethylbenzenesulfonamide

Prepared according to general procedure C: Yield 68%, colorless solid, mp: 190-191 ^{o}C;

^{1}H NMR (500 MHz, CD_3OD) δ = 7.87 – 7.80 (m, 2H), 7.73 – 7.66 (m, 2H), 7.62 (s, 1H), 2.67 (s, 6H), 1.42 (s, 6H) ppm;

^{13}C NMR (126 MHz, CD_3OD) δ = 159.8, 140.8, 133.2, 127.6, 127.2, 83.7, 36.9, 26.2 ppm;

^{11}B NMR (160 MHz, CD_3OD) δ = 31.52 ppm;

HRMS (ESI): *m*/*z* calculated for $C_{13}H_{17}BNO_4S^-$, $[M–H]^-$ = 294.0977, found 294.0977.

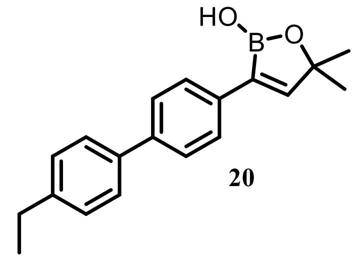

20

3-(4'-ethyl-[1,1'-biphenyl]-4-yl)-5,5-dimethyl-1,2-oxaborol-2(5*H*)-ol

Prepared according to general procedure C: Yield 60%, colorless solid, mp: 191-193 ^{o}C;

^{1}H NMR (500 MHz, CD_2Cl_2) δ = 7.75 – 7.69 (m, 2H), 7.65 – 7.56 (m, 4H), 7.42 (s, 1H), 7.35 – 7.29 (m, 2H), 5.39 (s, 1H, -OH), 2.73 (q, *J* = 7.5 Hz, 2H), 1.48 (s, 6H), 1.30 (t, *J* = 7.5 Hz, 3H) ppm;

^{13}C NMR (126 MHz, CD_2Cl_2) δ = 157.1, 143.6, 139.8, 138.0, 134.8, 128.2, 127.2, 126.8, 126.6, 83.6, 28.4, 27.1, 15.4 ppm;

^{11}B NMR (160 MHz, CD_2Cl_2) δ = 33.3 ppm;

HRMS (ESI): *m*/*z* calculated for $C_{19}H_{20}BO_2^-$, $[M–H]^-$ = 291.1562, found 291.1560.

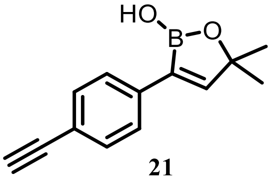

21

3-(4-ethynylphenyl)-5,5-dimethyl-1,2-oxaborol-2(5*H*)-ol

Prepared according to general procedure C: Yield 71%, colorless solid, mp: 121-122 ^{o}C;

^{1}H NMR (500 MHz, CD_3OD) δ = 7.63 – 7.61 (m, 2H), 7.47 (s, 1H), 7.41 – 7.38 (m, 2H), 3.48 (s, 1H), 1.42 (s, 6H) ppm;

^{13}C NMR (126 MHz, CD_3OD) δ = 157.4, 136.4, 131.5, 126.6, 120.9, 83.4, 83.1, 77.4, 26.3 ppm;

^{11}B NMR (160 MHz, CD_3OD) δ = 31.4 ppm;

HRMS (ESI): *m*/*z* calculated for $C_{13}H_{12}BO_2^-$, $[M–H]^-$ = 211.0936, found 211.0937.

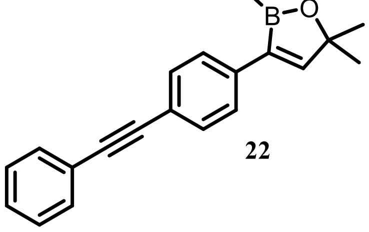

22

5,5-dimethyl-3-(4-(phenylethynyl)phenyl)-1,2-oxaborol-2(5*H*)-ol

Prepared according to general procedure C: Yield 85%, colorless solid, mp: 214-215 ^{o}C;

^{1}H NMR (500 MHz, CD_2Cl_2) δ = 7.52 – 7.49 (m, 2H), 7.42 – 7.37 (m, 4H), 7.27 (s, 1H), 7.25 – 7.21 (m, 3H), 5.08 (s, 1H, OH), 1.30 (s, 6H) ppm;

^{13}C NMR (126 MHz, CD_2Cl_2) δ = 158.1, 136.1, 131.6, 131.4, 128.4, 128.2, 126.9, 123.2, 121.9, 89.6, 89.4, 83.6, 27.0 ppm;

^{11}B NMR (160 MHz, CD_2Cl_2) δ = 33.1 ppm;

HRMS (ESI): *m*/*z* calculated for $C_{19}H_{16}BO_2^-$, $[M–H]^-$ = 287.1249, found 287.1249.

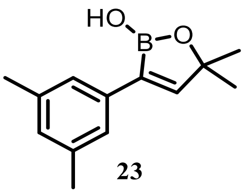
23

3-(3,5-dimethylphenyl)-5,5-dimethyl-1,2-oxaborol-2(5*H*)-ol

Prepared according to general procedure C: Yield 62%, colorless solid, mp: 109-111 ^oC;

^{1}H NMR (500 MHz, CD_3OD) δ = 7.22 (s, 1H), 7.12 (s, 2H), 6.74 (s, 1H), 2.16 (s, 6H), 1.28 (s, 6H) ppm;

^{13}C NMR (126 MHz, CD_3OD) δ = 156.0, 137.2, 135.7, 128.3, 124.4, 83.1, 26.5, 20.0 ppm;

^{11}B NMR (160 MHz, CD_3OD) δ = 31.5 ppm;

HRMS (ESI): *m*/*z* calculated for $C_{13}H_{16}BO_2^-$, $[M–H]^-$ = 215.1249, found 215.1249.

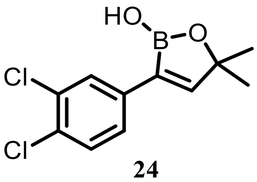
24

3-(3,4-dichlorophenyl)-5,5-dimethyl-1,2-oxaborol-2(5*H*)-ol

Prepared according to general procedure C: Yield 98%, colorless solid, mp: 123-124 ^oC;

^{1}H NMR (500 MHz, CD_3OD) δ = 7.65 (s, 1H), 7.42 – 7.40 (m,1H), 7.37 (s, 1H), 7.30 – 7.28 (m,1H), 1.29 (s, 6H) ppm;

^{13}C NMR (126 MHz, CD_3OD) δ = 158.2, 136.4, 131.8, 130.3, 129.9, 128.5, 126.2, 83.5, 26.2 ppm;

^{11}B NMR (160 MHz, CD_3OD) δ = 31.1 ppm;

HRMS (ESI): *m*/*z* calculated for $C_{11}H_{10}BCl_2O_2^-$, $[M–H]^-$ = 255.0156, found 255.0154.

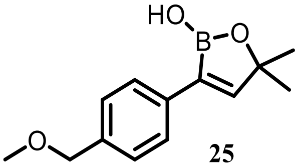
25

3-(4-(methoxymethyl)phenyl)-5,5-dimethyl-1,2-oxaborol-2(5*H*)-ol

Prepared according to general procedure C: Yield 60%, colorless solid, mp: 111-113 ^oC;

^{1}H NMR (500 MHz, CD_3OD) δ = 7.52 – 7.49 (m, 2H), 7.41 (s, 1H), 7.17 – 7.13 (m, 2H), 4.31 (s, 2H), 3.25 (s, 3H), 1.29 (s, 6H) ppm;

^{13}C NMR (126 MHz, CD_3OD) δ = 156.4, 136.8, 135.5, 127.5, 126.6, 83.3, 74.1, 56.8, 26.4 ppm;

^{11}B NMR (160 MHz, CD_3OD) δ = 31.4 ppm;

HRMS (ESI): *m*/*z* calculated for $C_{13}H_{16}BO_3^-$, $[M–H]^-$ = 231.1198, found 231.1198.

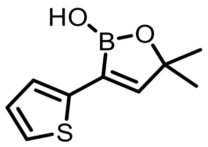

26

5,5-dimethyl-3-(thiophen-2-yl)-1,2-oxaborol-2(5*H*)-ol
Prepared according to general procedure C: Yield 96%, light yellow solid, mp: 130-131 ^{o}C;
^{1}H NMR (500 MHz, CD_3OD) δ = 7.27 – 7.21 (m, 2H), 7.15 (s, 1H), 6.97 – 6.92 (m, 1H), 1.38 (s, 6H) ppm;
^{13}C NMR (126 MHz, CD_3OD) δ = 153.9, 139.4, 126.8, 126.0, 124.1, 83.7, 26.4 ppm;
^{11}B NMR (160 MHz, CD_3OD) δ = 30.9 ppm;
HRMS (ESI): *m*/*z* calculated for $C_9H_{10}BO_2S^–$, $[M–H]^–$ = 193.0500, found 193.0500.

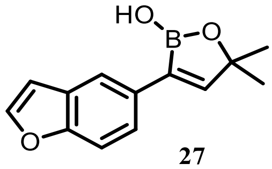

27

3-(benzofuran-5-yl)-5,5-dimethyl-1,2-oxaborol-2(5*H*)-ol
Prepared according to general procedure C: Yield 62%, colorless solid, mp: 140-142 ^{o}C;
^{1}H NMR (500 MHz, $CDCl_3$) δ = 7.94 (s, 1H), 7.64 – 7.60 (m, 2H), 7.51 – 7.48 (m, 1H), 7.32 (s, 1H), 6.80 (s, 1H), 5.90 (s, 1H, OH), 1.50 (s, 6H) ppm;
^{13}C NMR (126 MHz, $CDCl_3$) δ = 155.7, 154.5, 145.2, 130.8, 127.7, 123.3, 119.8, 111.3, 106.8, 83.8, 27.4 ppm;
^{11}B NMR (160 MHz, $CDCl_3$) δ = 33.1 ppm;
HRMS (ESI): *m*/*z* calculated for $C_{13}H_{12}BO_3^–$, $[M–H]^–$ = 227.0885, found 227.0884.

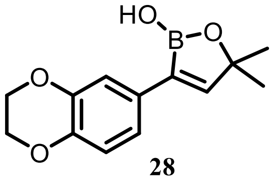

28

3-(2,3-dihydrobenzo[*b*][1,4]dioxin-6-yl)-5,5-dimethyl-1,2-oxaborol-2(5*H*)-ol
Prepared according to general procedure C: Yield 61%, colorless solid, mp: 112-113 ^{o}C;
^{1}H NMR (500 MHz, CD_3OD) δ = 7.23 (s, 1H), 7.13 – 7.09 (m, 2H), 6.75 – 6.69 (m, 1H), 4.21 (s, 4H), 1.37 (s, 6H) ppm;
^{13}C NMR (126 MHz, CD_3OD) δ = 154.5, 143.3, 143.0, 129.4, 119.8, 116.5, 115.4, 83.1, 64.2, 64.1, 26.5 ppm;
^{11}B NMR (160 MHz, CD_3OD) δ = 31.6 ppm;
HRMS (ESI): m/z calculated for C13H14BO4$^–$, $[M–H]^–$ = 245.0991, found 245.0991.

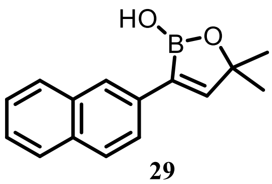

29

5,5-dimethyl-3-(naphthalen-2-yl)-1,2-oxaborol-2(5*H*)-ol

Prepared according to general procedure C: Yield 93%, colorless solid, mp: 153-154 °C;

^{1}H NMR (500 MHz, CD_3OD) δ = 8.12 (s, 1H), 7.81 – 7.71 (m, 4H), 7.52 (s, 1H), 7.45 – 7.37 (m, 2H), 1.43 (s, 6H) ppm;

^{13}C NMR (126 MHz, CD_3OD) δ = 156.7, 133.7, 133.3, 132.8, 127.7, 127.4, 127.1, 126.0, 125.6, 125.3, 124.4, 83.4, 26.4 ppm;

^{11}B NMR (160 MHz, CD_3OD) δ = 35.6 ppm;

HRMS (ESI): *m/z* calculated for $C_{15}H_{14}BO_2^-$, $[M–H]^-$ = 237.1092, found 237.1092.

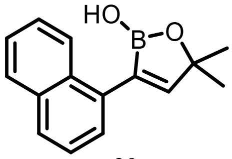

30

5,5-dimethyl-3-(naphthalen-1-yl)-1,2-oxaborol-2(5*H*)-ol

Prepared according to general procedure C: Yield 85%, colorless solid, mp: 148-149 °C;

^{1}H NMR (500 MHz, CD_3OD) δ = 8.02 – 7.98 (m, 1H), 7.85 – 7.80 (m, 1H), 7.76 – 7.71 (m, 1H), 7.47 – 7.38 (m, 3H), 7.33 – 7.29 (m 1H), 7.22 (s, 1H), 1.49 (s, 6H) ppm;

^{13}C NMR (126 MHz, CD_3OD) δ = 161.7, 135.2, 133.8, 131.2, 127.8, 126.7, 125.3, 125.2, 125.1, 125.0, 83.9, 26.4 ppm;

^{11}B NMR (160 MHz, CD_3OD) δ = 31.4 ppm;

HRMS (ESI): *m/z* calculated for $C_{15}H_{14}BO_2^-$, $[M–H]^-$ = 237.1092, found 237.1091.

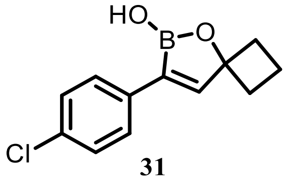

31

7-(4-chlorophenyl)-5-oxa-6-boraspiro[3.4]oct-7-en-6-ol

Prepared according to general procedure C: Yield 71%, colorless solid, mp: 158-160 °C;

^{1}H NMR (500 MHz, CD_2Cl_2) δ = 7.40 – 7.26 (m, 3H), 7.13 – 6.97 (m, 2H), 5.18 (s, 1H, -OH), 2.23 – 2.16 (m, 2H), 2.05 – 1.98 (m, 2H), 1.67 – 1.60 (m, 1H), 1.59 – 1.51 (m, 1H) ppm;

^{13}C NMR (126 MHz, CD_2Cl_2) δ = 153.6, 134.5, 132.8, 128.5, 128.2, 85.9, 33.1, 12.3 ppm;

^{11}B NMR (160 MHz, CD_2Cl_2) δ = 31.9 ppm;

HRMS (ESI): *m/z* calculated for $C_{12}H_{11}BClO_2^-$, $[M–H]^-$ = 233.0546, found 233.0545.

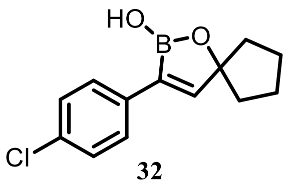

32

3-(4-chlorophenyl)-1-oxa-2-boraspiro[4.4]non-3-en-2-ol

Prepared according to general procedure C: Yield 68%, colorless solid, mp: 146-148 ºC;

^{1}H NMR (500 MHz, CD_2Cl_2) δ = 7.54 – 7.48 (m, 2H), 7.25 – 7.18 (m, 3H), 6.03 (s, 1H, -OH), 1.91 – 1.82 (m, 4H), 1.78 – 1.71 (m, 2H), 1.66 – 1.60 (m, 2H) ppm;

^{13}C NMR (126 MHz, CD_2Cl_2) δ = 154.9, 134.6, 132.8, 128.5, 128.2, 94.6, 38.0, 24.5 ppm;

^{11}B NMR (160 MHz, CD_2Cl_2) δ = 31.9 ppm;

HRMS (ESI): *m/z* calculated for $C_{13}H_{13}BClO_2^-$, $[M–H]^-$ = 247.0703, found 247.0703.

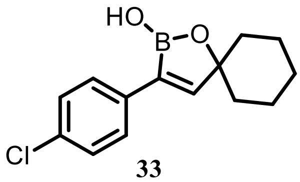

33

3-phenyl-1-oxa-2-boraspiro[4.5]dec-3-en-2-ol

Prepared according to general procedure C: Yield 92%, colorless solid, mp: 179-181 ºC;

^{1}H NMR (500 MHz, CD_2Cl_2) δ = 7.66 – 7.63 (m, 2H), 7.51 (s, 1H), 7.38 – 7.33 (m, 2H), 6.17 (s, 1H, -OH), 1.81 – 1.62 (m, 10H) ppm;

^{13}C NMR (126 MHz, CD_2Cl_2) δ = 156.4, 134.7, 132.8, 128.5, 128.2, 85.6, 36.4, 25.1, 22.9 ppm;

^{11}B NMR (160 MHz, CD_2Cl_2) δ = 31.7 ppm;

HRMS (ESI): *m/z* calculated for $C_{14}H_{15}BClO_2^-$, $[M–H]^-$ = 261.0859, found 261.0860.

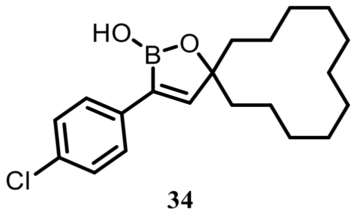

34

3-(4-chlorophenyl)-1-oxa-2-boraspiro[4.11]hexadec-3-en-2-ol

Prepared according to general procedure C: Yield 90%, colorless solid, mp: 142-143 ºC;

^{1}H NMR (500 MHz, CD_2Cl_2) δ = 7.50 – 7.46 (m, 2H), 7.40 (s, 1H), 7.25 – 7.19 (m, 2H), 4.82 (s, 1H, -OH), 1.65 – 1.60 (m, 2H), 1.44 – 1.40 (m, 3H), 1.35 – 1.17 (m, 17H) ppm;

^{13}C NMR (126 MHz, CD_2Cl_2) δ = 157.3, 134.9, 132.7, 128.5, 128.2, 88.5, 33.7, 26.4, 26.0, 22.5, 22.1, 20.4 ppm;

^{11}B NMR (160 MHz, CD_2Cl_2) δ = 28.2 ppm;

HRMS (ESI): *m/z* calculated for $C_{20}H_{27}BClO_2^-$, $[M–H]^-$ = 345.1798, found 345.1798.

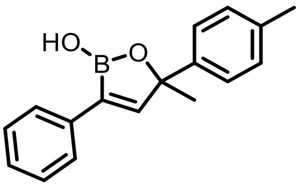

35

5-methyl-3-phenyl-5-(*p*-tolyl)-1,2-oxaborol-2(5*H*)-ol
Prepared according to general procedure C: Yield 80%, colorless solid, mp: 114-115 ^oC;
^{1}H NMR (500 MHz, CD_3OD) δ = 7.55 – 7.49 (m, 3H), 7.27 – 7.23 (m, 2H), 7.19 – 7.14 (m, 2H), 7.10 – 7.08 (m, 1H), 7.07 – 7.03 (m, 2H), 2.20 (s, 3H), 1.59 (s, 3H) ppm;
^{13}C NMR (126 MHz, CD_3OD) δ = 155.6, 140.7, 136.3, 135.7, 128.5, 127.9, 127.0, 126.7, 124.4, 86.1, 27.7, 19.6 ppm;
^{11}B NMR (160 MHz, CD_3OD) δ = 32.0 ppm;
HRMS (ESI): *m*/*z* calculated for $C_{17}H_{15}BO_2^-$, $[M–H]^-$ = 263.1249, found 263.1247.

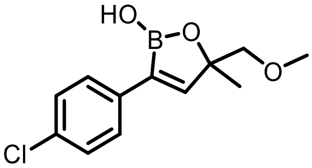

36

3-(4-chlorophenyl)-5-(methoxymethyl)-5-methyl-1,2-oxaborol-2(5*H*)-ol
Prepared according to general procedure C: Yield 67%, colorless solid, mp: 156-157 ^oC;
^{1}H NMR (500 MHz, CD_3OD) δ = 7.66 – 7.60 (m, 2H), 7.41 (s, 1H), 7.33 – 7.27 (m, 2H), 3.47 (s, 2H), 3.40 (s, 3H), 1.39 (s, 3H) ppm;
^{13}C NMR (126 MHz, CD_3OD) δ = 153.3, 134.4, 132.7, 128.2, 128.0, 85.3, 78.1, 58.5, 21.6 ppm;
^{11}B NMR (160 MHz, CD_3OD) δ = 31.6 ppm;
HRMS (ESI): *m*/*z* calculated for $C_{12}H_{13}BClO_3^-$, $[M–H]^-$ = 251.0652, found 251.0650.

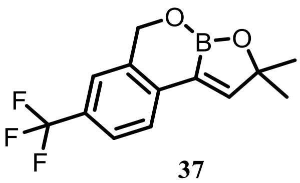

37

3-(2-(hydroxymethyl)-4-(trifluoromethyl)phenyl)-5,5-dimethyl-1,2-oxaborol-2(5*H*)-ol
Prepared according to general procedure C: Yield 76%, colorless solid, mp: 86-87 ^oC;
^{1}H NMR (500 MHz, $CDCl_3$) δ = 7.64 – 7.61 (m, 1H), 7.51 – 7.48 (m, 1H), 7.34 (s, 1H), 7.14 (s, 1H), 5.42 (s, 2H), 1.48 (s, 6H) ppm;
^{13}C NMR (126 MHz, $CDCl_3$) δ = 150.4, 137.2, 134.2, 129.9 (q, *J* = 31.2 Hz), 125.3, 124.1 (q, *J* = 3.7 Hz), 123.9 (q, *J* = 270 Hz), 122.1 (q, *J* = 3.7 Hz), 88.4, 69.1, 27.3 ppm;
^{11}B NMR (160 MHz, $CDCl_3$) δ = 33.1 ppm;
HRMS (ESI): *m*/*z* calculated for $C_{13}H_{13}BF_3O_2^+$, $[M+H]^+$ = 269.0955, found 269.0955.

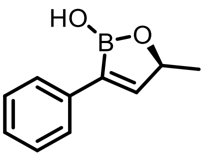

38

5-methyl-3-phenyl-1,2-oxaborol-2(5*H*)-ol

Prepared according to general procedure C: Yield 81%, light yellow solid, mp: 71-72 ºC;
^{1}H NMR (500 MHz, CD_3OD) δ = 7.63 – 7.60 (m, 2H), 7.40 (s, 1H), 7.29 – 7.25 (m, 2H), 7.21 – 7.17 (m, 1H), 4.87 (q, *J* = 7.0 Hz, 1H), 1.33 (d, *J* = 7.0 Hz, 3H) ppm;
^{13}C NMR (126 MHz, CD_3OD) δ = 152.1, 135.8, 127.9, 126.9, 126.6, 78.0, 20.0 ppm;
^{11}B NMR (160 MHz, CD_3OD) δ = 31.7 ppm;
HRMS (ESI): *m/z* calculated for $C_{10}H_{10}BO_2^-$, $[M–H]^-$ = 173.0779, found 173.0779.

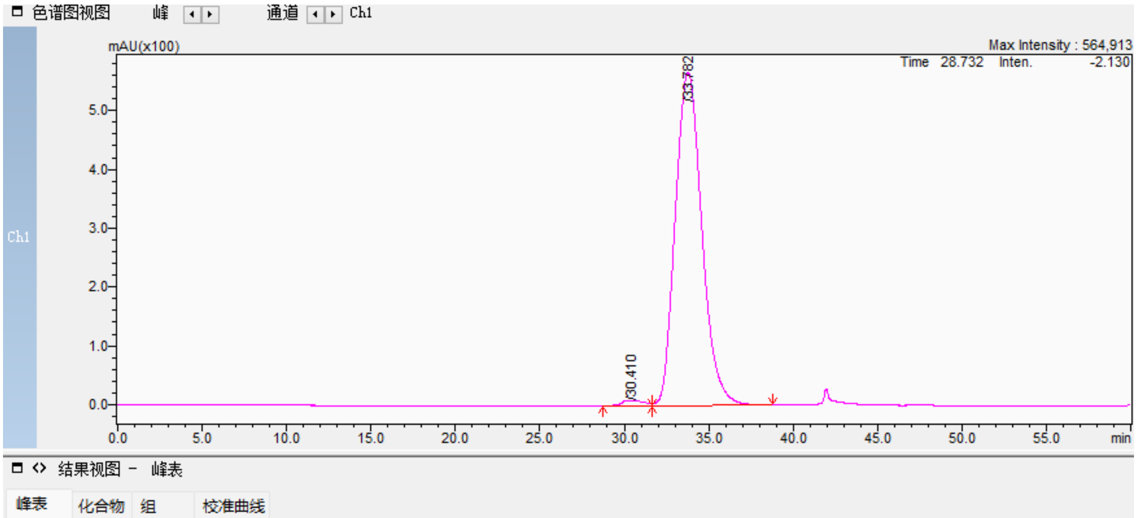

峰号	保留时间	面积	高度	标记	浓度	浓度单位	化合物ID号	化合物名	面积%
1	30. 410	837877	9317		0. 000				1. 332
2	33. 782	62050116	566543	V	0. 000				98. 668
总计		62887993	575860		0. 000				100. 000

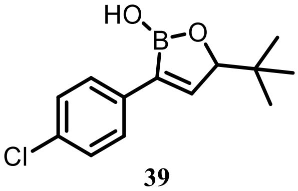

39

5-(tert-butyl)-3-(4-chlorophenyl)-1,2-oxaborol-2(5H)-ol

Prepared according to general procedure C: Yield 70%, colorless solid, mp: 105-106 ºC;
^{1}H NMR (500 MHz, CD_3OD) δ = 7.54 – 7.50 (m, 2H), 7.41 (s, 1H), 7.20 – 7.16 (m, 2H), 4.47 (s, 1H), 4.38 (s, 1H), 0.87 (s, 9H) ppm;
^{13}C NMR (126 MHz, CD_3OD) δ = 149.3, 134.6, 132.6, 128.1, 128.0, 89.4, 34.4, 24.7 ppm;
^{11}B NMR (160 MHz, CD_3OD) δ = 32.5 ppm;
HRMS (ESI): *m/z* calculated for $C_{13}H_{15}BClO_2^-$, $[M–H]^-$ = 249.0859, found 249.0860.

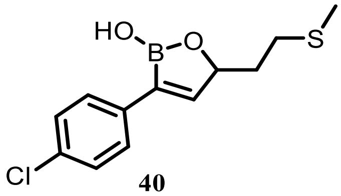

40

3-(4-chlorophenyl)-5-(2-(methylthio)ethyl)-1,2-oxaborol-2(5*H*)-ol
Prepared according to general procedure C: Yield 74%, colorless solid, mp: 134-136°C;
^{1}H NMR (500 MHz, CD_2Cl_2) δ = 7.53 – 7.47 (m, 2H), 7.30 (s, 1H), 7.25 – 7.19 (m, 2H), 5.41 (s, 1H, -OH), 4.88 – 4.82 (m, 1H), 2.59 – 2.51 (m, 2H), 2.03 (s, 3H), 1.95 – 1.90 (m, 1H), 1.74 – 1.68 (m, 1H) ppm;
^{13}C NMR (126 MHz, CD_2Cl_2) δ = 151.7, 134.3, 133.0, 128.5, 128.2, 80.5, 34.6, 30.0, 15.3 ppm;
^{11}B NMR (160 MHz, CD_2Cl_2) δ = 31.8 ppm;
HRMS (ESI): *m*/*z* calculated for $C_{12}H_{13}BClO_2S^-$, $[M–H]^-$ = 267.0423, found 267.0423.

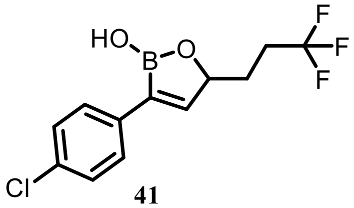

41

3-(4-chlorophenyl)-5-(3,3,3-trifluoropropyl)-1,2-oxaborol-2(5*H*)-ol
Prepared according to general procedure C: Yield 85%, colorless solid, mp: 118-119 ^{o}C;
^{1}H NMR (500 MHz, CD_3OD) δ = 7.67 – 7.62 (m, 2H), 7.47 (s, 1H), 7.34 – 7.27 (m, 2H), 4.87 (t, *J* = 6.5 Hz, 1H), 2.39 – 2.28 (m, 2H), 2.11 – 2.04 (m, 1H), 1.73 – 1.65 (m, 1H) ppm;
^{13}C NMR (126 MHz, CD_3OD) δ = 150.2, 134.3, 132.9, 128.2, 128.1, 127.4 (q, *J* = 273.7 Hz), 80.0, 29.2 (q, *J* = 28.7 Hz), 27.3 (q, *J* = 3.7 Hz) ppm;
^{11}B NMR (160 MHz, CD_3OD) δ = 32.5 ppm;
HRMS (ESI): *m*/*z* calculated for $C_{12}H_{10}BClF_3O_2^-$, $[M–H]^-$ = 289.0420, found 289.0422.

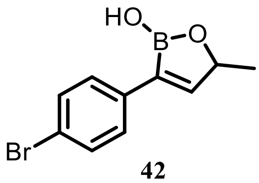

42

3-(4-bromophenyl)-5-methyl-1,2-oxaborol-2(5*H*)-ol
Prepared according to general procedure C: Yield 83%, colorless solid, mp: 114-116 ^{o}C;
^{1}H NMR (500 MHz, CD_3OD) δ = 7.56 – 7.53 (m, 2H), 7.46 (s, 1H), 7.44 – 7.41 (m, 2H), 4.88 (q, *J* = 6.5 Hz, 1H), 1.33 (d, *J* = 6.5 Hz, 3H) ppm;
^{13}C NMR (126 MHz, CD_3OD) δ = 153.0, 135.0, 131.0, 128.4, 120.6, 78.1, 19.9 ppm;
^{11}B NMR (160 MHz, CD_3OD) δ = 31.8 ppm;
HRMS (ESI): *m*/*z* calculated for $C_{10}H_9BBrO_2^-$, $[M–H]^-$ = 250.9884, found 250.9885.

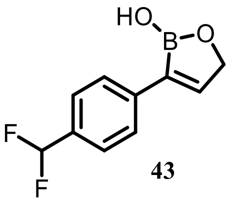

43

3-(4-(difluoromethyl)phenyl)-5-methyl-1,2-oxaborol-2(5*H*)-ol
Prepared according to general procedure C: Yield 51%, colorless solid, mp: 109-111 ^{o}C;
^{1}H NMR (500 MHz, CD_2Cl_2) δ = 7.81 – 7.74 (m, 2H), 7.58 (s, 1H), 7.55 – 7.50 (m, 2H), 6.71 (t, *J* = 6.5 Hz, 1H), 4.74 (s, 2H), 1.64 (s, 1H, -OH) ppm;
^{13}C NMR (126 MHz, $CDCl_3$) δ = 151.7, 140.4, 135.0 (t, *J* = 21.2 Hz), 129.0, 127.66 (t, *J* = 6.2 Hz), 116.9 (t, *J* = 236.2 Hz), 73.7 ppm;
^{11}B NMR (160 MHz, CD_2Cl_2) δ = 32.4 ppm;
HRMS (ESI): *m*/*z* calculated for $C_{10}H_8BF_2O_2^-$, [M–H]$^-$ = 209.0591, found 209.0591.

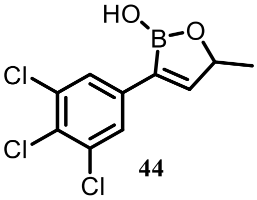

44

5-methyl-3-(3,4,5-trichlorophenyl)-1,2-oxaborol-2(5*H*)-ol
Prepared according to general procedure C: Yield 73%, colorless solid, mp: 154-155 ^{o}C;
^{1}H NMR (500 MHz, CD_2Cl_2) δ = 7.61 (s, 2H), 7.35 (s, 1H), 4.84 (q, *J* = 7.0 Hz, 1H), 1.28 (d, *J* = 7.0 Hz, 3H) ppm;
^{13}C NMR (126 MHz, CD_2Cl_2) δ = 155.6, 136.2, 134.0, 129.6, 127.1, 78.3, 20.3 ppm;
^{11}B NMR (160 MHz, CD_2Cl_2) δ = 32.7 ppm;
HRMS (ESI): *m*/*z* calculated for $C_{10}H_7BCl_3O_2^-$, [M–H]$^-$ = 274.9610, found 274.9611.

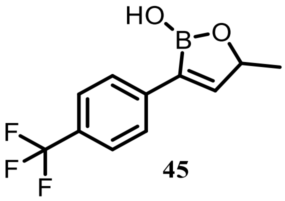

45

5-methyl-3-(4-(trifluoromethyl)phenyl)-1,2-oxaborol-2(5*H*)-ol
Prepared according to general procedure C: Yield 85%, colorless solid, mp: 167-168 ^{o}C;
^{1}H NMR (500 MHz, CD_3OD) δ = 7.70 – 7.66 (m, 2H), 7.49 – 7.43 (m, 3H), 4.80 (q, *J* = 7.0 Hz, 1H), 1.24 (d, *J* = 7.0 Hz, 3H) ppm;
^{13}C NMR (126 MHz, CD_3OD) δ = 154.9, 139.8, 128.6 (q, *J* = 31.2 Hz), 124.8 (q, *J* = 3.7 Hz), 124.4 (q, *J* = 268.7 Hz), 121.2, 78.2, 19.8 ppm;
^{11}B NMR (160 MHz, CD_3OD) δ = 32.3 ppm;
HRMS (ESI): *m*/*z* calculated for $C_{11}H_9BF_3O_2^-$, [M–H]$^-$ = 241.0653, found 241.0653.

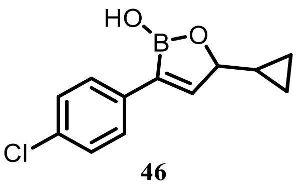

46

3-(4-chlorophenyl)-5-cyclopropyl-1,2-oxaborol-2(5*H*)-ol
Prepared according to general procedure C: Yield 52%, colorless solid, mp: 136-137 ºC;
^{1}H NMR (500 MHz, CD_2Cl_2) δ = 7.68 – 7.62 (m, 2H), 7.43 (s, 1H), 7.39 – 7.30 (m, 2H), 6.14 (s, 1H, -OH), 4.28 (d, *J* = 8.0 Hz, 1H), 0.99 – 0.92 (m, 1H), 0.68 – 0.59 (m, 2H), 0.54 – 0.49 (m, 1H), 0.47 – 0.42 (m, 1H) ppm;
^{13}C NMR (126 MHz, CD_2Cl_2) δ = 150.9, 134.4, 133.0, 128.5, 128.2, 85.7, 14.6, 2.7, 1.1 ppm;
^{11}B NMR (160 MHz, CD_2Cl_2) δ = 32.2 ppm;
HRMS (ESI): *m*/*z* calculated for $C_{12}H_{11}BClO_2^-$, $[M–H]^-$ = 233.0546, found 233.0547.

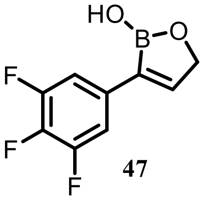

47

3-(3,4,5-trifluorophenyl)-1,2-oxaborol-2(5*H*)-ol
Prepared according to general procedure C: Yield 60%, colorless solid, mp: 61-62 ºC;
^{1}H NMR (500 MHz, CD_2Cl_2) δ = 7.37 (s, 1H), 7.28 – 7.18 (m, 2H), 4.59 (s, 2H) ppm;
^{13}C NMR (126 MHz, CD_3OD) δ = 151.0 (ddd, *J* = 245, 10, 5 Hz), 149.8, 138.5 (dt, *J* = 247, 15 Hz), 132.6 (q, *J* = 7.4 Hz), 110.49 (dd, *J* = 11.2, 5 Hz), 71.5 ppm;
^{11}B NMR (160 MHz, CD_2Cl_2) δ = 32.1 ppm;
HRMS (ESI): *m*/*z* calculated for $C_9H_5BF_3O_2^-$, $[M–H]^-$ = 213.0340, found 213.0340.

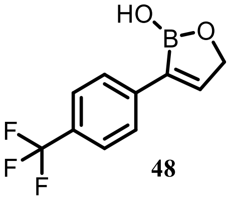

48

3-(4-(trifluoromethyl)phenyl)-1,2-oxaborol-2(5*H*)-ol
Prepared according to general procedure C: Yield 76%, colorless solid, mp: 151-152 ºC;
^{1}H NMR (500 MHz, CD_3OD) δ = 7.71 – 7.68 (d, *J* = 8.1 Hz, 2H), 7.54 (s, 1H), 7.49 – 7.45 (m, 2H), 4.58 (s, 2H) ppm;
^{13}C NMR (126 MHz, CD_3OD) δ = 150.1, 139.8, 128.58 (q, *J* = 31.2 Hz), 127.0, 124.7 (q, *J* = 3.7 Hz), 124.4 (q, *J* = 270 Hz), 71.6 ppm;
^{11}B NMR (160 MHz, CD_3OD) δ = 32.4 ppm;
HRMS (ESI): *m*/*z* calculated for $C_{10}H_7BF_3O_2^-$, $[M–H]^-$ = 227.0497, found 227.0497.

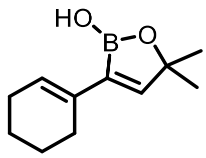

49

3-(cyclohex-1-en-1-yl)-5,5-dimethyl-1,2-oxaborol-2(5*H*)-ol

Prepared according to general procedure C: Yield 52%, colorless solid, mp: 104-106 ºC;

^{1}H NMR (500 MHz, $CDCl_3$) δ = 6.74 (s, 1H), 6.38 (s, 1H, -OH), 5.98 – 5.97 (m, 1H), 2.19 – 2.14 (m, 4H), 1.72 – 1.67 (m, 2H), 1.62 – 1.58 (m, 2H), 1.36 (s, 6H) ppm;

^{13}C NMR (126 MHz, $CDCl_3$) δ = 152.7, 133.4, 128.9, 83.4, 27.6, 26.1, 25.9, 22.7, 22.3 ppm;

^{11}B NMR (160 MHz, $CDCl_3$) δ = 33.0 ppm;

HRMS (ESI): *m*/*z* calculated for $C_{11}H_{17}BO_2$+H^+, $[M+H]^+$ = 193.1394, found 193.1394.

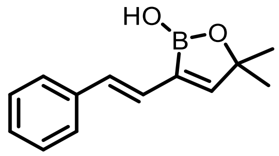

50

(*E*)-5,5-dimethyl-3-styryl-5λ-1,2-oxaborol-2(3*H*)-ol

Prepared according to general procedure D: Yield 70%, colorless solid, mp: 137-138 ºC;

^{1}H NMR (500 MHz, $CDCl_3$) δ = 7.52 – 7.44 (m, 2H), 7.37 – 7.31 (m, 2H), 7.27 – 7.22 (m, 1H), 7.15 (d, *J* = 15 Hz, 1H), 7.00 (s, 1H), 6.95 (d, *J* = 15 Hz, 1H), 6.05 (s, 1H, -OH), 1.45 (s, 6H) ppm;

^{13}C NMR (126 MHz, $CDCl_3$) δ = 159.0, 137.7, 133.4, 128.5, 127.4, 126.4, 125.0, 83.9, 27.4 ppm;

^{11}B NMR (160 MHz, $CDCl_3$) δ = 33.1 ppm;

HRMS (ESI): *m*/*z* calculated for $C_{13}H_{14}BO_2^-$, $[M–H]^-$ = 213.1092, found 213.1092.

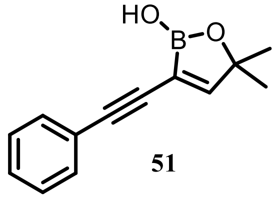

51

5,5-dimethyl-3-(phenylethynyl)-1,2-oxaborol-2(5*H*)-ol

Prepared according to general procedure C: Yield 82%, colorless solid, mp: 115-116 ºC;

^{1}H NMR (500 MHz, CD_2Cl_2) δ = 7.38 – 7.35 (m, 2H), 7.28 (s, 1H), 7.26 – 7.23 (m, 3H), 5.10 (s, 1H, -OH), 1.30 (s, 6H) ppm;

^{13}C NMR (126 MHz, CD_2Cl_2) δ = 167.6, 131.4, 128.3, 128.2, 123.3, 93.2, 84.8, 84.6, 26.6 ppm;

^{11}B NMR (160 MHz, CD_2Cl_2) δ = 31.2 ppm;

HRMS (ESI): *m*/*z* calculated for $C_{13}H_{12}BO_2^-$, $[M–H]^-$ = 211.0936, found 211.0936.

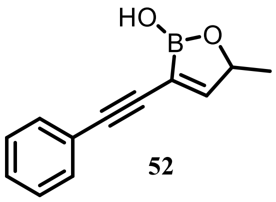

52

5-methyl-3-(phenylethynyl)-1,2-oxaborol-2(5*H*)-ol

Prepared according to general procedure C: Yield 79%, colorless solid, mp: 101-102 °C;
^{1}H NMR (500 MHz, $CDCl_3$) δ = 7.49 – 7.45 (m, 2H), 7.37 (s, 1H), 7.33 – 7.29 (m, 3H), 5.36 (s, 1H, -OH), 4.93 (q, *J* = 7.0 Hz, 1H), 1.36 (d, *J* = 7.0 Hz, 3H) ppm;
^{13}C NMR (126 MHz, $CDCl_3$) δ = 163.1, 131.6, 128.31, 128.29, 123.3, 94.0, 84.6, 78.9, 20.4 ppm;
^{11}B NMR (160 MHz, $CDCl_3$) δ = 31.7 ppm;
HRMS (ESI): *m*/*z* calculated for $C_{12}H_{10}BO_2^-$, $[M–H]^-$ = 197.0779, found 197.0779.

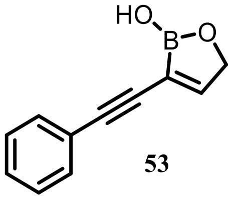

53

3-(phenylethynyl)-1,2-oxaborol-2(5*H*)-ol

Prepared according to general procedure C: Yield 68%, light yellow solid, mp: 91-93 °C;
^{1}H NMR (500 MHz, $CDCl_3$) δ = 7.48 – 7.45 (m, 3H), 7.33 – 7.30 (m, 3H), 4.81 (s, 1H, -OH), 4.69 (s, 2H) ppm;
^{13}C NMR (126 MHz, $CDCl_3$) δ = 158.2, 131.5, 128.33, 128.31, 123.2, 93.9, 84.6, 72.3 ppm;
^{11}B NMR (160 MHz, $CDCl_3$) δ = 32.1 ppm;
HRMS (ESI): *m*/*z* calculated for $C_{11}H_8BO_2^-$, $[M–H]^-$ = 183.0623 found 183.0623.

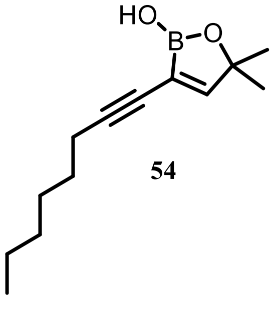

54

5,5-dimethyl-3-(oct-1-yn-1-yl)-1,2-oxaborol-2(5*H*)-ol

Prepared according to general procedure D: Yield 70%, light yellow oil;
^{1}H NMR (500 MHz, $CDCl_3$) δ = 7.15 (s, 1H), 5.32 (brs, 1H, -OH), 2.35 (t, *J* = 7.0 Hz, 2H), 1.57 – 1.53 (m, 2H), 1.42 – 1.37 (m,2H), 1.37 (s, 6H), 1.31 – 1.25 (m, 4H), 0.89 (t, *J* = 6.5 Hz, 3H) ppm;
^{13}C NMR (126 MHz, $CDCl_3$) δ = 165.8, 95.2, 84.6, 75.7, 31.3, 28.8, 28.6, 26.9, 22.5, 19.7, 14.0 ppm;
^{11}B NMR (160 MHz, $CDCl_3$) δ = 31.4 ppm;
HRMS (ESI): *m*/*z* calculated for $C_{13}H_{20}BO_2^-$, $[M–H]^-$ = 219.1562, found 219.1564.

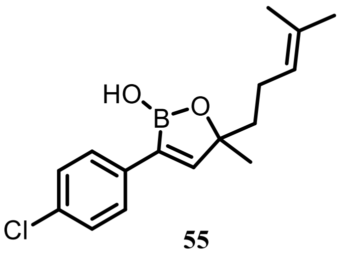

55

3-(4-chlorophenyl)-5-methyl-5-(4-methylpent-3-en-1-yl)-1,2-oxaborol-2(5*H*)-ol
Prepared according to general procedure C: Yield 85%, colorless solid, mp: 107-108 °C;
^{1}H NMR (500 MHz, CD_2Cl_2) δ = 7.53 – 7.48 (m, 2H), 7.25 – 7.21 (m, 2H), 7.21 (s, 1H), 5.88 (s, 1H, -OH), 4.99 (t, J = 7.5 Hz, 1H), 1.94 – 1.83 (m, 2H), 1.71 – 1.62 (m, 2H), 1.57 (s, 3H), 1.46 (s, 3H), 1.33 (s, 3H) ppm;
^{13}C NMR (126 MHz, CD_2Cl_2) δ = 156.1, 134.6, 132.8, 131.7, 128.5, 128.2, 123.9, 86.0, 39.7, 25.8, 25.3, 22.8, 17.3 ppm;
^{11}B NMR (160 MHz, CD_2Cl_2) δ = 33.3 ppm;
HRMS (ESI): *m*/*z* calculated for $C_{16}H_{19}BClO_2^-$, [M–H]$^-$ = 289.1172, found 289.1171.

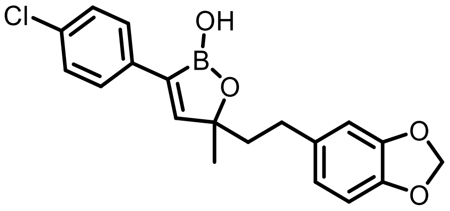

56

5-(2-(benzo[*d*][1,3]dioxol-5-yl)ethyl)-3-(4-chlorophenyl)-5-methyl-1,2-oxaborol-2(5*H*)-ol
Prepared according to general procedure C: Yield 83%, colorless solid, mp: 149-150 °C;
^{1}H NMR (500 MHz, CD_2Cl_2) δ = 7.50 (d, J = 10 Hz, 2H), 7.24 (d, J = 10 Hz, 2H), 7.20 (s, 1H), 6.61 (d, J = 10 Hz, 1H), 6.57 (s, 1H), 6.54 – 6.51 (m, 1H), 5.81 (s, 1H), 5.80 (s, 1H), 5.42 (s, 1H, OH), 2.48 – 2.37 (m, 2H), 1.95 – 1.85 (m, 2H), 1.36 (s, 3H) ppm;
^{13}C NMR (126 MHz, CD_2Cl_2) δ = 156.1, 147.6, 145.6, 135.9, 134.5, 132.9, 128.5, 128.2, 120.9, 108.7, 108.0, 100.9, 85.6, 41.7, 30.1, 25.8 ppm;
^{11}B NMR (160 MHz, CD_2Cl_2) δ = 33.5 ppm;
HRMS (ESI): *m*/*z* calculated for $C_{19}H_{17}BClO_4^-$, [M–H]$^-$ = 355.0914, found 355.0915.

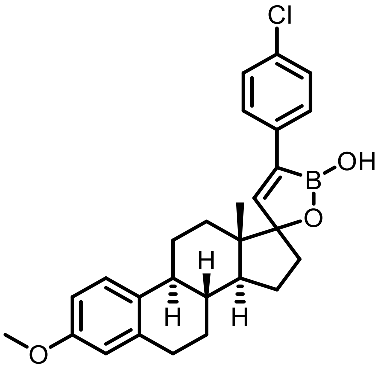

57

(8*R*,9*S*,13*S*,14*S*)-3'-(4-chlorophenyl)-3-methoxy-13-methyl-6,7,8,9,11,12,13,14,15,16-decahydro-2'*H*-spiro[cyclopenta[*a*]phenanthrene-17,5'-[1,2]oxaborol]-2'-ol
Prepared according to general procedure C: Yield 78%, colorless solid, mp: 198-200 °C;
^{1}H NMR (500 MHz, CD_2Cl_2) δ = 7.53 – 7.49 (m, 2H), 7.42 (s, 1H), 7.25 – 7.19 (m, 2H), 7.08 – 7.04 (m, 1H), 6.58 – 6.55 (m, 1H), 6.53 (s, 1H), 5.56 (s, 1H, -OH), 3.65 (s, 3H), 2.83 – 2.74 (m, 2H), 2.17 – 2.07 (m, 3H), 1.89 – 1.78 (m, 3H), 1.64 – 1.58 (m, 1H), 1.51 – 1.33 (m, 4H), 1.26 – 1.09 (m, 2H), 0.94 (s, 3H) ppm;
^{13}C NMR (126 MHz, CD_2Cl_2) δ = 157.8, 155.8, 138.3, 135.1, 133.0, 132.8, 128.8, 128.5, 126.5, 113.9, 111.6, 96.4, 55.4, 51.0, 47.3, 44.1, 39.6, 33.6, 33.3, 30.0, 27.8, 26.6, 23.8, 15.0 ppm;
^{11}B NMR (160 MHz, CD_2Cl_2) δ = 34.3 ppm;
HRMS (ESI): *m*/*z* calculated for $C_{27}H_{31}BClO_3^+$, $[M+H]^+$ = 449.2049, found 449.2049

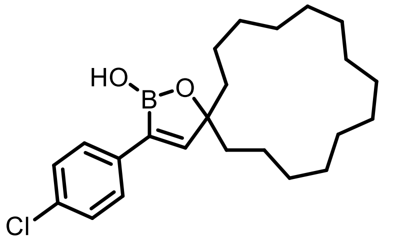

58

3-(4-chlorophenyl)-1-oxa-2-boraspiro[4.14]nonadec-3-en-2-ol
Prepared according to general procedure C: Yield 70%, colorless solid, mp: 132-133 °C;
^{1}H NMR (500 MHz, CD_2Cl_2) δ = 7.53 – 7.48 (m, 2H), 7.37 (s, 1H), 7.24 – 7.20 (m, 2H), 5.61 (s, 1H, -OH), 1.63 – 1.50 (m, 4H), 1.43 – 1.32 (m, 24H) ppm;
^{13}C NMR (126 MHz, CD_2Cl_2) δ = 158.4, 136.5, 134.8, 130.5, 130.1, 90.5, 55.6, 55.3, 55.1, 54.9, 54.7, 38.9, 33.5, 29.6, 28.9, 28.62, 28.61, 28.3, 24.6, 15.9 ppm;
^{11}B NMR (160 MHz, CD_2Cl_2) δ = 33.8 ppm;
HRMS (ESI): *m*/*z* calculated for $C_{23}H_{33}BO_2^-$, $[M–H]^-$ = 387.2268, found 387.2270.

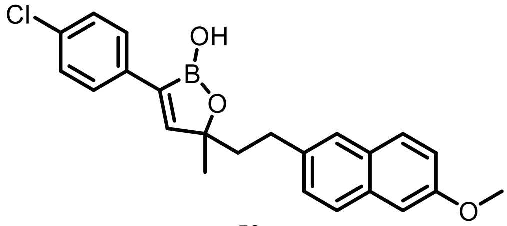

59

3-(4-chlorophenyl)-5-(2-(6-methoxynaphthalen-2-yl)ethyl)-5-methyl-1,2-oxaborol-2(5*H*)-ol
Prepared according to general procedure C: Yield 85%, colorless solid, mp: 179-181 °C;
^{1}H NMR (500 MHz, Acetone-d_6) δ = 7.76 – 7.69 (m, 4H), 7.59 (s, 1H), 7.55 (s, 1H, -OH), 7.39 – 7.35 (m, 2H), 7.32 (dd, *J* = 8.5, 1.5 Hz, 1H), 7.25 (d, *J* = 2.5 Hz, 1H), 7.11 (dd, *J* = 8.5, 2.5 Hz, 1H), 3.90 (s, 3H), 2.79 – 2.69 (m, 2H), 2.12 – 2.08 (m, 2H), 1.46 (s, 3H) ppm;
^{13}C NMR (126 MHz, Acetone-d_6) δ = 157.3, 156.6, 137.4, 135.3, 133.2, 132.3, 129.2, 128.8, 128.6, 128.3, 127.6, 126.8, 126.0, 118.5, 105.6, 84.6, 54.7, 41.7, 30.3, 25.6 ppm;
^{11}B NMR (160 MHz, Acetone-d_6) δ = 32.6 ppm;

HRMS (ESI): *m*/*z* calculated for $C_{23}H_{21}BClO_3^{-}$, $[M–H]^{-}$ = 391.1278, found 391.1279.

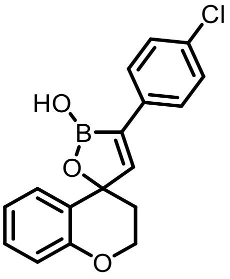

60

3'-(4-chlorophenyl)-2'*H*-spiro[chromane-4,5'-[1,2]oxaborol]-2'-ol

Prepared according to general procedure C: Yield 62%, colorless solid, mp: 178-179 ^{o}C;

^{1}H NMR (500 MHz, CD_2Cl_2) δ = 7.59 – 7.55 (m, 2H), 7.29 – 7.24 (m, 3H), 7.14 – 7.10 (m, 1H), 7.03 – 6.99 (m, 1H), 6.80 – 6.74 (m, 2H), 4.98 (s, 1H, -OH), 4.33 – 4.25 (m, 2H), 2.33 – 2.27 (m, 1H), 1.91 – 1.87 (m, 1H) ppm;

^{13}C NMR (126 MHz, Acetone-*d*$_6$) δ = 155.0, 154.6, 134.8, 132.7, 129.6, 128.8, 128.4, 127.7, 122.7, 120.3, 117.1, 79.7, 63.0, 34.6 ppm;

^{11}B NMR (160 MHz, Acetone-*d*$_6$) δ = 32.3 ppm;

HRMS (ESI): *m*/*z* calculated for $C_{17}H_{13}BClO_3^{-}$, $[M–H]^{-}$ = 311.0652, found 311.0652.

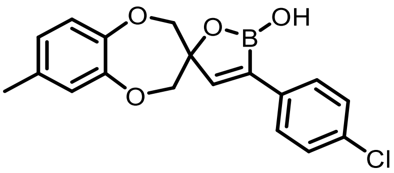

61

3'-(4-chlorophenyl)-7-methyl-2*H*,2'*H*,4*H*-spiro[benzo[*b*][1,4]dioxepine-3,5'-[1,2]oxaborol]-2'-ol

Prepared according to general procedure C: Yield 41%, colorless solid, mp: 150-151 ^{o}C;

^{1}H NMR (500 MHz, Acetone-*d*$_6$) δ = 7.82 (d, *J* = 5.0 Hz, 2H), 7.73 (s, 1H), 7.43 – 7.38 (m, 2H), 6.87 – 6.82 (m, 1H), 6.80 – 6.74 (m, 2H), 4.26 – 4.19 (m, 4H), 2.97 (s, 1H, -OH), 2.24 (s, 3H) ppm;

^{13}C NMR (126 MHz, Acetone-*d*$_6$) δ = 149.7, 149.6, 147.9, 134.5, 133.0, 132.8, 128.9, 128.5, 123.7, 121.0, 120.4, 86.3, 76.1, 76.0, 19.6 ppm;

^{11}B NMR (160 MHz, Acetone-*d*$_6$) δ = 34.2 ppm;

HRMS (ESI): *m*/*z* calculated for $C_{18}H_{15}BClO_4^{-}$, $[M–H]^{-}$ = 341.0757, found 341.0757.

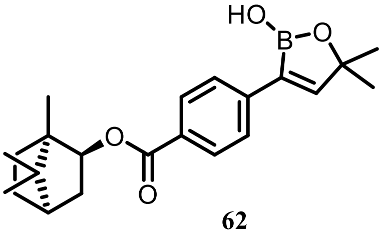

62

(1*R*,4*R*)-1,7,7-trimethylbicyclo[2.2.1]heptan-2-yl 4-(2-hydroxy-5,5-dimethyl-2,5-dihydro-1,2-oxaborol-3-yl)benzoate

Prepared according to general procedure C: Yield 71%, colorless solid, mp: 172-173 °C;
^{1}H NMR (500 MHz, CD_3OD) δ = 7.86 – 7.81 (m, 2H), 7.65 – 7.61 (m, 2H), 7.45 (s, 1H), 5.05 – 4.95 (m, 1H), 2.40 – 2.32 (m, 1H), 2.10 – 2.03 (m, 1H), 1.78 – 1.71 (m, 1H), 1.63 – 1.61 (m, 1H), 1.39 – 1.34 (m, 1H), 1.31 (s, 6H), 1.24 – 1.20 (m, 1H), 1.03-0.98 (m, 1H), 0.88 (s, 3H), 0.84 (s, 3H), 0.81 (s, 3H) ppm;
^{13}C NMR (126 MHz, CD_3OD) δ = 166.9, 159.0, 140.9, 129.10, 128.9, 126.8, 83.6, 80.4, 48.7, 47.6, 45.0, 36.5, 27.6, 27.0, 26.3, 18.7, 17.9, 12.6 ppm;
^{11}B NMR (160 MHz, CD_3OD) δ = 32.6 ppm;
HRMS (ESI): *m/z* calculated for $C_{22}H_{28}BO_4^-$, $[M–H]^-$ = 367.2086, found 367.2087.
3-(2,4-dichlorophenyl)-5-methyl-1,2-oxaborol-2(5*H*)-ol

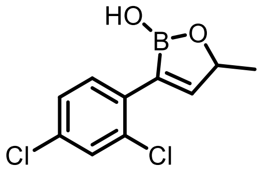

63

Prepared according to general procedure C: Yield 69%, colorless solid, mp: 96-98 °C;
^{1}H NMR (500 MHz, CD_3OD) δ = 7.47 – 7.40 (m, 2H), 7.38 – 7.33 (m, 1H), 7.28 – 7.22 (m, 1H), 4.91 (q, *J* = 6.5 Hz, 1H), 1.36 (d, *J* = 6.5 Hz, 3H) ppm;
^{13}C NMR (126 MHz, CD_3OD) δ = 158.3, 134.4, 132.9, 132.5, 131.2, 128.9, 126.6, 78.4, 19.8 ppm;
^{11}B NMR (160 MHz, CD_3OD) δ = 31.6 ppm;
HRMS (ESI): *m/z* calculated for $C_{10}H_8BCl_2O_2^-$, $[M–H]^-$ = 241.0000, found 241.0000.

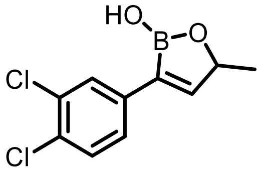

64

3-(3,4-dichlorophenyl)-5-methyl-1,2-oxaborol-2(5H)-ol
Prepared according to general procedure C: Yield 92%, colorless soild, mp: 100-101°C;
^{1}H NMR (500 MHz, CD_3OD) δ = 7.74 (s, 1H), 7.52 – 7.48 (m, 1H), 7.47 (s, 1H), 7.41 – 7.35 (m, 1H), 4.89 (d, *J* = 7.5 Hz, 1H), 1.33 (d, *J* = 7.5 Hz, 3H) ppm;
^{13}C NMR (126 MHz, CD_3OD) δ = 154.2, 136.3, 131.8, 130.4, 130.0, 128.6, 126.2, 78.2, 19.8 ppm;
^{11}B NMR (160 MHz, CD_3OD) δ = 31.7 ppm;
HRMS (ESI): *m/z* calculated for $C_{10}H_8BCl_2O_2^-$, $[M–H]^-$ = 241.0000, found 241.0000.

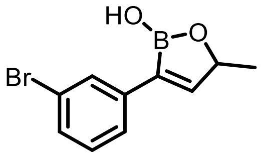

65

3-(3-bromophenyl)-5-methyl-1,2-oxaborol-2(5H)-ol

Prepared according to general procedure C: Yield 80%, colorless soild, mp: 77-78 °C;

^{1}H NMR (500 MHz, CD_3OD) δ = 7.80 (s, 1H), 7.62 – 7.57 (m, 1H), 7.46 (s, 1H), 7.39 – 7.33 (m, 1H), 7.24 – 7.18 (m, 1H), 4.90 (d, *J* = 7.0 Hz, 1H), 1.35 (d, *J* = 7.0 Hz, 3H) ppm;

^{13}C NMR (126 MHz, CD_3OD) δ = 153.7, 138.2, 129.7, 129.7, 129.6, 125.2, 122.0, 78.1, 19.9 ppm;

^{11}B NMR (160 MHz, CD_3OD) δ = 31.4 ppm;

HRMS (ESI): *m/z* calculated for $C_{10}H_9BBrO_2^-$, $[M–H]^-$ = 250.9884, found 250.9885.

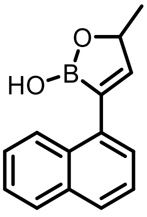

66

5-methyl-3-(naphthalen-1-yl)-1,2-oxaborol-2(5H)-ol

Prepared according to general procedure C: Yield 78%, colorless soild, mp: 126-127 °C;

^{1}H NMR (500 MHz, CD_3OD) δ = 8.04 – 7.98 (m, 1H), 7.87 – 7.81 (m, 1H), 7.76 – 7.72 (m, 1H), 7.47 – 7.39 (m, 3H), 7.35 – 7.29 (m, 1H), 7.25 (s, 1H), 4.99 (q, *J* = 7.0 Hz, 1H), 1.43 (d, *J* = 7.0 Hz, 3H) ppm;

^{13}C NMR (126 MHz, CD_3OD) δ = 157.5, 135.3, 133.8, 131.2, 127.9, 126.8, 125.4, 125.2, 125.1, 125.04, 125.01, 78.6, 20.1 ppm;

^{11}B NMR (160 MHz, CD_3OD) δ = 31.8 ppm;

HRMS (ESI): *m/z* calculated for $C_{14}H_{12}BO_2^-$, $[M-H]^-$ = 223.0936, found 223.0935.

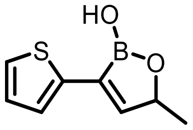

67

5-methyl-3-(thiophen-2-yl)-1,2-oxaborol-2(5H)-ol

Prepared according to general procedure C: Yield 91%, light yellow soild, mp: 102-103 °C;

^{1}H NMR (500 MHz, CD_3OD) δ = 7.34 – 7.22 (m, 2H), 7.18 (s, 1H), 7.01 – 6.93 (m, 1H), 4.89 (q, *J* = 6.5 Hz, 1H), 1.33 (d, *J* = 6.5 Hz, 3H) ppm;

^{13}C NMR (126 MHz, CD_3OD) δ = 149.8, 139.4, 126.9, 126.0, 124.2, 78.3, 20.0 ppm;

^{11}B NMR (160 MHz, CD_3OD) δ = 31.1 ppm;

HRMS (ESI): *m/z* calculated for $C_8H_{10}BO_2S^+$ $[M+H]^+$ = 181.0489, found 181.0433.

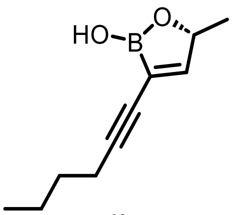

69

(*R*)-3-(hex-1-yn-1-yl)-5-methyl-1,2-oxaborol-2(5*H*)-ol

Prepared according to general procedure D: Yield 52%, light yellow oil;

^{1}H NMR (500 MHz, CD_3OD) δ = 7.17 (s, 1H), 4.79 (q, *J* = 7.0 Hz, 1H), 2.33 (t, *J* = 7.0 Hz, 2H), 1.51 – 1.42 (m, 4H), 1.24 (d, *J* = 7.0 Hz, 3H), 0.93 (t, *J* = 7.0 Hz, 3H) ppm;

^{13}C NMR (126 MHz, CD_3OD) δ = 161.0, 93.2, 77.8, 75.3, 30.1, 21.0, 19.1, 18.0, 12.0 ppm;

^{11}B NMR (160 MHz, CD_3OD) δ = 31.3 ppm;

HRMS (ESI): *m*/*z* calculated for $C_{10}H_{14}BO_2^-$, $[M–H]^-$ = 177.1092, found 177.1092.

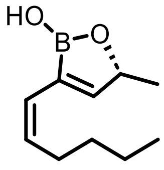

70

Methyl hydrogen ((3*E*,5*Z*)-deca-3,5-dien-4-yl)boronate

Yield 55%, light yellow oil;

^{1}H NMR (500 MHz, CD_3OD) δ = 6.83 (s, 1H), 5.87 (d, *J* = 11.5 Hz, 1H), 5.37 (dt, *J* = 11.5, 7.5 Hz, 1H), 4.67 (q, *J* = 7.0 Hz, 1H), 2.22 – 2.16 (m, 2H), 1.27 – 1.23 (m, 4H), 1.16 (d, *J* = 7.0 Hz, 3H), 0.81 (t, *J* = 7.0 Hz, 3H) ppm;

^{13}C NMR (126 MHz, CD_3OD) δ = 156.4, 133.4, 123.7, 77.9, 31.8, 28.1, 22.0, 20.1, 12.9 ppm;

^{11}B NMR (160 MHz, CD_3OD) δ = 31.3 ppm;

HRMS (ESI): *m*/*z* calculated for $C_{10}H_{16}BO_2^-$, $[M–H]^-$ = 179.1249, found 179.1248.

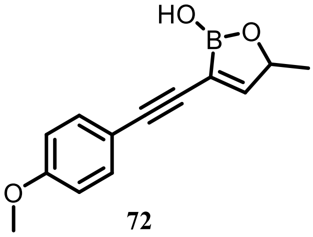

72

3-((4-methoxyphenyl)ethynyl)-5-methyl-1,2-oxaborol-2(5H)-ol

Prepared according to general procedure C: Yield 59%, light yellow soild, mp: 85-87 ^{o}C;

^{1}H NMR (500 MHz, CD_3OD) δ = 7.31 – 7.20 (m, 2H), 7.21 (s, 1H), 6.80 – 6.73 (m, 2H), 4.75 (q, *J* = 6.5 Hz, 1H), 3.68 (s, 3H), 1.18 (d, *J* = 6.5 Hz, 3H) ppm;

^{13}C NMR (126 MHz, CD_3OD) δ = 162.1, 159.8, 132.5, 115.7, 113.7, 93.0, 83.4, 78.7, 54.4, 19.6 ppm;

^{11}B NMR (160 MHz, CD_3OD) δ = 32.3 ppm;

HRMS (ESI): *m*/*z* calculated for $C_{13}H_{12}BO_3^-$, $[M-H]^-$ = 227.0885, found 227.0886.

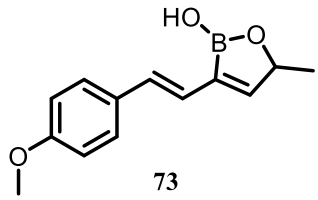

73

(*E*)-3-(4-methoxystyryl)-5-methyl-1,2-oxaborol-2(5H)-ol

Yield 65%, light yellow soild, mp: 78-80 ^oC;

^{1}H NMR (500 MHz, CD_3OD) δ = 7.37 – 7.30 (m, 2H), 6.99 (d, *J* = 16.0 Hz, 1H), 6.96 (s, 1H), 6.88 – 6.83 (m, 2H), 6.81 (d, *J* = 16.0 Hz, 1H), 4.80 (d, *J* = 6.5 Hz, 1H), 3.78 (s, 3H), 1.28 (d, *J* = 6.5 Hz, 3H) ppm;

^{13}C NMR (126 MHz, CD_3OD) δ = 159.4, 153.4, 132.5, 130.5, 127.1, 122.8, 113.6, 78.0, 54.3, 20.2 ppm;

^{11}B NMR (160 MHz, CD_3OD) δ = 32.7 ppm;

HRMS (ESI): *m/z* calculated for $C_{13}H_{15}BO_3Na^+$, $[M+Na]^+$ = 253.1006, found 253.1039.

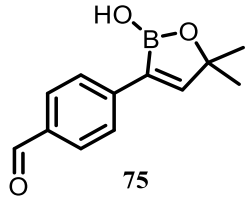

75

4-(2-hydroxy-5,5-dimethyl-2,5-dihydro-1,2-oxaborol-3-yl)benzaldehyde

Prepared according to general procedure C: Yield 60 %, colorless solid, mp: 133-134 ^oC;

^{1}H NMR (500 MHz, $CDCl_3$) δ =10.02 (s, 1H), 7.88 (d, *J* = 7.5 Hz, 2H), 7.83 (d, *J* = 7.5 Hz, 2H), 7.48 (s, 1H), 6.57 (s, 1H), 1.51 (s, 6H) ppm;

^{13}C NMR (126 MHz, $CDCl_3$) δ = 192.0, 159.5, 142.1, 135.3, 130.1, 127.6, 84.3, 27.2 ppm;

^{11}B MR (160 MHz, $CDCl_3$) δ = 31.7 ppm;

HRMS (ESI): *m/z* calculated for $C_{12}H_{12}BO_3^-$, $[M–H]^-$ = 215.0885, found 215.0885.

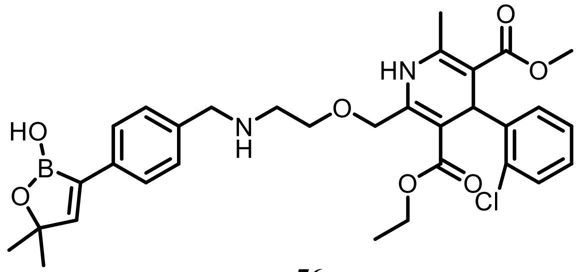

76

3-ethyl 5-methyl 2-((2-((4-(2-hydroxy-5,5-dimethyl-2,5-dihydro-1,2-oxaborol-3-yl)benzyl)amino)ethoxy)methyl)-6-methyl-4-phenyl-1,4-dihydropyridine-3,5-dicarboxylate

Yield 81 %, colorless solid, mp: 82-83 ^oC;

^{1}H NMR (500 MHz, $CDCl_3$) δ = 7.69 – 7.62 (m, 3H), 7.40 – 7.37 (m, 1H), 7.33 – 7.28 (m, 3H), 7.25 – 7.22 (m, 1H), 7.15 – 7.11 (m, 1H), 7.06 – 7.02 (m, 1H), 5.42 (s, 1H), 4.81 – 4.68 (m, 2H), 4.09 – 4.02 (m, 2H), 3.87 (s, 2H), 3.68 (q, *J* = 7.5 Hz, 2H), 3.62 (s, 3H), 2.92 – 2.86 (m, 2H), 2.31 (s, 3H), 1.44 (s, 6H), 1.19 (t, *J* = 7.5 Hz, 3H) ppm;

^{13}**C NMR** (126 MHz, $CDCl_3$) δ = 168.1, 167.2, 156.6, 145.9, 145.8, 144.5, 138.3, 135.2, 132.3, 131.5, 129.2, 128.4, 127.3, 127.2, 126.9, 103.8, 101.4, 83.5, 70.8, 68.0, 59.8, 53.3, 50.8, 47.8, 37.2, 27.5, 19.3, 14.3 ppm;
^{11}**B NMR** (160 MHz, $CDCl_3$) δ = 34.7 ppm;
HRMS (ESI): *m*/*z* calculated for $C_{32}H_{37}BClN_2O_7^-$, $[M–H]^-$ = 607.2388, found 607.2387.

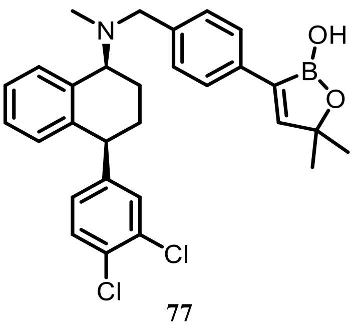

77

3-(4-((((1S)-4-(3,4-dichlorophenyl)-1,2,3,4-tetrahydronaphthalen-1-yl)(methyl)amino)methyl)phenyl)-5,5-dimethyl-1,2-oxaborol-2(5H)-ol
Yield 71 %, colorless solid, mp: 79-80 ^{o}C;
^{1}**H NMR** (500 MHz, $CDCl_3$) δ = 7.99 –7.97 (m, 1H), 7.64 (d, *J* = 8.5 Hz, 2H), 7.44 (d, *J* = 8.5 Hz, 2H), 7.35–7.29 (m, 3H), 7.18 – 7.15 (m, 2H), 6.93 – 6.89 (m, 1H), 6.87 – 6.83 (m, 1H), 5.80 (s, 1H), 4.15 (t, *J* = 4.5 Hz, 1H), 3.97 (t, *J* = 7.5 Hz, 1H), 3.77 – 3.72 (m, 1H), 3.62 – 3.56 (m, 1H), 2.23 (s, 3H), 2.20 – 2.10 (m, 1H), 2.10 – 2.01 (m, 1H), 1.80 – 1.74 (m, 2H), 1.48 (s, 6H) ppm;
^{13}**C NMR** (126 MHz, $CDCl_3$) δ = 156.4, 147.6, 139.6, 139.5, 138.2, 134.5, 132.2, 130.8, 130.2, 130.0, 128.8, 128.6, 128.2, 127.0, 126.9, 126.8, 83.8, 61.2, 57.6, 43.7, 37.1, 30.1, 27.4, 15.5 ppm;
^{11}**B NMR** (160 MHz, $CDCl_3$) δ = 34.7.ppm;
HRMS (ESI): *m*/*z* calculated for $C_{29}H_{31}BCl_2NO_2^+$, $[M+H]^+$ = 506.1819, found 506.1819.

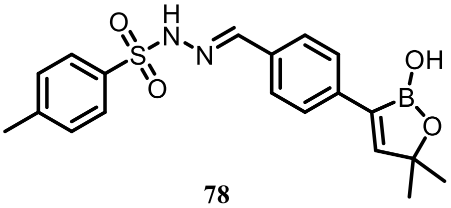

78

(*E*)-N'-(4-(2-hydroxy-5,5-dimethyl-2,5-dihydro-1,2-oxaborol-3-yl)benzylidene)-4-methylbenzenesulfonohydrazide
Yield 75%, light yellow soild, mp: 127-129°C;
^{1}**H NMR** (500 MHz, DMSO-*d*$_6$) δ = 11.40 (s, 1H), 8.87 (s, 1H), 7.88 (s, 1H), 7.77 (d, *J* = 8.5 Hz, 2H), 7.66 (d, *J* = 8.0 Hz, 2H), 7.56 (s, 1H), 7.51 (d, *J* = 8.0 Hz, 2H), 7.41 (d, *J* = 8.5 Hz, 2H), 2.36 (s, 3H), 1.34 (s, 6H) ppm;
^{13}**C NMR** (126 MHz, DMSO-*d*$_6$) δ = 158.9, 147.3, 143.9, 138.3, 136.6, 132.8, 130.1, 127.7, 127.6, 127.3, 82.8, 27.9, 21.5 ppm;
^{11}**B NMR** (160 MHz, DMSO-*d*$_6$) δ = 34.6 ppm;
HRMS (ESI): *m*/*z* calculated for $C_{19}H_{22}BN_2O_4S^+$ $[M+H]^+$ = 385.1388, found 385.1383.

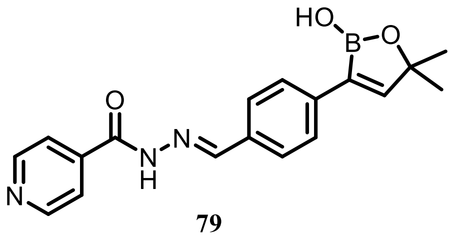

79

(*E*)-N'-(4-(2-hydroxy-5,5-dimethyl-2,5-dihydro-1,2-oxaborol-3-yl)benzylidene)isonicotinohydrazide
Yield 80%, light yellow soild, mp: 115-116 °C;
^{1}H NMR (500 MHz, DMSO-*d*$_6$) δ = 12.05 (s, 1H), 8.91 (s, 1H), 8.80 (d, *J* = 6.0 Hz, 2H), 8.45 (s, 1H), 7.83 (d, *J* = 6.0 Hz, 2H), 7.74 (q, *J* = 8.0 Hz, 4H), 7.62 (s, 1H), 1.36 (s, 6H) ppm;
^{13}C NMR (126 MHz, DMSO-*d*$_6$) δ = 162.0, 159.1, 150.8, 149.3, 141.0, 138.7, 133.2, 127.9, 127.7, 122.0, 82.8, 27.9 ppm;
^{11}B NMR (160 MHz, DMSO-*d*$_6$) δ = 34.8 ppm;
HRMS (ESI): *m*/*z* calculated for $C_{18}H_{19}BN_3O_3^+$ $[M+H]^+$ = 336.1514, found 336.1508.

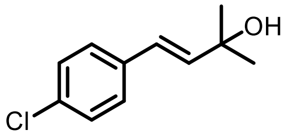

80

(*E*)-4-(4-chlorophenyl)-2-methylbut-3-en-2-ol
Yield 80%, colorless solid, mp: 65-67 °C;
^{1}H NMR (500 MHz, $CDCl_3$) δ = 7.35 – 7.27 (m, 4H), 6.57 (d, *J* = 16 Hz, 1H), 6.35 (d, *J* = 16 Hz, 1H), 1.55 (s, 1H, OH), 1.45 (s, 6H) ppm;
^{13}C NMR (126 MHz, $CDCl_3$) δ = 138.1, 135.4, 133.0, 128.7, 127.6, 125.2, 71.0, 29.9 ppm;
The NMR data is in agreement with previous literature[13].

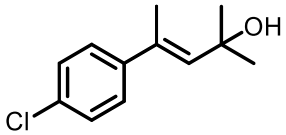

81

(*E*)-4-(4-chlorophenyl)-2-methylpent-3-en-2-ol
Yield 64%, colorless solid, mp: 91-92 °C;
^{1}H NMR (500 MHz, $CDCl_3$) δ = 7.30 – 7.24 (m, 4H), 5.83 (s, 1H), 2.25 (s, 3H), 1.46 (s, 6H) ppm;
^{13}C NMR (126 MHz, $CDCl_3$) δ = 143.0, 136.1, 135.7, 132.6, 128.2, 127.2, 71.2, 31.1, 16.7 ppm;
HRMS (ESI): *m*/*z* calculated for $C_{12}H_{14}ClO^-$, $[M-H]^-$ = 209.0739, found 209.0740.

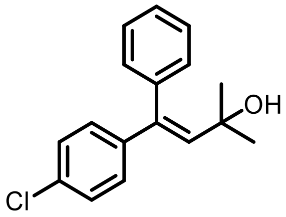

82

(*E*)-4-(4-chlorophenyl)-2-methyl-4-phenylbut-3-en-2-ol
Yield 65%, light yellow solid, mp: 101-102 ^oC;
^{1}H NMR (500 MHz, CD_2Cl_2) δ = 7.46 – 7.37 (m, 3H), 7.30 – 7.22 (m, 4H), 7.20 –7.12 (m, 2H), 6.26 (s, 1H), 1.48 (s, 1H), 1.33 (s, 6H) ppm;
^{13}C NMR (126 MHz, CD_2Cl_2) δ = 141.6, 139.4, 138.3, 137.4, 132.8, 129.8, 128.4, 128.3, 128.1, 127.5, 71.6, 31.1 ppm;
HRMS (ESI): *m*/*z* calculated for $C_{17}H_{16}ClO^-$, $[M–H]^-$ = 271.0895, found 271.0895.

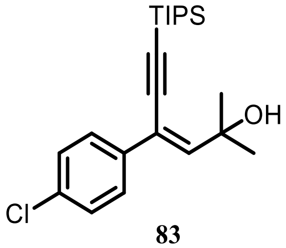

83

(*Z*)-2-methyl-4-phenyl-6-(triisopropylsilyl)hex-3-en-5-yn-2-ol
Yield 75%, light yellow oil;
^{1}H NMR (500 MHz, CD_3OD) δ = 7.61 – 7.56 (m, 2H), 7.36 – 7.31 (m, 2H), 6.65 (s, 1H), 1.64 (s, 6H), 1.16 (s, 21H) ppm;
^{13}C NMR (126 MHz, CD_3OD) δ = 148.1, 137.5, 133.3, 128.1, 127.2, 120.0, 103.3, 100.6, 71.1, 27.6, 17.7, 11.2 ppm;
HRMS (ESI): *m*/*z* calculated for $C_{22}H_{32}ClOSi^-$, $[M–H]^-$ = 375.1916, found 375.1915.

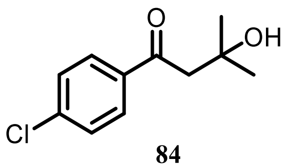

84

1-(4-chlorophenyl)-3-hydroxy-3-methylbutan-1-one
Yield 85%, light yellow oil;
^{1}H NMR (500 MHz, $CDCl_3$) δ = 7.92 – 7.86(m, 2H), 7.49 – 7.41 (m, 2H), 3.12 (s, 2H), 1.35 (s, 6H) ppm;
^{13}C NMR (126 MHz, $CDCl_3$) δ = 200.3, 140.1, 135.5, 129.5, 129.0, 69.8, 48.6, 29.5 ppm;
The NMR data is in agreement with previous literature[14].

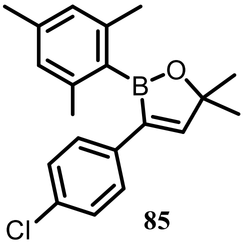

85

3-(4-chlorophenyl)-2-mesityl-5,5-dimethyl-2,5-dihydro-1,2-oxaborole
Yield 82%, light yellow oil;
^{1}H NMR (500 MHz, $CDCl_3$) δ = 7.51 (s, 1H), 7.16 – 7,12 (m, 2H), 7.11 – 7.07 (m, 2H), 6.73 (s, 2H), 2.22 (s, 3H), 2.09 (s, 6H), 1.48 (s, 6H) ppm;
^{13}C NMR (126 MHz, $CDCl_3$) δ = 159.7, 139.4, 138.2, 135.1, 133.0, 128.7, 127.9, 127.4, 90.0, 27.0, 21.7, 21.3 ppm;
^{11}B NMR (160 MHz, $CDCl_3$) δ = 34.3 ppm;
HRMS (ESI): *m/z* calculated for $C_{20}H_{23}BClO^+$, $[M+H]^+$ = 325.1525, found 325.1525.

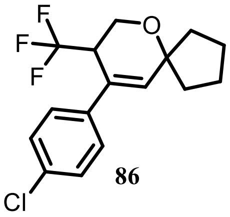

86

9-(4-chlorophenyl)-8-(trifluoromethyl)-6-oxaspiro[4.5]dec-9-ene
Yield 47%, colorless oil;
^{1}H NMR (500 MHz, $CDCl_3$) δ = 7.29 – 7.22 (m, 2H), 7.20 – 7.17 (m, 2H), 6.00 (s, 1H), 4.17 (d, *J* = 12.0 Hz, 1H), 3.75 (d, *J* = 12.0 Hz, 1H), 3.15 – 3.08 (m, 1H), 1.94 – 1.50 (m, 8H) ppm;
^{13}C NMR (126 MHz, $CDCl_3$) δ = 138.0, 137.3, 133.6, 128.7, 128.2 (d, *J* = 1.2 Hz), 127.3, 125.8 (q, *J* = 280 Hz), 84.0, 59.9, 59.8, 40.1 (q, *J* = 26.2 Hz), 39.5, 36.6, 24.2 (d, *J* = 4 Hz) ppm;
HRMS (ESI): *m/z* calculated for $C_{16}H_{17}ClF_3O^+$, $[M+H]^+$ = 317.0915, found 317.0914.

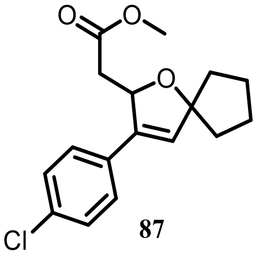

87

methyl 2-(3-(4-chlorophenyl)-1-oxaspiro[4.4]non-3-en-2-yl)acetate
Yield 64%, colorless oil;
^{1}H NMR (500 MHz, $CDCl_3$) δ = 7.35 – 7.30 (m, 2H), 7.29 – 7.26 (m, 2H), 6.05 (s, 1H), 5.63 (d, *J* = 8.5 Hz, 1H), 3.68 (s, 3H), 2.70 (dd, *J* = 15, 3.0 Hz, 1H), 2.42 (q, *J* = 9.0 Hz, 1H), 1.88 – 1.73 (m, 6H), 1.68 – 1.60 (m, 2H) ppm;
^{13}C NMR (126 MHz, $CDCl_3$) δ = 171.5, 138.7, 133.6, 131.3, 130.4, 128.9, 127.7, 98.0, 81.0, 51.7, 41.4, 40.2, 39.1, 24.7, 24.5 ppm;

HRMS (ESI): *m/z* calculated for $C_{17}H_{19}ClNaO_3^+$, $[M+Na]^+$ = 329.0915, found 329.0915.

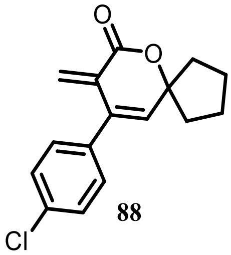

88

9-(4-chlorophenyl)-8-methylene-6-oxaspiro[4.5]dec-9-en-7-one
Yield 53%, light yellow oil;
^{1}H NMR (500 MHz, $CDCl_3$) δ = 7.41 – 7.37 (m, 2H), 7.28 – 7.24 (m, 2H), 6.53 (s, 1H), 5.91 (s, 1H), 5.56 (s, 1H), 2.13 – 2.04 (m, 4H), 1.91 – 1.80 (m, 4H) ppm;
^{13}C NMR (126 MHz, $CDCl_3$) δ = 164.3, 135.7, 135.0, 134.2, 130.8, 130.7, 129.7, 128.8, 125.4, 91.5, 58.4, 40.8, 23.8, 18.4 ppm;
HRMS (ESI): *m/z* calculated for $C_{16}H_{16}ClO_2^+$, $[M+H]^+$ = 275.0833, found 275.0833.

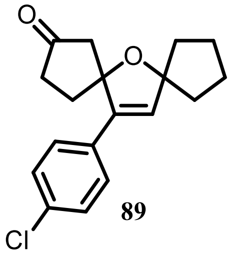

89

13-(4-chlorophenyl)-6-oxadispiro[4.1.4^{7}.2^{5}]tridec-12-en-2-one
yield 61%, colorless solid ,mp: 67-69 ^{o}C;
^{1}H NMR (500 MHz, $CDCl_3$) δ = 7.36 – 7.31 (m, 2H), 7.27 – 7.22 (m, 2H), 6.00 (s, 1H), 2.65 – 2.57 (m, 2H), 2.43 – 2.38 (m, 1H), 2.36 – 2.25 (m, 2H), 2.19 – 2.13 (m, 1H), 1.85 – 1.76 (m, 6H), 1.68 – 1.63 (m, 2H) ppm;
^{13}C NMR (126 MHz, $CDCl_3$) δ = 211.9, 134.6, 128.6, 128.0, 127.0, 123.6, 123.5, 90.4, 88.2, 45.3, 35.0, 34.7, 31.9, 30.7, 19.4, 19.3 ppm;
HRMS (ESI): *m/z* calculated for $C_{18}H_{19}ClNaO_2^+$, $[M+Na]^+$ = 325.0966, found 325.0966.

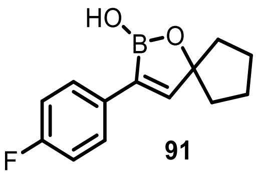

91

3-(4-fluorophenyl)-1-oxa-2-boraspiro[4.4]non-3-en-2-ol
Prepared according to general procedure C: Yield 82%, light yellow solid, mp: 134-135 ^{o}C;
^{1}H NMR (500 MHz, CD_3OD) δ = 7.57 – 7.49 (m, 2H), 7.19 (s, 1H), 6.92 – 6.84 (m, 2H), 1.89 – 1.79 (m, 4H), 1.74 – 1.67 (m, 2H), 1.55 – 1.48 (m, 2H) ppm;
^{13}C NMR (126 MHz, CD_3OD) δ = 162.1 (d, *J* = 243.7 Hz), 153.0 (d, *J* = 2.5 Hz), 132.2 (d, *J* = 2.5 Hz), 128.4 (d, *J* = 7.5 Hz), 114.5 (d, *J* = 22.5 Hz), 94.0, 37.8, 24.1 ppm;
^{11}B NMR (160 MHz, CD_3OD) δ = 31.6 ppm;
HRMS (ESI): *m/z* calculated for $C_{13}H_{13}BFO_2^-$, $[M–H]^-$ = 231.0998, found 231.0981.

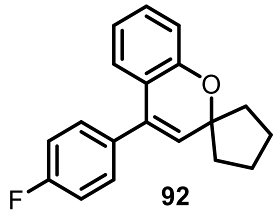

92

4-(4-fluorophenyl)spiro[chromene-2,1'-cyclopentane][30]

Prepared according to the previous literature[30]: Yield 52%, light yellow oil;

^{1}H NMR (500 MHz, Chloroform-*d*) δ = 7.35 – 7.28 (m, 2H), 7.16 – 7.11 (m, 1H), 7.10 – 7.03 (m, 2H), 6.96 – 6.91 (m, 1H), 6.88 – 6.77 (m, 2H), 5.64 (s, 1H), 2.25 – 2.16 (m, 2H), 1.98 – 1.90 (m, 2H), 1.75 – 1.61 (m, 4H) ppm.

^{13}C NMR (126 MHz, Chloroform-*d*) δ = 162.4 (d, *J* = 245.0 Hz), 153.4, 134.7, 134.5 (d, *J* = 3.7 Hz), 130.3 (d, *J* = 8.7 Hz), 129.1, 128.3, 125.3, 123.0, 120.6, 117.0, 115.2 (d, *J* = 21.2 Hz), 86.7, 38.9, 23.6 ppm.

The NMR spectra is identical to the prior report[30].

6. NMR Spectra

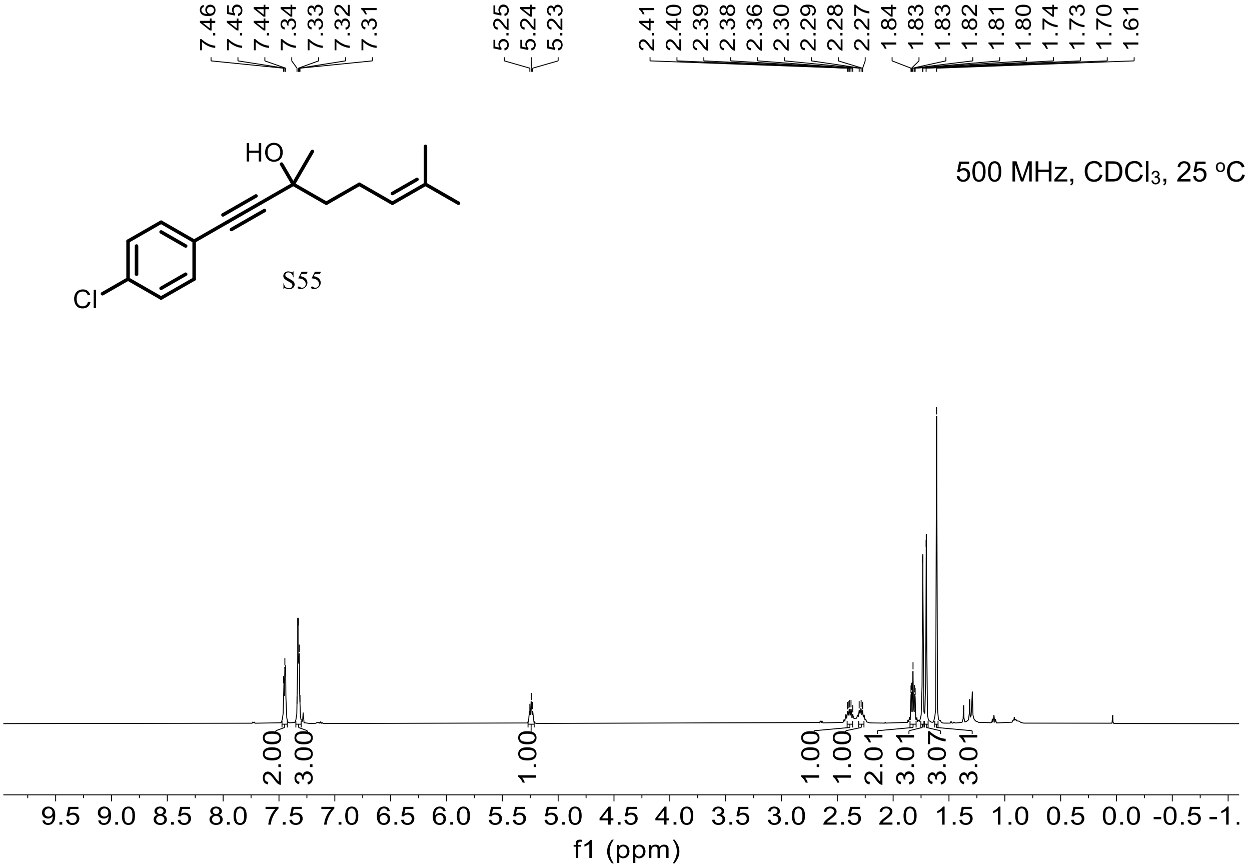

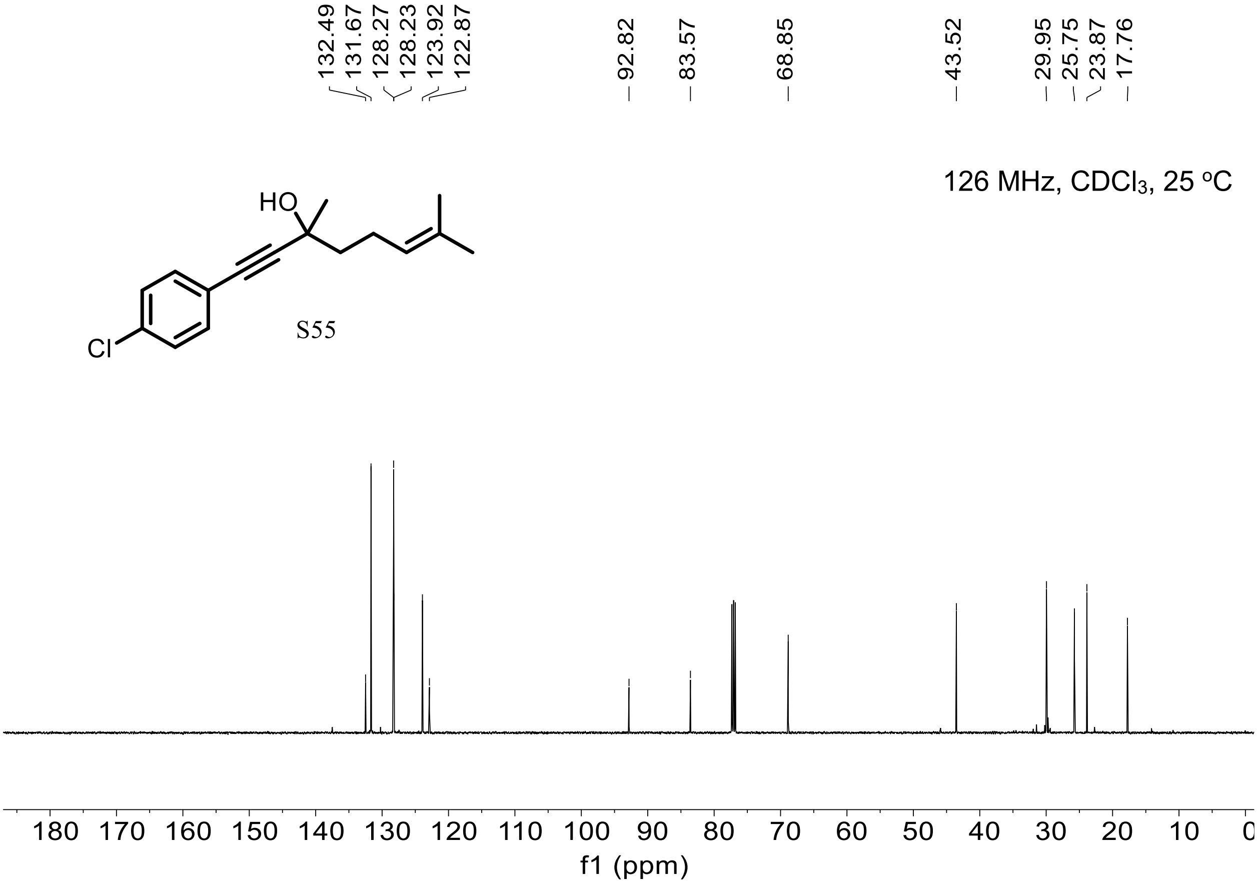

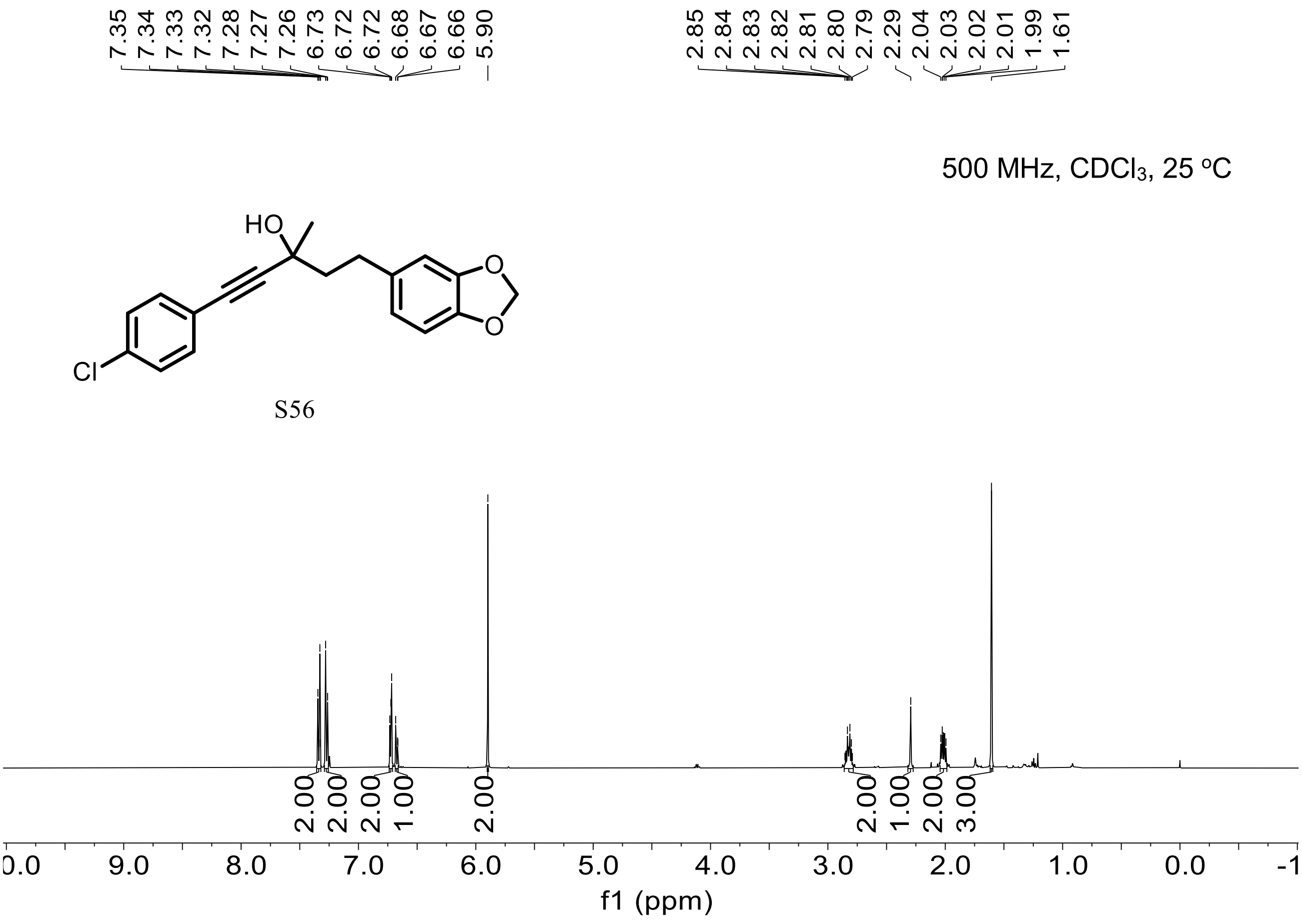
7.35 7.34 7.33 7.32 7.28 7.27 7.26 6.73 6.72 6.72 6.68 6.67 6.66 5.90
2.85 2.84 2.83 2.82 2.81 2.80 2.79 2.29 2.04 2.03 2.02 2.01 1.99 1.61
500 MHz, $CDCl_3$, 25 °C
HO
O
O
Cl
S56
2.00 2.00 2.00 1.00 2.00
2.00 1.00 2.00 3.00
f1 (ppm)

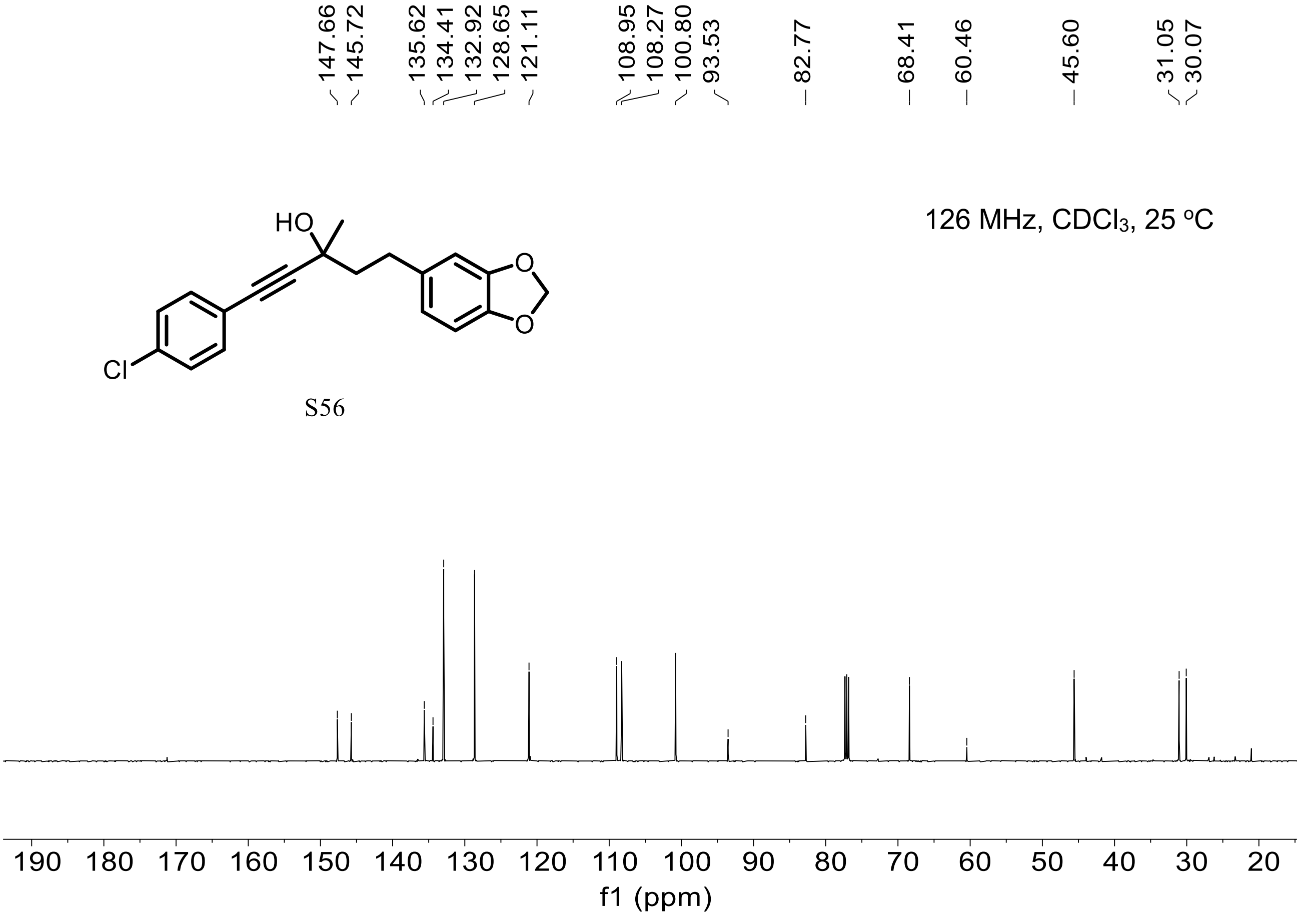
147.66 145.72 135.62 134.41 132.92 128.65 121.11 108.95 108.27 100.80 93.53 82.77 68.41 60.46 45.60 31.05 30.07
126 MHz, $CDCl_3$, 25 °C
HO
O
O
Cl
S56
f1 (ppm)

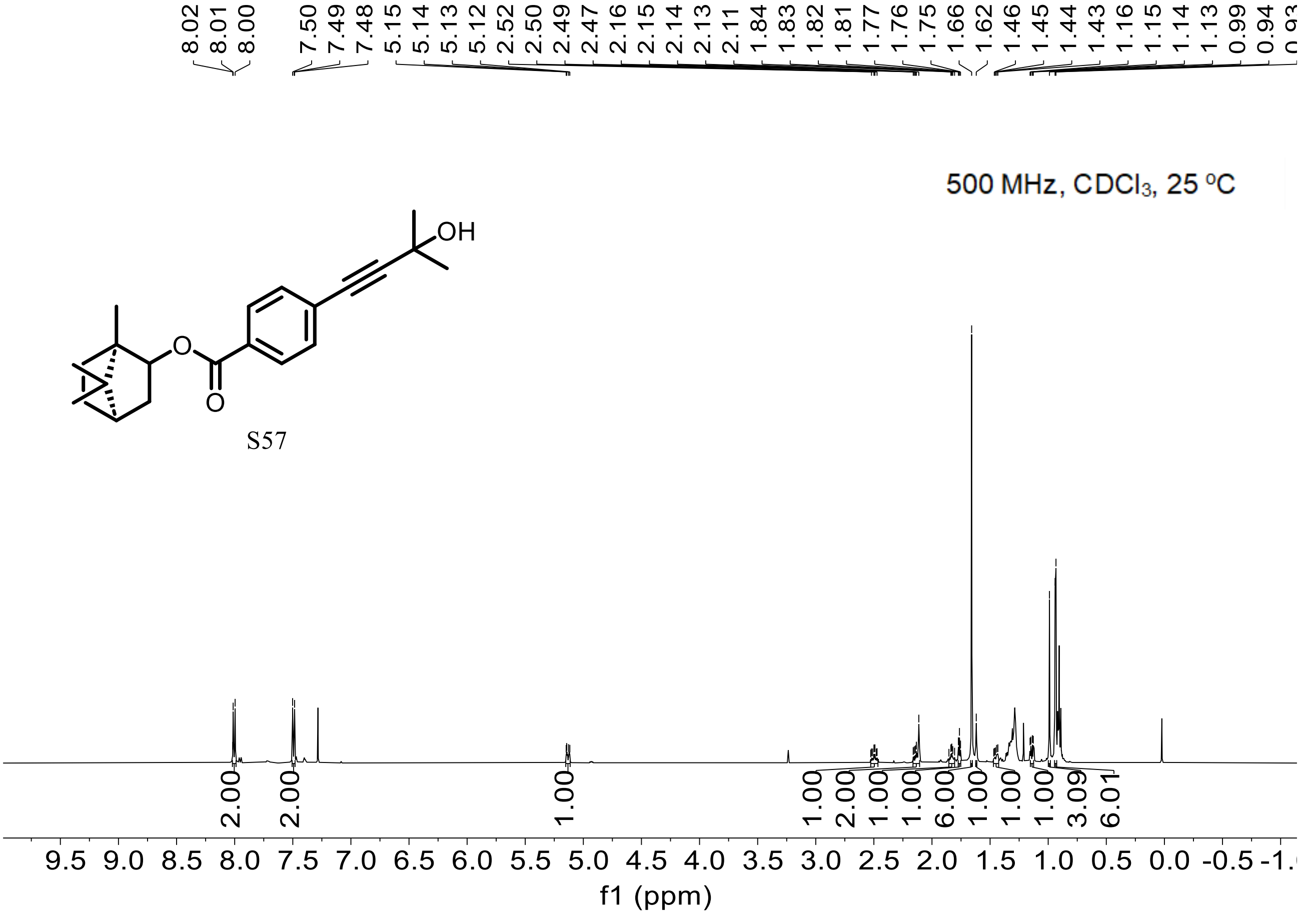
8.02 8.01 8.00 7.50 7.49 7.48 5.15 5.14 5.13 5.12 2.52 2.50 2.49 2.47 2.16 2.15 2.14 2.13 2.11 1.84 1.83 1.82 1.81 1.77 1.76 1.75 1.66 1.62 1.46 1.45 1.44 1.43 1.16 1.15 1.14 1.13 0.99 0.94
500 MHz, CDCl3, 25 °C
OH
O
O
S57
2.00 2.00 1.00 1.00 2.00 1.00 1.00 6.00 1.00 1.00 1.00 3.09 6.01
9.5 9.0 8.5 8.0 7.5 7.0 6.5 6.0 5.5 5.0 4.5 4.0 3.5 3.0 2.5 2.0 1.5 1.0 0.5 0.0 -0.5
f1 (ppm)

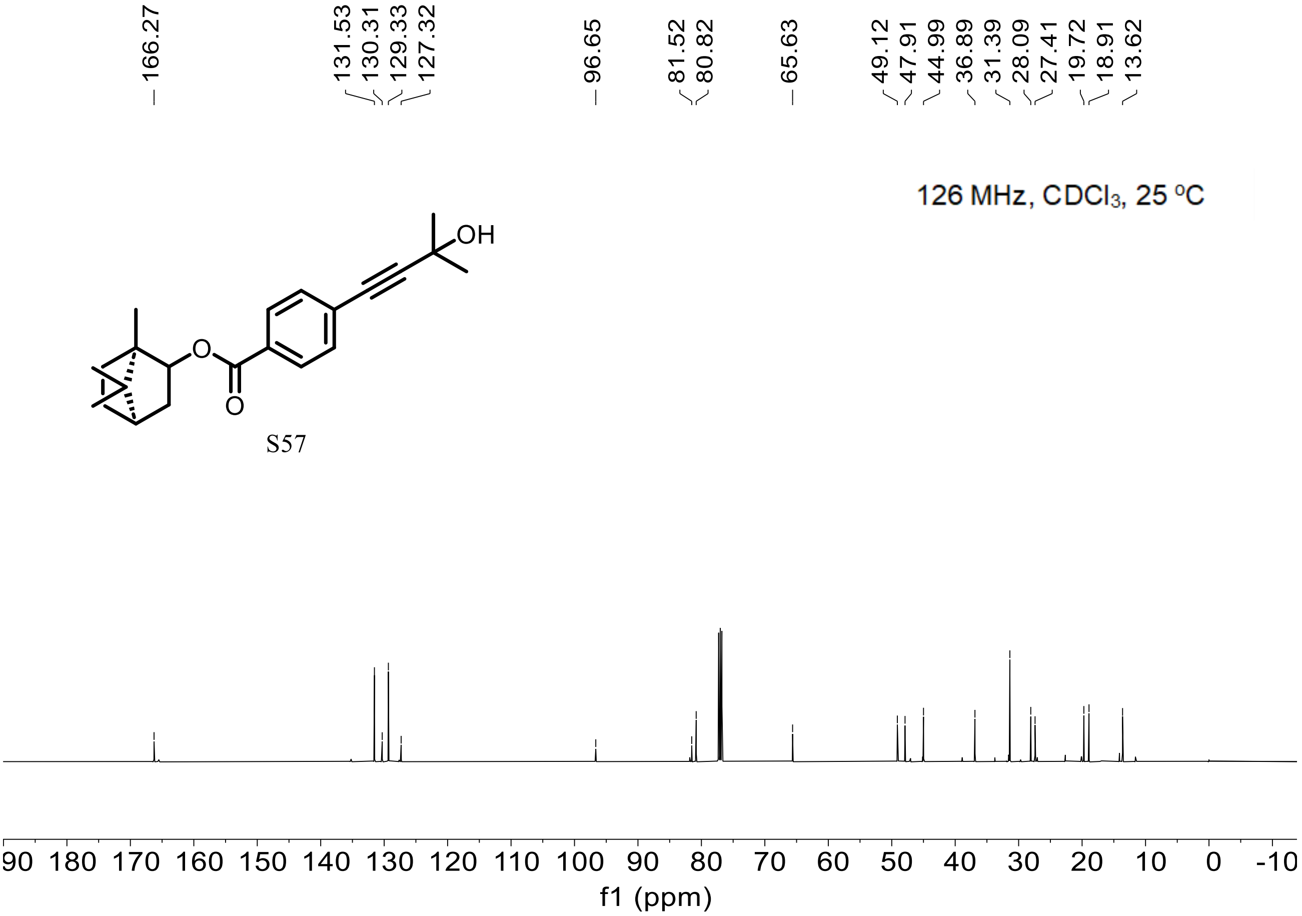
166.27
131.53 130.31 129.33 127.32
96.65
81.52 80.82
65.63
49.12 47.91 44.99 36.89 31.39 28.09 27.41 19.72 18.91 13.62
126 MHz, CDCl3, 25 °C
OH
O
O
S57
180 170 160 150 140 130 120 110 100 90 80 70 60 50 40 30 20 10 0 -10
f1 (ppm)

500 MHz, CDCl$_3$, 25 °C

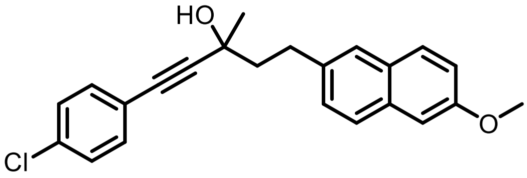

S59

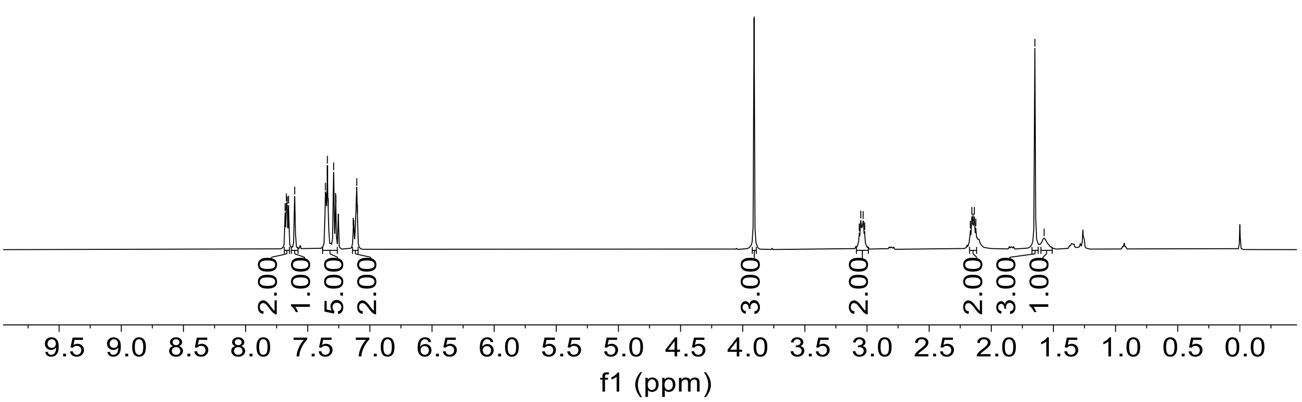

126 MHz, CDCl$_3$, 25 °C

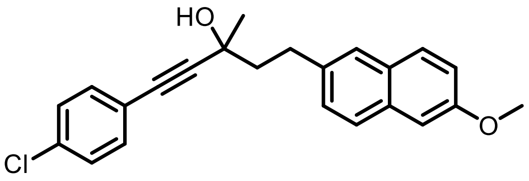

S59

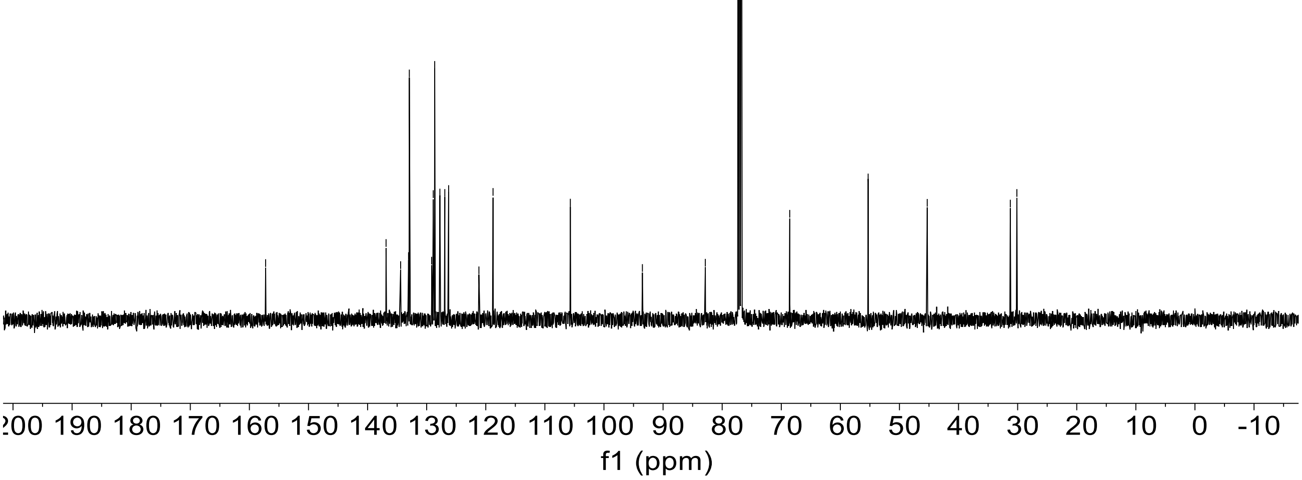

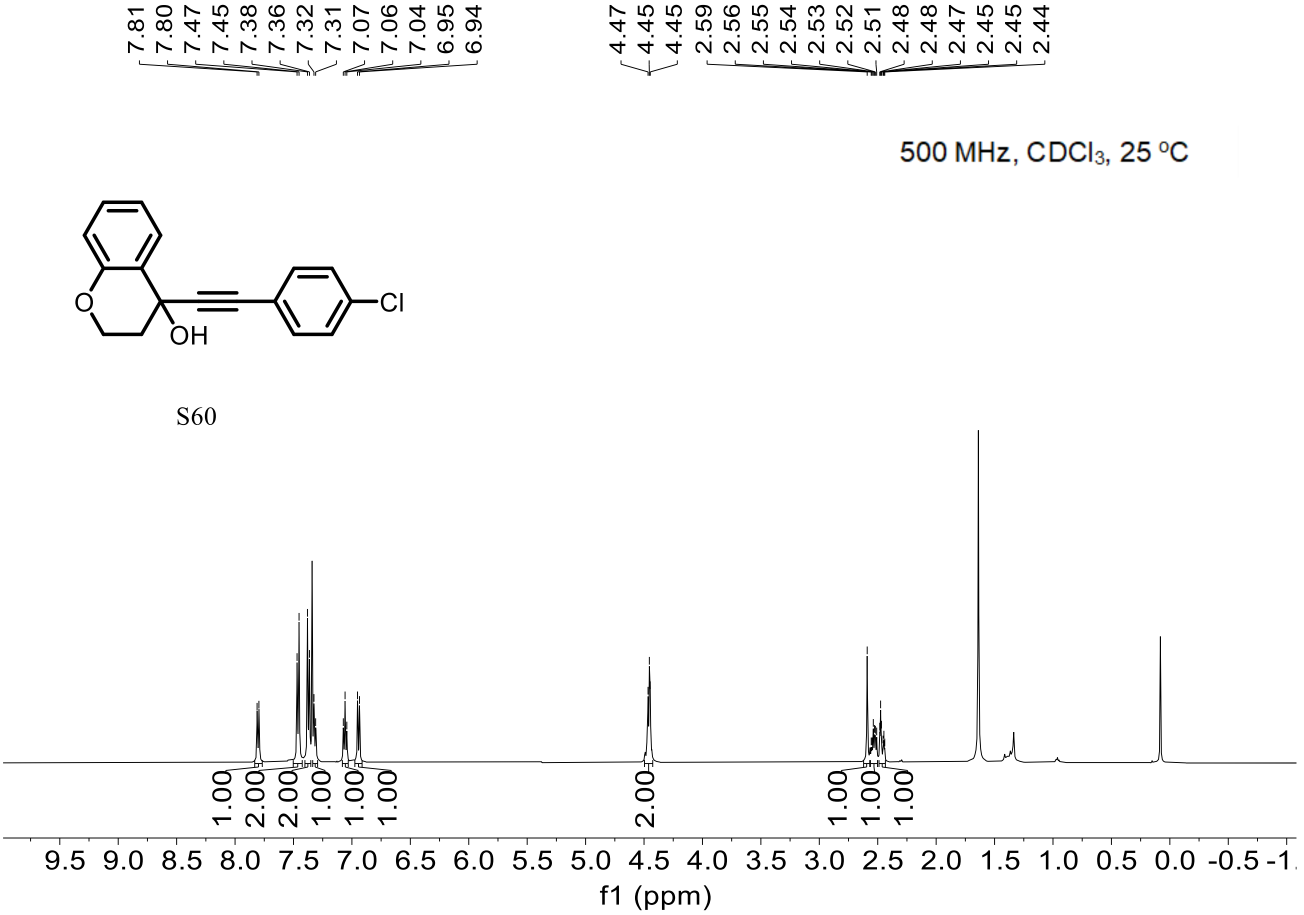
7.81 7.80 7.47 7.45 7.38 7.36 7.32 7.31 7.07 7.06 7.04 6.95 6.94
4.47 4.45 4.45 2.59 2.56 2.55 2.54 2.53 2.52 2.51 2.48 2.48 2.47 2.45 2.45 2.44
500 MHz, CDCl3, 25 °C
OH
Cl
O
S60
1.00 2.00 2.00 1.00 1.00 1.00
2.00
1.00 1.00 1.00
9.5 9.0 8.5 8.0 7.5 7.0 6.5 6.0 5.5 5.0 4.5 4.0 3.5 3.0 2.5 2.0 1.5 1.0 0.5 0.0 -0.5 -1.
f1 (ppm)

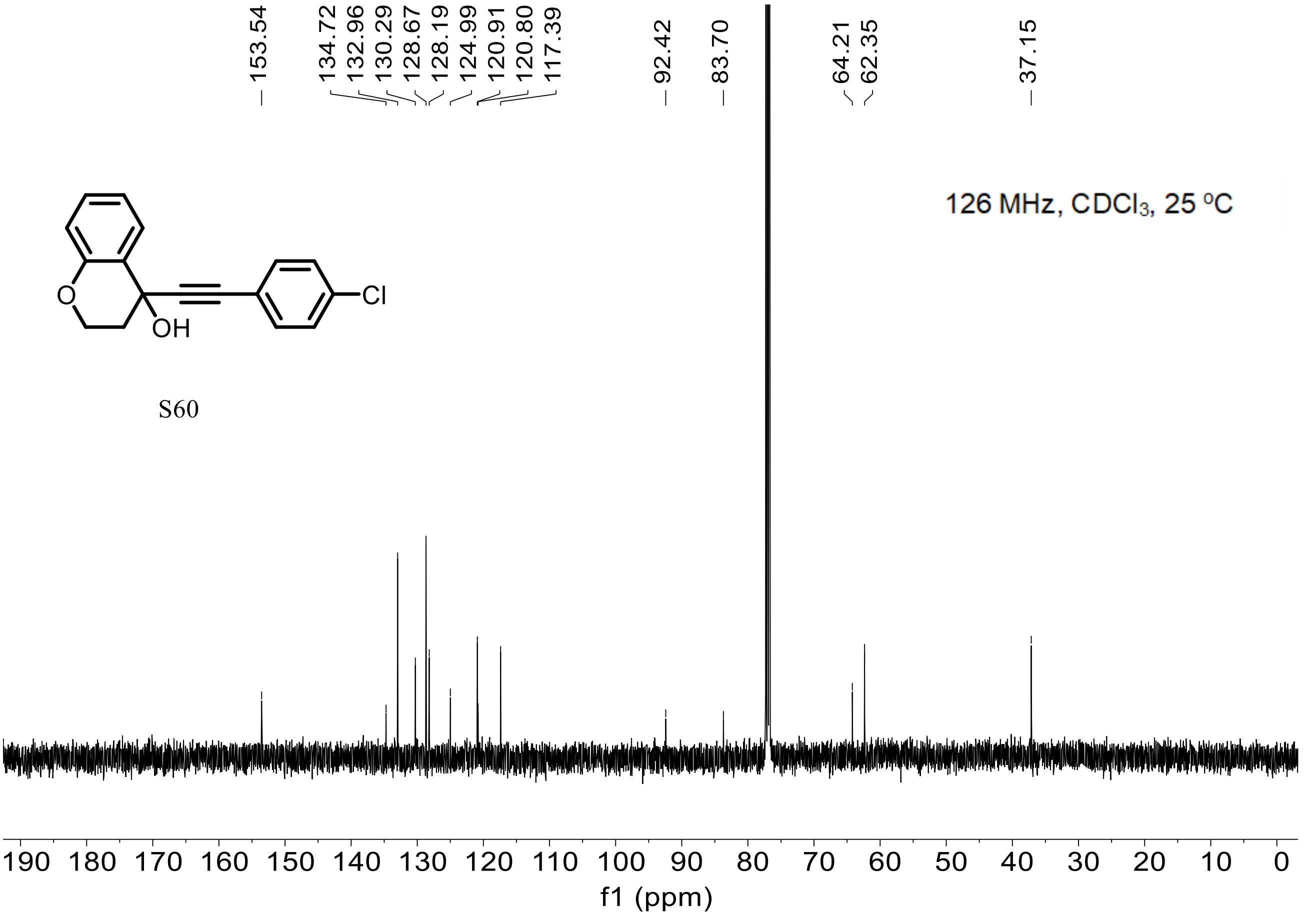
153.54
134.72 132.96 130.29 128.67 128.19 124.99 120.91 120.80 117.39
92.42
83.70
64.21 62.35
37.15
126 MHz, CDCl3, 25 °C
OH
Cl
O
S60
190 180 170 160 150 140 130 120 110 100 90 80 70 60 50 40 30 20 10 0
f1 (ppm)

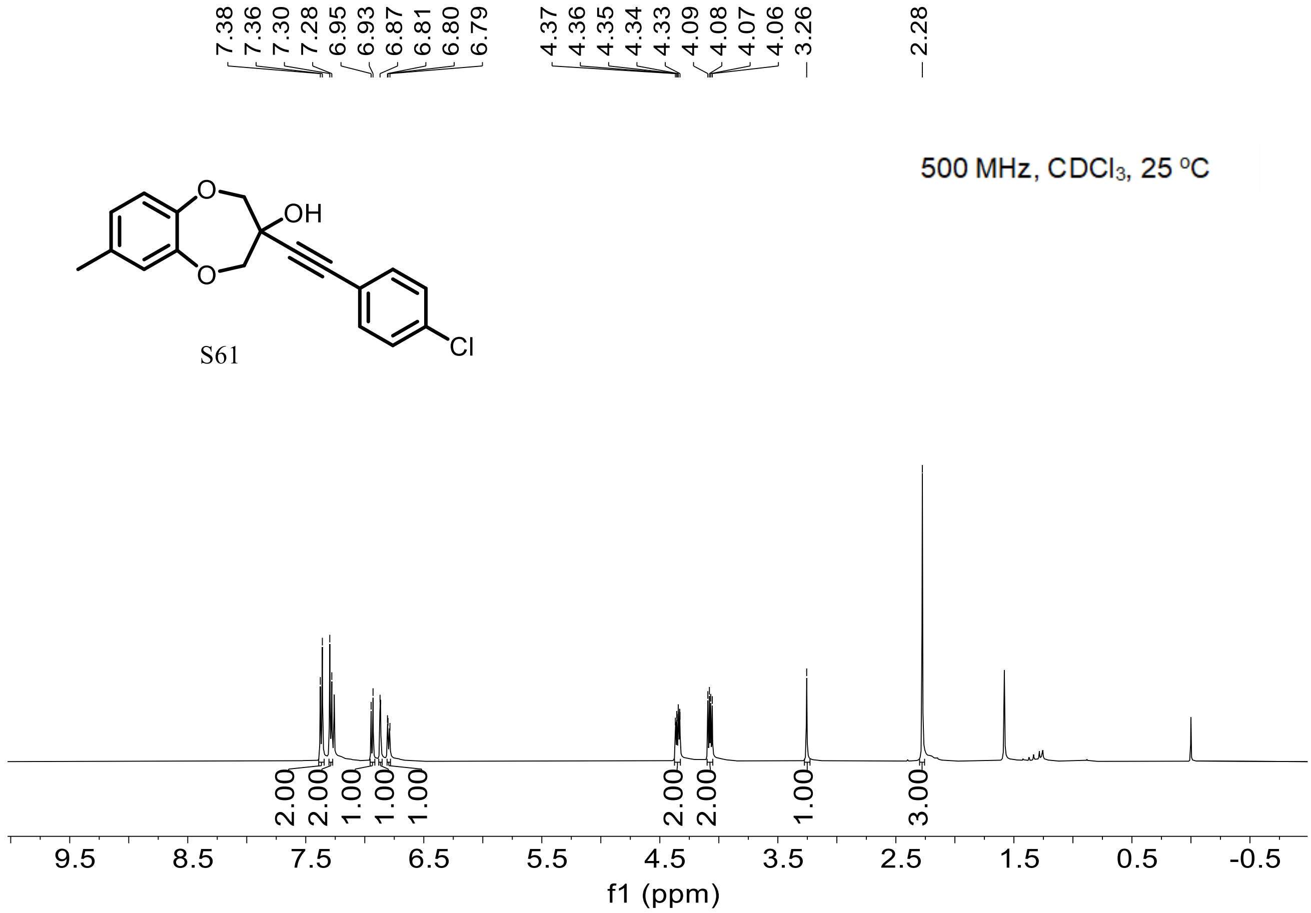
7.38 7.36 7.30 7.28 6.95 6.93 6.87 6.81 6.80 6.79 4.37 4.36 4.35 4.34 4.33 4.09 4.08 4.07 4.06 3.26 2.28
500 MHz, CDCl3, 25 °C
OH
O
O
Cl
S61
2.00 2.00 1.00 1.00 1.00 2.00 2.00 1.00 3.00
9.5 8.5 7.5 6.5 5.5 4.5 3.5 2.5 1.5 0.5 -0.5
f1 (ppm)

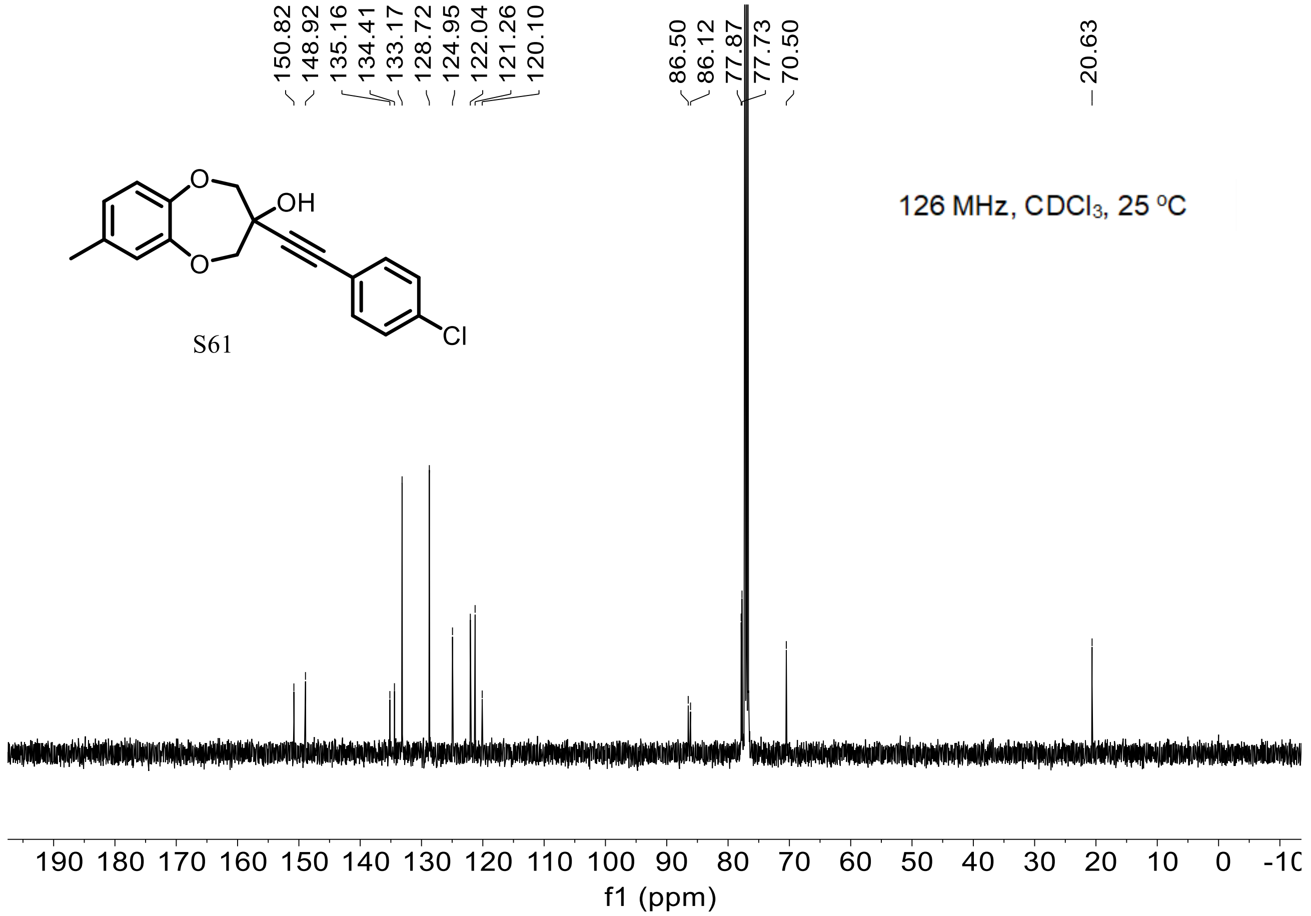
150.82 148.92 135.16 134.41 133.17 128.72 124.95 122.04 121.26 120.10 86.50 86.12 77.87 77.73 70.50 20.63
126 MHz, CDCl3, 25 °C
OH
O
O
Cl
S61
190 180 170 160 150 140 130 120 110 100 90 80 70 60 50 40 30 20 10 0 -10
f1 (ppm)

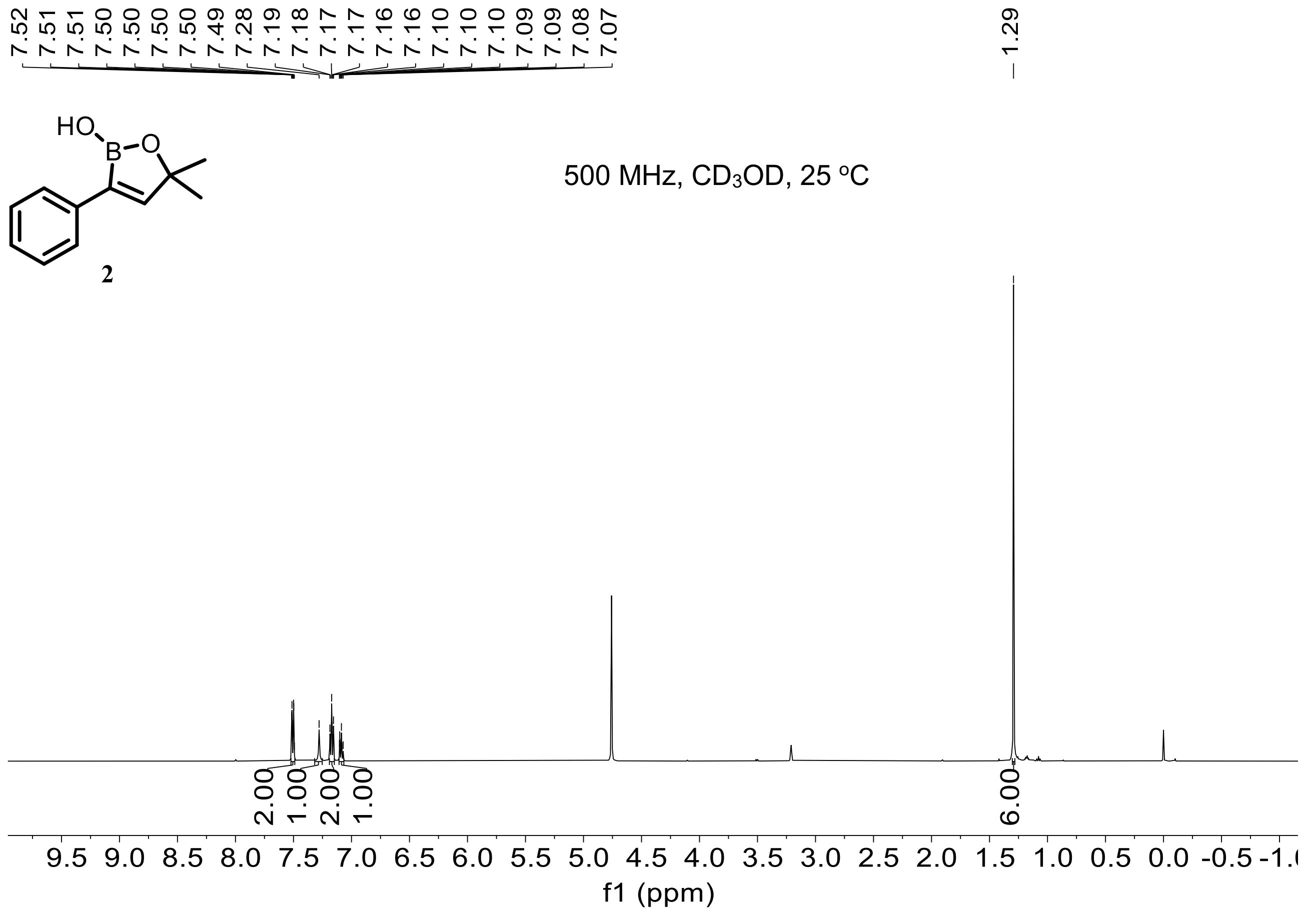
7.52 7.51 7.51 7.50 7.50 7.50 7.50 7.49 7.28 7.19 7.18 7.17 7.17 7.16 7.16 7.10 7.10 7.10 7.09 7.09 7.08 7.07
1.29
HO
B
O
2
500 MHz, CD3OD, 25 °C
2.00 1.00 2.00 1.00
6.00
9.5 9.0 8.5 8.0 7.5 7.0 6.5 6.0 5.5 5.0 4.5 4.0 3.5 3.0 2.5 2.0 1.5 1.0 0.5 0.0 -0.5
f1 (ppm)

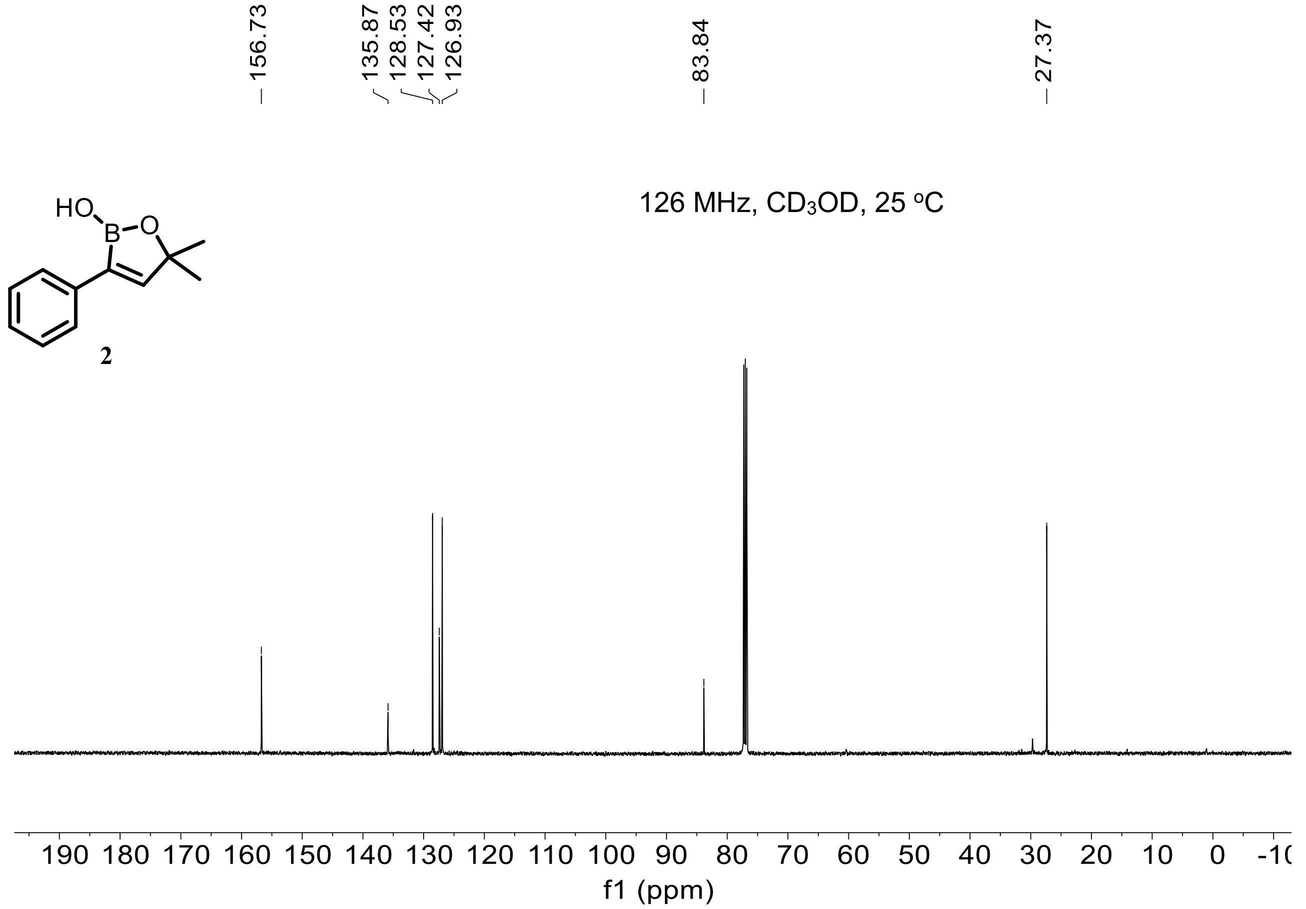
156.73
135.87 128.53 127.42 126.93
83.84
27.37
HO
B
O
2
126 MHz, CD3OD, 25 °C
190 180 170 160 150 140 130 120 110 100 90 80 70 60 50 40 30 20 10 0
f1 (ppm)

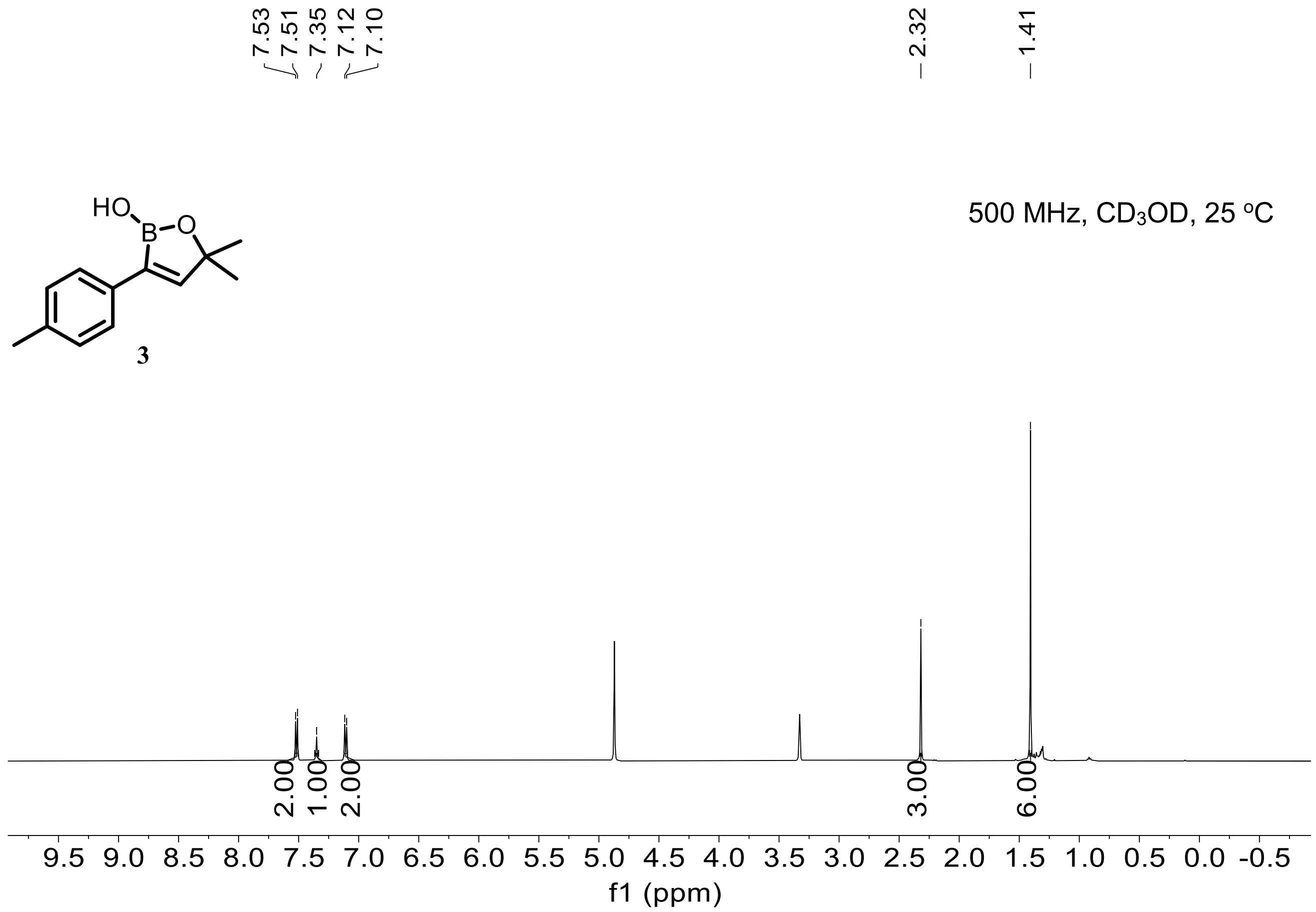
7.53
7.51
7.35
7.12
7.10
2.32
1.41
HO
B
O
3
500 MHz, CD3OD, 25 °C
2.00
1.00
2.00
3.00
6.00
9.5 9.0 8.5 8.0 7.5 7.0 6.5 6.0 5.5 5.0 4.5 4.0 3.5 3.0 2.5 2.0 1.5 1.0 0.5 0.0 -0.5
f1 (ppm)

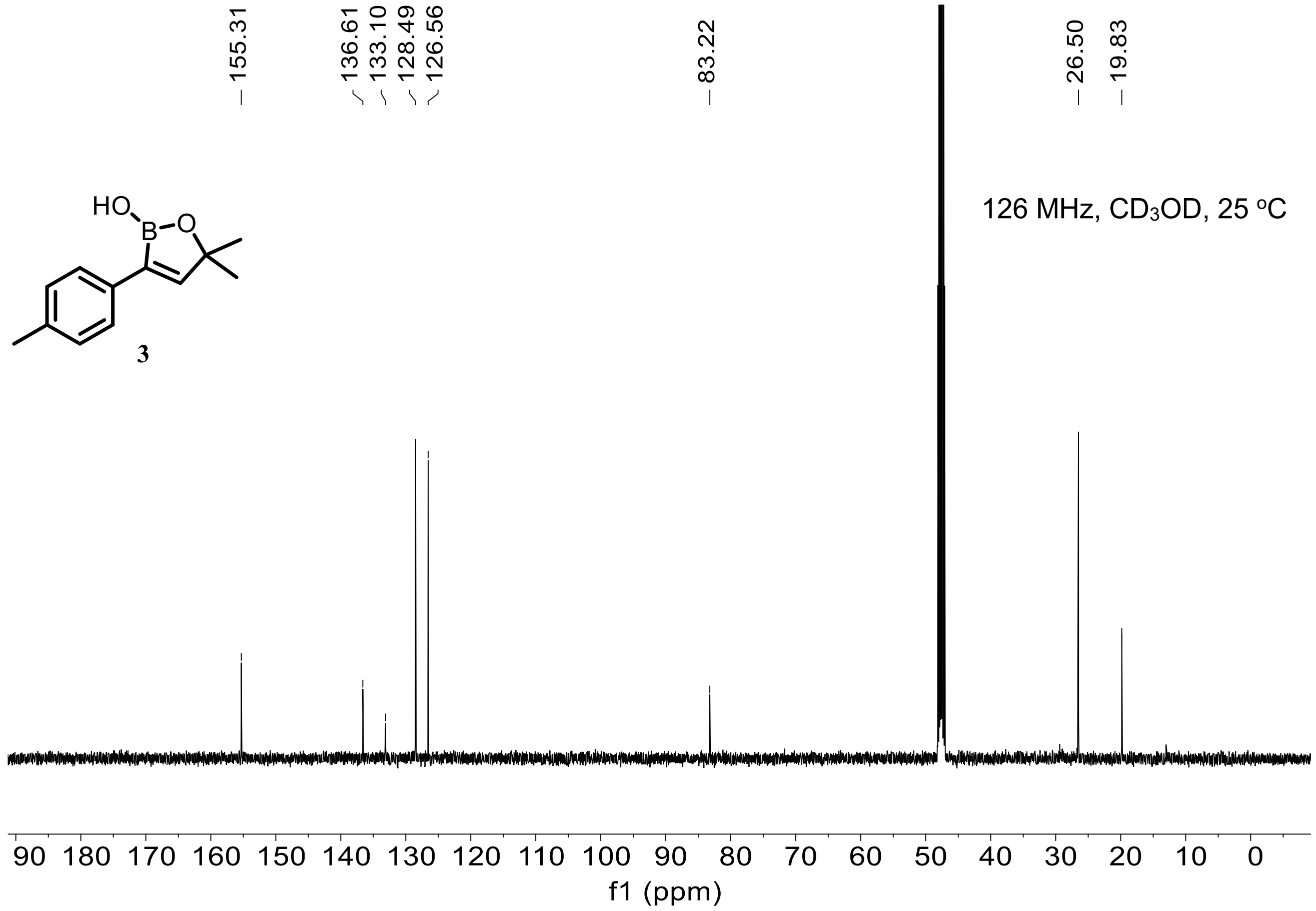
155.31
136.61
133.10
128.49
126.56
83.22
26.50
19.83
HO
B
O
3
126 MHz, CD3OD, 25 °C
90 180 170 160 150 140 130 120 110 100 90 80 70 60 50 40 30 20 10 0
f1 (ppm)

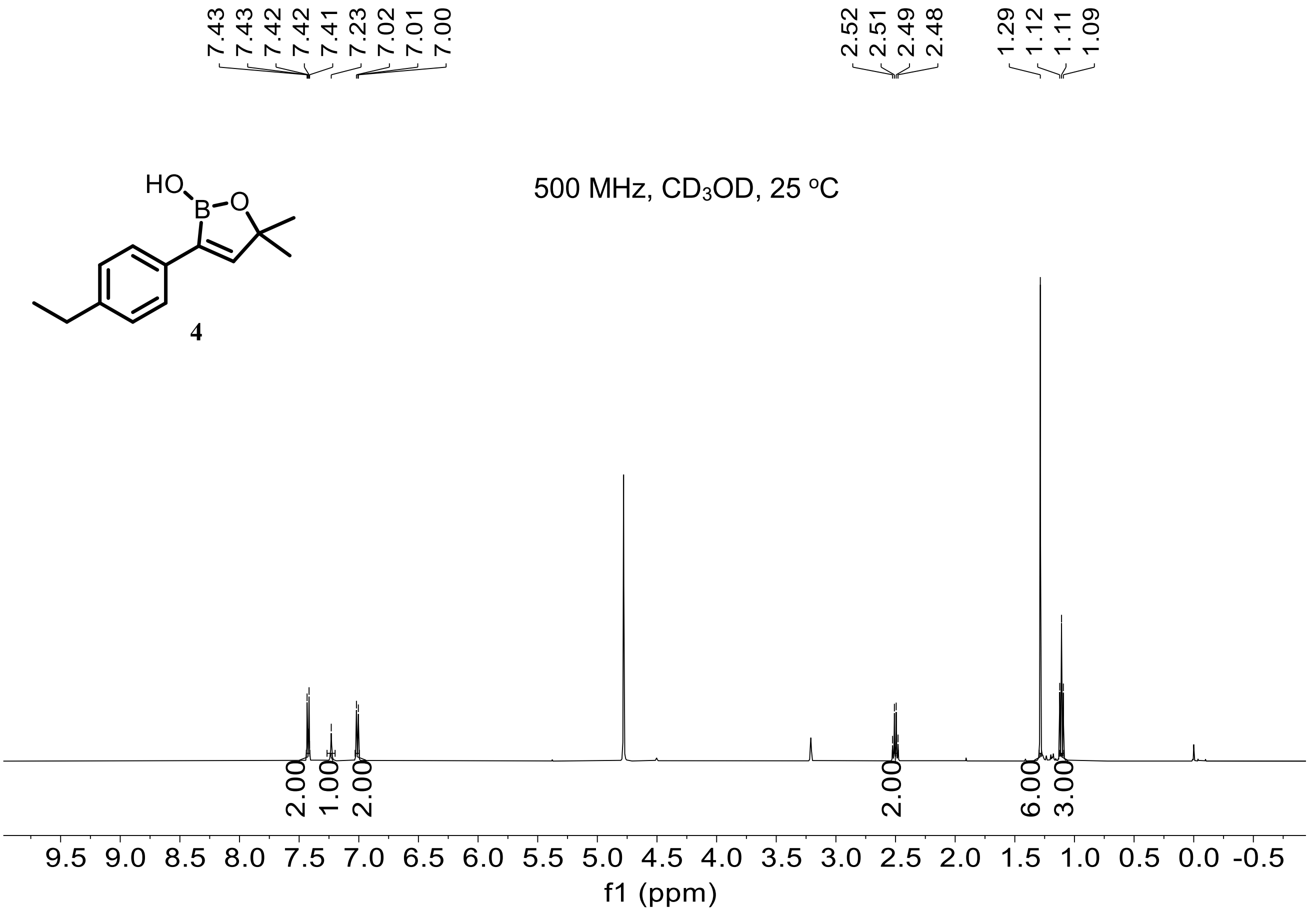
7.43 7.43 7.42 7.42 7.41 7.23 7.02 7.01 7.00
2.52 2.51 2.49 2.48
1.29 1.12 1.11 1.09
HO
B
O
4
500 MHz, CD3OD, 25 °C
2.00 1.00 2.00
2.00
6.00 3.00
9.5 9.0 8.5 8.0 7.5 7.0 6.5 6.0 5.5 5.0 4.5 4.0 3.5 3.0 2.5 2.0 1.5 1.0 0.5 0.0 -0.5
f1 (ppm)

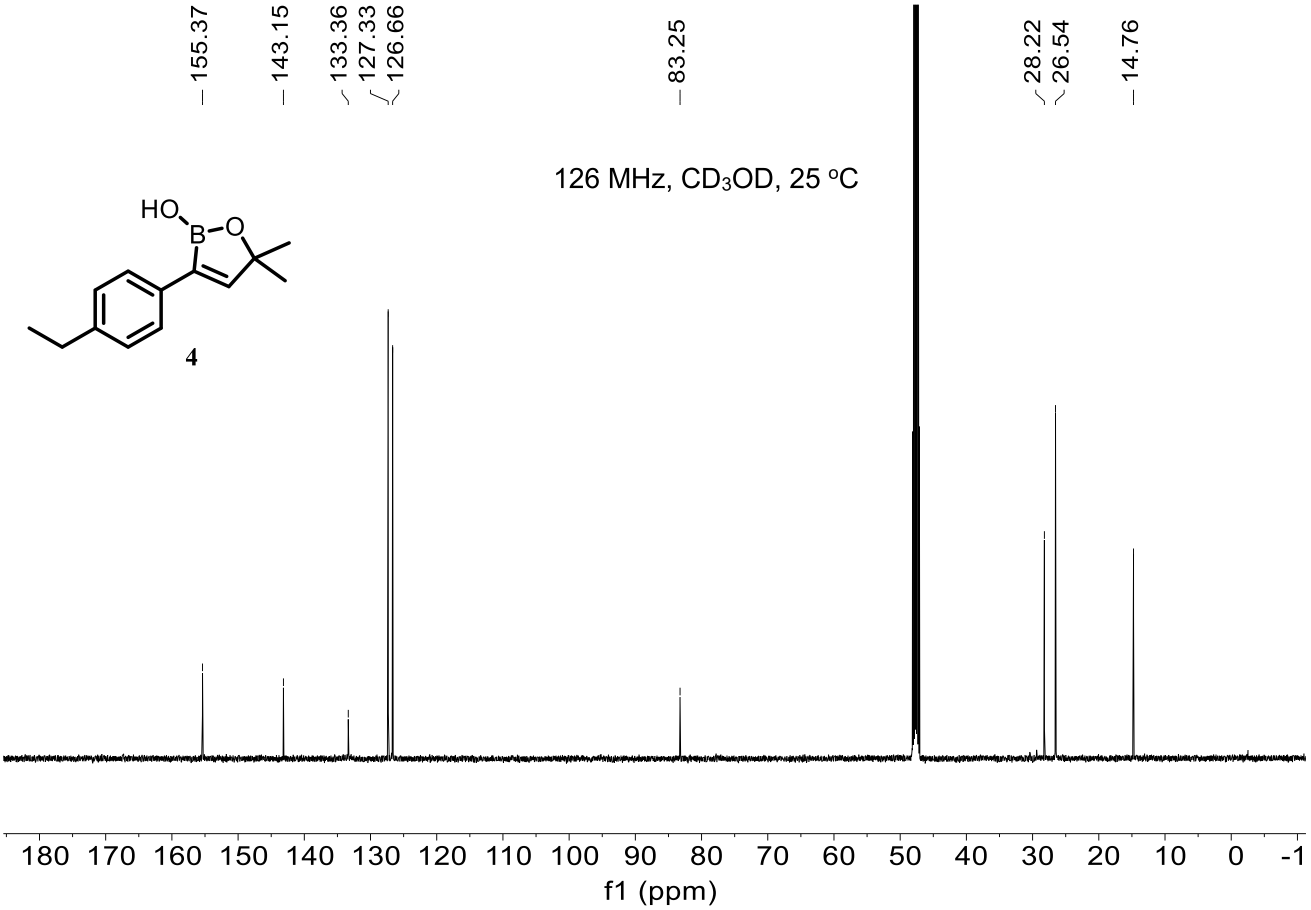
155.37
143.15
133.36 127.33 126.66
83.25
28.22 26.54
14.76
HO
B
O
4
126 MHz, CD3OD, 25 °C
180 170 160 150 140 130 120 110 100 90 80 70 60 50 40 30 20 10 0 -1
f1 (ppm)

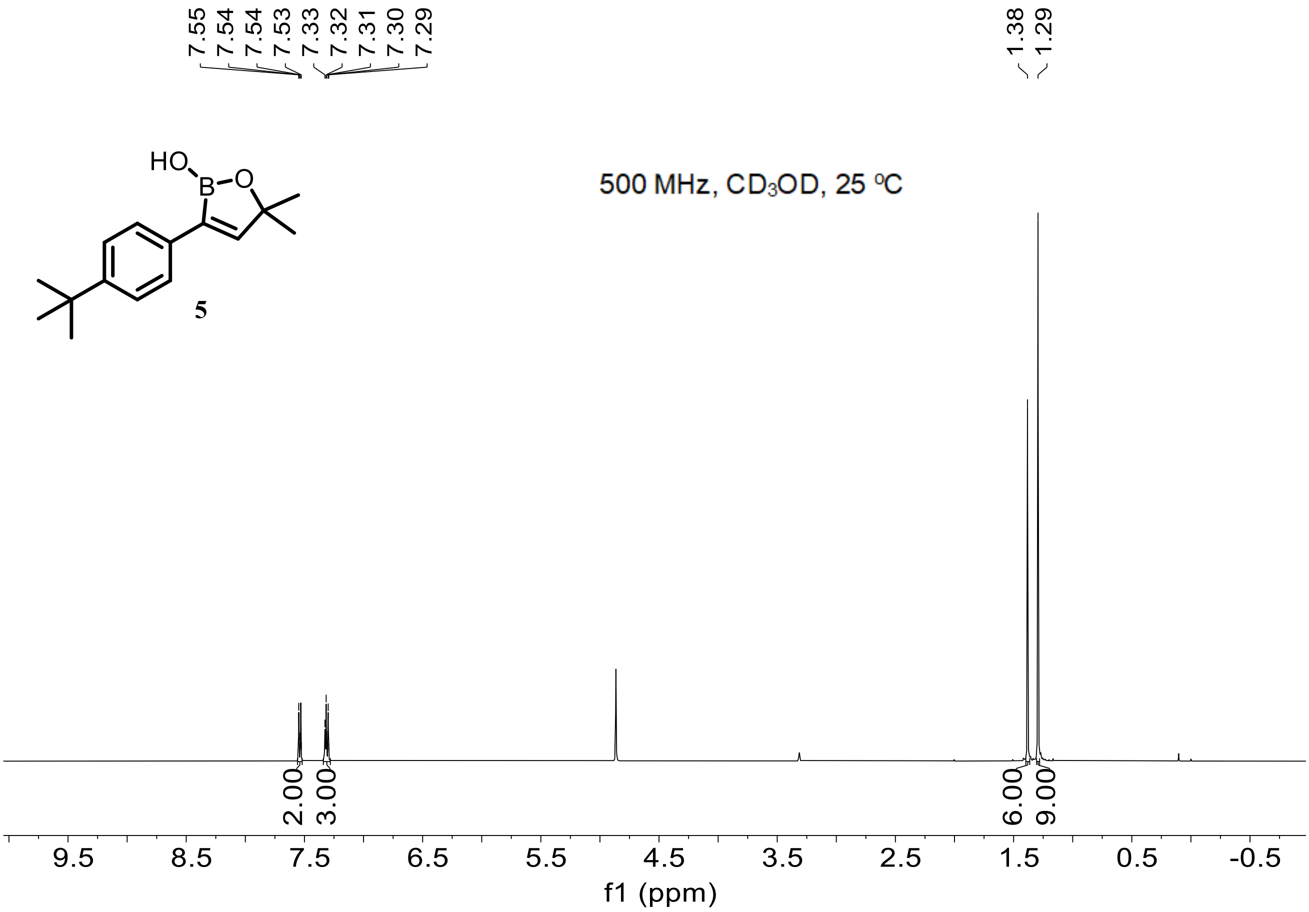
7.55
7.54
7.54
7.53
7.33
7.32
7.31
7.30
7.29
1.38
1.29
HO
B
O
5
500 MHz, CD3OD, 25 °C
2.00
3.00
6.00
9.00
9.5 8.5 7.5 6.5 5.5 4.5 3.5 2.5 1.5 0.5 -0.5
f1 (ppm)

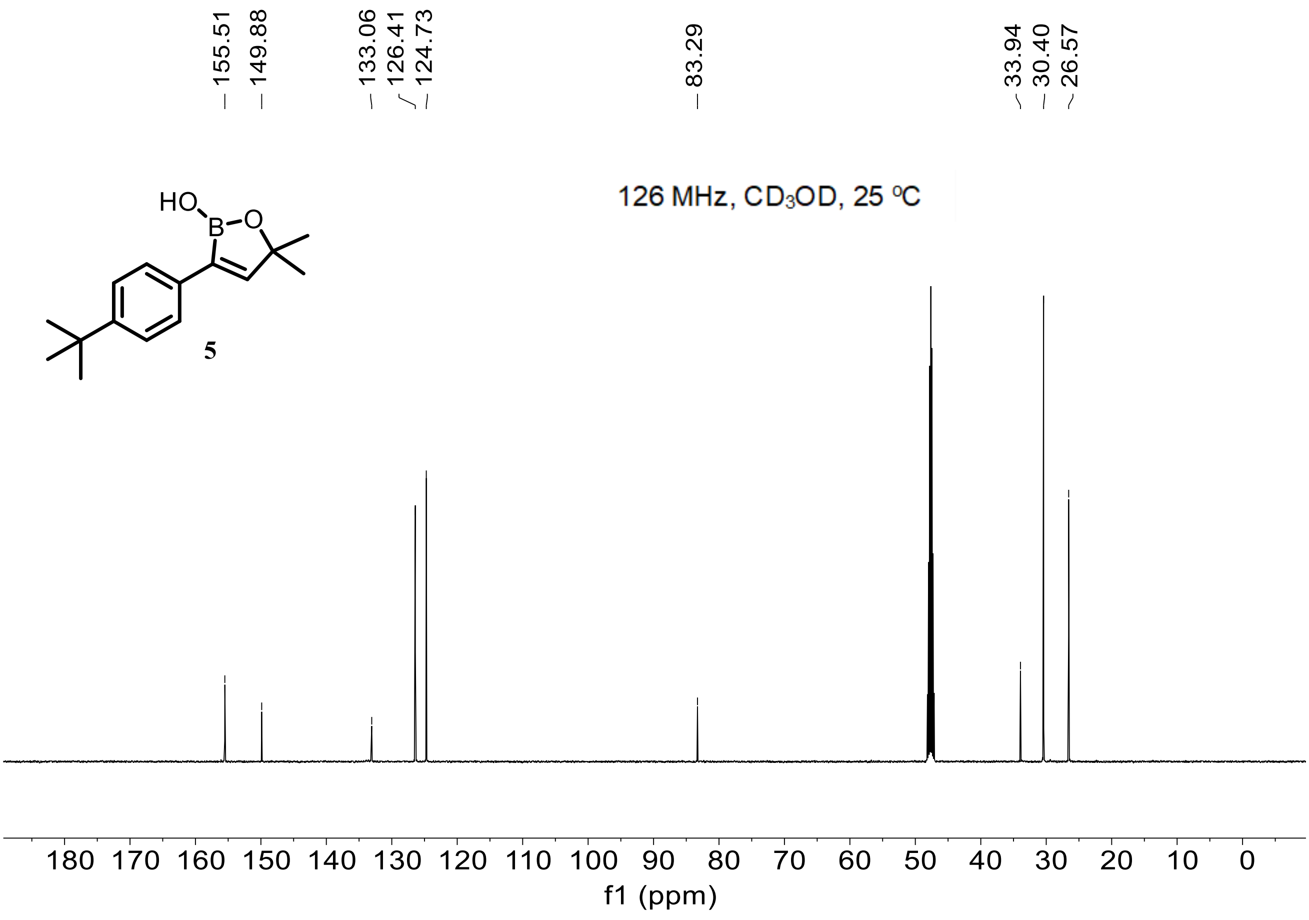
155.51
149.88
133.06
126.41
124.73
83.29
33.94
30.40
26.57
HO
B
O
5
126 MHz, CD3OD, 25 °C
180 170 160 150 140 130 120 110 100 90 80 70 60 50 40 30 20 10 0
f1 (ppm)

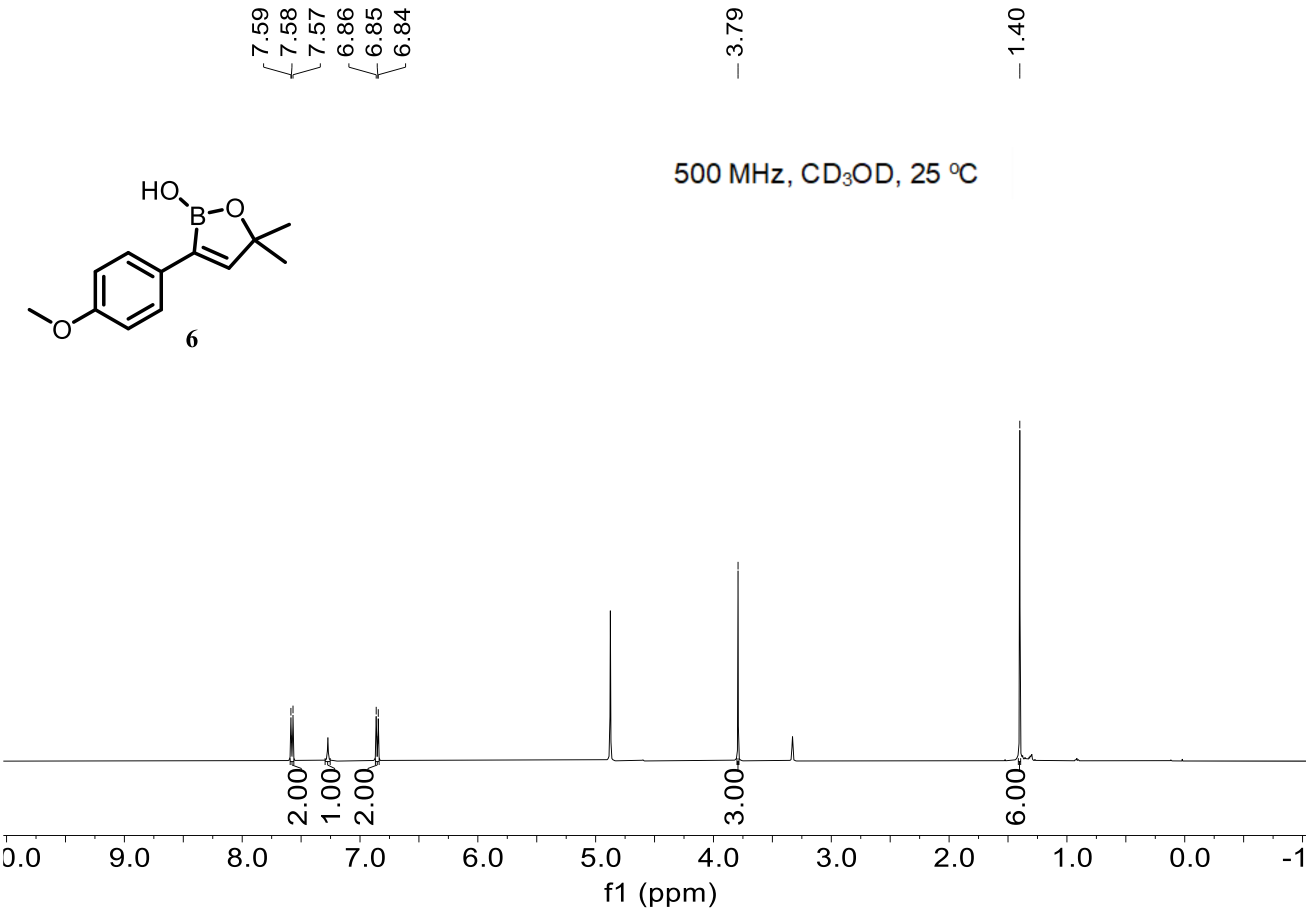
7.59
7.58
7.57
6.86
6.85
6.84
3.79
1.40
500 MHz, CD_3OD, 25 °C
HO
B
O
O
6
2.00
1.00
2.00
3.00
6.00
f1 (ppm)

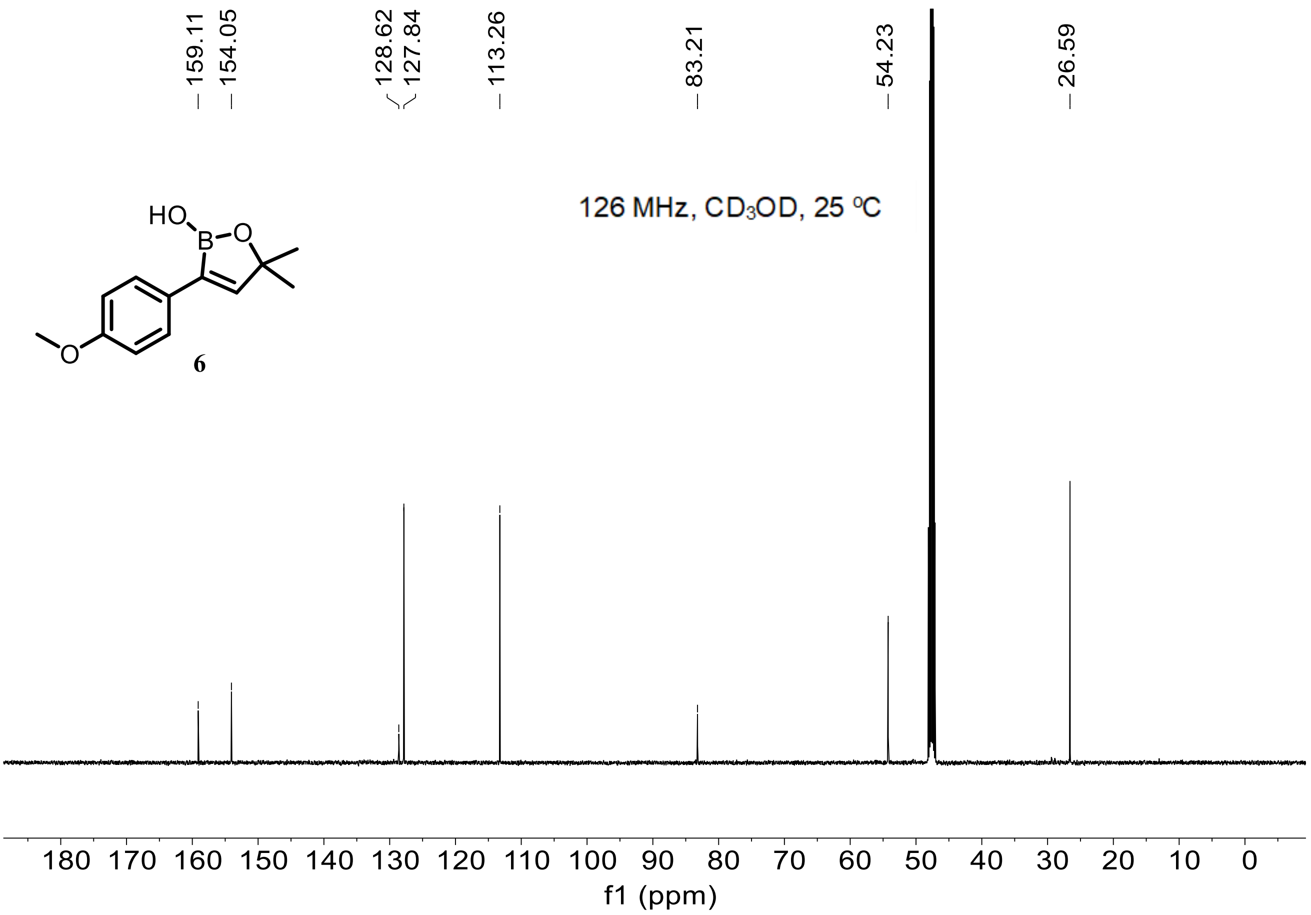
159.11
154.05
128.62
127.84
113.26
83.21
54.23
26.59
126 MHz, CD_3OD, 25 °C
HO
B
O
O
6
f1 (ppm)

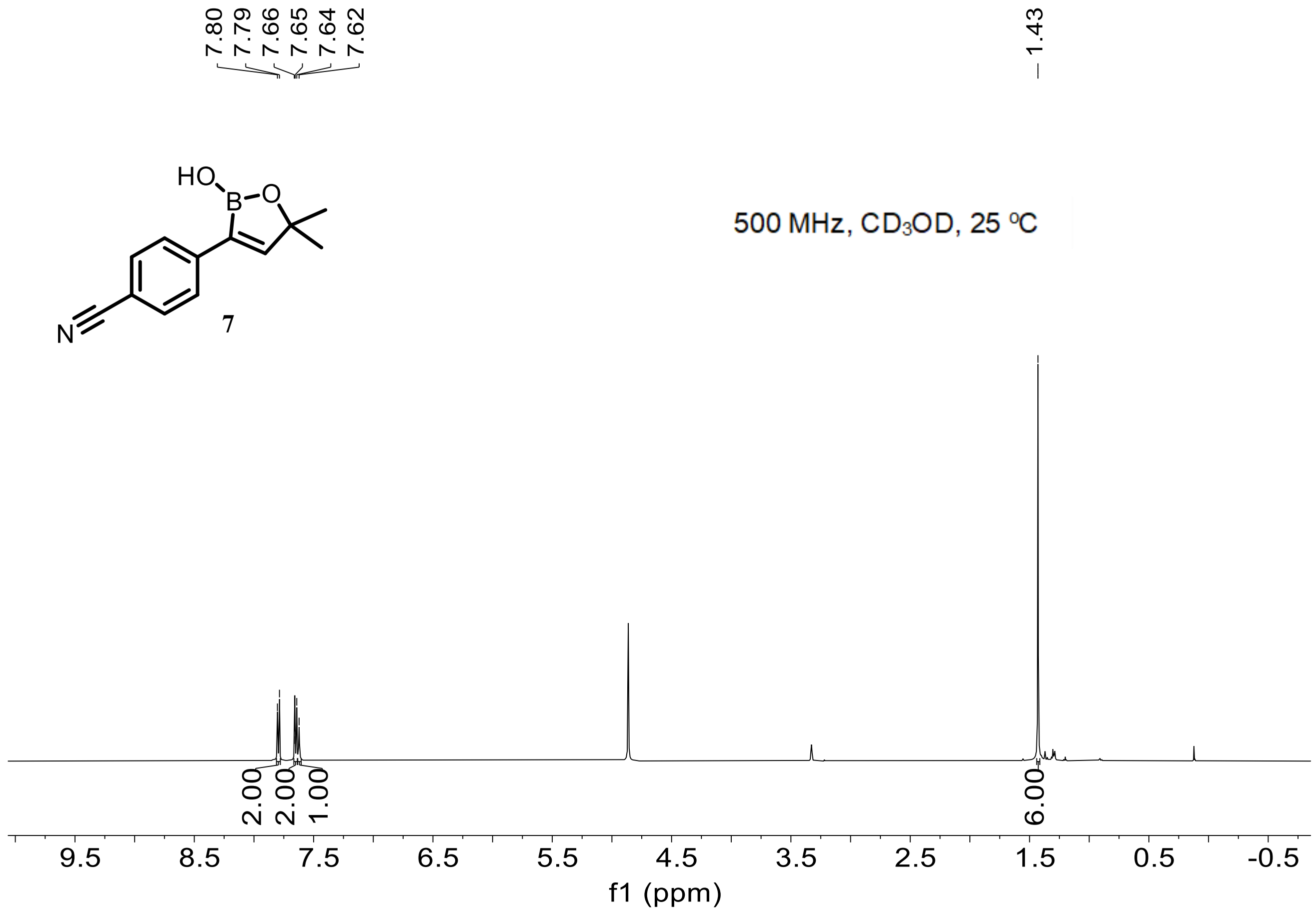
7.80
7.79
7.66
7.65
7.64
7.62
1.43
HO
B
O
N
7
500 MHz, CD3OD, 25 °C
2.00
2.00
1.00
6.00
9.5
8.5
7.5
6.5
5.5
4.5
3.5
2.5
1.5
0.5
-0.5
f1 (ppm)

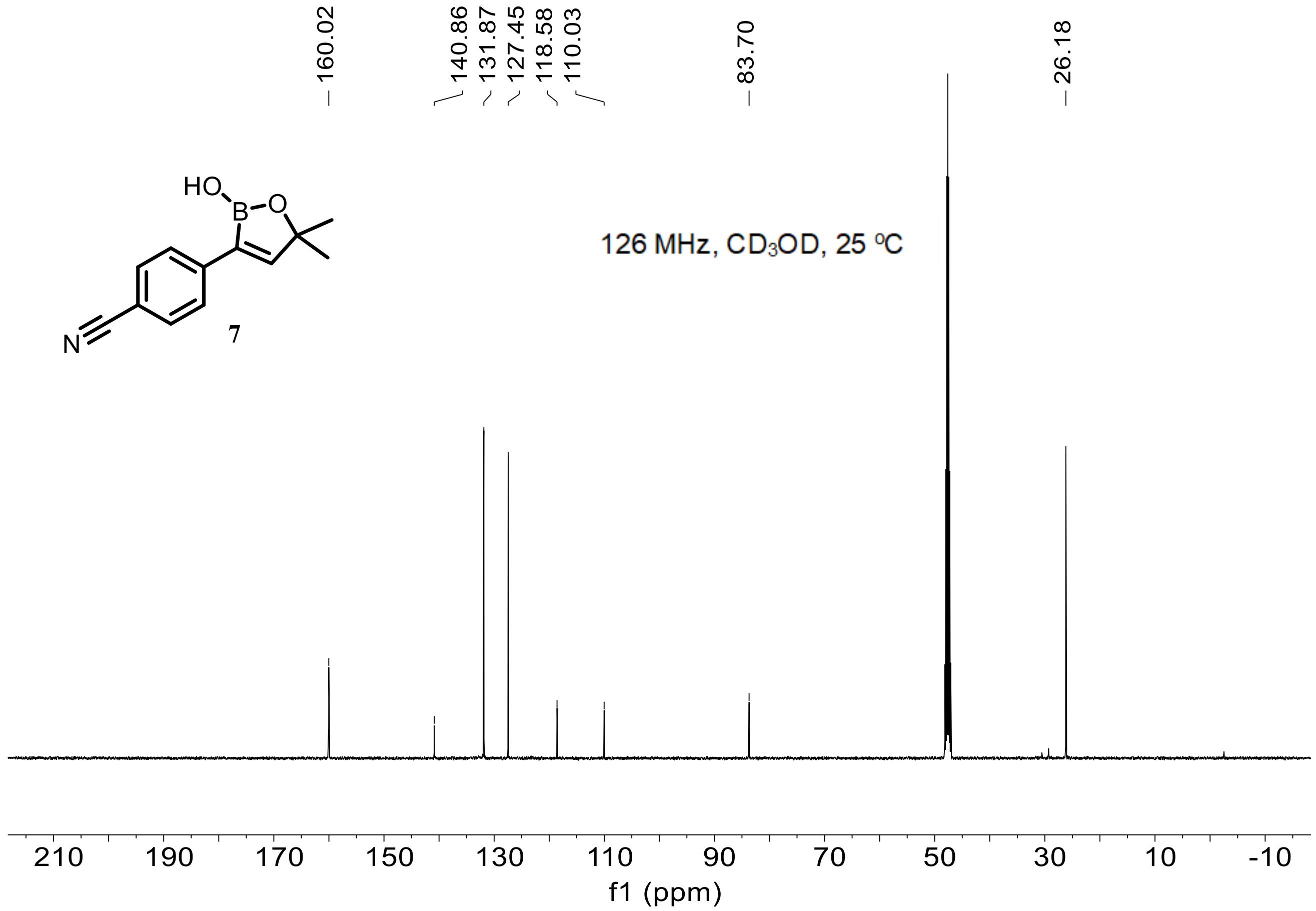
160.02
140.86
131.87
127.45
118.58
110.03
83.70
26.18
HO
B
O
N
7
126 MHz, CD3OD, 25 °C
210
190
170
150
130
110
90
70
50
30
10
-10
f1 (ppm)

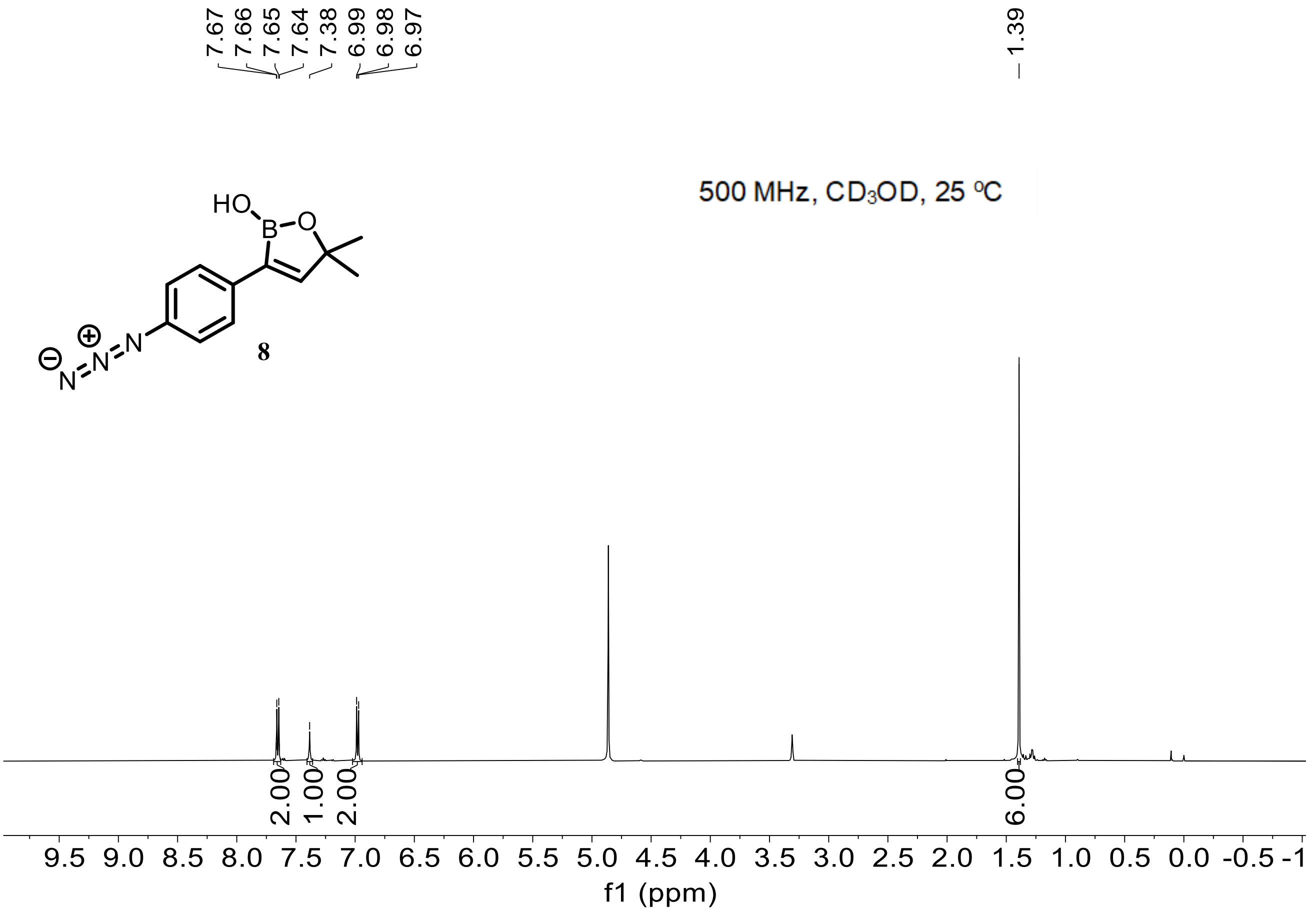
7.67
7.66
7.65
7.64
7.38
6.99
6.98
6.97
1.39
500 MHz, CD3OD, 25 °C
HO
B
O
N
N
N
8
2.00
1.00
2.00
6.00
9.5 9.0 8.5 8.0 7.5 7.0 6.5 6.0 5.5 5.0 4.5 4.0 3.5 3.0 2.5 2.0 1.5 1.0 0.5 0.0 -0.5 -1
f1 (ppm)

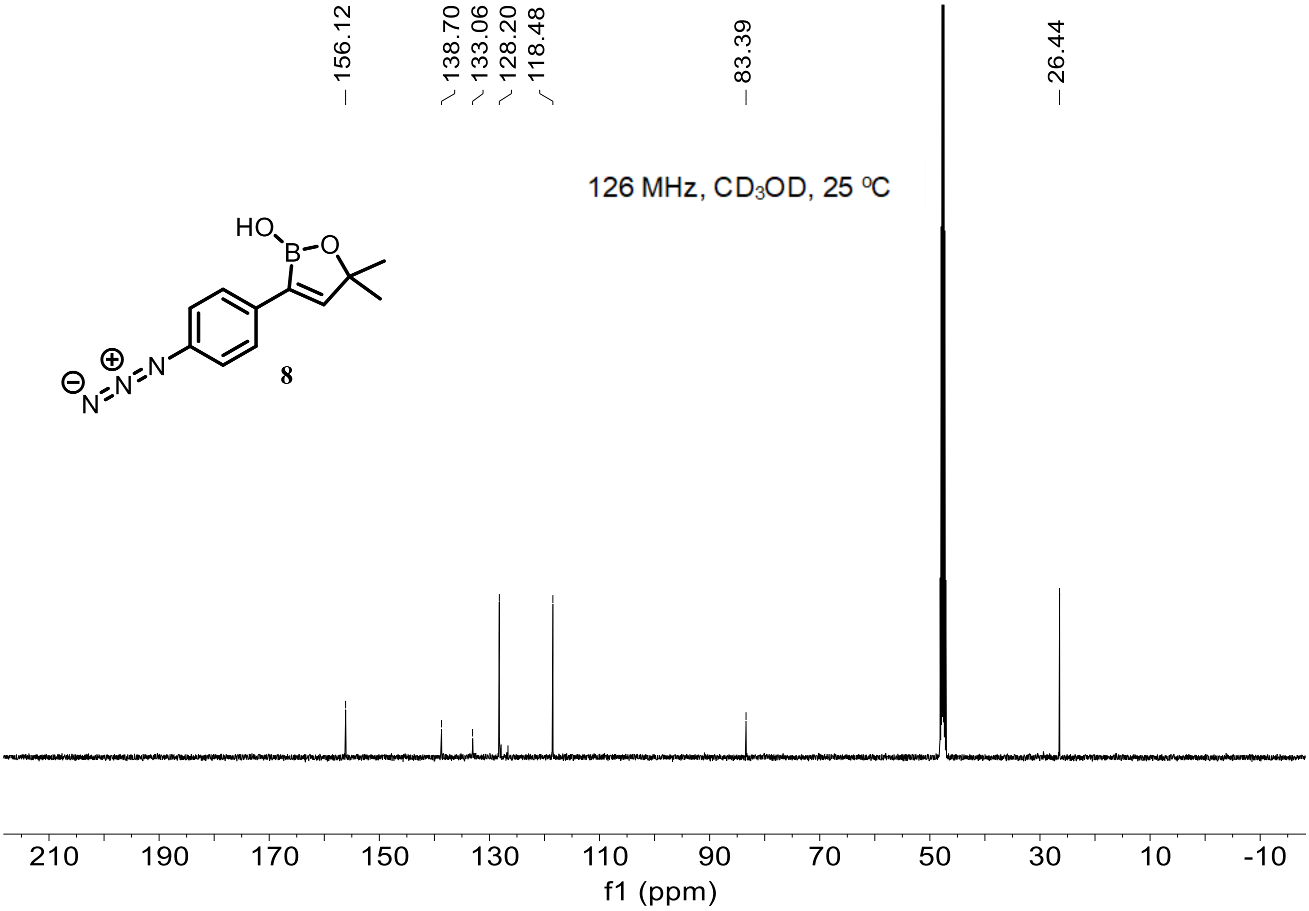
156.12
138.70
133.06
128.20
118.48
83.39
26.44
126 MHz, CD3OD, 25 °C
HO
B
O
N
N
N
8
210 190 170 150 130 110 90 70 50 30 10 -10
f1 (ppm)

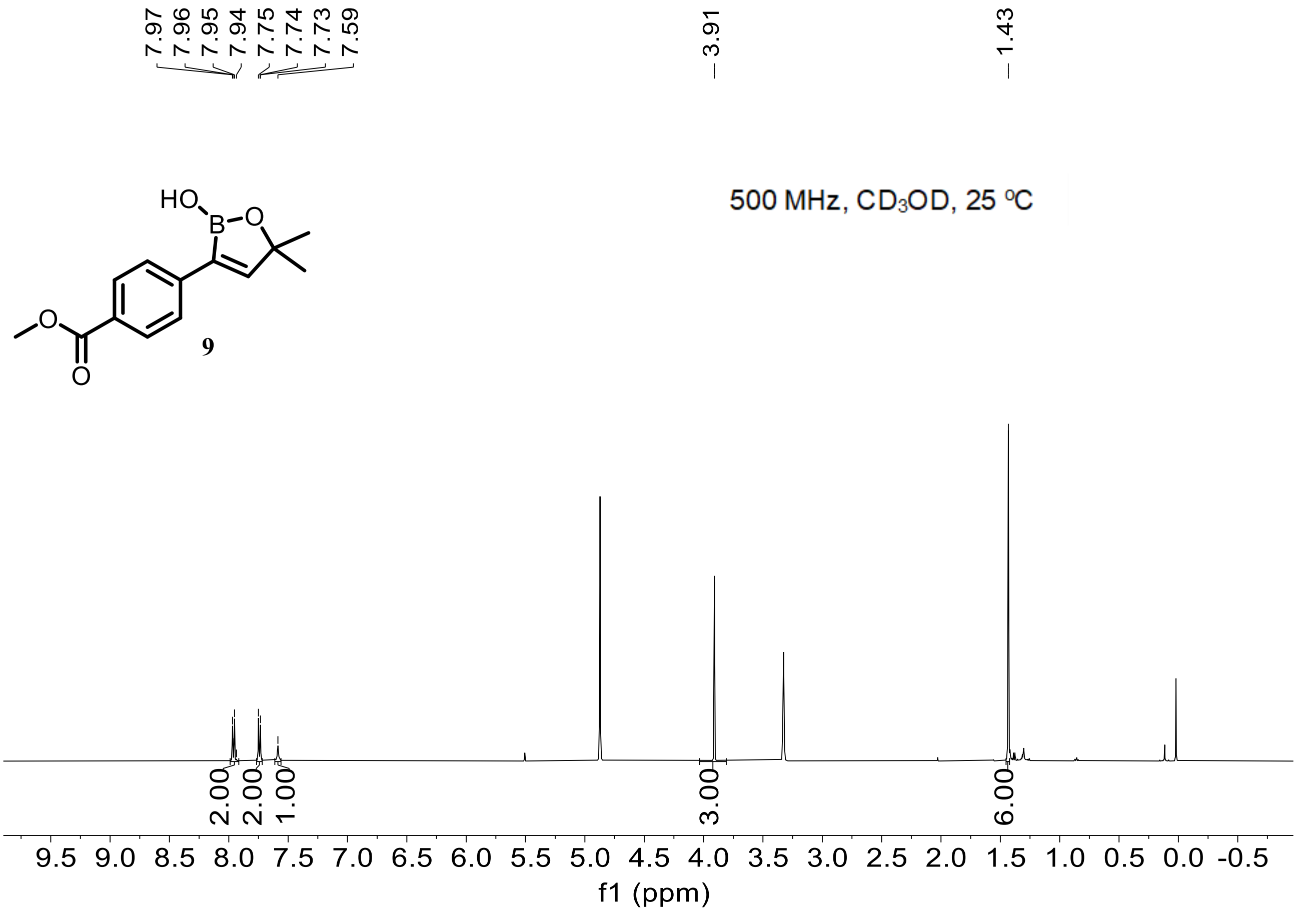
7.97
7.96
7.95
7.94
7.75
7.74
7.73
7.59
3.91
1.43
500 MHz, CD3OD, 25 °C
HO
B
O
O
O
9
2.00
2.00
1.00
3.00
6.00
9.5 9.0 8.5 8.0 7.5 7.0 6.5 6.0 5.5 5.0 4.5 4.0 3.5 3.0 2.5 2.0 1.5 1.0 0.5 0.0 -0.5
f1 (ppm)

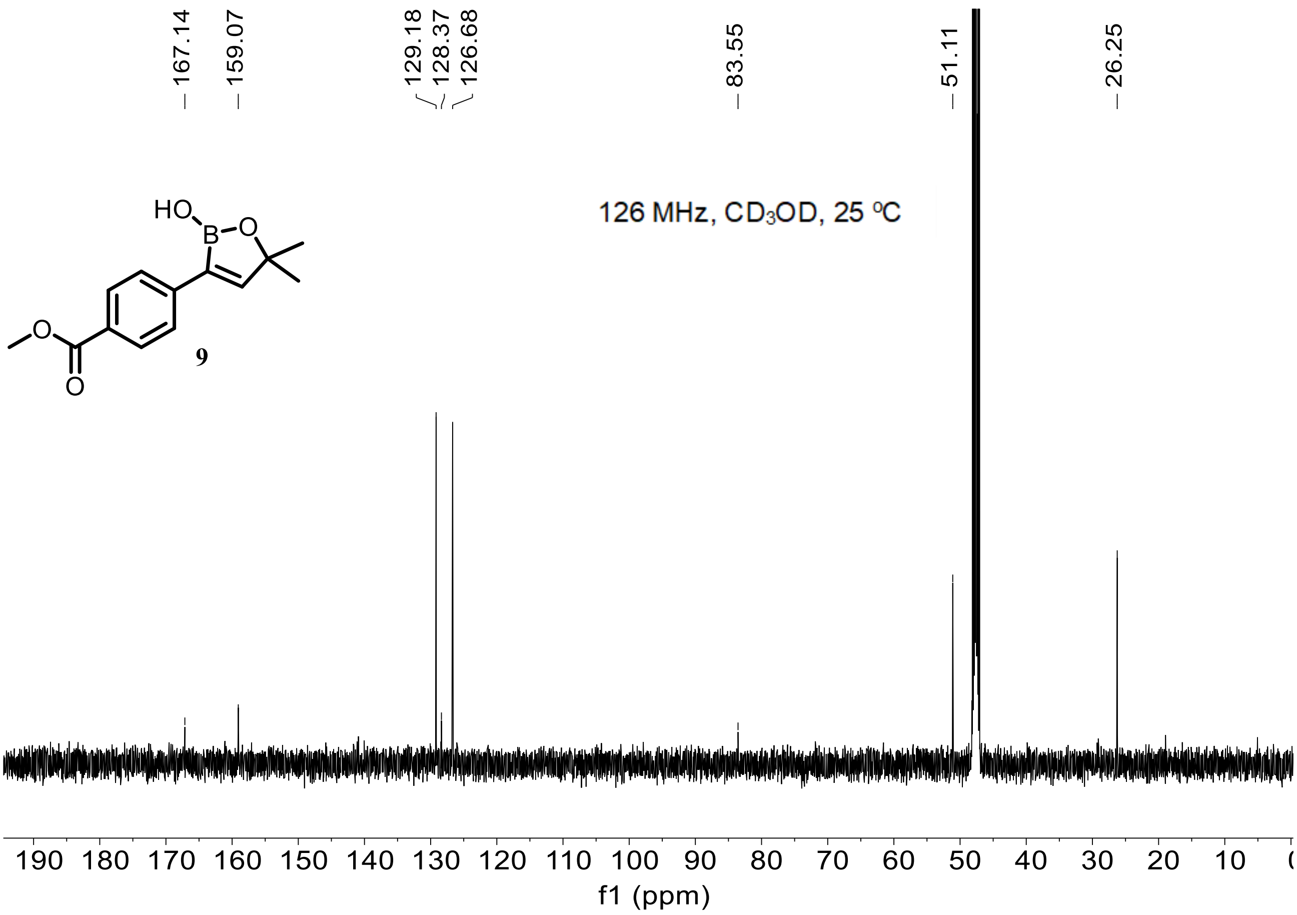
167.14
159.07
129.18
128.37
126.68
83.55
51.11
26.25
126 MHz, CD3OD, 25 °C
HO
B
O
O
O
9
190 180 170 160 150 140 130 120 110 100 90 80 70 60 50 40 30 20 10
f1 (ppm)

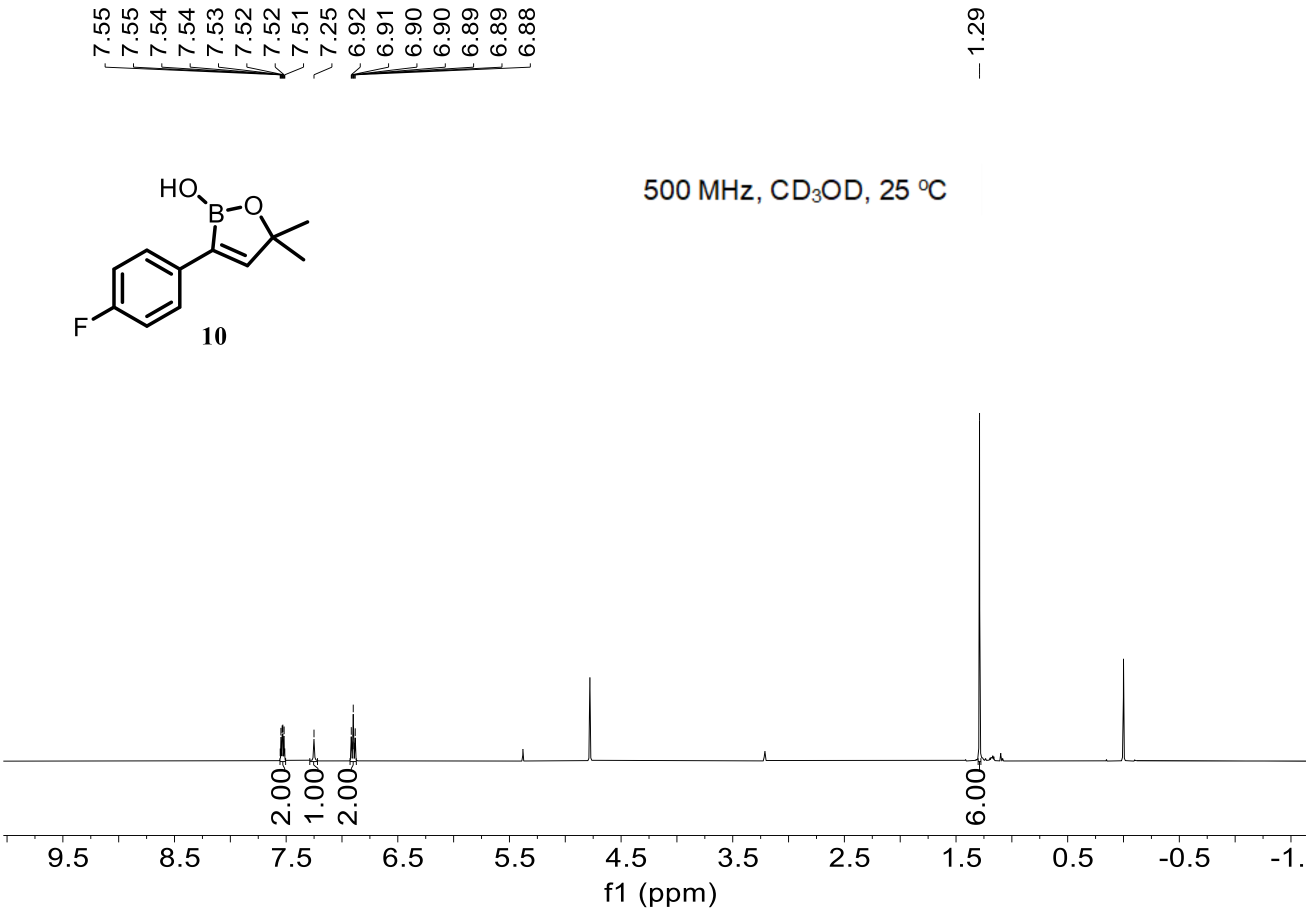

7.55 7.55 7.54 7.54 7.53 7.52 7.52 7.51 7.25 6.92 6.91 6.90 6.90 6.89 6.89 6.88
1.29
500 MHz, CD3OD, 25 °C
HO
B
O
F
10
2.00 1.00 2.00
6.00
9.5 8.5 7.5 6.5 5.5 4.5 3.5 2.5 1.5 0.5 -0.5
f1 (ppm)

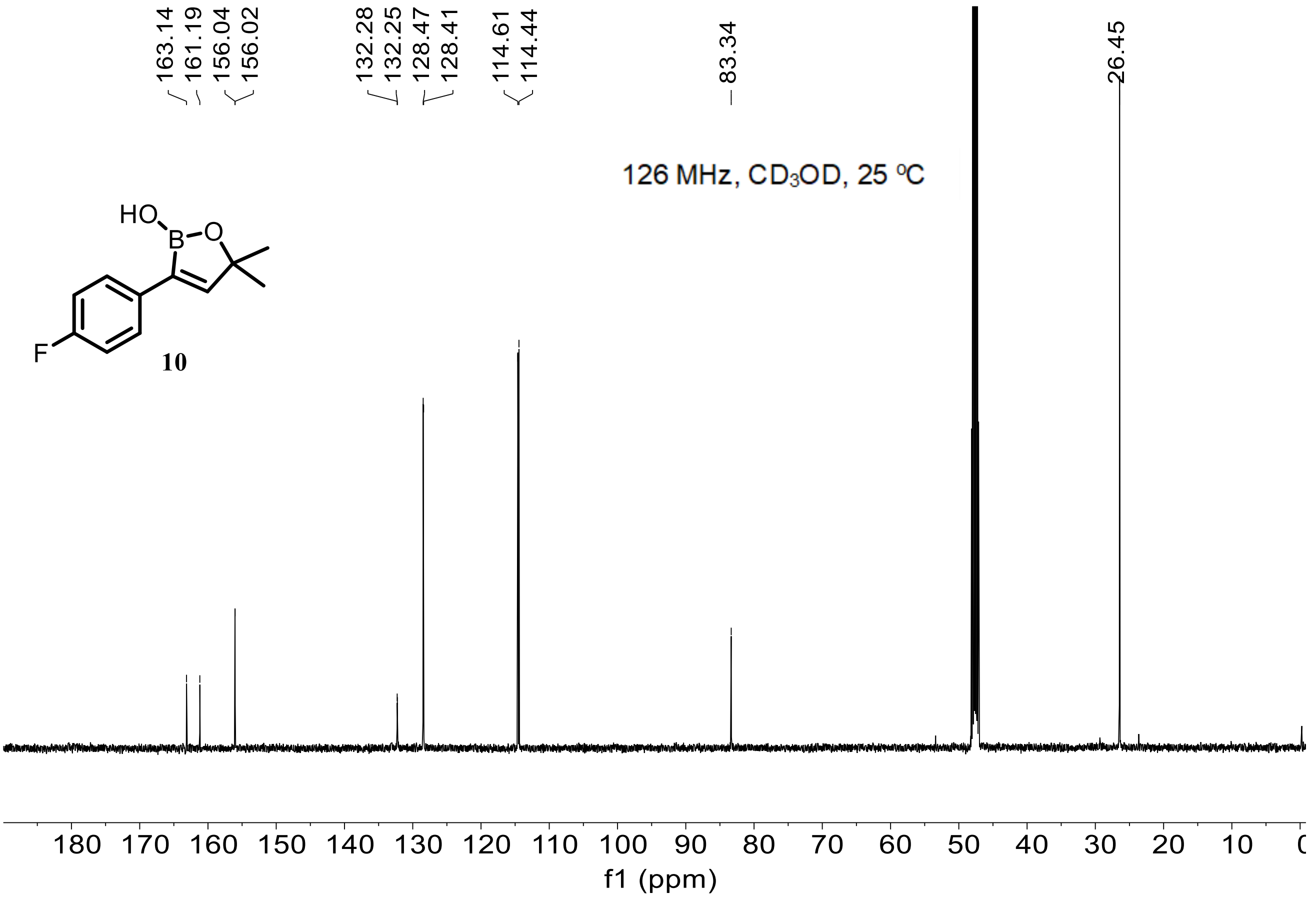

163.14 161.19 156.04 156.02
132.28 132.25 128.47 128.41 114.61 114.44
83.34
26.45
126 MHz, CD3OD, 25 °C
HO
B
O
F
10
180 170 160 150 140 130 120 110 100 90 80 70 60 50 40 30 20 10
f1 (ppm)

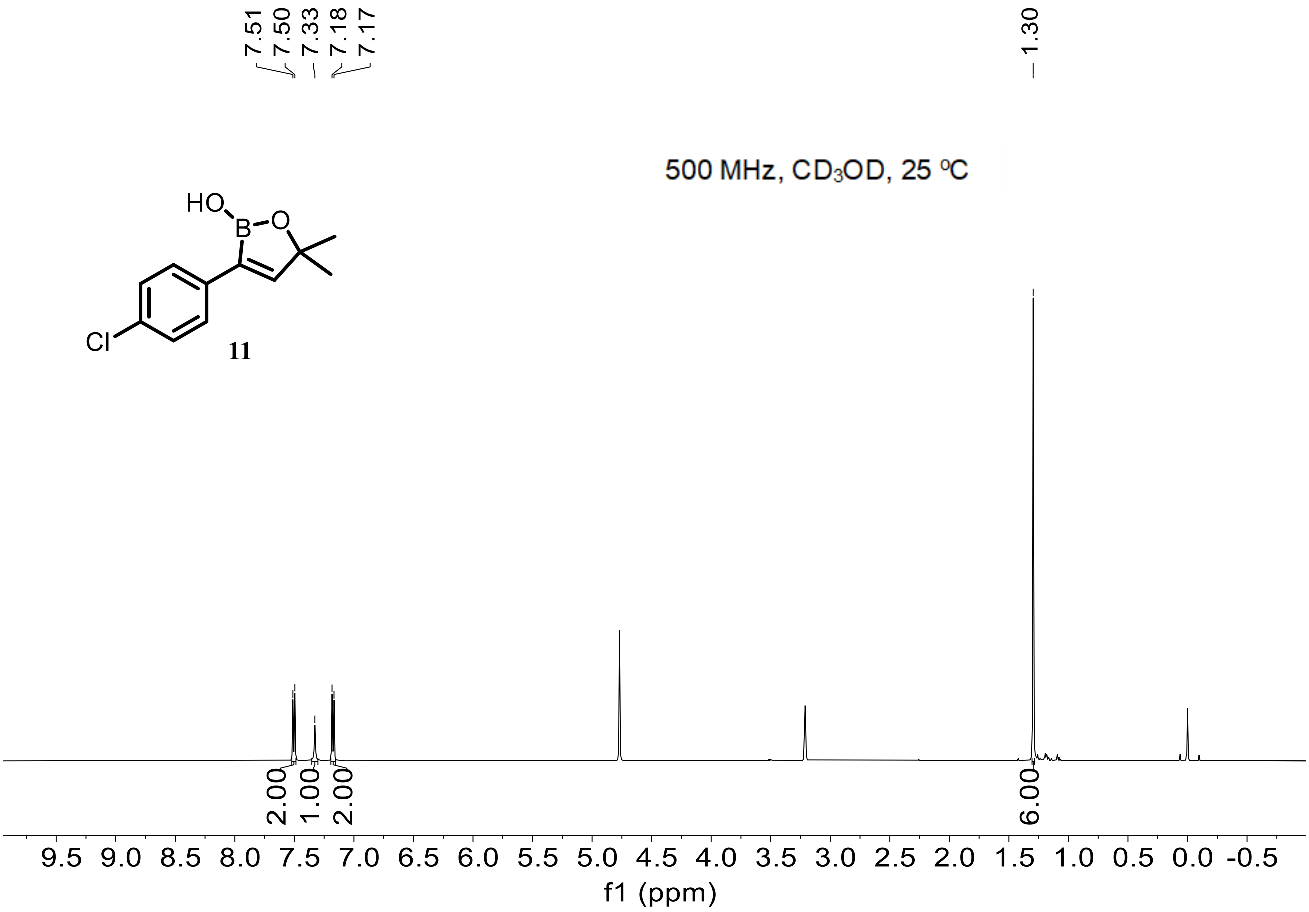
7.51
7.50
7.33
7.18
7.17
1.30
500 MHz, CD3OD, 25 °C
HO
B
O
Cl
11
2.00
1.00
2.00
6.00
9.5 9.0 8.5 8.0 7.5 7.0 6.5 6.0 5.5 5.0 4.5 4.0 3.5 3.0 2.5 2.0 1.5 1.0 0.5 0.0 -0.5
f1 (ppm)

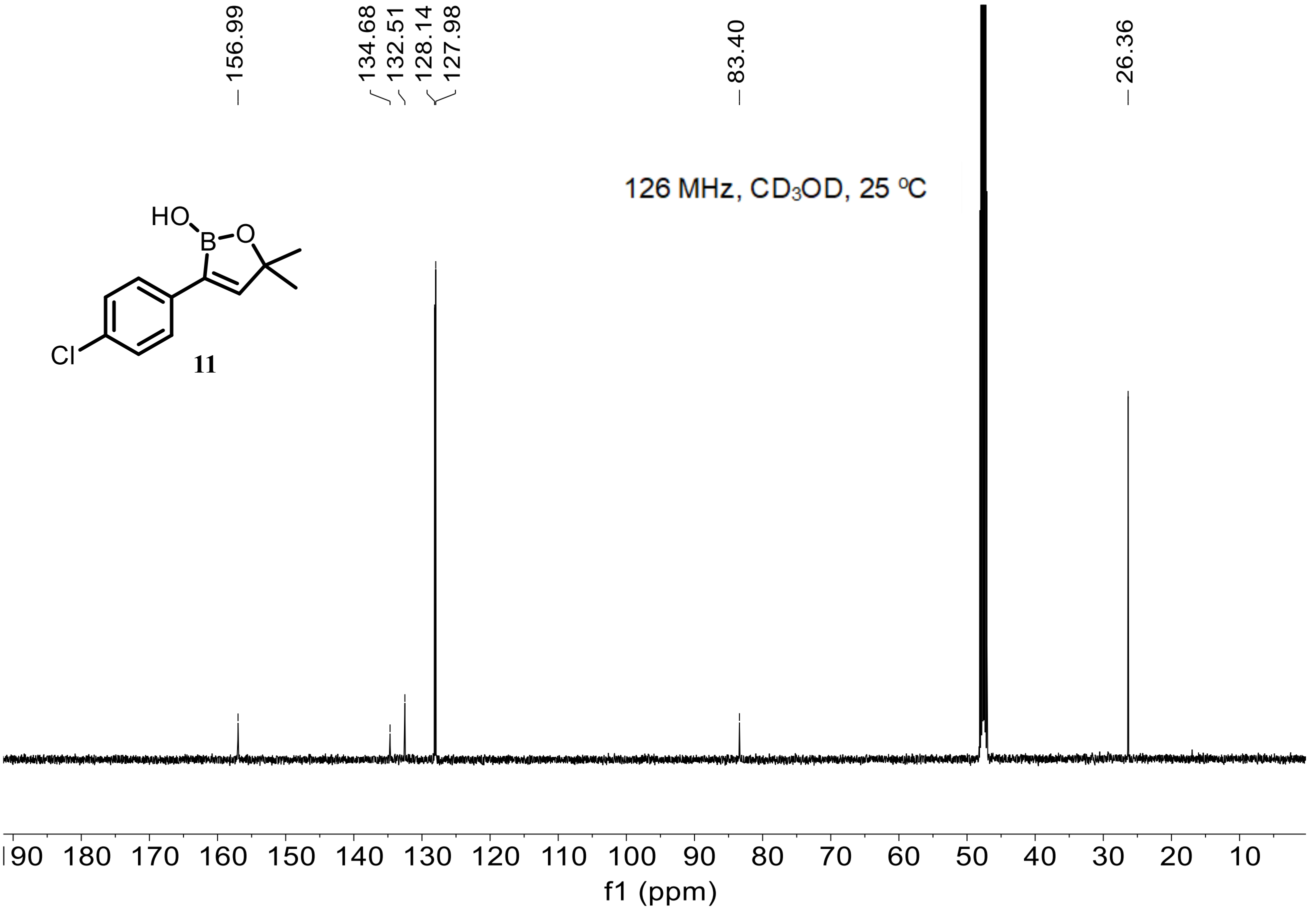
156.99
134.68
132.51
128.14
127.98
83.40
26.36
126 MHz, CD3OD, 25 °C
HO
B
O
Cl
11
190 180 170 160 150 140 130 120 110 100 90 80 70 60 50 40 30 20 10
f1 (ppm)

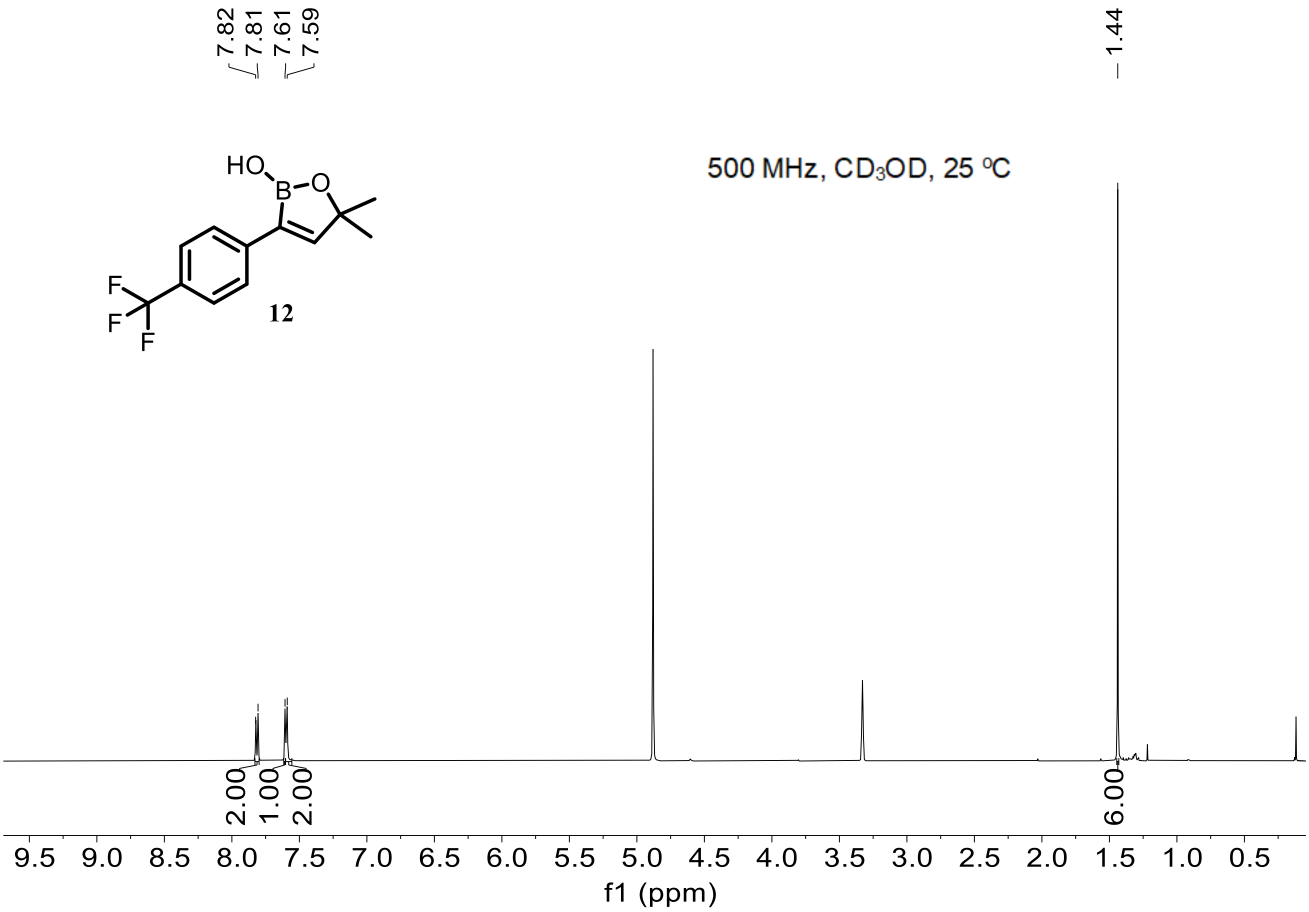
7.82
7.81
7.61
7.59
1.44
500 MHz, CD3OD, 25 °C
HO
B
O
F
F
F
12
2.00
1.00
2.00
6.00
9.5 9.0 8.5 8.0 7.5 7.0 6.5 6.0 5.5 5.0 4.5 4.0 3.5 3.0 2.5 2.0 1.5 1.0 0.5
f1 (ppm)

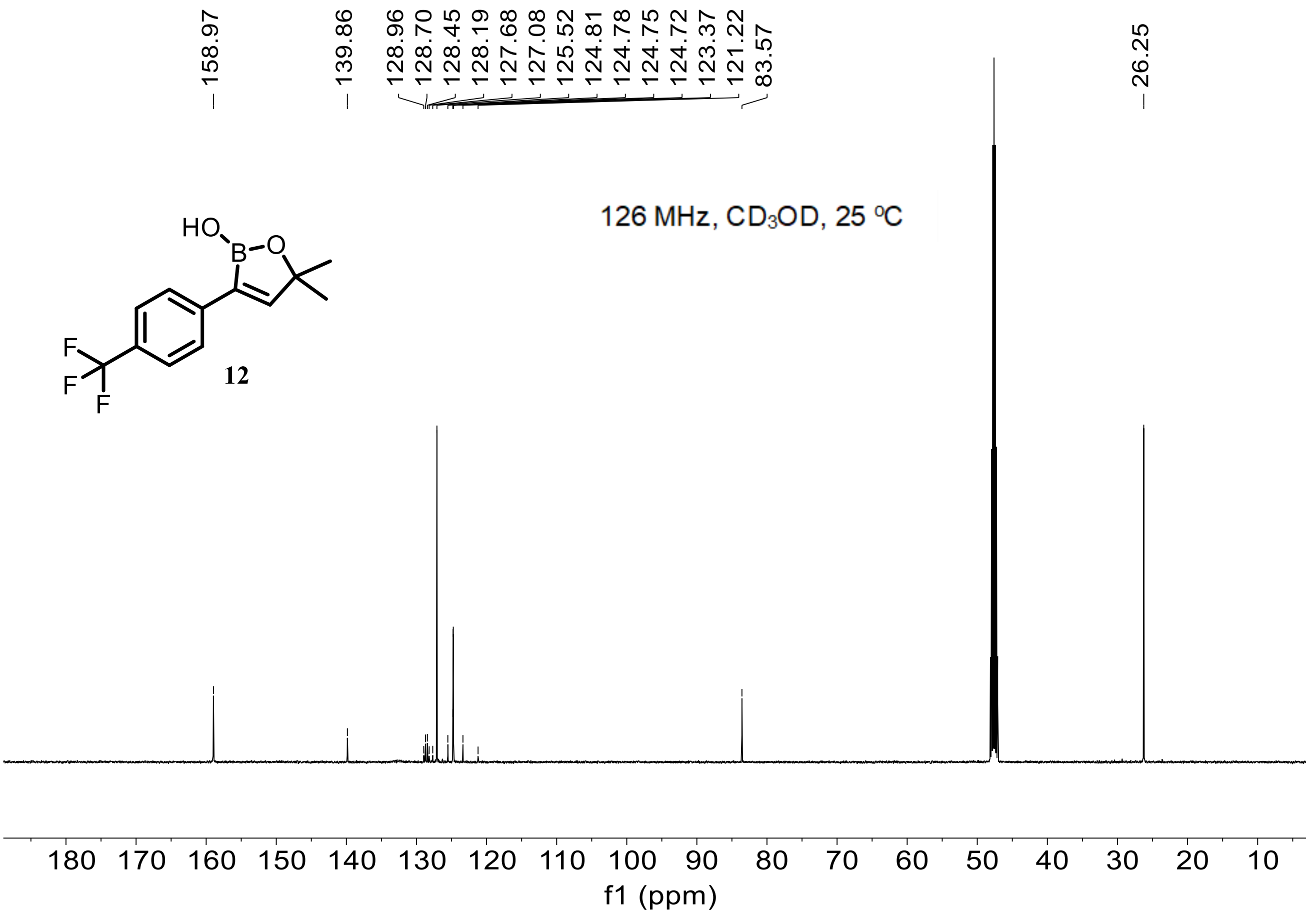
158.97
139.86
128.96
128.70
128.45
128.19
127.68
127.08
125.52
124.81
124.78
124.75
124.72
123.37
121.22
83.57
26.25
126 MHz, CD3OD, 25 °C
HO
B
O
F
F
F
12
180 170 160 150 140 130 120 110 100 90 80 70 60 50 40 30 20 10
f1 (ppm)

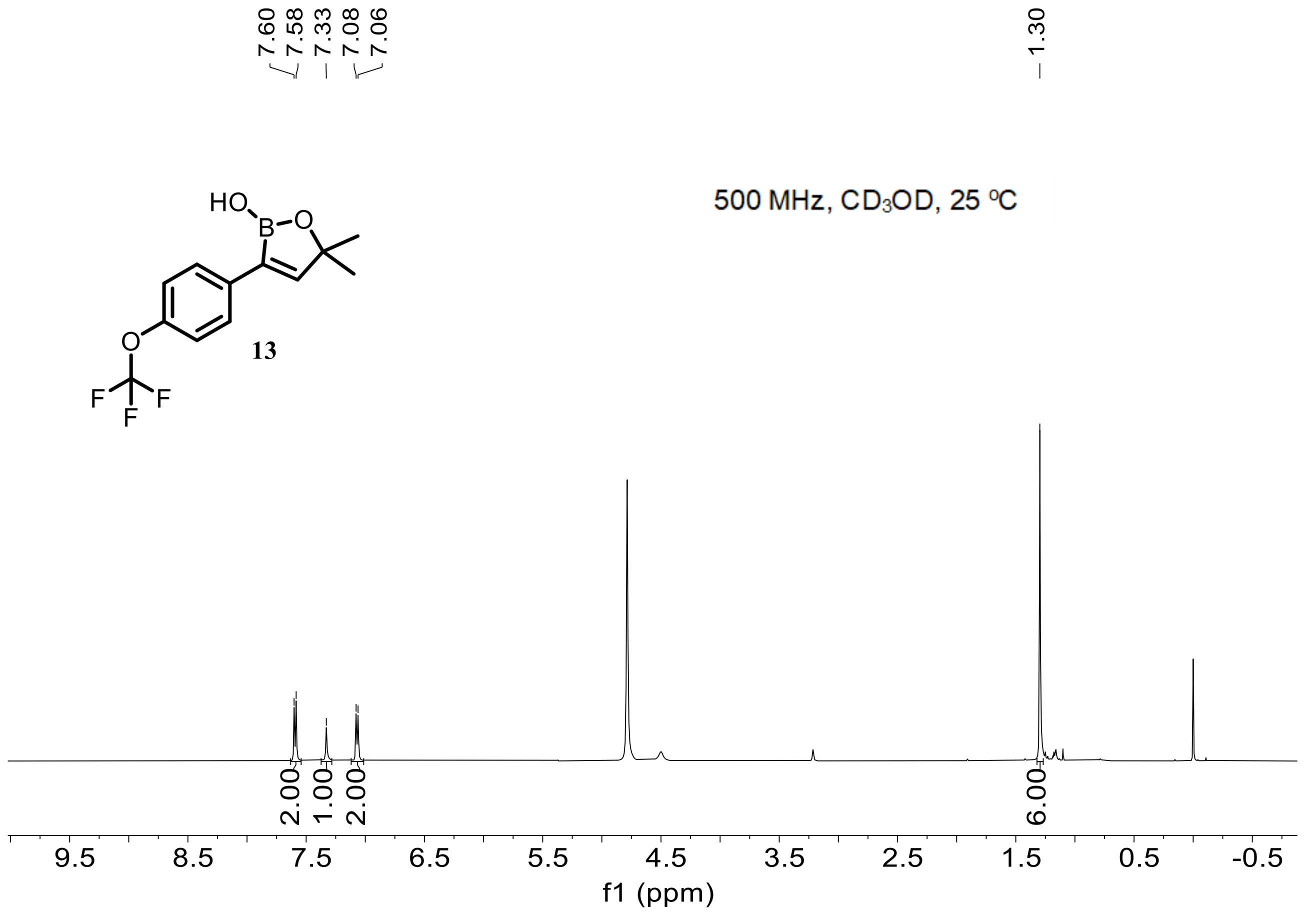
7.60
7.58
7.33
7.08
7.06
1.30
500 MHz, CD3OD, 25 °C
HO
B
O
O
F
F
F
13
2.00
1.00
2.00
6.00
9.5
8.5
7.5
6.5
5.5
4.5
3.5
2.5
1.5
0.5
-0.5
f1 (ppm)

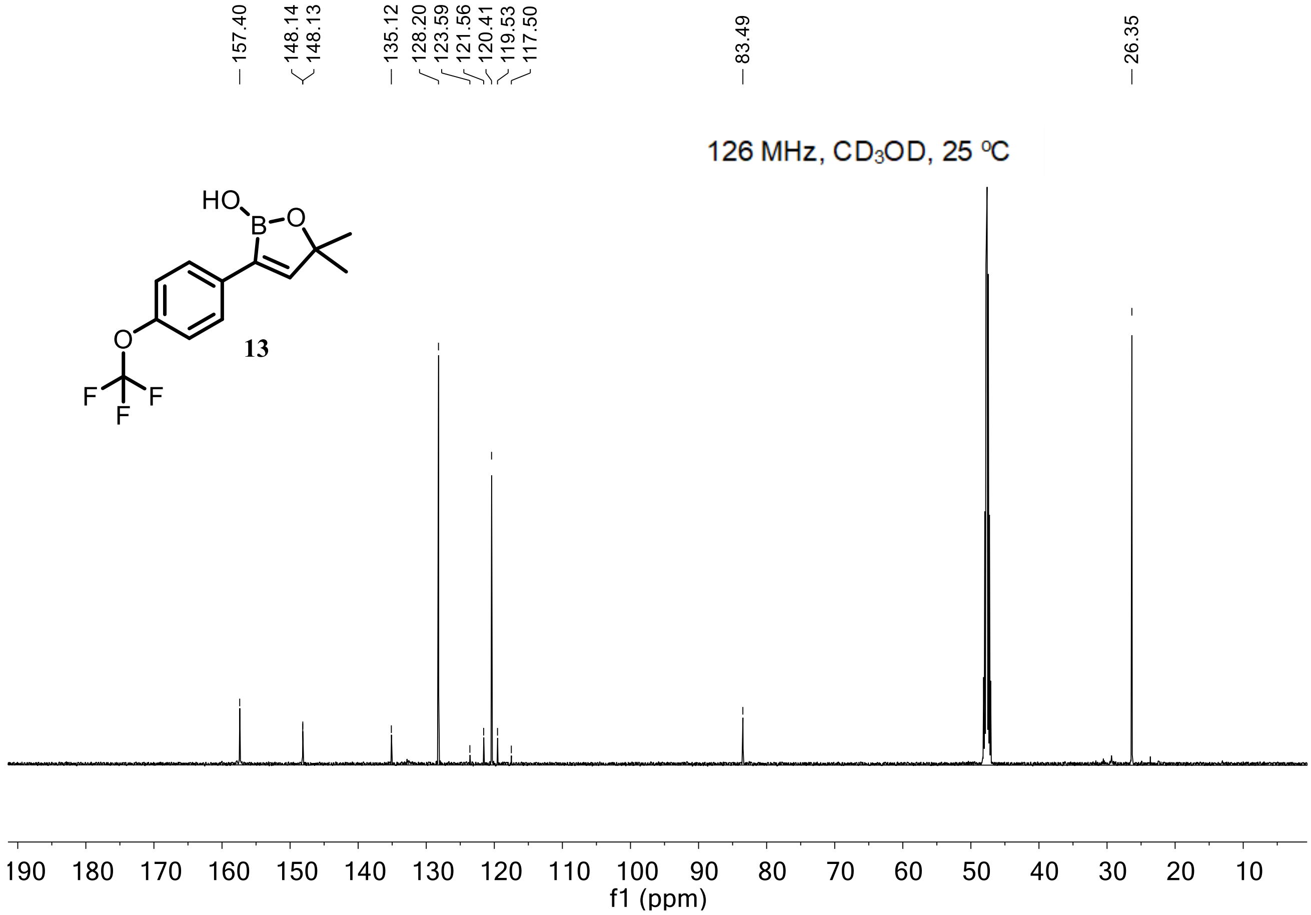
157.40
148.14
148.13
135.12
128.20
123.59
121.56
120.41
119.53
117.50
83.49
26.35
126 MHz, CD3OD, 25 °C
HO
B
O
O
F
F
F
13
190
180
170
160
150
140
130
120
110
100
90
80
70
60
50
40
30
20
10
f1 (ppm)

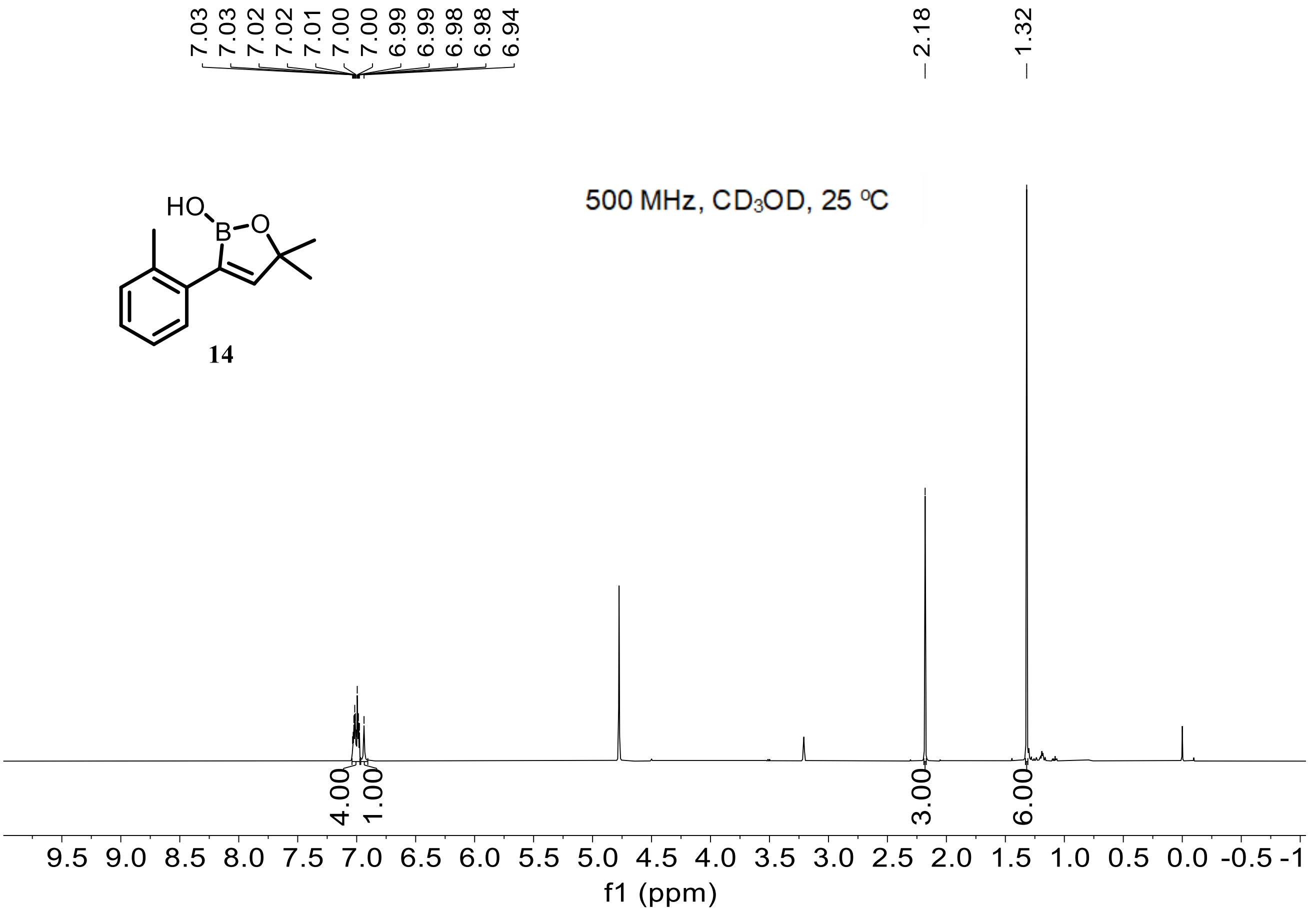
7.03
7.03
7.02
7.02
7.01
7.00
7.00
6.99
6.99
6.98
6.98
6.94
2.18
1.32
HO
B
O
14
500 MHz, CD3OD, 25 °C
4.00
1.00
3.00
6.00
9.5 9.0 8.5 8.0 7.5 7.0 6.5 6.0 5.5 5.0 4.5 4.0 3.5 3.0 2.5 2.0 1.5 1.0 0.5 0.0 -0.5 -1
f1 (ppm)

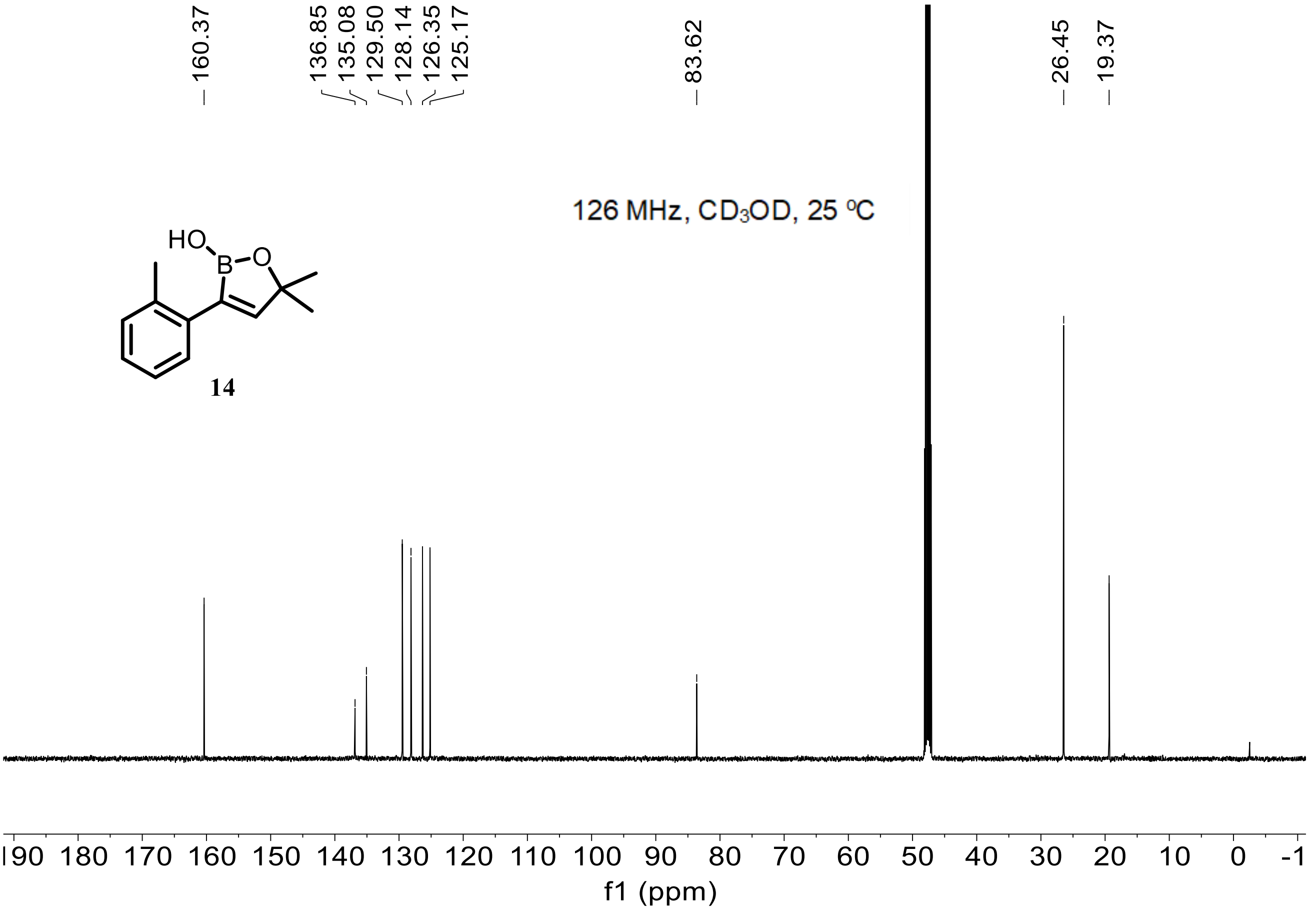
160.37
136.85
135.08
129.50
128.14
126.35
125.17
83.62
26.45
19.37
HO
B
O
14
126 MHz, CD3OD, 25 °C
190 180 170 160 150 140 130 120 110 100 90 80 70 60 50 40 30 20 10 0 -1
f1 (ppm)

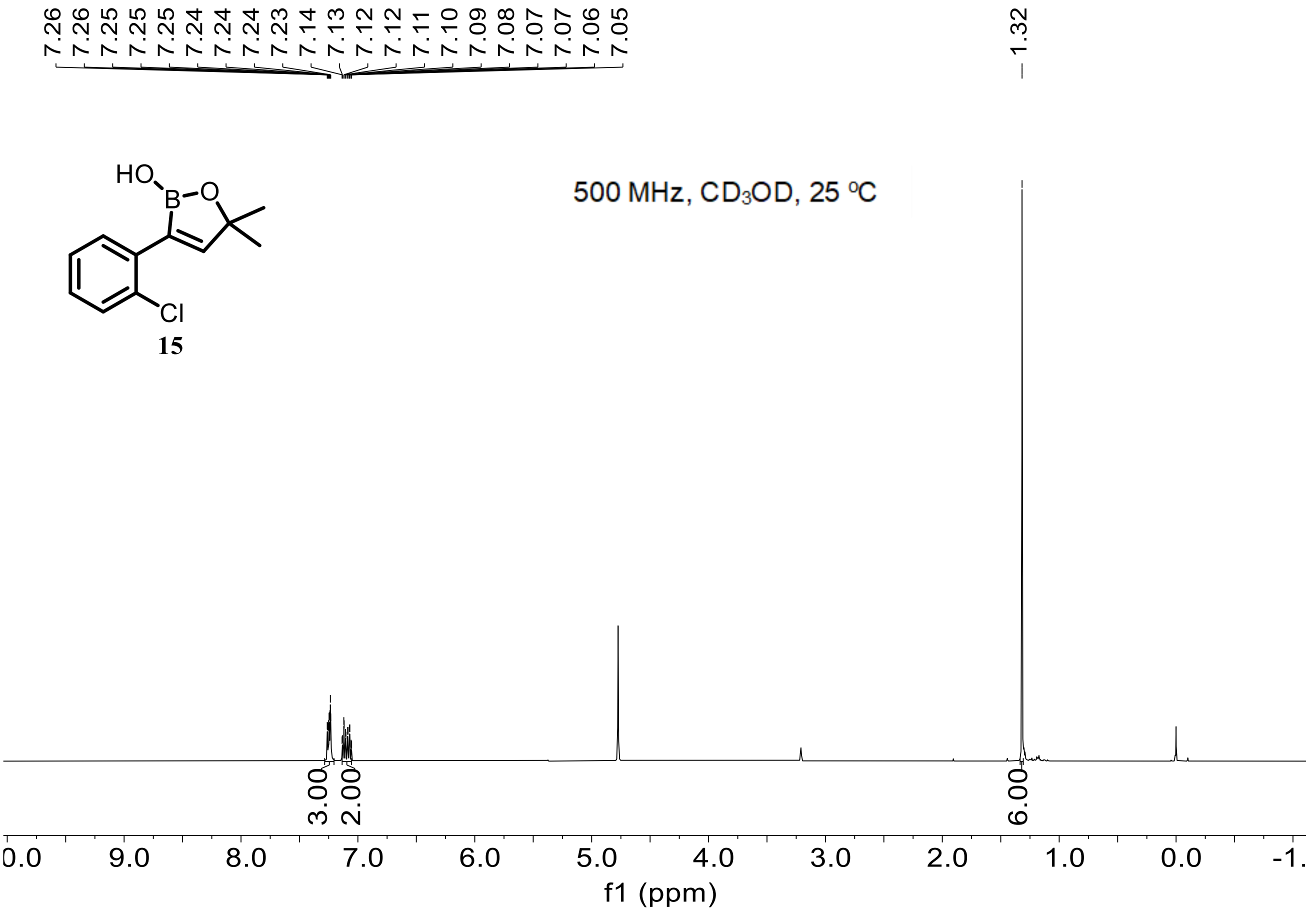
7.26
7.26
7.25
7.25
7.25
7.24
7.24
7.24
7.23
7.14
7.13
7.12
7.12
7.11
7.10
7.09
7.08
7.07
7.07
7.06
7.05
1.32
HO
B
O
Cl
15
500 MHz, CD3OD, 25 °C
3.00
2.00
6.00
0.0
9.0
8.0
7.0
6.0
5.0
4.0
3.0
2.0
1.0
0.0
-1.
f1 (ppm)

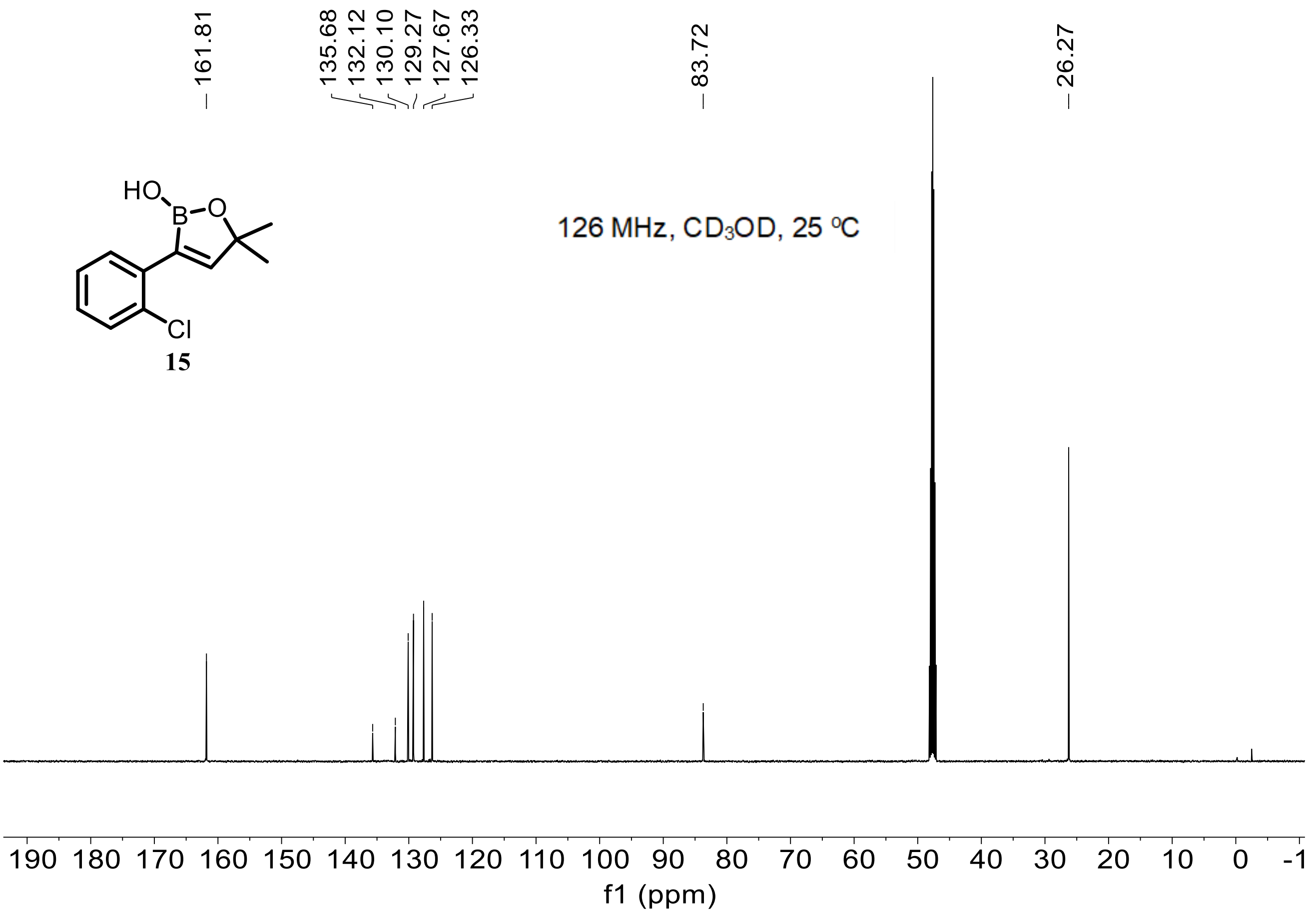
161.81
135.68
132.12
130.10
129.27
127.67
126.33
83.72
26.27
HO
B
O
Cl
15
126 MHz, CD3OD, 25 °C
190
180
170
160
150
140
130
120
110
100
90
80
70
60
50
40
30
20
10
0
-1
f1 (ppm)

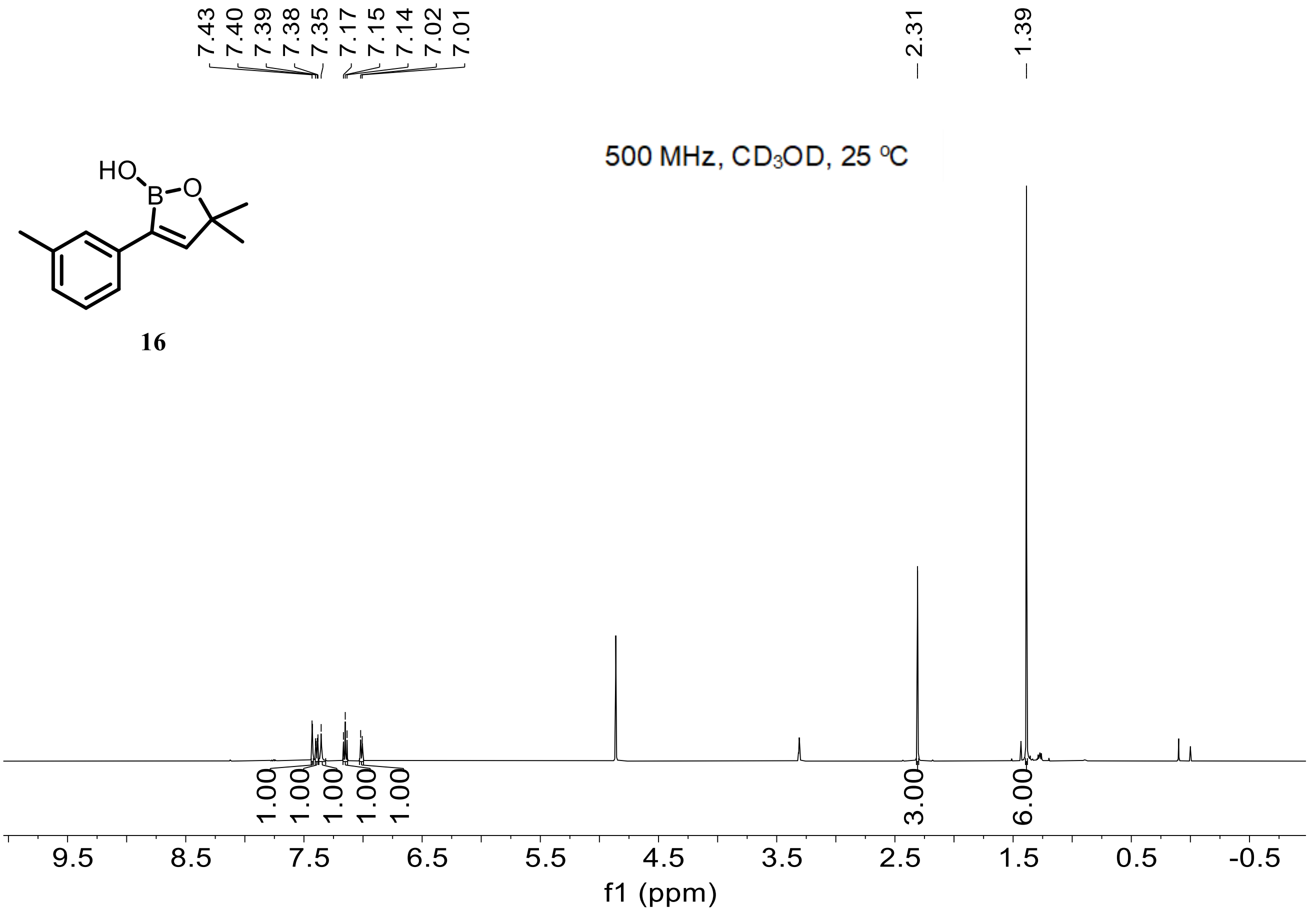
7.43
7.40
7.39
7.38
7.35
7.17
7.15
7.14
7.02
7.01
2.31
1.39
500 MHz, CD3OD, 25 °C
HO
B
O
16
1.00
1.00
1.00
1.00
1.00
3.00
6.00
9.5
8.5
7.5
6.5
5.5
4.5
3.5
2.5
1.5
0.5
-0.5
f1 (ppm)

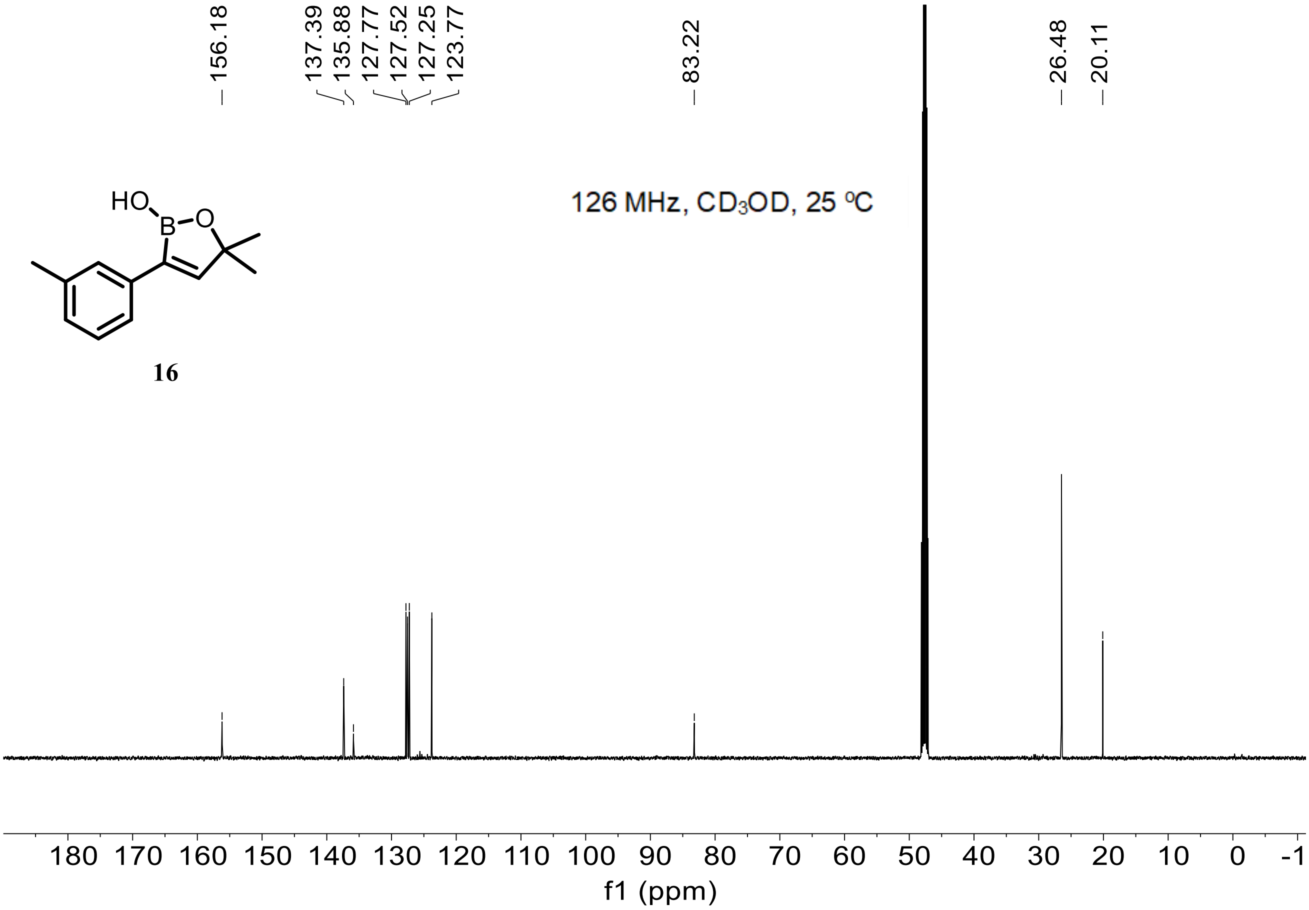
156.18
137.39
135.88
127.77
127.52
127.25
123.77
83.22
26.48
20.11
126 MHz, CD3OD, 25 °C
HO
B
O
16
180 170 160 150 140 130 120 110 100 90 80 70 60 50 40 30 20 10 0 -1
f1 (ppm)

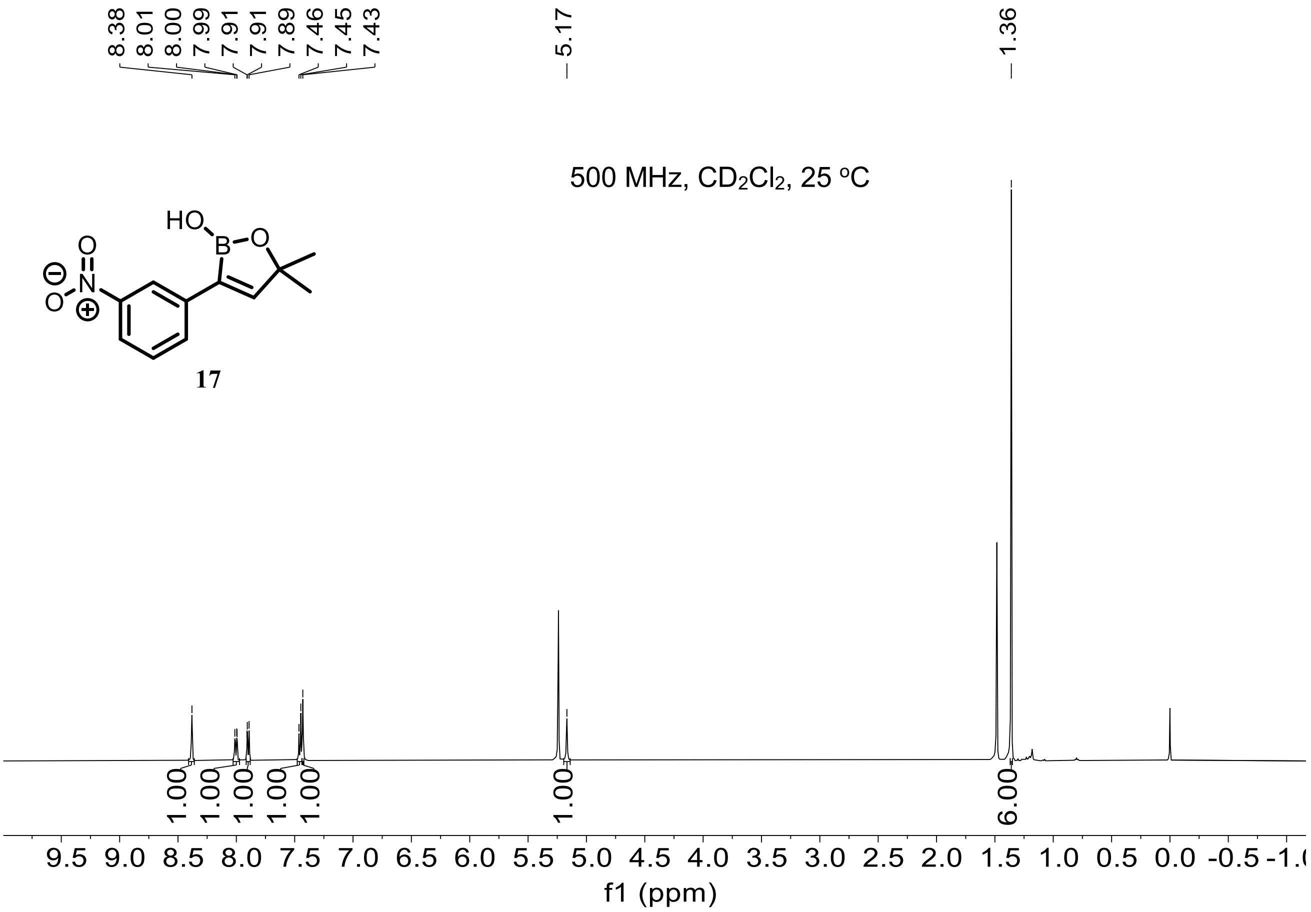
8.38 8.01 8.00 7.99 7.91 7.91 7.89 7.46 7.45 7.43
5.17
1.36
500 MHz, CD2Cl2, 25 °C
HO B O
O N O
17
1.00 1.00 1.00 1.00 1.00
1.00
6.00
9.5 9.0 8.5 8.0 7.5 7.0 6.5 6.0 5.5 5.0 4.5 4.0 3.5 3.0 2.5 2.0 1.5 1.0 0.5 0.0 -0.5 -1.0
f1 (ppm)

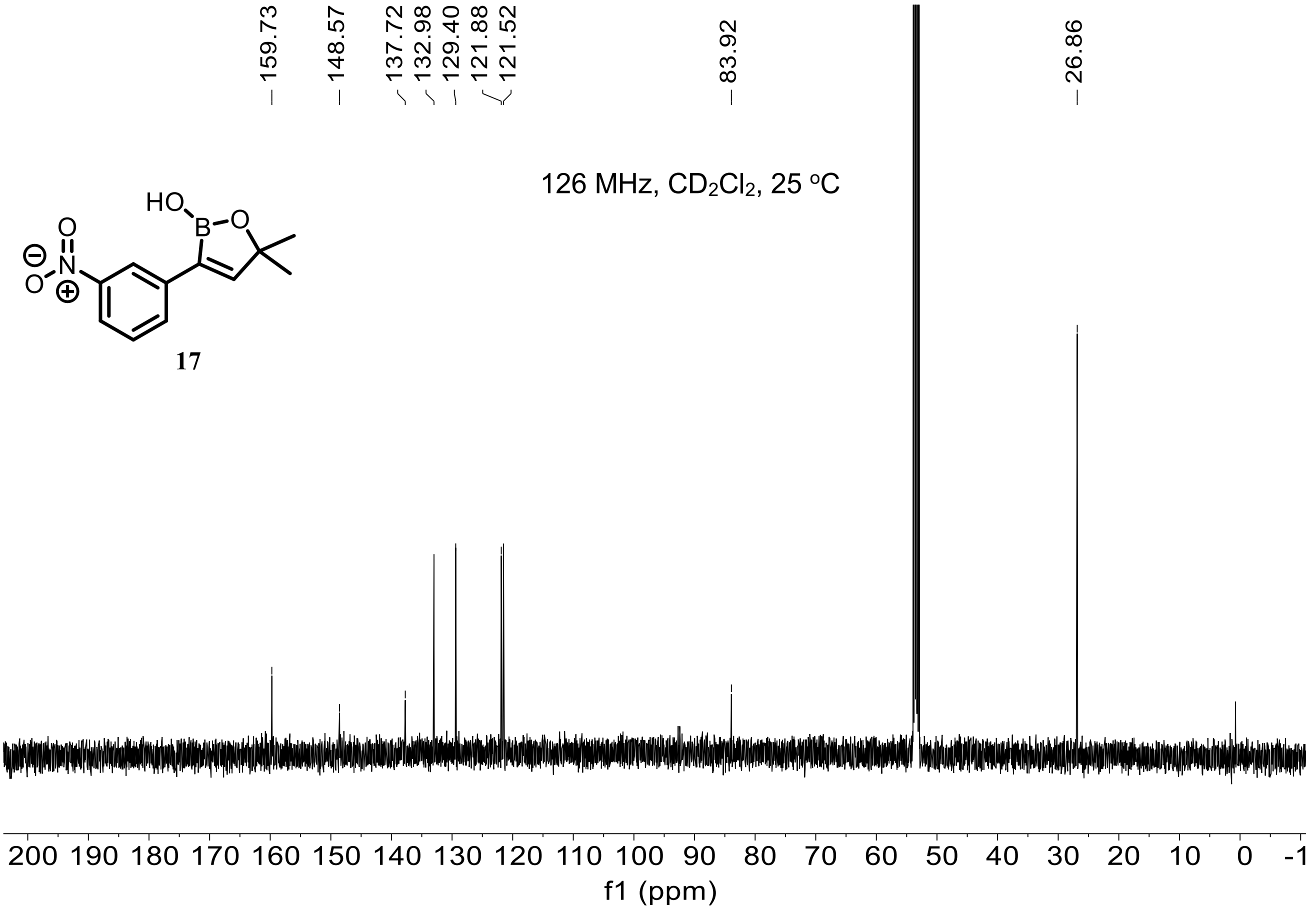
159.73
148.57
137.72 132.98 129.40 121.88 121.52
83.92
26.86
126 MHz, CD2Cl2, 25 °C
HO B O
O N O
17
200 190 180 170 160 150 140 130 120 110 100 90 80 70 60 50 40 30 20 10 0 -1
f1 (ppm)

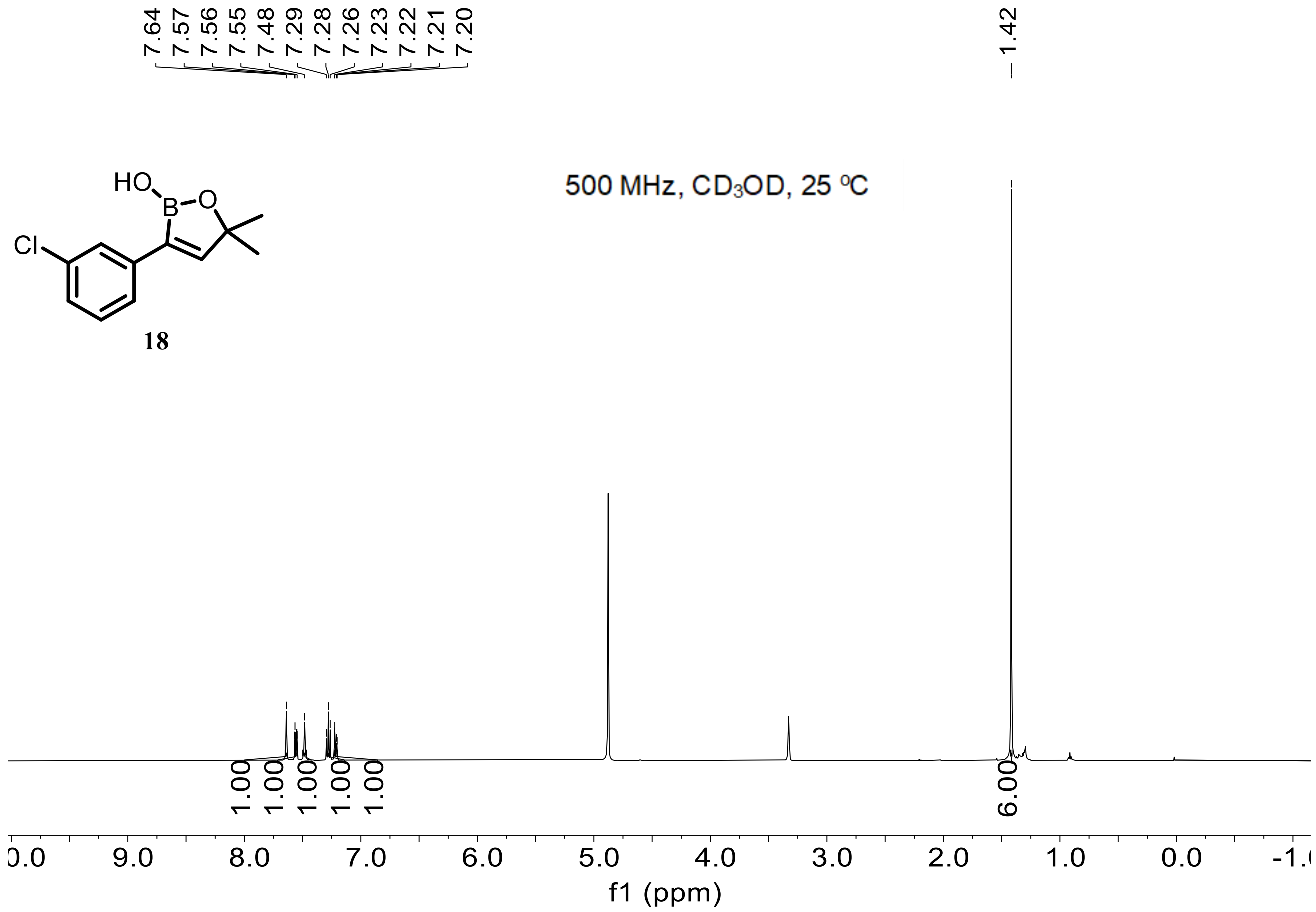
7.64
7.57
7.56
7.55
7.48
7.29
7.28
7.26
7.23
7.22
7.21
7.20
1.42
500 MHz, CD3OD, 25 °C
HO
B
O
Cl
18
1.00
1.00
1.00
1.00
1.00
6.00
9.0
8.0
7.0
6.0
5.0
4.0
3.0
2.0
1.0
0.0
f1 (ppm)

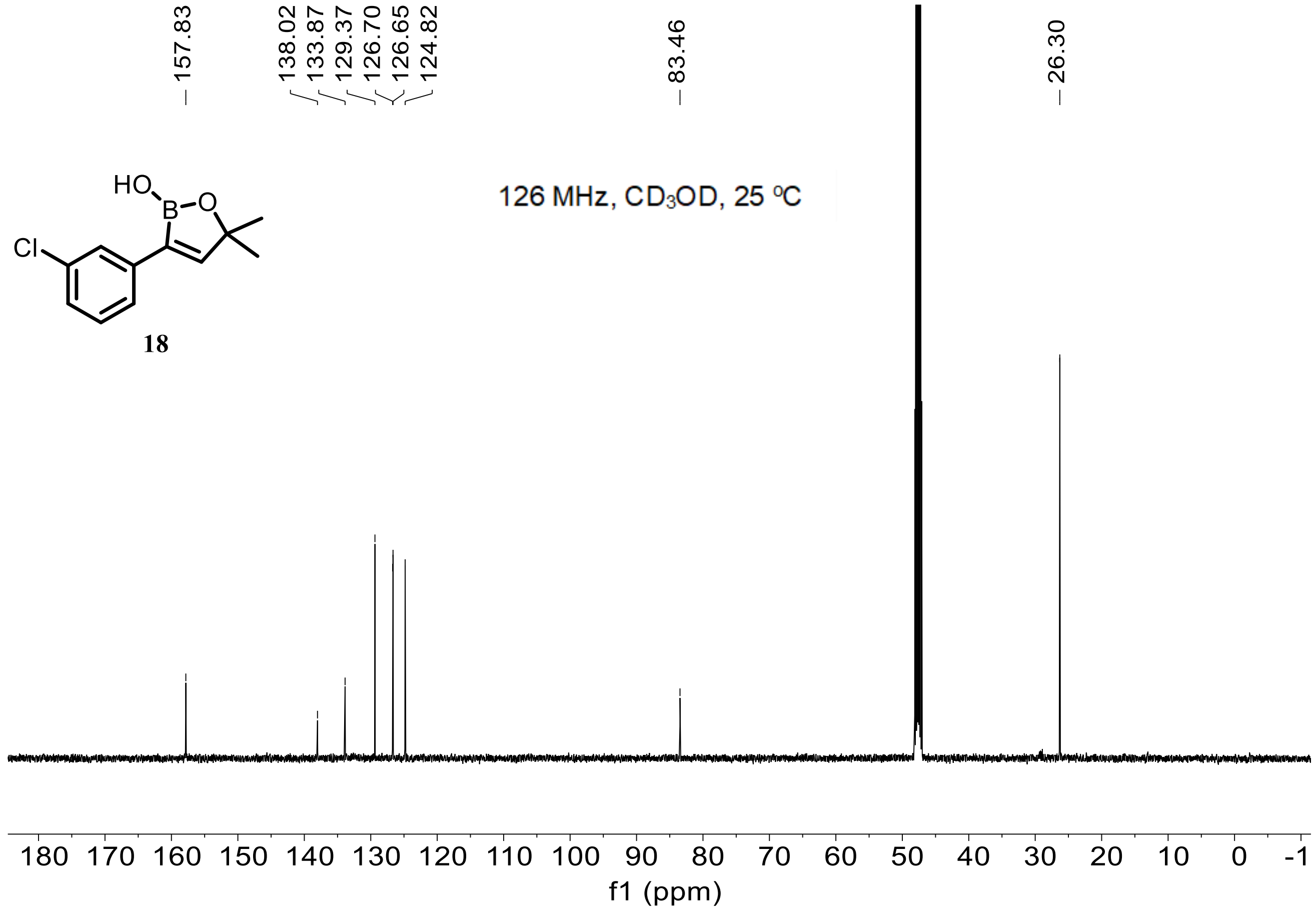
157.83
138.02
133.87
129.37
126.70
126.65
124.82
83.46
26.30
126 MHz, CD3OD, 25 °C
HO
B
O
Cl
18
180
170
160
150
140
130
120
110
100
90
80
70
60
50
40
30
20
10
0
-1
f1 (ppm)

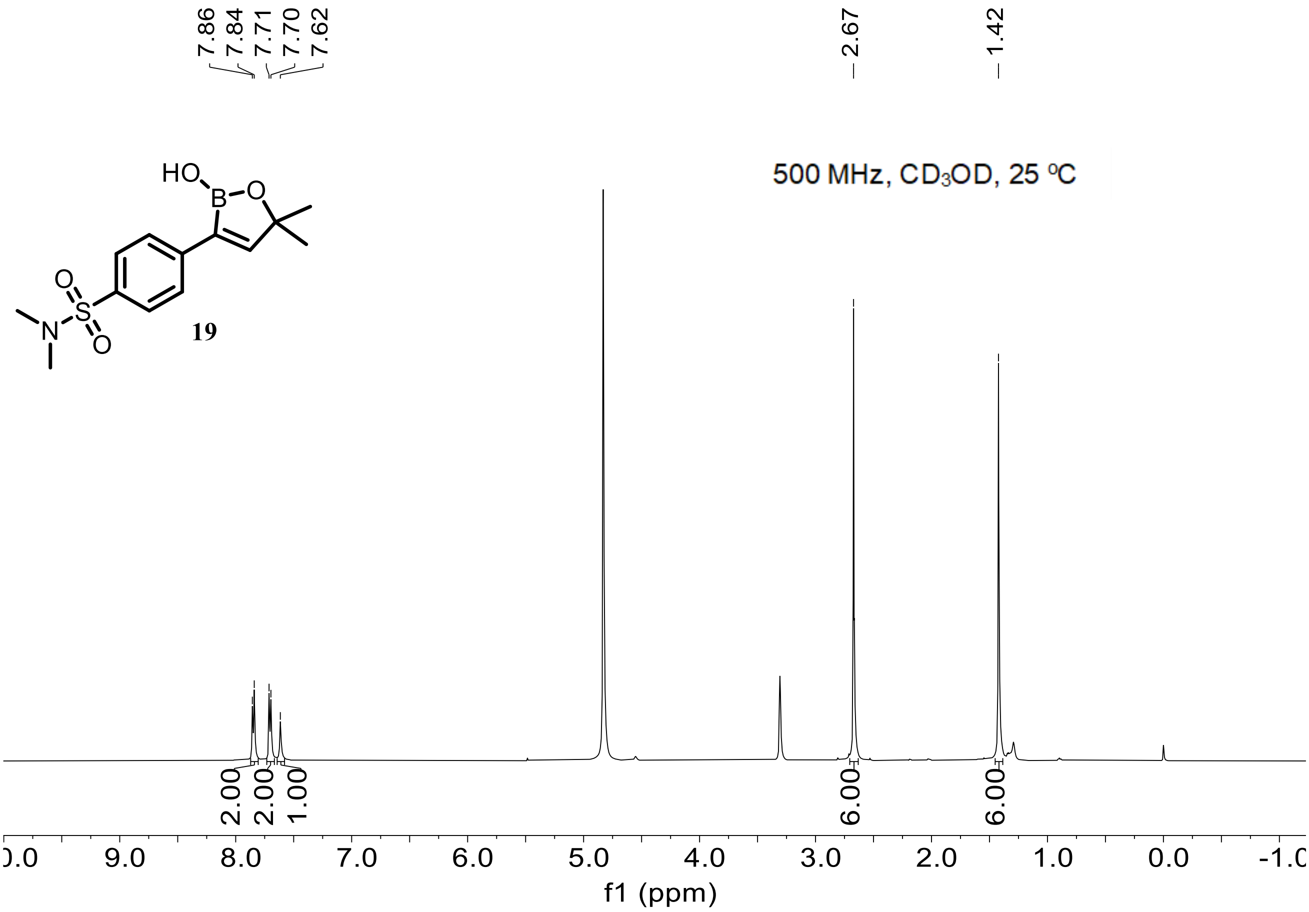
7.86
7.84
7.71
7.70
7.62
2.67
1.42
500 MHz, CD3OD, 25 ºC
HO
B
O
O
S
O
N
19
2.00
2.00
1.00
6.00
6.00
f1 (ppm)

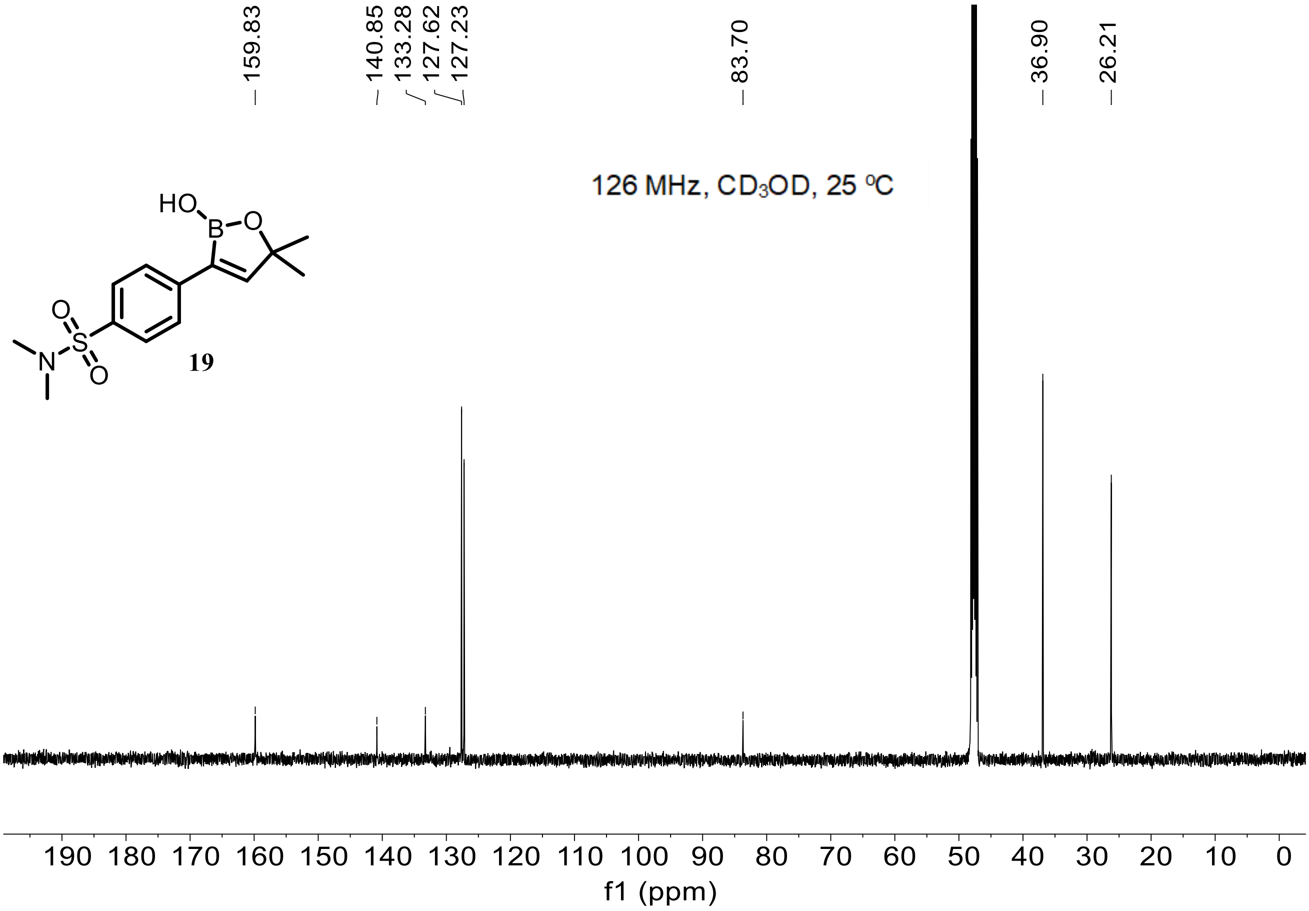
159.83
140.85
133.28
127.62
127.23
83.70
36.90
26.21
126 MHz, CD3OD, 25 ºC
HO
B
O
O
S
O
N
19
f1 (ppm)

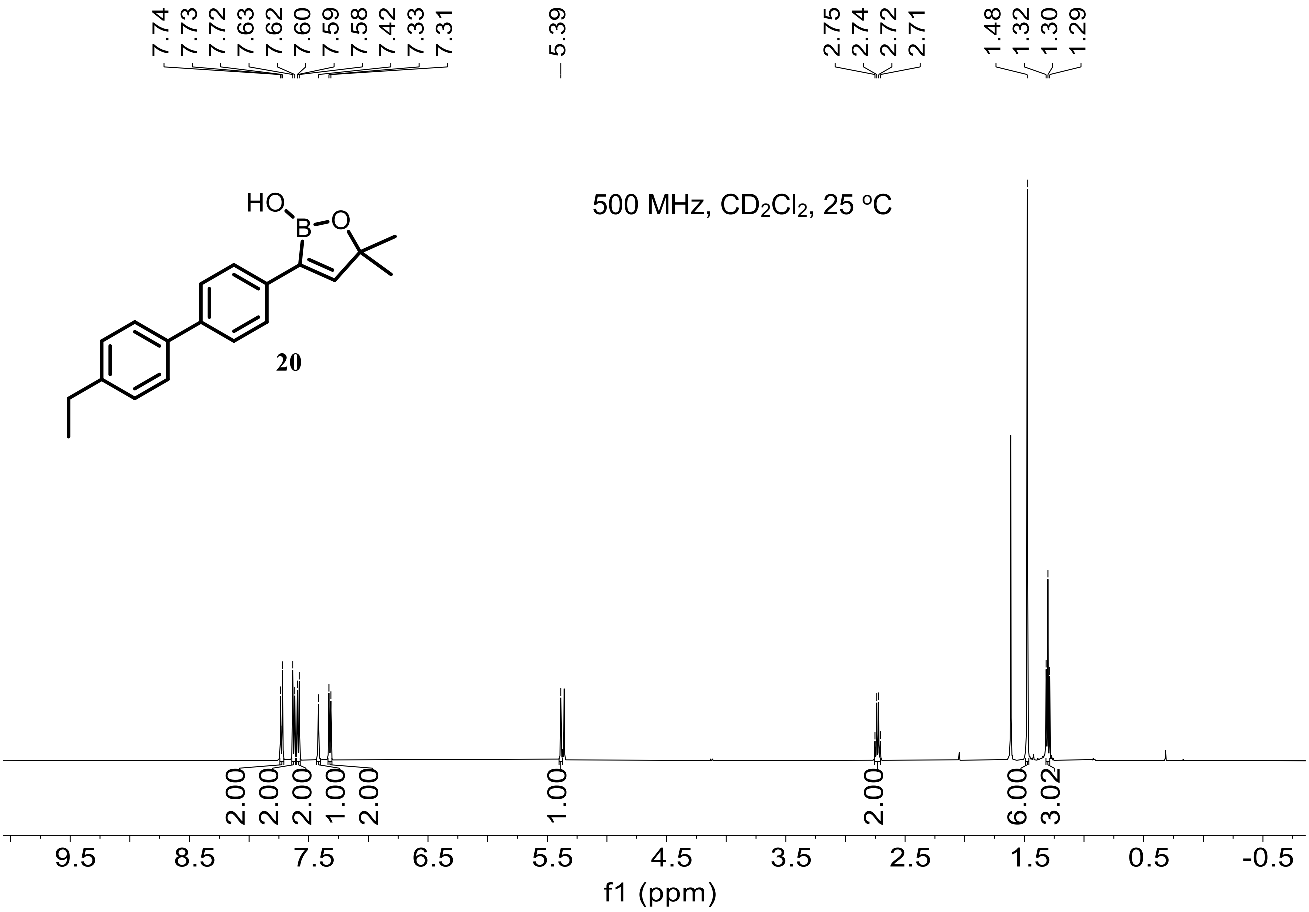
7.74 7.73 7.72 7.63 7.62 7.60 7.59 7.58 7.42 7.33 7.31
5.39
2.75 2.74 2.72 2.71
1.48 1.32 1.30 1.29
500 MHz, CD_2Cl_2, 25 °C
HO B O
20
2.00 2.00 2.00 1.00 2.00
1.00
2.00
6.00 3.02
9.5 8.5 7.5 6.5 5.5 4.5 3.5 2.5 1.5 0.5 -0.5
f1 (ppm)

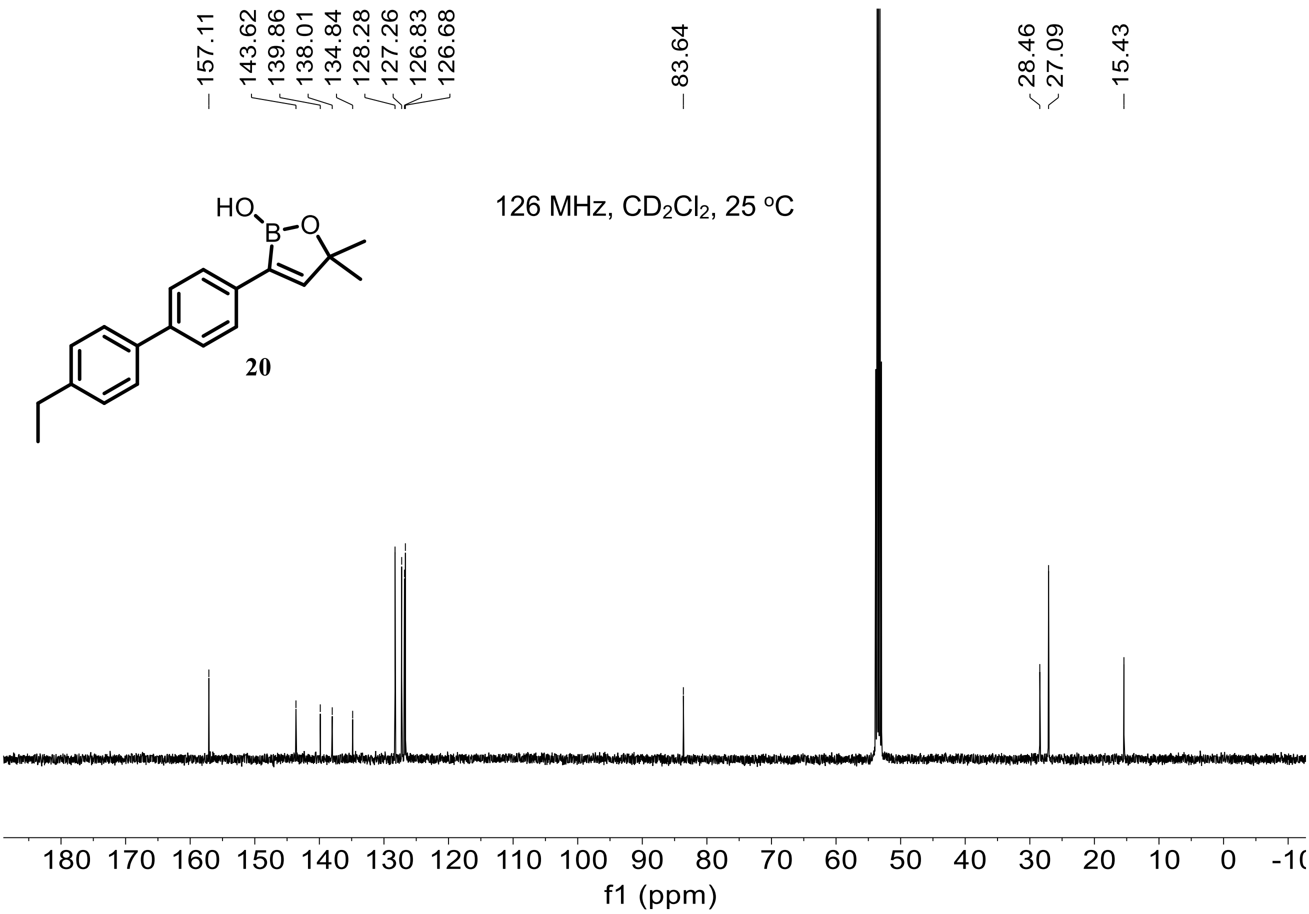
157.11
143.62 139.86 138.01 134.84 128.28 127.26 126.83 126.68
83.64
28.46 27.09
15.43
126 MHz, CD_2Cl_2, 25 °C
HO B O
20
180 170 160 150 140 130 120 110 100 90 80 70 60 50 40 30 20 10 0 -1(
f1 (ppm)

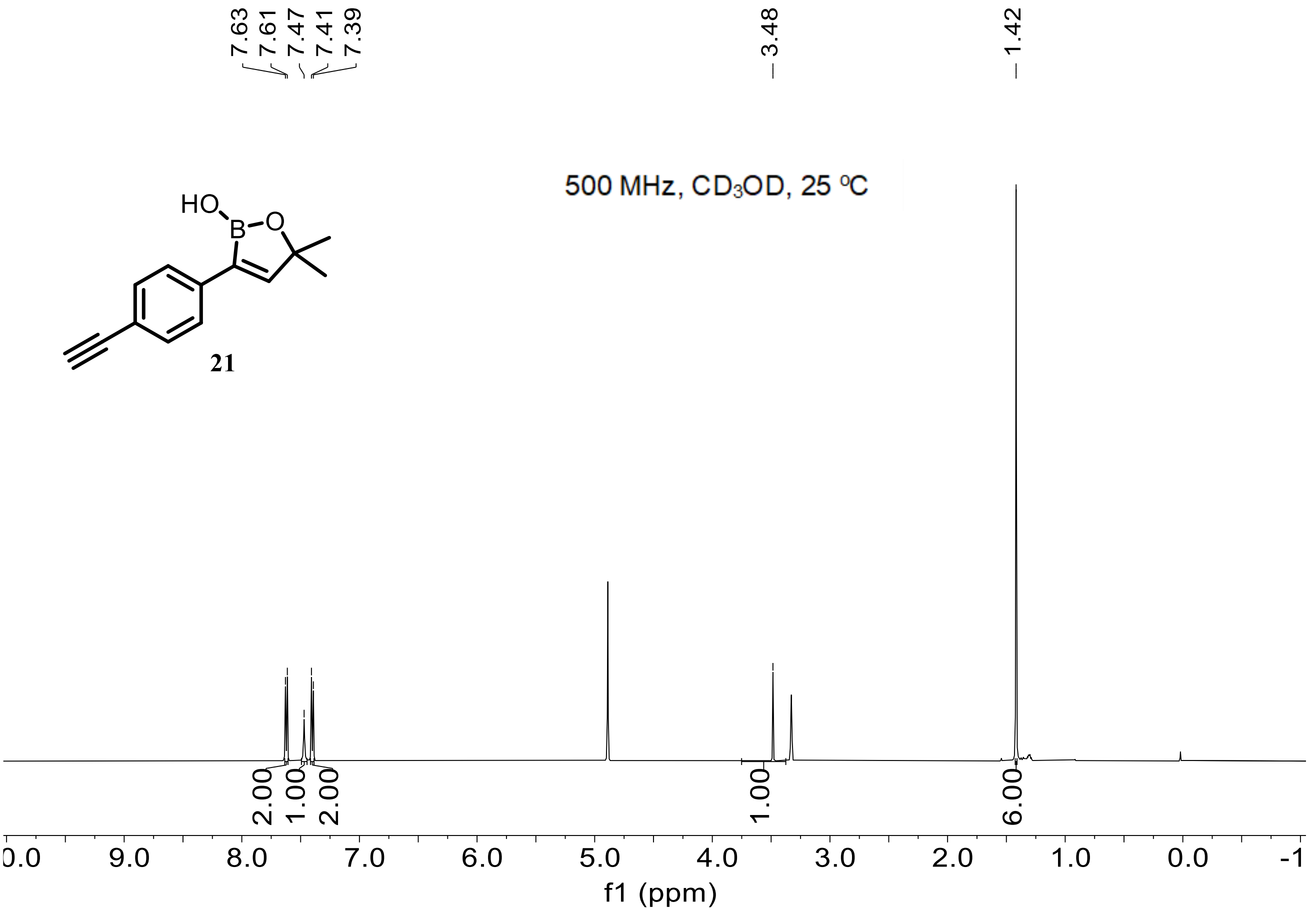
7.63
7.61
7.47
7.41
7.39
3.48
1.42
500 MHz, CD_3OD, 25 °C
HO
B
O
21
2.00
1.00
2.00
1.00
6.00
0.0 9.0 8.0 7.0 6.0 5.0 4.0 3.0 2.0 1.0 0.0 -1
f1 (ppm)

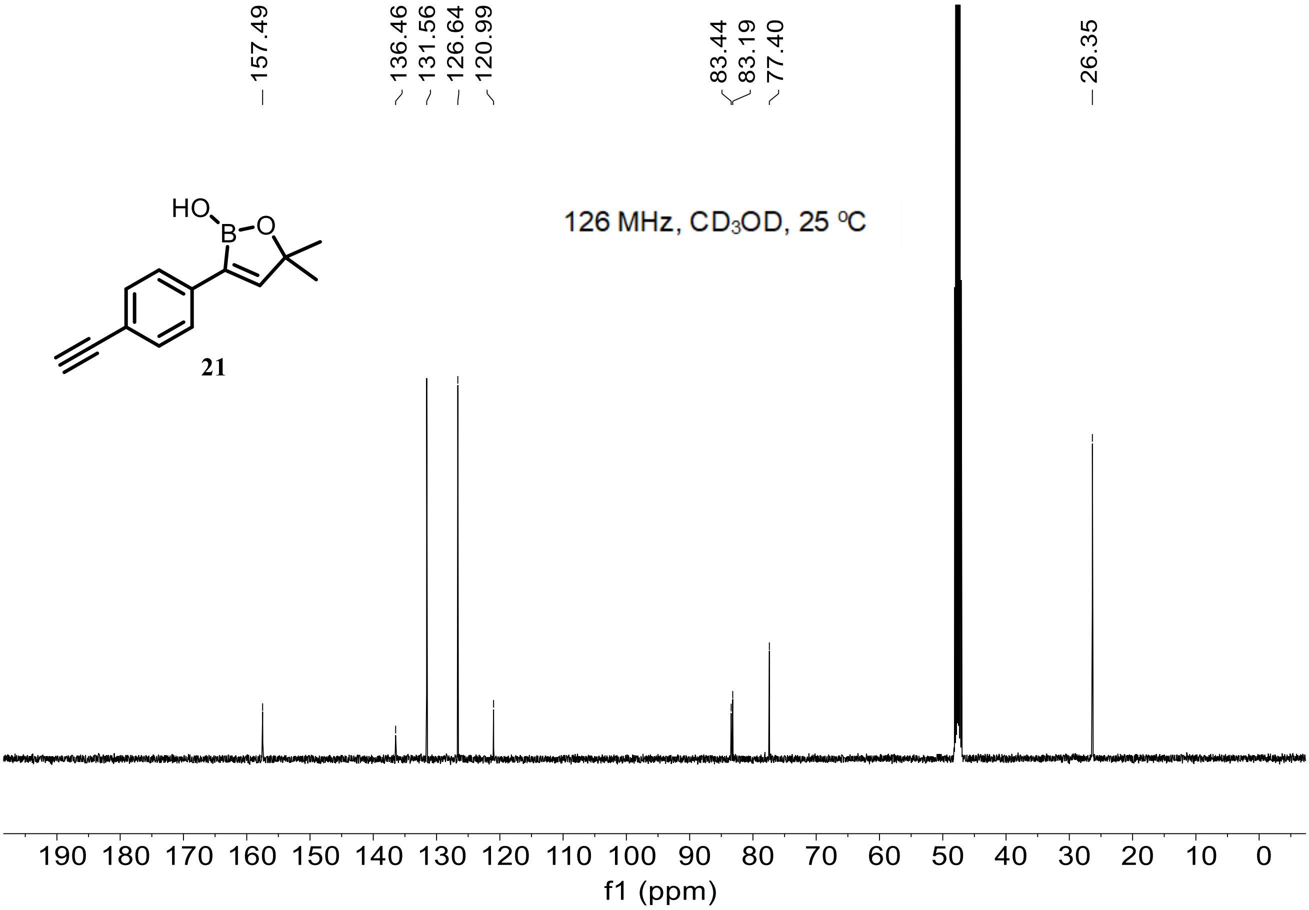
157.49
136.46
131.56
126.64
120.99
83.44
83.19
77.40
26.35
126 MHz, CD_3OD, 25 °C
HO
B
O
21
190 180 170 160 150 140 130 120 110 100 90 80 70 60 50 40 30 20 10 0
f1 (ppm)

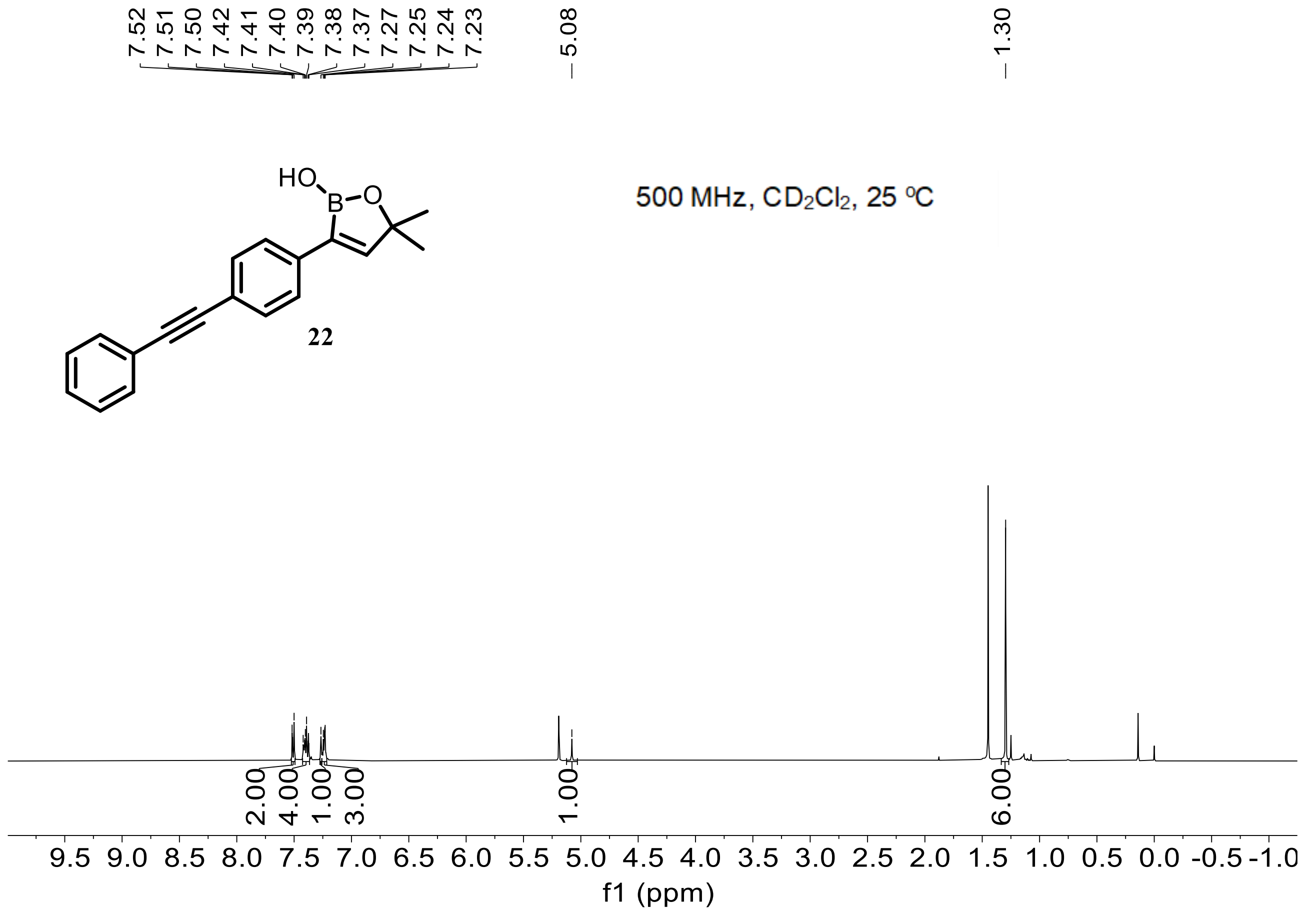
7.52
7.51
7.50
7.42
7.41
7.40
7.39
7.38
7.37
7.27
7.25
7.24
7.23
5.08
1.30
HO
B
O
22
500 MHz, CD2Cl2, 25 ºC
2.00
4.00
1.00
3.00
1.00
6.00
9.5 9.0 8.5 8.0 7.5 7.0 6.5 6.0 5.5 5.0 4.5 4.0 3.5 3.0 2.5 2.0 1.5 1.0 0.5 0.0 -0.5 -1.0
f1 (ppm)

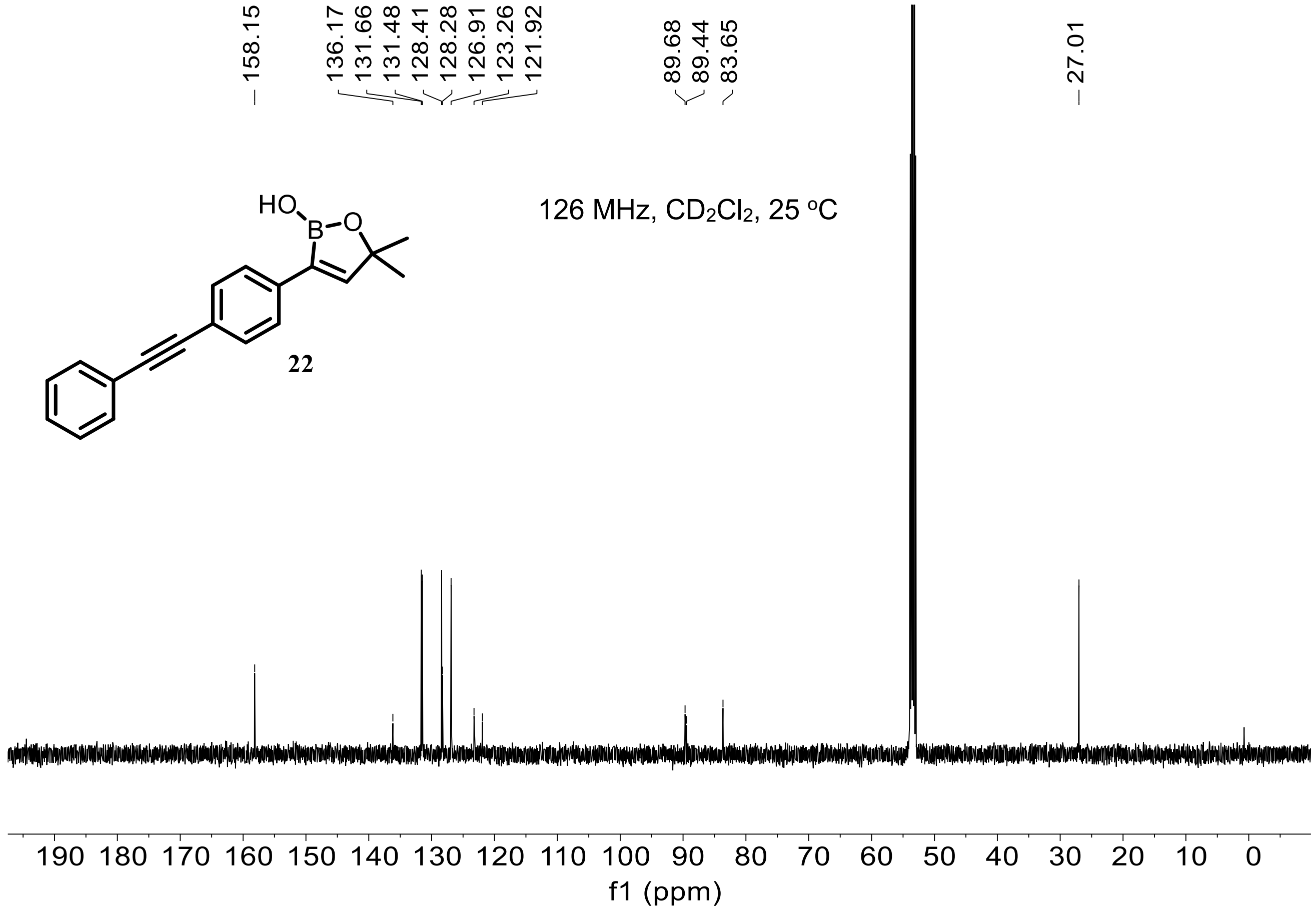
158.15
136.17
131.66
131.48
128.41
128.28
126.91
123.26
121.92
89.68
89.44
83.65
27.01
HO
B
O
22
126 MHz, CD2Cl2, 25 ºC
190 180 170 160 150 140 130 120 110 100 90 80 70 60 50 40 30 20 10 0
f1 (ppm)

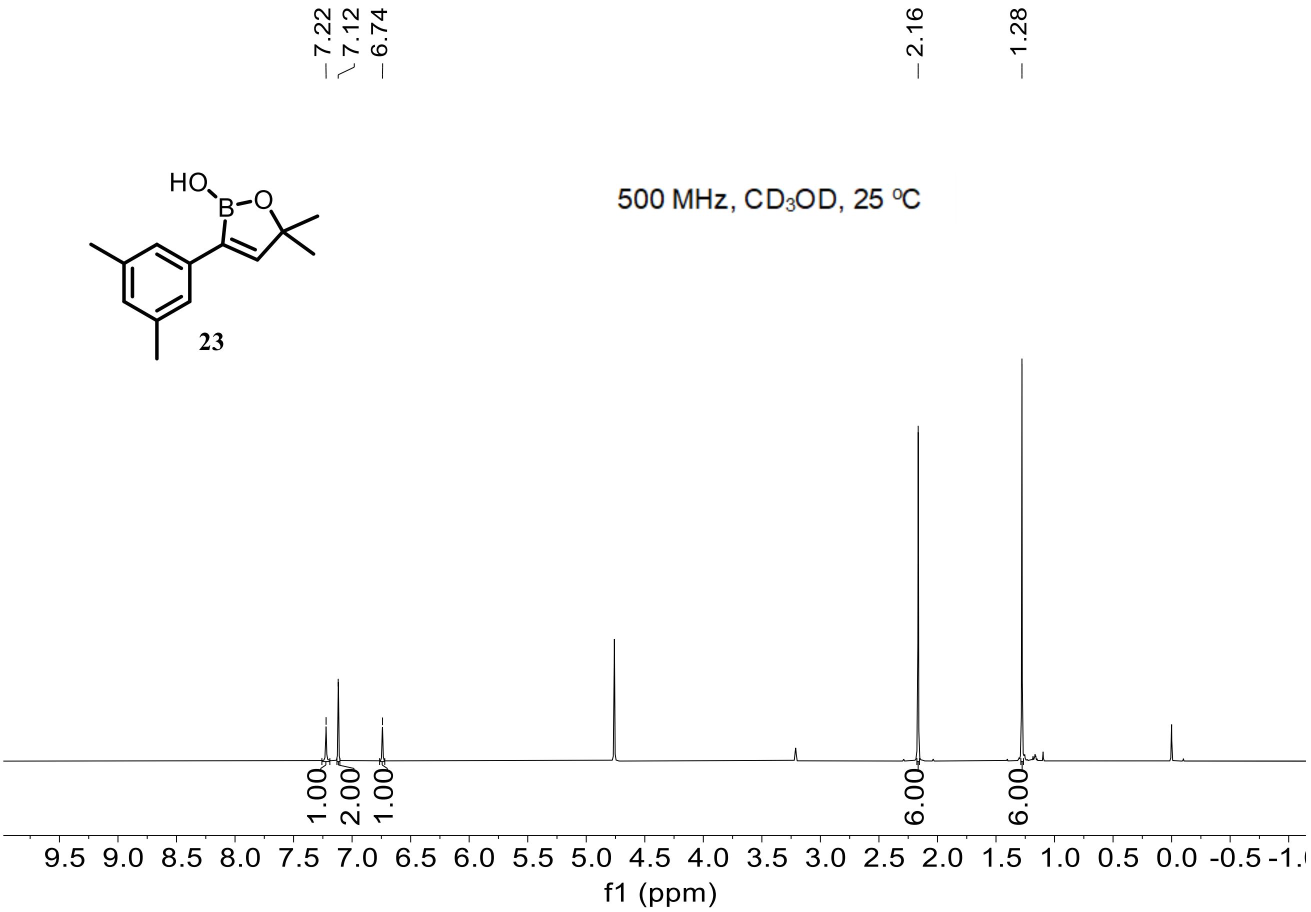
7.22
7.12
6.74
2.16
1.28
HO
B
O
23
500 MHz, CD3OD, 25 ºC
1.00
2.00
1.00
6.00
6.00
9.5 9.0 8.5 8.0 7.5 7.0 6.5 6.0 5.5 5.0 4.5 4.0 3.5 3.0 2.5 2.0 1.5 1.0 0.5 0.0 -0.5
f1 (ppm)

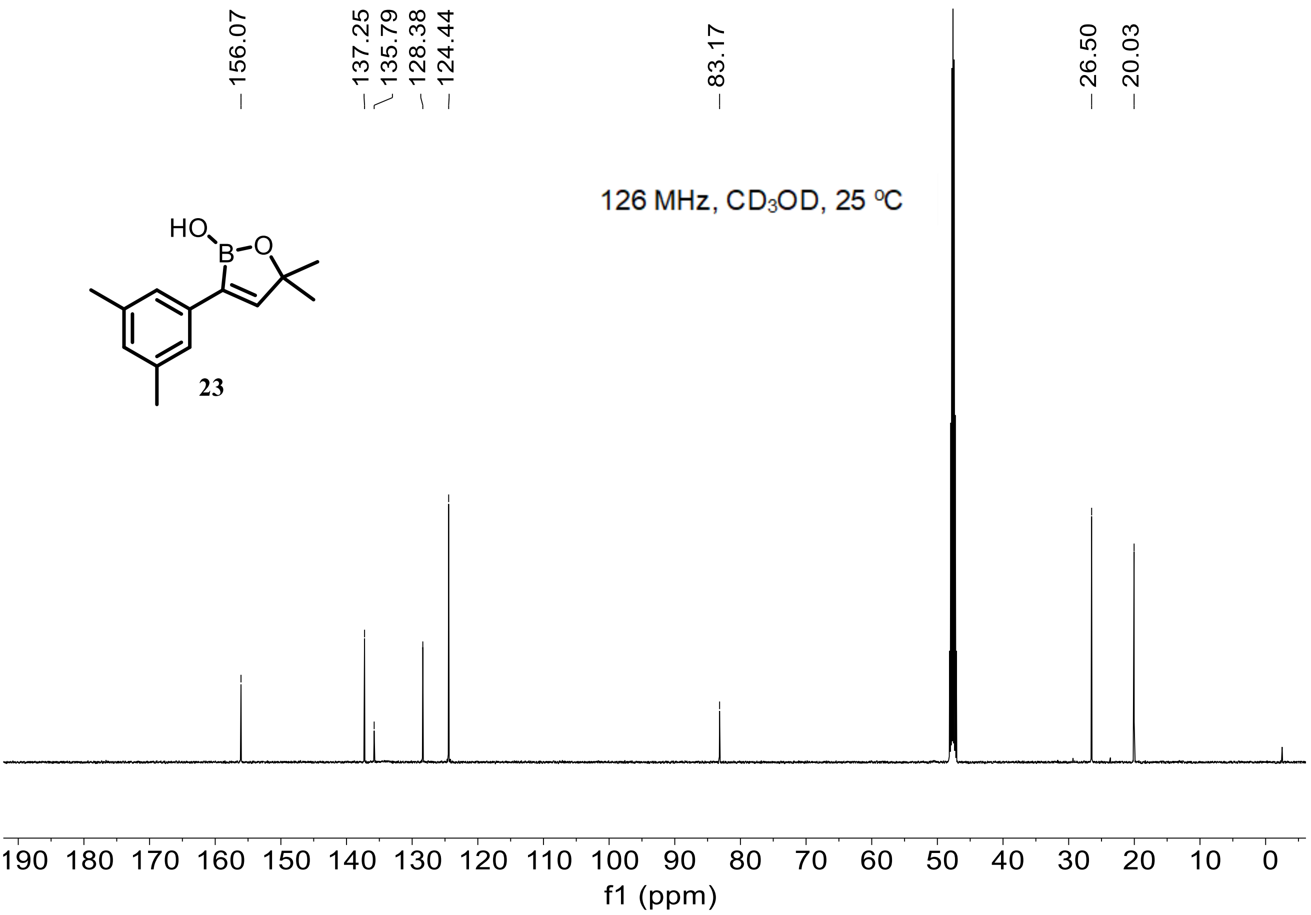
156.07
137.25
135.79
128.38
124.44
83.17
26.50
20.03
126 MHz, CD3OD, 25 ºC
HO
B
O
23
190 180 170 160 150 140 130 120 110 100 90 80 70 60 50 40 30 20 10 0
f1 (ppm)

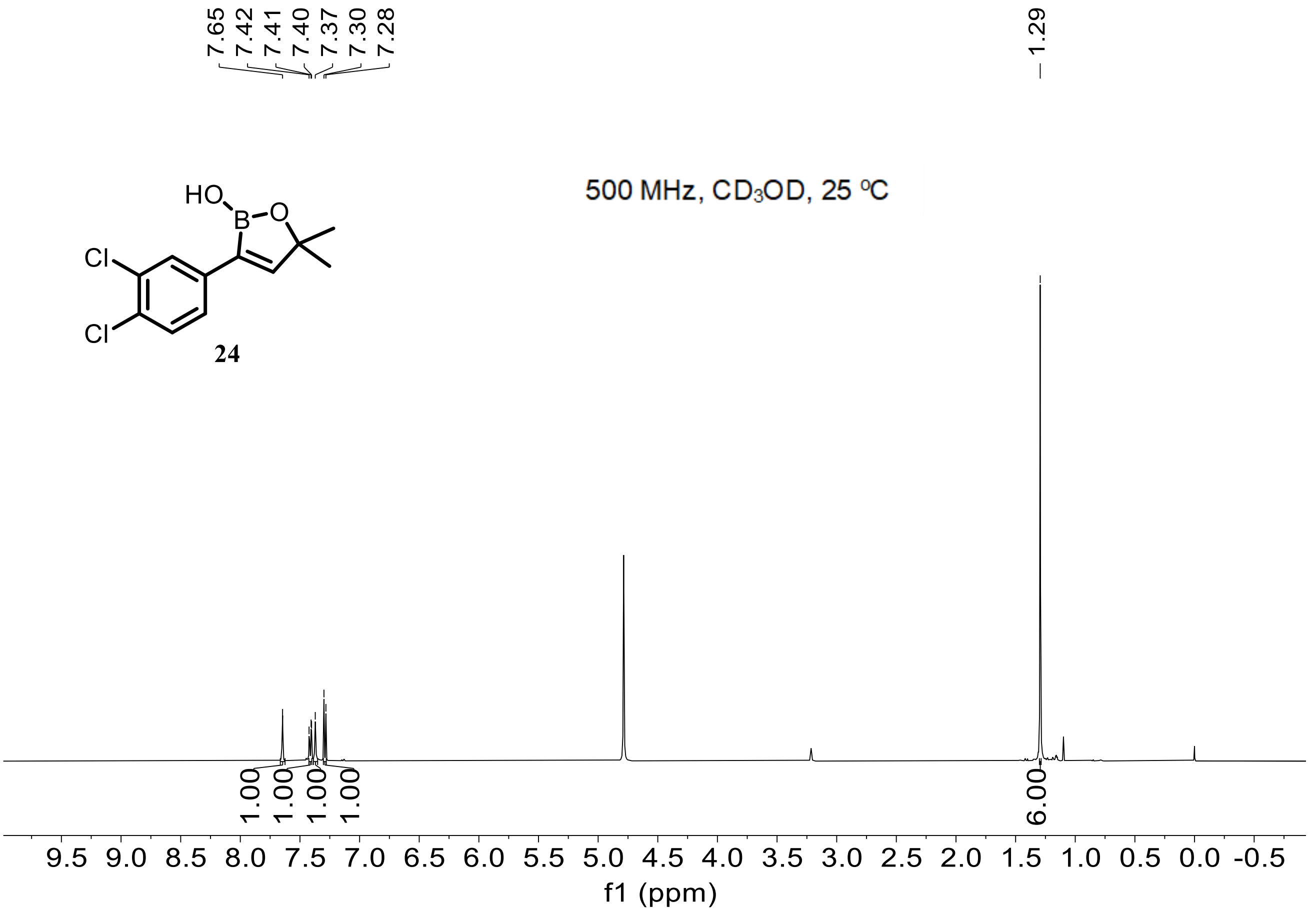
7.65
7.42
7.41
7.40
7.37
7.30
7.28
1.29
HO
B
O
Cl
Cl
24
500 MHz, CD3OD, 25 °C
1.00
1.00
1.00
1.00
6.00
9.5 9.0 8.5 8.0 7.5 7.0 6.5 6.0 5.5 5.0 4.5 4.0 3.5 3.0 2.5 2.0 1.5 1.0 0.5 0.0 -0.5
f1 (ppm)

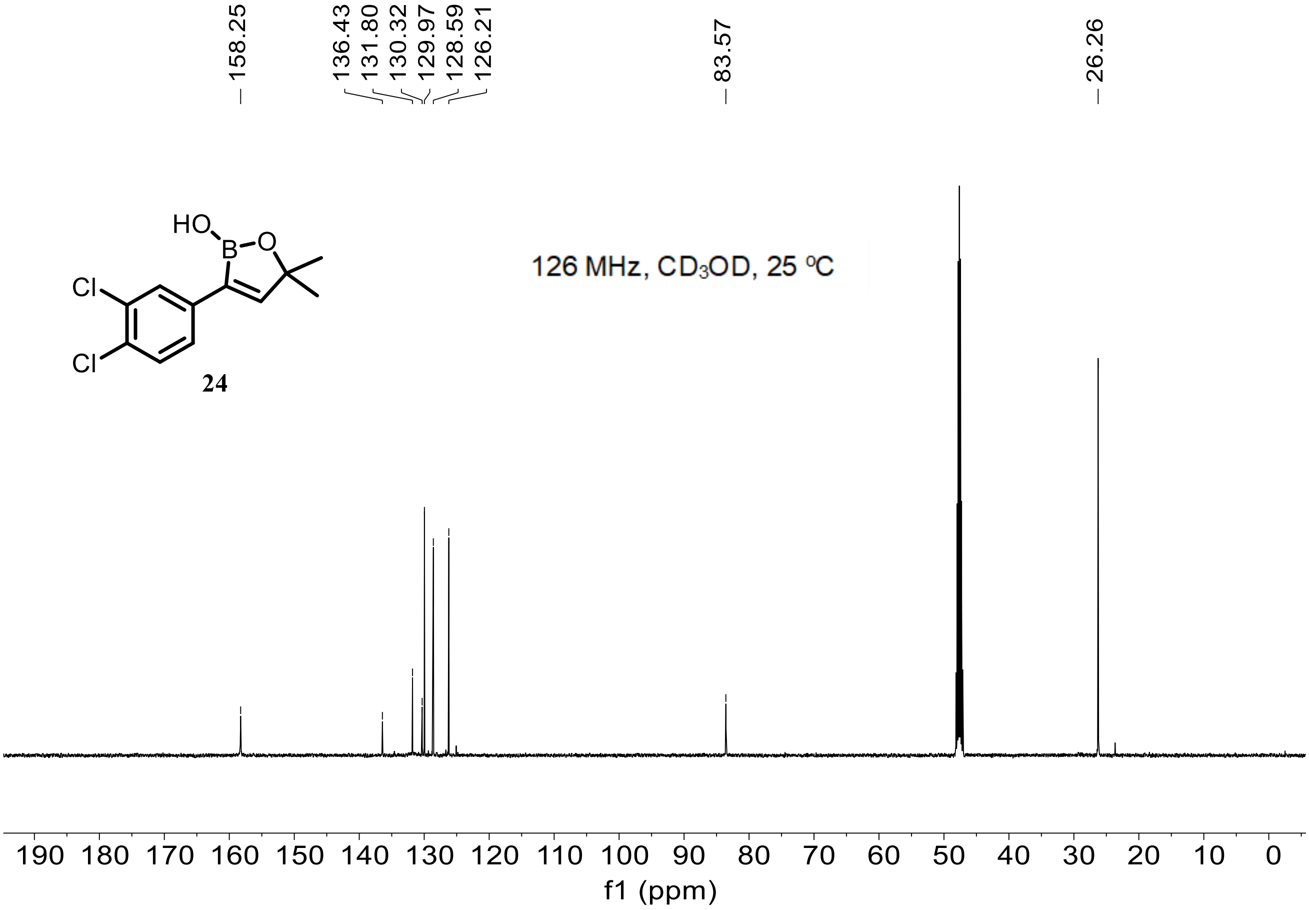
158.25
136.43
131.80
130.32
129.97
128.59
126.21
83.57
26.26
HO
B
O
Cl
Cl
24
126 MHz, CD3OD, 25 °C
190 180 170 160 150 140 130 120 110 100 90 80 70 60 50 40 30 20 10 0
f1 (ppm)

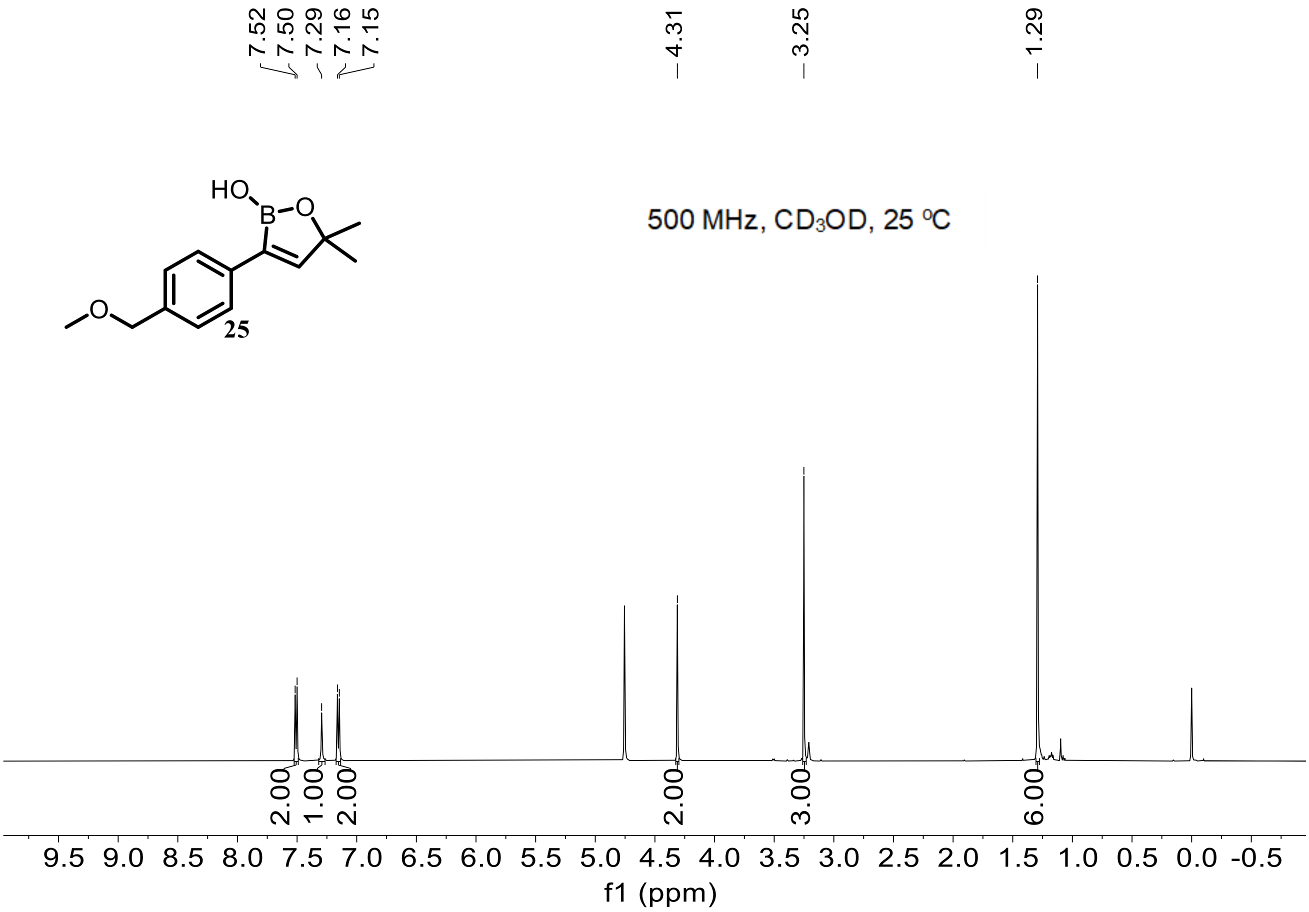
7.52
7.50
7.29
7.16
7.15
4.31
3.25
1.29
HO
B
O
25
500 MHz, CD3OD, 25 °C
2.00
1.00
2.00
2.00
3.00
6.00
9.5 9.0 8.5 8.0 7.5 7.0 6.5 6.0 5.5 5.0 4.5 4.0 3.5 3.0 2.5 2.0 1.5 1.0 0.5 0.0 -0.5
f1 (ppm)

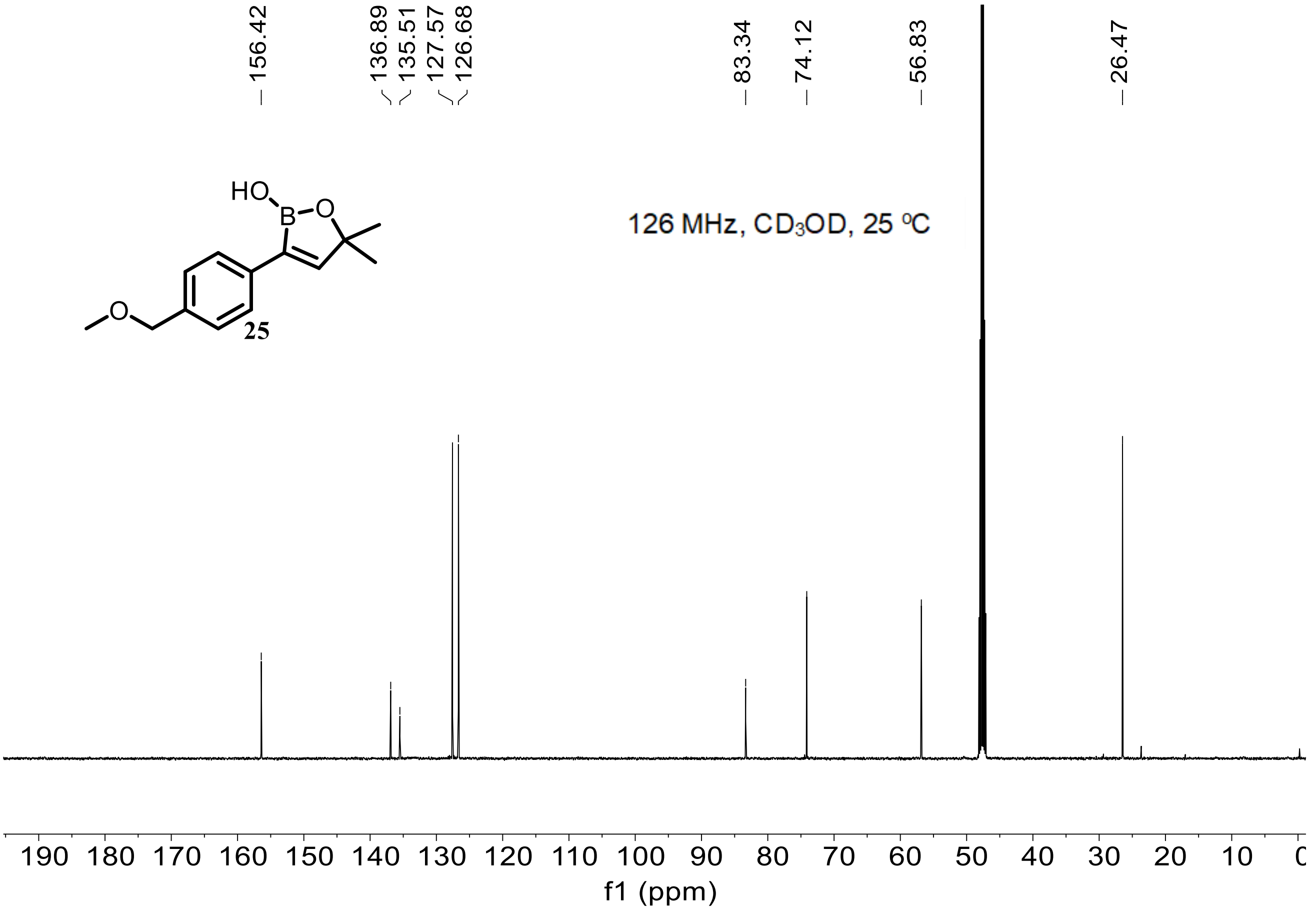
156.42
136.89
135.51
127.57
126.68
83.34
74.12
56.83
26.47
HO
B
O
25
126 MHz, CD3OD, 25 °C
190 180 170 160 150 140 130 120 110 100 90 80 70 60 50 40 30 20 10
f1 (ppm)

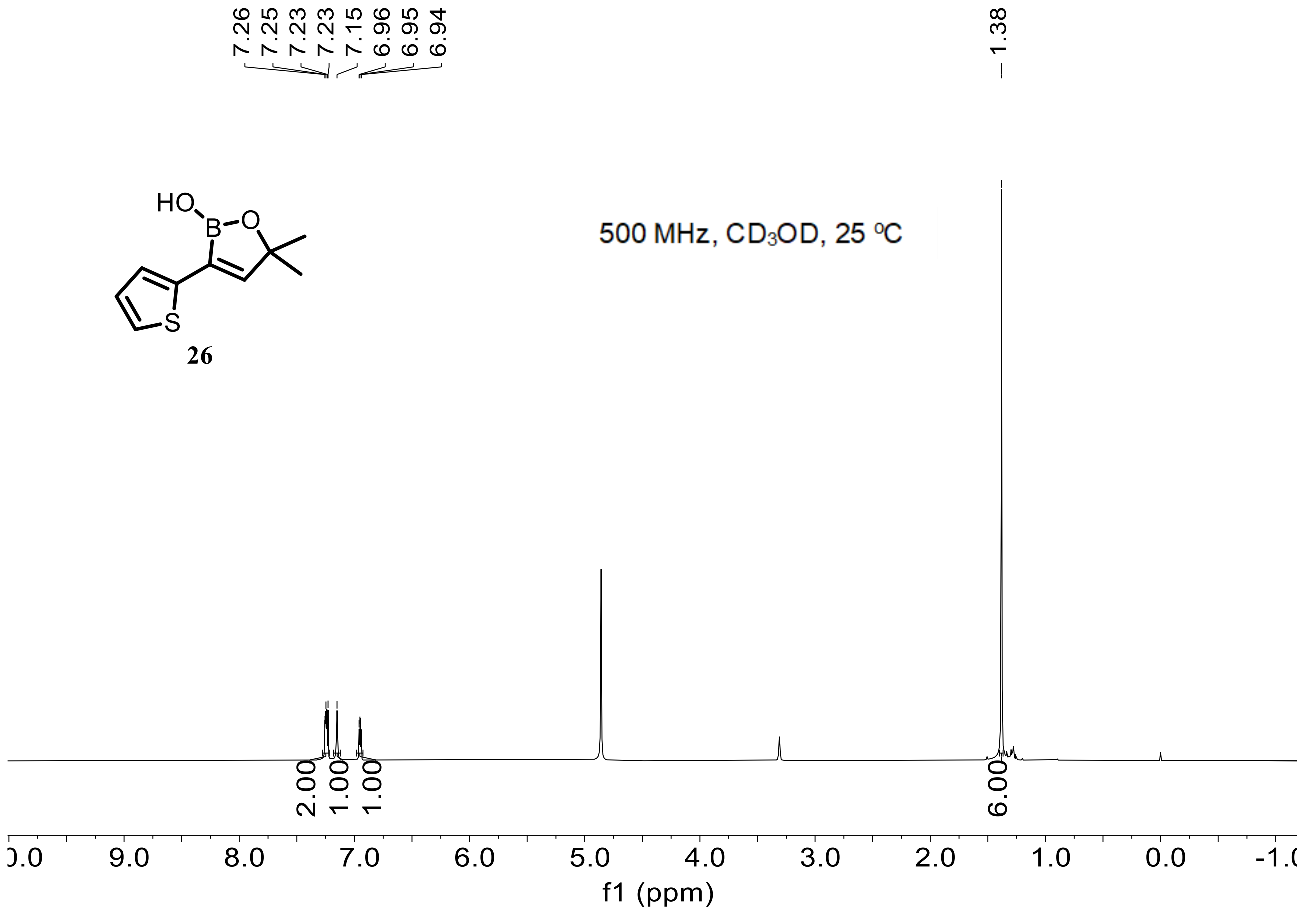
7.26 7.25 7.23 7.23 7.15 6.96 6.95 6.94
1.38
500 MHz, CD3OD, 25 °C
HO B O S
26
2.00 1.00 1.00
6.00
f1 (ppm)

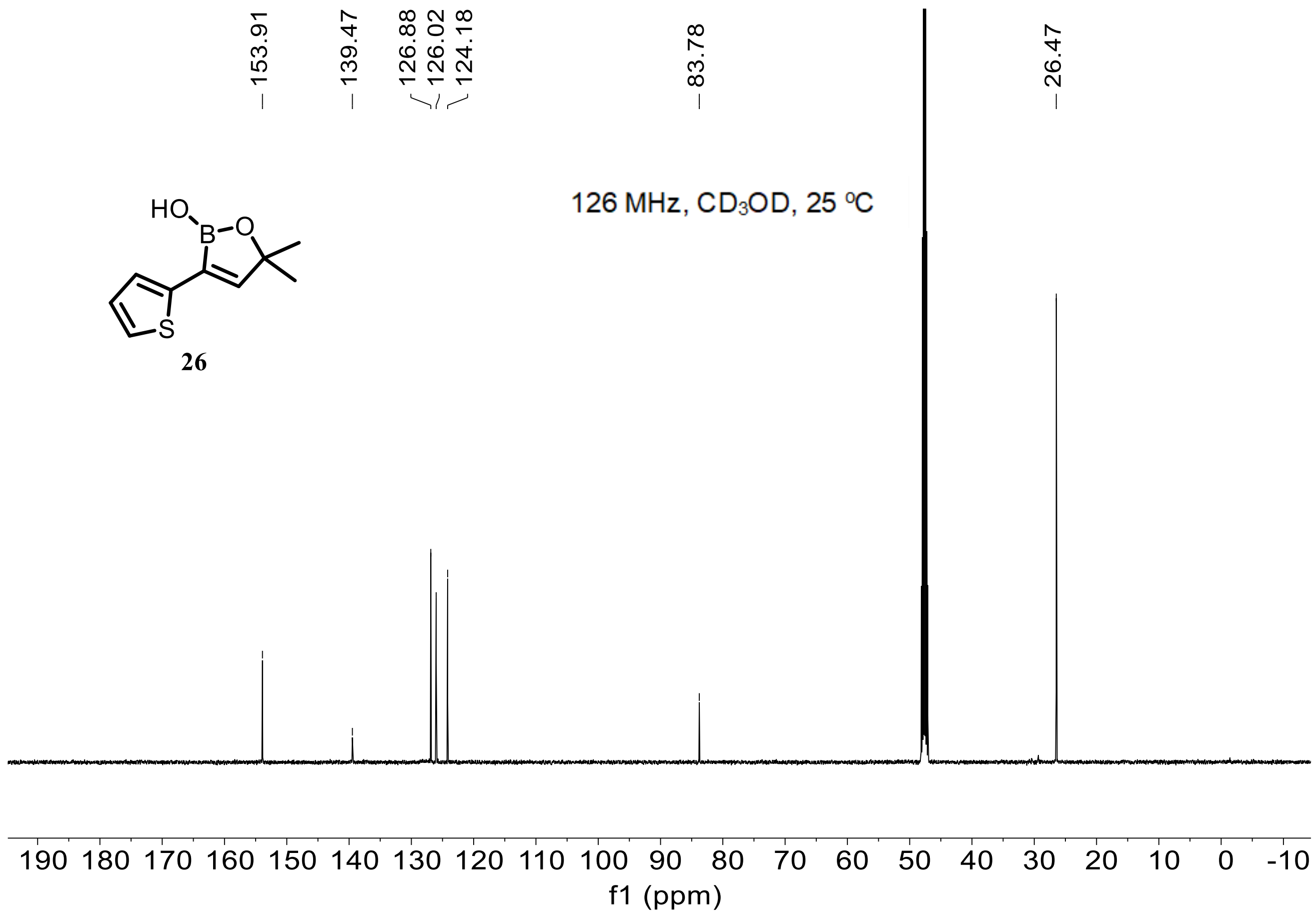
153.91
139.47
126.88 126.02 124.18
83.78
26.47
126 MHz, CD3OD, 25 °C
HO B O S
26
f1 (ppm)

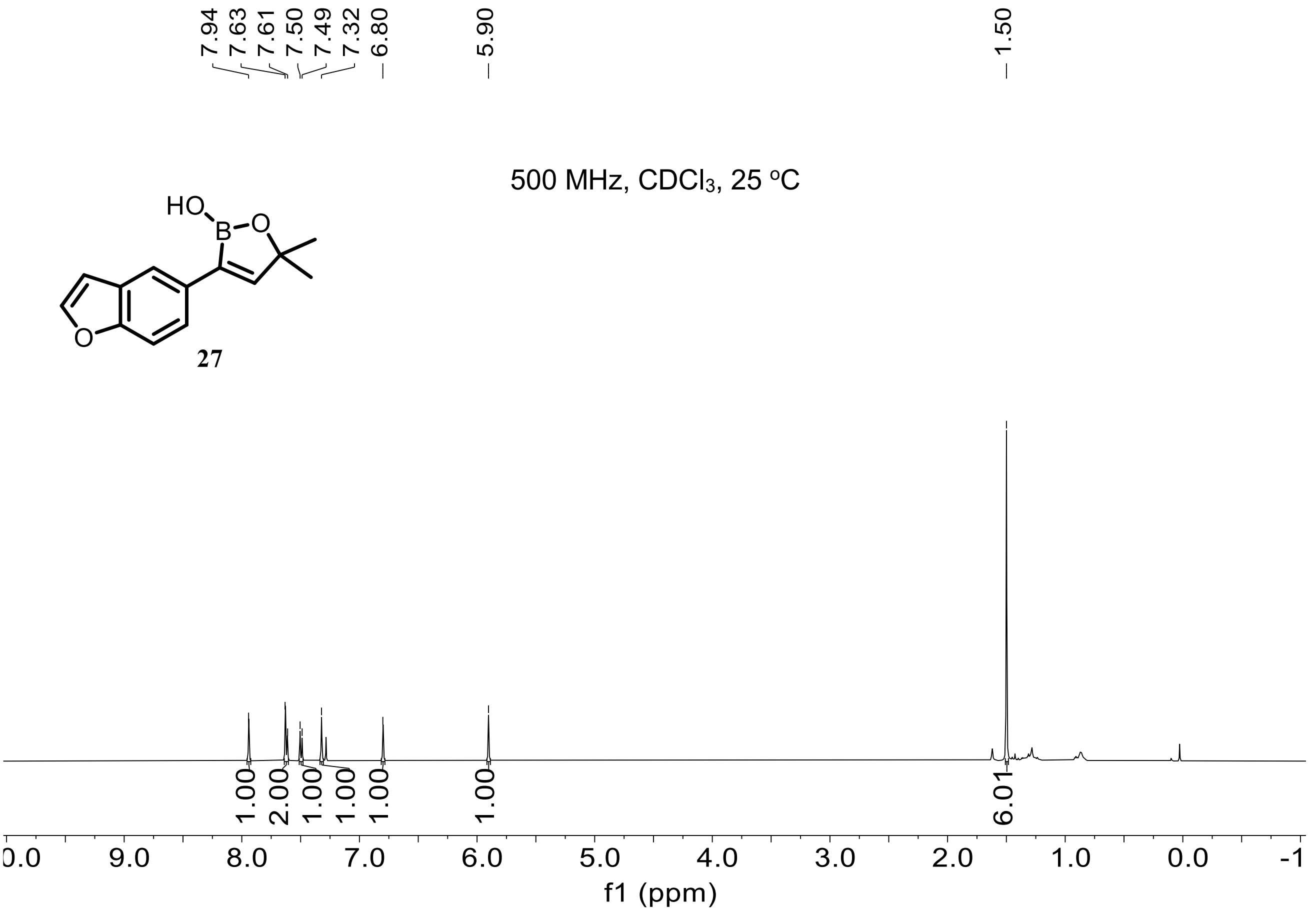
7.94
7.63
7.61
7.50
7.49
7.32
6.80
5.90
1.50
500 MHz, CDCl3, 25 °C
HO
B
O
O
27
1.00
2.00
1.00
1.00
1.00
1.00
6.01
10.0 9.0 8.0 7.0 6.0 5.0 4.0 3.0 2.0 1.0 0.0 -1
f1 (ppm)

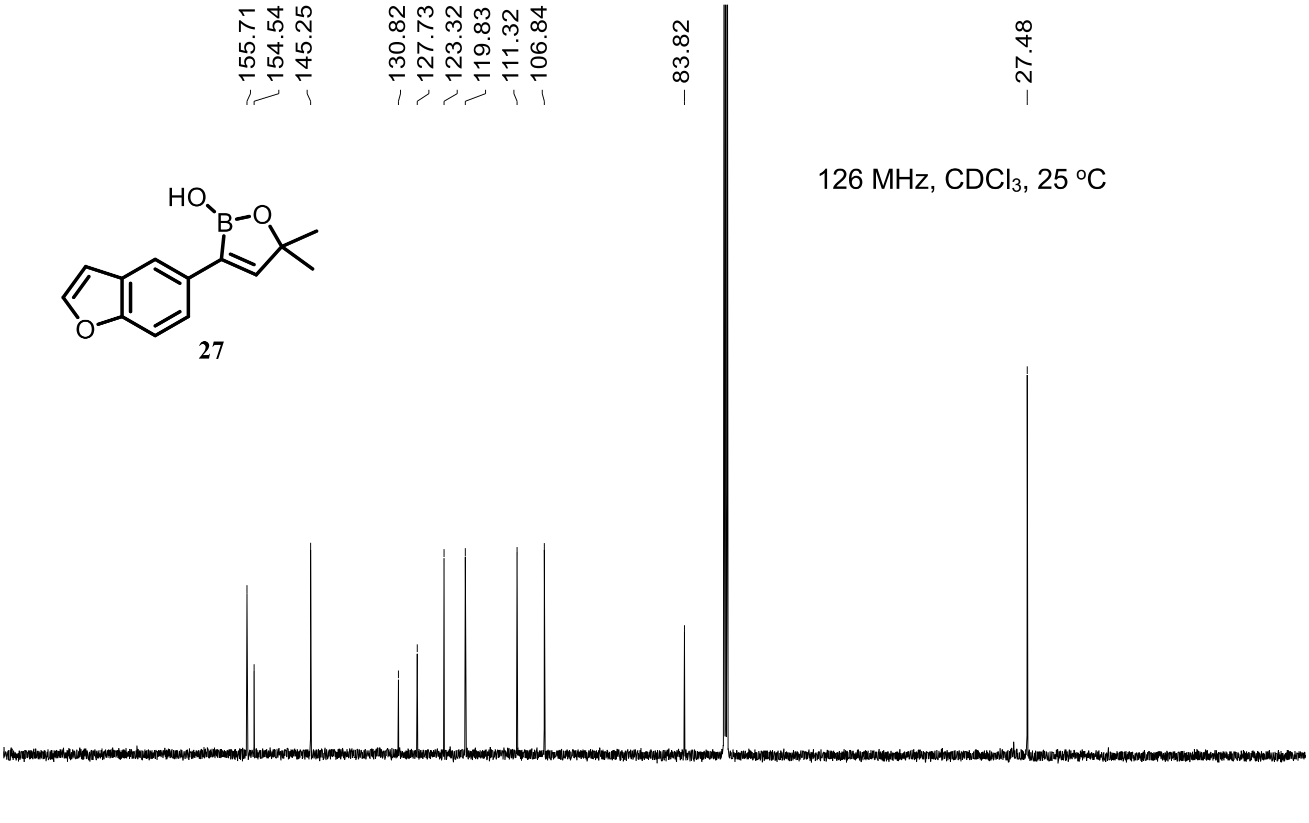
155.71
154.54
145.25
130.82
127.73
123.32
119.83
111.32
106.84
83.82
27.48
126 MHz, CDCl3, 25 °C
HO
B
O
O
27
190 180 170 160 150 140 130 120 110 100 90 80 70 60 50 40 30 20 10 0 -10
f1 (ppm)

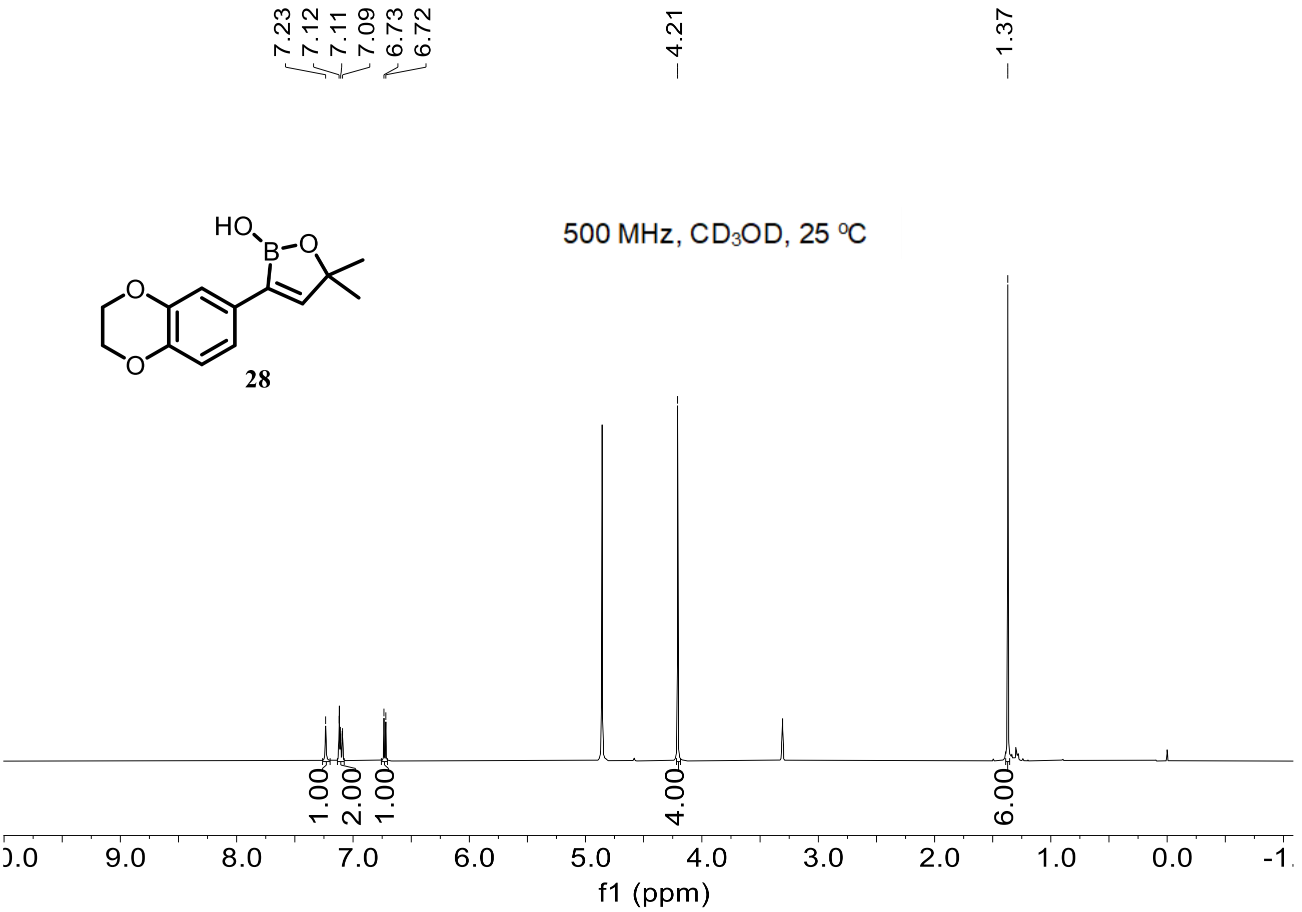
7.23
7.12
7.11
7.09
6.73
6.72
4.21
1.37
HO
B
O
O
O
28
500 MHz, CD3OD, 25 ºC
1.00
2.00
1.00
4.00
6.00
9.0
8.0
7.0
6.0
5.0
4.0
3.0
2.0
1.0
0.0
f1 (ppm)

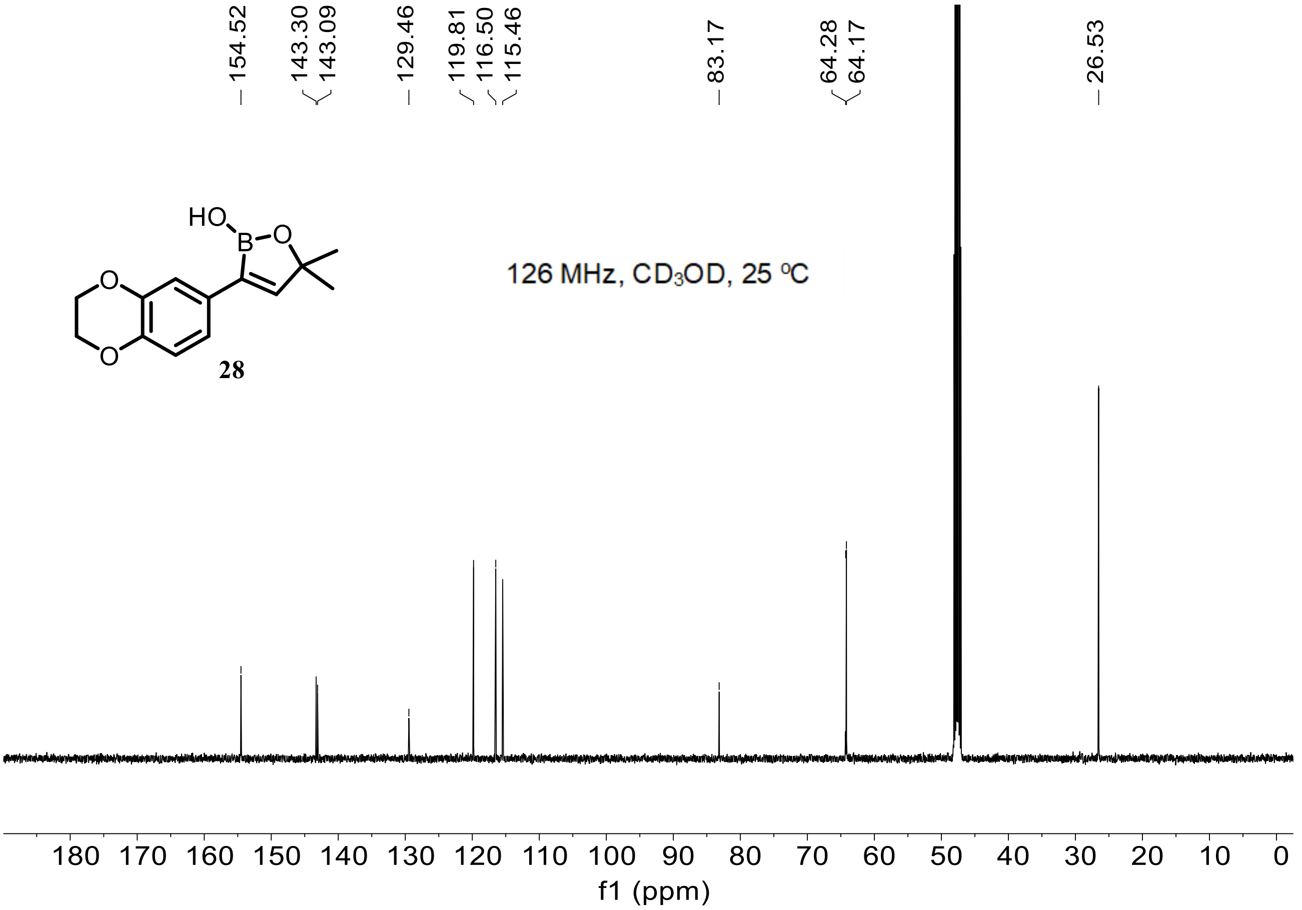
154.52
143.30
143.09
129.46
119.81
116.50
115.46
83.17
64.28
64.17
26.53
HO
B
O
O
O
28
126 MHz, CD3OD, 25 ºC
180
170
160
150
140
130
120
110
100
90
80
70
60
50
40
30
20
10
0
f1 (ppm)

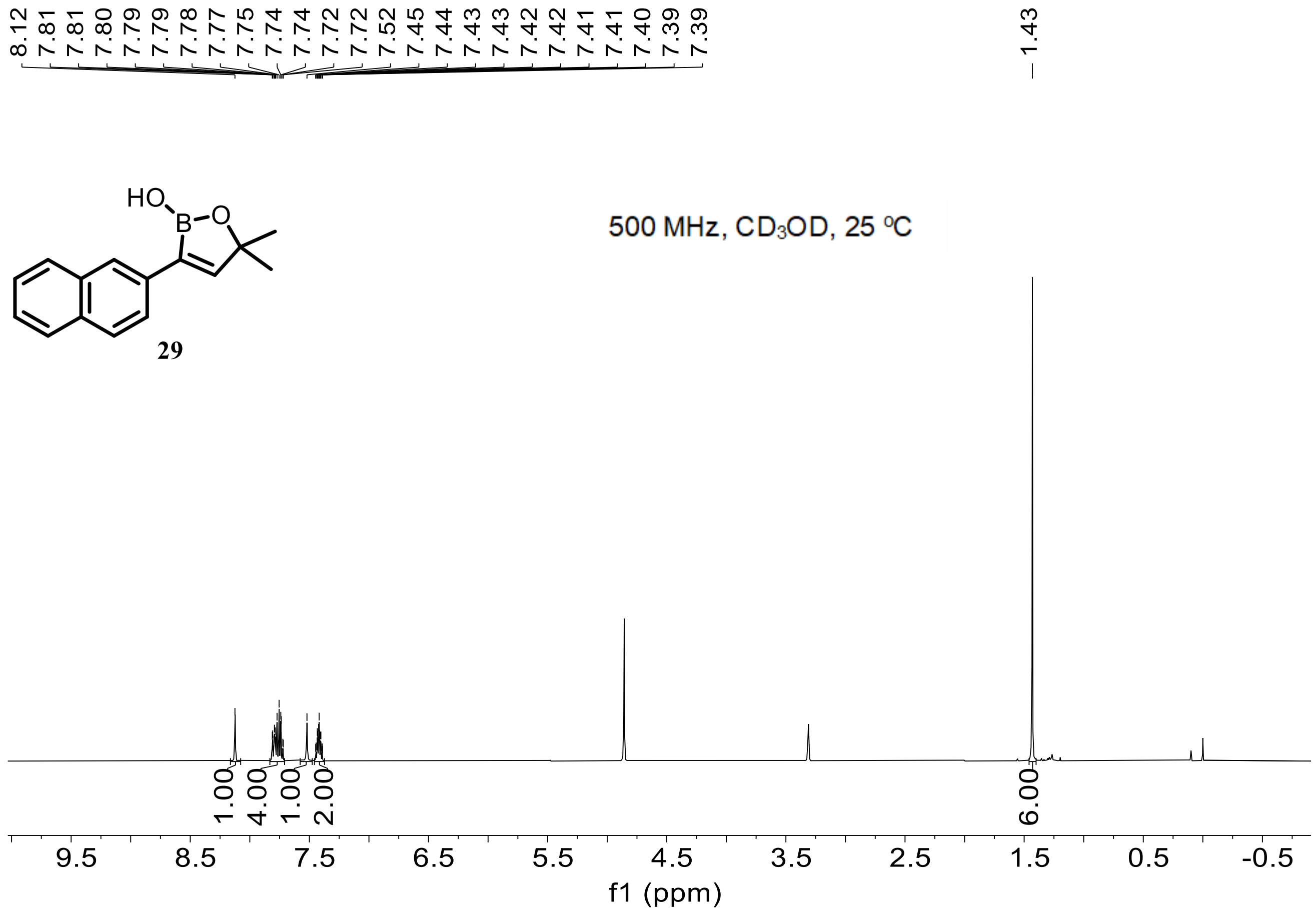
8.12 7.81 7.81 7.80 7.79 7.79 7.78 7.77 7.75 7.74 7.74 7.72 7.72 7.52 7.45 7.44 7.43 7.43 7.42 7.42 7.41 7.41 7.40 7.39 7.39
1.43
HO
B
O
29
500 MHz, CD3OD, 25 °C
1.00 4.00 1.00 2.00
6.00
9.5 8.5 7.5 6.5 5.5 4.5 3.5 2.5 1.5 0.5 -0.5
f1 (ppm)

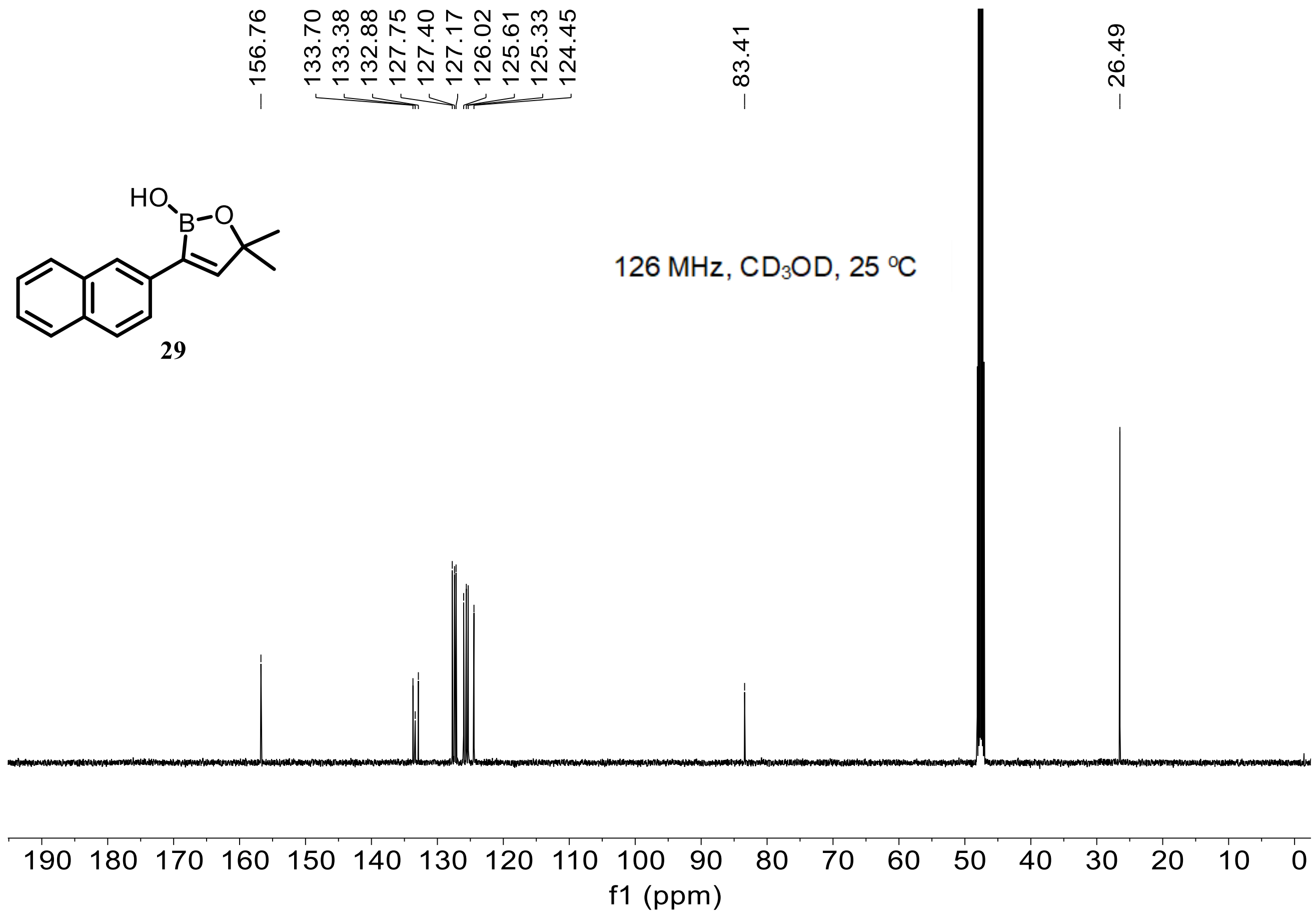
156.76
133.70 133.38 132.88 127.75 127.40 127.17 126.02 125.61 125.33 124.45
83.41
26.49
HO
B
O
29
126 MHz, CD3OD, 25 °C
190 180 170 160 150 140 130 120 110 100 90 80 70 60 50 40 30 20 10 0
f1 (ppm)

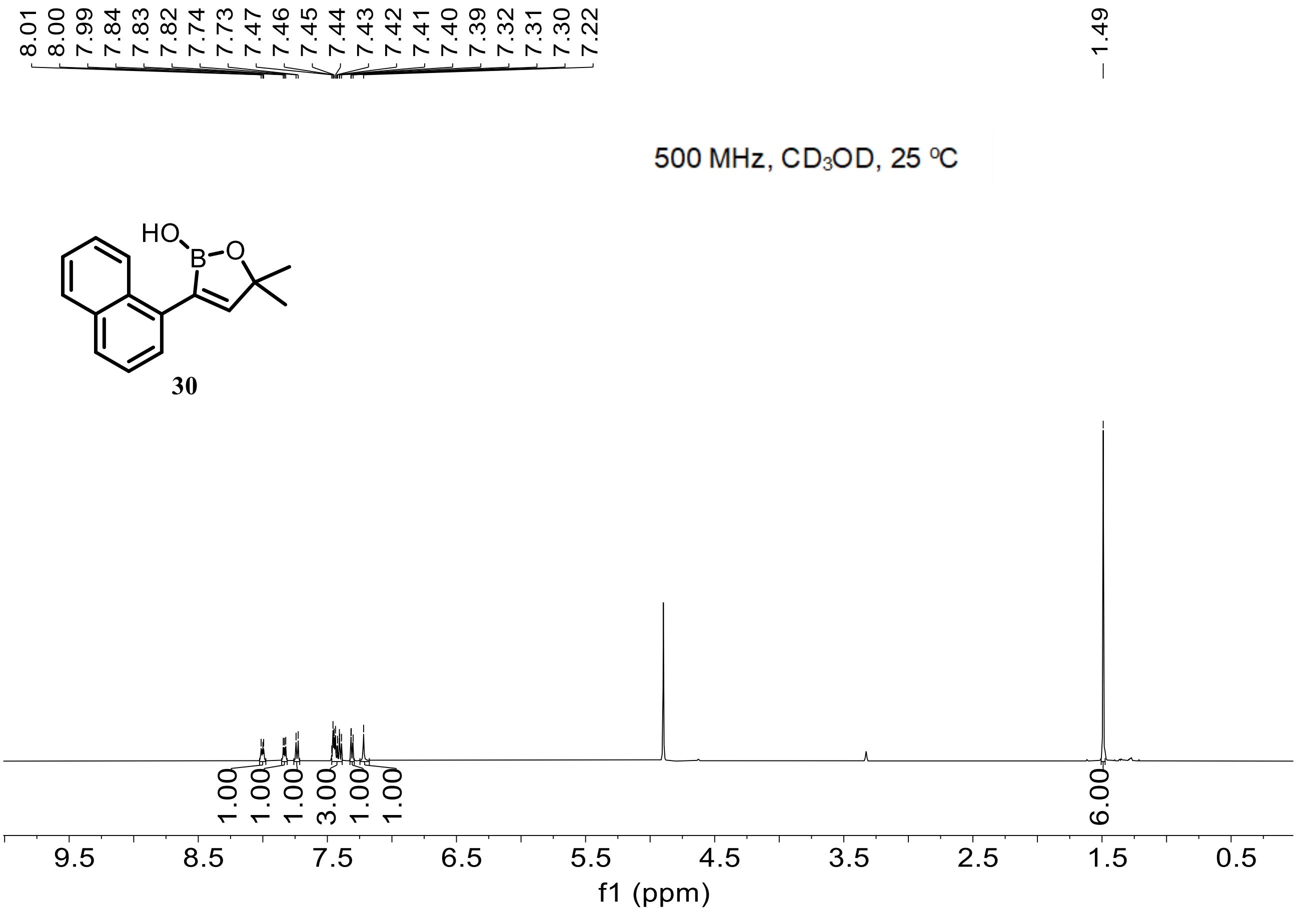
8.01 8.00 7.99 7.84 7.83 7.82 7.74 7.73 7.47 7.46 7.45 7.44 7.43 7.42 7.41 7.40 7.39 7.32 7.31 7.30 7.22
1.49
500 MHz, CD3OD, 25 °C
HO
B
O
30
1.00 1.00 1.00 3.00 1.00 1.00
6.00
9.5 8.5 7.5 6.5 5.5 4.5 3.5 2.5 1.5 0.5
f1 (ppm)

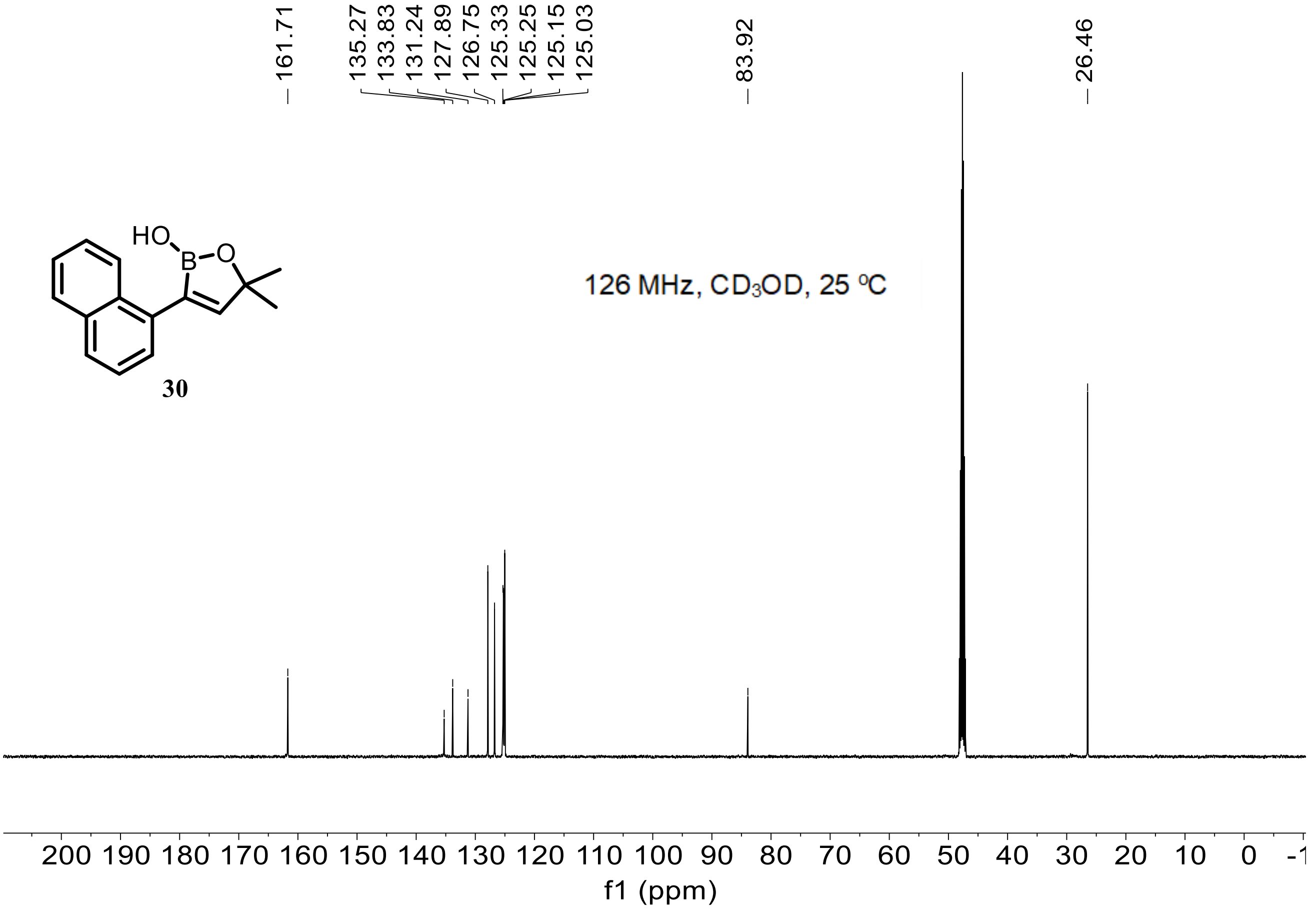
161.71
135.27 133.83 131.24 127.89 126.75 125.33 125.25 125.15 125.03
83.92
26.46
HO
B
O
30
126 MHz, CD3OD, 25 °C
200 190 180 170 160 150 140 130 120 110 100 90 80 70 60 50 40 30 20 10 0 -1
f1 (ppm)

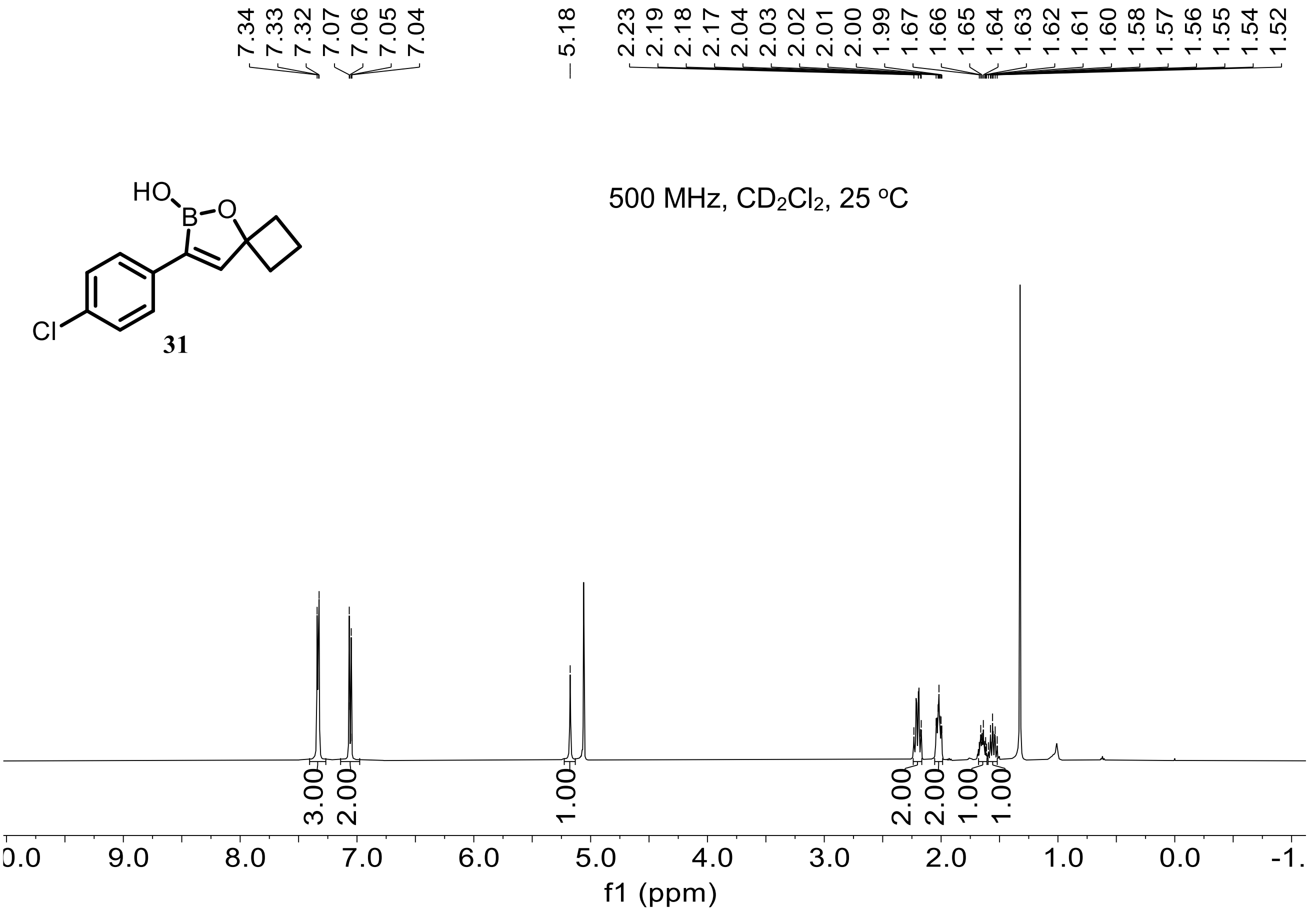
7.34 7.33 7.32 7.07 7.06 7.05 7.04
5.18
2.23 2.19 2.18 2.17 2.04 2.03 2.02 2.01 2.00 1.99 1.67 1.66 1.65 1.64 1.63 1.62 1.61 1.60 1.58 1.57 1.56 1.55 1.54 1.52
HO B O
Cl
31
500 MHz, CD_2Cl_2, 25 °C
3.00 2.00 1.00 2.00 2.00 1.00 1.00
9.0 8.0 7.0 6.0 5.0 4.0 3.0 2.0 1.0 0.0
f1 (ppm)

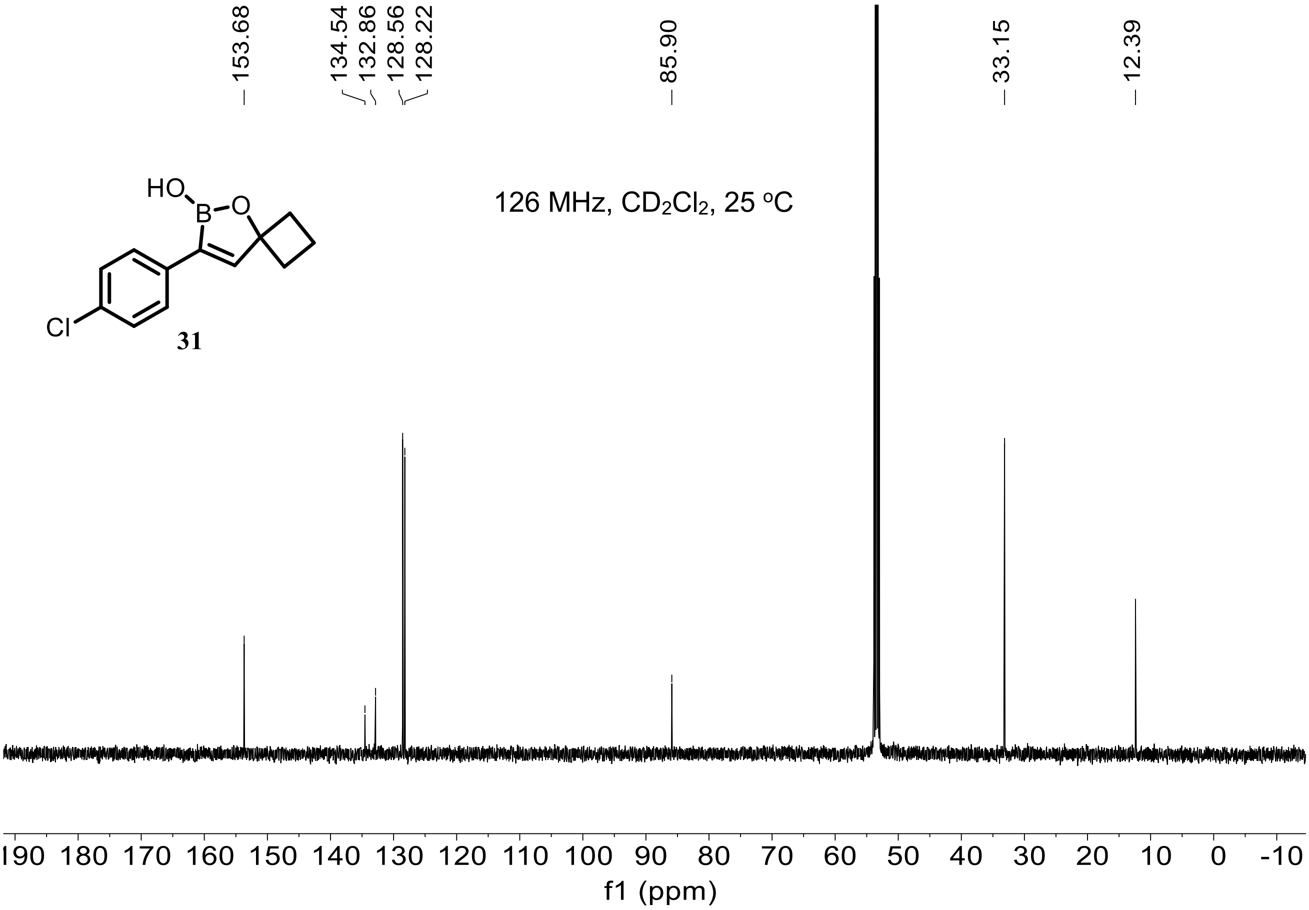
153.68
134.54 132.86 128.56 128.22
85.90
33.15
12.39
HO B O
Cl
31
126 MHz, CD_2Cl_2, 25 °C
190 180 170 160 150 140 130 120 110 100 90 80 70 60 50 40 30 20 10 0 -10
f1 (ppm)

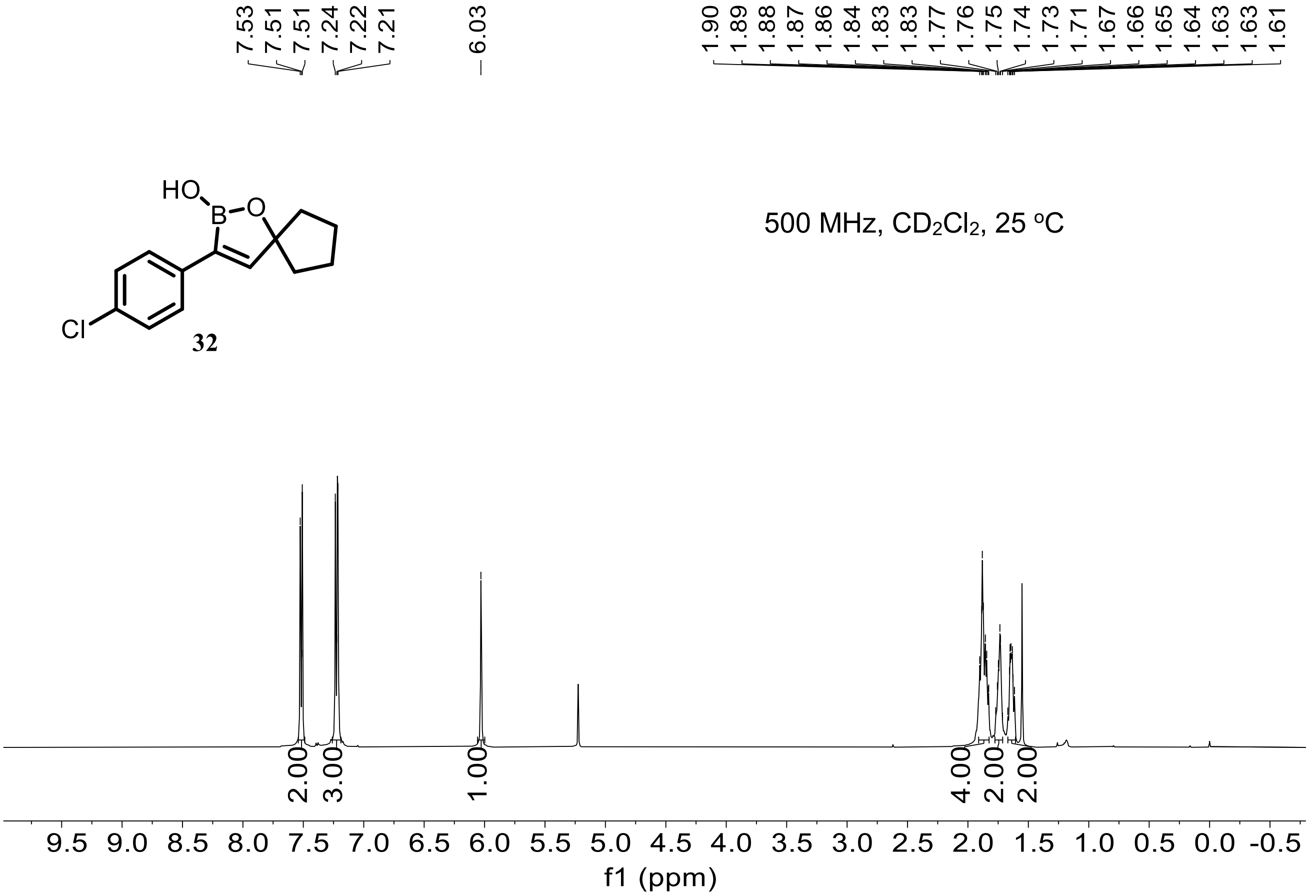
7.53 7.51 7.51 7.24 7.22 7.21
6.03
1.90 1.89 1.88 1.87 1.86 1.84 1.83 1.83 1.77 1.76 1.75 1.74 1.73 1.71 1.67 1.66 1.65 1.64 1.63 1.63 1.61
HO
B
O
Cl
32
500 MHz, CD2Cl2, 25 °C
2.00 3.00 1.00 4.00 2.00 2.00
9.5 9.0 8.5 8.0 7.5 7.0 6.5 6.0 5.5 5.0 4.5 4.0 3.5 3.0 2.5 2.0 1.5 1.0 0.5 0.0 -0.5
f1 (ppm)

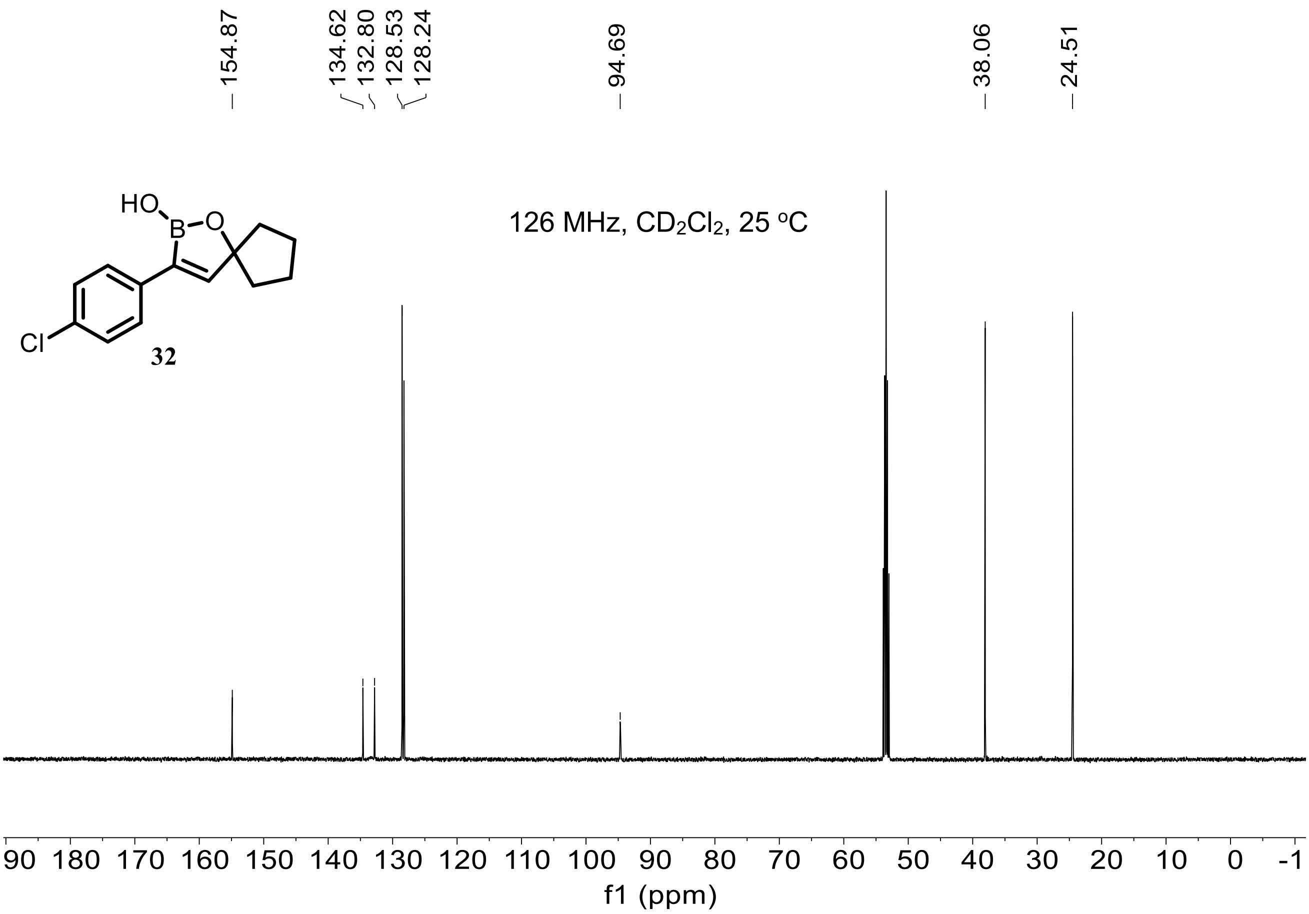
154.87
134.62 132.80 128.53 128.24
94.69
38.06
24.51
HO
B
O
Cl
32
126 MHz, CD2Cl2, 25 °C
90 180 170 160 150 140 130 120 110 100 90 80 70 60 50 40 30 20 10 0 -1
f1 (ppm)

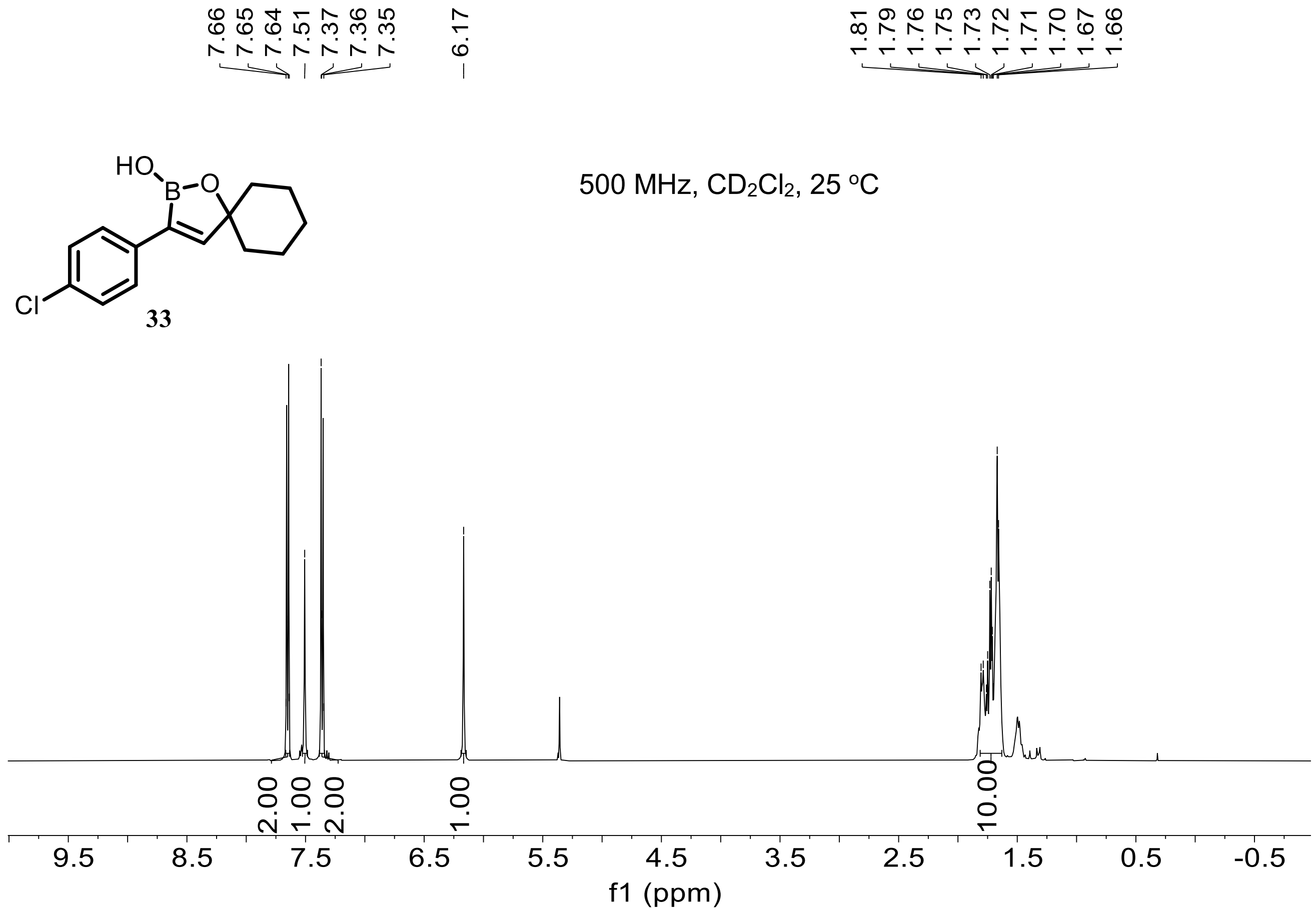
7.66
7.65
7.64
7.51
7.37
7.36
7.35
6.17
1.81
1.79
1.76
1.75
1.73
1.72
1.71
1.70
1.67
1.66
HO
B
O
Cl
33
500 MHz, CD_2Cl_2, 25 °C
2.00
1.00
2.00
1.00
10.00
9.5
8.5
7.5
6.5
5.5
4.5
3.5
2.5
1.5
0.5
-0.5
f1 (ppm)

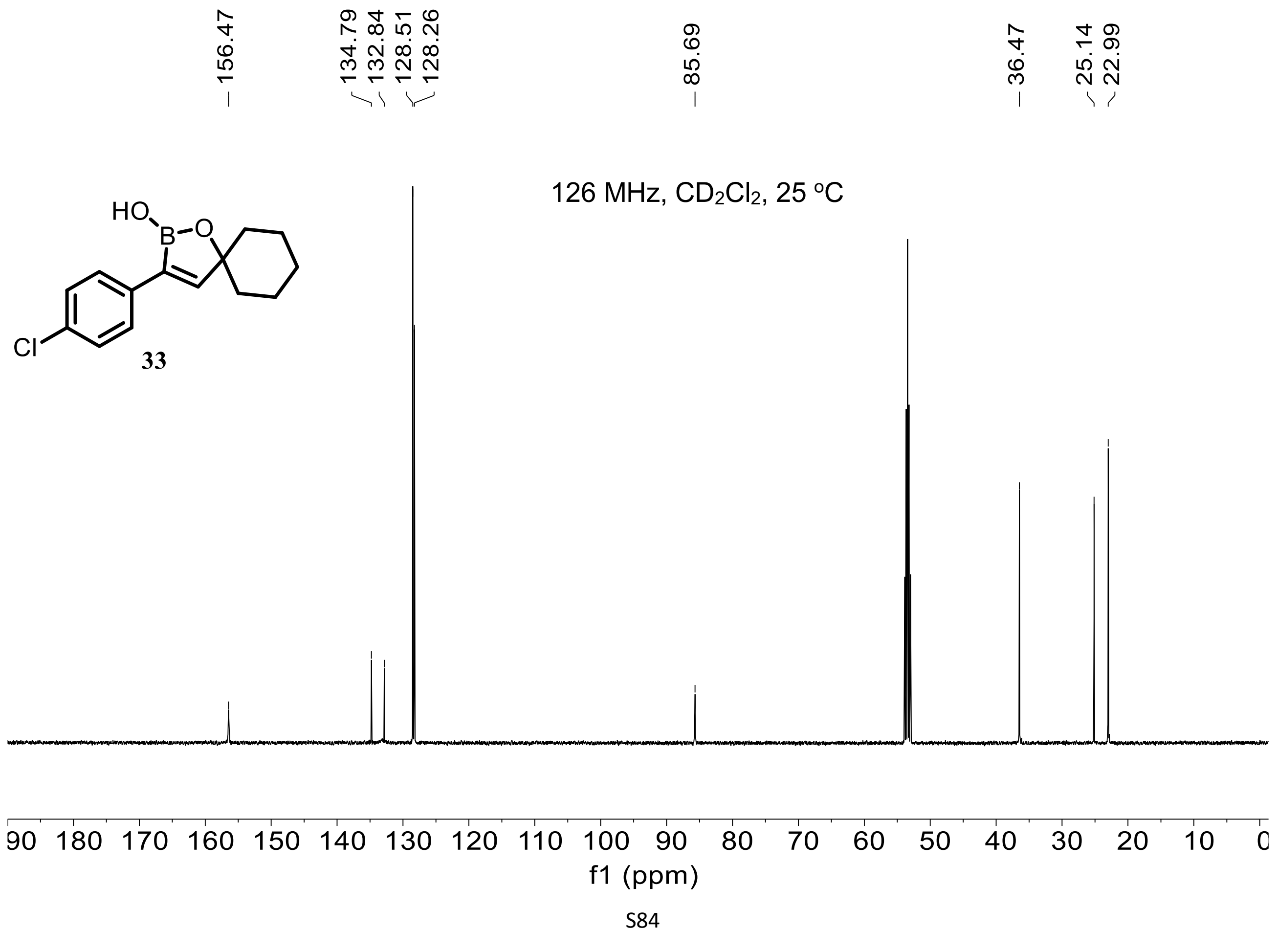
156.47
134.79
132.84
128.51
128.26
85.69
36.47
25.14
22.99
HO
B
O
Cl
33
126 MHz, CD_2Cl_2, 25 °C
90
180
170
160
150
140
130
120
110
100
90
80
70
60
50
40
30
20
10
0
f1 (ppm)

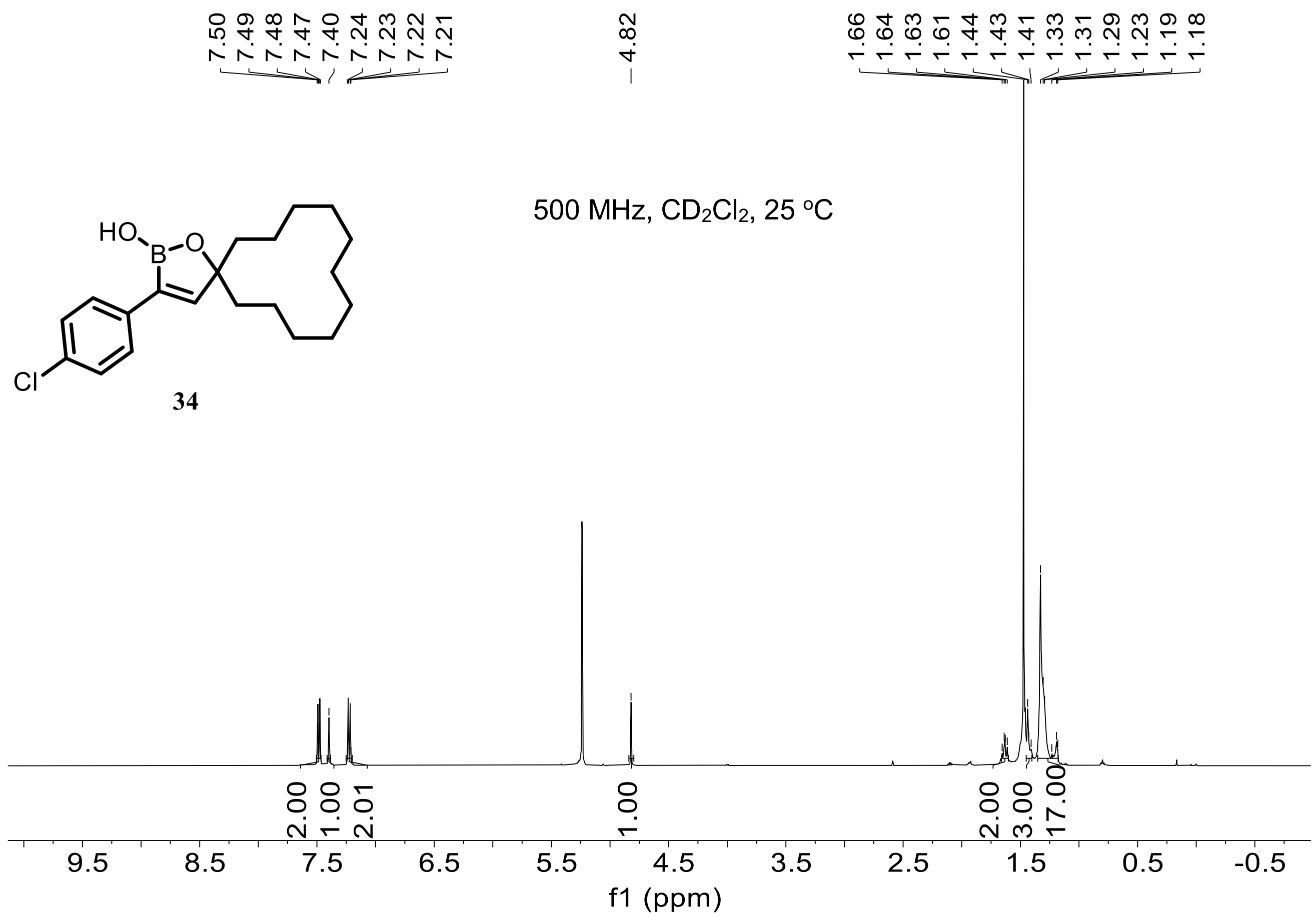
7.50 7.49 7.48 7.47 7.40 7.24 7.23 7.22 7.21
4.82
1.66 1.64 1.63 1.61 1.44 1.43 1.41 1.33 1.31 1.29 1.23 1.19 1.18
HO
B
O
Cl
34
500 MHz, CD2Cl2, 25 °C
2.00 1.00 2.01
1.00
2.00 3.00 17.00
9.5 8.5 7.5 6.5 5.5 4.5 3.5 2.5 1.5 0.5 -0.5
f1 (ppm)

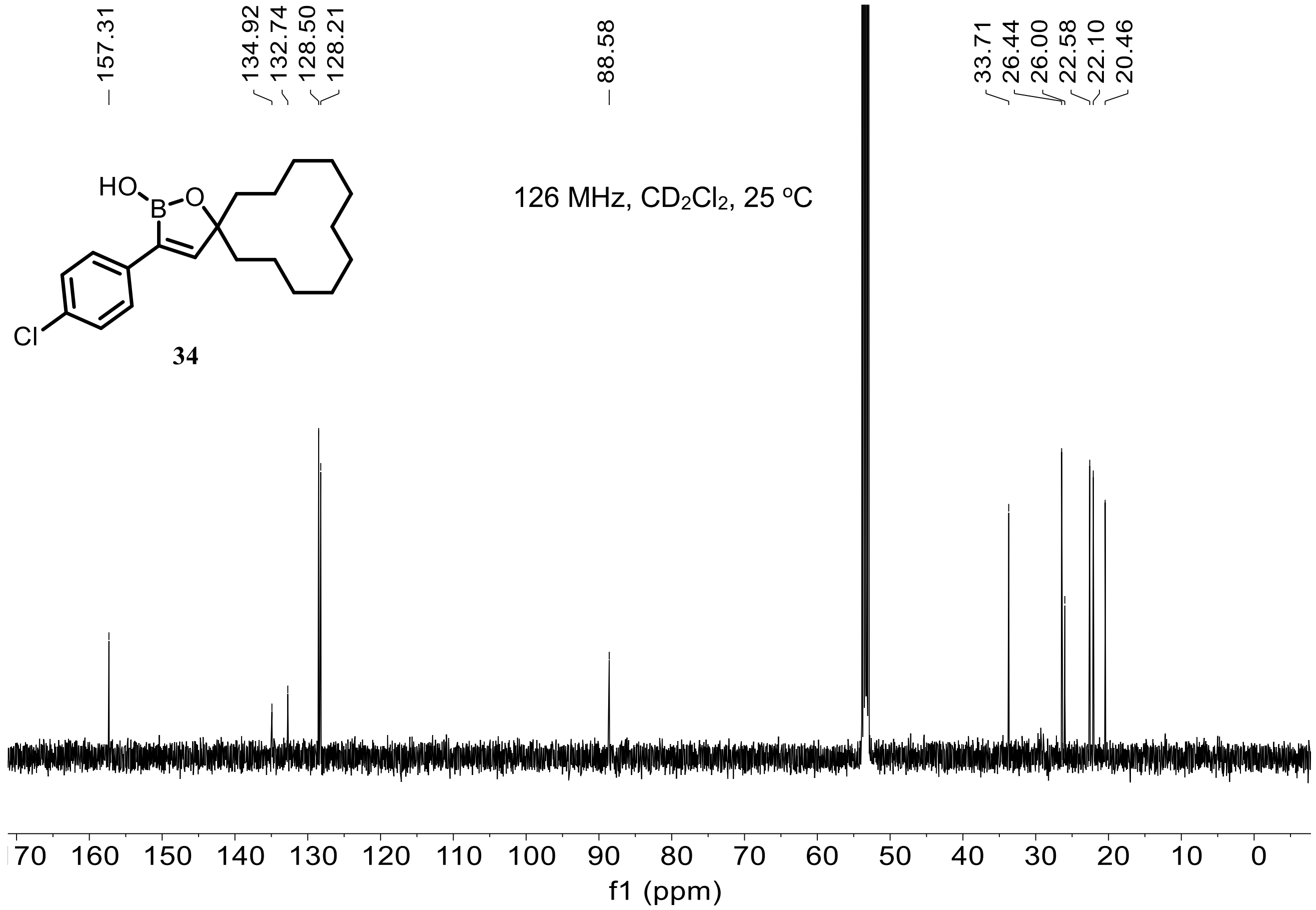
157.31
134.92 132.74 128.50 128.21
88.58
33.71 26.44 26.00 22.58 22.10 20.46
HO
B
O
Cl
34
126 MHz, CD2Cl2, 25 °C
70 160 150 140 130 120 110 100 90 80 70 60 50 40 30 20 10 0
f1 (ppm)

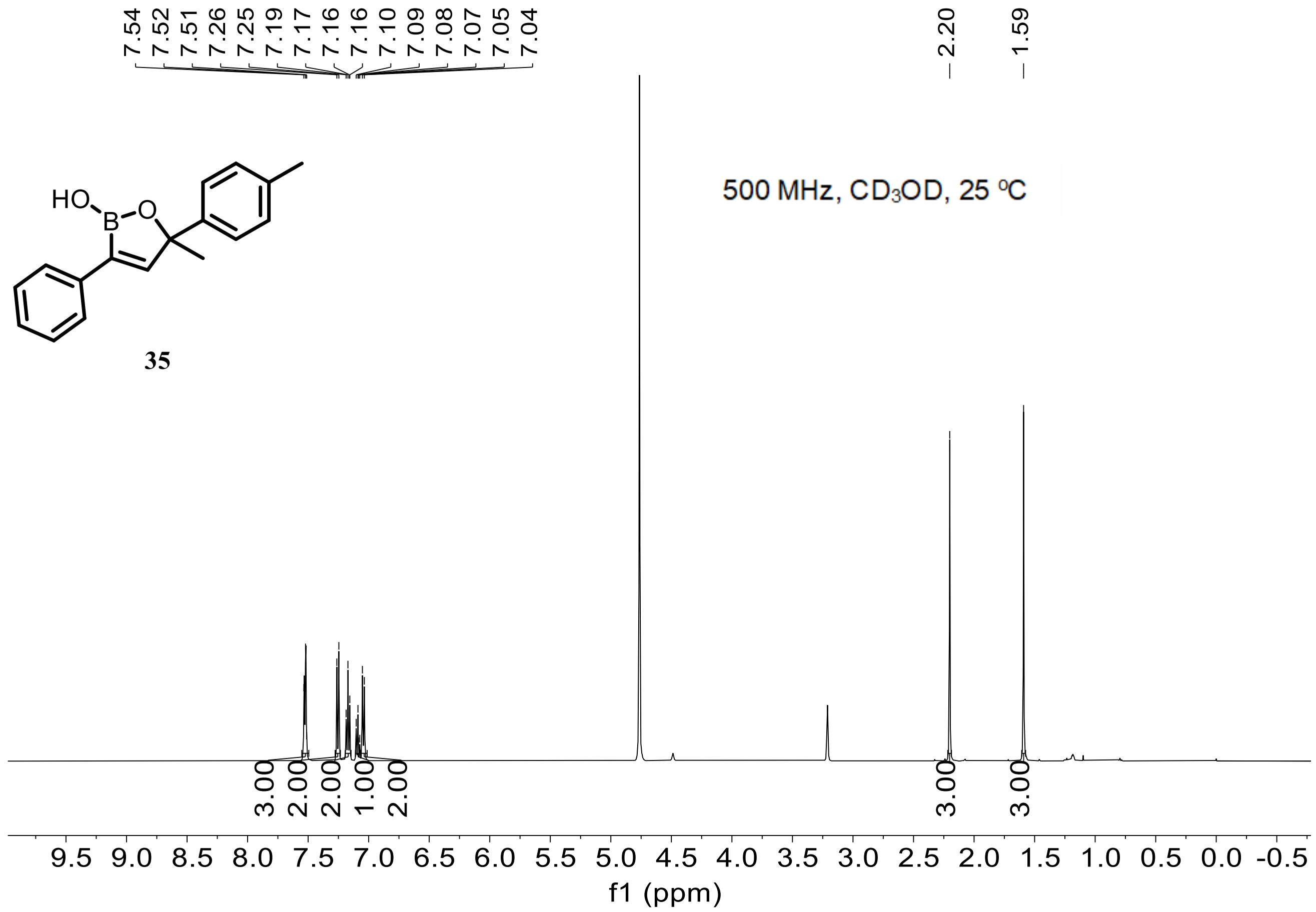
7.54 7.52 7.51 7.26 7.25 7.19 7.17 7.16 7.16 7.10 7.09 7.08 7.07 7.05 7.04
2.20
1.59
HO
B
O
35
500 MHz, CD3OD, 25 °C
3.00 2.00 2.00 1.00 2.00
3.00
3.00
f1 (ppm)

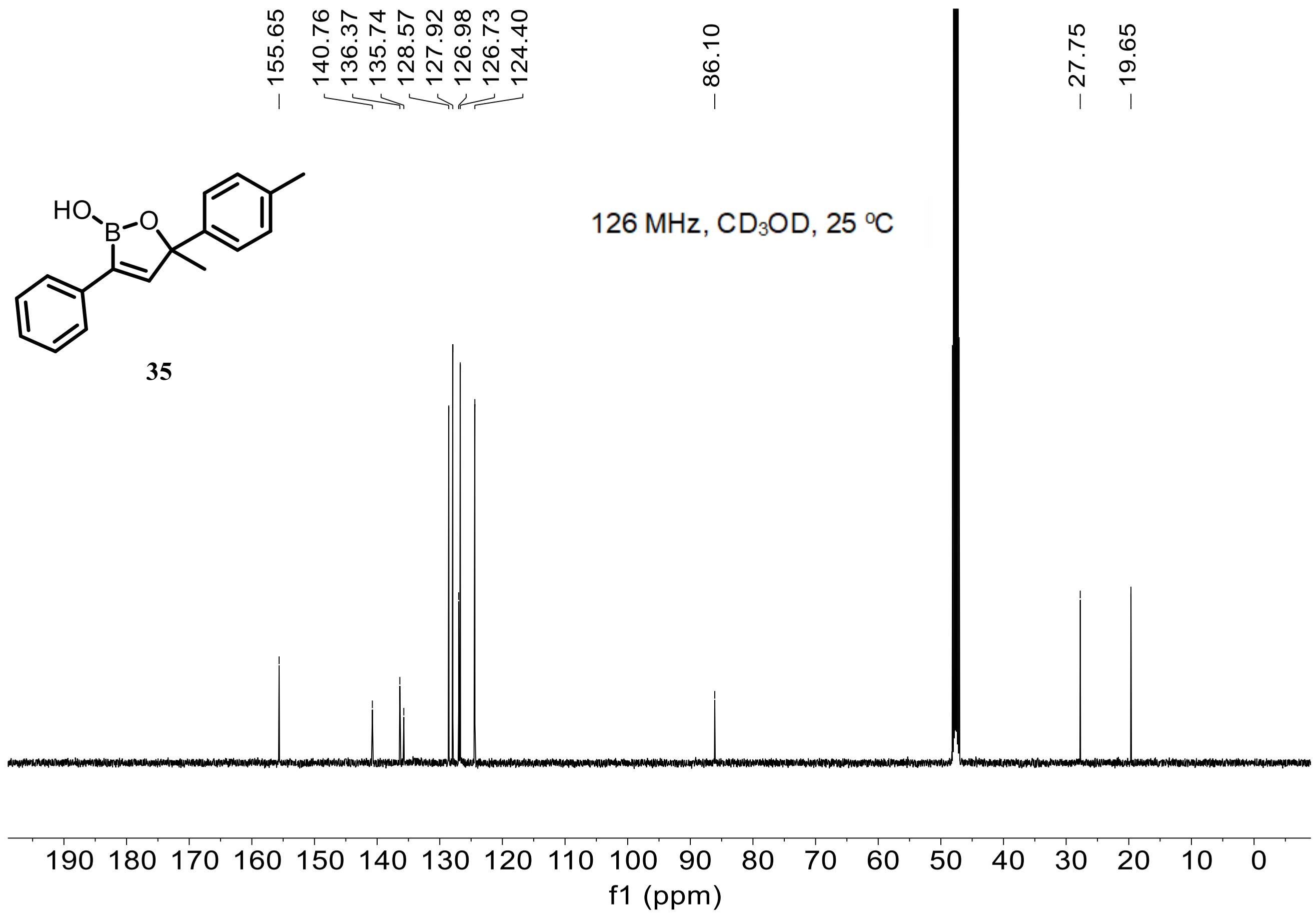
155.65
140.76 136.37 135.74 128.57 127.92 126.98 126.73 124.40
86.10
27.75
19.65
HO
B
O
35
126 MHz, CD3OD, 25 °C
f1 (ppm)

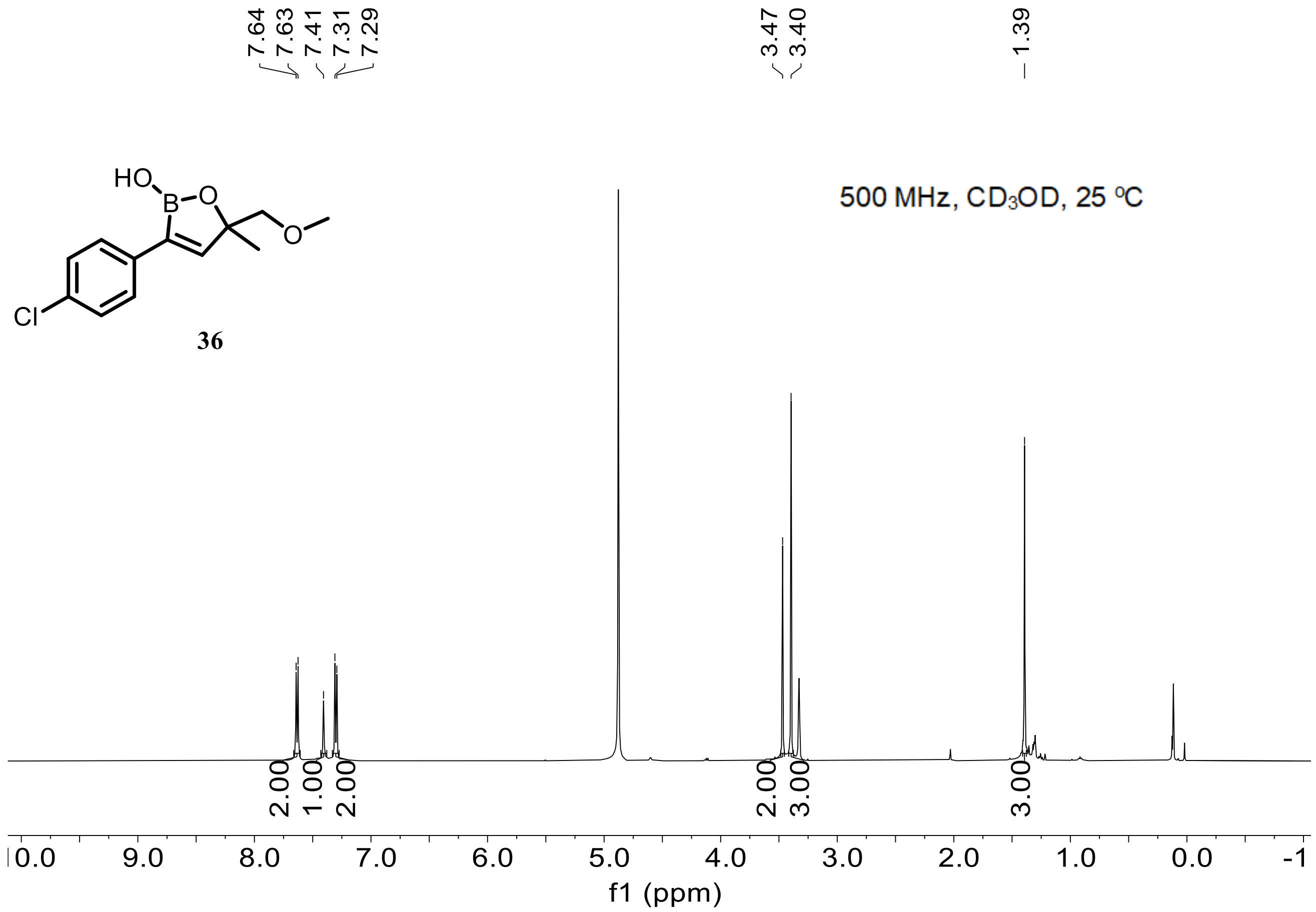
7.64
7.63
7.41
7.31
7.29
3.47
3.40
1.39
HO
B
O
O
Cl
36
500 MHz, CD3OD, 25 °C
2.00
1.00
2.00
2.00
3.00
3.00
f1 (ppm)

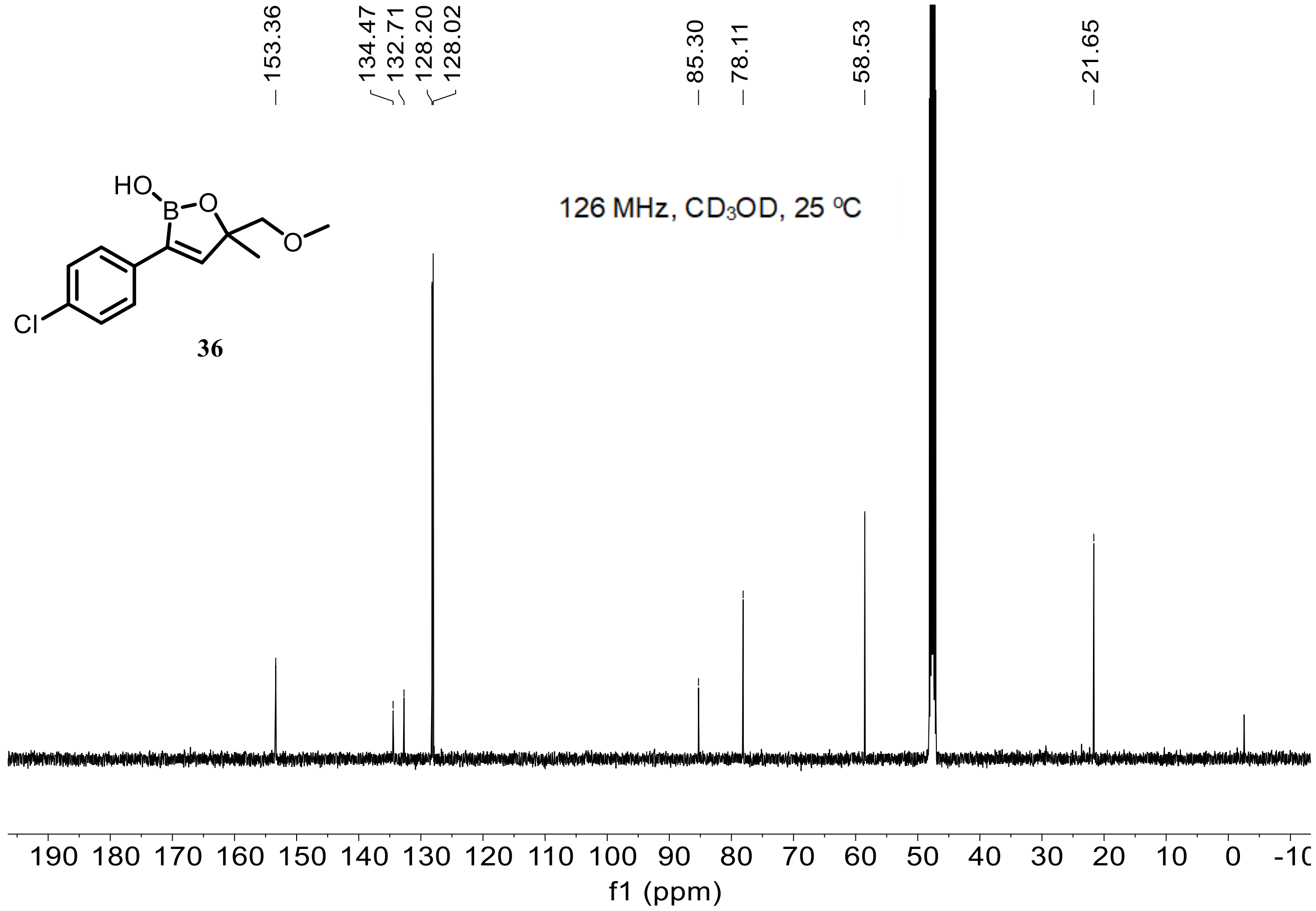
153.36
134.47
132.71
128.20
128.02
85.30
78.11
58.53
21.65
HO
B
O
O
Cl
36
126 MHz, CD3OD, 25 °C
f1 (ppm)

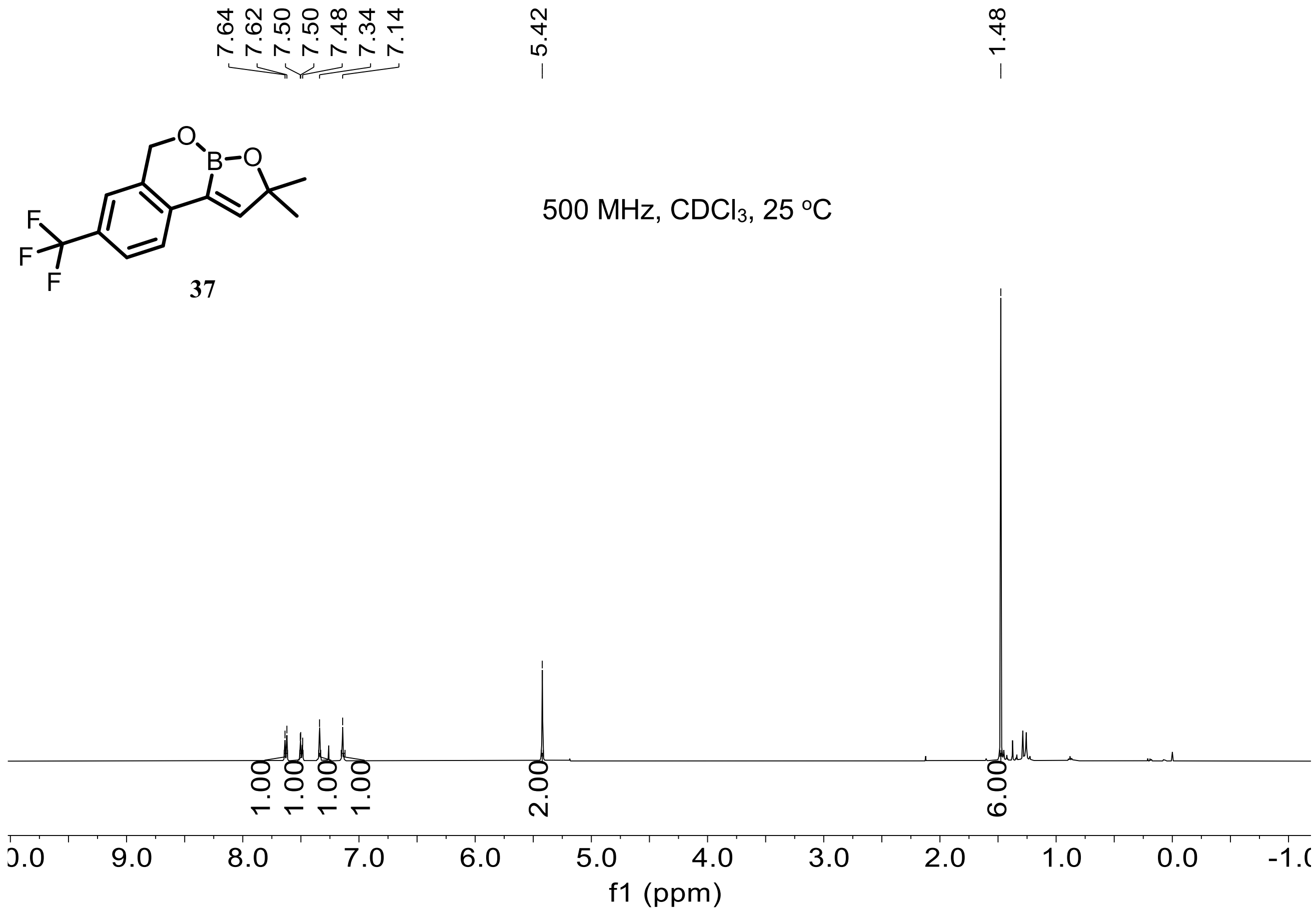
7.64
7.62
7.50
7.50
7.48
7.34
7.14
5.42
1.48
F
F
F
O
B
O
37
500 MHz, CDCl3, 25 °C
1.00
1.00
1.00
1.00
2.00
6.00
9.0 8.0 7.0 6.0 5.0 4.0 3.0 2.0 1.0 0.0
f1 (ppm)

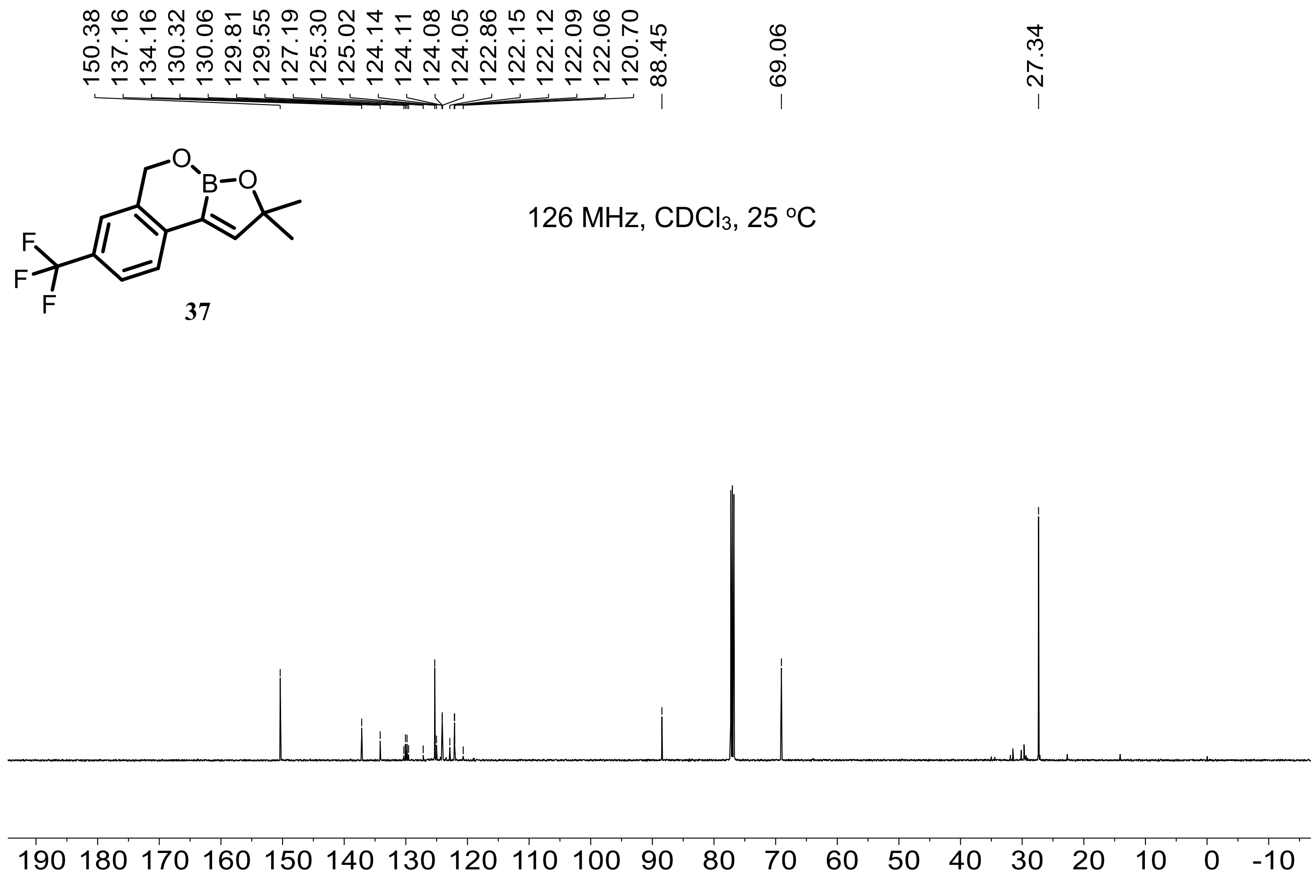
150.38
137.16
134.16
130.32
130.06
129.81
129.55
127.19
125.30
125.02
124.14
124.11
124.08
124.05
122.86
122.15
122.12
122.09
122.06
120.70
88.45
69.06
27.34
F
F
F
O
B
O
37
126 MHz, CDCl3, 25 °C
190 180 170 160 150 140 130 120 110 100 90 80 70 60 50 40 30 20 10 0 -10
f1 (ppm)

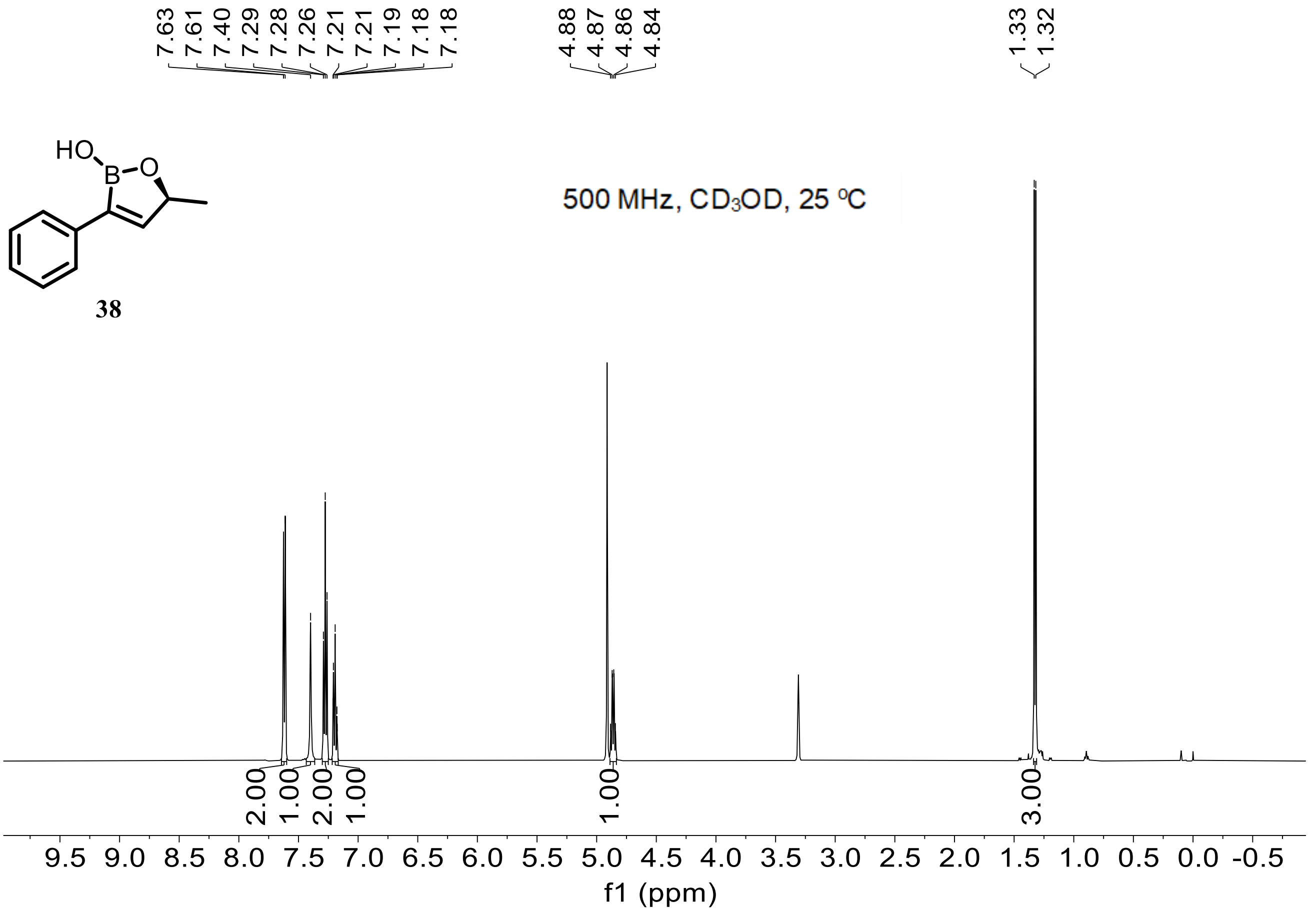
7.63
7.61
7.40
7.29
7.28
7.26
7.21
7.21
7.19
7.18
7.18
4.88
4.87
4.86
4.84
1.33
1.32
HO
B
O
38
500 MHz, CD3OD, 25 °C
2.00
1.00
2.00
1.00
1.00
3.00
9.5 9.0 8.5 8.0 7.5 7.0 6.5 6.0 5.5 5.0 4.5 4.0 3.5 3.0 2.5 2.0 1.5 1.0 0.5 0.0 -0.5
f1 (ppm)

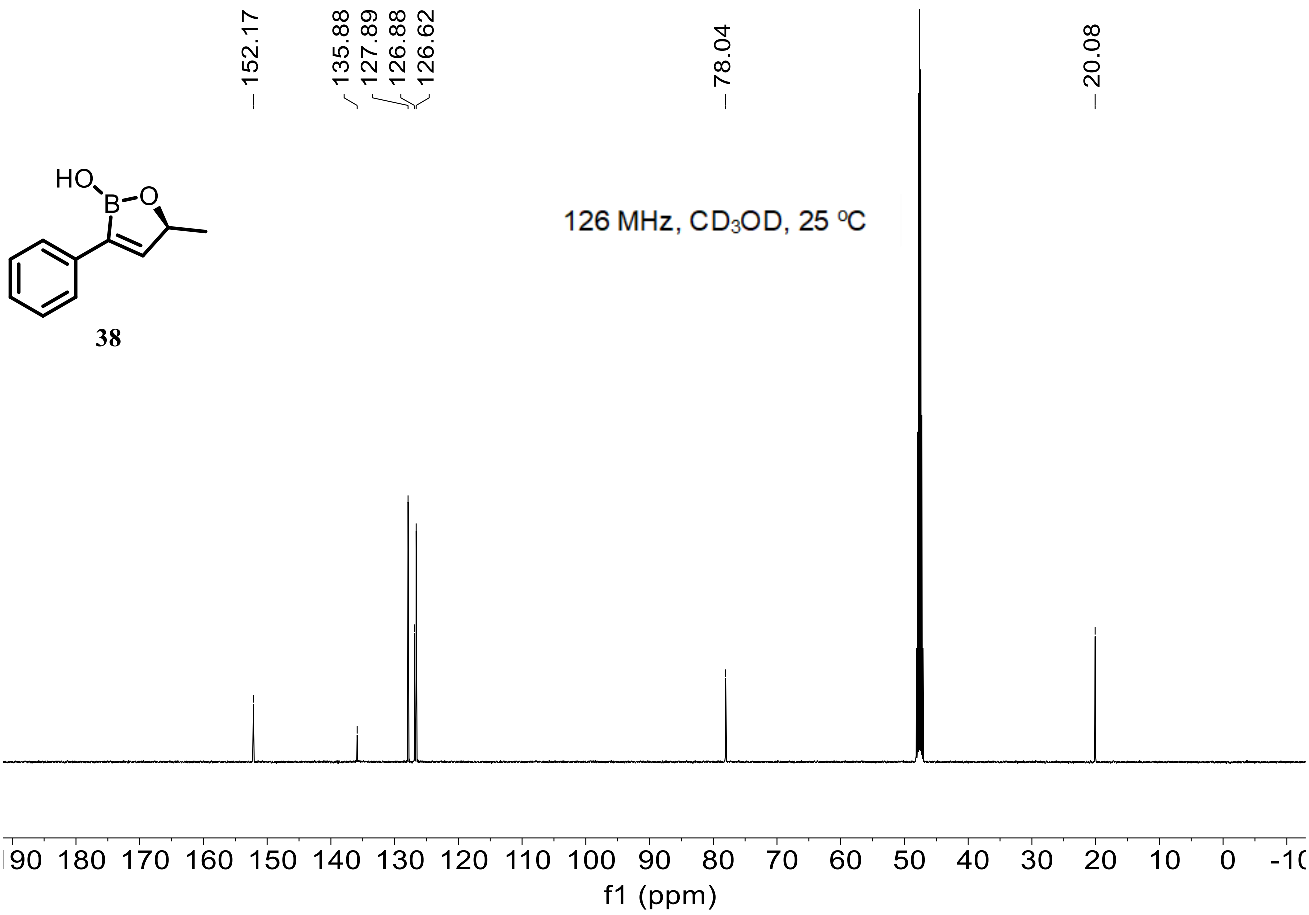
152.17
135.88
127.89
126.88
126.62
78.04
20.08
HO
B
O
38
126 MHz, CD3OD, 25 °C
90 180 170 160 150 140 130 120 110 100 90 80 70 60 50 40 30 20 10 0
f1 (ppm)

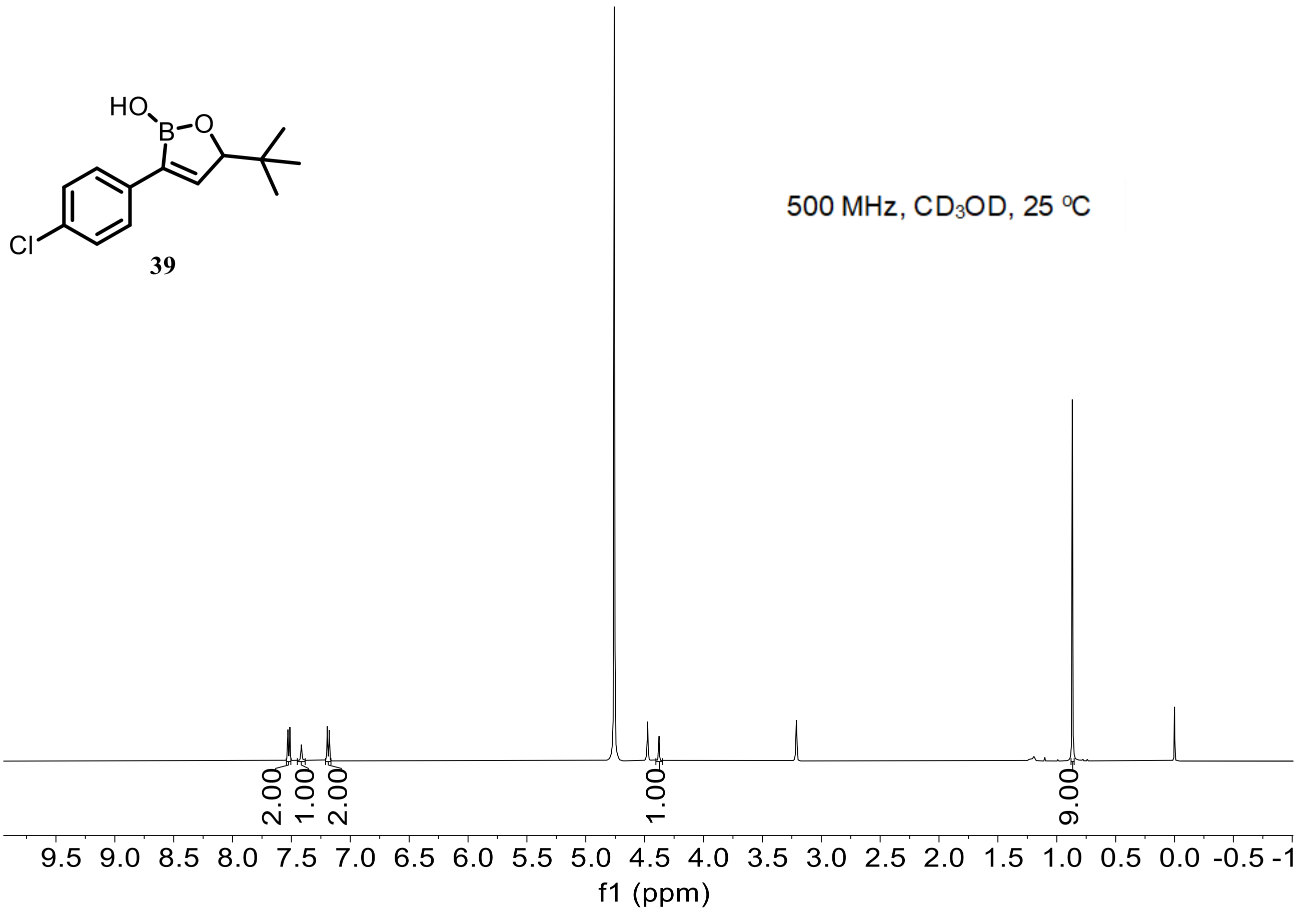
HO
B
O
Cl
500 MHz, CD3OD, 25 °C
2.00
1.00
2.00
1.00
9.00
9.5 9.0 8.5 8.0 7.5 7.0 6.5 6.0 5.5 5.0 4.5 4.0 3.5 3.0 2.5 2.0 1.5 1.0 0.5 0.0 -0.5 -1
f1 (ppm)

39

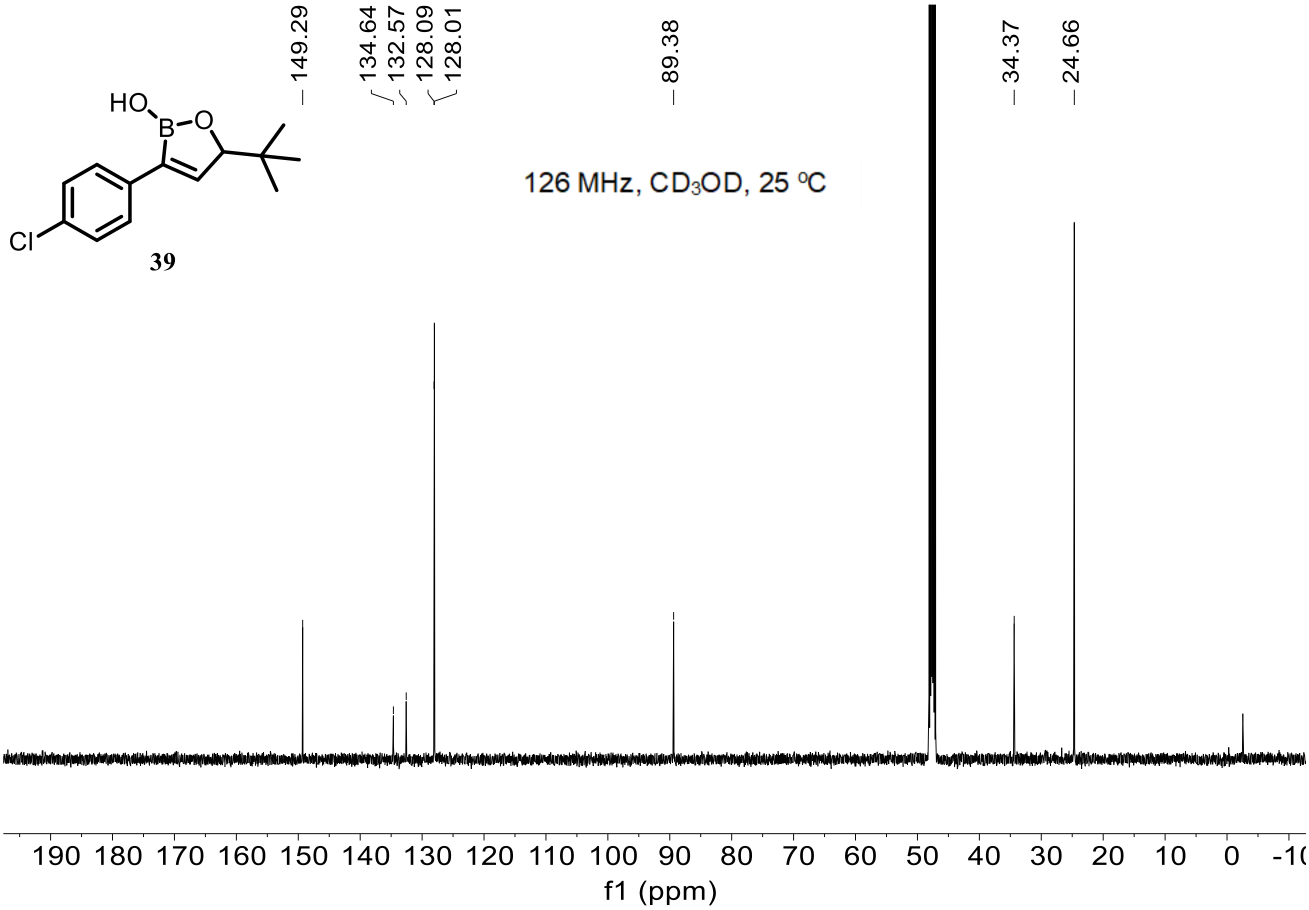
149.29
134.64
132.57
128.09
128.01
89.38
34.37
24.66
HO
B
O
Cl
126 MHz, CD3OD, 25 °C
190 180 170 160 150 140 130 120 110 100 90 80 70 60 50 40 30 20 10 0 -10
f1 (ppm)

39

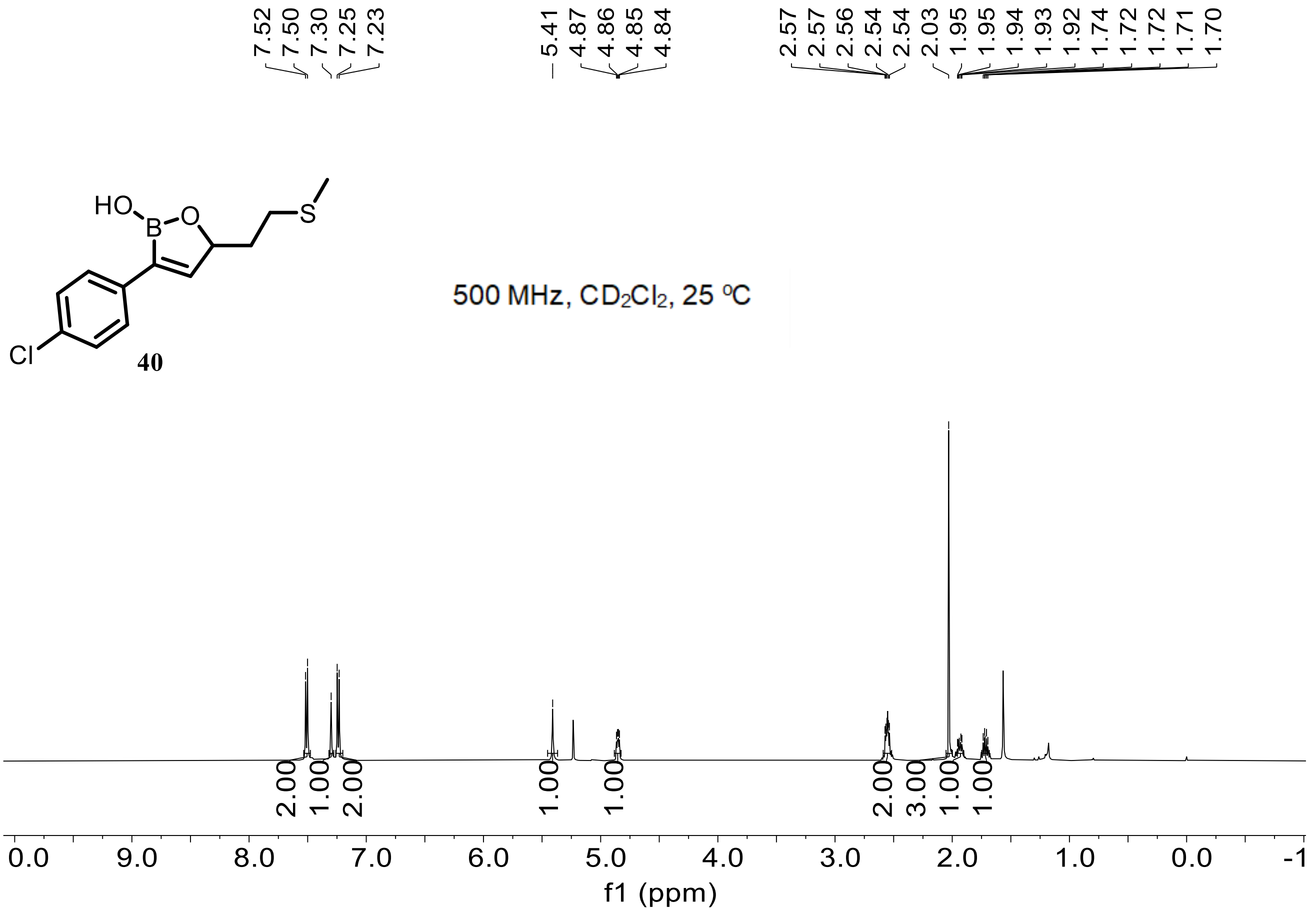
7.52 7.50 7.30 7.25 7.23
5.41 4.87 4.86 4.85 4.84
2.57 2.57 2.56 2.54 2.54 2.03 1.95 1.95 1.94 1.93 1.92 1.74 1.72 1.72 1.71 1.70
HO B O S Cl
40
500 MHz, CD2Cl2, 25 °C
2.00 1.00 2.00 1.00 1.00 2.00 3.00 1.00 1.00
0.0 9.0 8.0 7.0 6.0 5.0 4.0 3.0 2.0 1.0 0.0 -1
f1 (ppm)

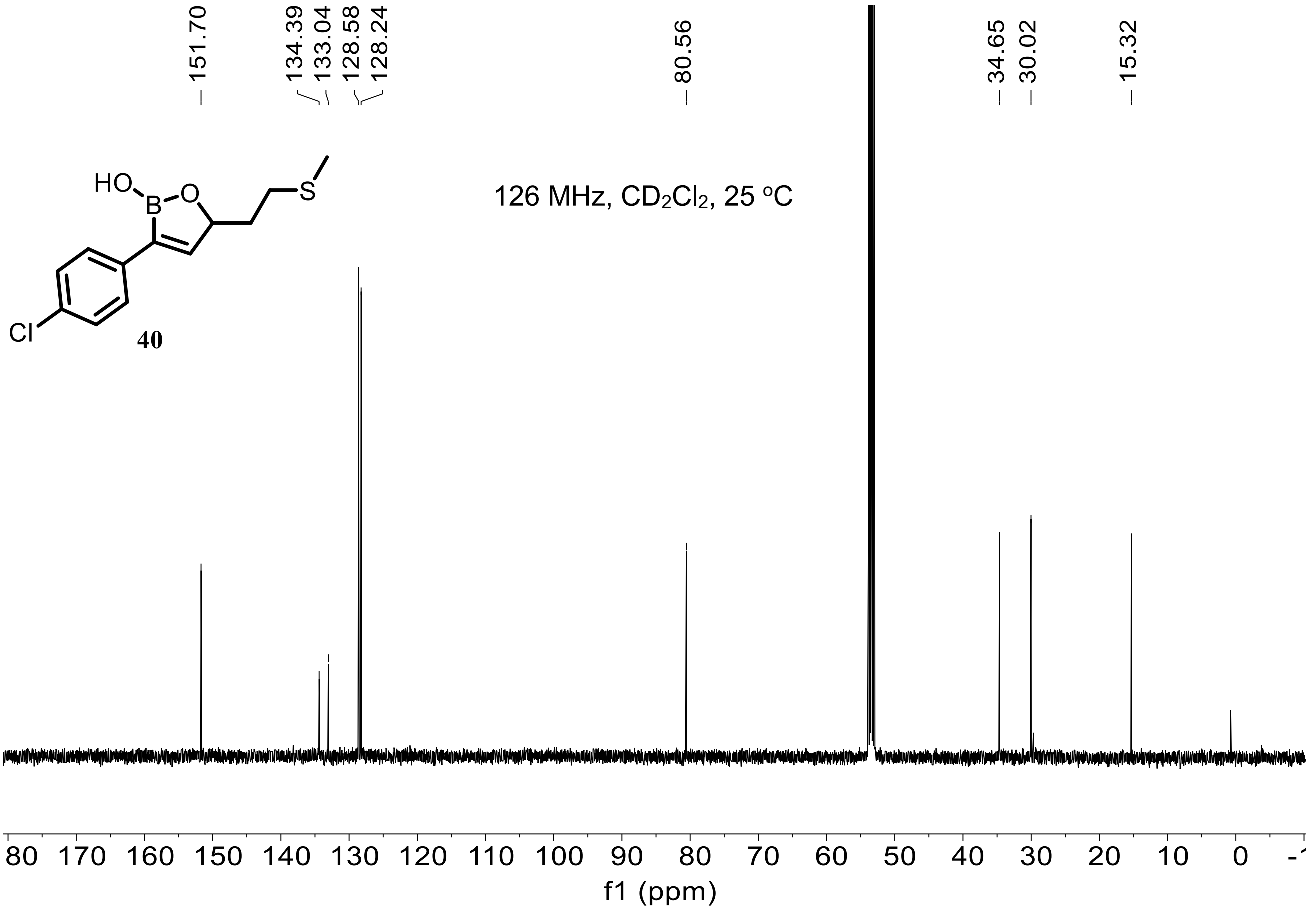
151.70
134.39 133.04 128.58 128.24
80.56
34.65 30.02
15.32
HO B O S Cl
40
126 MHz, CD2Cl2, 25 °C
80 170 160 150 140 130 120 110 100 90 80 70 60 50 40 30 20 10 0
f1 (ppm)

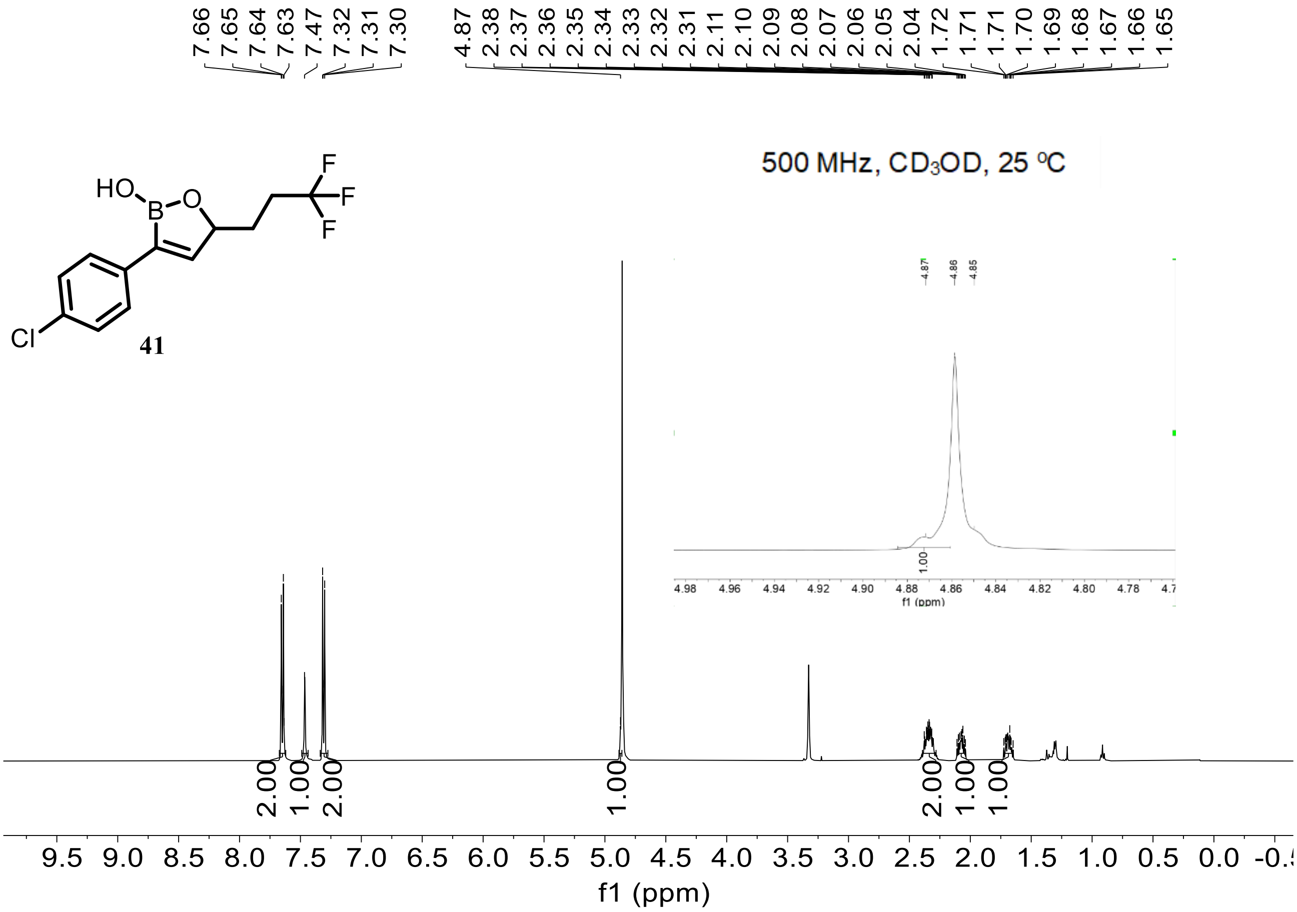
7.66 7.65 7.64 7.63 7.47 7.32 7.31 7.30
4.87 2.38 2.37 2.36 2.35 2.34 2.33 2.32 2.31 2.11 2.10 2.09 2.08 2.07 2.06 2.05 2.04 1.72 1.71 1.71 1.70 1.69 1.68 1.67 1.66 1.65
500 MHz, CD3OD, 25 °C
HO
B
O
F
F
F
Cl
41
2.00 1.00 2.00
1.00
2.00 1.00 1.00
f1 (ppm)

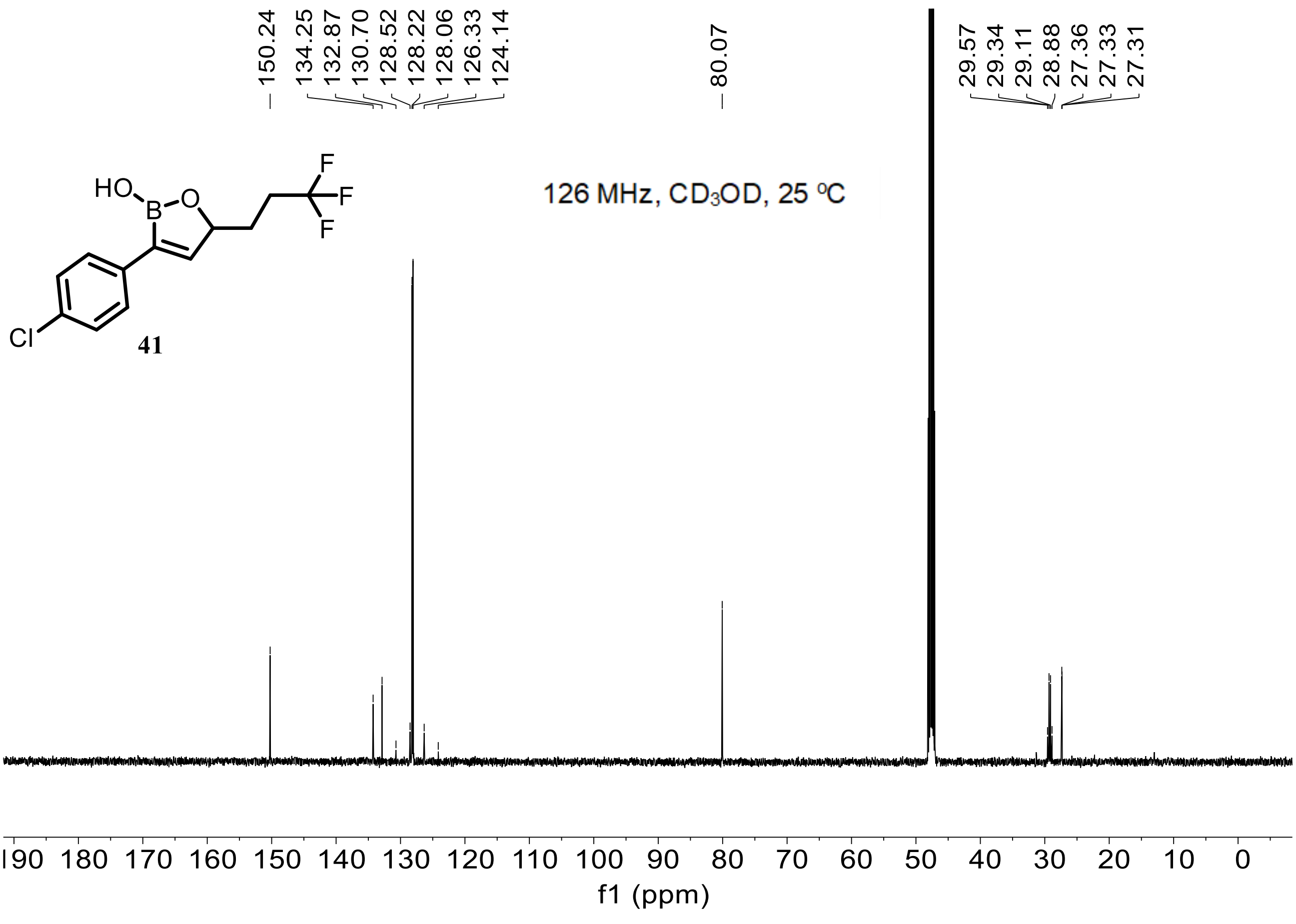
150.24
134.25 132.87 130.70 128.52 128.22 128.06 126.33 124.14
80.07
29.57 29.34 29.11 28.88 27.36 27.33 27.31
126 MHz, CD3OD, 25 °C
HO
B
O
F
F
F
Cl
41
f1 (ppm)

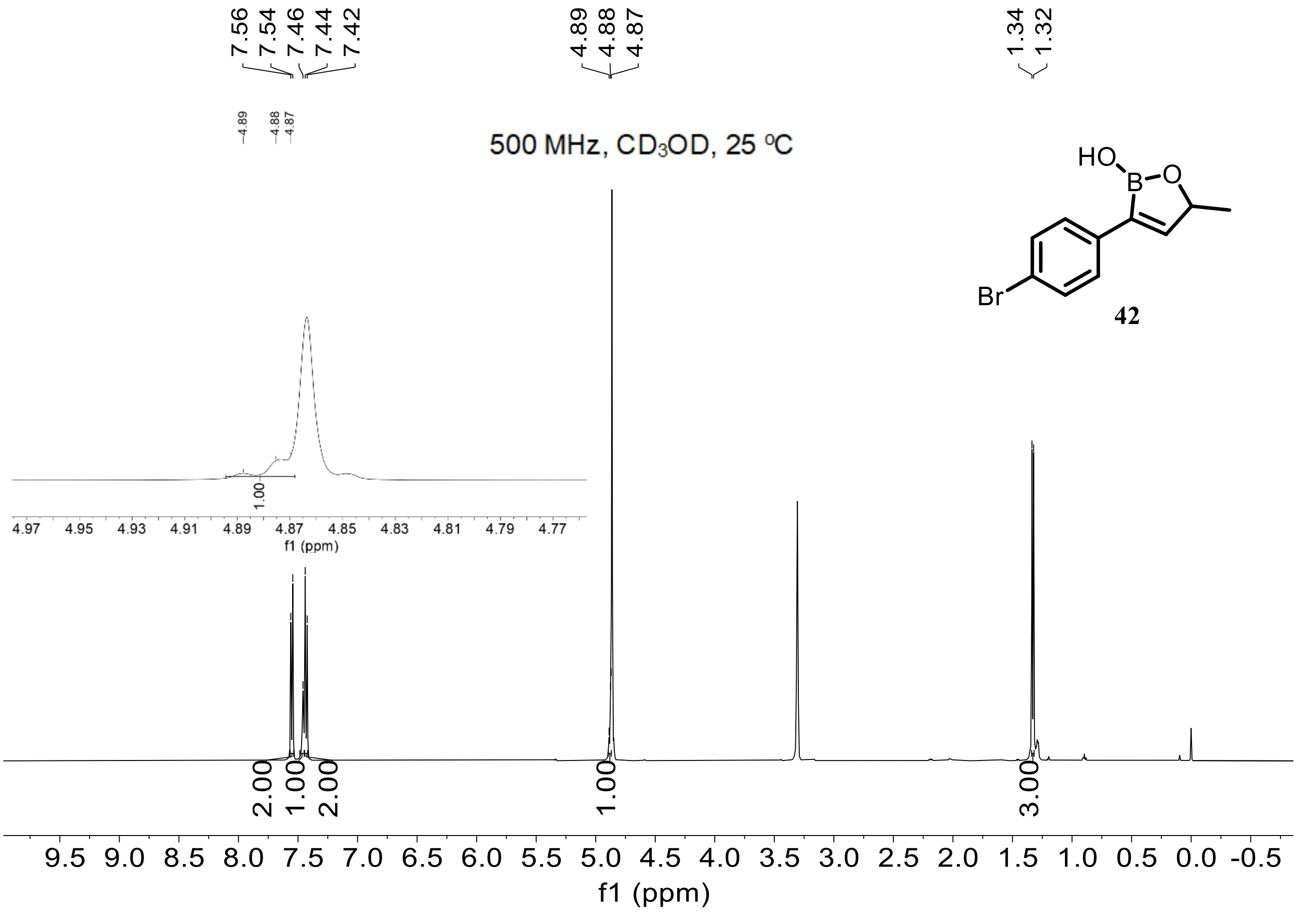
7.56
7.54
7.46
7.44
7.42
4.89
4.88
4.87
1.34
1.32
500 MHz, CD3OD, 25 °C
HO
B
O
Br
42
2.00
1.00
2.00
1.00
3.00
9.5 9.0 8.5 8.0 7.5 7.0 6.5 6.0 5.5 5.0 4.5 4.0 3.5 3.0 2.5 2.0 1.5 1.0 0.5 0.0 -0.5
f1 (ppm)

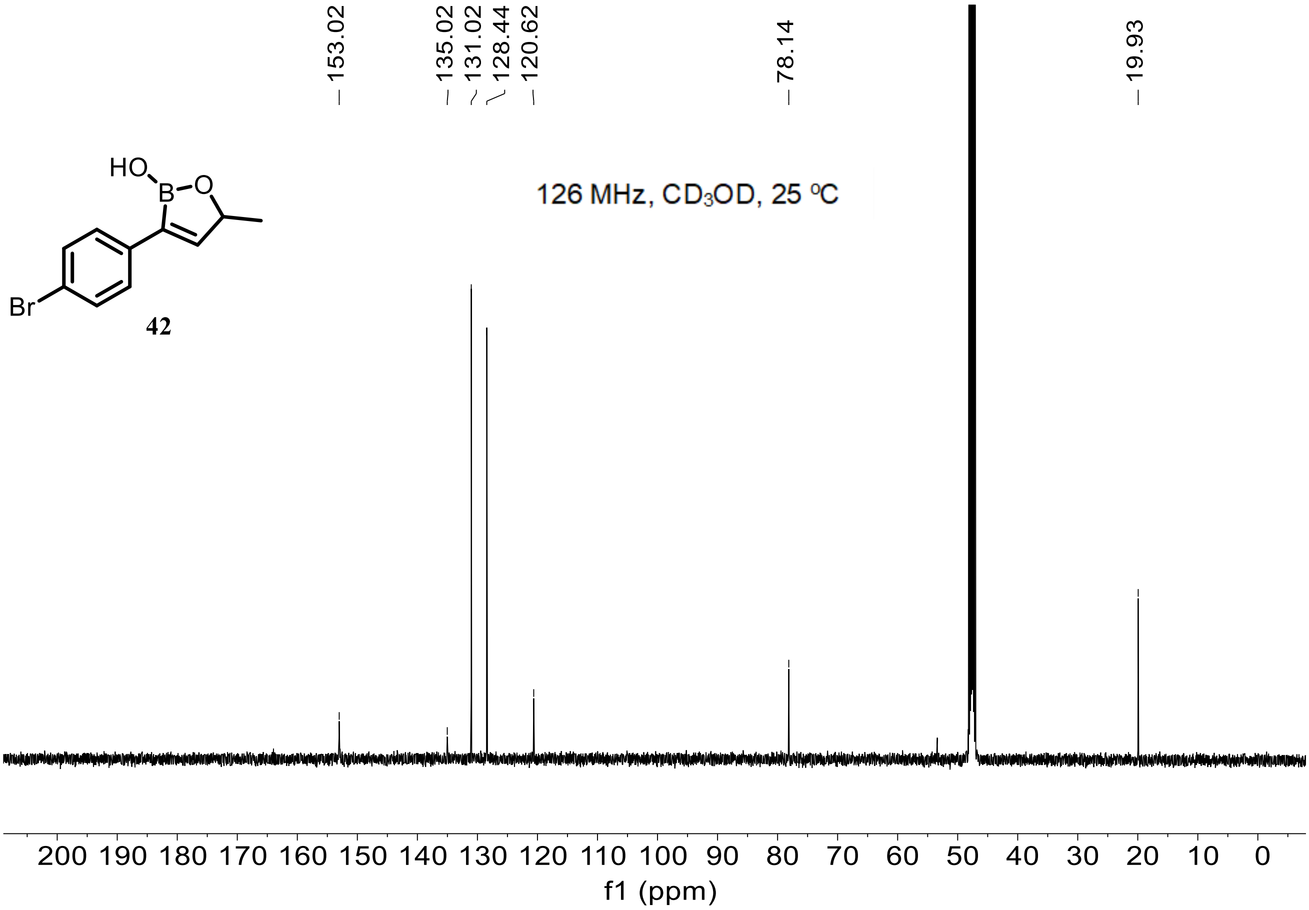
153.02
135.02
131.02
128.44
120.62
78.14
19.93
126 MHz, CD3OD, 25 °C
HO
B
O
Br
42
200 190 180 170 160 150 140 130 120 110 100 90 80 70 60 50 40 30 20 10 0
f1 (ppm)

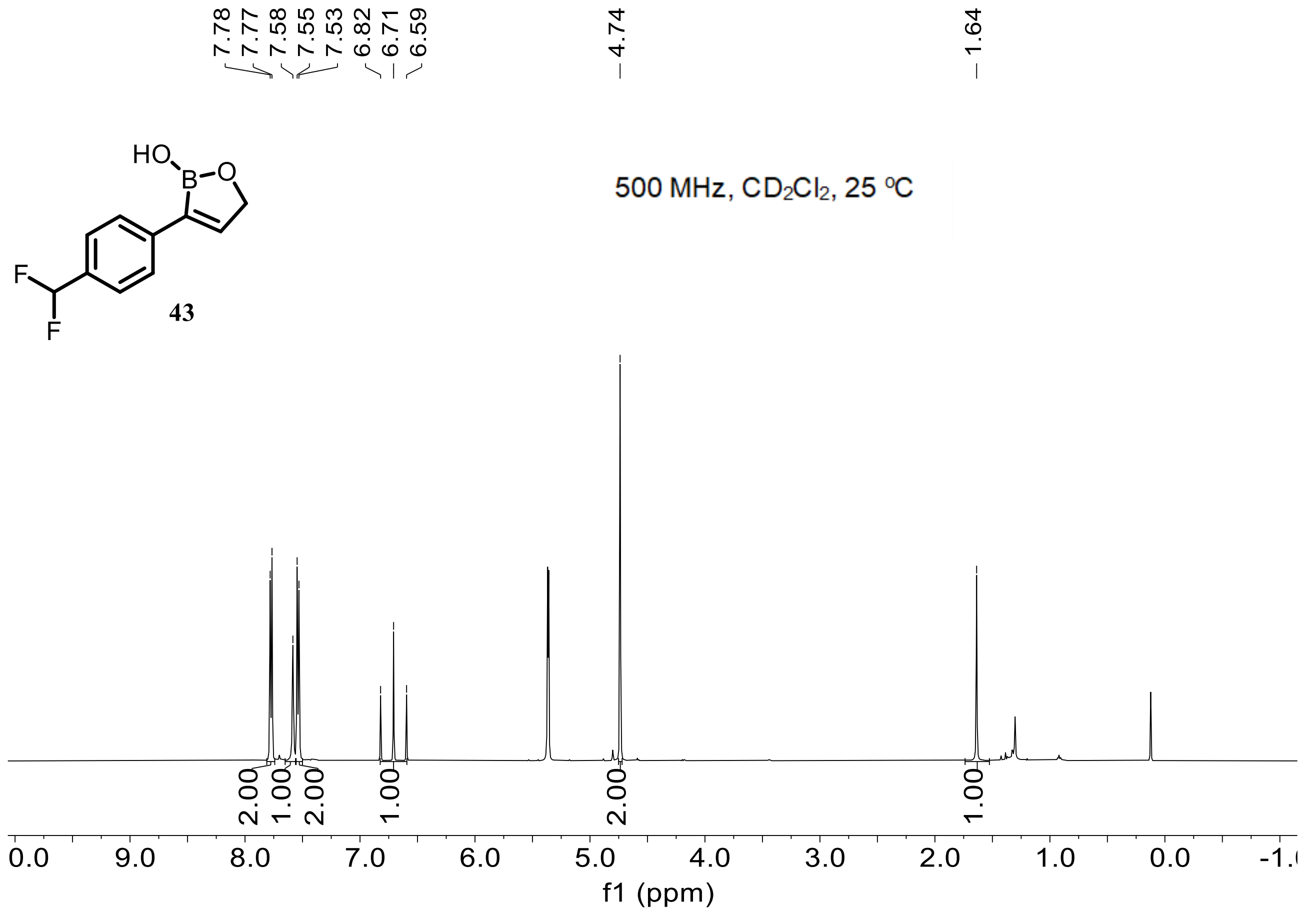
7.78
7.77
7.58
7.55
7.53
6.82
6.71
6.59
4.74
1.64
HO
B
O
F
F
43
500 MHz, CD_2Cl_2, 25 ºC
2.00
1.00
2.00
1.00
2.00
1.00
0.0 9.0 8.0 7.0 6.0 5.0 4.0 3.0 2.0 1.0 0.0
f1 (ppm)

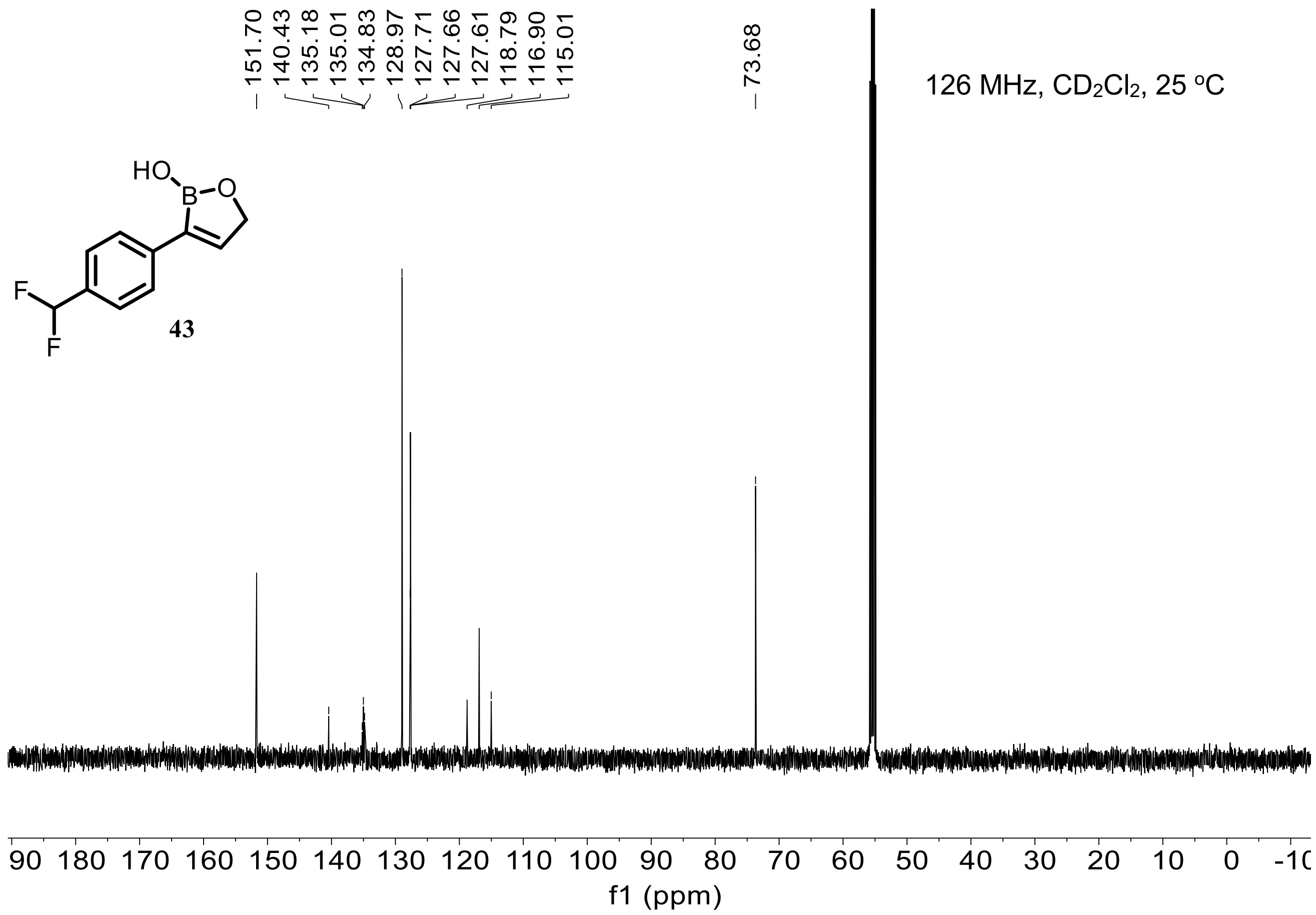
151.70
140.43
135.18
135.01
134.83
128.97
127.71
127.66
127.61
118.79
116.90
115.01
73.68
126 MHz, CD_2Cl_2, 25 ºC
HO
B
O
F
F
43
90 180 170 160 150 140 130 120 110 100 90 80 70 60 50 40 30 20 10 0
f1 (ppm)

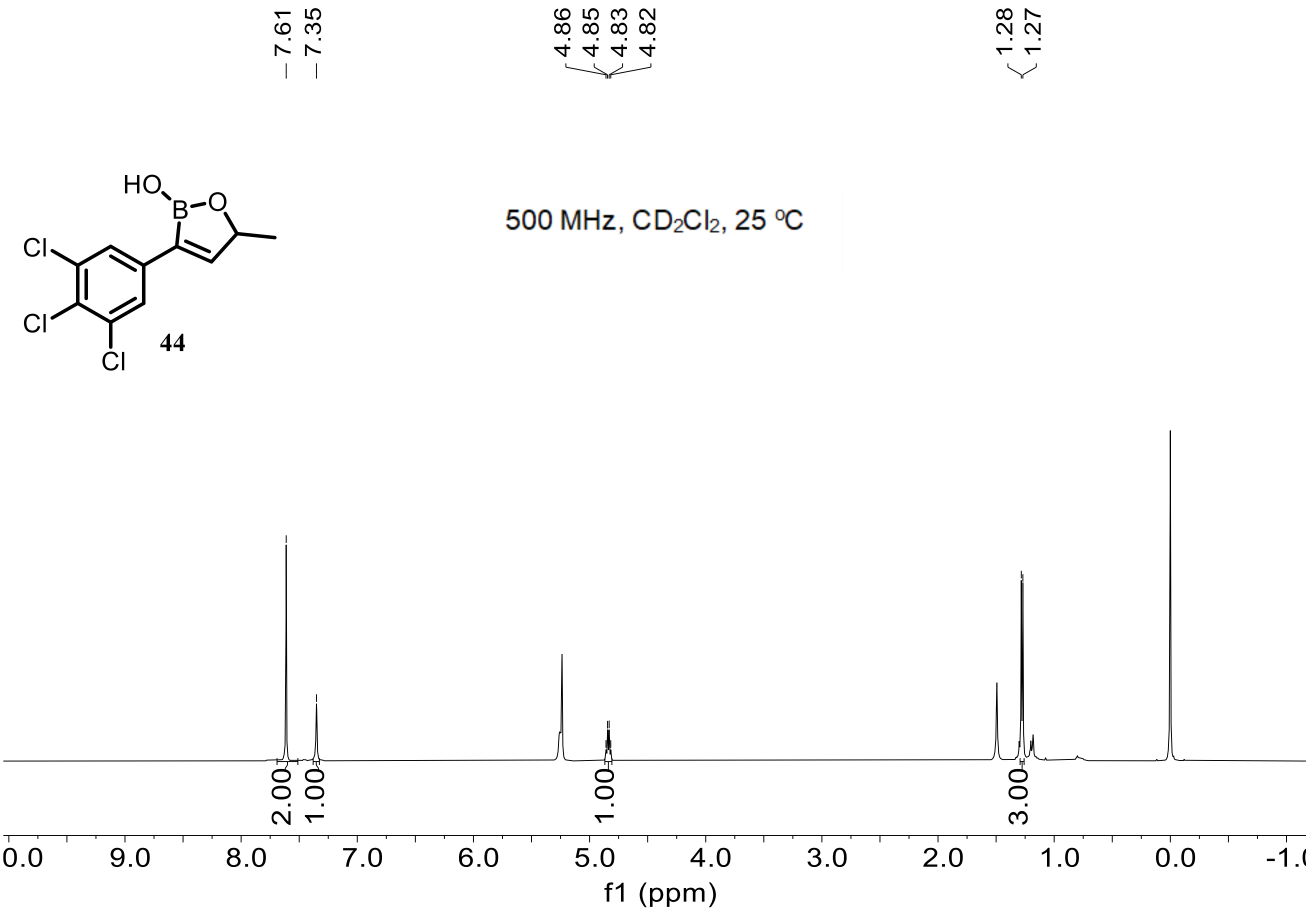

7.61
7.35
4.86
4.85
4.83
4.82
1.28
1.27
HO
B
O
Cl
Cl
Cl
44
500 MHz, CD2Cl2, 25 ºC
2.00
1.00
1.00
3.00
f1 (ppm)

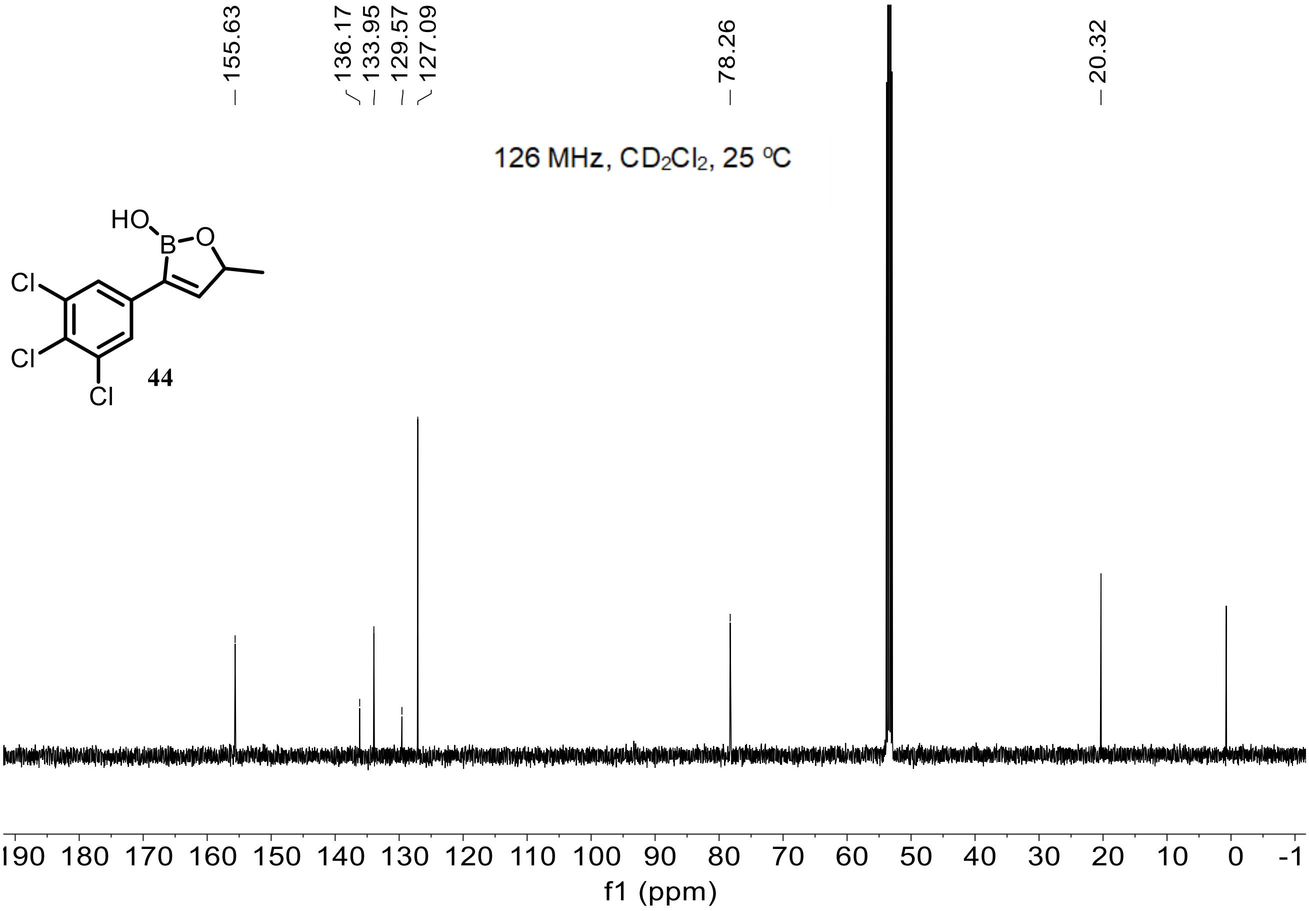

155.63
136.17
133.95
129.57
127.09
78.26
20.32
126 MHz, CD2Cl2, 25 ºC
HO
B
O
Cl
Cl
Cl
44
f1 (ppm)

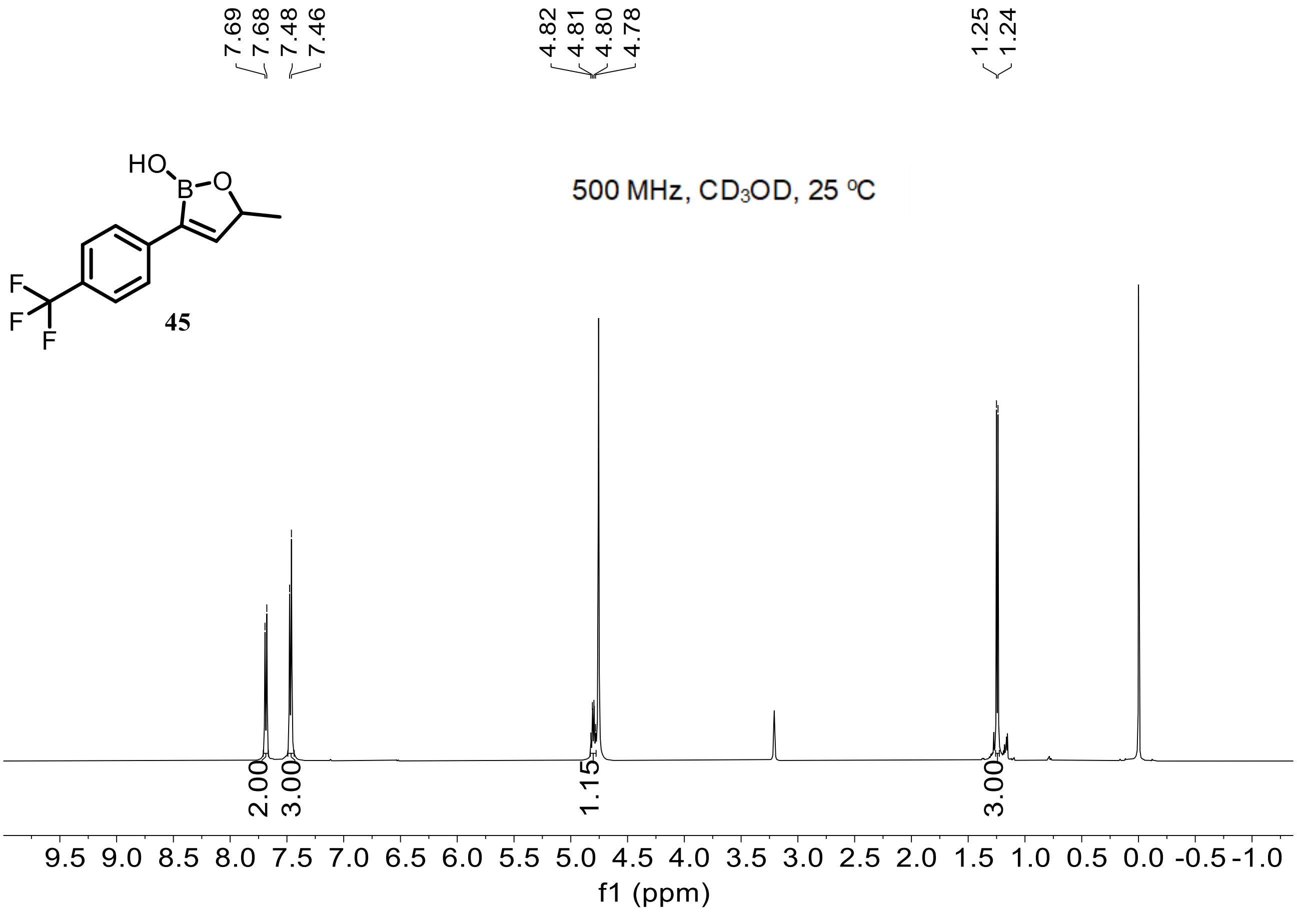
7.69
7.68
7.48
7.46
4.82
4.81
4.80
4.78
1.25
1.24
HO
B
O
F
F
F
45
500 MHz, CD3OD, 25 °C
2.00
3.00
1.15
3.00
9.5 9.0 8.5 8.0 7.5 7.0 6.5 6.0 5.5 5.0 4.5 4.0 3.5 3.0 2.5 2.0 1.5 1.0 0.5 0.0 -0.5 -1.0
f1 (ppm)

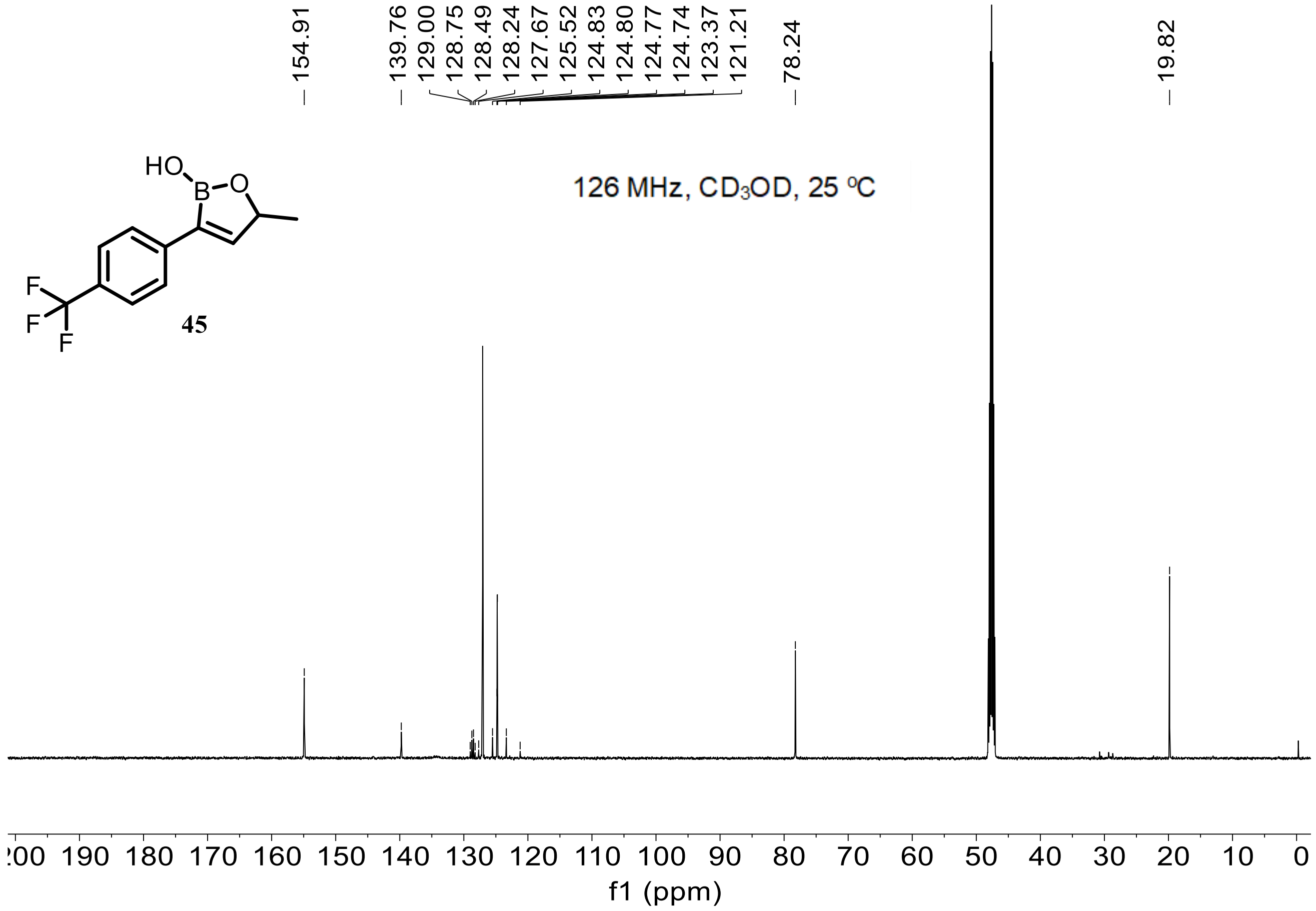
154.91
139.76
129.00
128.75
128.49
128.24
127.67
125.52
124.83
124.80
124.77
124.74
123.37
121.21
78.24
19.82
HO
B
O
F
F
F
45
126 MHz, CD3OD, 25 °C
200 190 180 170 160 150 140 130 120 110 100 90 80 70 60 50 40 30 20 10 0
f1 (ppm)

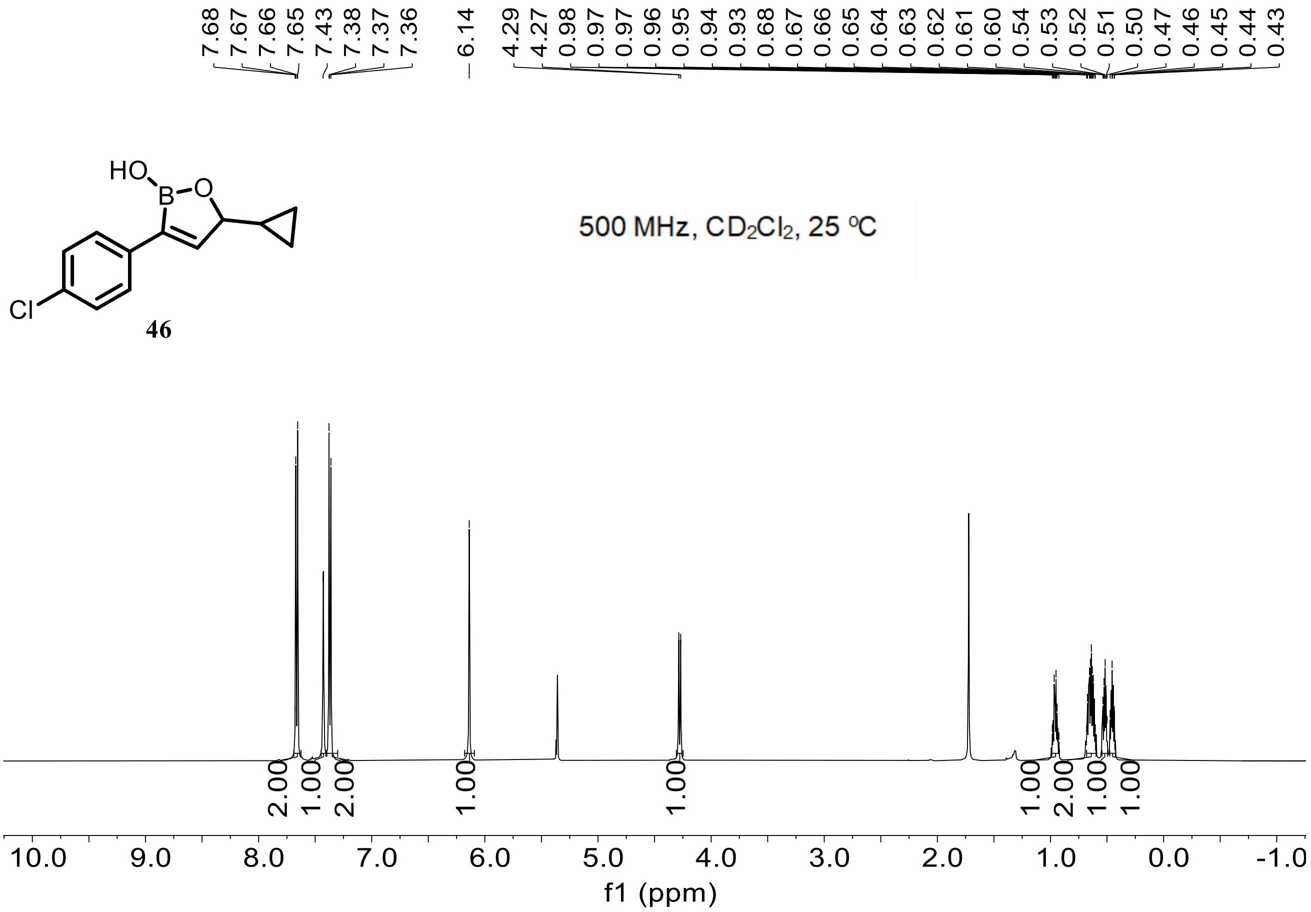
7.68 7.67 7.66 7.65 7.43 7.38 7.37 7.36 6.14 4.29 4.27 0.98 0.97 0.97 0.96 0.95 0.94 0.93 0.68 0.67 0.66 0.65 0.64 0.63 0.62 0.61 0.60 0.54 0.53 0.52 0.51 0.50 0.47 0.46 0.45 0.44 0.43
HO
B
O
Cl
46
500 MHz, CD_2Cl_2, 25 °C
2.00 1.00 2.00 1.00 1.00 1.00 2.00 1.00 1.00
10.0 9.0 8.0 7.0 6.0 5.0 4.0 3.0 2.0 1.0 0.0 -1.0
f1 (ppm)

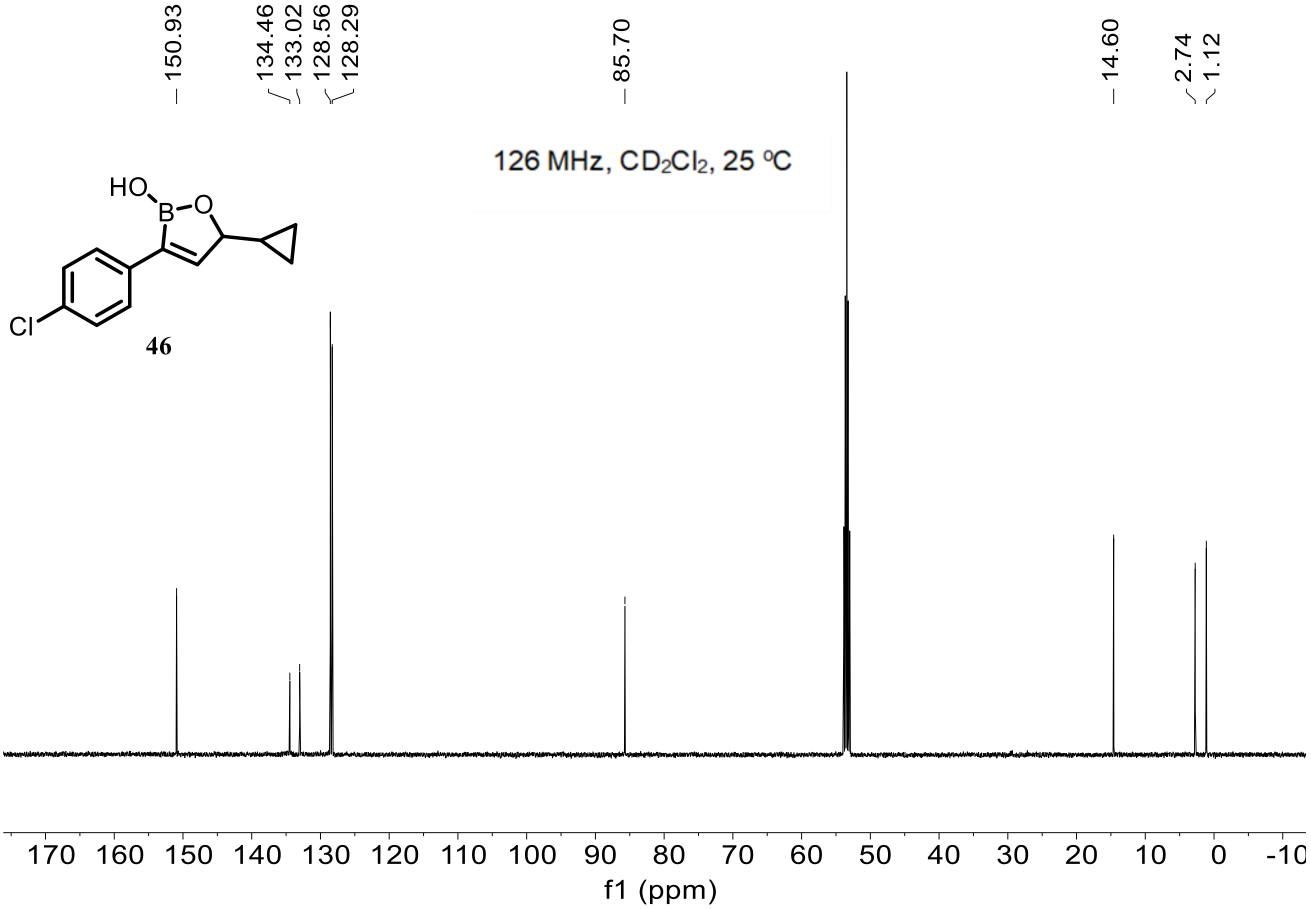
150.93 134.46 133.02 128.56 128.29 85.70 14.60 2.74 1.12
126 MHz, CD_2Cl_2, 25 °C
HO
B
O
Cl
46
170 160 150 140 130 120 110 100 90 80 70 60 50 40 30 20 10 0 -10
f1 (ppm)

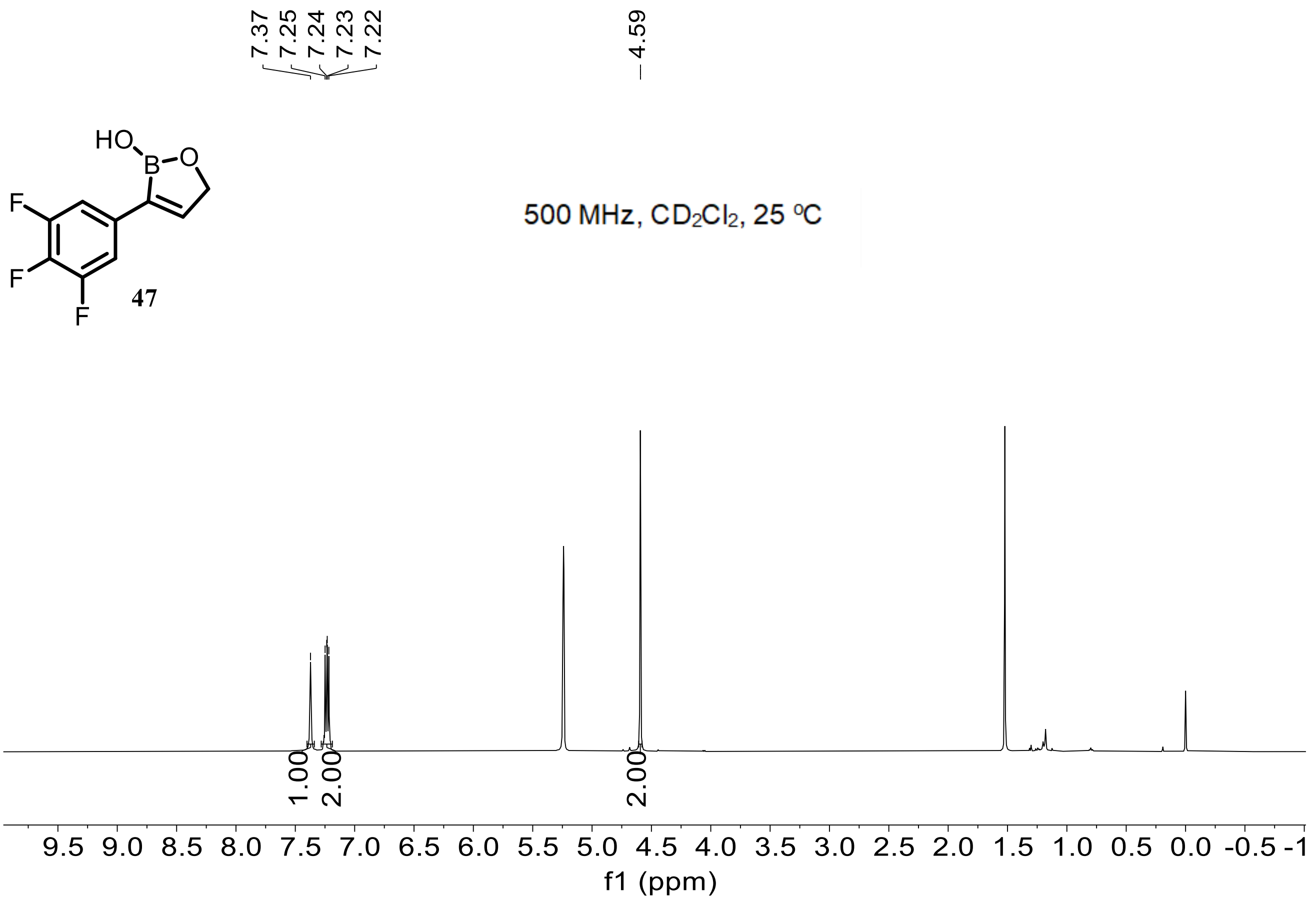
7.37
7.25
7.24
7.23
7.22
4.59
HO
B
O
F
F
F
47
500 MHz, CD_2Cl_2, 25 °C
1.00
2.00
2.00
9.5 9.0 8.5 8.0 7.5 7.0 6.5 6.0 5.5 5.0 4.5 4.0 3.5 3.0 2.5 2.0 1.5 1.0 0.5 0.0 -0.5 -1
f1 (ppm)

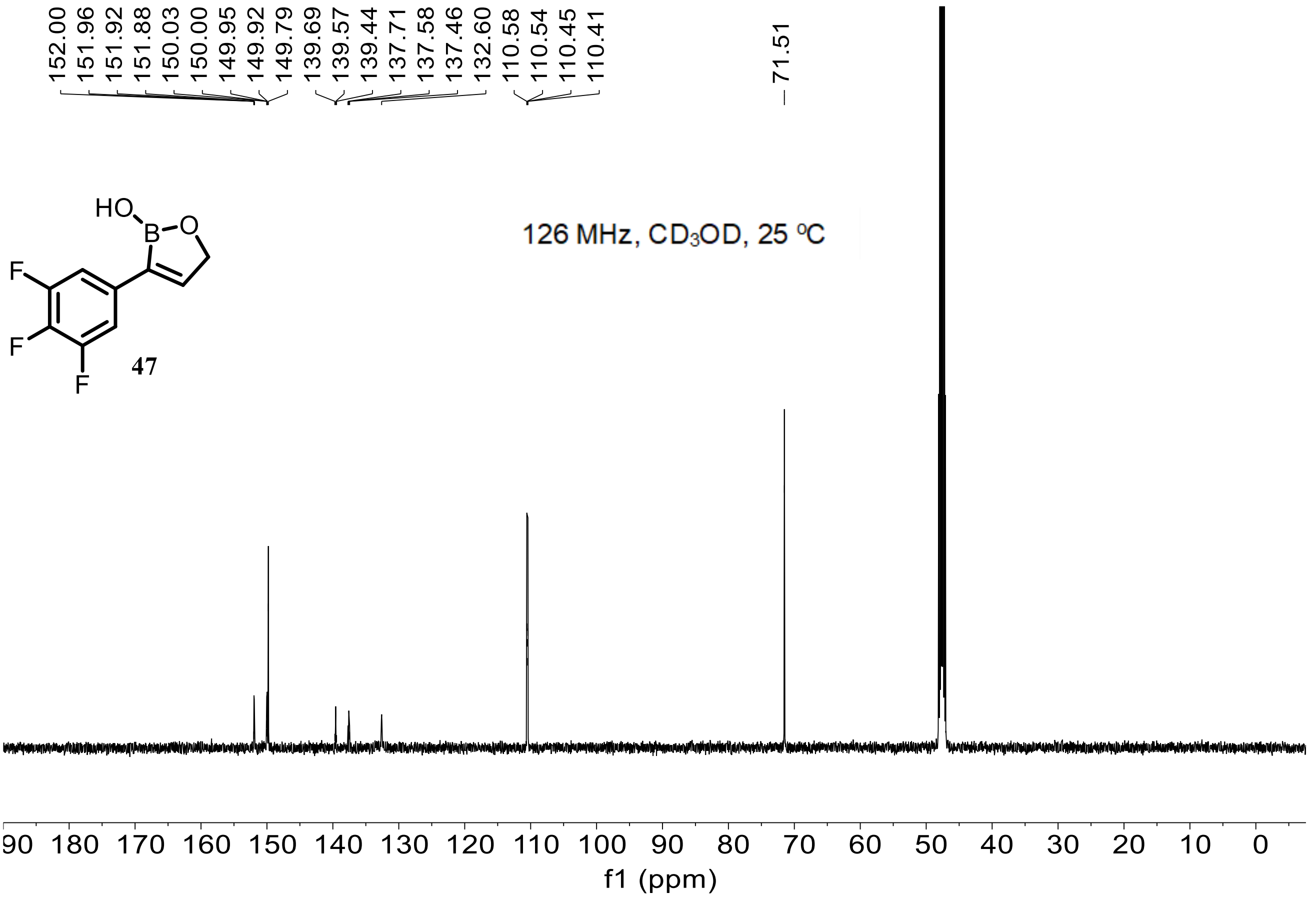
152.00
151.96
151.92
151.88
150.03
150.00
149.95
149.92
149.79
139.69
139.57
139.44
137.71
137.58
137.46
132.60
110.58
110.54
110.45
110.41
71.51
HO
B
O
F
F
F
47
126 MHz, CD_3OD, 25 °C
90 180 170 160 150 140 130 120 110 100 90 80 70 60 50 40 30 20 10 0
f1 (ppm)

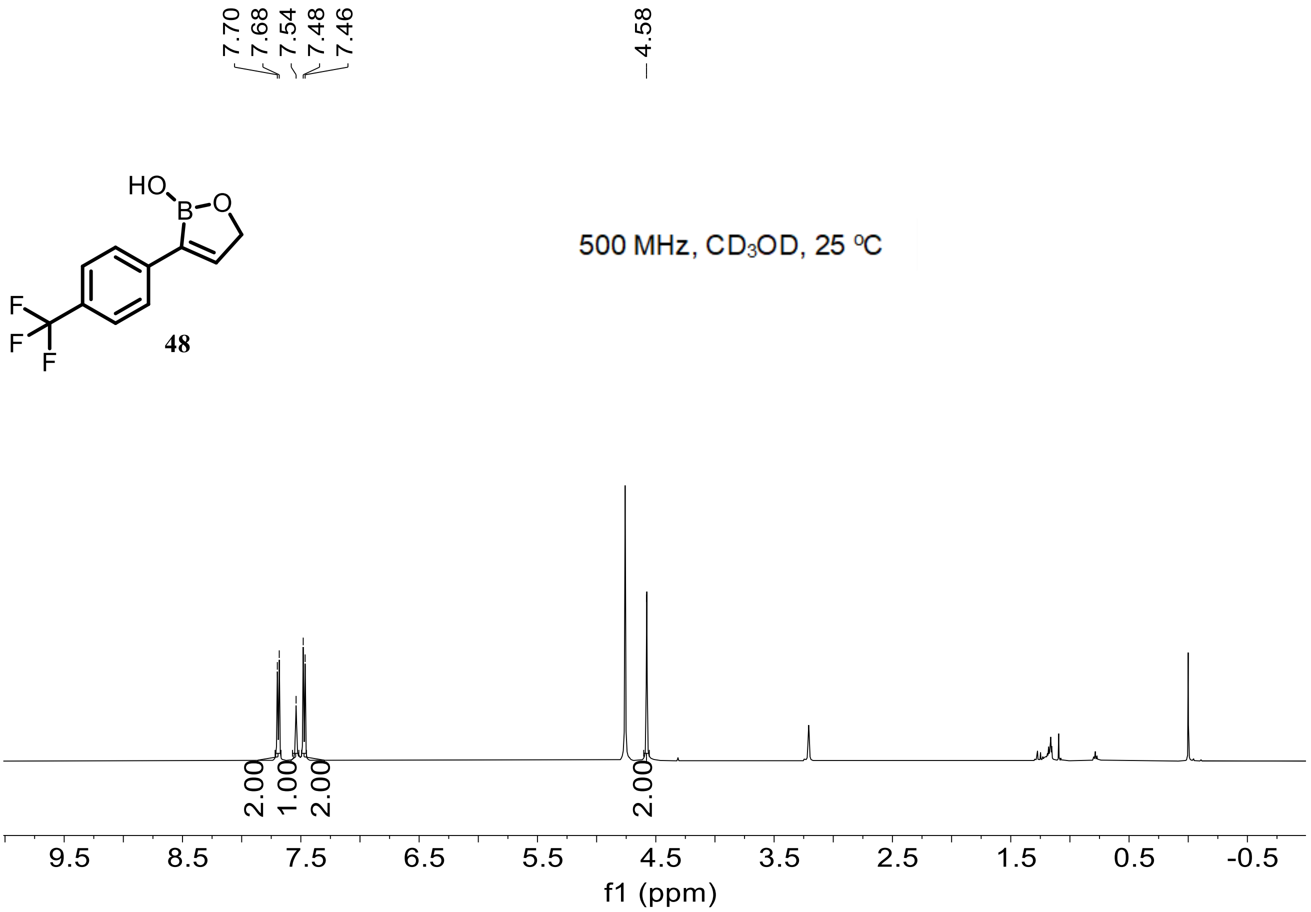
7.70
7.68
7.54
7.48
7.46
4.58
48
500 MHz, CD3OD, 25 °C
2.00
1.00
2.00
2.00
9.5 8.5 7.5 6.5 5.5 4.5 3.5 2.5 1.5 0.5 -0.5
f1 (ppm)

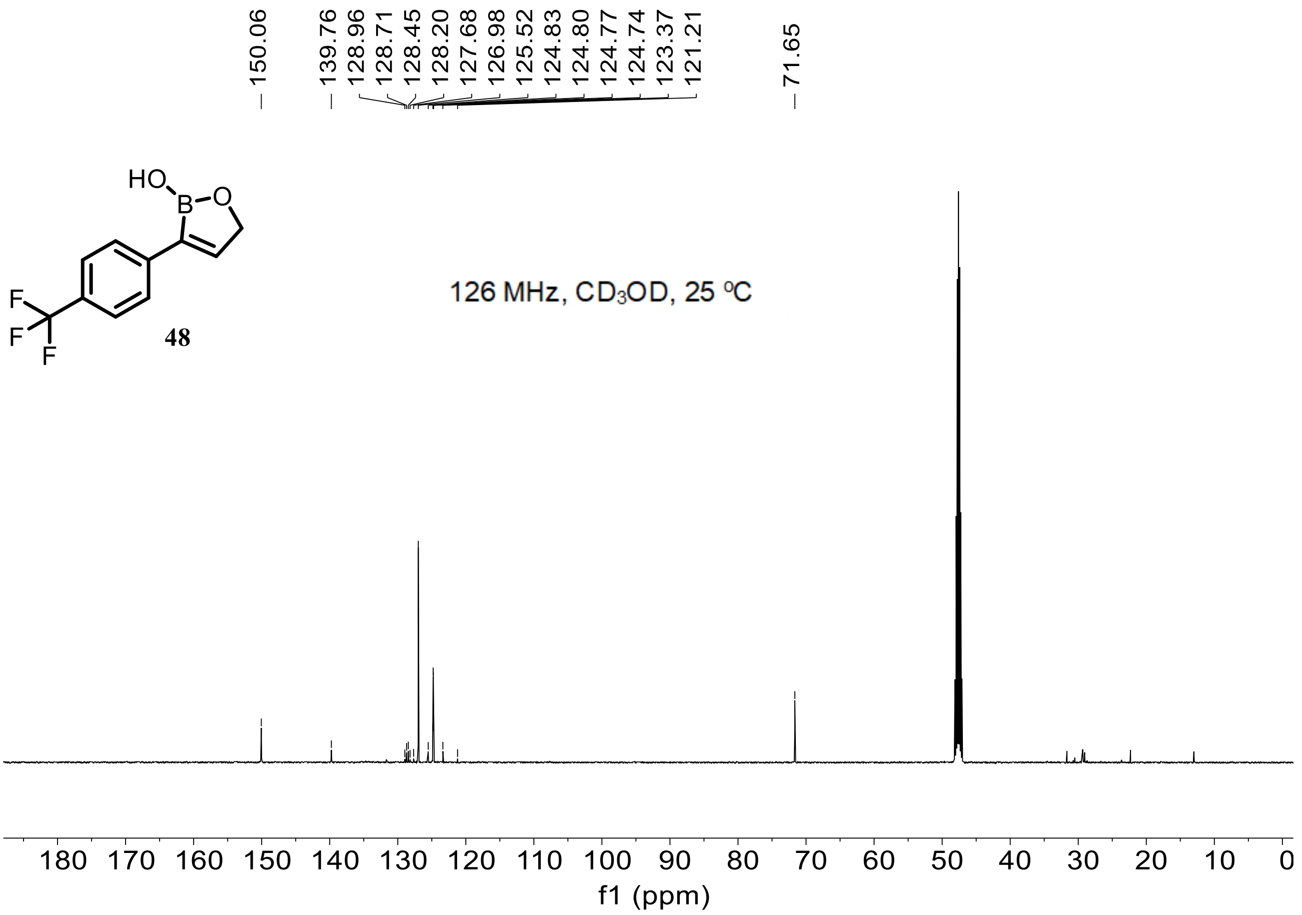
150.06
139.76
128.96
128.71
128.45
128.20
127.68
126.98
125.52
124.83
124.80
124.77
124.74
123.37
121.21
71.65
48
126 MHz, CD3OD, 25 °C
180 170 160 150 140 130 120 110 100 90 80 70 60 50 40 30 20 10 0
f1 (ppm)

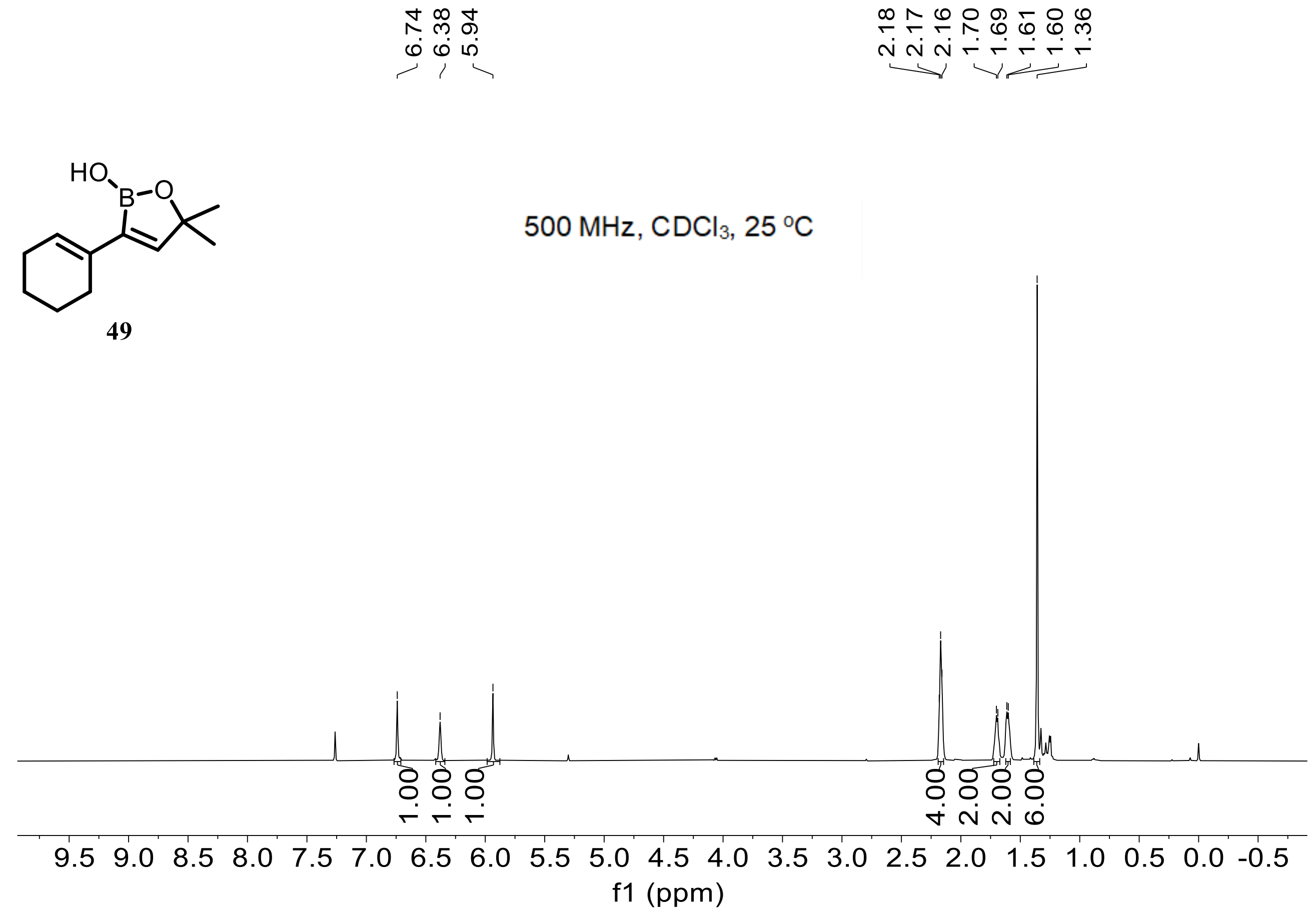

6.74
6.38
5.94
2.18
2.17
2.16
1.70
1.69
1.61
1.60
1.36
HO
B
O
49
500 MHz, CDCl3, 25 °C
1.00
1.00
1.00
4.00
2.00
2.00
6.00
9.5 9.0 8.5 8.0 7.5 7.0 6.5 6.0 5.5 5.0 4.5 4.0 3.5 3.0 2.5 2.0 1.5 1.0 0.5 0.0 -0.5
f1 (ppm)

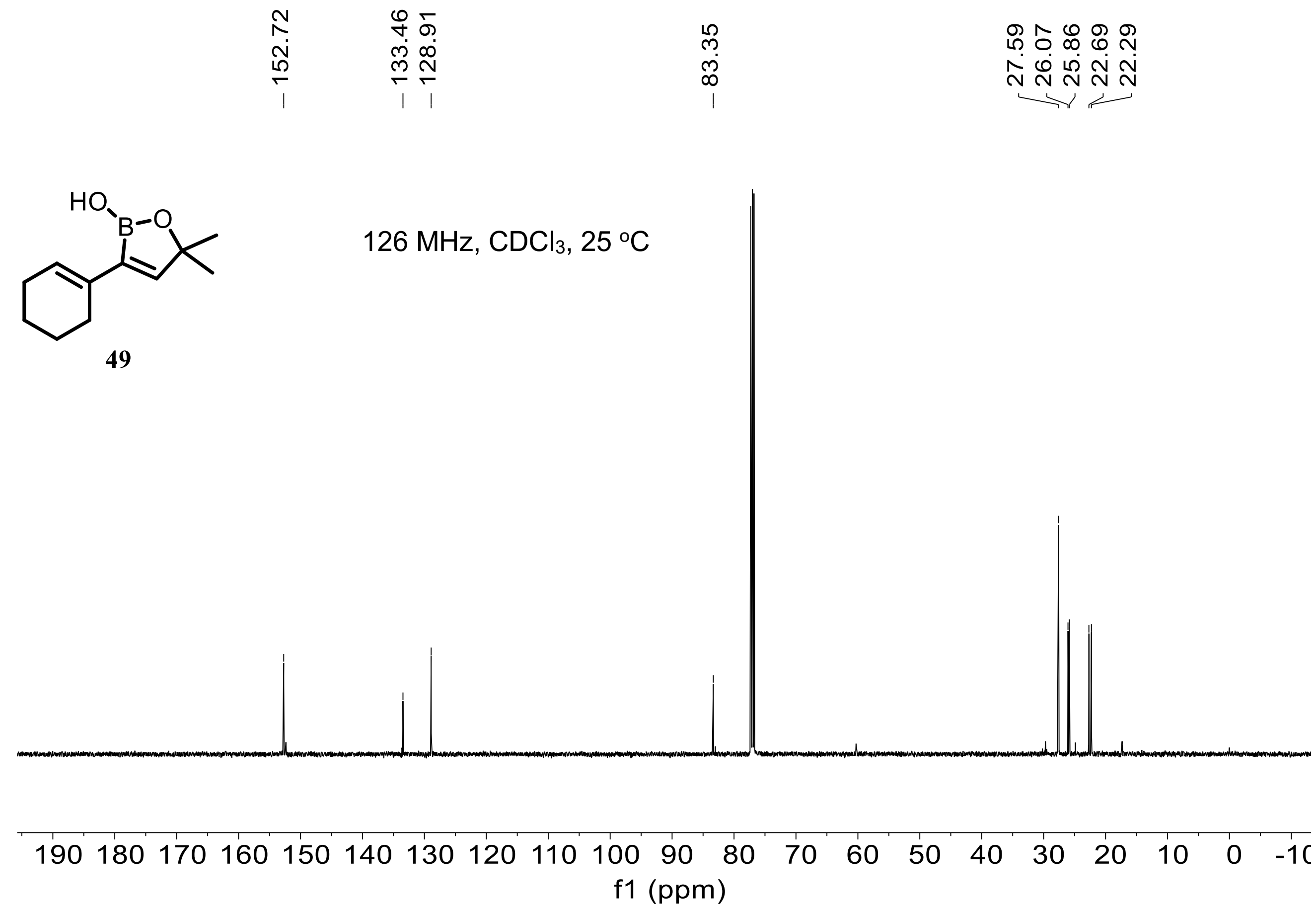

152.72
133.46
128.91
83.35
27.59
26.07
25.86
22.69
22.29
HO
B
O
49
126 MHz, CDCl3, 25 °C
190 180 170 160 150 140 130 120 110 100 90 80 70 60 50 40 30 20 10 0 -10
f1 (ppm)

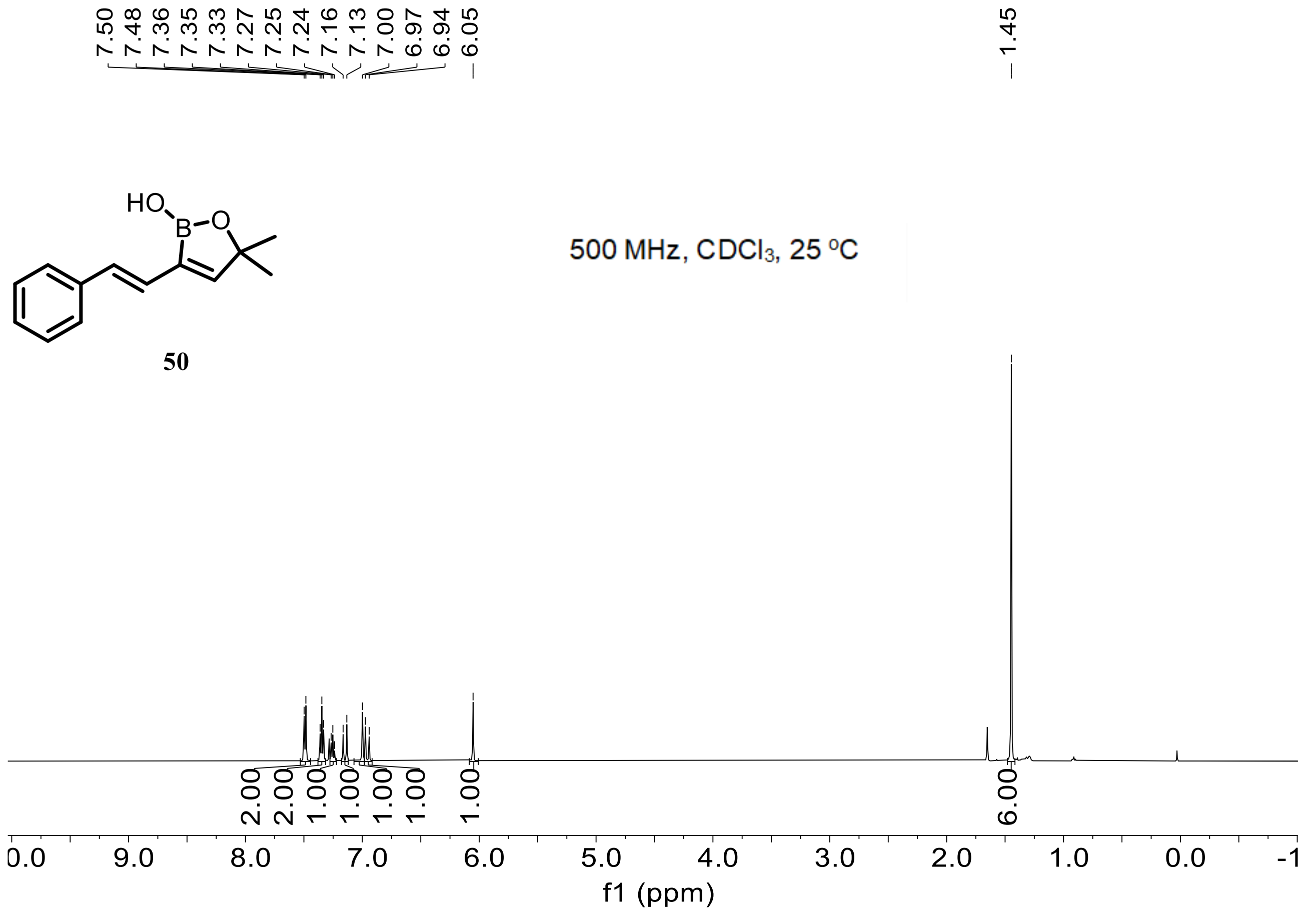
7.50 7.48 7.36 7.35 7.33 7.27 7.25 7.24 7.16 7.13 7.00 6.97 6.94 6.05
1.45
HO B O
50
500 MHz, CDCl3, 25 oC
2.00 2.00 1.00 1.00 1.00 1.00 1.00
6.00
0.0 9.0 8.0 7.0 6.0 5.0 4.0 3.0 2.0 1.0 0.0 -1
f1 (ppm)

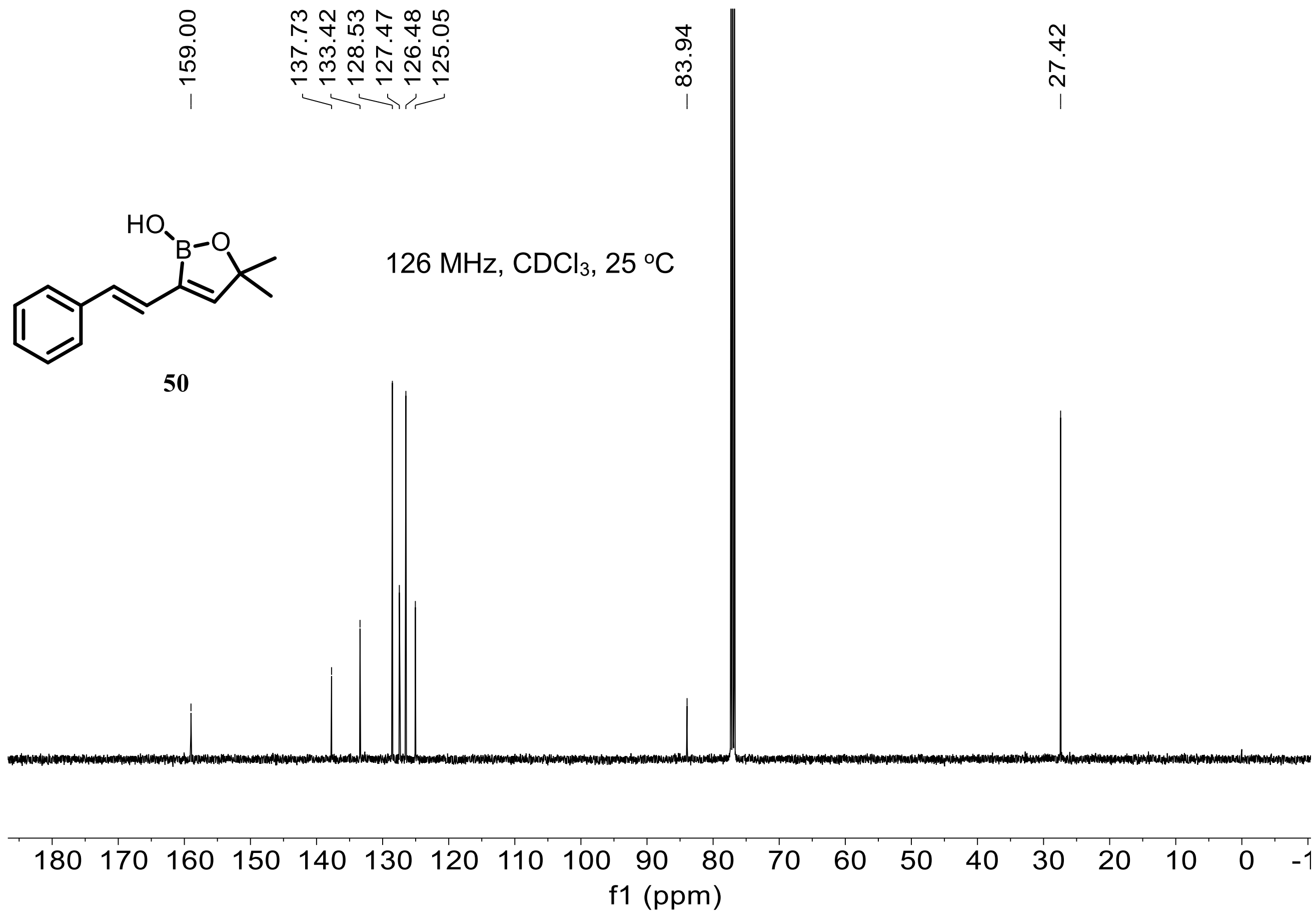
159.00
137.73 133.42 128.53 127.47 126.48 125.05
83.94
27.42
HO B O
50
126 MHz, CDCl3, 25 oC
180 170 160 150 140 130 120 110 100 90 80 70 60 50 40 30 20 10 0 -1
f1 (ppm)

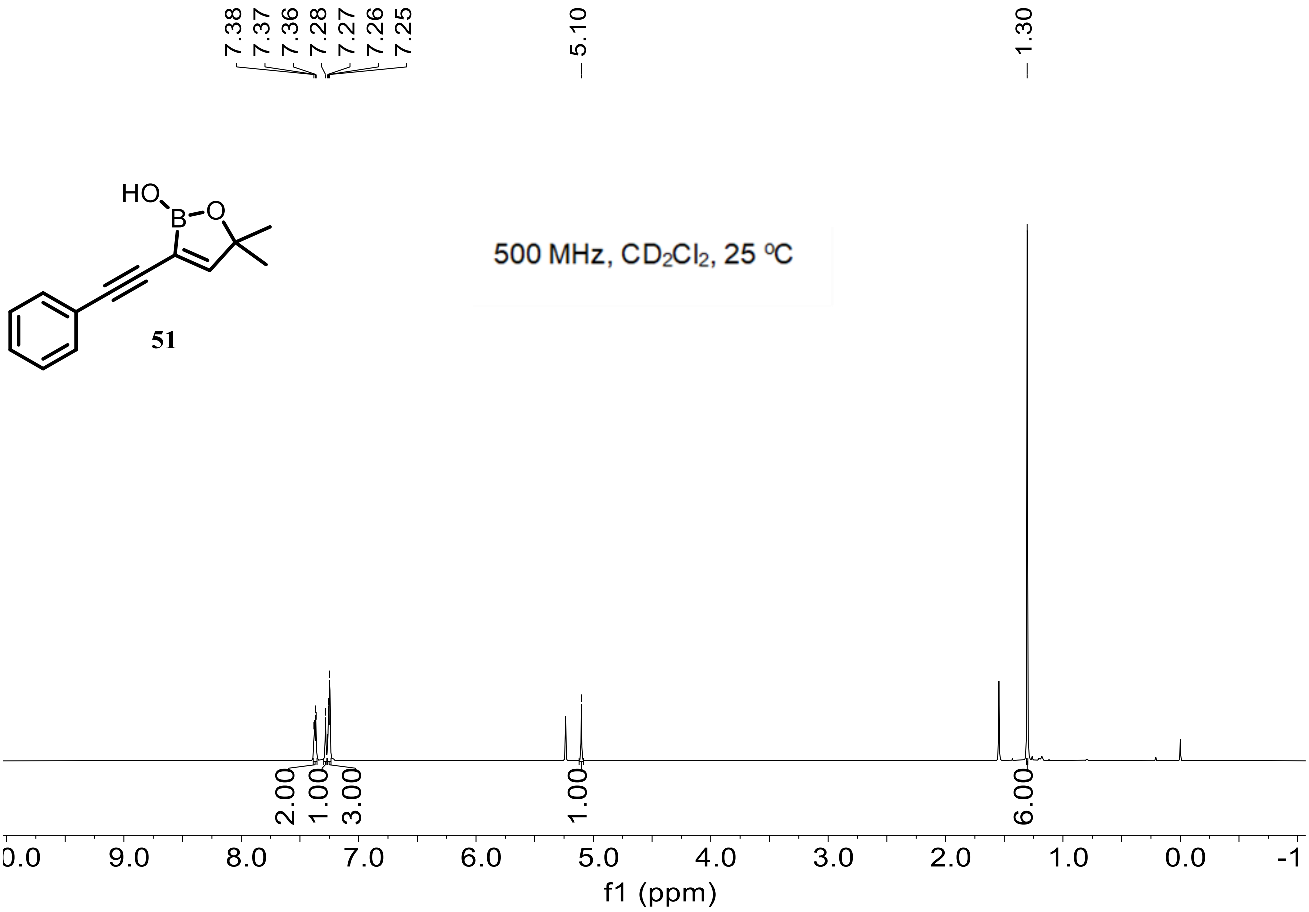
7.38 7.37 7.36 7.28 7.27 7.26 7.25
5.10
1.30
HO
B
O
51
500 MHz, CD2Cl2, 25 °C
2.00 1.00 3.00
1.00
6.00
0.0 9.0 8.0 7.0 6.0 5.0 4.0 3.0 2.0 1.0 0.0 -1
f1 (ppm)

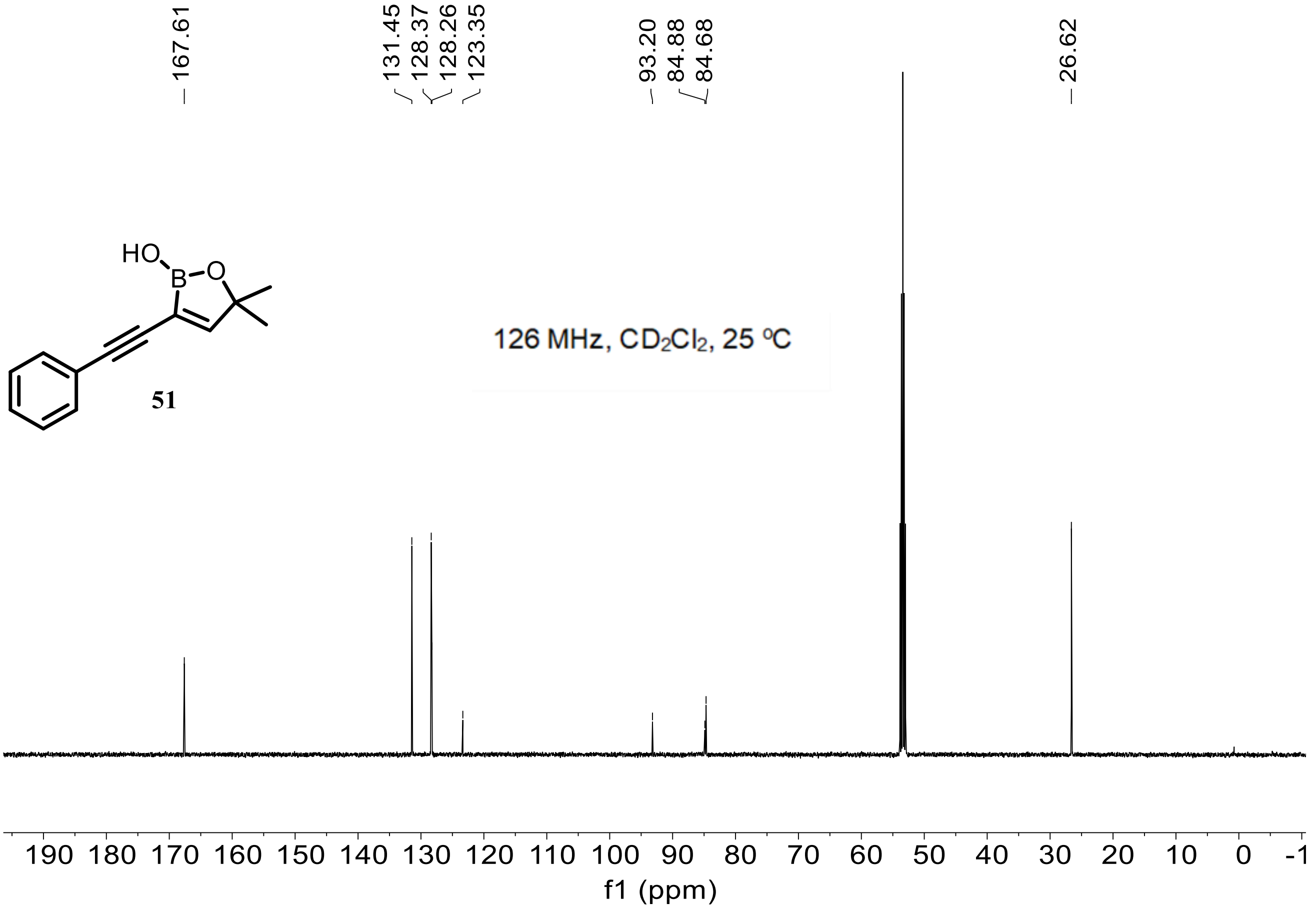
167.61
131.45 128.37 128.26 123.35
93.20 84.88 84.68
26.62
HO
B
O
51
126 MHz, CD2Cl2, 25 °C
190 180 170 160 150 140 130 120 110 100 90 80 70 60 50 40 30 20 10 0 -1
f1 (ppm)

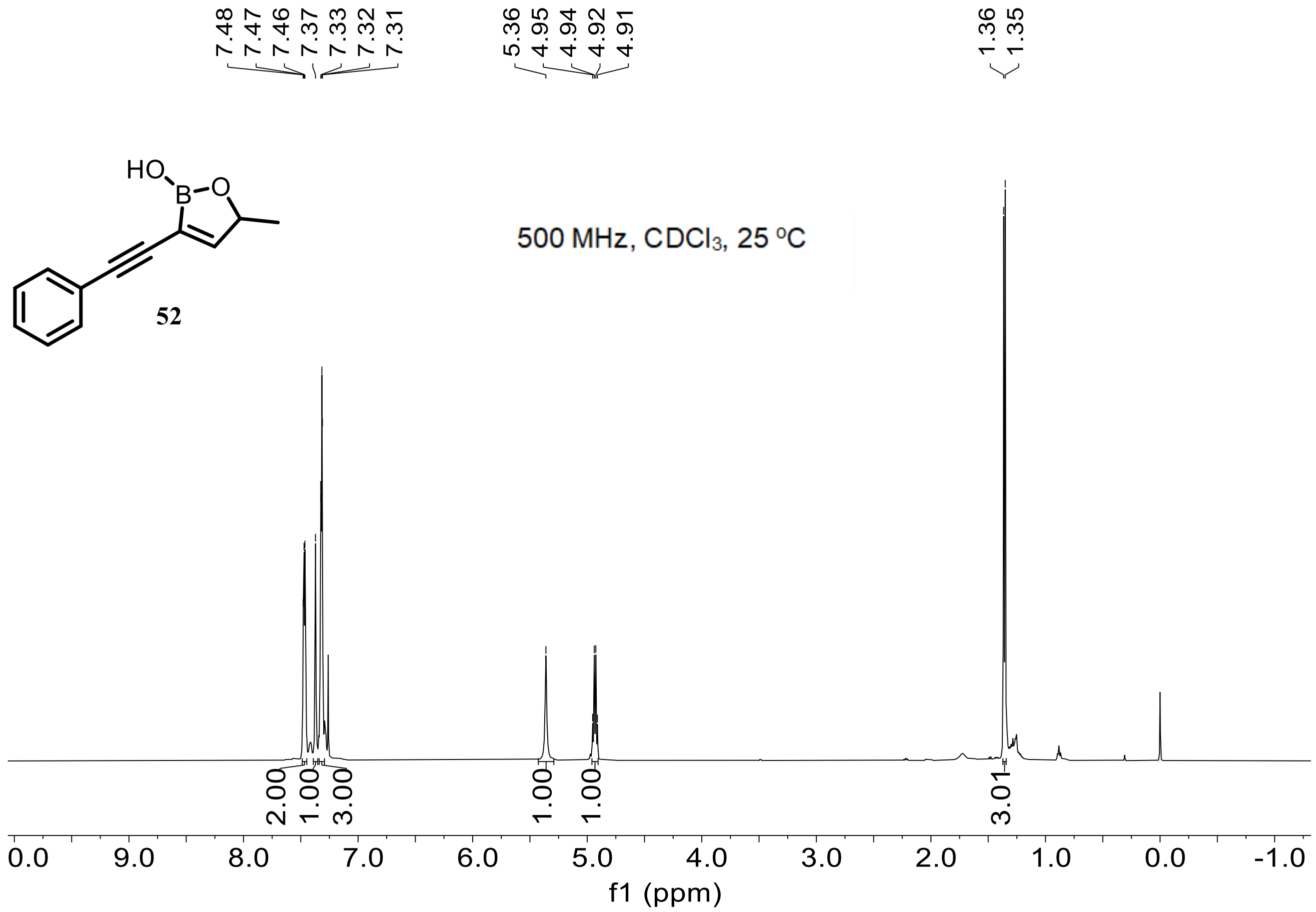
7.48
7.47
7.46
7.37
7.33
7.32
7.31
5.36
4.95
4.94
4.92
4.91
1.36
1.35
HO
B
O
52
500 MHz, $CDCl_3$, 25 °C
2.00
1.00
3.00
1.00
1.00
3.01
0.0 9.0 8.0 7.0 6.0 5.0 4.0 3.0 2.0 1.0 0.0 -1.0
f1 (ppm)

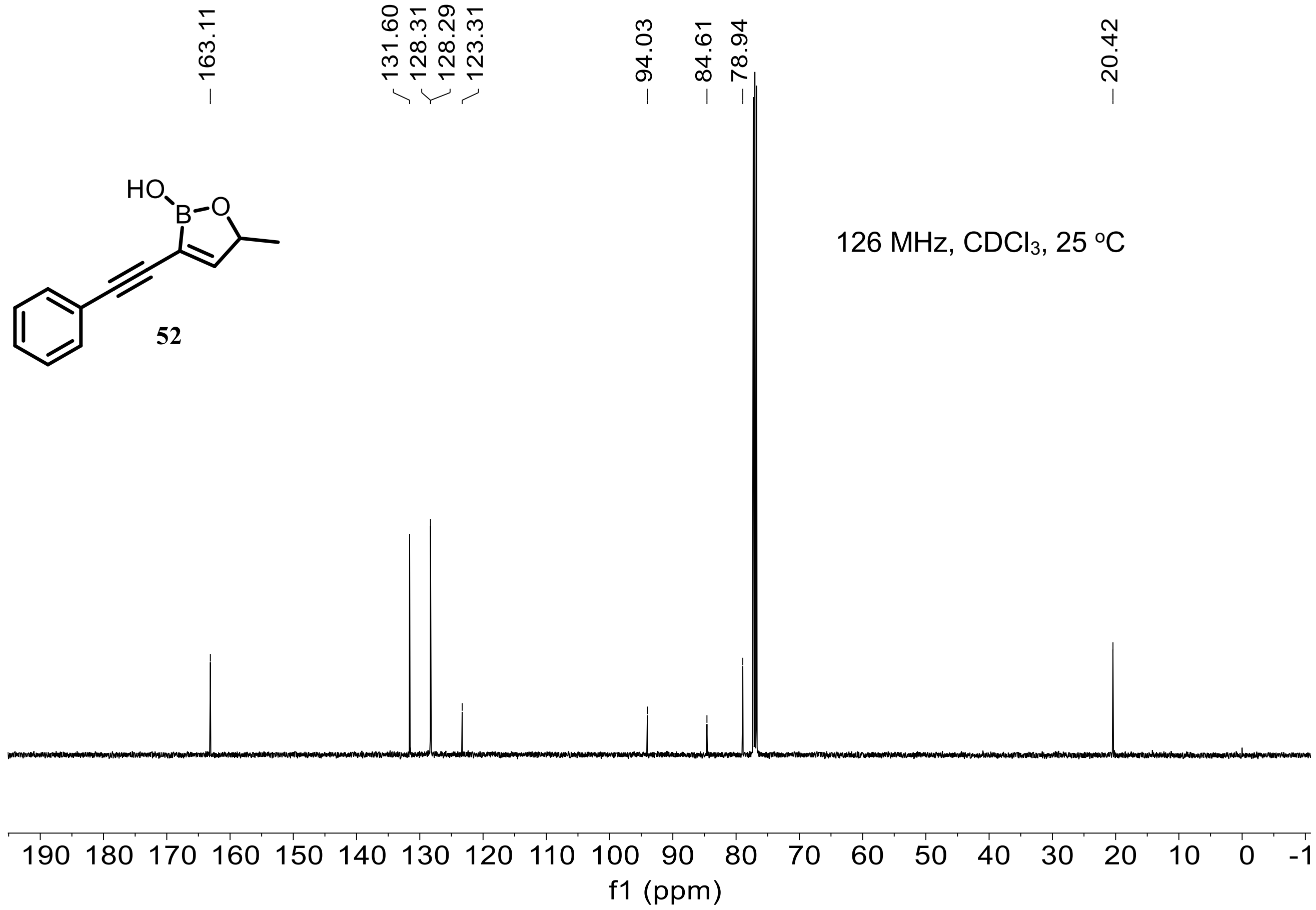
163.11
131.60
128.31
128.29
123.31
94.03
84.61
78.94
20.42
HO
B
O
52
126 MHz, $CDCl_3$, 25 °C
190 180 170 160 150 140 130 120 110 100 90 80 70 60 50 40 30 20 10 0 -1
f1 (ppm)

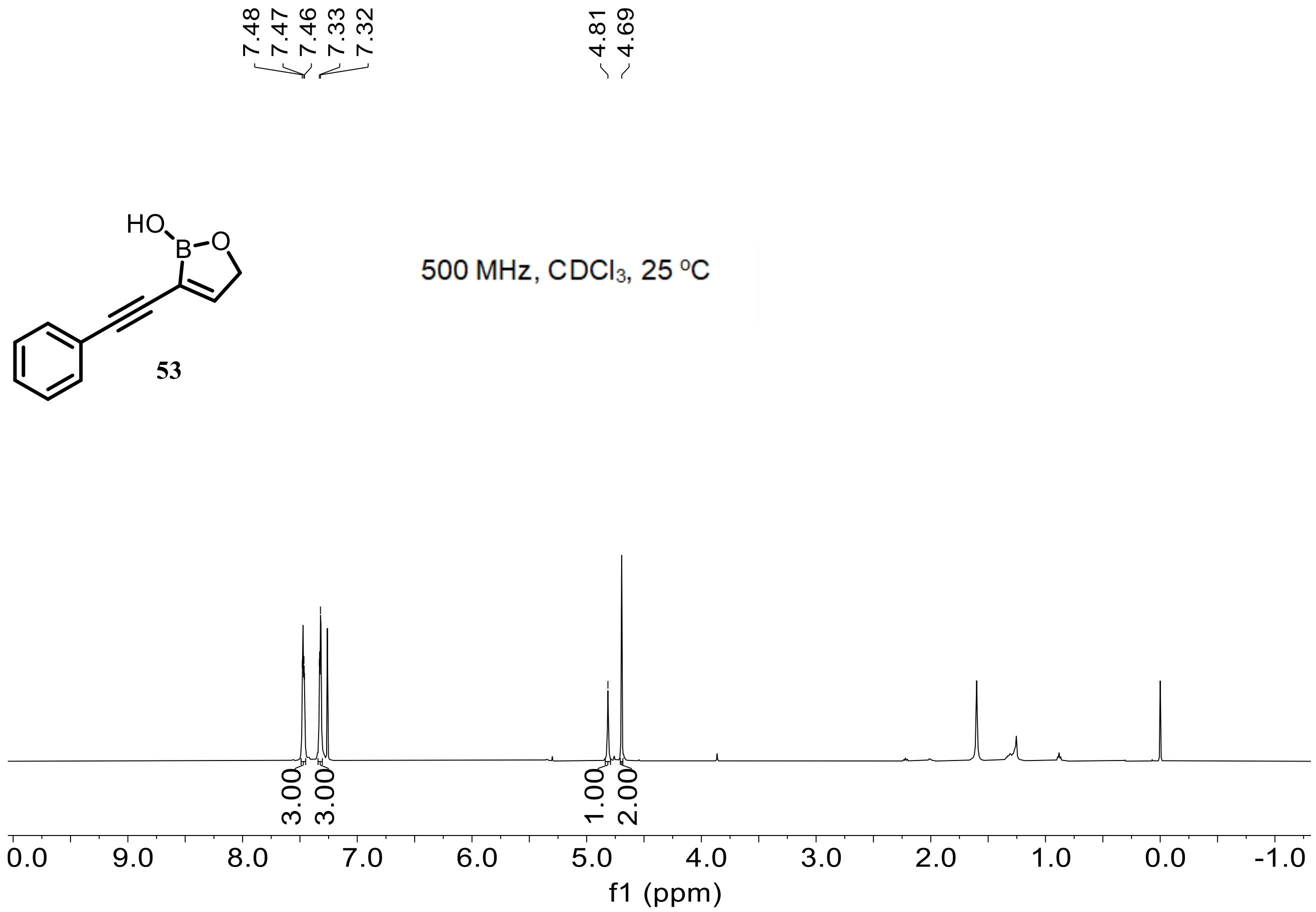
7.48
7.47
7.46
7.33
7.32
4.81
4.69
HO
B
O
53
500 MHz, CDCl3, 25 °C
3.00
3.00
1.00
2.00
0.0 9.0 8.0 7.0 6.0 5.0 4.0 3.0 2.0 1.0 0.0 -1.0
f1 (ppm)

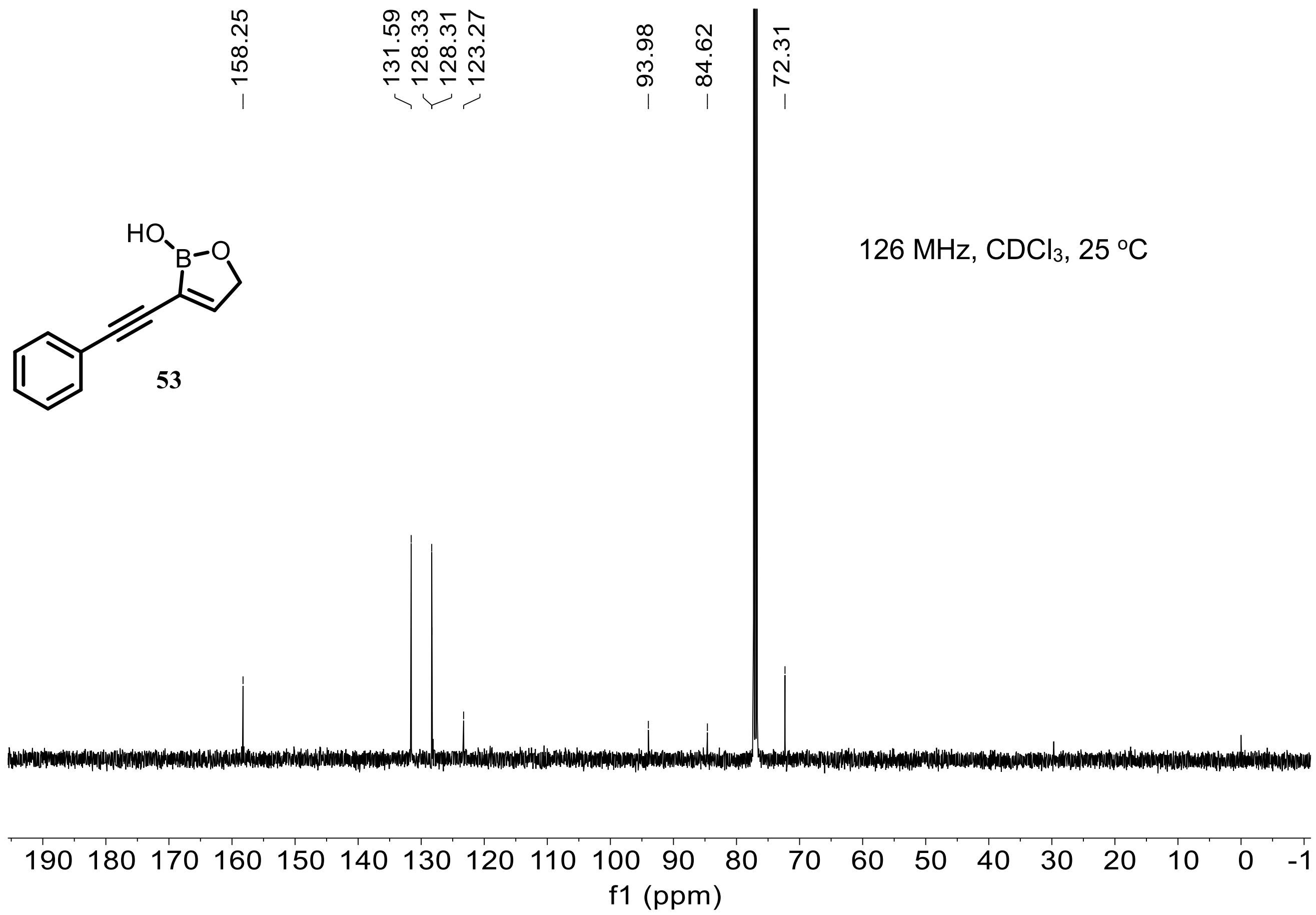
158.25
131.59
128.33
128.31
123.27
93.98
84.62
72.31
HO
B
O
53
126 MHz, CDCl3, 25 °C
190 180 170 160 150 140 130 120 110 100 90 80 70 60 50 40 30 20 10 0 -1
f1 (ppm)

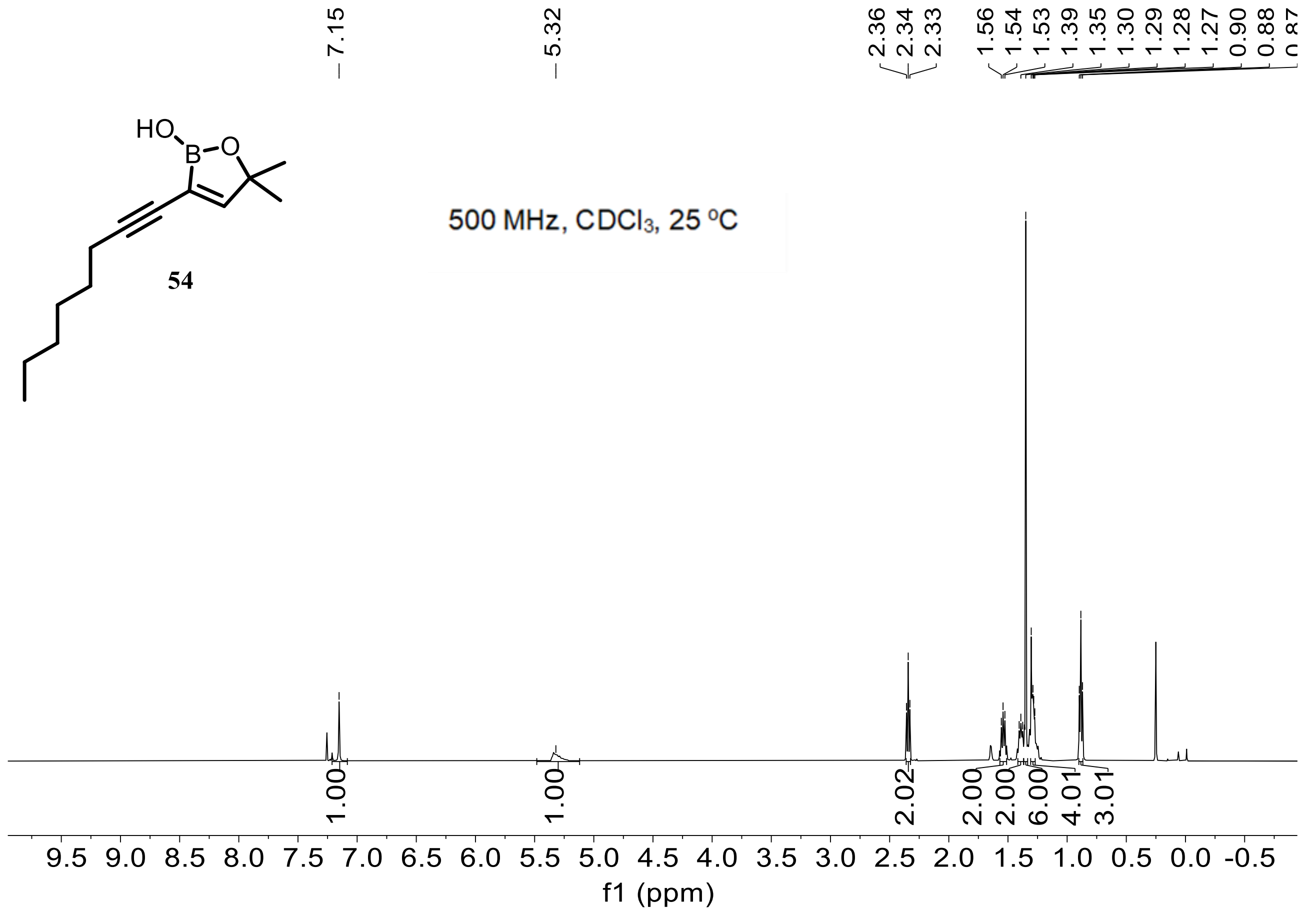
7.15
5.32
2.36
2.34
2.33
1.56
1.54
1.53
1.39
1.35
1.30
1.29
1.28
1.27
0.90
0.88
HO
B
O
54
500 MHz, CDCl3, 25 °C
1.00
1.00
2.02
2.00
2.00
6.00
4.01
3.01
9.5 9.0 8.5 8.0 7.5 7.0 6.5 6.0 5.5 5.0 4.5 4.0 3.5 3.0 2.5 2.0 1.5 1.0 0.5 0.0 -0.5
f1 (ppm)

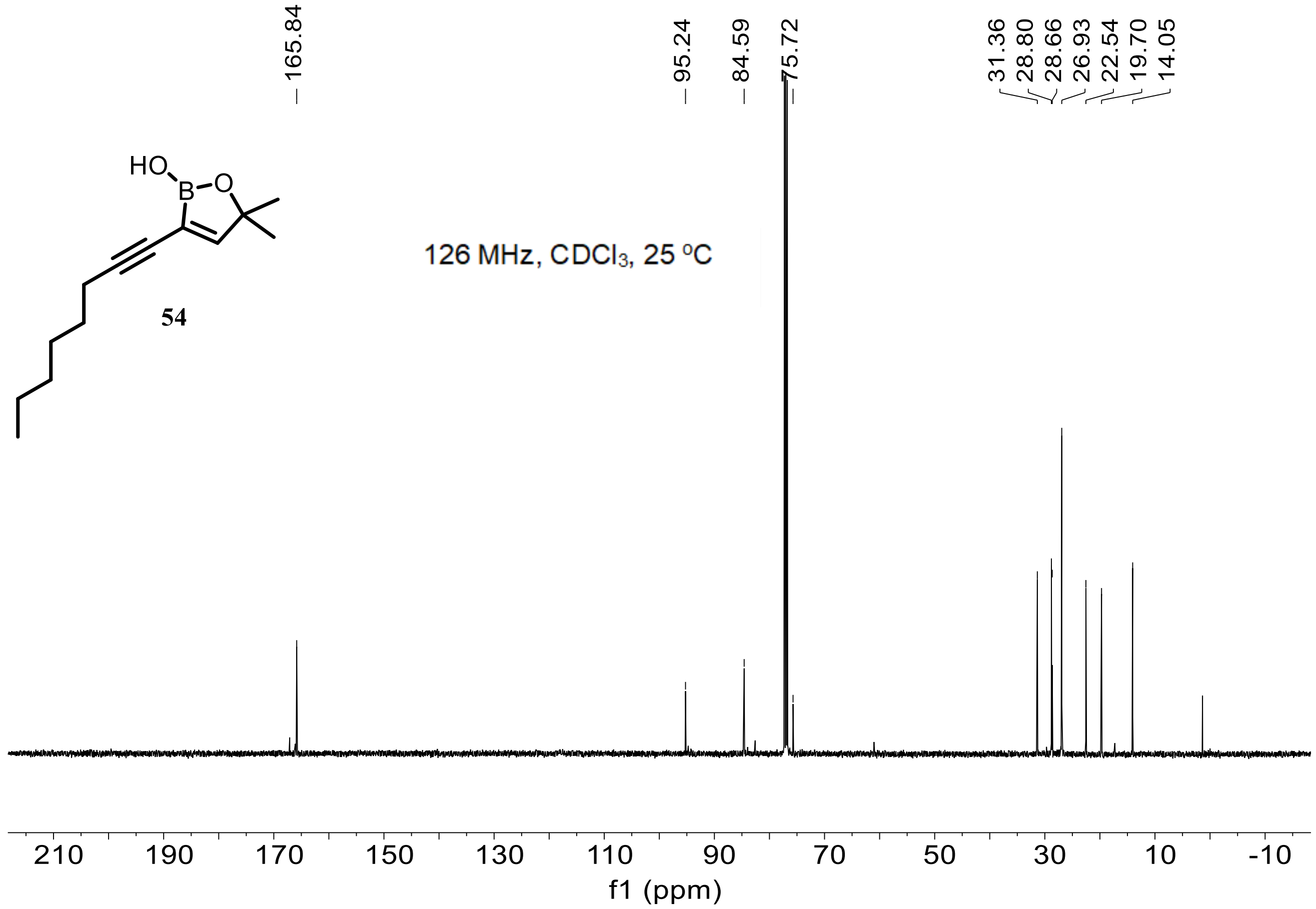
165.84
95.24
84.59
75.72
31.36
28.80
28.66
26.93
22.54
19.70
14.05
HO
B
O
54
126 MHz, CDCl3, 25 °C
210 190 170 150 130 110 90 70 50 30 10 -10
f1 (ppm)

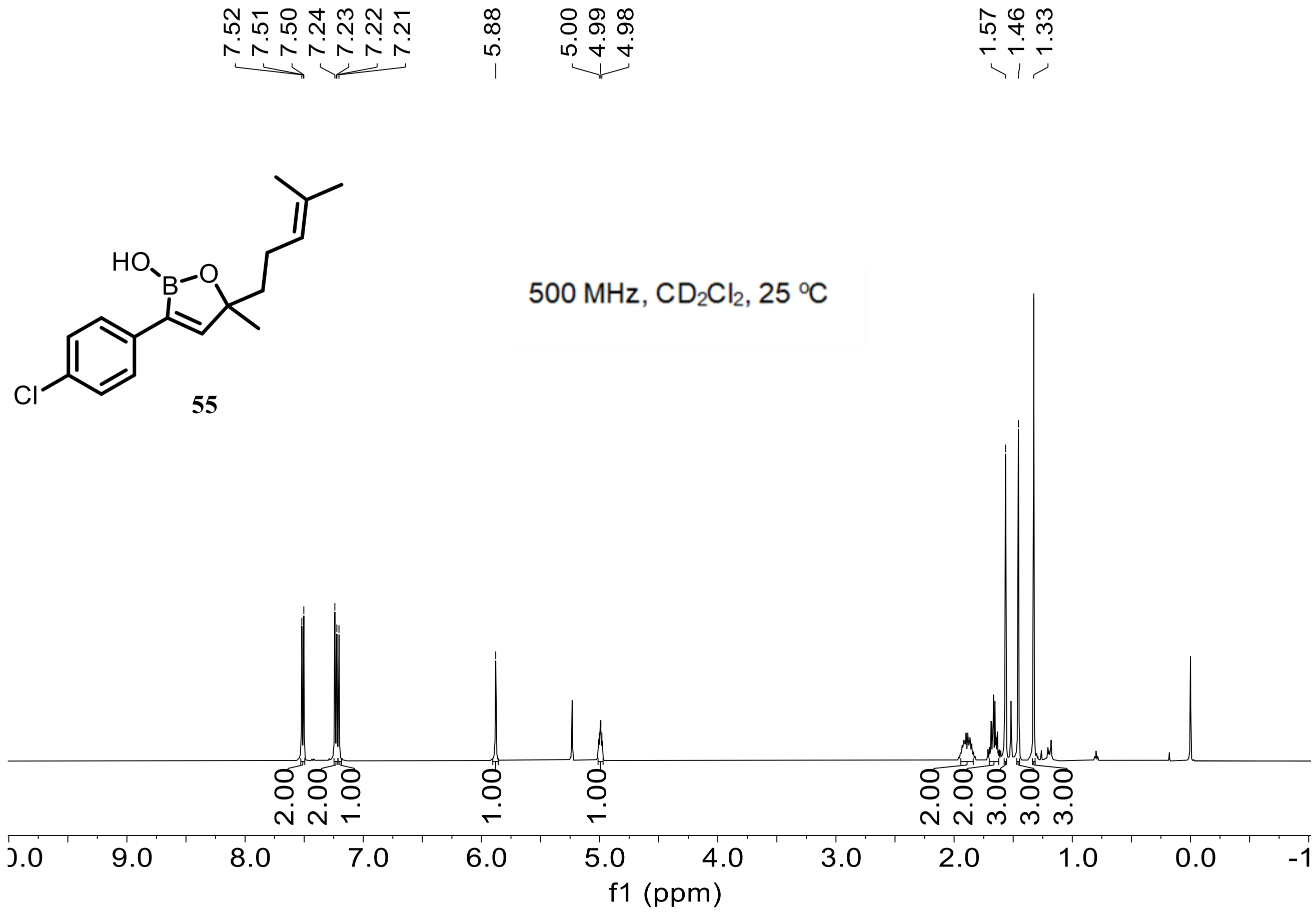
7.52
7.51
7.50
7.24
7.23
7.22
7.21
5.88
5.00
4.99
4.98
1.57
1.46
1.33
HO
B
O
Cl
55
500 MHz, CD2Cl2, 25 °C
2.00
2.00
1.00
1.00
1.00
2.00
2.00
3.00
3.00
3.00
9.0
8.0
7.0
6.0
5.0
4.0
3.0
2.0
1.0
0.0
f1 (ppm)

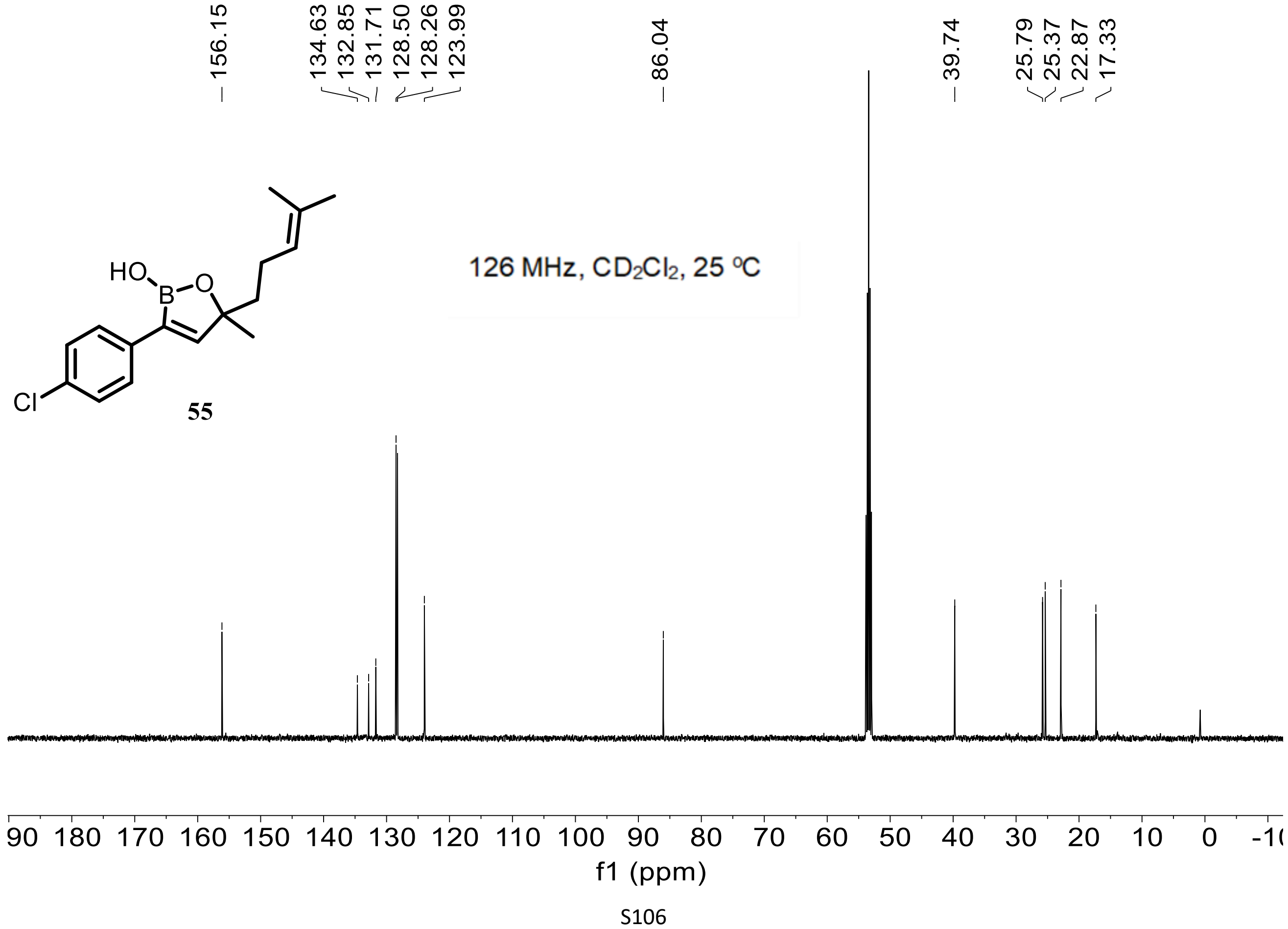
156.15
134.63
132.85
131.71
128.50
128.26
123.99
86.04
39.74
25.79
25.37
22.87
17.33
HO
B
O
Cl
55
126 MHz, CD2Cl2, 25 °C
180
170
160
150
140
130
120
110
100
90
80
70
60
50
40
30
20
10
0
f1 (ppm)

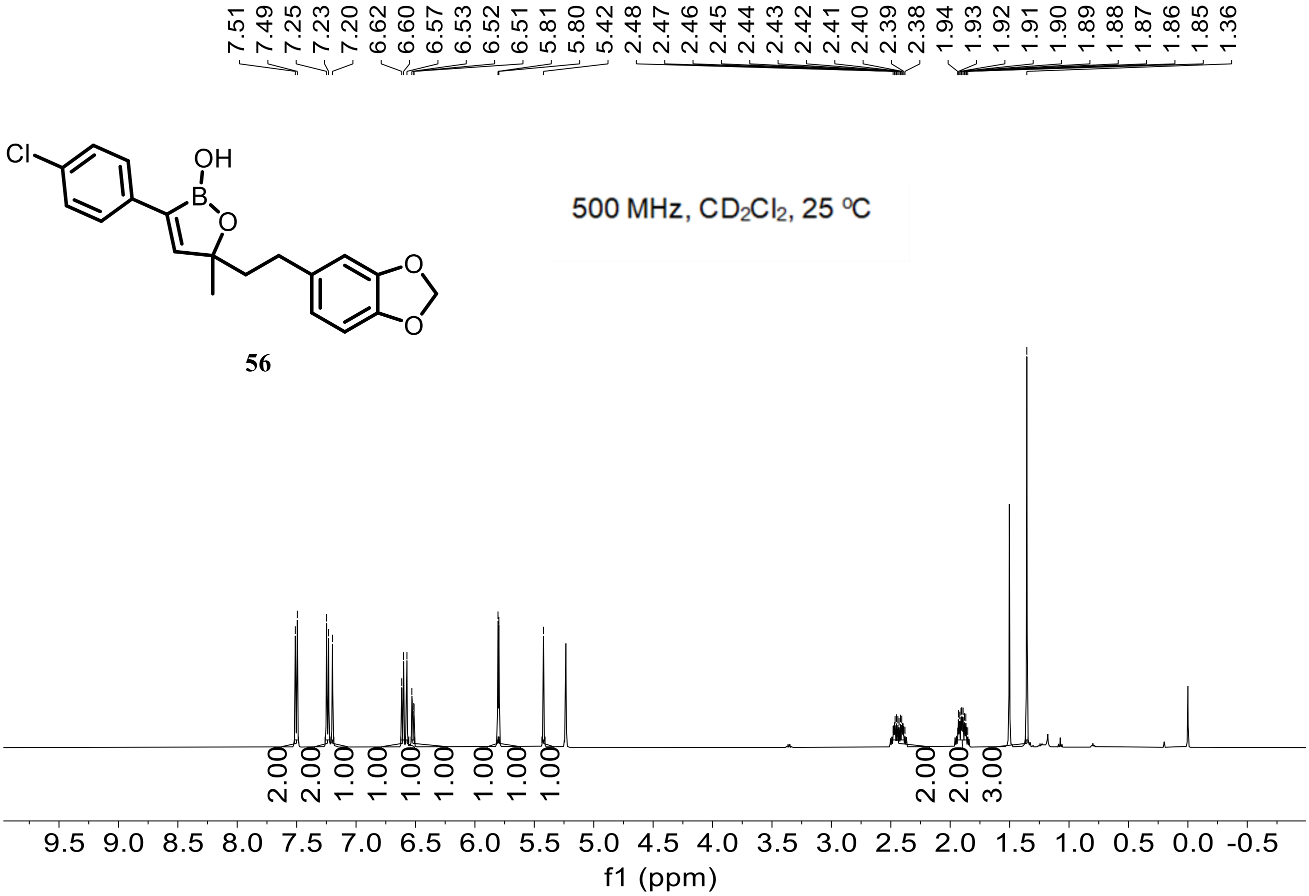
7.51 7.49 7.25 7.23 7.20 6.62 6.60 6.57 6.53 6.52 6.51 5.81 5.80 5.42 2.48 2.47 2.46 2.45 2.44 2.43 2.42 2.41 2.40 2.39 2.38 1.94 1.93 1.92 1.91 1.90 1.89 1.88 1.87 1.86 1.85 1.36
Cl
OH
B
O
O
O
56
500 MHz, CD_2Cl_2, 25 °C
2.00 2.00 1.00 1.00 1.00 1.00 1.00 1.00 1.00 2.00 2.00 3.00
9.5 9.0 8.5 8.0 7.5 7.0 6.5 6.0 5.5 5.0 4.5 4.0 3.5 3.0 2.5 2.0 1.5 1.0 0.5 0.0 -0.5
f1 (ppm)

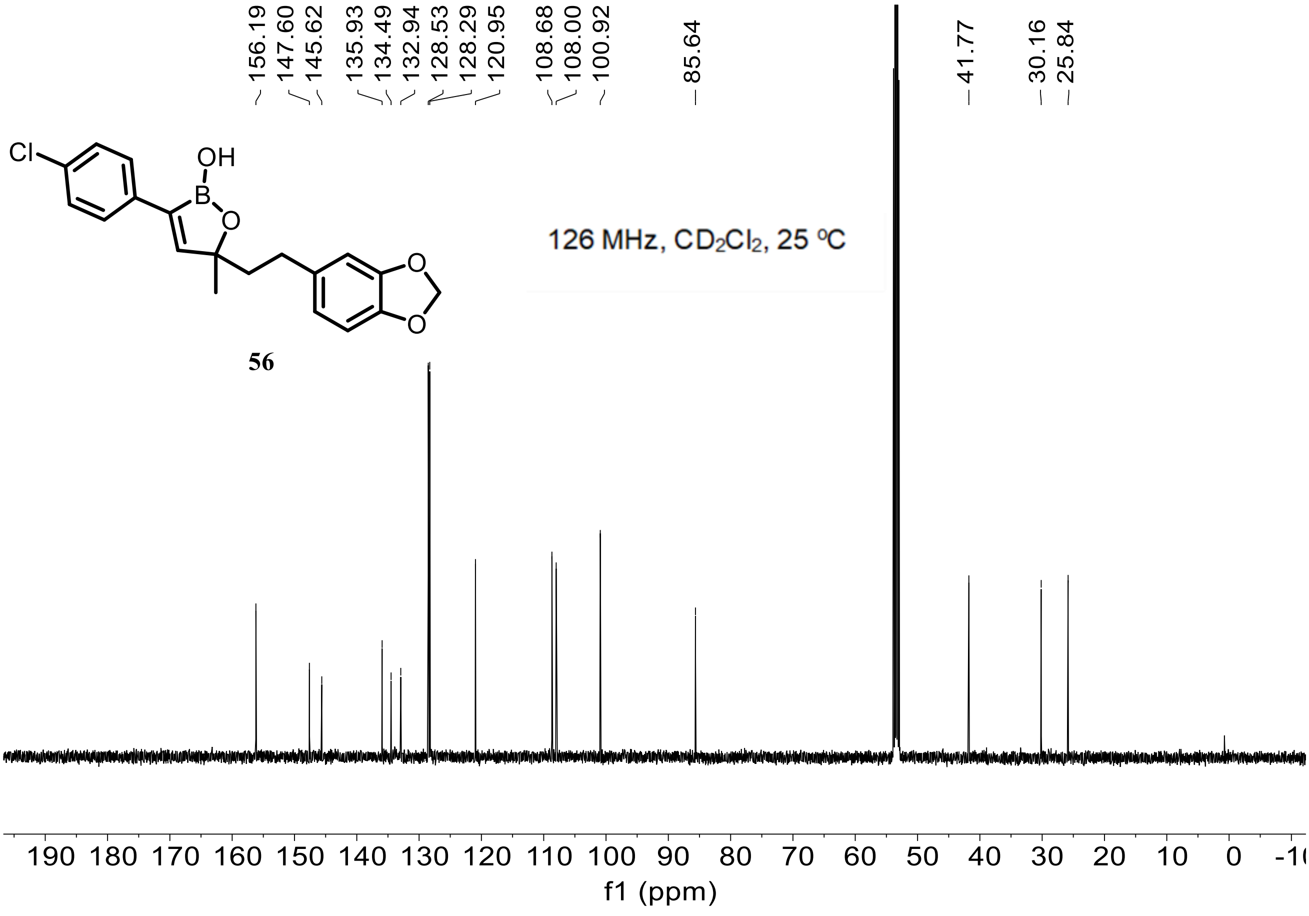
156.19 147.60 145.62 135.93 134.49 132.94 128.53 128.29 120.95 108.68 108.00 100.92 85.64 41.77 30.16 25.84
Cl
OH
B
O
O
O
56
126 MHz, CD_2Cl_2, 25 °C
190 180 170 160 150 140 130 120 110 100 90 80 70 60 50 40 30 20 10 0 -1
f1 (ppm)

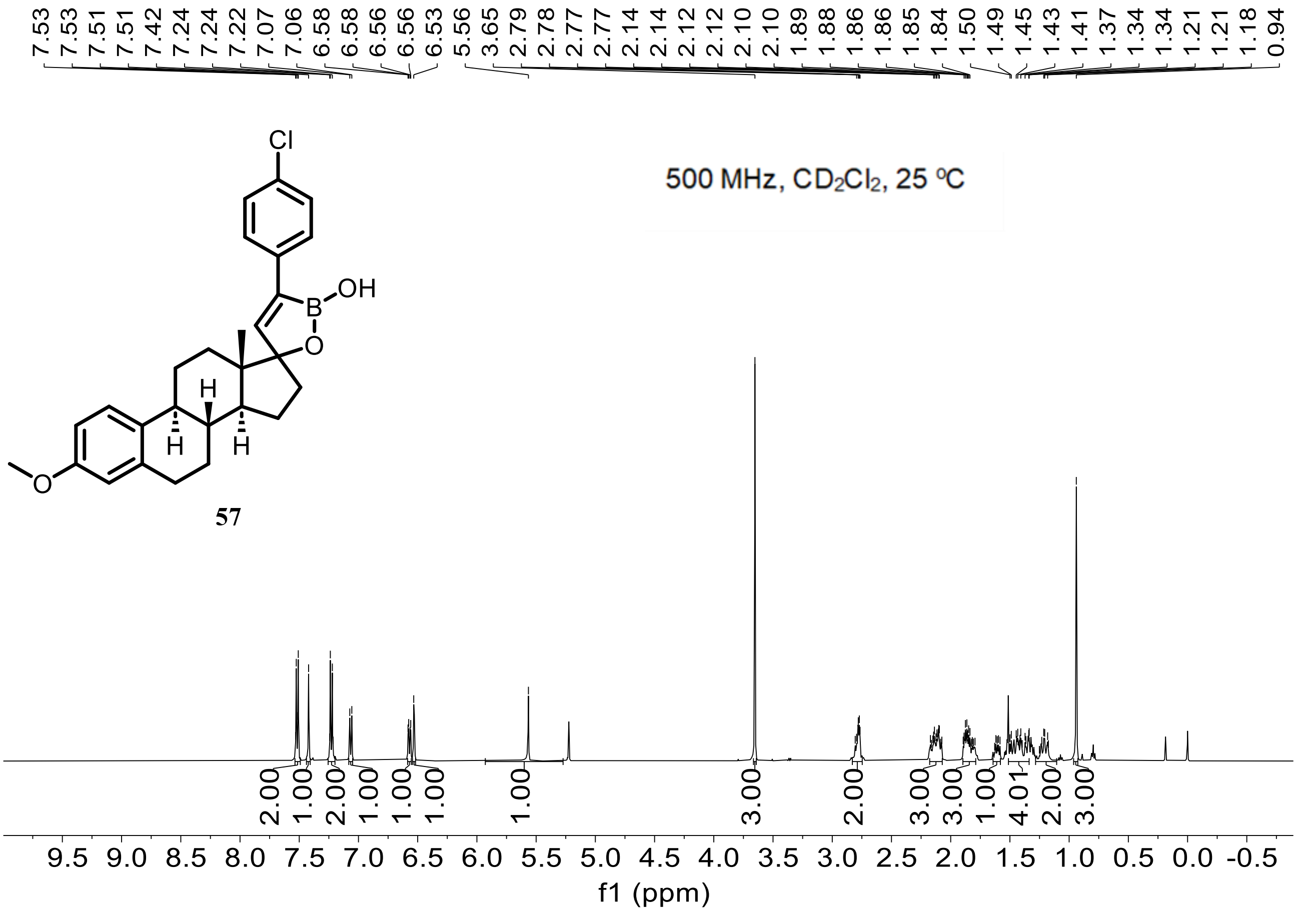
7.53 7.53 7.51 7.51 7.42 7.24 7.24 7.22 7.07 7.06 6.58 6.58 6.56 6.56 6.53 5.56 3.65 2.79 2.78 2.77 2.77 2.14 2.14 2.12 2.12 2.10 2.10 1.89 1.88 1.86 1.86 1.85 1.84 1.50 1.49 1.45 1.43 1.41 1.37 1.34 1.34 1.21 1.21 1.18 0.94
500 MHz, CD_2Cl_2, 25 °C
Cl
OH
B
O
H
H
H
O
57
2.00 1.00 2.00 1.00 1.00 1.00 1.00 3.00 2.00 3.00 3.00 1.00 4.01 2.00 3.00
9.5 9.0 8.5 8.0 7.5 7.0 6.5 6.0 5.5 5.0 4.5 4.0 3.5 3.0 2.5 2.0 1.5 1.0 0.5 0.0 -0.5
f1 (ppm)

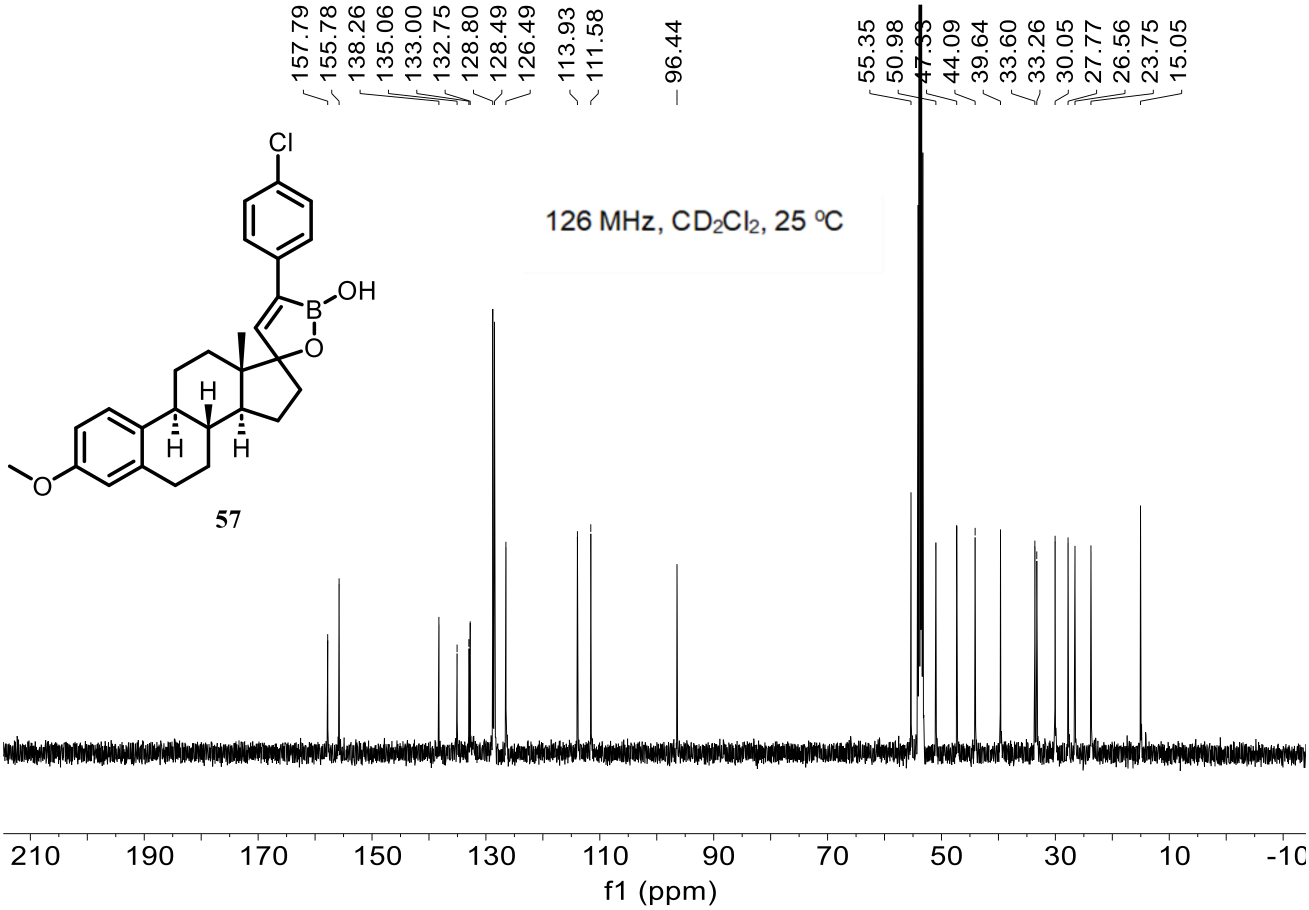
157.79 155.78 138.26 135.06 133.00 132.75 128.80 128.49 126.49 113.93 111.58 96.44
55.35 50.98 47.33 44.09 39.64 33.60 33.26 30.05 27.77 26.56 23.75 15.05
126 MHz, CD_2Cl_2, 25 °C
Cl
OH
B
O
H
H
H
O
57
210 190 170 150 130 110 90 70 50 30 10 -10
f1 (ppm)

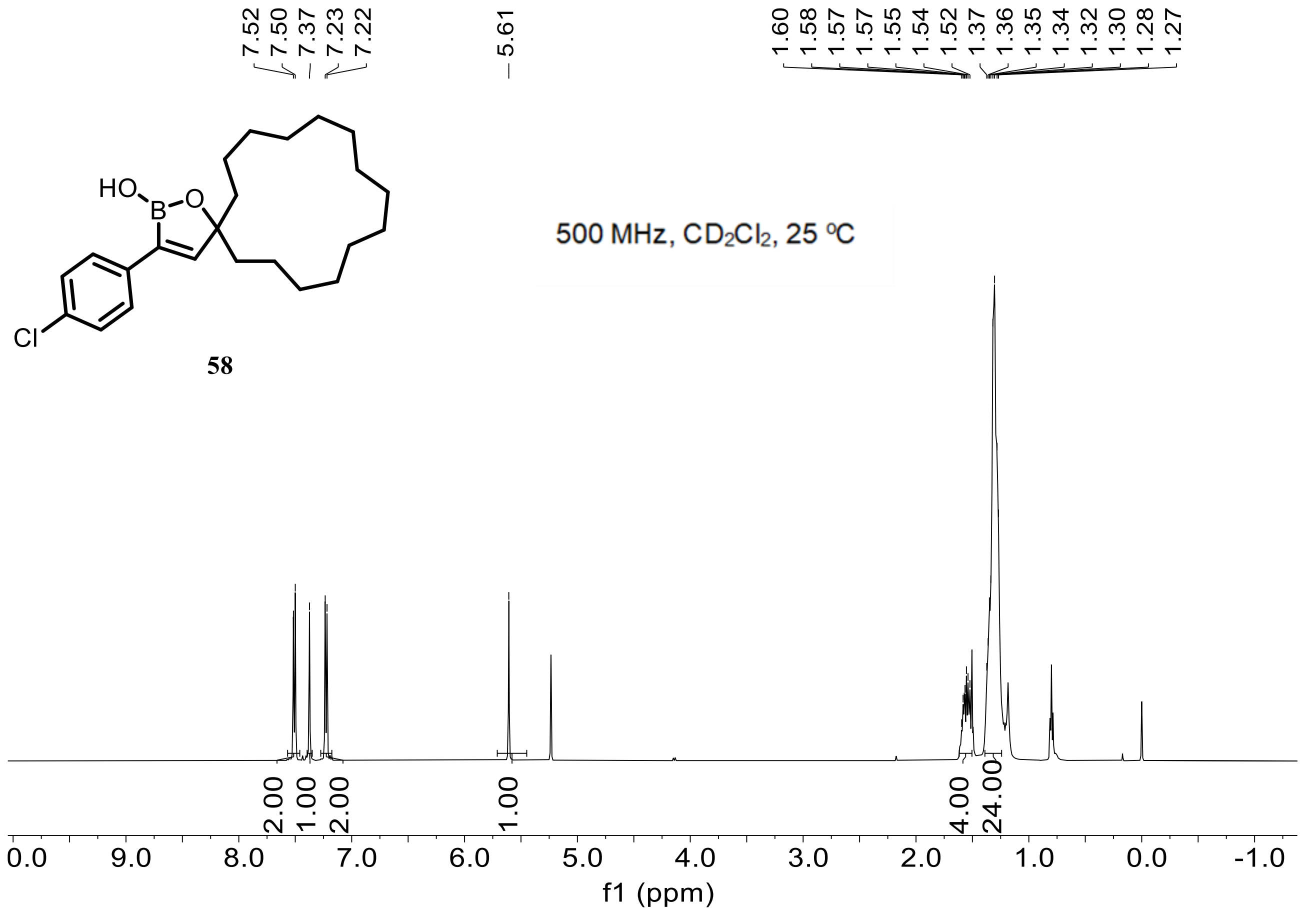
7.52
7.50
7.37
7.23
7.22
5.61
1.60
1.58
1.57
1.57
1.55
1.54
1.52
1.37
1.36
1.35
1.34
1.32
1.30
1.28
1.27
HO
B
O
Cl
58
500 MHz, CD2Cl2, 25 ºC
2.00
1.00
2.00
1.00
4.00
24.00
0.0 9.0 8.0 7.0 6.0 5.0 4.0 3.0 2.0 1.0 0.0 -1.0
f1 (ppm)

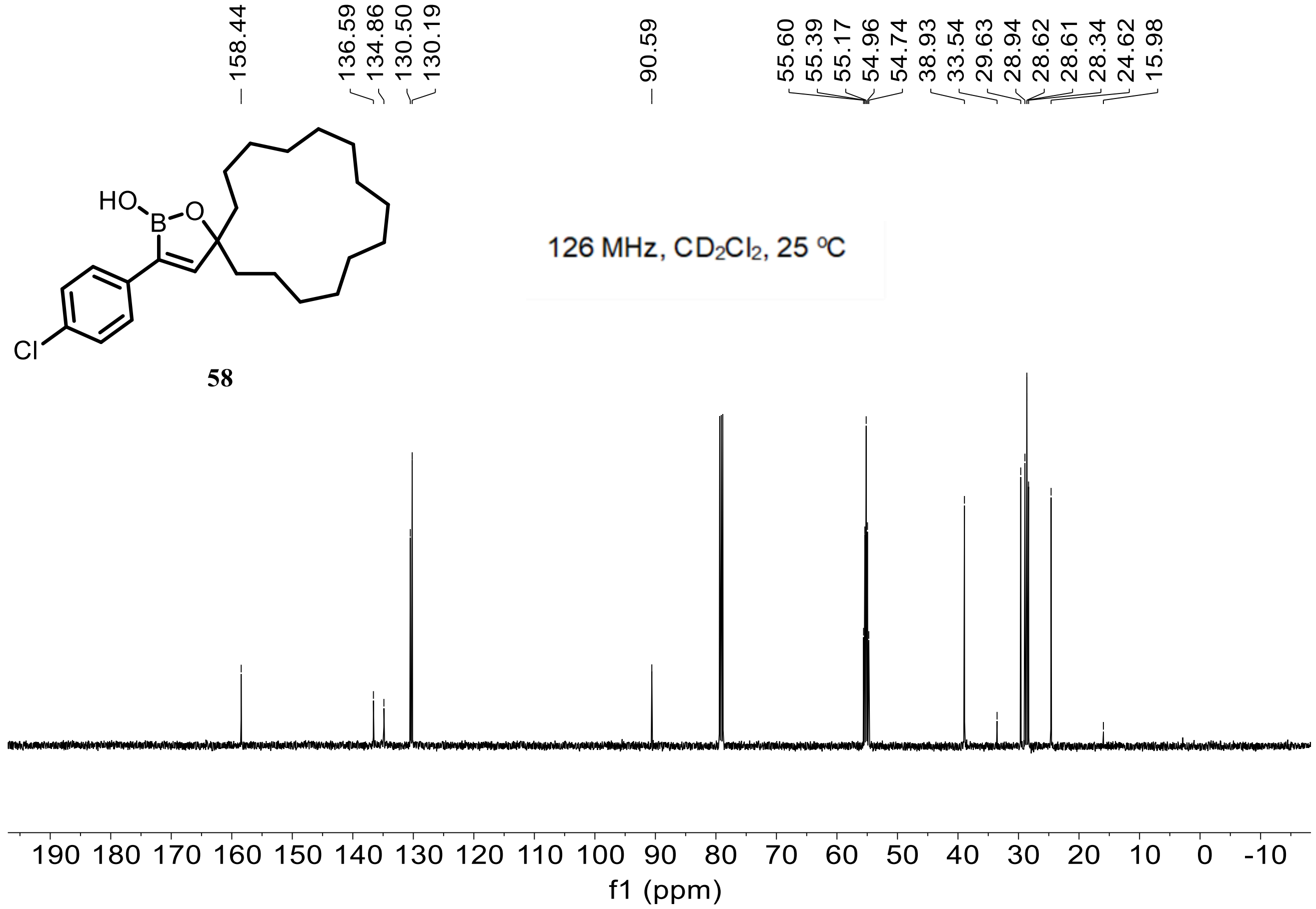
158.44
136.59
134.86
130.50
130.19
90.59
55.60
55.39
55.17
54.96
54.74
38.93
33.54
29.63
28.94
28.62
28.61
28.34
24.62
15.98
HO
B
O
Cl
58
126 MHz, CD2Cl2, 25 ºC
190 180 170 160 150 140 130 120 110 100 90 80 70 60 50 40 30 20 10 0 -10
f1 (ppm)

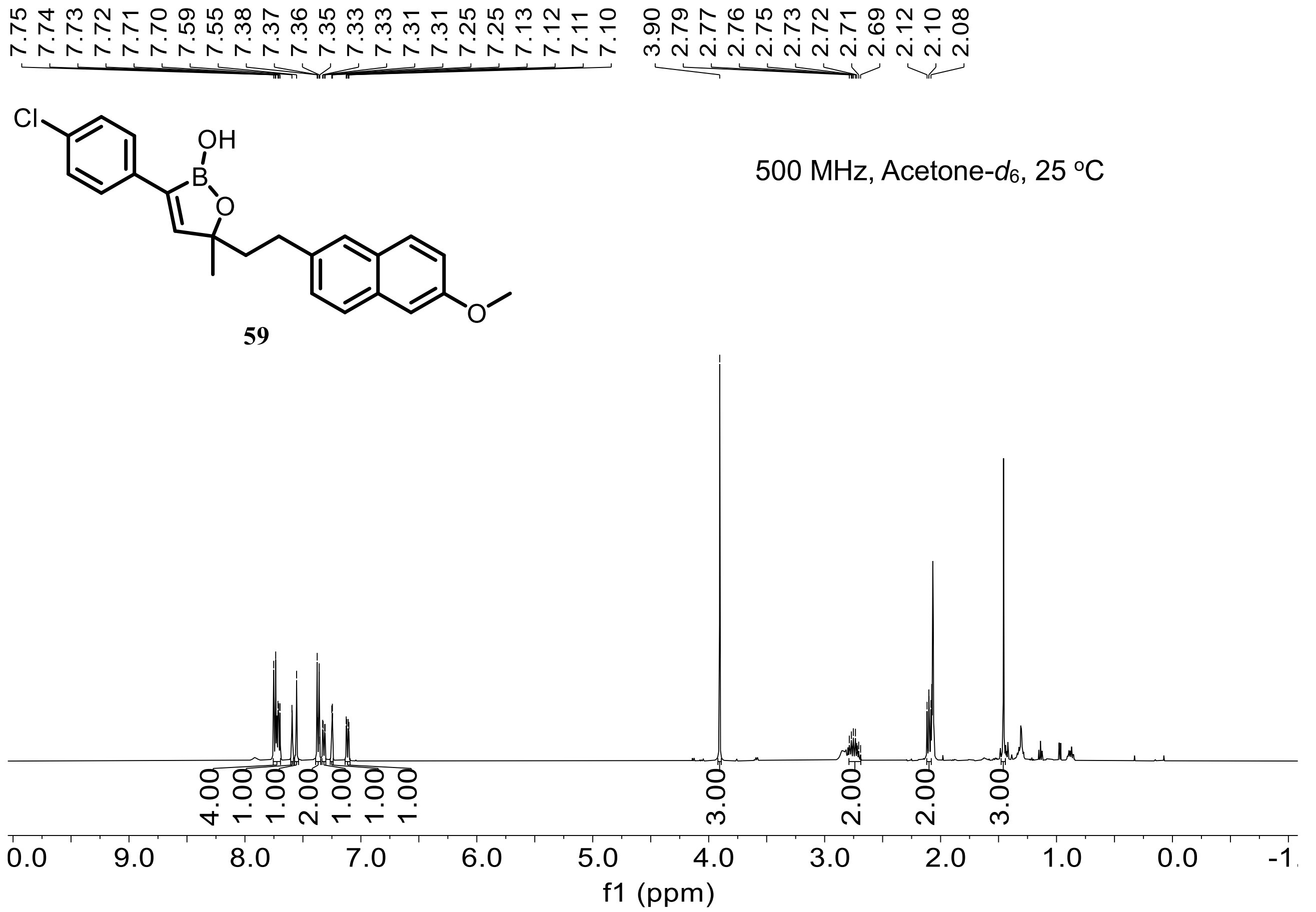
7.75 7.74 7.73 7.72 7.71 7.70 7.59 7.55 7.38 7.37 7.36 7.35 7.33 7.33 7.31 7.31 7.25 7.25 7.13 7.12 7.11 7.10
3.90 2.79 2.77 2.76 2.75 2.73 2.72 2.71 2.69 2.12 2.10 2.08
Cl
OH
B
O
59
500 MHz, Acetone-d_6, 25 °C
4.00 1.00 1.00 2.00 1.00 1.00 1.00
3.00
2.00
2.00
3.00
f1 (ppm)

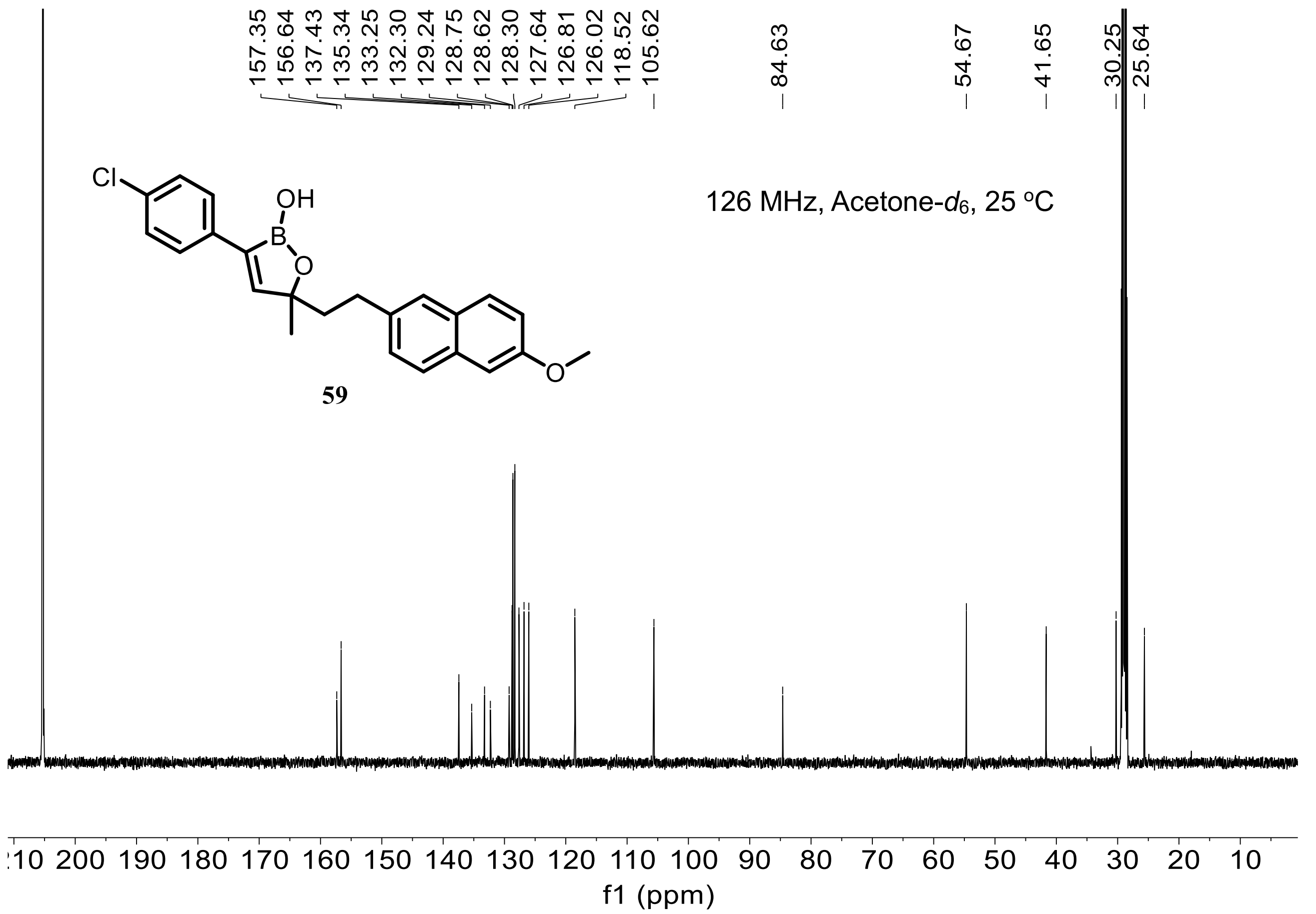
157.35 156.64 137.43 135.34 133.25 132.30 129.24 128.75 128.62 128.30 127.64 126.81 126.02 118.52 105.62
84.63
54.67
41.65
30.25
25.64
Cl
OH
B
O
59
126 MHz, Acetone-d_6, 25 °C
f1 (ppm)

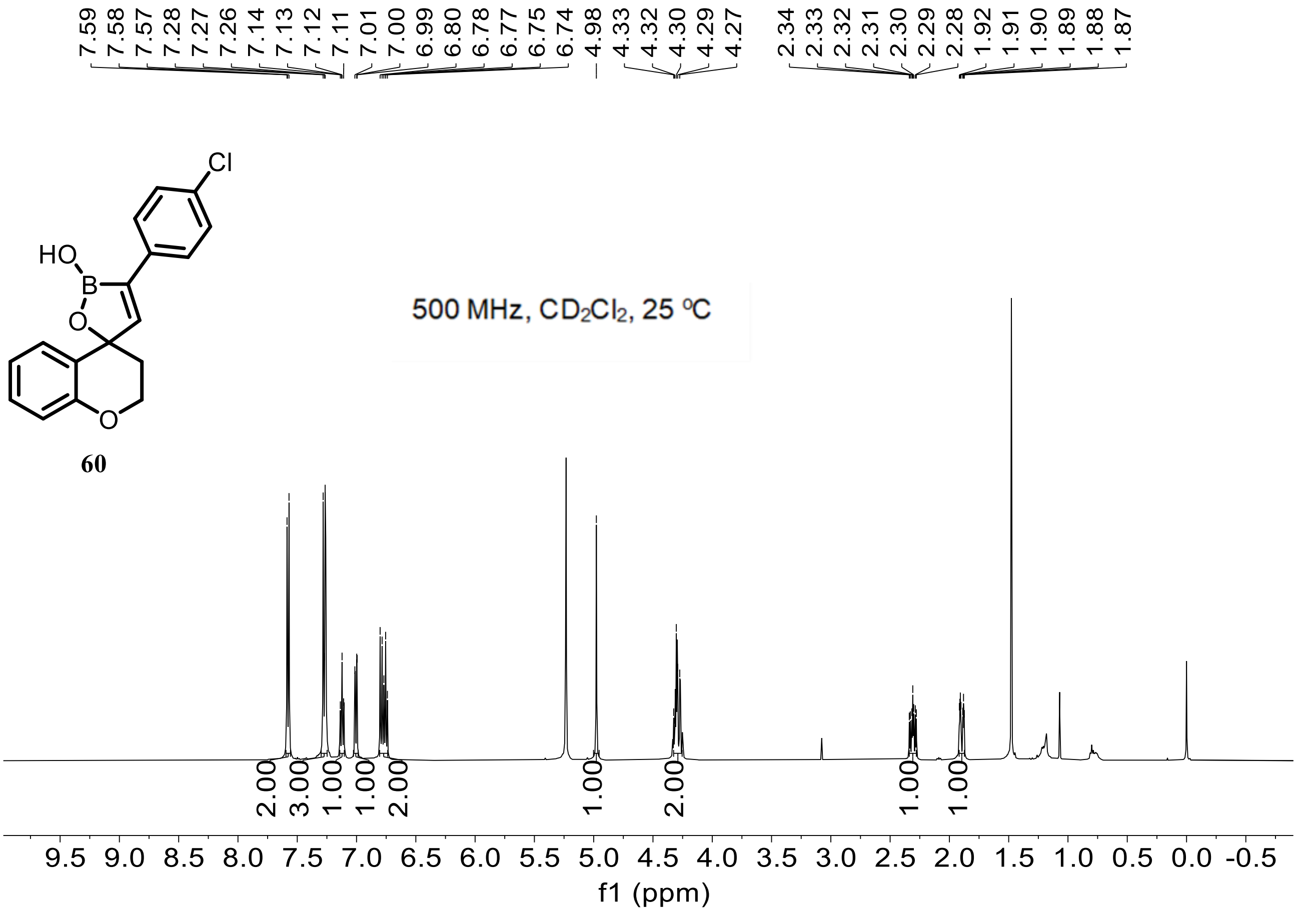
7.59 7.58 7.57 7.28 7.27 7.26 7.14 7.13 7.12 7.11 7.01 7.00 6.99 6.80 6.78 6.77 6.75 6.74 4.98 4.33 4.32 4.30 4.29 4.27 2.34 2.33 2.32 2.31 2.30 2.29 2.28 1.92 1.91 1.90 1.89 1.88 1.87
Cl
HO
B
O
O
60
500 MHz, CD2Cl2, 25 °C
2.00 3.00 1.00 1.00 2.00 1.00 2.00 1.00 1.00
9.5 9.0 8.5 8.0 7.5 7.0 6.5 6.0 5.5 5.0 4.5 4.0 3.5 3.0 2.5 2.0 1.5 1.0 0.5 0.0 -0.5
f1 (ppm)

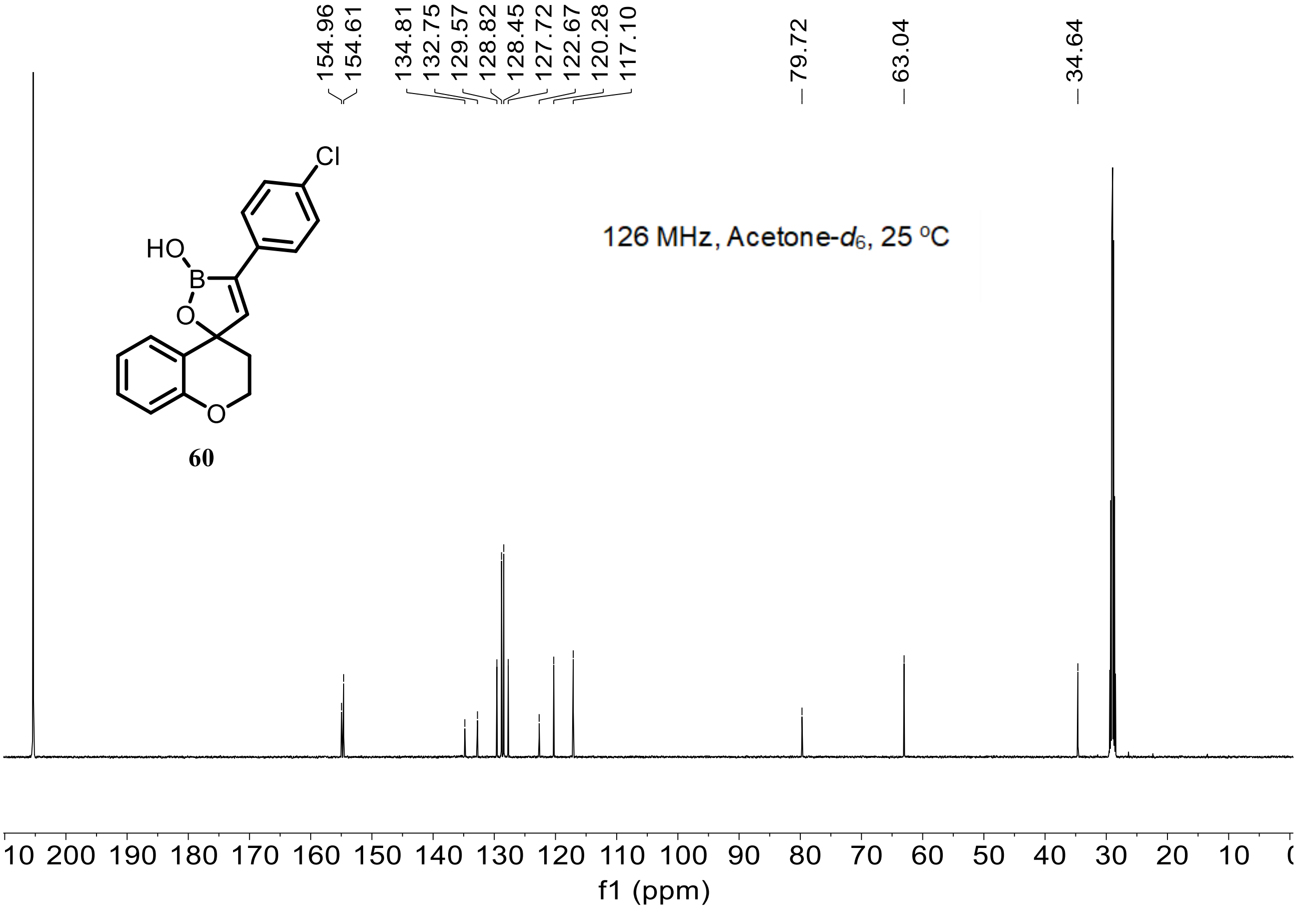
154.96 154.61 134.81 132.75 129.57 128.82 128.45 127.72 122.67 120.28 117.10 79.72 63.04 34.64
Cl
HO
B
O
O
60
126 MHz, Acetone-d6, 25 °C
10 200 190 180 170 160 150 140 130 120 110 100 90 80 70 60 50 40 30 20 10
f1 (ppm)

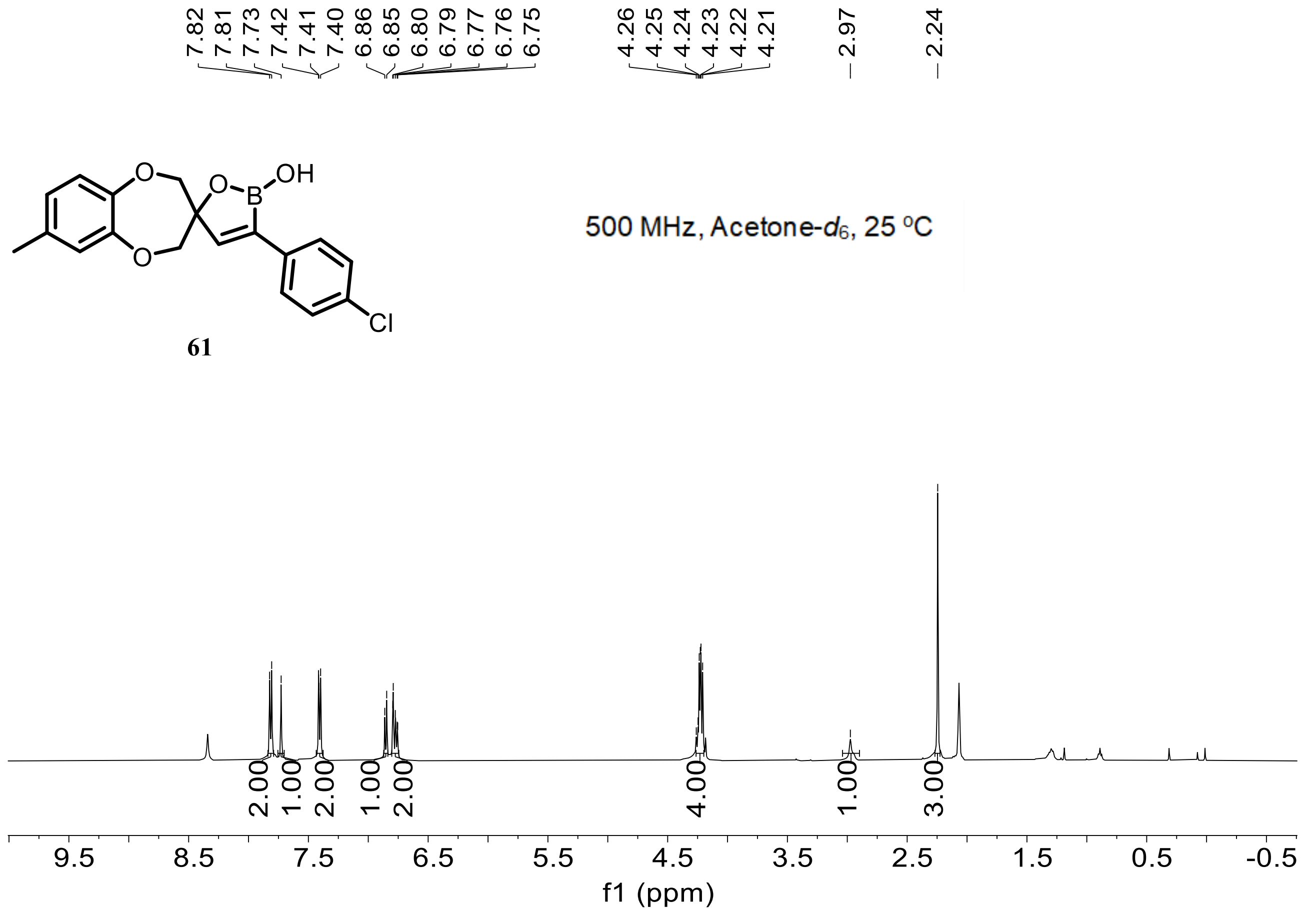
7.82 7.81 7.73 7.42 7.41 7.40 6.86 6.85 6.80 6.79 6.77 6.76 6.75
4.26 4.25 4.24 4.23 4.22 4.21
2.97
2.24
OH
O
B
O
O
Cl
61
500 MHz, Acetone-d_6, 25 °C
2.00 1.00 2.00 1.00 2.00
4.00
1.00
3.00
9.5 8.5 7.5 6.5 5.5 4.5 3.5 2.5 1.5 0.5 -0.5
f1 (ppm)

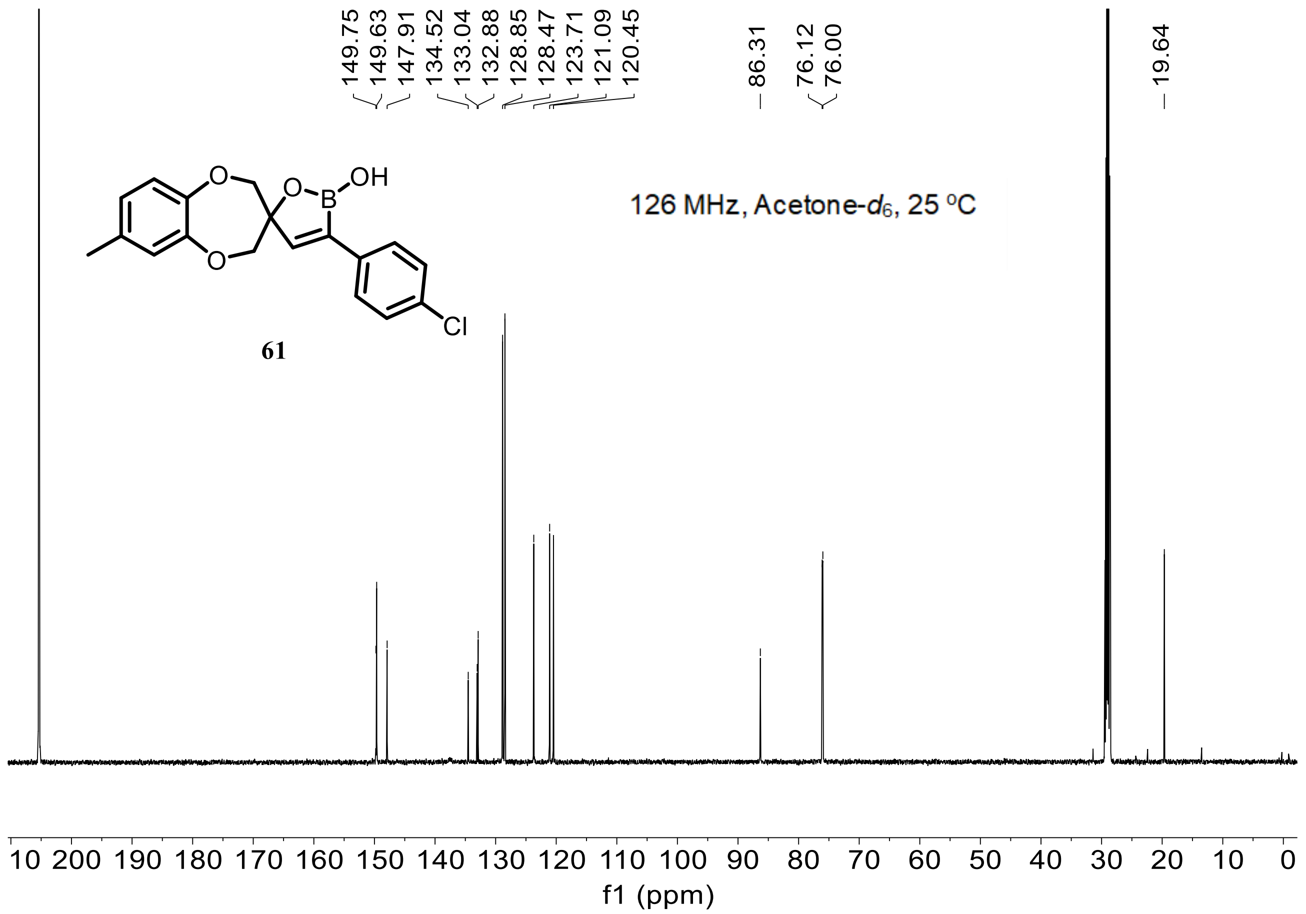
149.75 149.63 147.91 134.52 133.04 132.88 128.85 128.47 123.71 121.09 120.45
86.31
76.12 76.00
19.64
OH
O
B
O
O
Cl
61
126 MHz, Acetone-d_6, 25 °C
10 200 190 180 170 160 150 140 130 120 110 100 90 80 70 60 50 40 30 20 10 0
f1 (ppm)

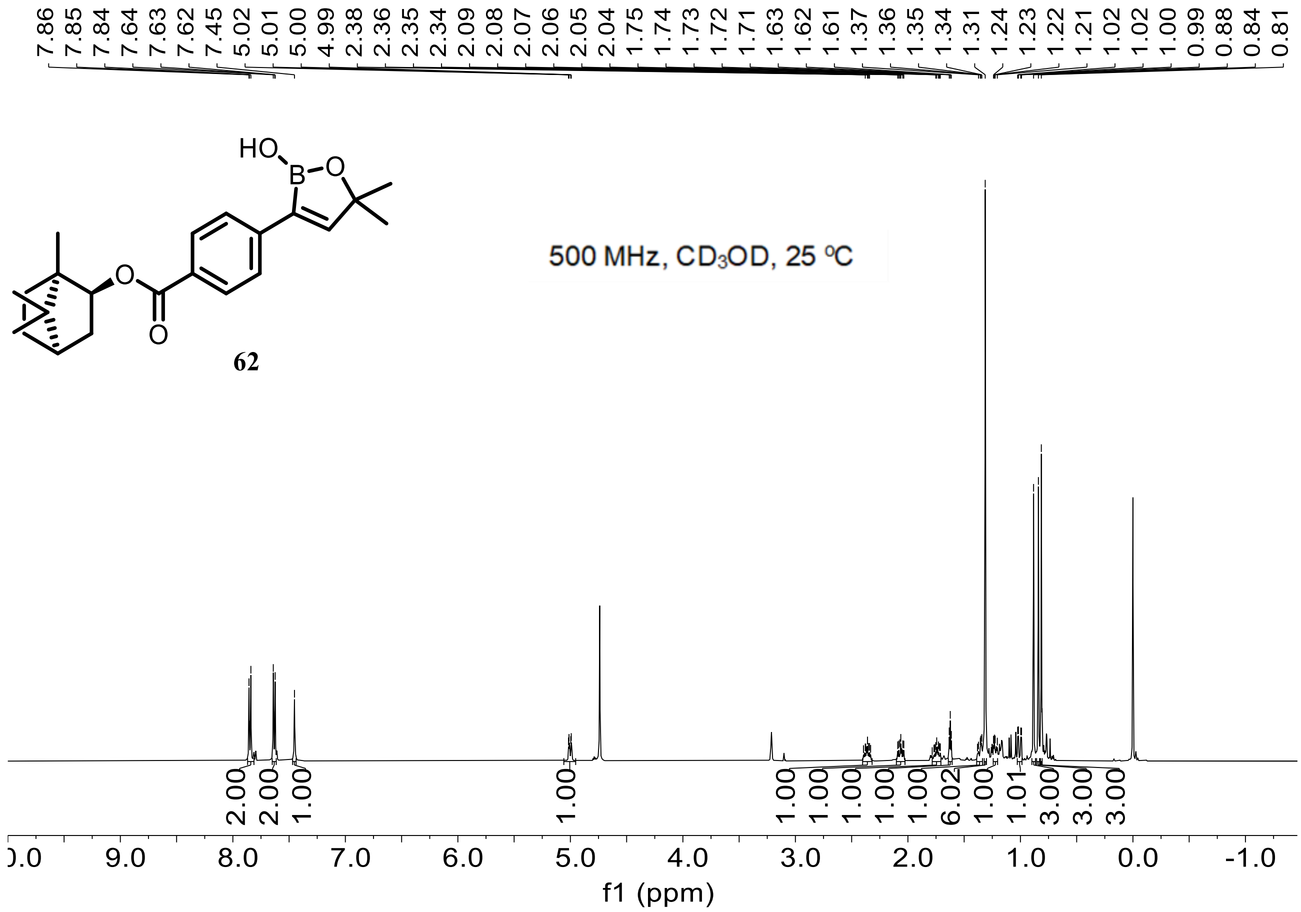
7.86 7.85 7.84 7.64 7.63 7.62 7.45 5.02 5.01 5.00 4.99 2.38 2.36 2.35 2.34 2.09 2.08 2.07 2.06 2.05 2.04 1.75 1.74 1.73 1.72 1.71 1.63 1.62 1.61 1.37 1.36 1.35 1.34 1.31 1.24 1.23 1.22 1.21 1.02 1.02 1.00 0.99 0.88 0.84 0.81
HO
B
O
O
O
62
500 MHz, CD3OD, 25 °C
2.00 2.00 1.00 1.00 1.00 1.00 1.00 1.00 1.00 6.02 1.00 1.01 3.00 3.00 3.00
9.0 8.0 7.0 6.0 5.0 4.0 3.0 2.0 1.0 0.0 -1.0
f1 (ppm)

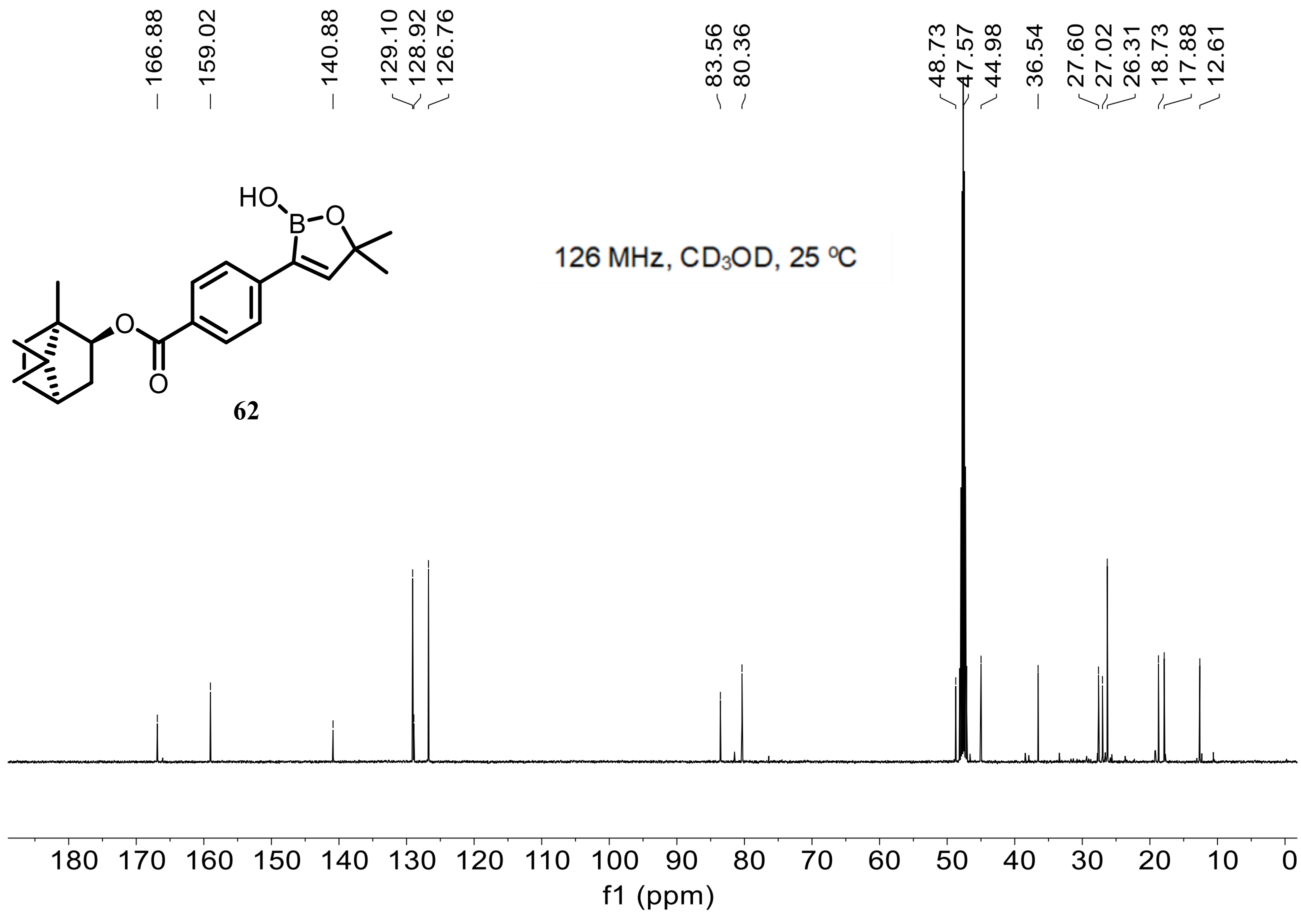
166.88 159.02 140.88 129.10 128.92 126.76 83.56 80.36 48.73 47.57 44.98 36.54 27.60 27.02 26.31 18.73 17.88 12.61
HO
B
O
O
O
62
126 MHz, CD3OD, 25 °C
180 170 160 150 140 130 120 110 100 90 80 70 60 50 40 30 20 10 0
f1 (ppm)

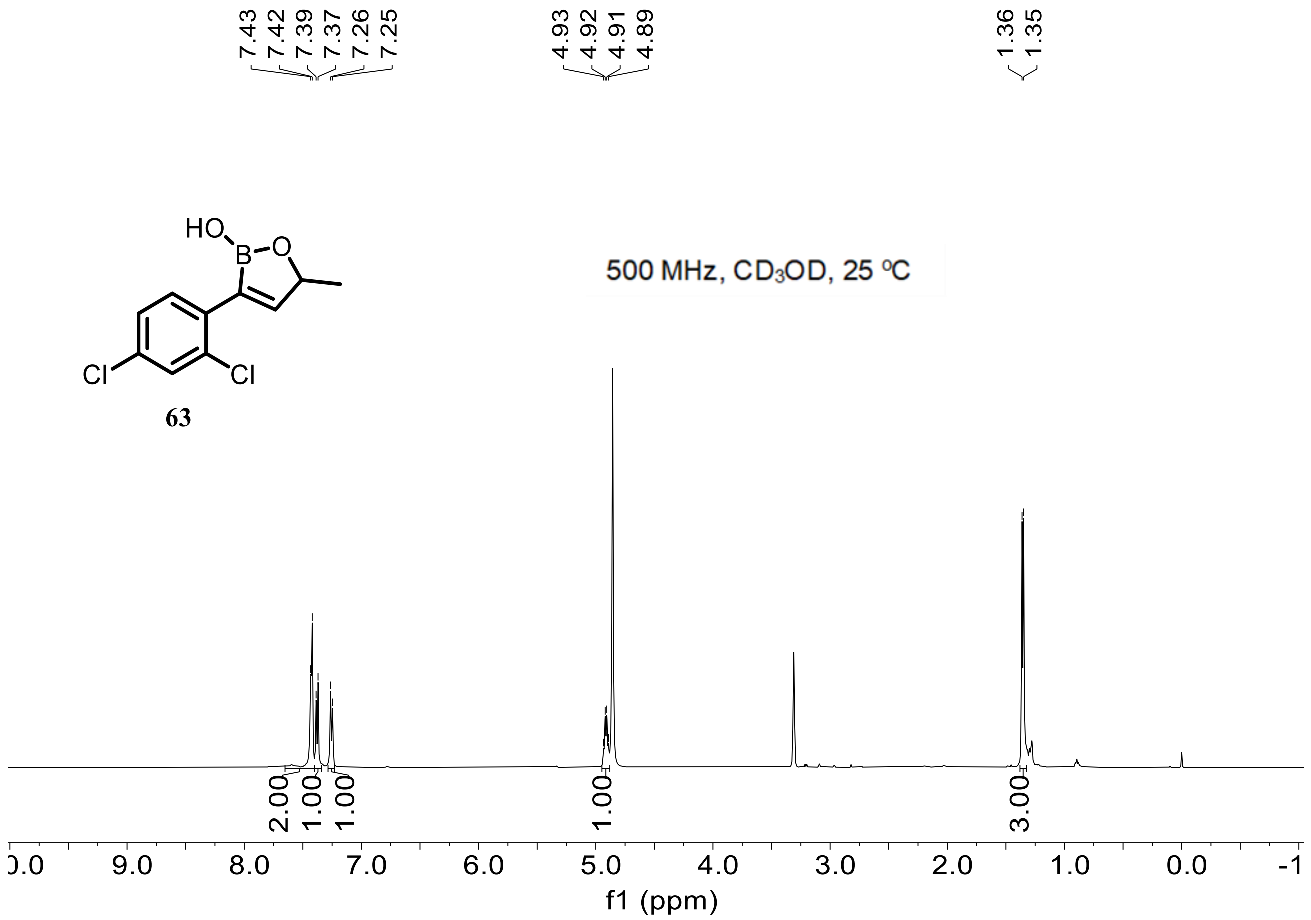
7.43
7.42
7.39
7.37
7.26
7.25
4.93
4.92
4.91
4.89
1.36
1.35
HO
B
O
Cl
Cl
63
500 MHz, CD3OD, 25 ºC
2.00
1.00
1.00
1.00
3.00
f1 (ppm)

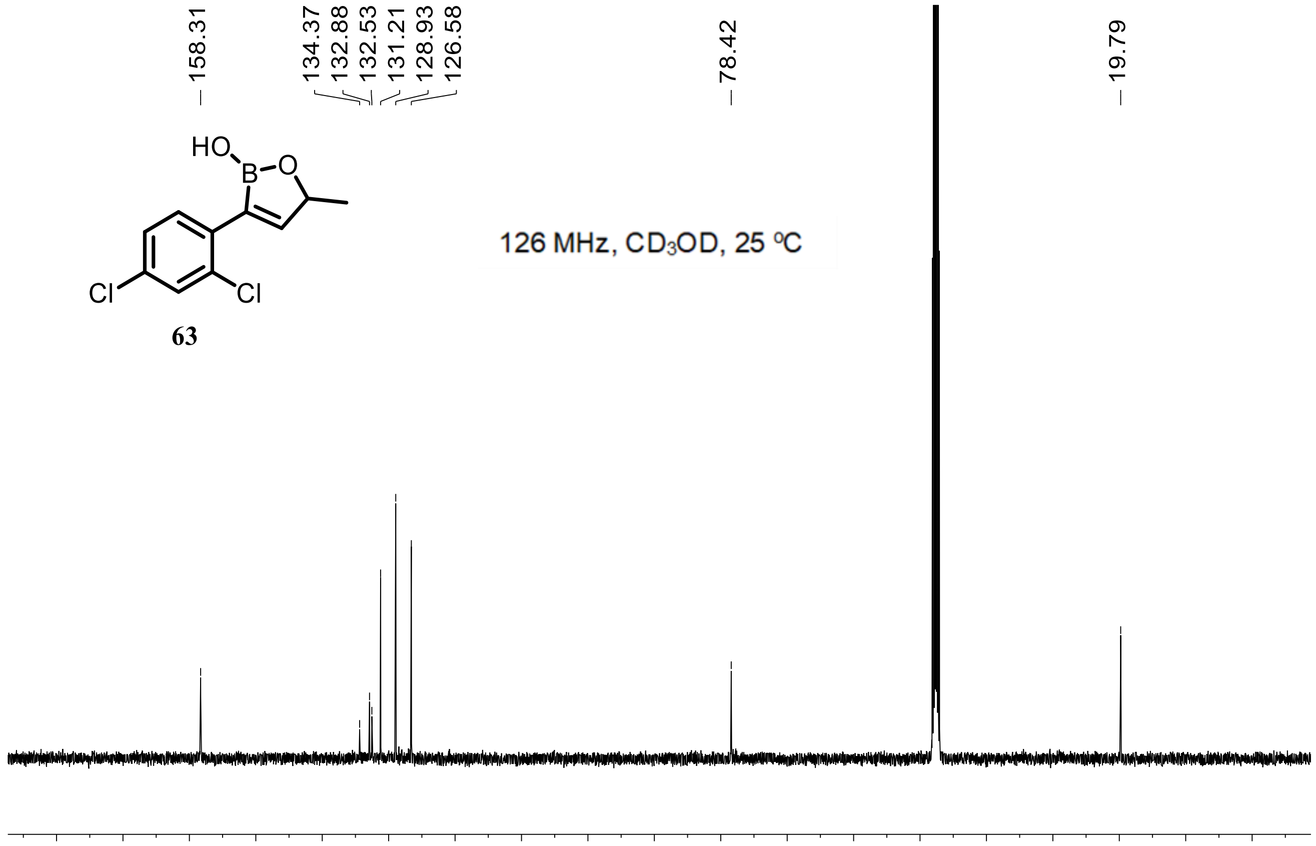
158.31
134.37
132.88
132.53
131.21
128.93
126.58
78.42
19.79
HO
B
O
Cl
Cl
63
126 MHz, CD3OD, 25 ºC
f1 (ppm)

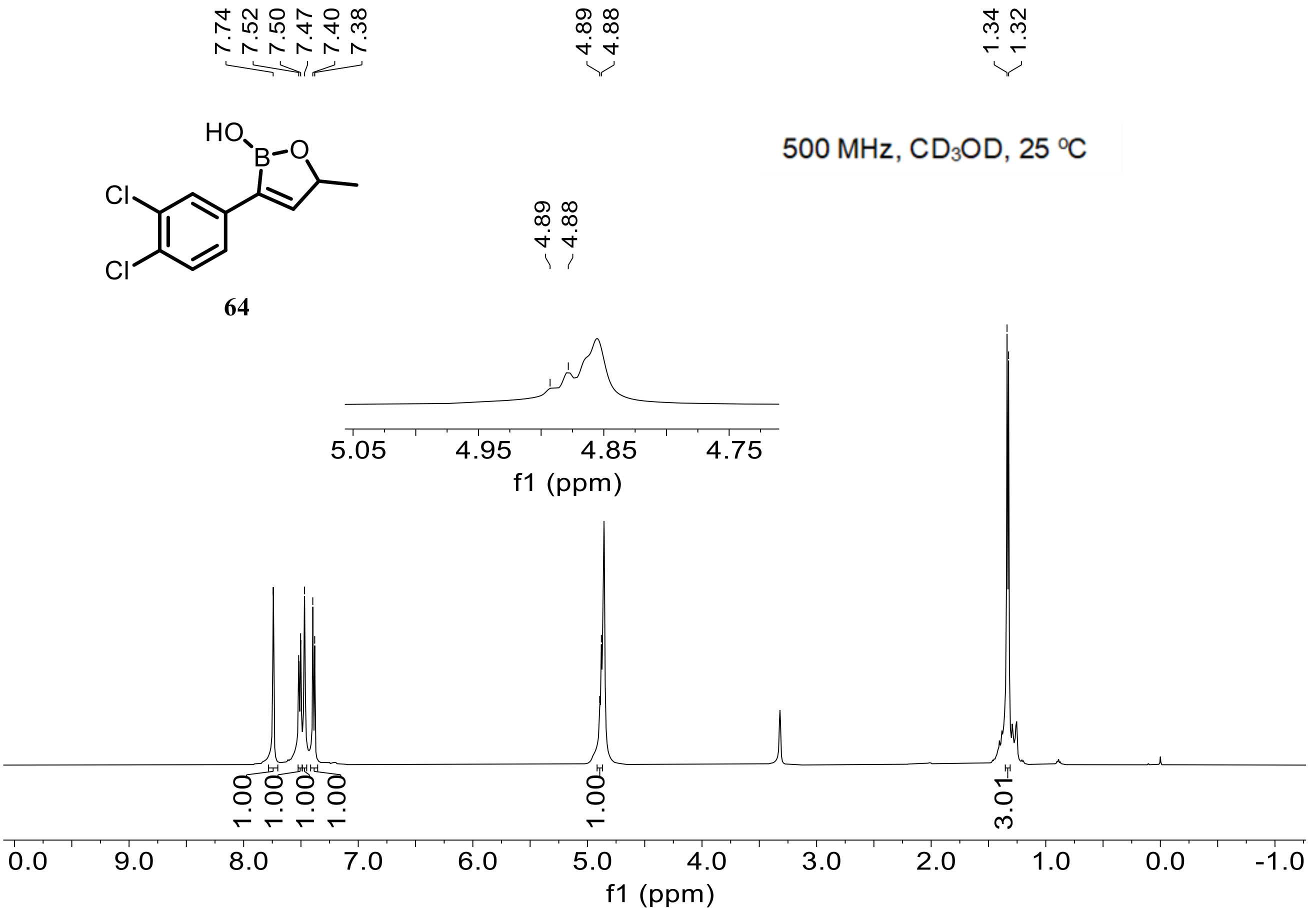
7.74
7.52
7.50
7.47
7.40
7.38
4.89
4.88
1.34
1.32
HO
B
O
Cl
Cl
64
500 MHz, CD3OD, 25 °C
5.05
4.95
4.85
4.75
f1 (ppm)
1.00
1.00
1.00
1.00
1.00
3.01
10.0
9.0
8.0
7.0
6.0
5.0
4.0
3.0
2.0
1.0
0.0
-1.0
f1 (ppm)

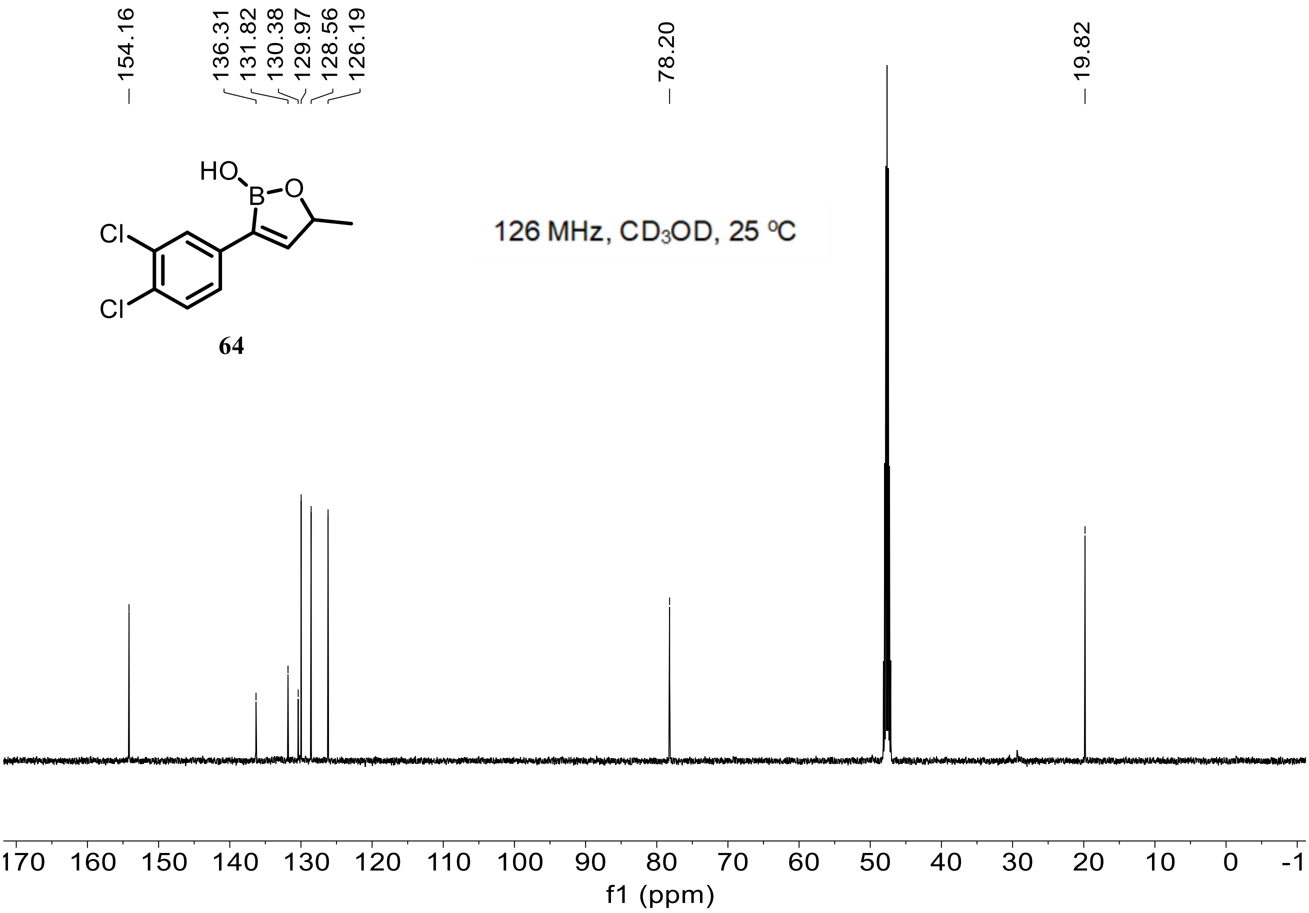
154.16
136.31
131.82
130.38
129.97
128.56
126.19
78.20
19.82
HO
B
O
Cl
Cl
64
126 MHz, CD3OD, 25 °C
170
160
150
140
130
120
110
100
90
80
70
60
50
40
30
20
10
0
-1
f1 (ppm)

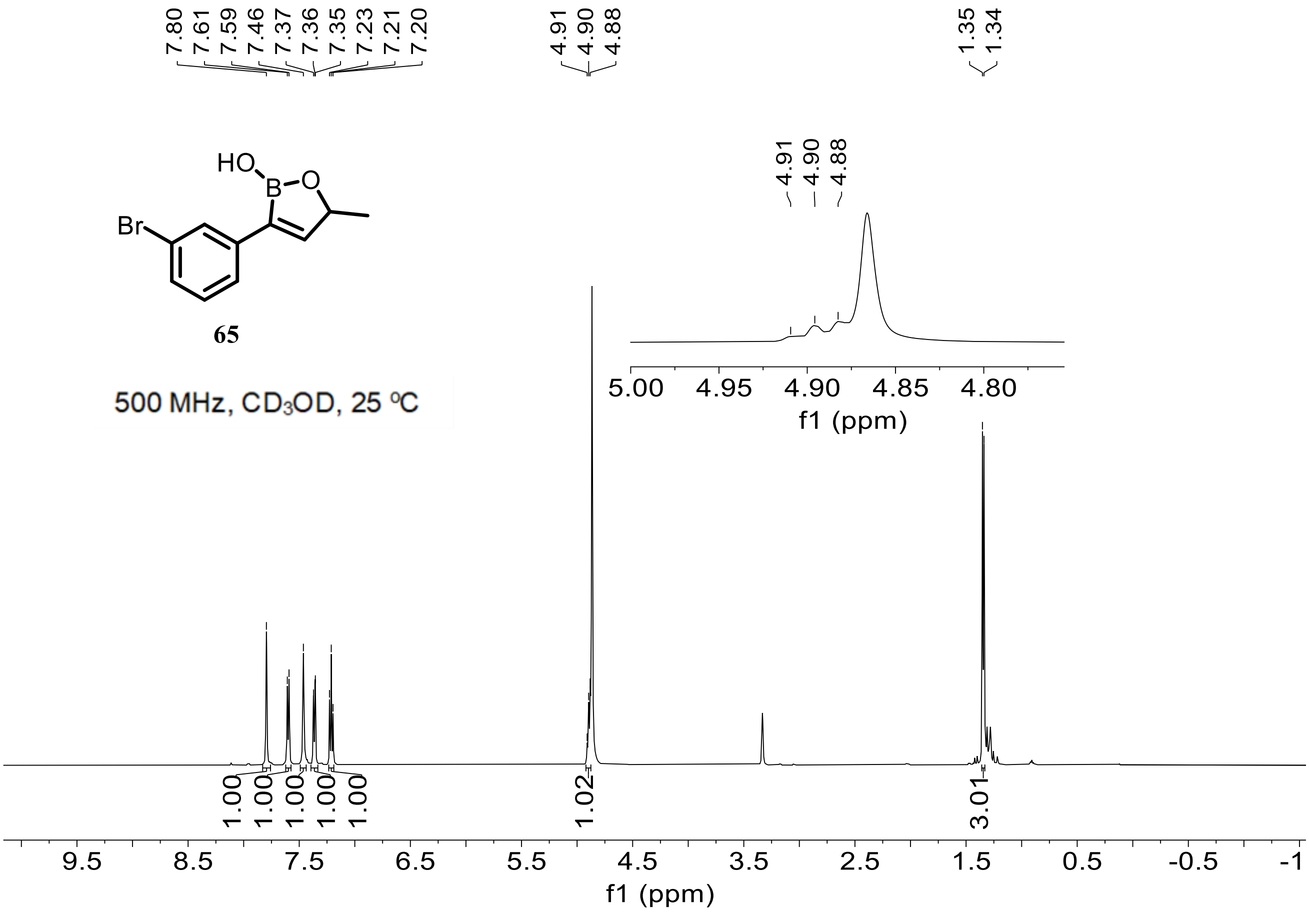
7.80 7.61 7.59 7.46 7.37 7.36 7.35 7.23 7.21 7.20
4.91 4.90 4.88
1.35 1.34
HO B O
Br
65
500 MHz, CD3OD, 25 °C
4.91 4.90 4.88
5.00 4.95 4.90 4.85 4.80
f1 (ppm)
1.00 1.00 1.00 1.00 1.00
1.02
3.01
9.5 8.5 7.5 6.5 5.5 4.5 3.5 2.5 1.5 0.5 -0.5 -1
f1 (ppm)

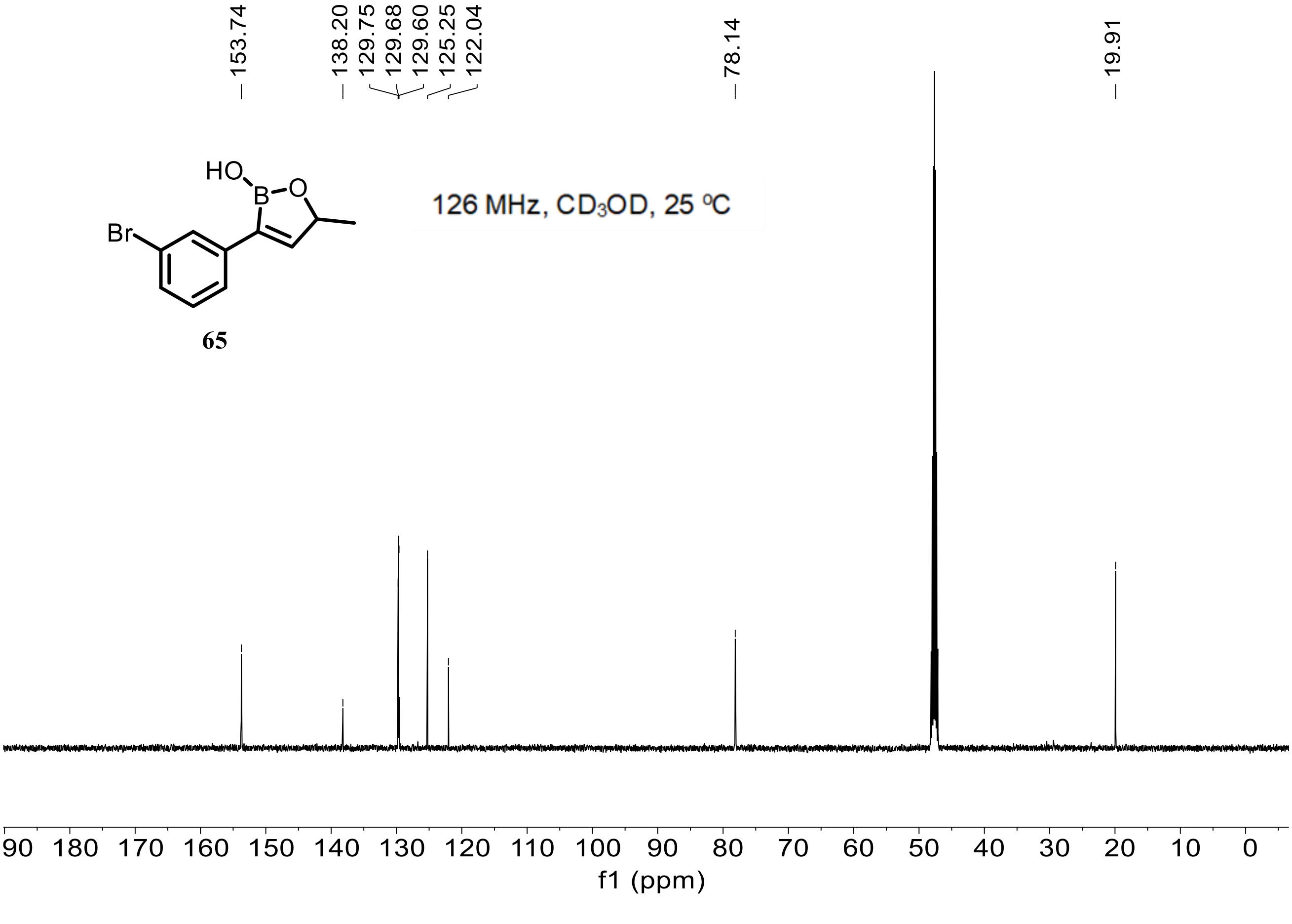
153.74
138.20 129.75 129.68 129.60 125.25 122.04
78.14
19.91
HO B O
Br
65
126 MHz, CD3OD, 25 °C
90 180 170 160 150 140 130 120 110 100 90 80 70 60 50 40 30 20 10 0
f1 (ppm)

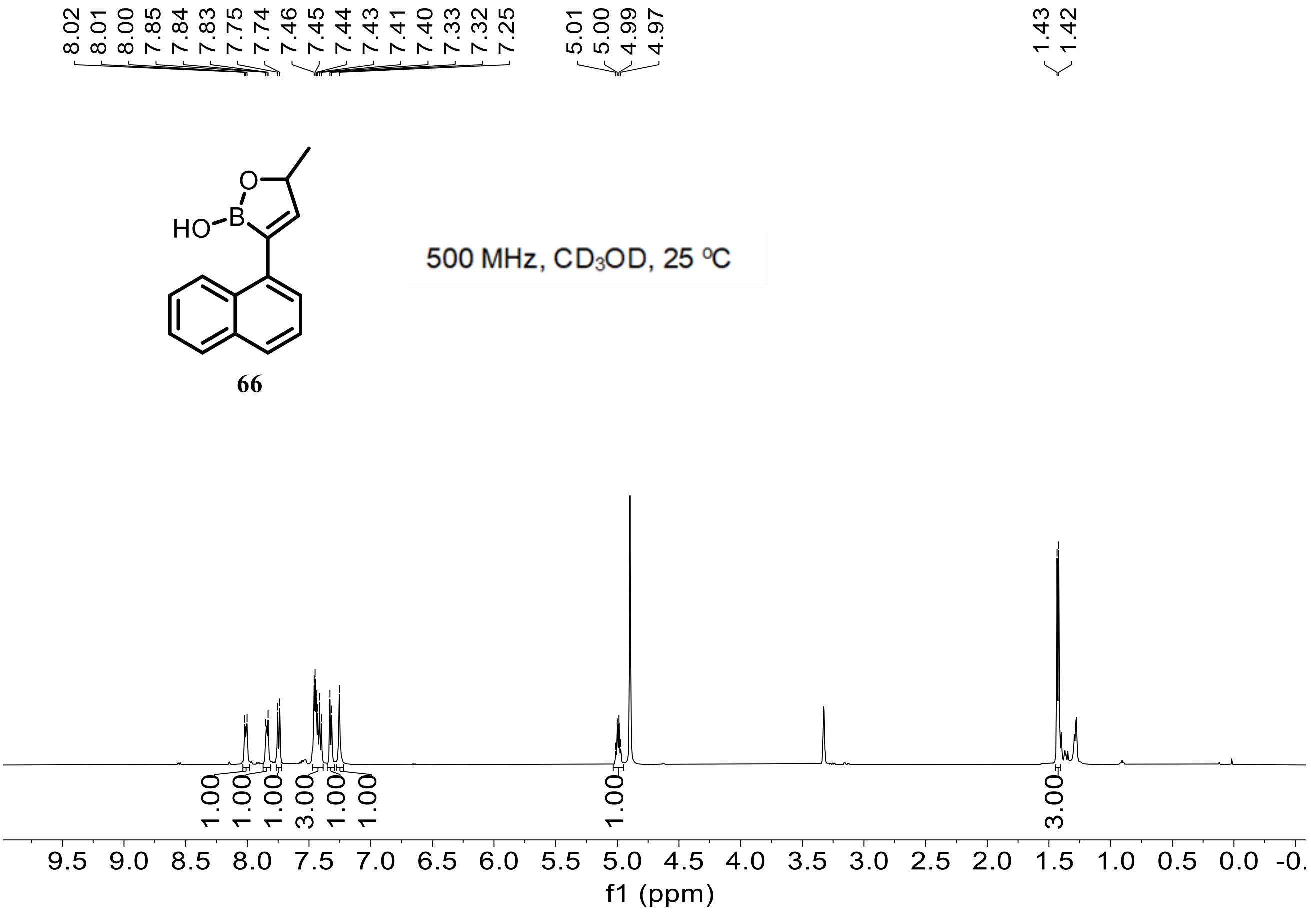
8.02 8.01 8.00 7.85 7.84 7.83 7.75 7.74 7.46 7.45 7.44 7.43 7.41 7.40 7.33 7.32 7.25
5.01 5.00 4.99 4.97
1.43 1.42
HO
B
O
66
500 MHz, CD3OD, 25 °C
1.00 1.00 1.00 3.00 1.00 1.00
1.00
3.00
9.5 9.0 8.5 8.0 7.5 7.0 6.5 6.0 5.5 5.0 4.5 4.0 3.5 3.0 2.5 2.0 1.5 1.0 0.5 0.0
f1 (ppm)

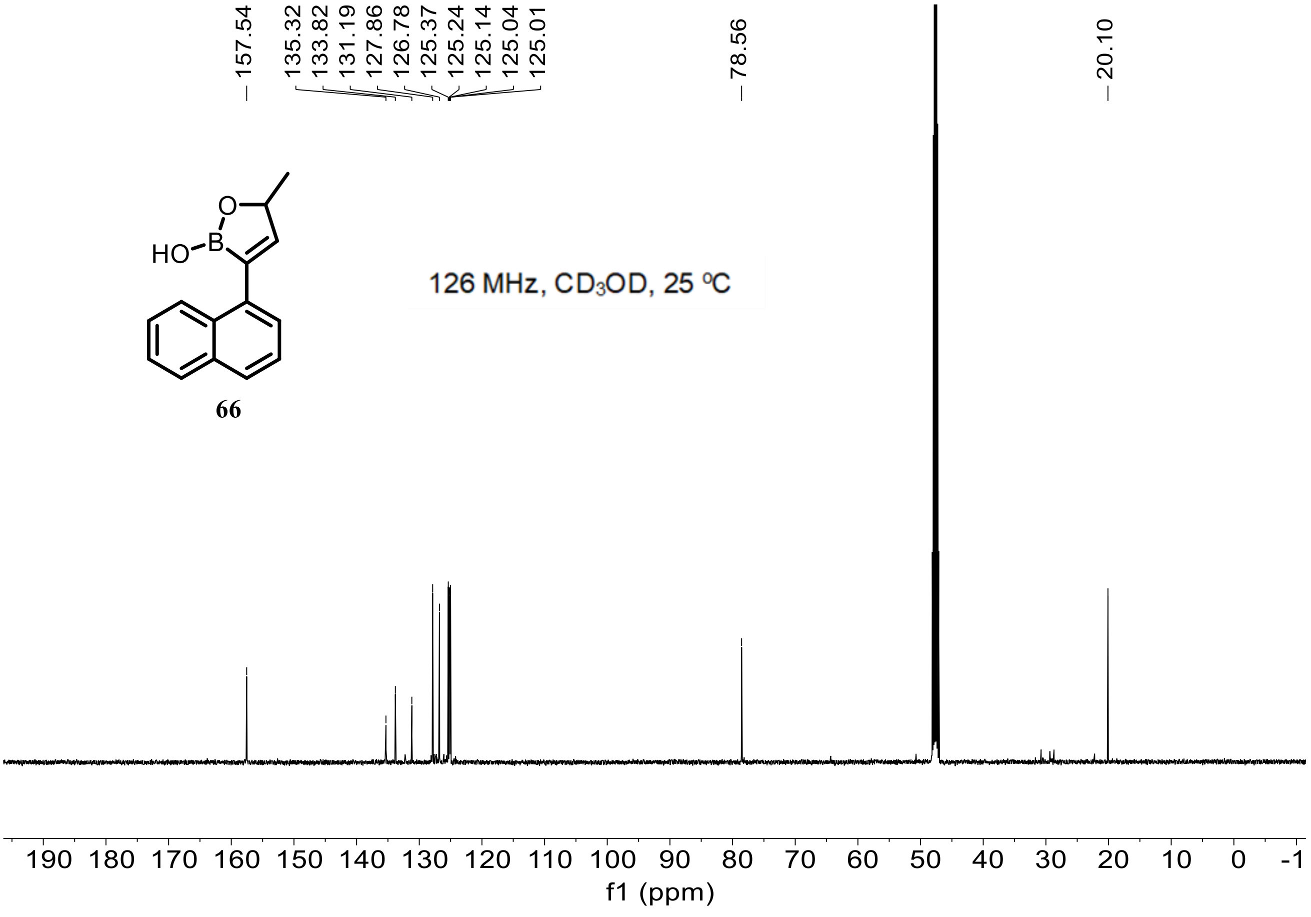
157.54
135.32 133.82 131.19 127.86 126.78 125.37 125.24 125.14 125.04 125.01
78.56
20.10
HO
B
O
66
126 MHz, CD3OD, 25 °C
190 180 170 160 150 140 130 120 110 100 90 80 70 60 50 40 30 20 10 0 -1
f1 (ppm)

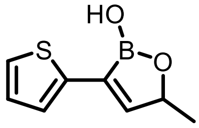

67

500 MHz, CD_3OD, 25 °C

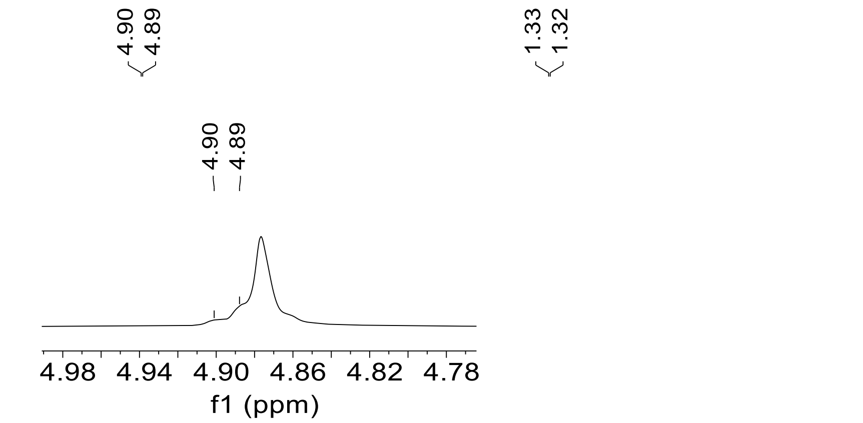

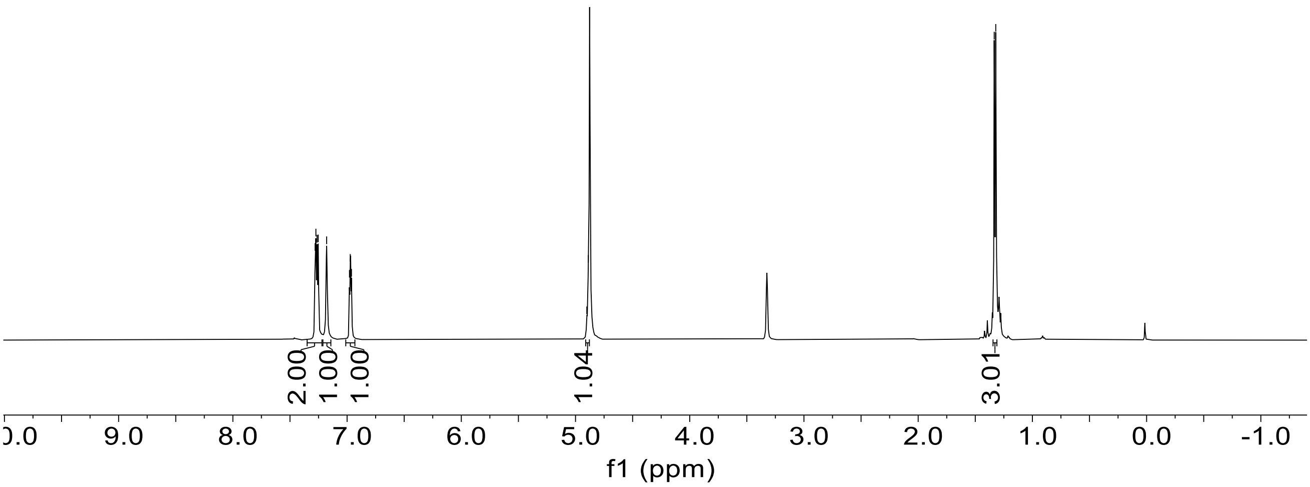

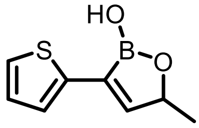

67

126 MHz, CD_3OD, 25 °C

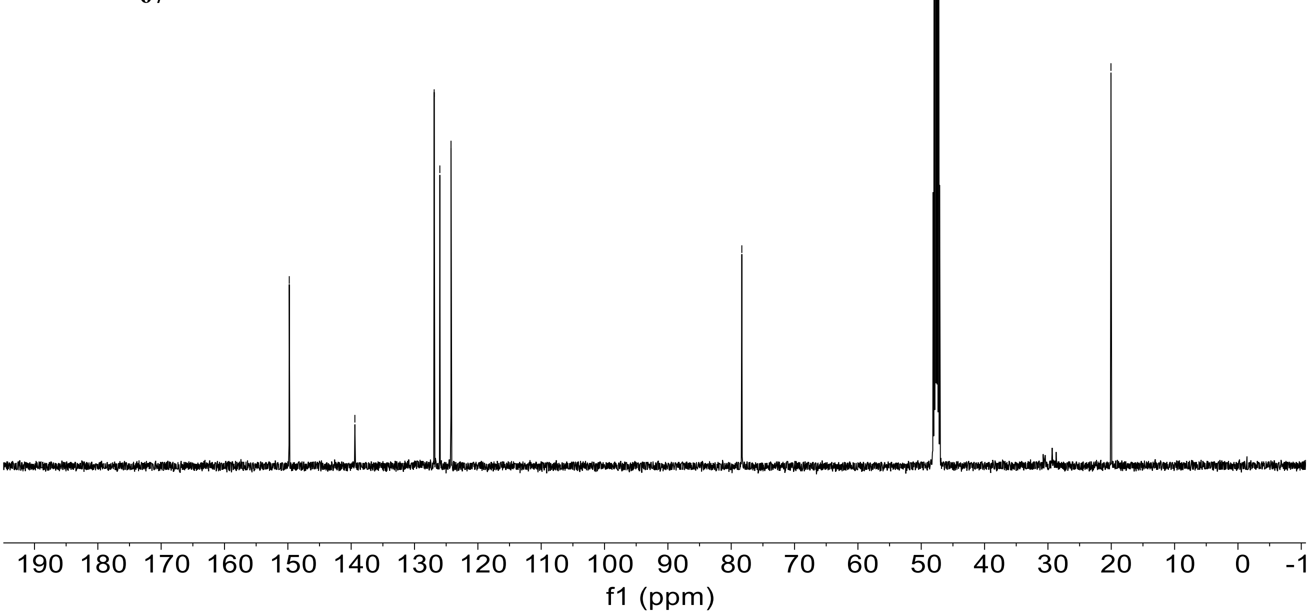

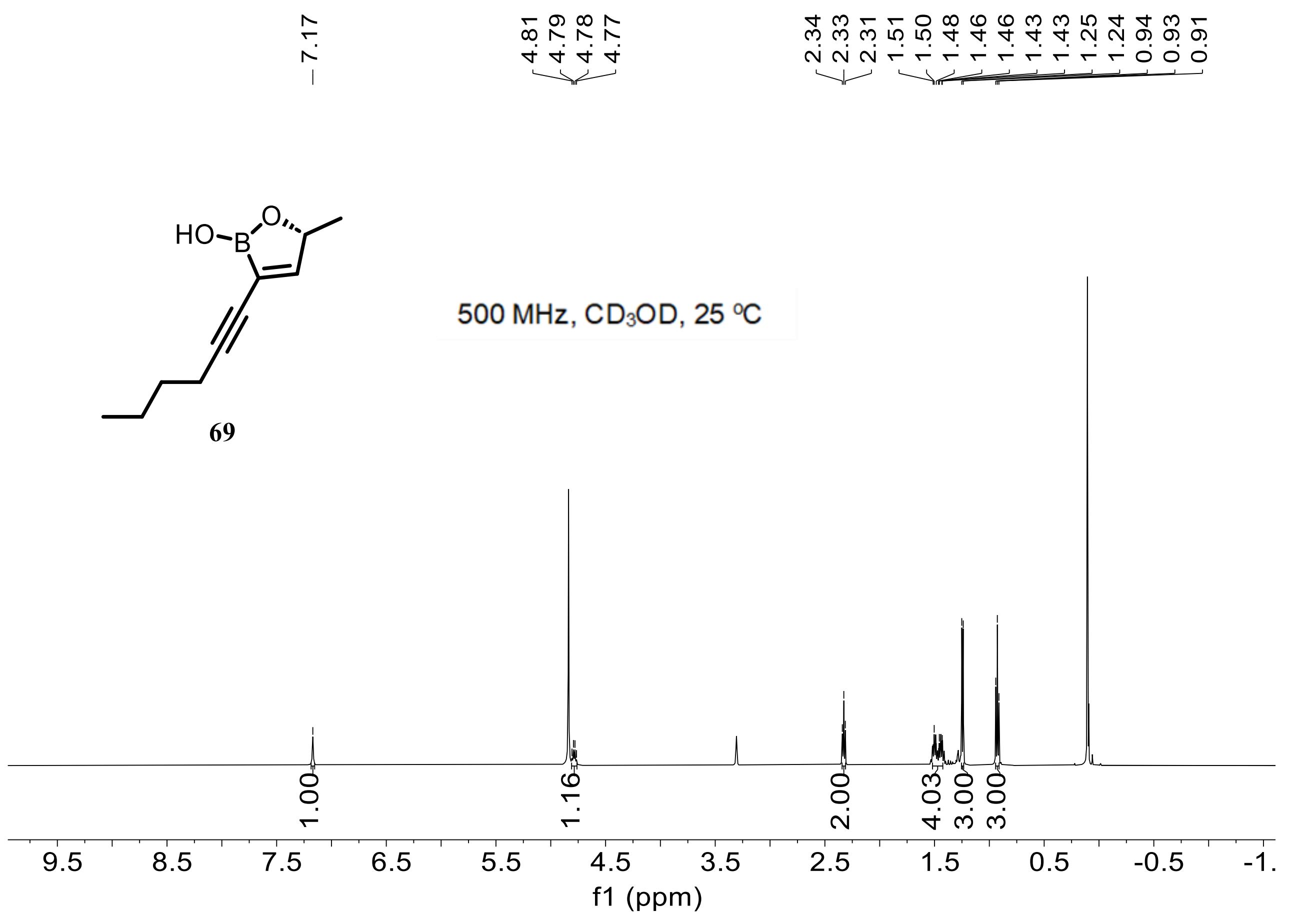

7.17
4.81
4.79
4.78
4.77
2.34
2.33
2.31
1.51
1.50
1.48
1.46
1.46
1.43
1.43
1.25
1.24
0.94
0.93
0.91
HO
B
O
69
500 MHz, CD3OD, 25 °C
1.00
1.16
2.00
4.03
3.00
3.00
9.5
8.5
7.5
6.5
5.5
4.5
3.5
2.5
1.5
0.5
-0.5
f1 (ppm)

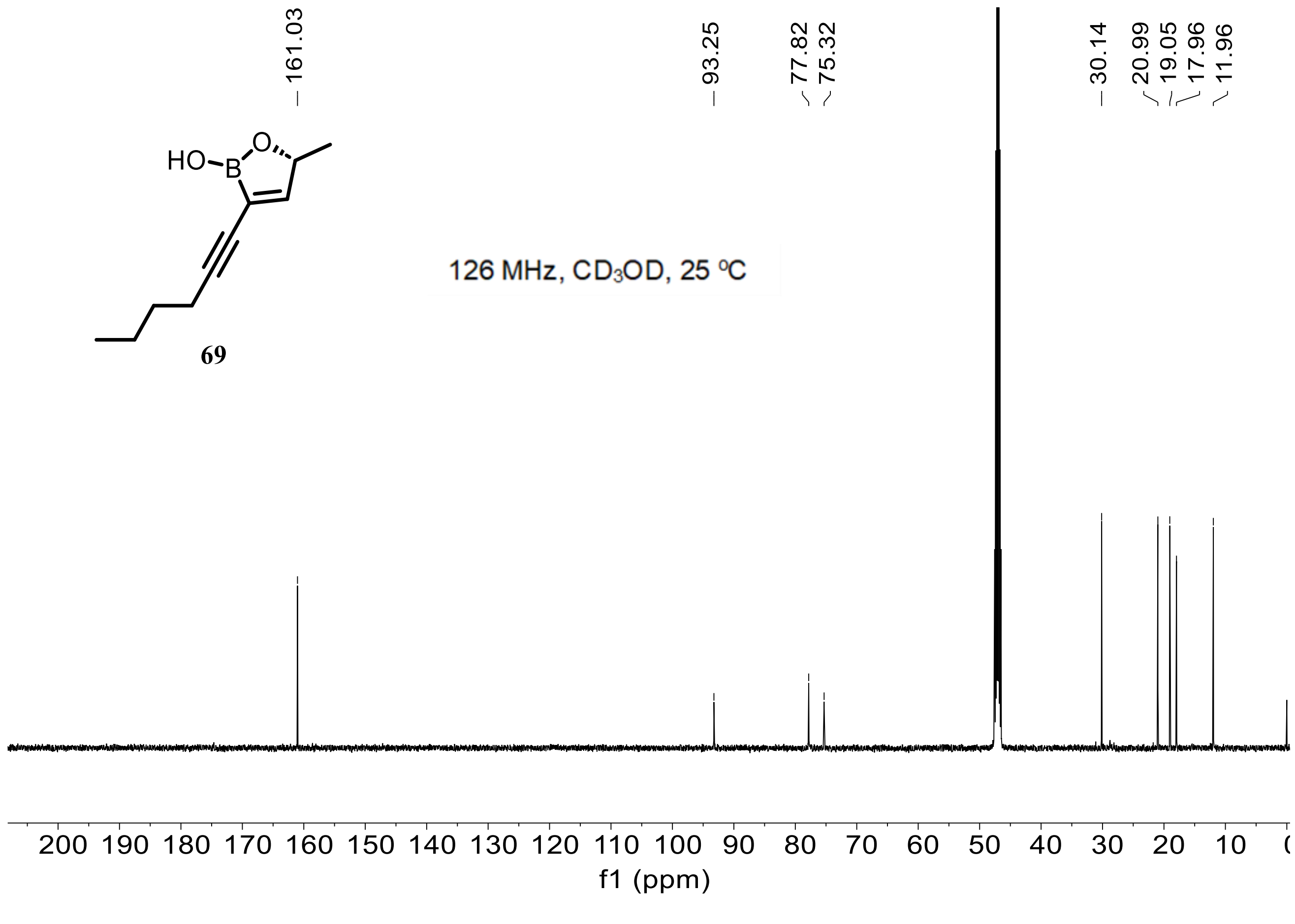

161.03
93.25
77.82
75.32
30.14
20.99
19.05
17.96
11.96
HO
B
O
69
126 MHz, CD3OD, 25 °C
200
190
180
170
160
150
140
130
120
110
100
90
80
70
60
50
40
30
20
10
f1 (ppm)

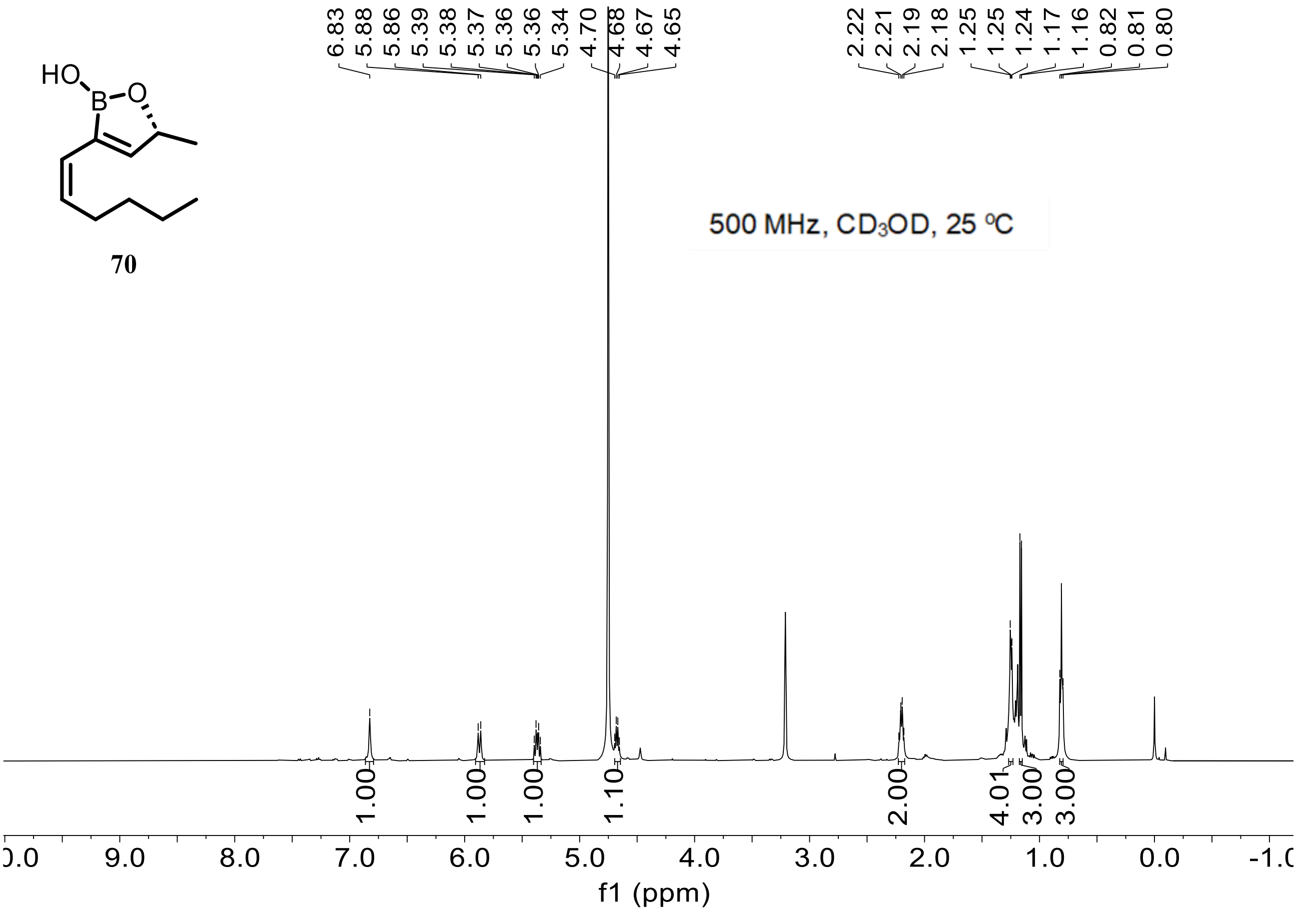
6.83 5.88 5.86 5.39 5.38 5.37 5.36 5.36 5.34 4.70 4.68 4.67 4.65
2.22 2.21 2.19 2.18 1.25 1.25 1.24 1.17 1.16 0.82 0.81 0.80
HO
B
O
70
500 MHz, CD3OD, 25 °C
1.00 1.00 1.00 1.10 2.00 4.01 3.00 3.00
9.0 8.0 7.0 6.0 5.0 4.0 3.0 2.0 1.0 0.0
f1 (ppm)

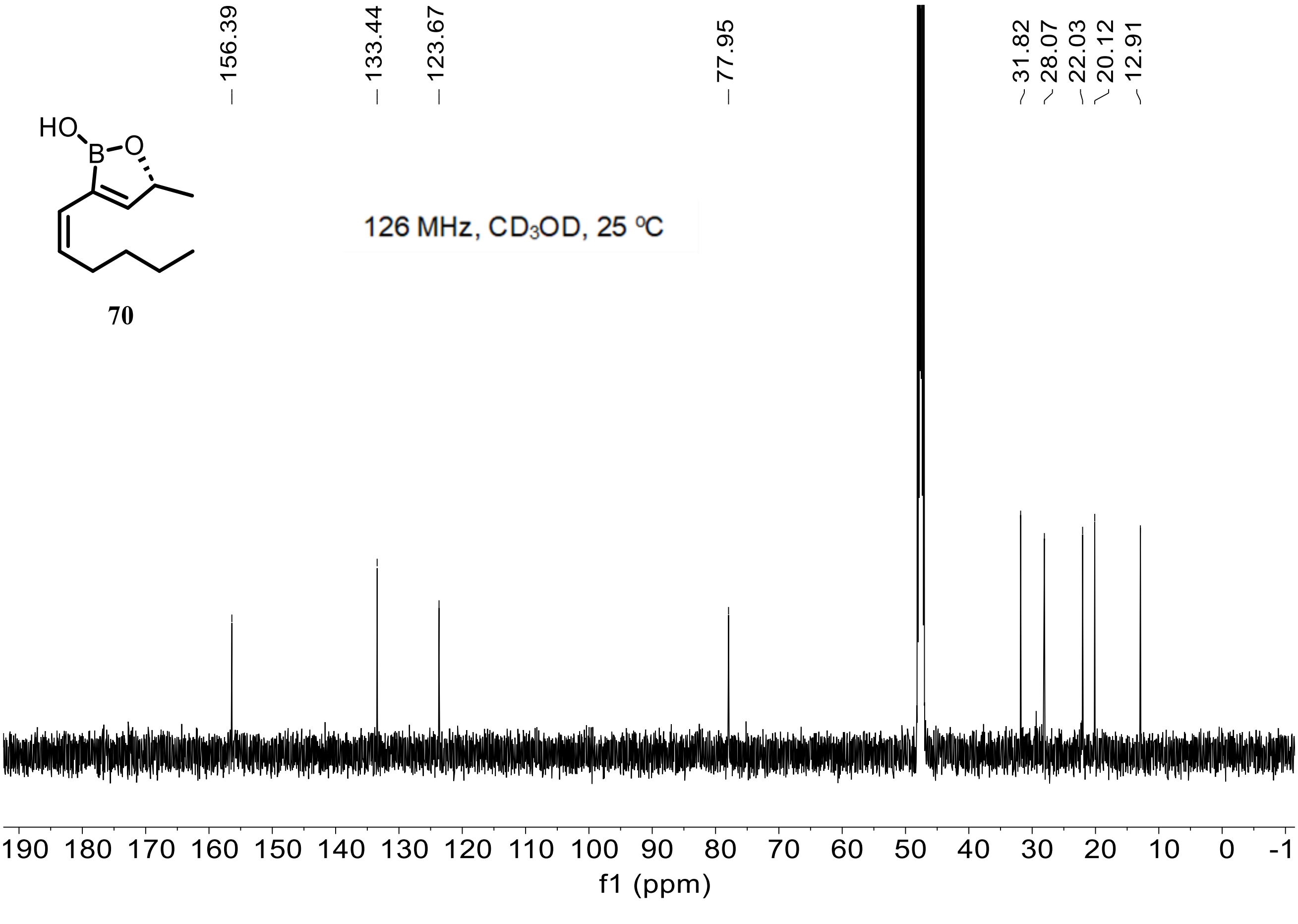
156.39
133.44
123.67
77.95
31.82 28.07 22.03 20.12 12.91
HO
B
O
70
126 MHz, CD3OD, 25 °C
190 180 170 160 150 140 130 120 110 100 90 80 70 60 50 40 30 20 10 0 -1
f1 (ppm)

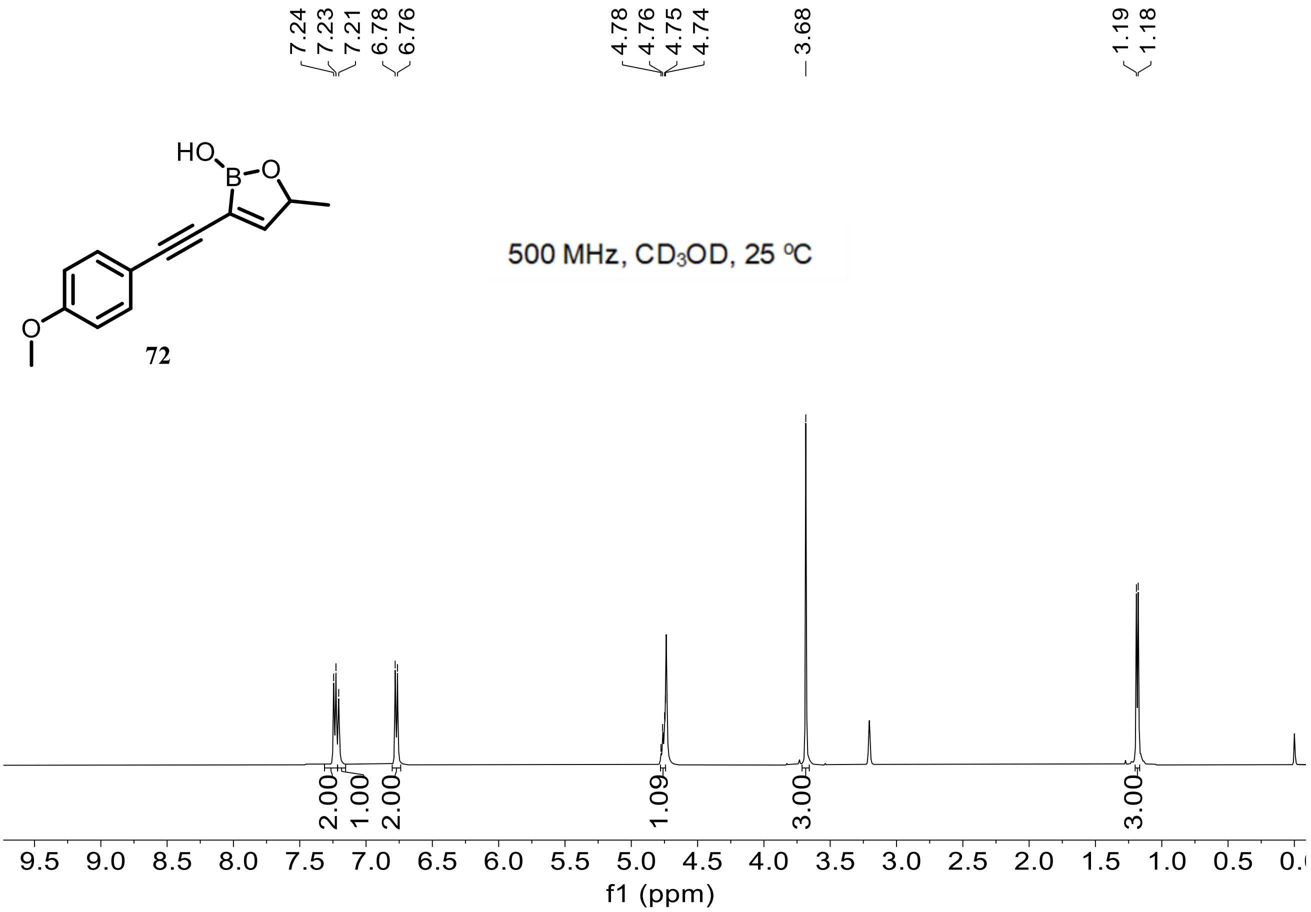

7.24
7.23
7.21
6.78
6.76
4.78
4.76
4.75
4.74
3.68
1.19
1.18
HO
B
O
72
500 MHz, CD3OD, 25 ºC
2.00
1.00
2.00
1.09
3.00
3.00
9.5 9.0 8.5 8.0 7.5 7.0 6.5 6.0 5.5 5.0 4.5 4.0 3.5 3.0 2.5 2.0 1.5 1.0 0.5 0.0
f1 (ppm)

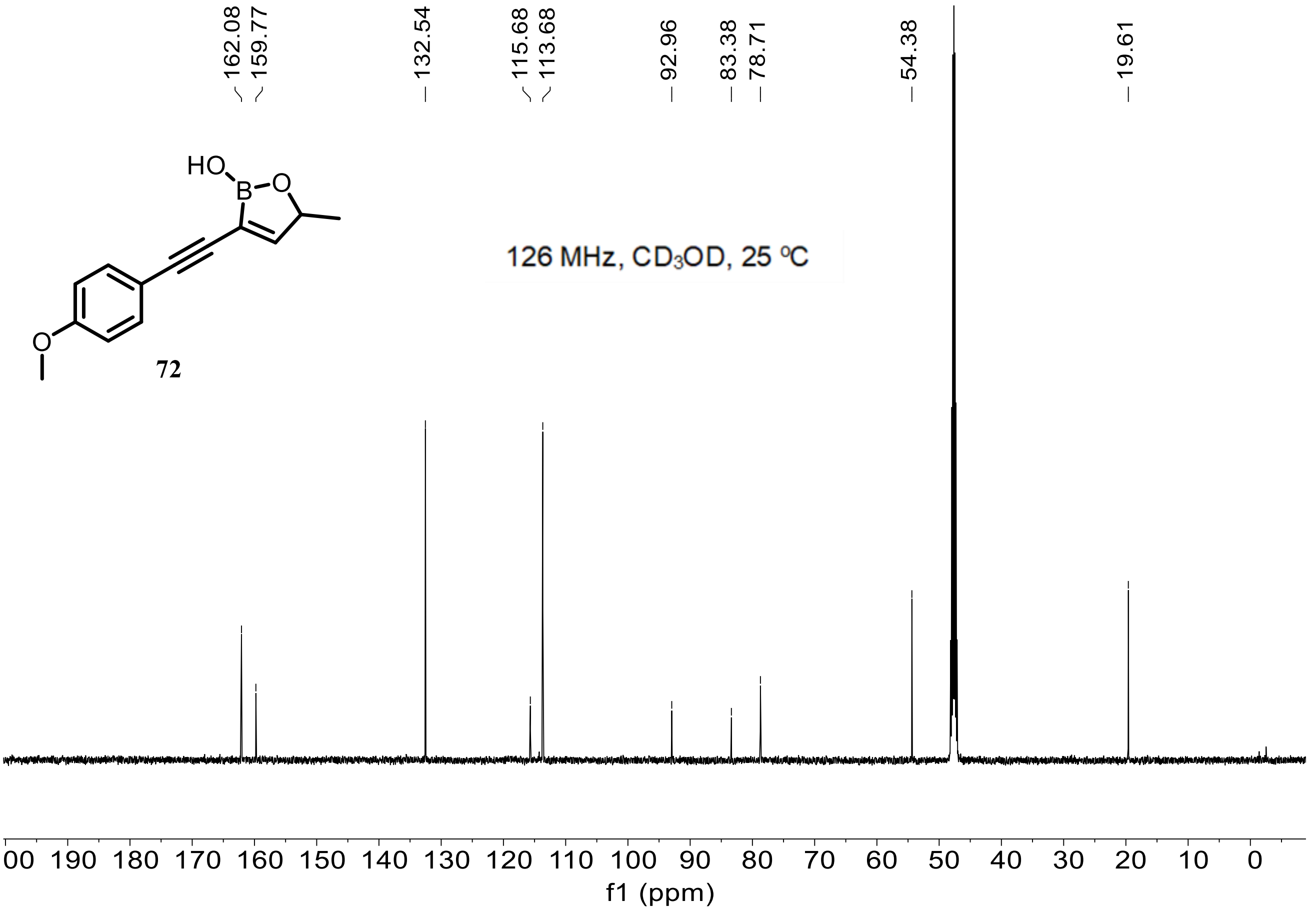

162.08
159.77
132.54
115.68
113.68
92.96
83.38
78.71
54.38
19.61
HO
B
O
72
126 MHz, CD3OD, 25 ºC
00 190 180 170 160 150 140 130 120 110 100 90 80 70 60 50 40 30 20 10 0
f1 (ppm)

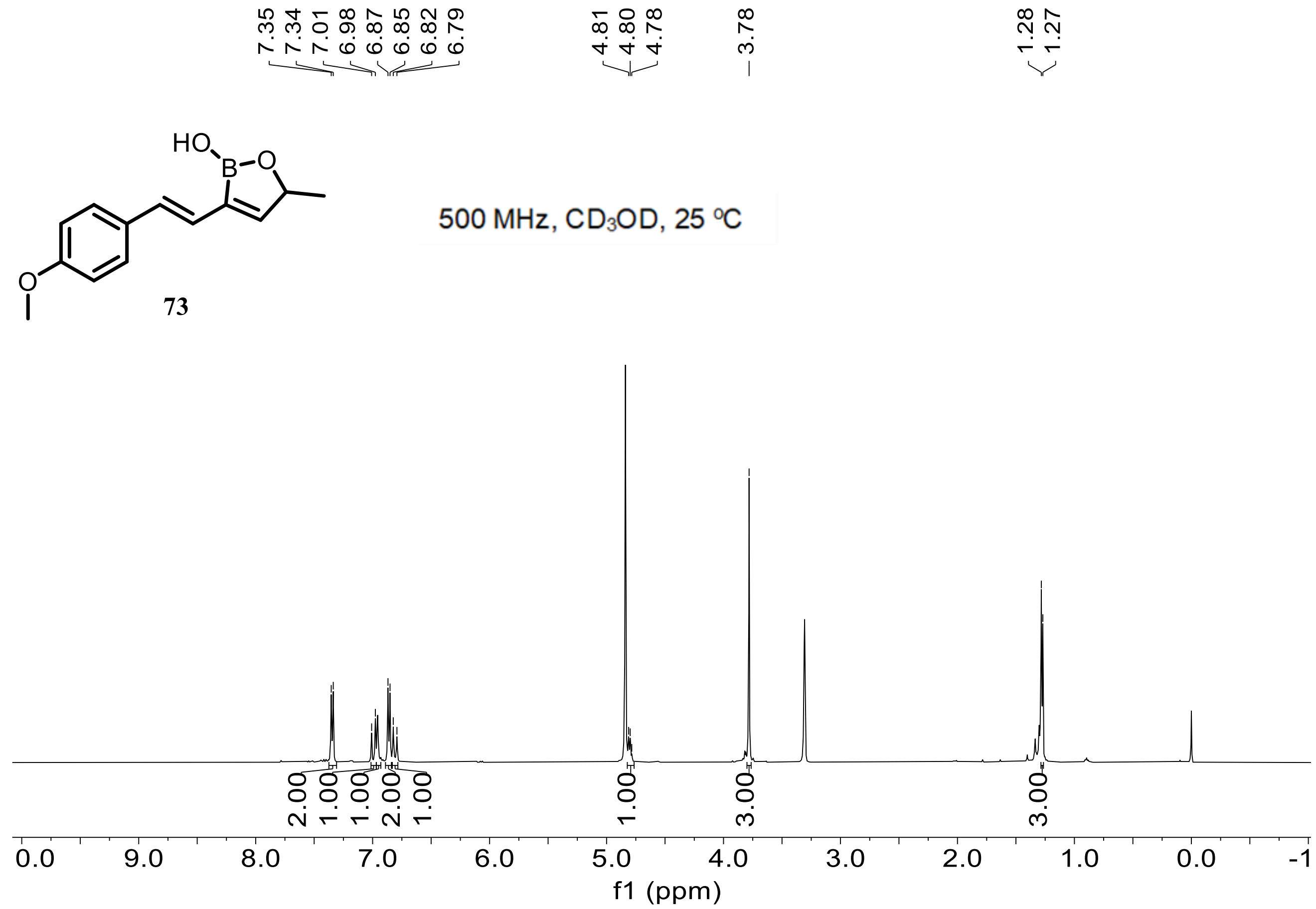
7.35 7.34 7.01 6.98 6.87 6.85 6.82 6.79
4.81 4.80 4.78
3.78
1.28 1.27
HO B O
73
500 MHz, CD3OD, 25 °C
2.00 1.00 1.00 2.00 1.00
1.00
3.00
3.00
0.0 9.0 8.0 7.0 6.0 5.0 4.0 3.0 2.0 1.0 0.0 -1
f1 (ppm)

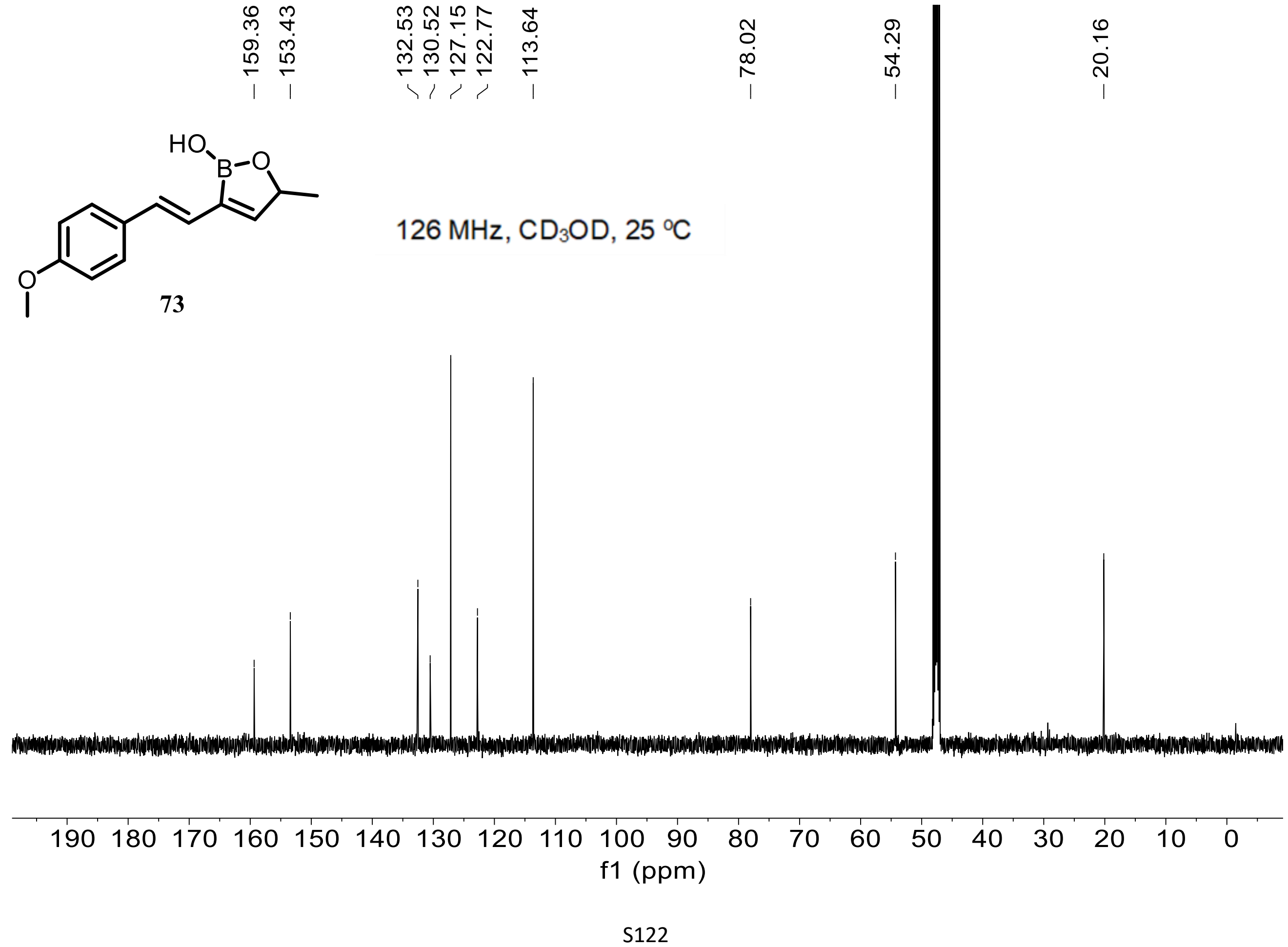
159.36 153.43
132.53 130.52 127.15 122.77
113.64
78.02
54.29
20.16
HO B O
73
126 MHz, CD3OD, 25 °C
190 180 170 160 150 140 130 120 110 100 90 80 70 60 50 40 30 20 10 0
f1 (ppm)

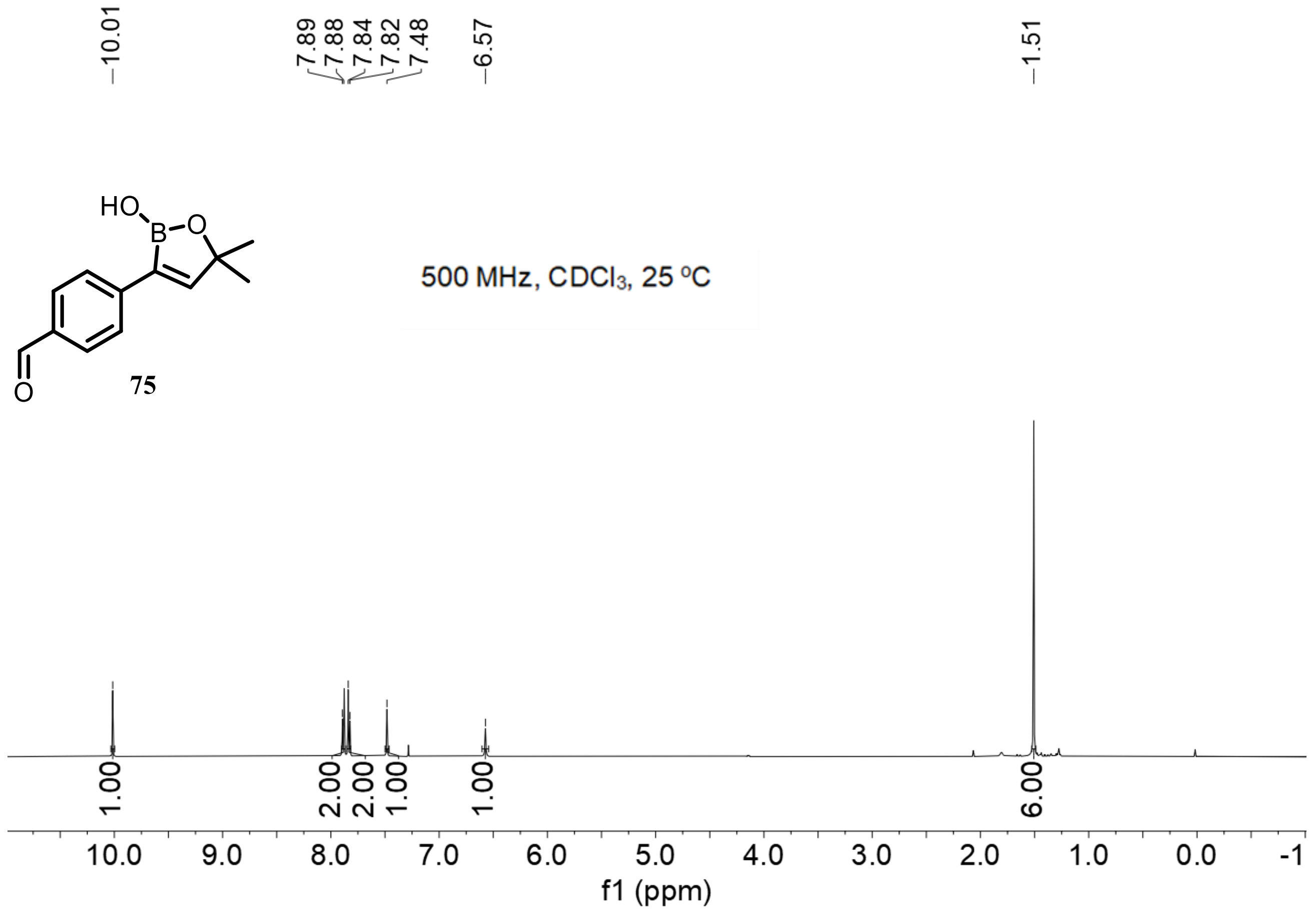
10.01
7.89
7.88
7.84
7.82
7.48
6.57
1.51
HO
B
O
75
500 MHz, CDCl3, 25 °C
1.00
2.00
2.00
1.00
1.00
6.00
10.0 9.0 8.0 7.0 6.0 5.0 4.0 3.0 2.0 1.0 0.0 -1
f1 (ppm)

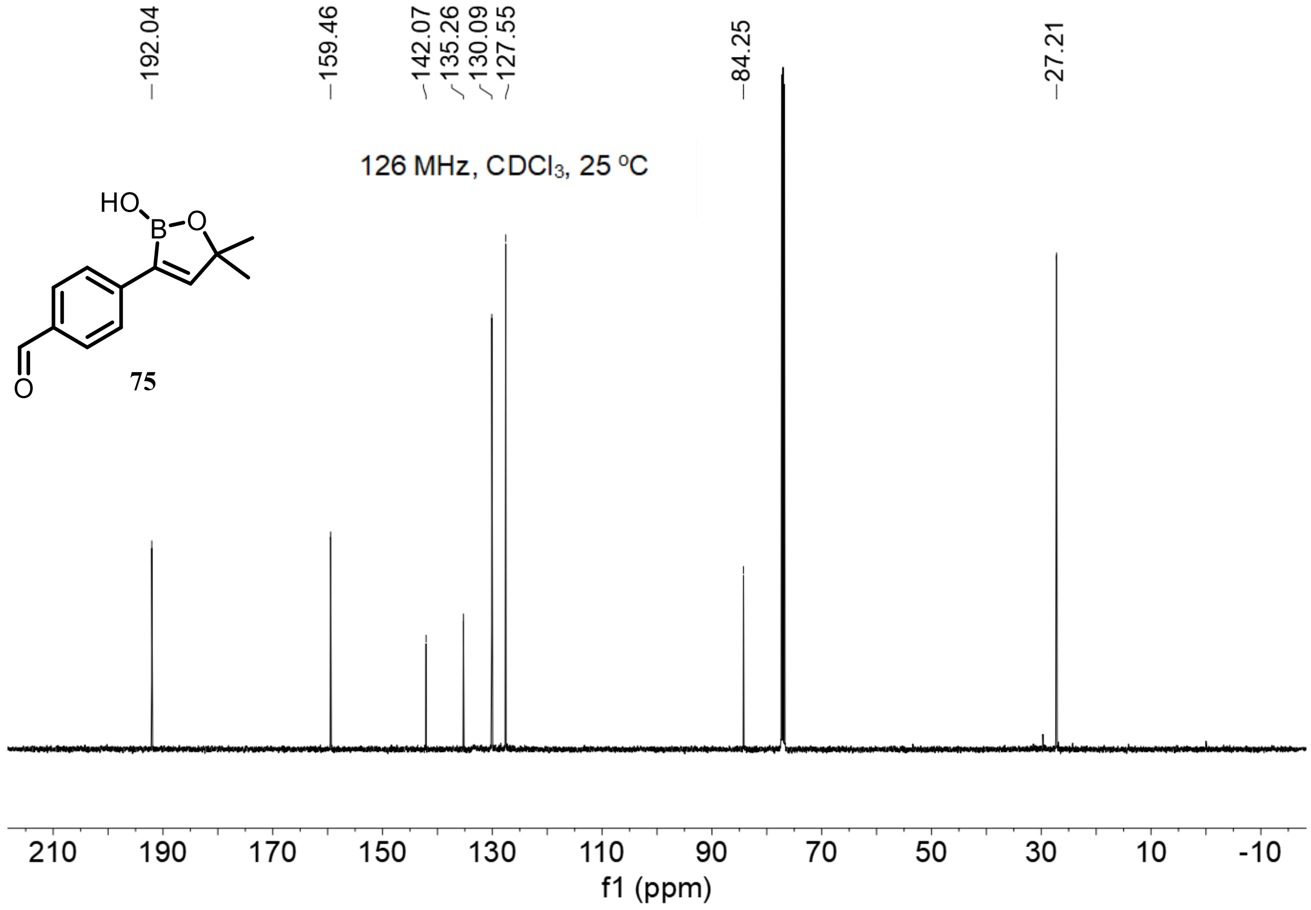
192.04
159.46
142.07
135.26
130.09
127.55
84.25
27.21
126 MHz, CDCl3, 25 °C
HO
B
O
75
210 190 170 150 130 110 90 70 50 30 10 -10
f1 (ppm)

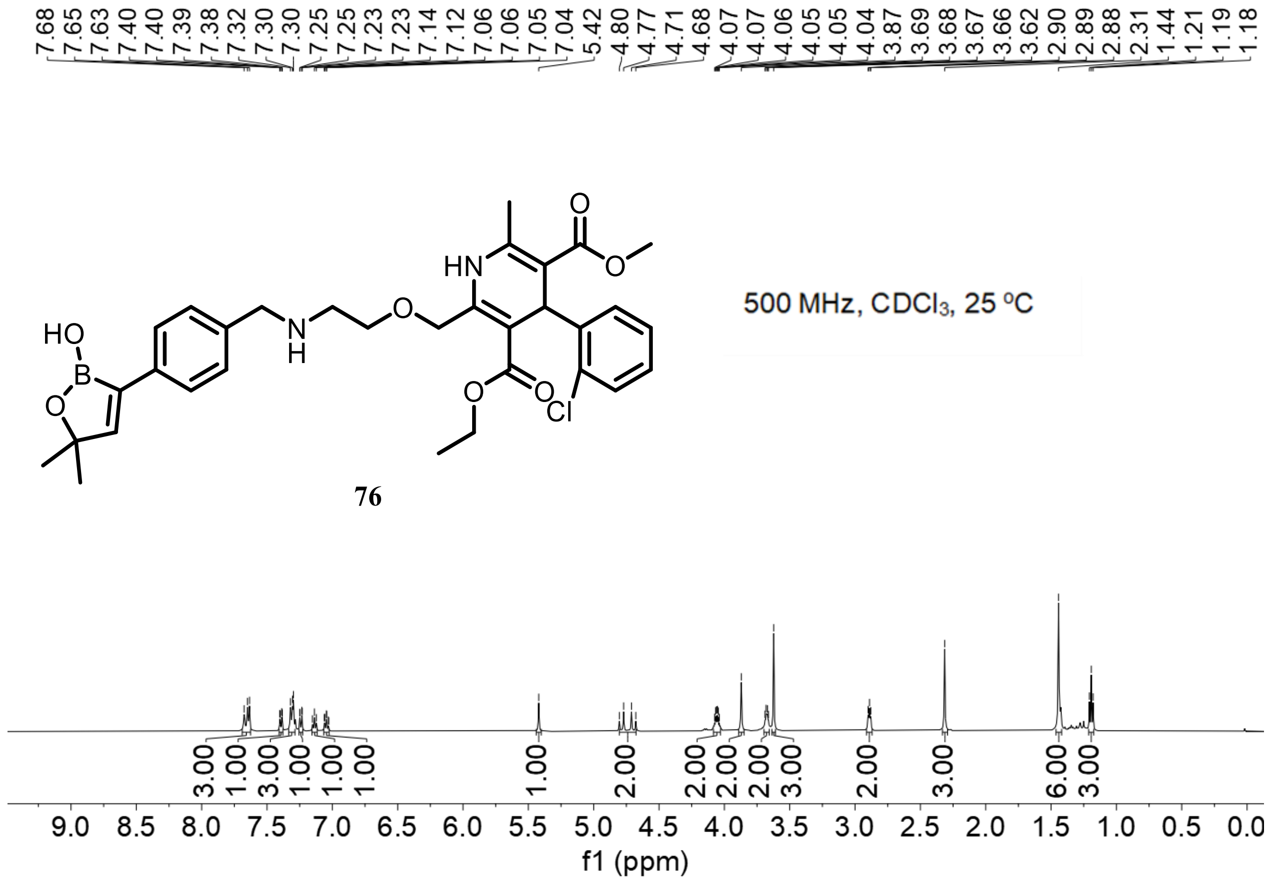
7.68 7.65 7.63 7.40 7.40 7.39 7.38 7.32 7.30 7.30 7.25 7.25 7.23 7.23 7.14 7.12 7.06 7.06 7.05 7.04 5.42 4.80 4.77 4.71 4.68 4.07 4.07 4.06 4.05 4.05 4.04 3.87 3.69 3.68 3.67 3.66 3.62 2.90 2.89 2.88 2.31 1.44 1.21 1.19 1.18
HO
B
O
HN
O
O
N
H
O
O
Cl
500 MHz, CDCl3, 25 °C
3.00 1.00 3.00 1.00 1.00 1.00 1.00 2.00 2.00 2.00 2.00 3.00 2.00 3.00 6.00 3.00
9.0 8.5 8.0 7.5 7.0 6.5 6.0 5.5 5.0 4.5 4.0 3.5 3.0 2.5 2.0 1.5 1.0 0.5 0.0
f1 (ppm)

76

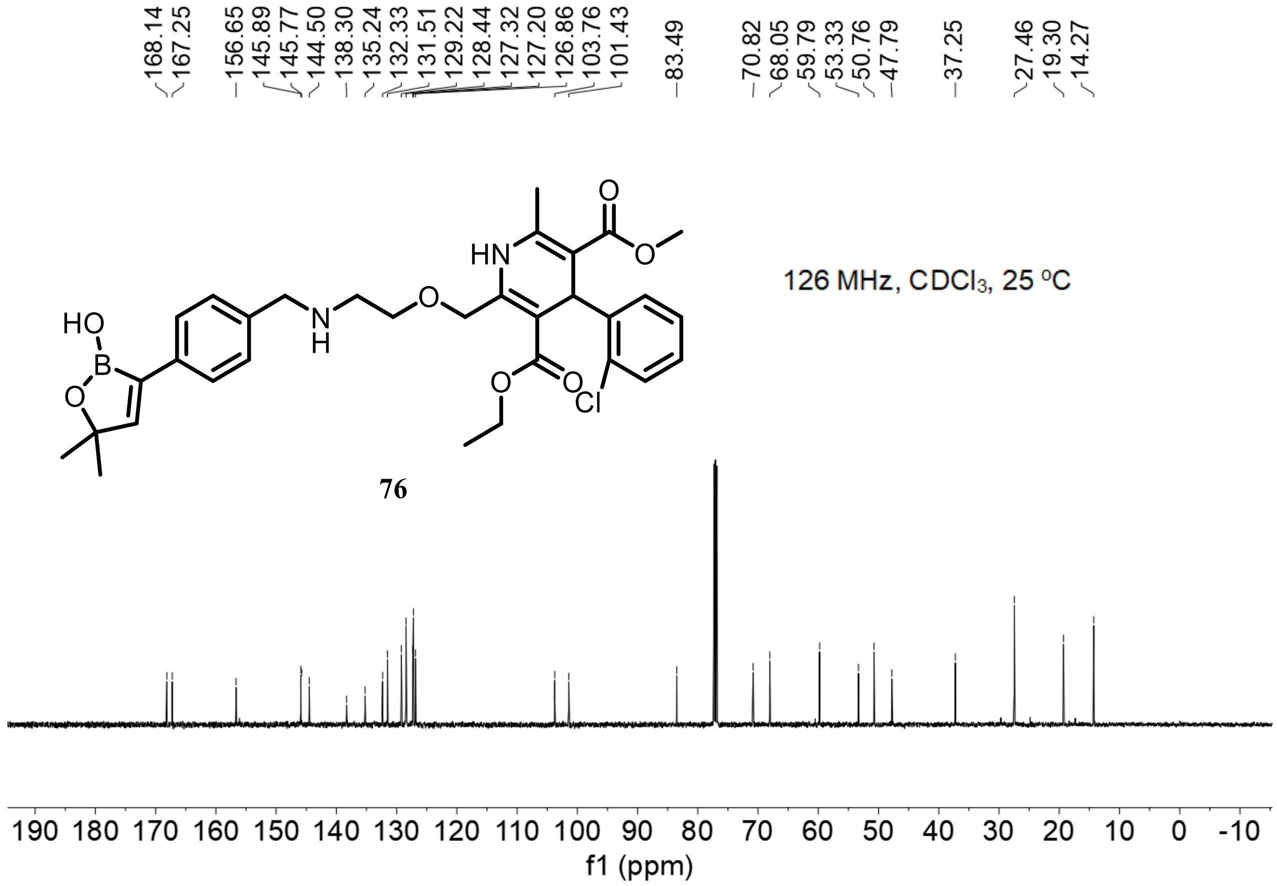
168.14 167.25 156.65 145.89 145.77 144.50 138.30 135.24 132.33 131.51 129.22 128.44 127.32 127.20 126.86 103.76 101.43 83.49 70.82 68.05 59.79 53.33 50.76 47.79 37.25 27.46 19.30 14.27
HO
B
O
HN
O
O
N
H
O
O
Cl
126 MHz, CDCl3, 25 °C
190 180 170 160 150 140 130 120 110 100 90 80 70 60 50 40 30 20 10 0 -10
f1 (ppm)

76

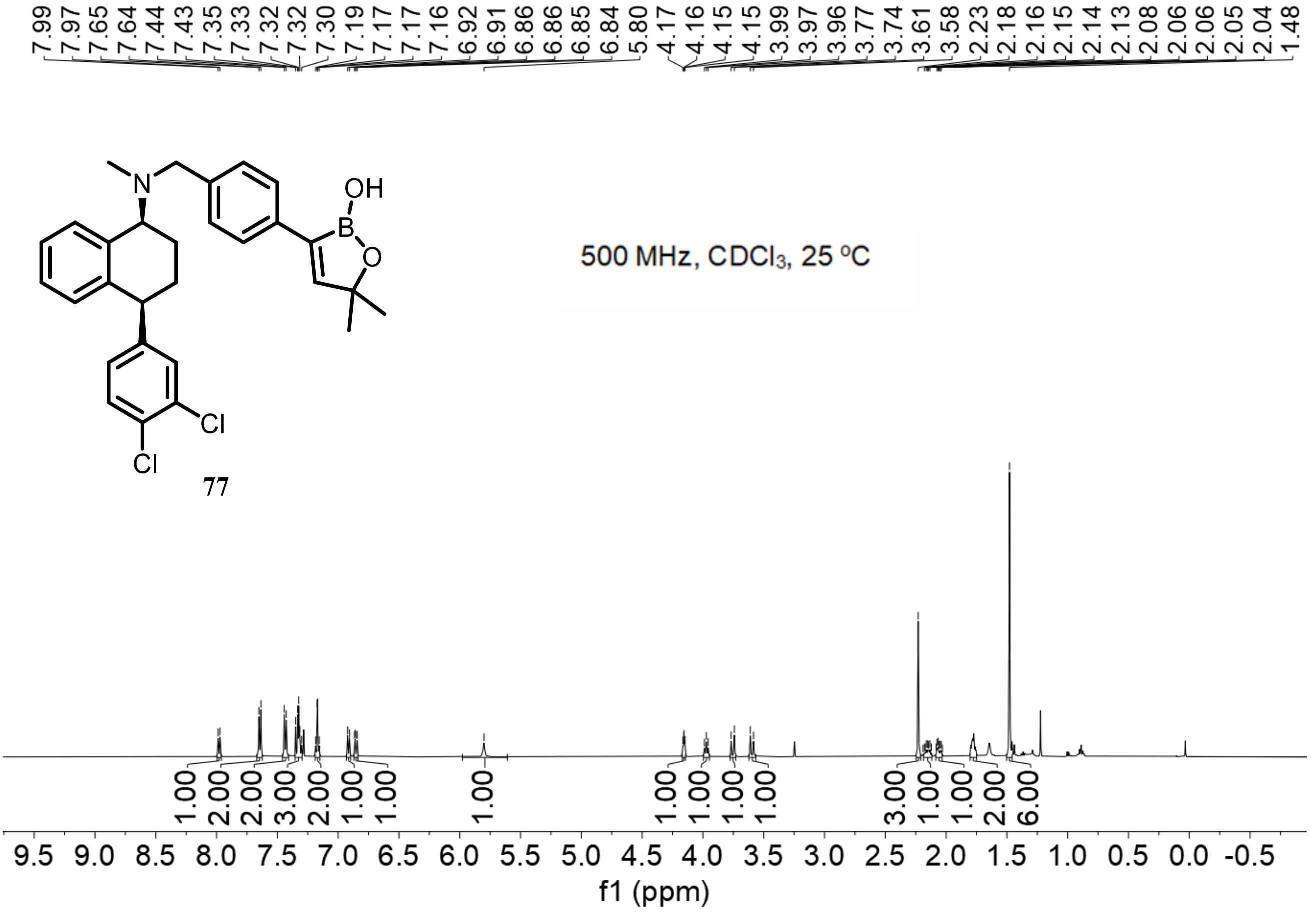
7.99 7.97 7.65 7.64 7.44 7.43 7.35 7.33 7.32 7.32 7.30 7.19 7.17 7.17 7.16 6.92 6.91 6.86 6.86 6.85 6.84 5.80 4.17 4.16 4.15 4.15 3.99 3.97 3.96 3.77 3.74 3.61 3.58 2.23 2.18 2.16 2.15 2.14 2.13 2.08 2.06 2.06 2.05 2.04 1.48
OH
B
O
N
Cl
Cl
77
500 MHz, CDCl3, 25 °C
1.00 2.00 2.00 3.00 2.00 1.00 1.00 1.00 1.00 1.00 1.00 1.00 3.00 1.00 1.00 2.00 6.00
9.5 9.0 8.5 8.0 7.5 7.0 6.5 6.0 5.5 5.0 4.5 4.0 3.5 3.0 2.5 2.0 1.5 1.0 0.5 0.0 -0.5
f1 (ppm)

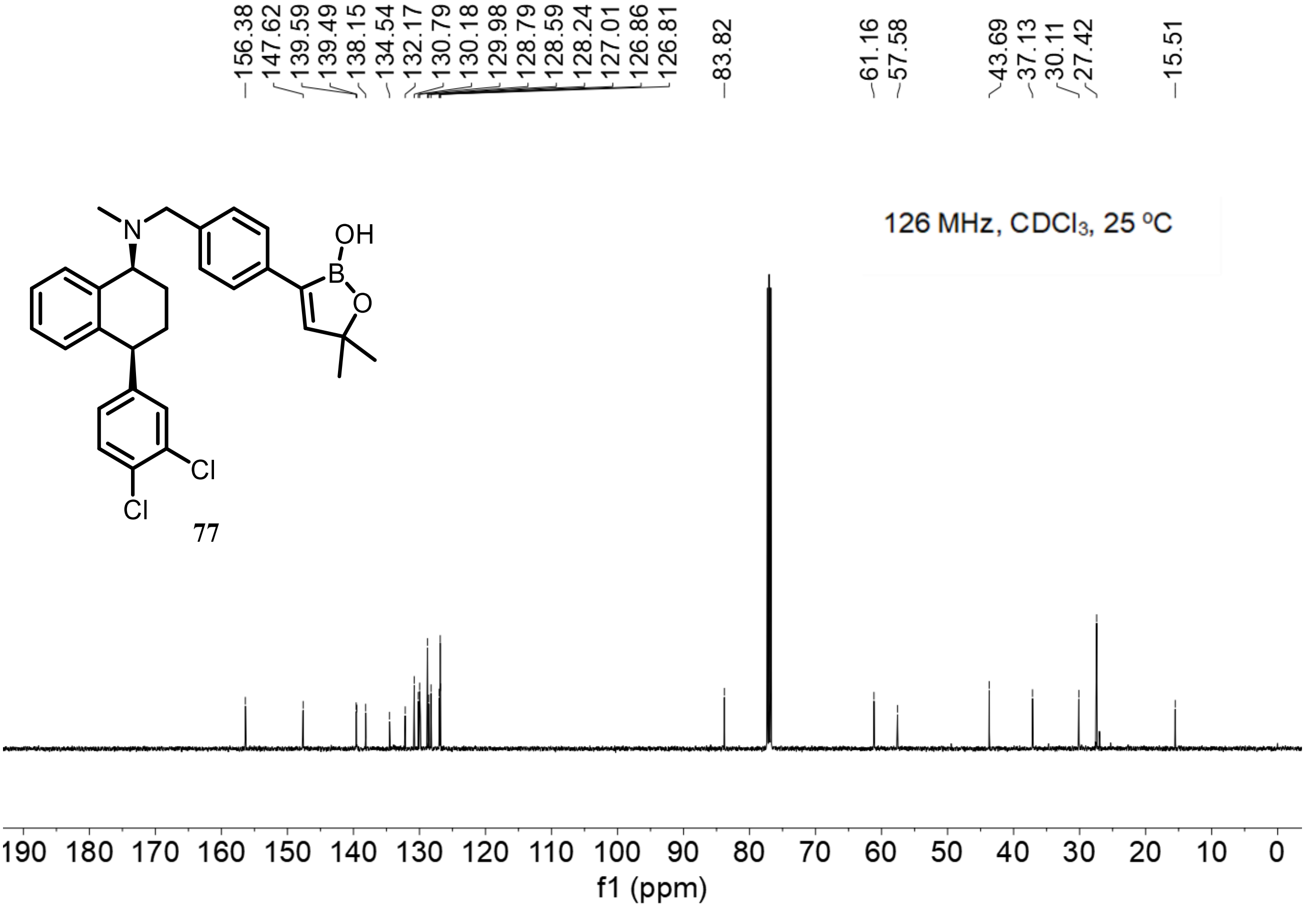
156.38 147.62 139.59 139.49 138.15 134.54 132.17 130.79 130.18 129.98 128.79 128.59 128.24 127.01 126.86 126.81 83.82 61.16 57.58 43.69 37.13 30.11 27.42 15.51
OH
B
O
N
Cl
Cl
77
126 MHz, CDCl3, 25 °C
190 180 170 160 150 140 130 120 110 100 90 80 70 60 50 40 30 20 10 0
f1 (ppm)

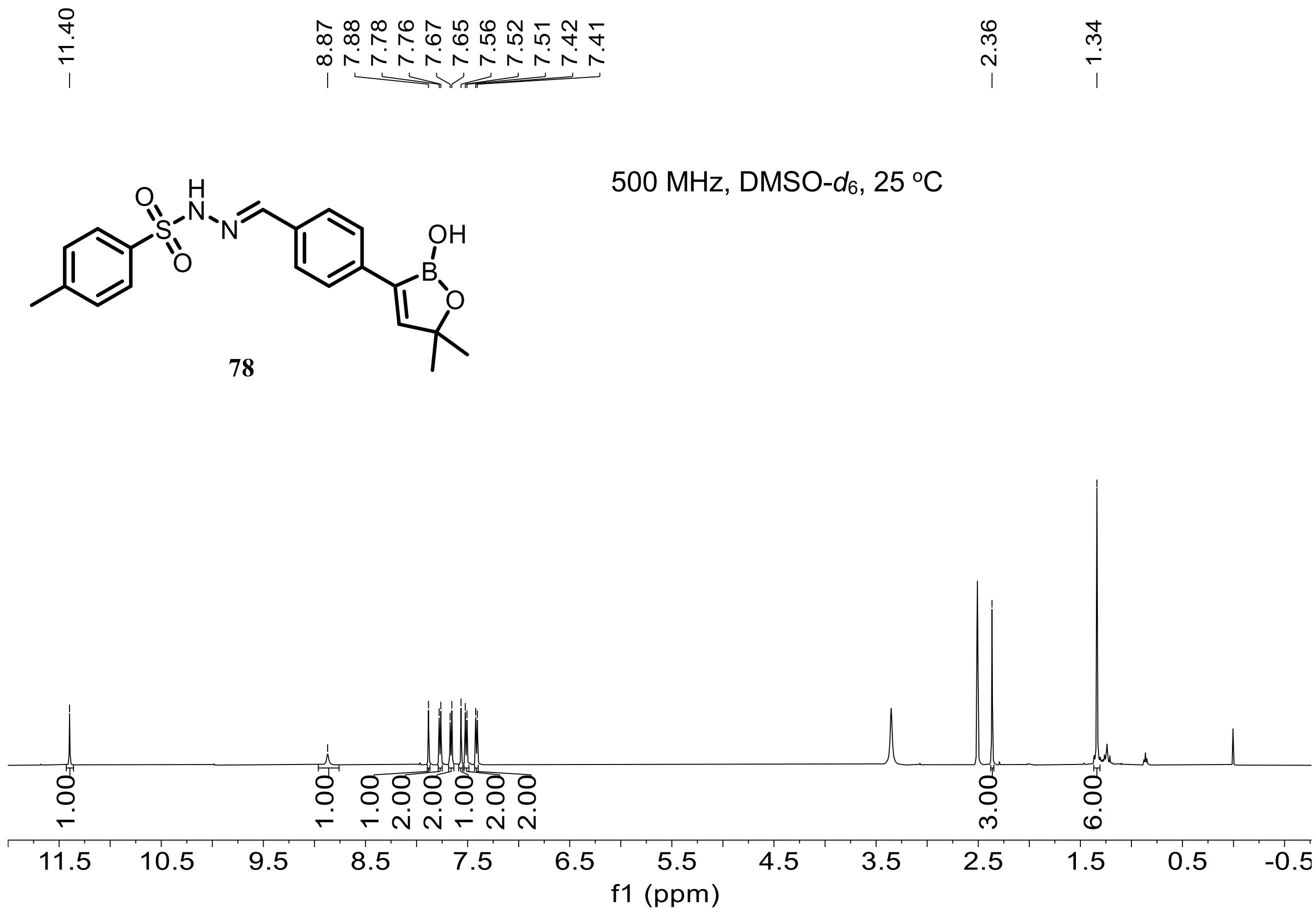

11.40
8.87 7.88 7.78 7.76 7.67 7.65 7.56 7.52 7.51 7.42 7.41
2.36
1.34
500 MHz, DMSO-d_6, 25 °C
78
1.00 1.00 1.00 2.00 2.00 1.00 2.00 2.00 3.00 6.00
11.5 10.5 9.5 8.5 7.5 6.5 5.5 4.5 3.5 2.5 1.5 0.5 -0.5
f1 (ppm)

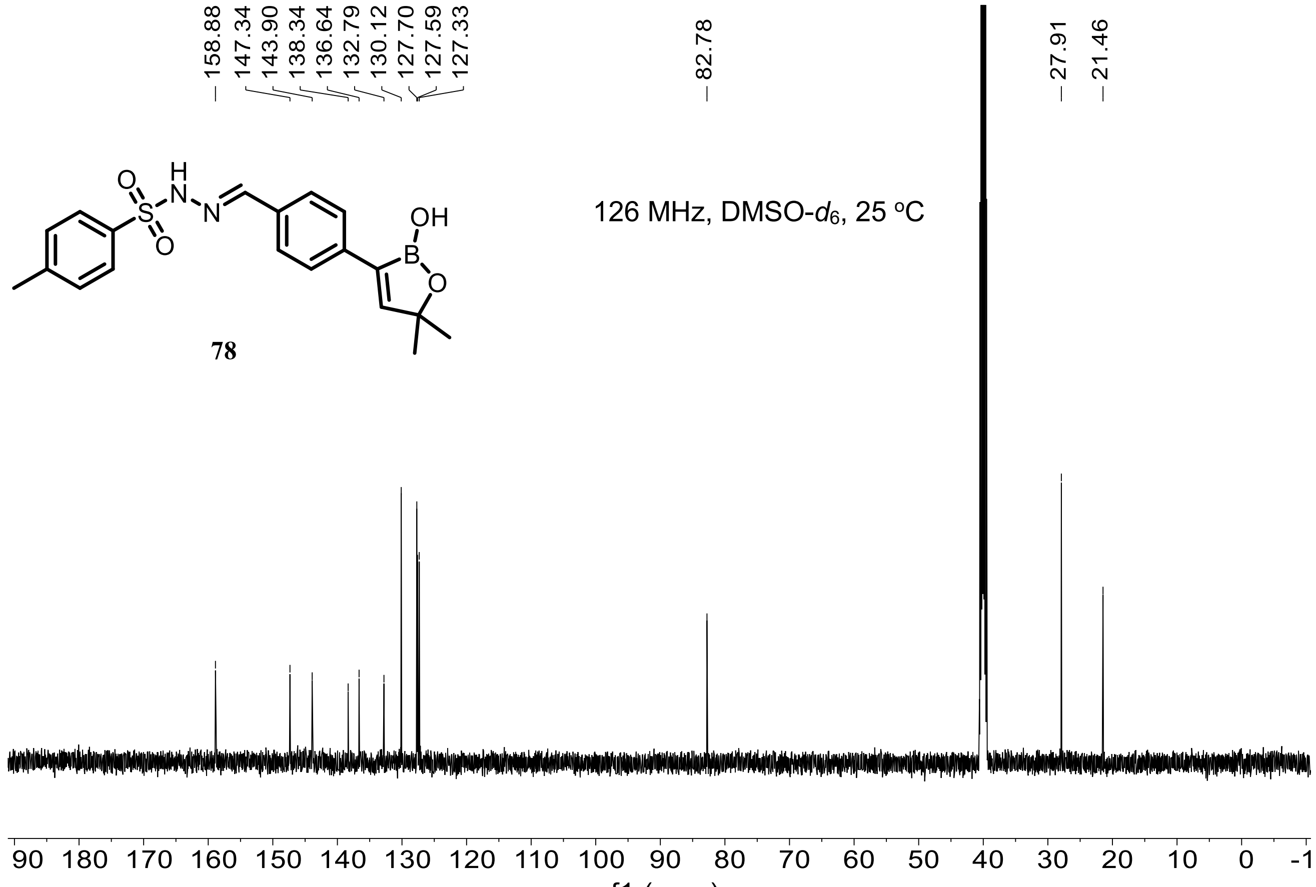

158.88 147.34 143.90 138.34 136.64 132.79 130.12 127.70 127.59 127.33
82.78
27.91 21.46
126 MHz, DMSO-d_6, 25 °C
78
90 180 170 160 150 140 130 120 110 100 90 80 70 60 50 40 30 20 10 0 -1
f1 (ppm)

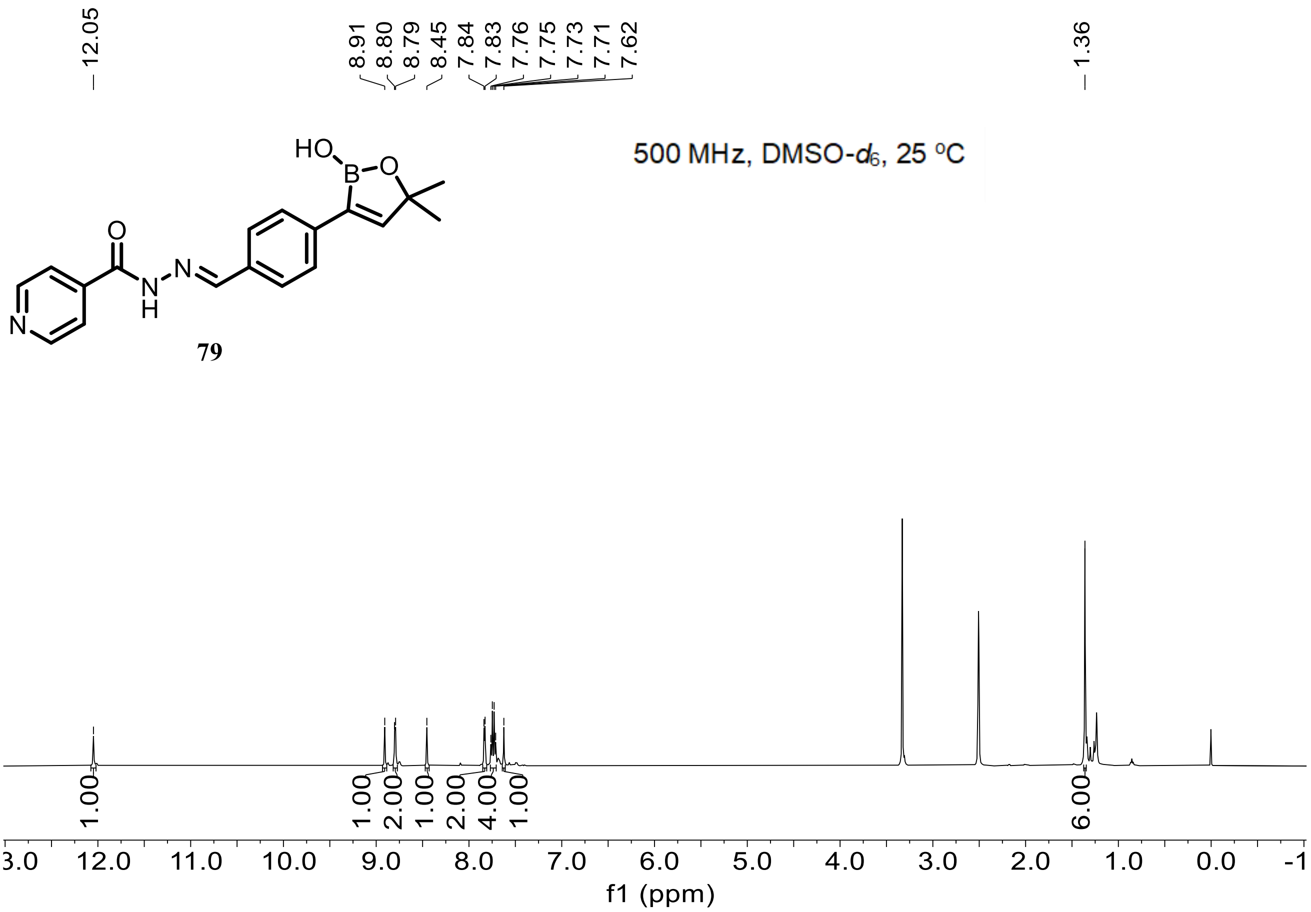
12.05
8.91
8.80
8.79
8.45
7.84
7.83
7.76
7.75
7.73
7.71
7.62
1.36
500 MHz, DMSO-d6, 25 °C
79
1.00
1.00
2.00
1.00
2.00
4.00
1.00
6.00
3.0 12.0 11.0 10.0 9.0 8.0 7.0 6.0 5.0 4.0 3.0 2.0 1.0 0.0 -1
f1 (ppm)

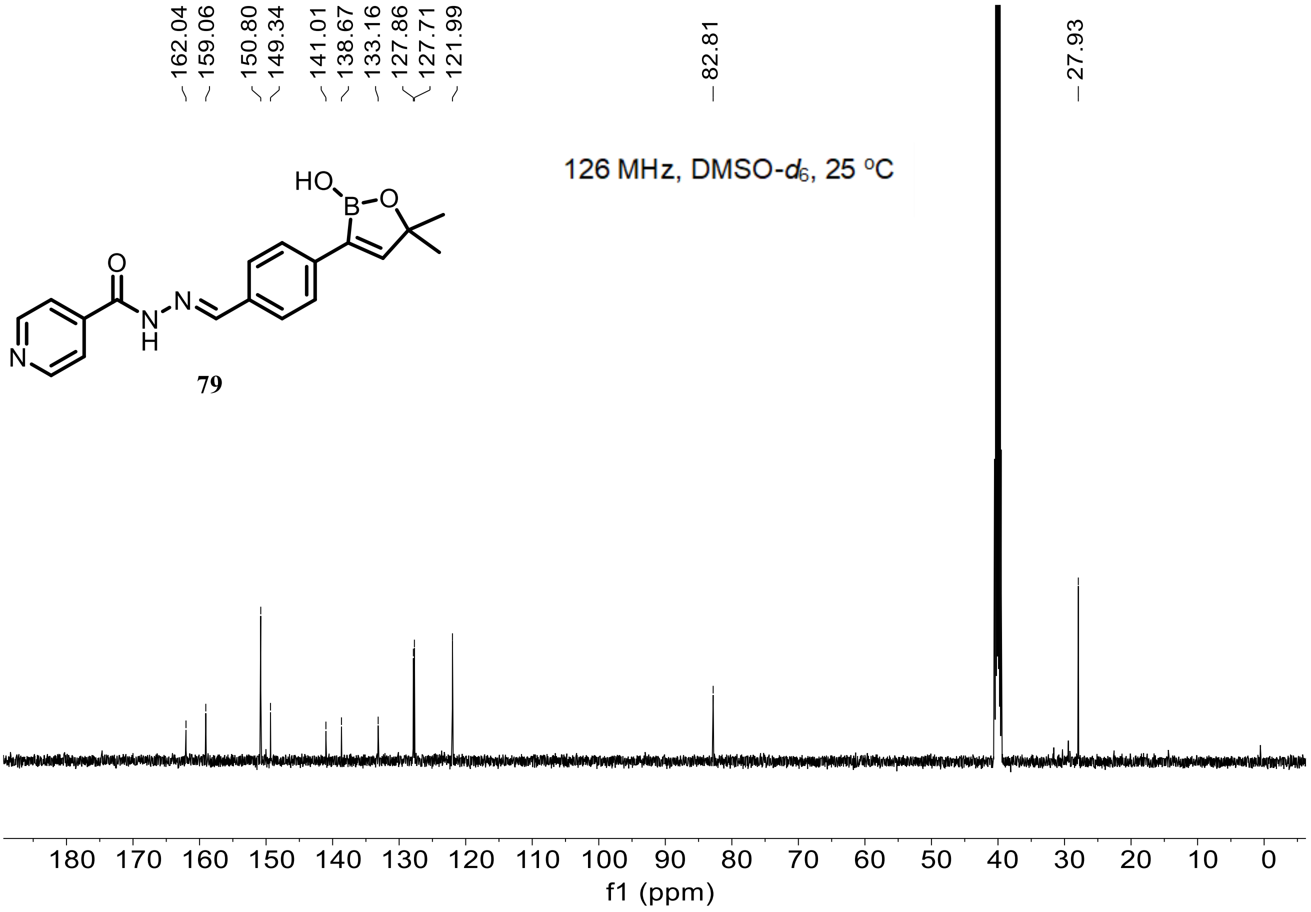
162.04
159.06
150.80
149.34
141.01
138.67
133.16
127.86
127.71
121.99
82.81
27.93
126 MHz, DMSO-d6, 25 °C
79
180 170 160 150 140 130 120 110 100 90 80 70 60 50 40 30 20 10 0
f1 (ppm)

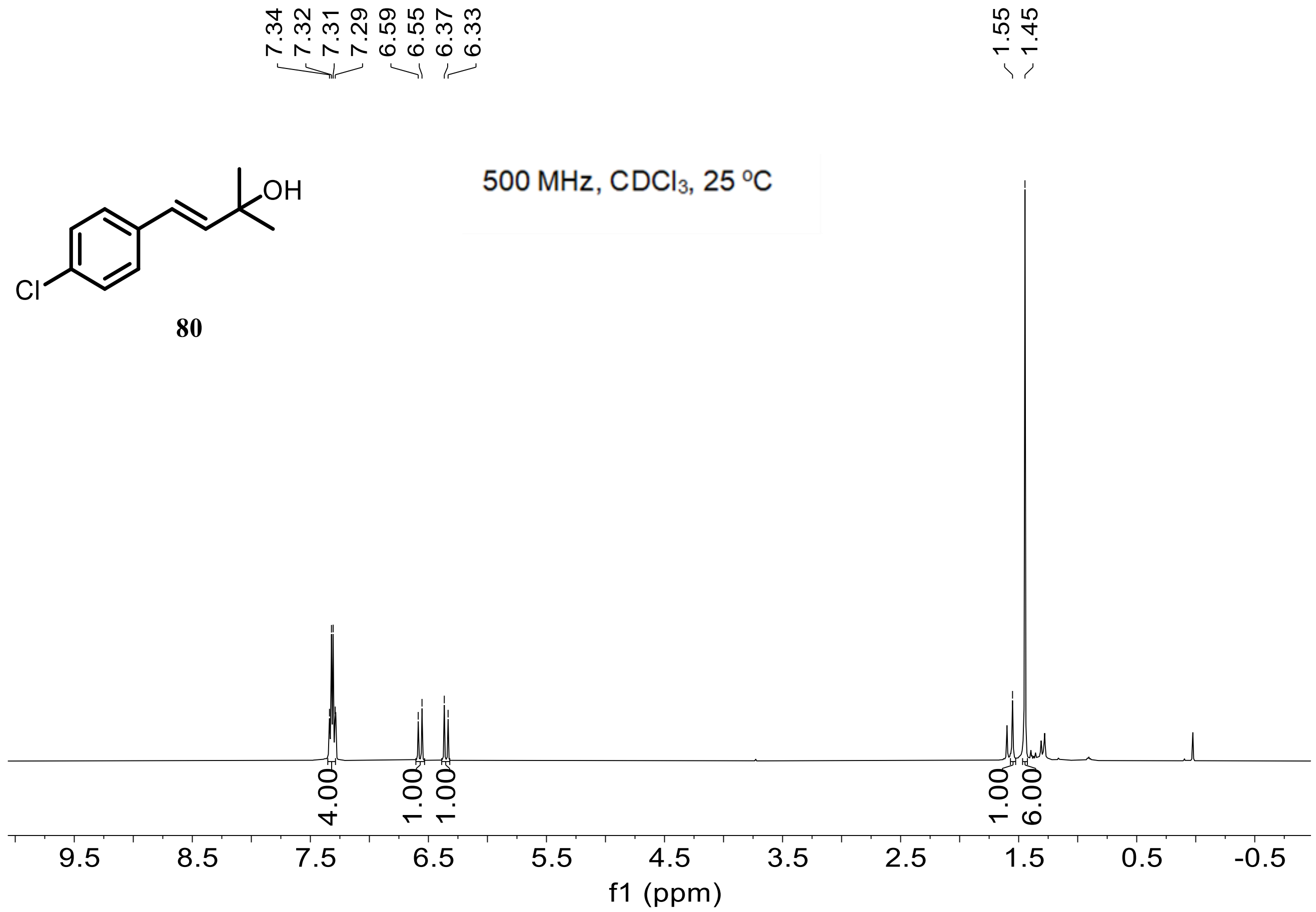
7.34
7.32
7.31
7.29
6.59
6.55
6.37
6.33
1.55
1.45
OH
Cl
80
500 MHz, CDCl3, 25 °C
4.00
1.00
1.00
1.00
6.00
9.5 8.5 7.5 6.5 5.5 4.5 3.5 2.5 1.5 0.5 -0.5
f1 (ppm)

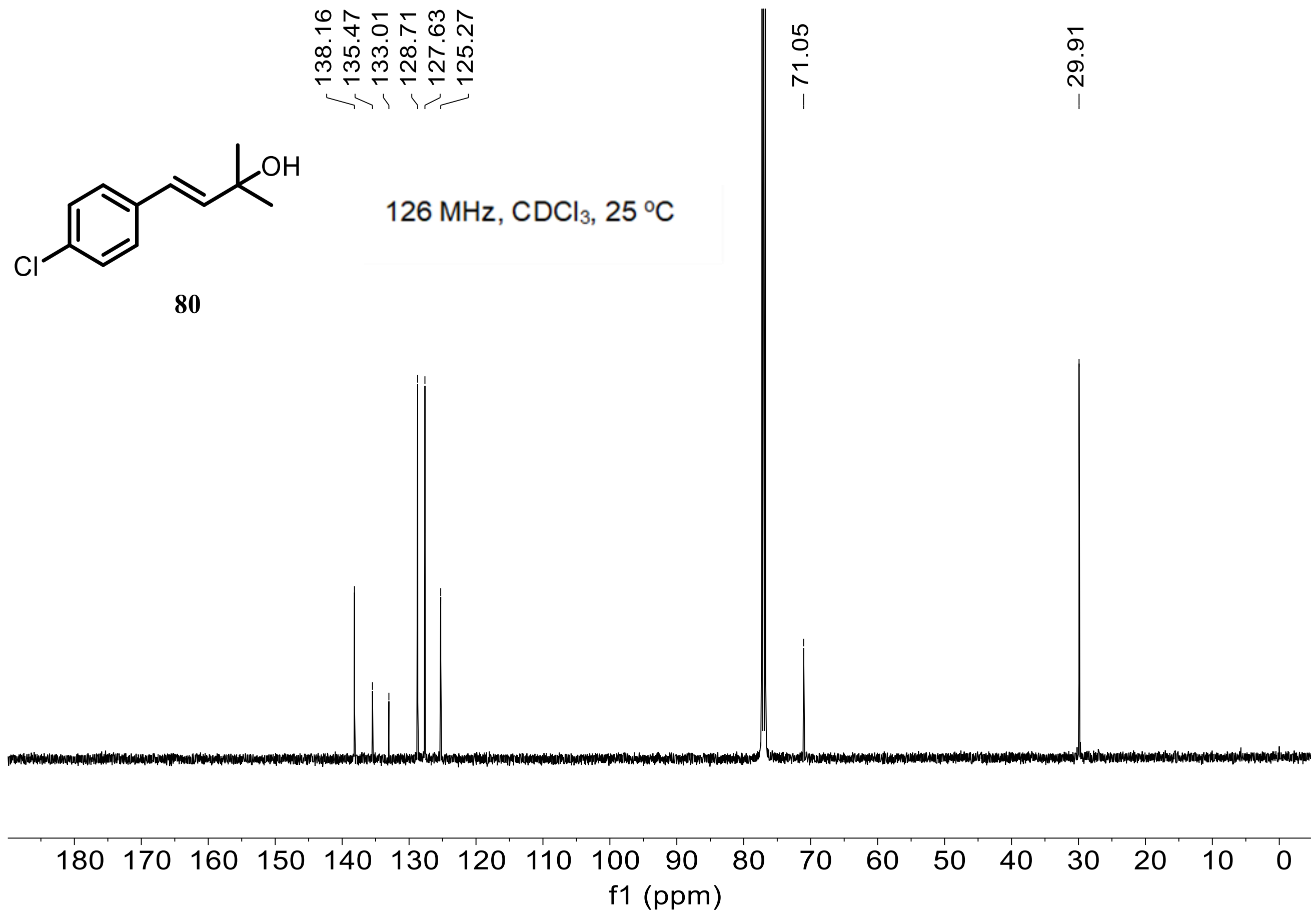
138.16
135.47
133.01
128.71
127.63
125.27
71.05
29.91
OH
Cl
80
126 MHz, CDCl3, 25 °C
180 170 160 150 140 130 120 110 100 90 80 70 60 50 40 30 20 10 0
f1 (ppm)

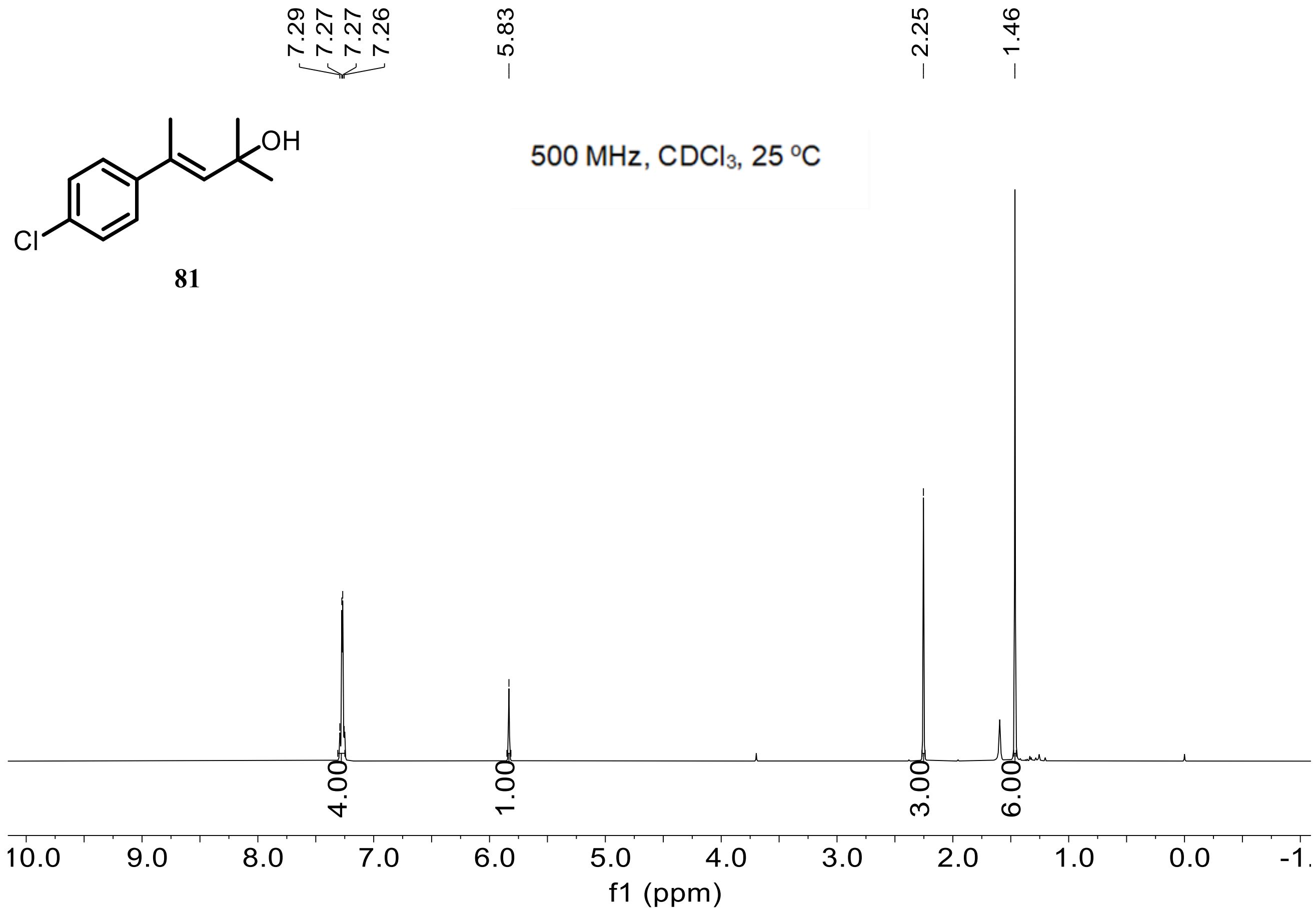
7.29
7.27
7.27
7.26
5.83
2.25
1.46
OH
Cl
81
500 MHz, CDCl$_3$, 25 °C
4.00
1.00
3.00
6.00
10.0 9.0 8.0 7.0 6.0 5.0 4.0 3.0 2.0 1.0 0.0 -1.
f1 (ppm)

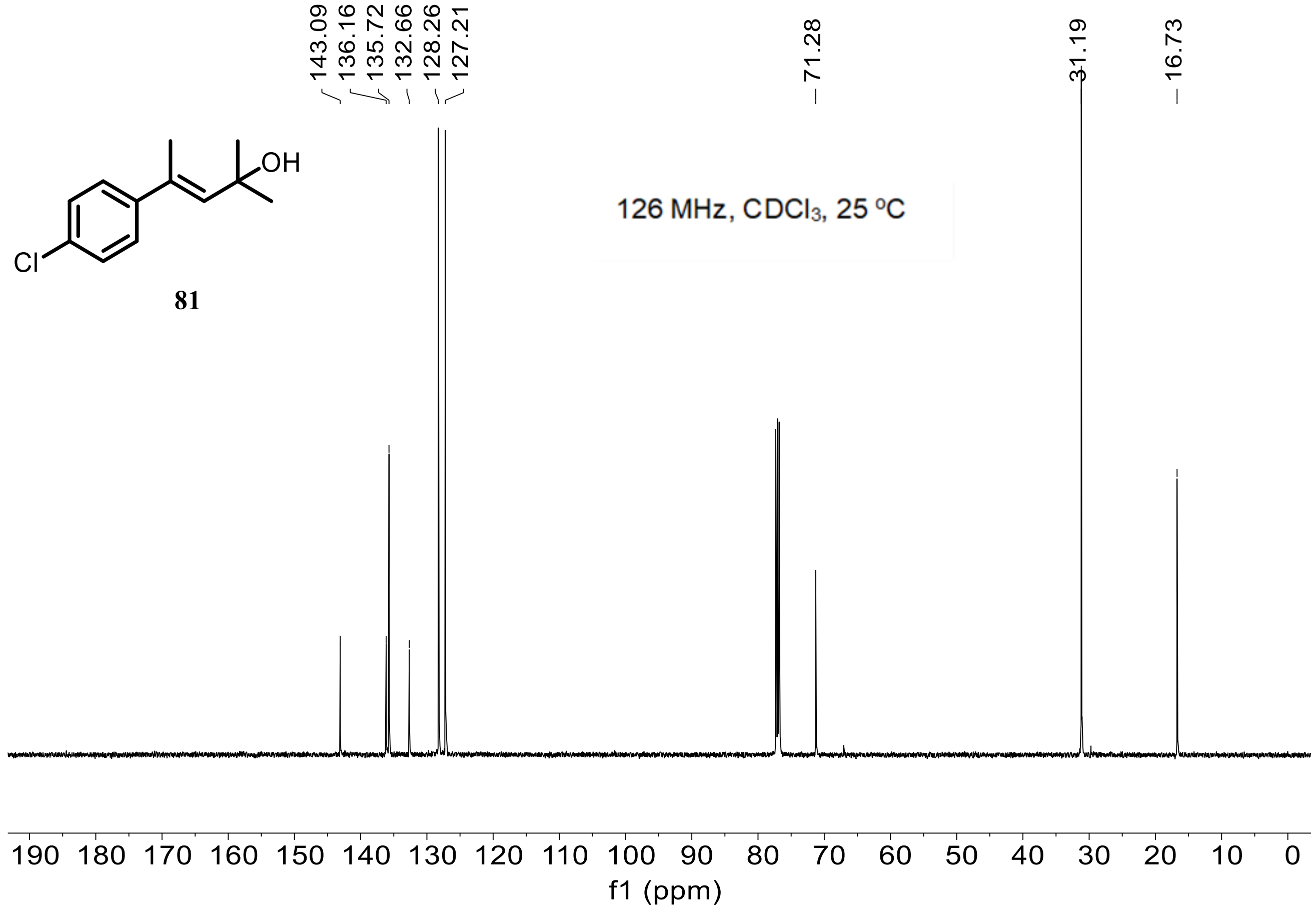
143.09
136.16
135.72
132.66
128.26
127.21
71.28
31.19
16.73
OH
Cl
81
126 MHz, CDCl$_3$, 25 °C
190 180 170 160 150 140 130 120 110 100 90 80 70 60 50 40 30 20 10 0
f1 (ppm)

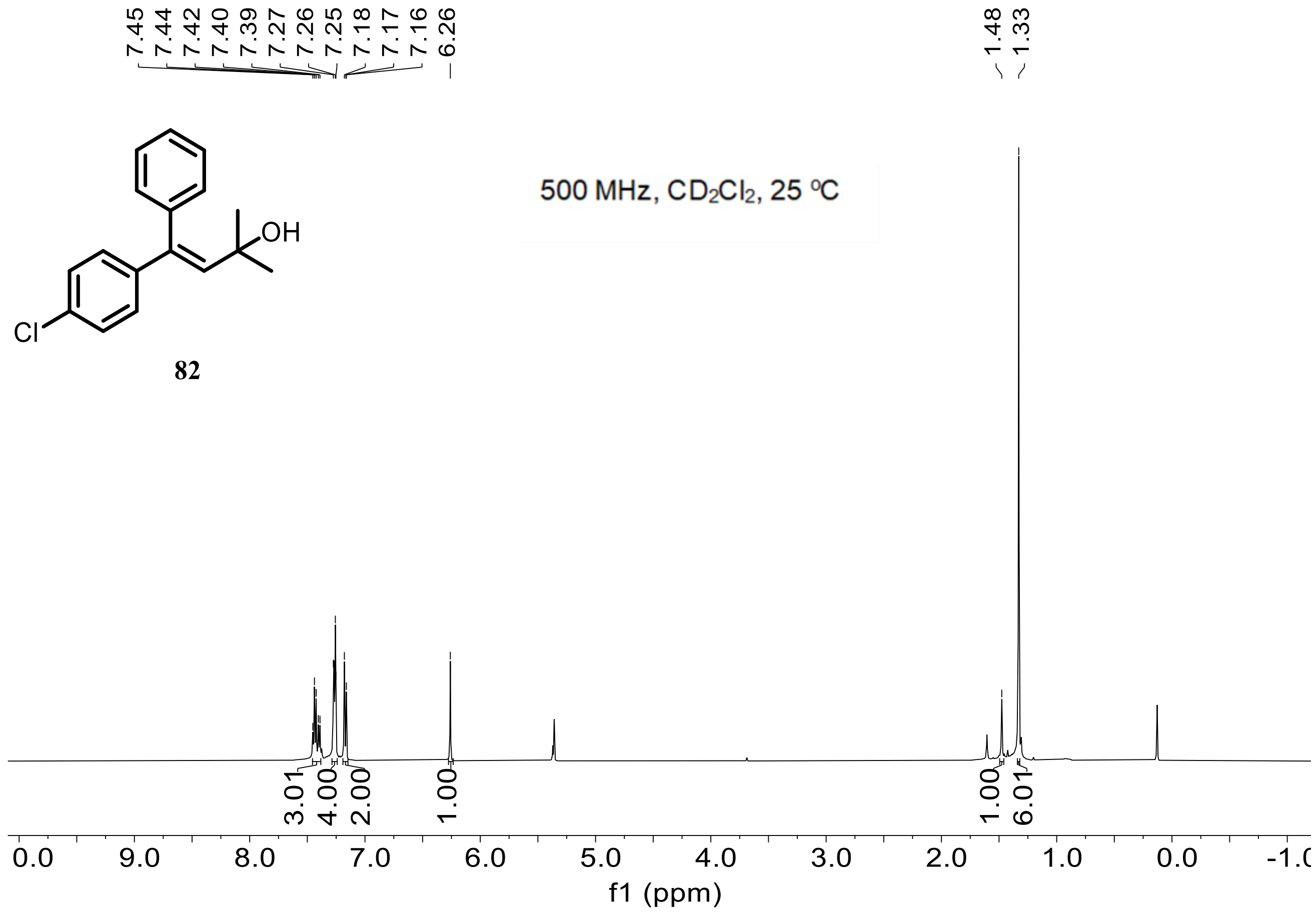
7.45 7.44 7.42 7.40 7.39 7.27 7.26 7.25 7.18 7.17 7.16 6.26
1.48 1.33
500 MHz, CD_2Cl_2, 25 °C
OH
Cl
82
3.01 4.00 2.00 1.00 1.00 6.01
0.0 9.0 8.0 7.0 6.0 5.0 4.0 3.0 2.0 1.0 0.0 -1.0
f1 (ppm)

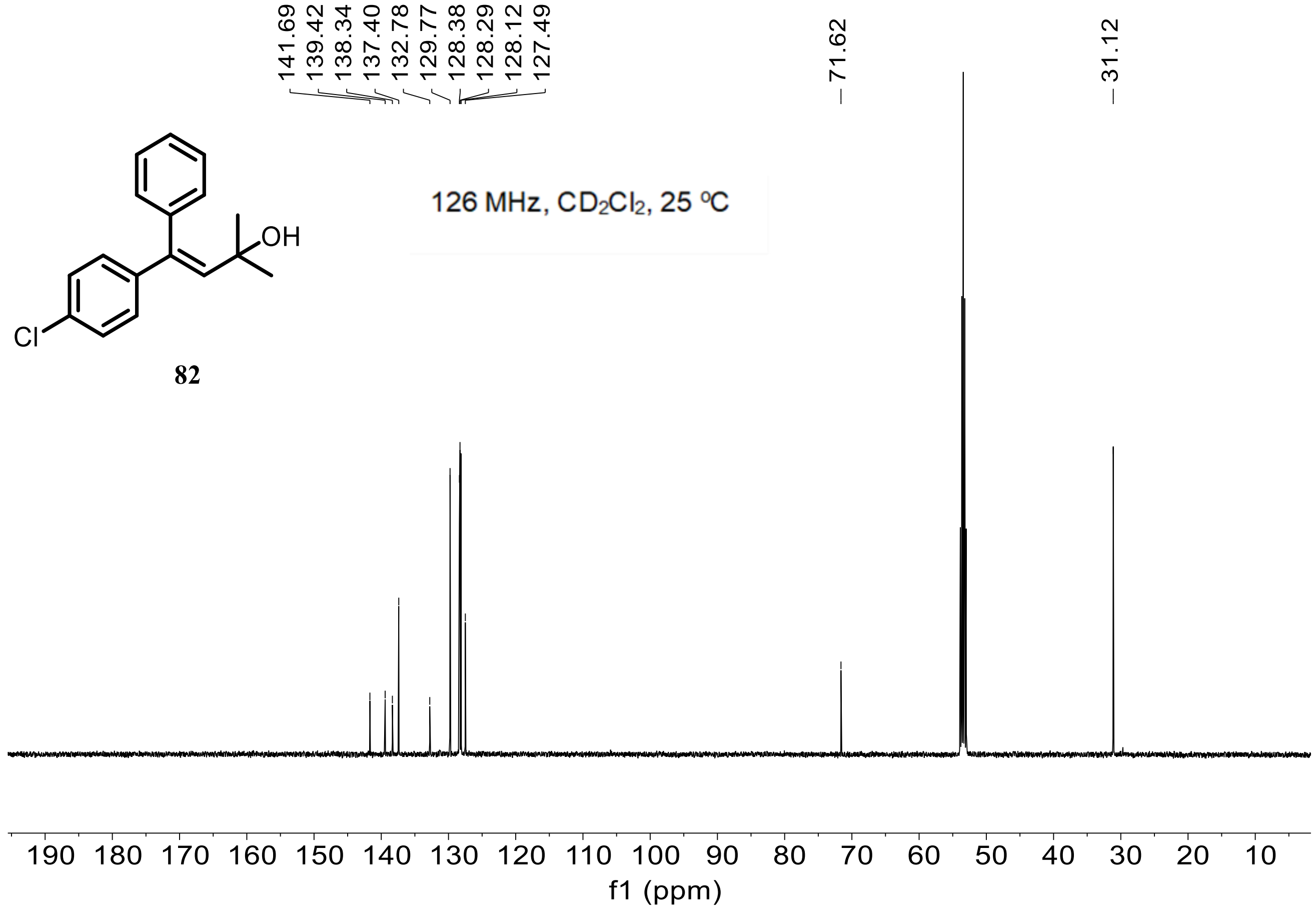
141.69 139.42 138.34 137.40 132.78 129.77 128.38 128.29 128.12 127.49
71.62
31.12
126 MHz, CD_2Cl_2, 25 °C
OH
Cl
82
190 180 170 160 150 140 130 120 110 100 90 80 70 60 50 40 30 20 10
f1 (ppm)

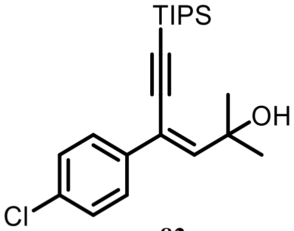
TIPS
OH
Cl

83

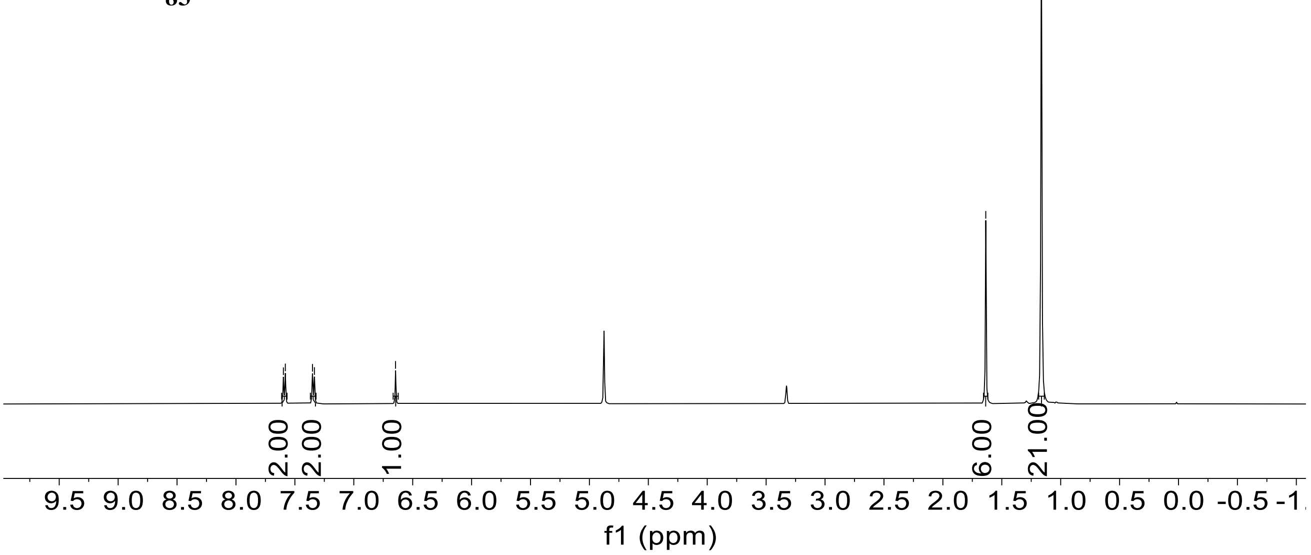
7.60
7.58
7.35
7.33
6.65
1.64
1.16
500 MHz, CD3OD, 25 °C
2.00
2.00
1.00
6.00
21.00
9.5 9.0 8.5 8.0 7.5 7.0 6.5 6.0 5.5 5.0 4.5 4.0 3.5 3.0 2.5 2.0 1.5 1.0 0.5 0.0 -0.5
f1 (ppm)

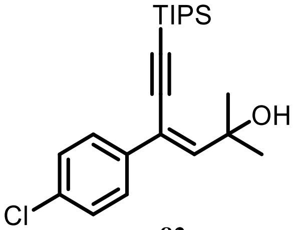
TIPS
OH
Cl

83

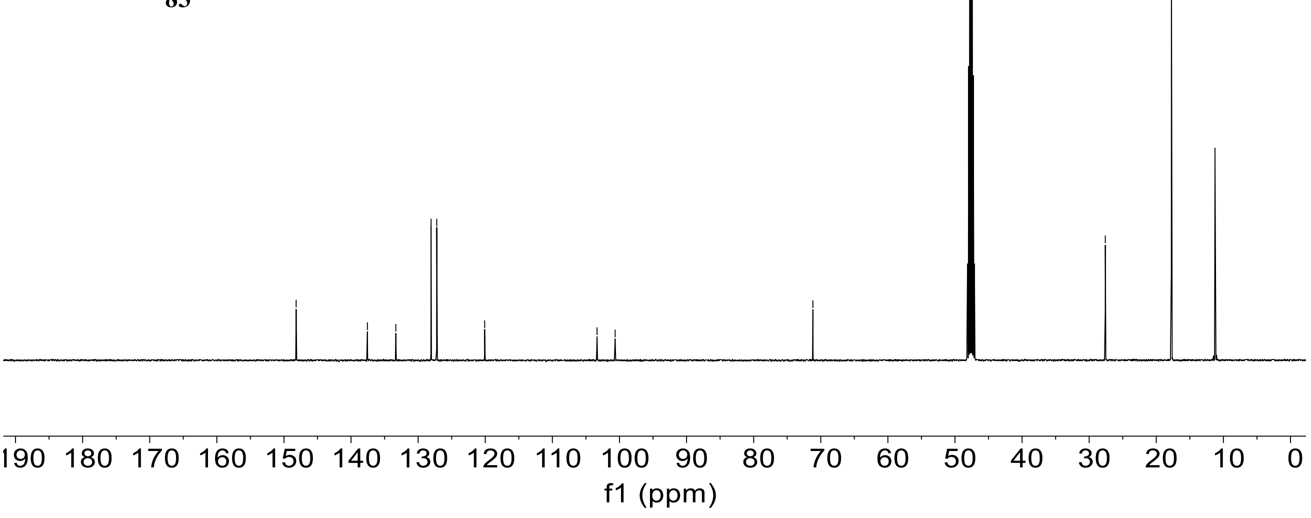
148.18
137.56
133.33
128.07
127.23
120.09
103.34
100.64
71.17
27.60
17.75
11.25
126 MHz, CD3OD, 25 °C
180 170 160 150 140 130 120 110 100 90 80 70 60 50 40 30 20 10 0
f1 (ppm)

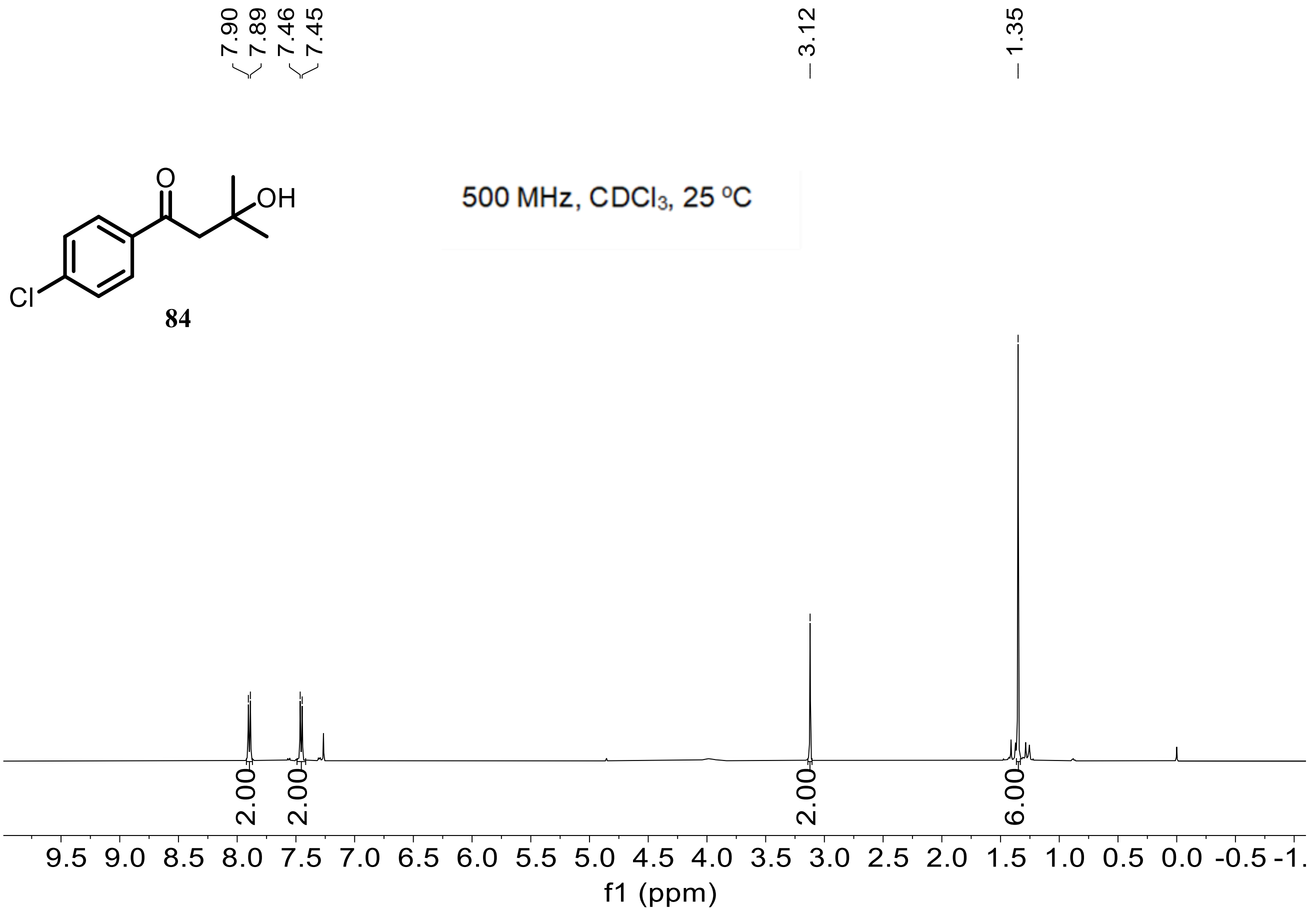
7.90
7.89
7.46
7.45
3.12
1.35
O
OH
Cl
84
500 MHz, CDCl3, 25 °C
2.00
2.00
2.00
6.00
9.5 9.0 8.5 8.0 7.5 7.0 6.5 6.0 5.5 5.0 4.5 4.0 3.5 3.0 2.5 2.0 1.5 1.0 0.5 0.0 -0.5 -1.
f1 (ppm)

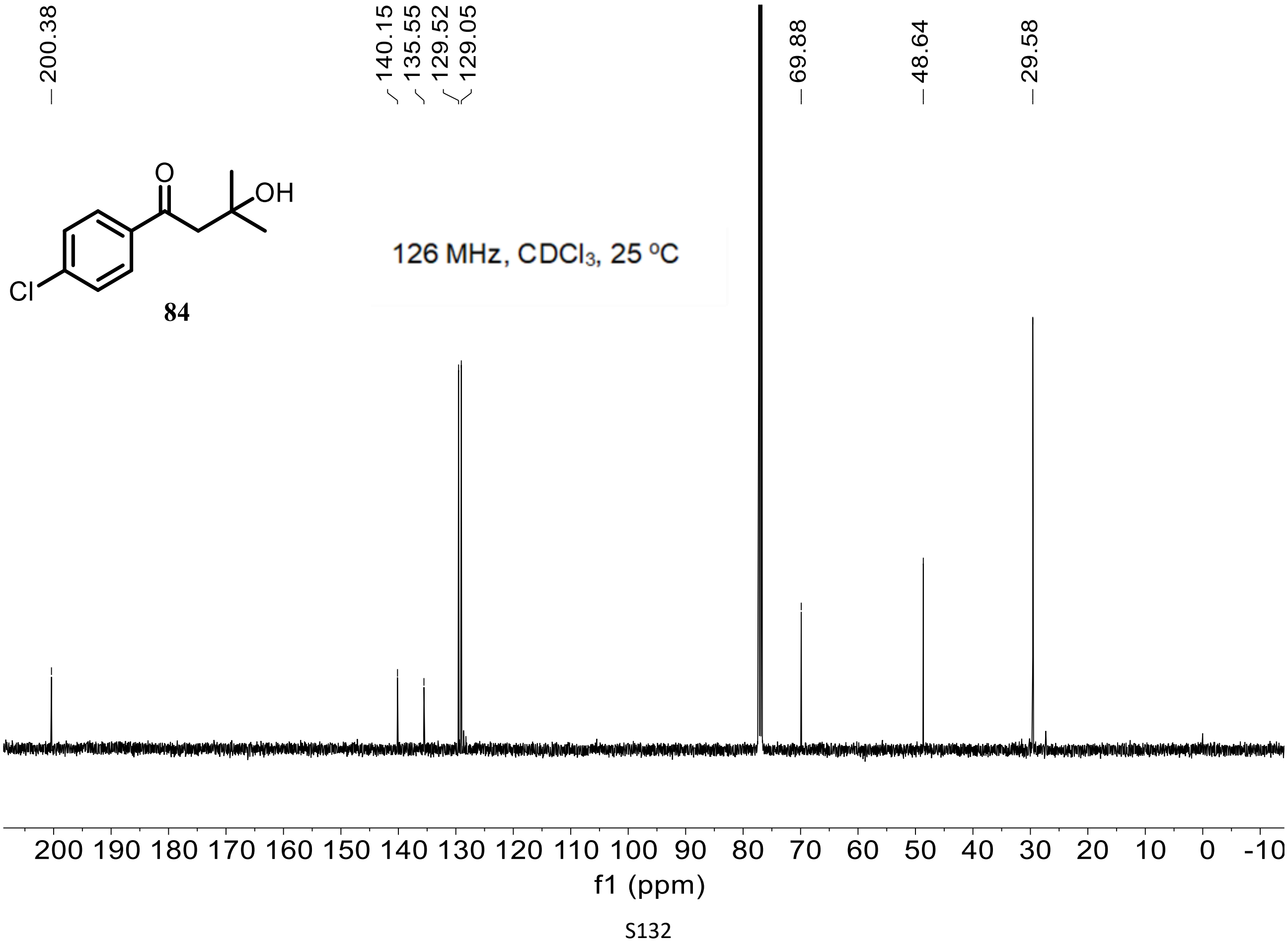
200.38
140.15
135.55
129.52
129.05
69.88
48.64
29.58
O
OH
Cl
84
126 MHz, CDCl3, 25 °C
200 190 180 170 160 150 140 130 120 110 100 90 80 70 60 50 40 30 20 10 0 -10
f1 (ppm)

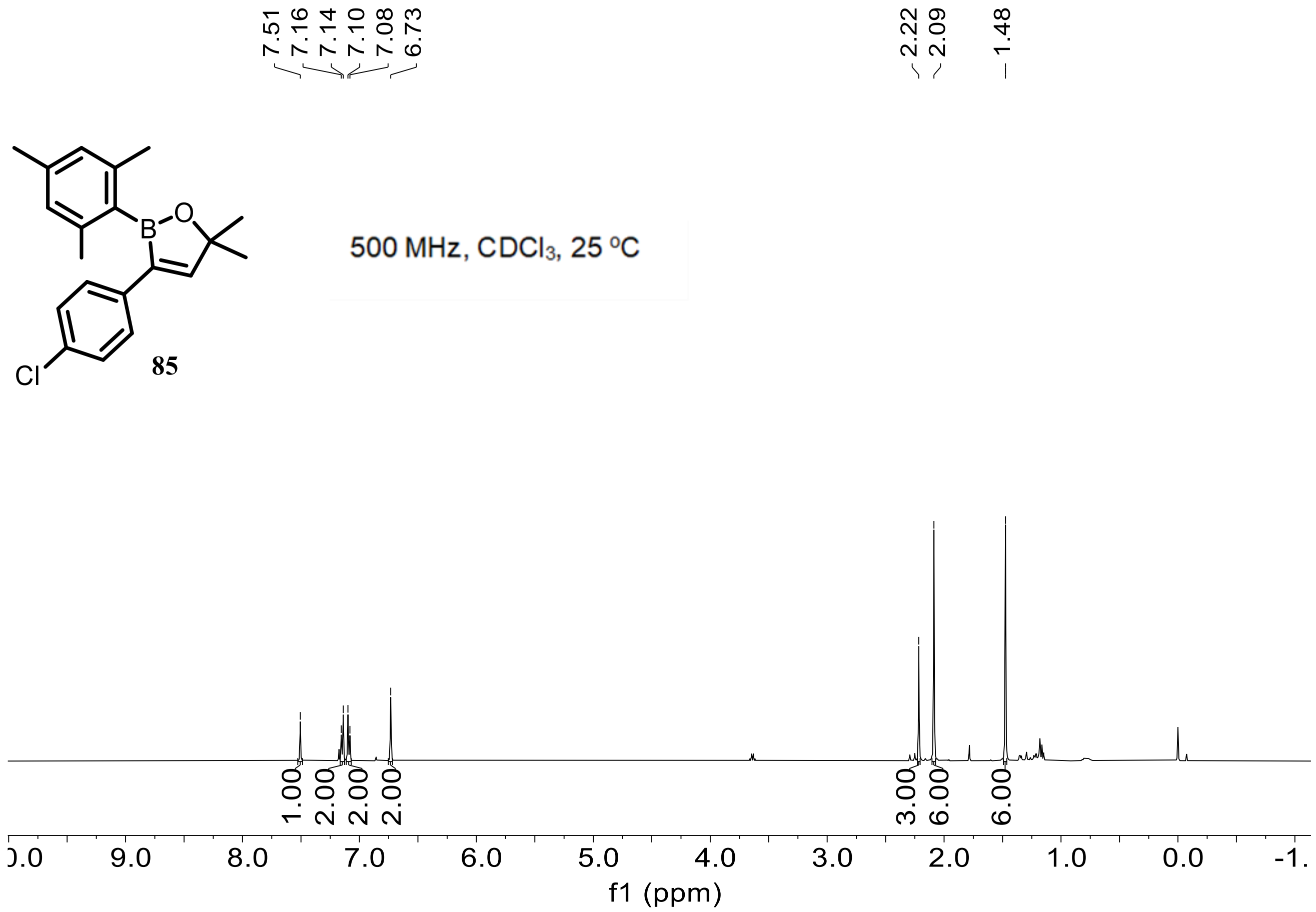
7.51
7.16
7.14
7.10
7.08
6.73
2.22
2.09
1.48
500 MHz, CDCl3, 25 °C
85
1.00
2.00
2.00
2.00
3.00
6.00
6.00
f1 (ppm)

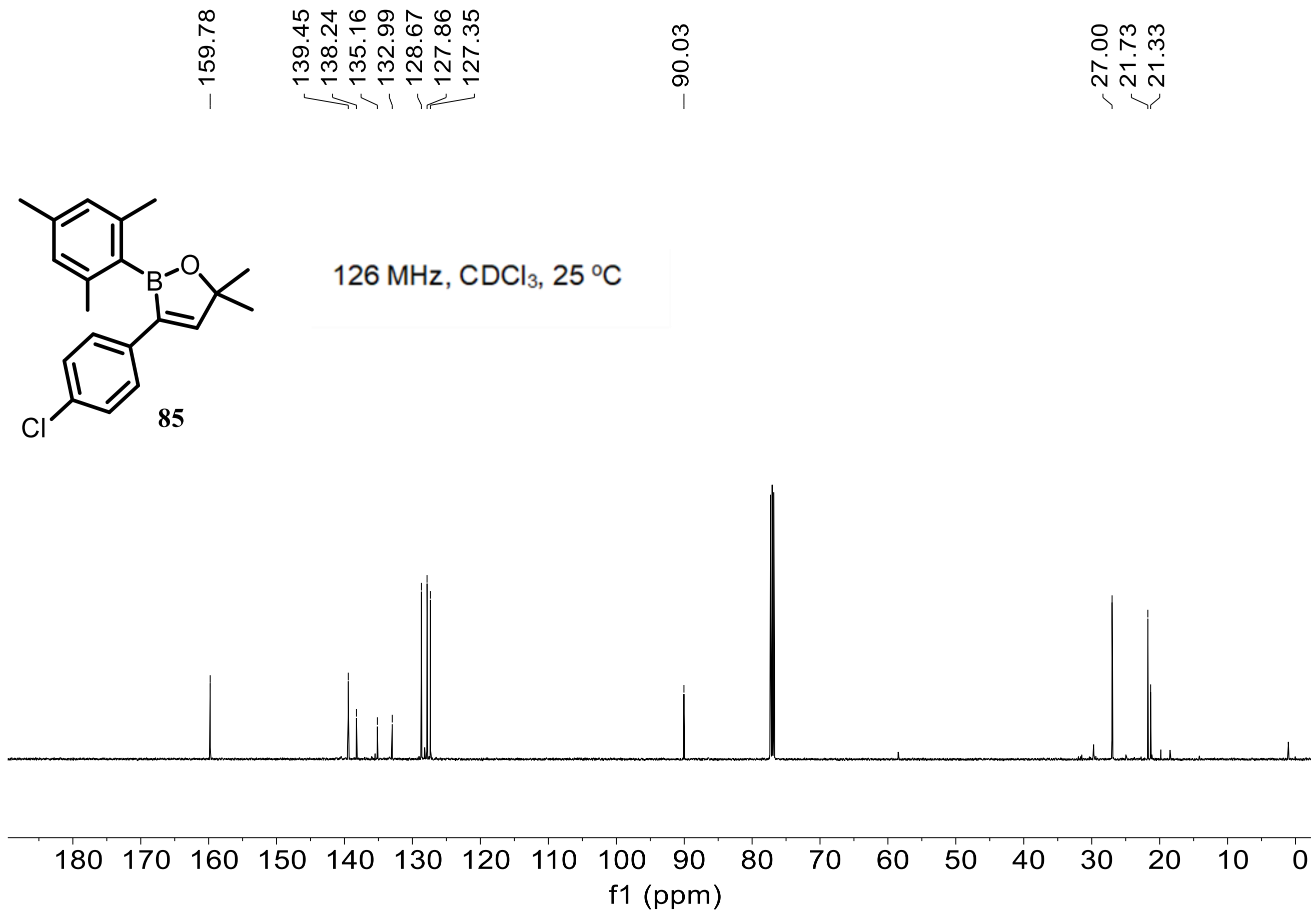
159.78
139.45
138.24
135.16
132.99
128.67
127.86
127.35
90.03
27.00
21.73
21.33
126 MHz, CDCl3, 25 °C
85
f1 (ppm)

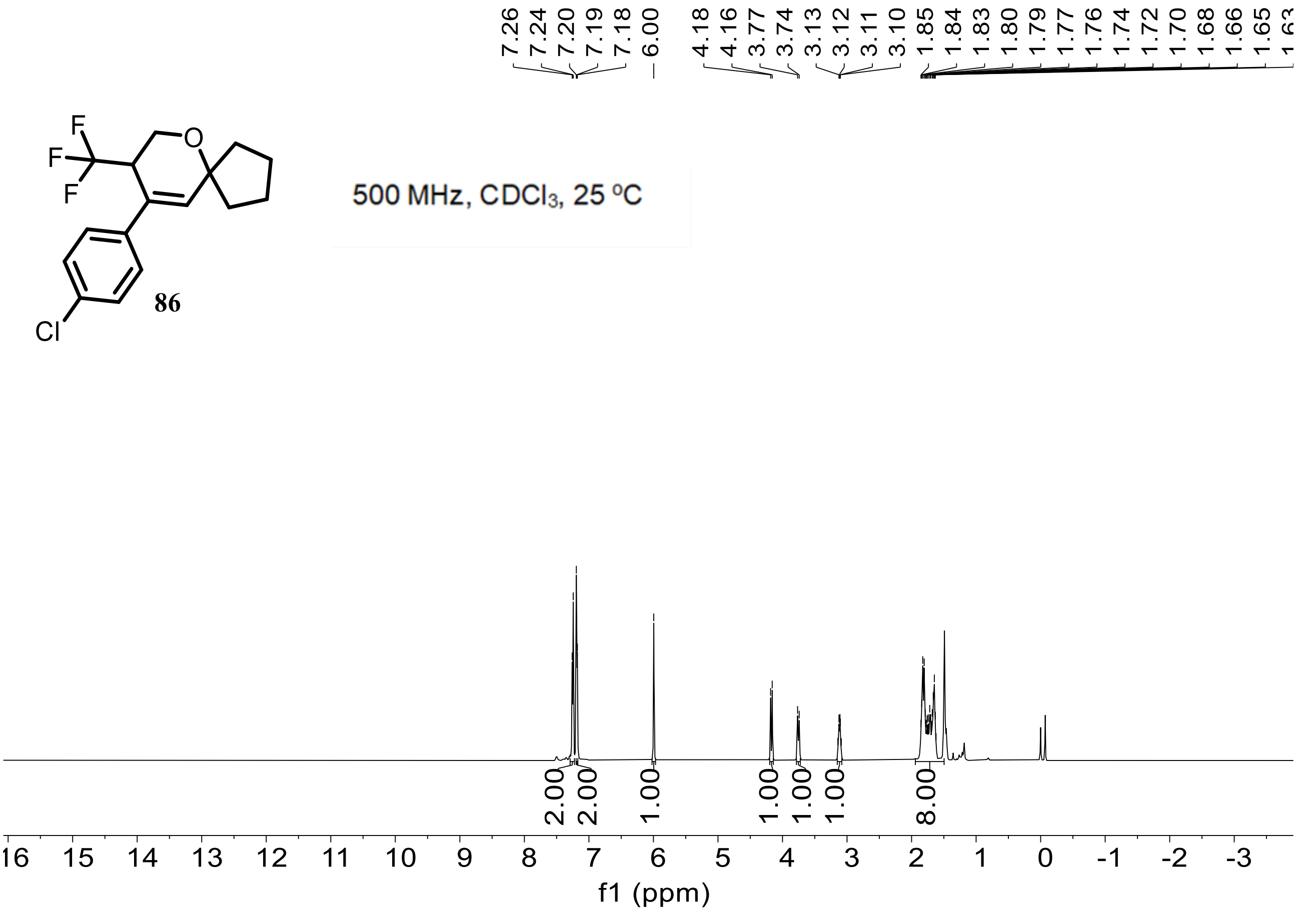

7.26 7.24 7.20 7.19 7.18 6.00 4.18 4.16 3.77 3.74 3.13 3.12 3.11 3.10 1.85 1.84 1.83 1.80 1.79 1.77 1.76 1.74 1.72 1.70 1.68 1.66 1.65
500 MHz, CDCl3, 25 °C
F
F
F
O
Cl
86
2.00 2.00 1.00 1.00 1.00 1.00 8.00
16 15 14 13 12 11 10 9 8 7 6 5 4 3 2 1 0 -1 -2 -3
f1 (ppm)

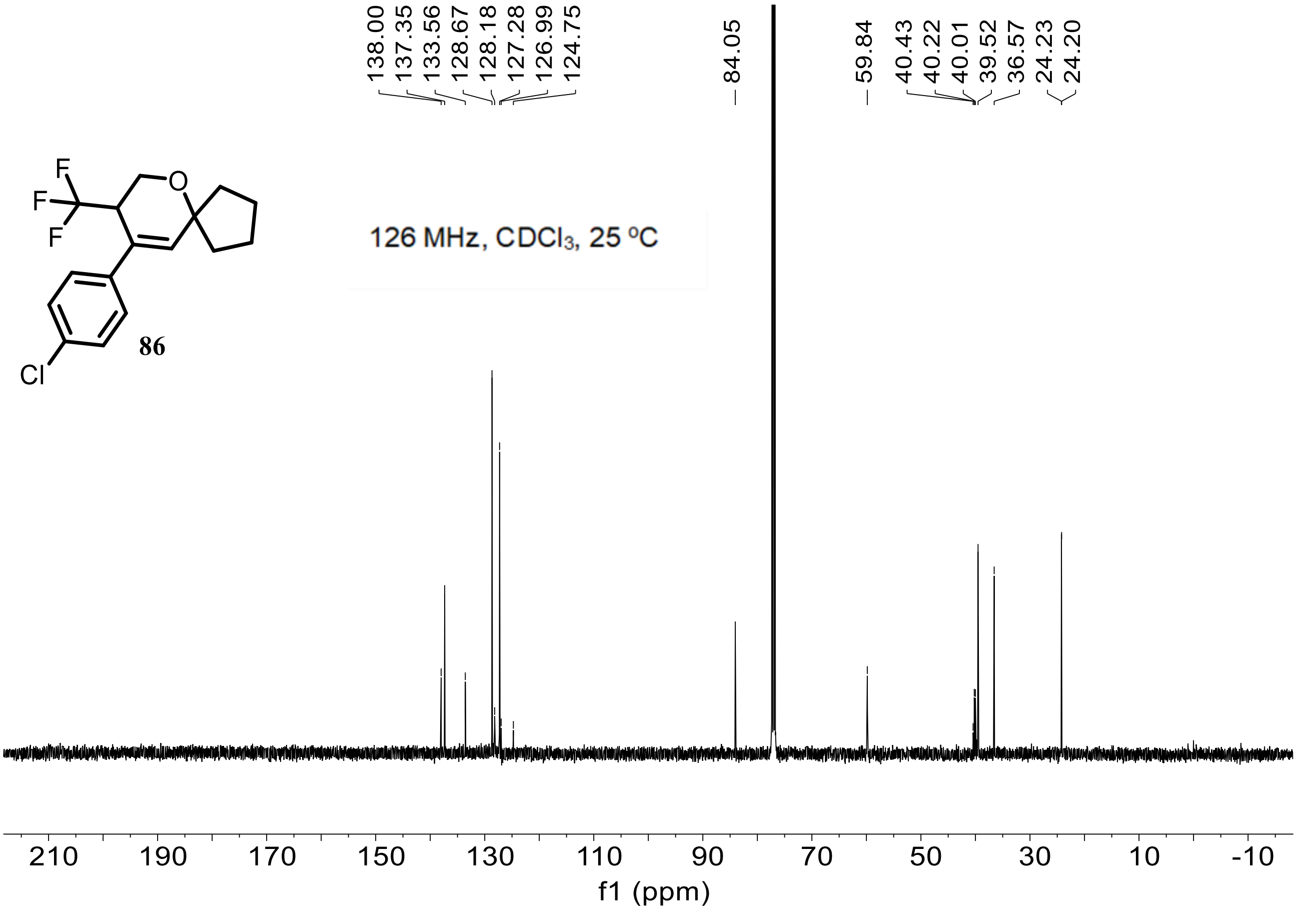

138.00 137.35 133.56 128.67 128.18 127.28 126.99 124.75 84.05 59.84 40.43 40.22 40.01 39.52 36.57 24.23 24.20
126 MHz, CDCl3, 25 °C
F
F
F
O
Cl
86
210 190 170 150 130 110 90 70 50 30 10 -10
f1 (ppm)

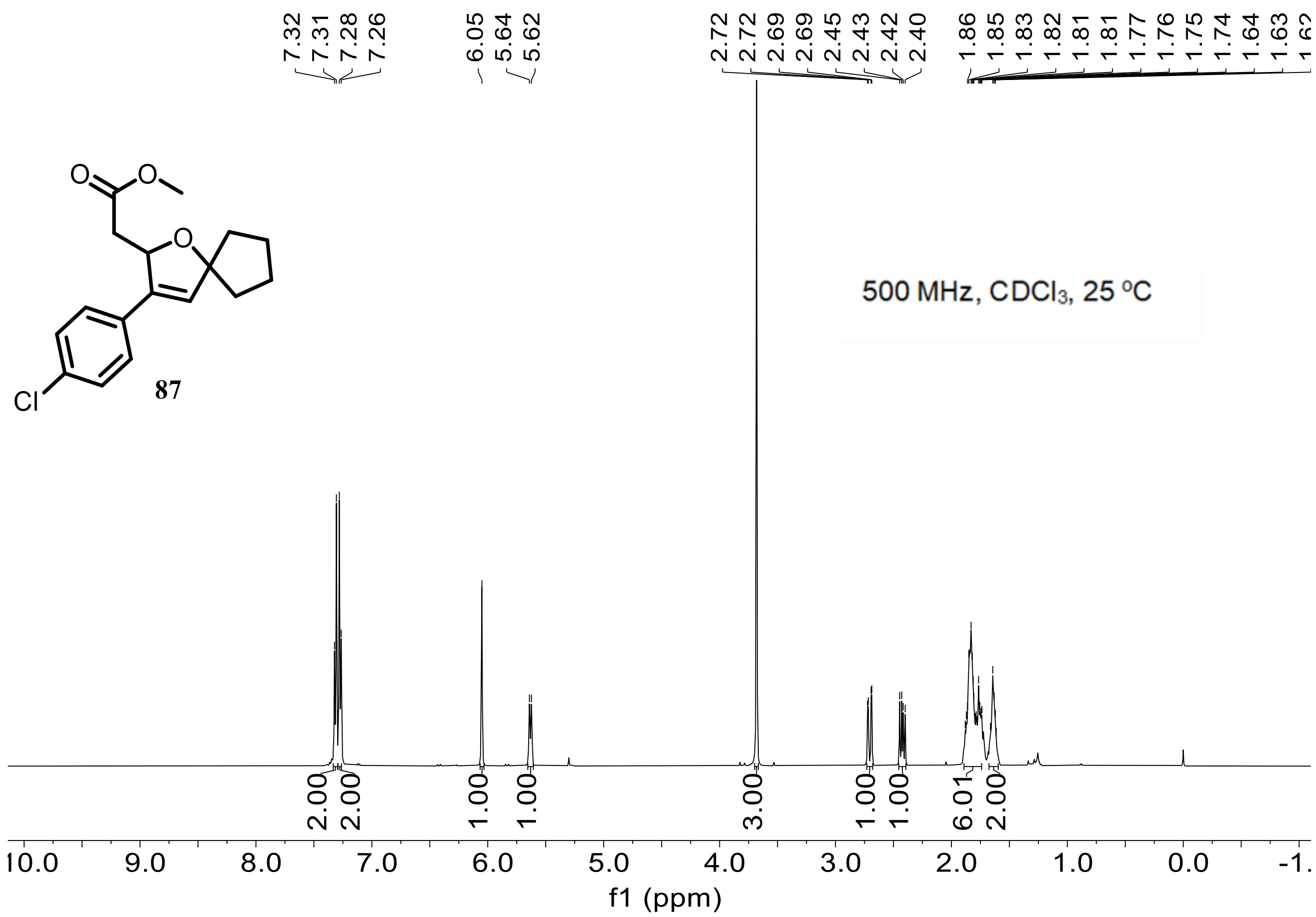

7.32 7.31 7.28 7.26
6.05 5.64 5.62
2.72 2.72 2.69 2.69 2.45 2.43 2.42 2.40
1.86 1.85 1.83 1.82 1.81 1.81 1.77 1.76 1.75 1.74 1.64 1.63
O
O
O
Cl
87
500 MHz, CDCl3, 25 °C
2.00 2.00 1.00 1.00 3.00 1.00 1.00 6.01 2.00
10.0 9.0 8.0 7.0 6.0 5.0 4.0 3.0 2.0 1.0 0.0
f1 (ppm)

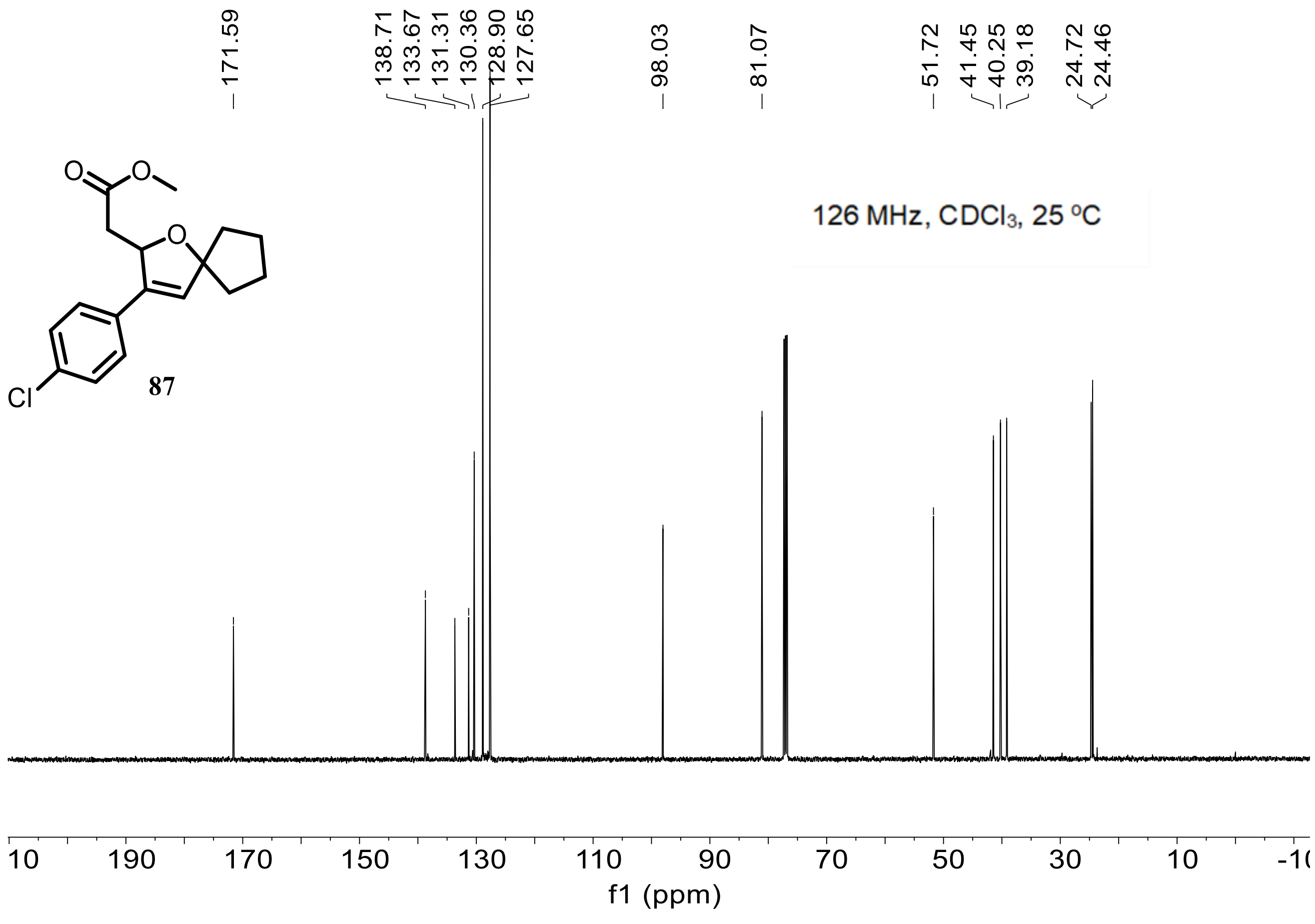

171.59
138.71 133.67 131.31 130.36 128.90 127.65
98.03
81.07
51.72 41.45 40.25 39.18 24.72 24.46
O
O
O
Cl
87
126 MHz, CDCl3, 25 °C
190 170 150 130 110 90 70 50 30 10
f1 (ppm)

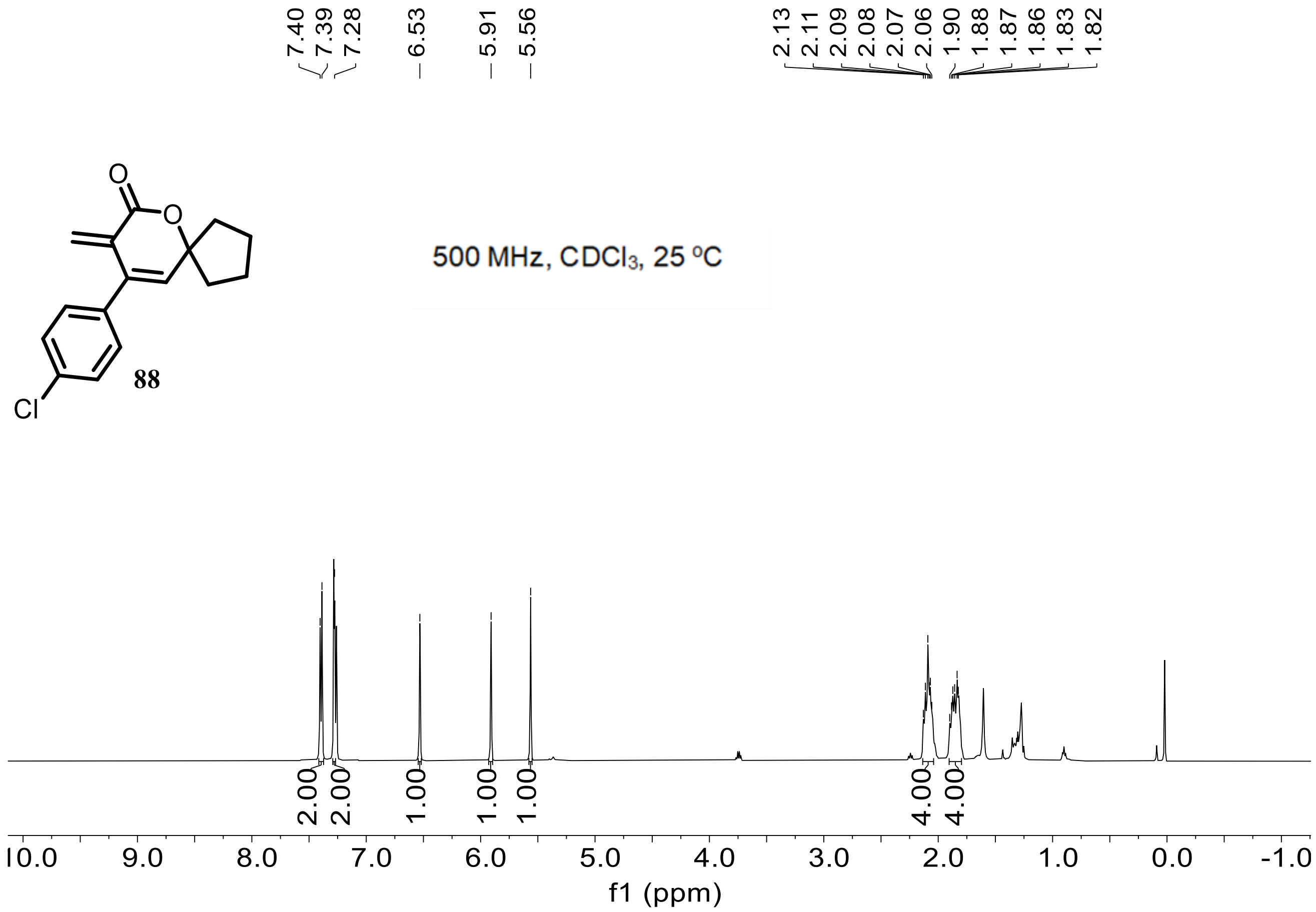
7.40
7.39
7.28
6.53
5.91
5.56
2.13
2.11
2.09
2.08
2.07
2.06
1.90
1.88
1.87
1.86
1.83
1.82
O
O
Cl
88
500 MHz, CDCl3, 25 °C
2.00
2.00
1.00
1.00
1.00
4.00
4.00
10.0 9.0 8.0 7.0 6.0 5.0 4.0 3.0 2.0 1.0 0.0 -1.0
f1 (ppm)

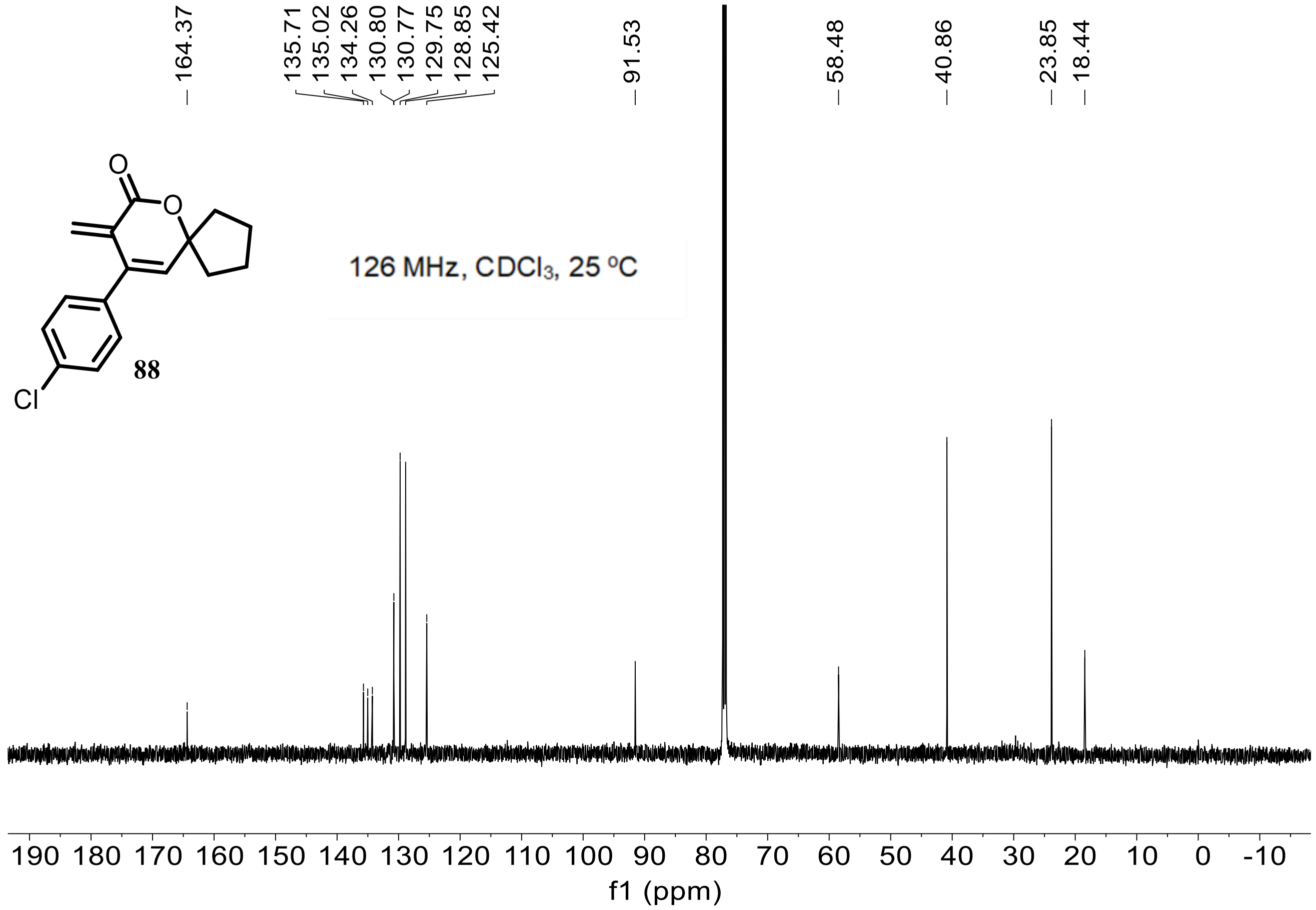
164.37
135.71
135.02
134.26
130.80
130.77
129.75
128.85
125.42
91.53
58.48
40.86
23.85
18.44
O
O
Cl
88
126 MHz, CDCl3, 25 °C
190 180 170 160 150 140 130 120 110 100 90 80 70 60 50 40 30 20 10 0 -10
f1 (ppm)

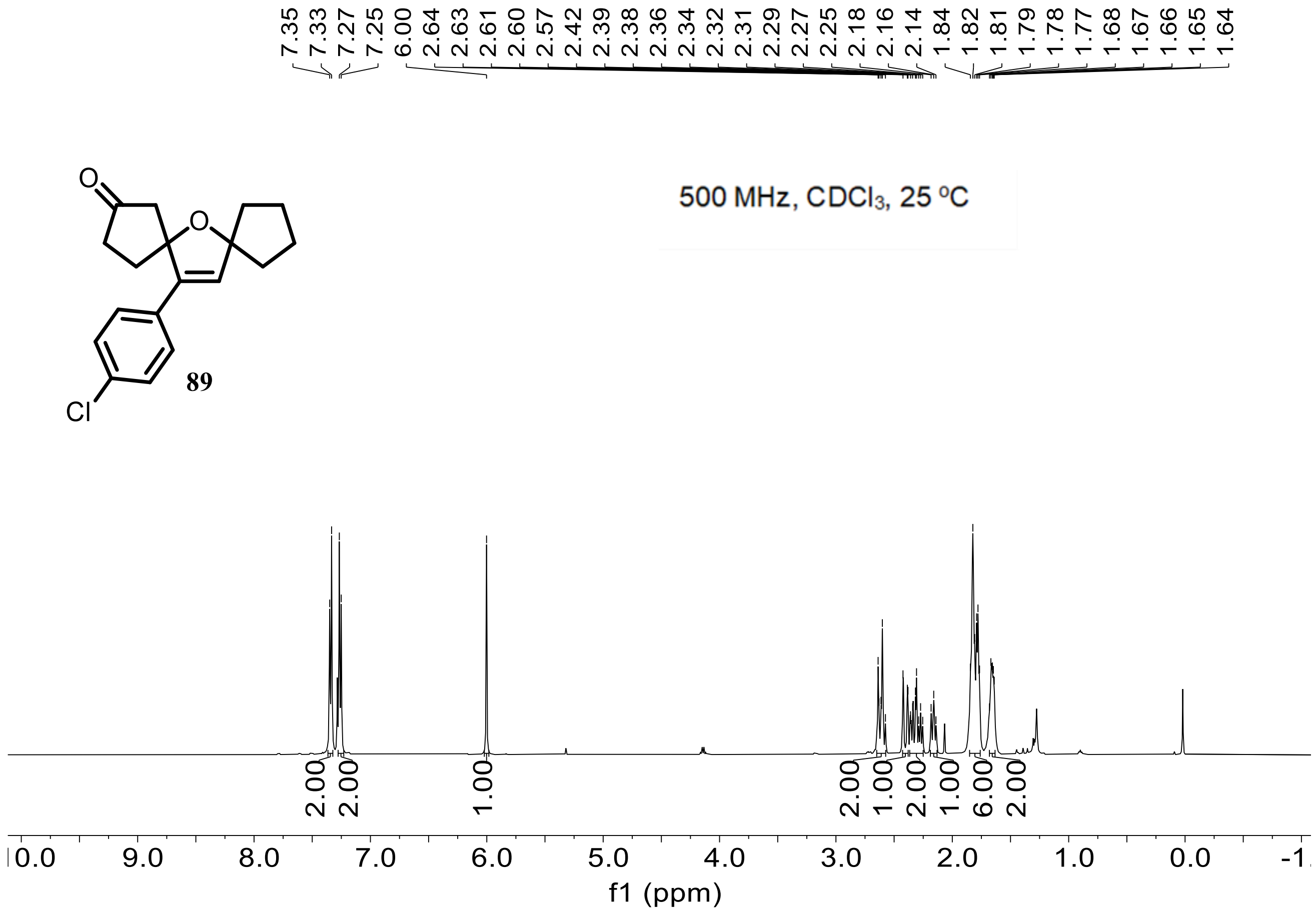
7.35 7.33 7.27 7.25 6.00 2.64 2.63 2.61 2.60 2.57 2.42 2.39 2.38 2.36 2.34 2.32 2.31 2.29 2.27 2.25 2.18 2.16 2.14 1.84 1.82 1.81 1.79 1.78 1.77 1.68 1.67 1.66 1.65 1.64
O
O
Cl
89
500 MHz, CDCl$_3$, 25 °C
2.00 2.00 1.00 2.00 1.00 2.00 1.00 6.00 2.00
f1 (ppm)

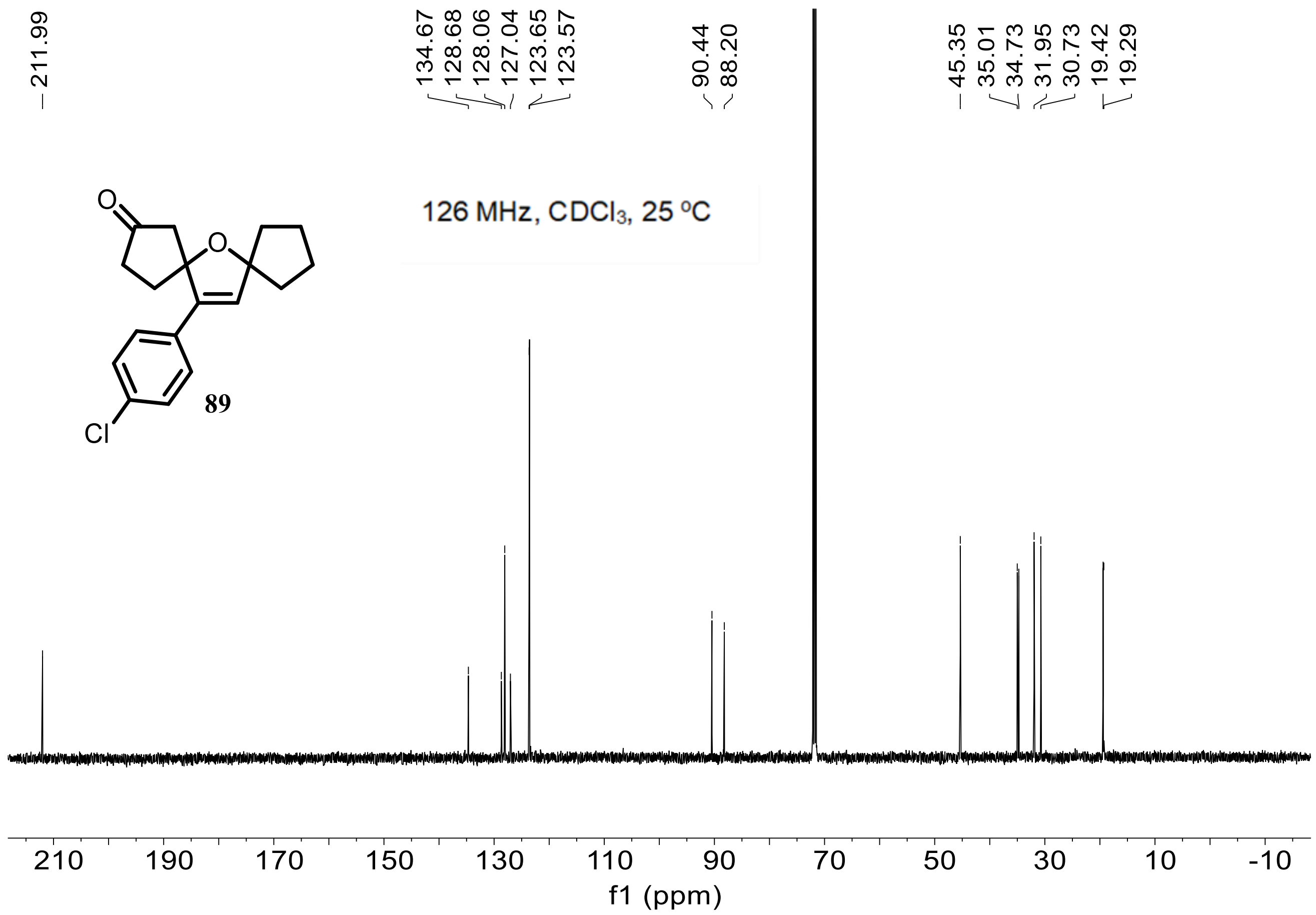
211.99
134.67 128.68 128.06 127.04 123.65 123.57
90.44 88.20
45.35 35.01 34.73 31.95 30.73 19.42 19.29
O
O
Cl
89
126 MHz, CDCl$_3$, 25 °C
f1 (ppm)

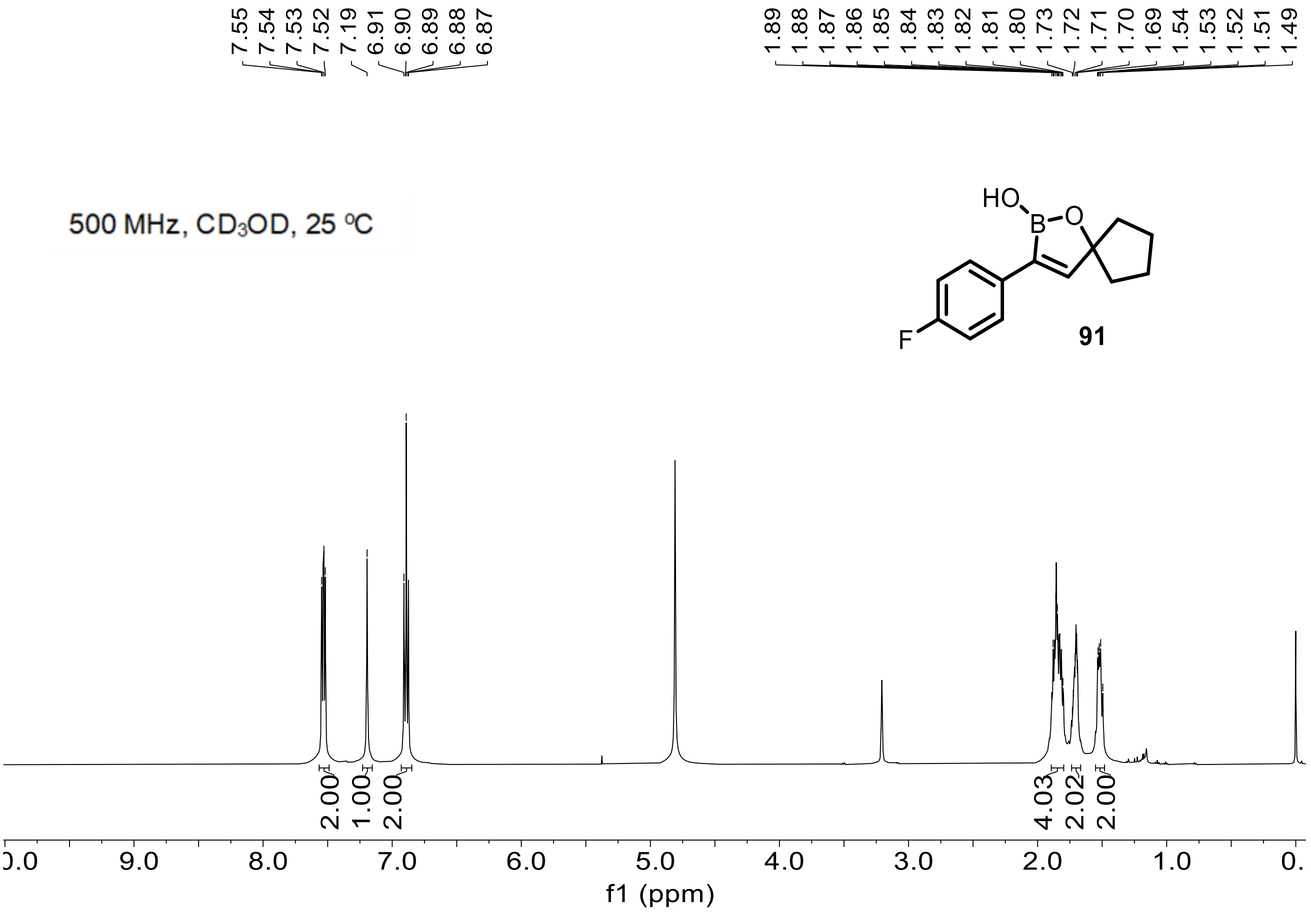
7.55 7.54 7.53 7.52 7.19 6.91 6.90 6.89 6.88 6.87
1.89 1.88 1.87 1.86 1.85 1.84 1.83 1.82 1.81 1.80 1.73 1.72 1.71 1.70 1.69 1.54 1.53 1.52 1.51 1.49
500 MHz, CD3OD, 25 °C
HO
B
O
F
91
2.00 1.00 2.00
4.03 2.02 2.00
f1 (ppm)

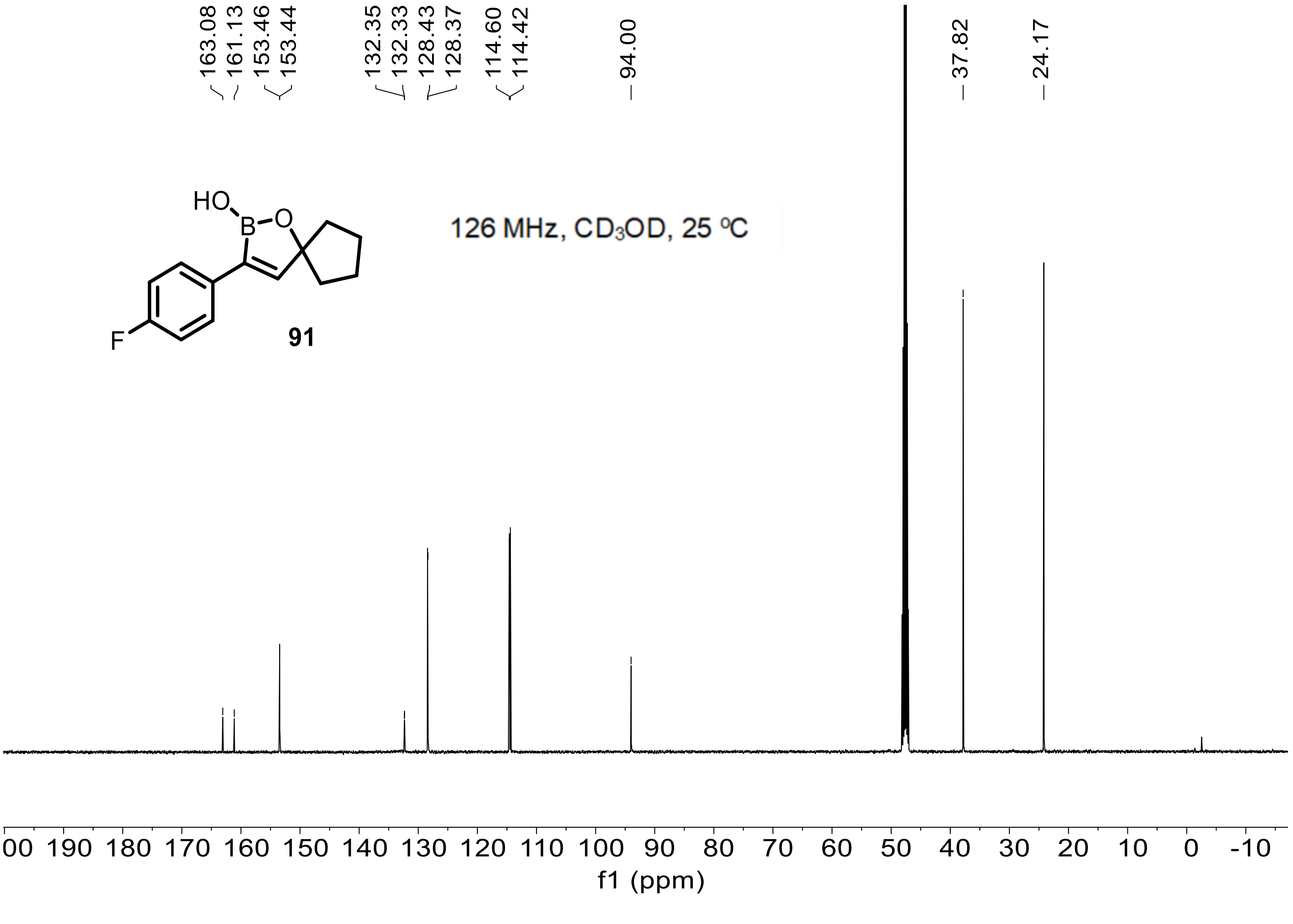
163.08 161.13 153.46 153.44
132.35 132.33 128.43 128.37 114.60 114.42
94.00
37.82
24.17
HO
B
O
F
91
126 MHz, CD3OD, 25 °C
f1 (ppm)

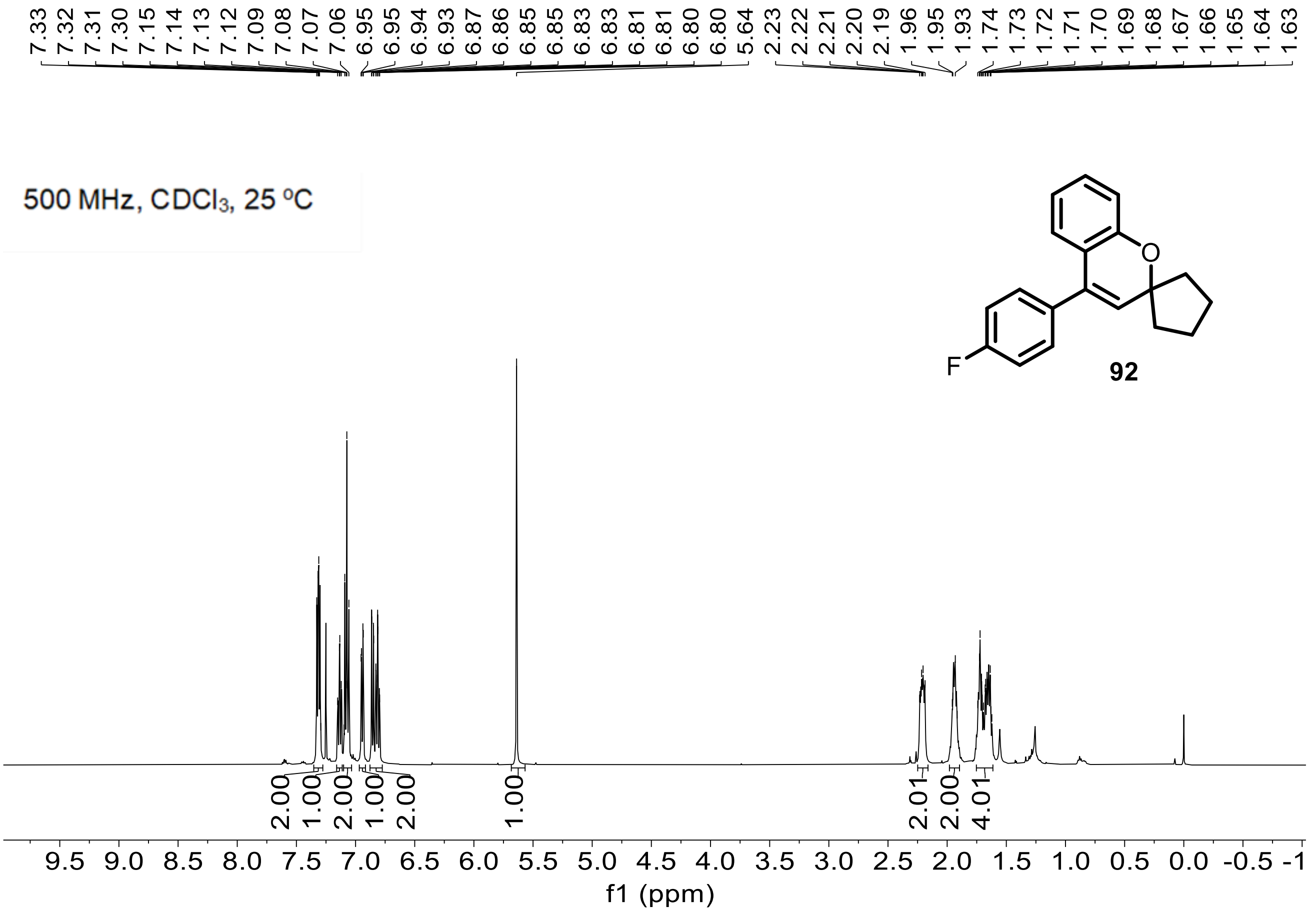
7.33 7.32 7.31 7.30 7.15 7.14 7.13 7.12 7.09 7.08 7.07 7.06 6.95 6.95 6.94 6.93 6.87 6.86 6.85 6.85 6.83 6.83 6.81 6.81 6.80 6.80 5.64 2.23 2.22 2.21 2.20 2.19 1.96 1.95 1.93 1.74 1.73 1.72 1.71 1.70 1.69 1.68 1.67 1.66 1.65 1.64 1.63
500 MHz, CDCl3, 25 oC
O
F
92
2.00 1.00 2.00 1.00 2.00 1.00 2.01 2.00 4.01
9.5 9.0 8.5 8.0 7.5 7.0 6.5 6.0 5.5 5.0 4.5 4.0 3.5 3.0 2.5 2.0 1.5 1.0 0.5 0.0 -0.5 -1
f1 (ppm)

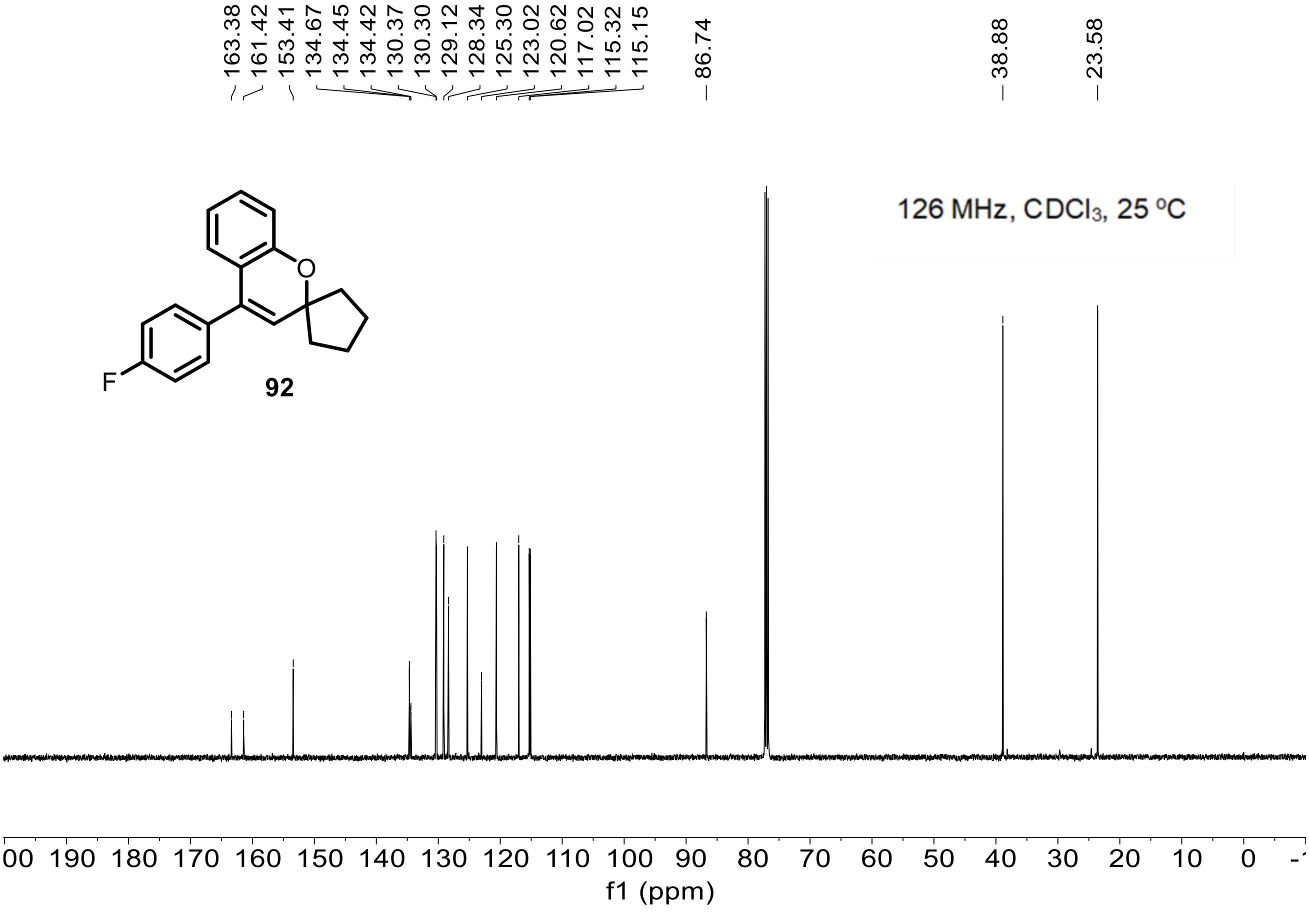
163.38 161.42 153.41 134.67 134.45 134.42 130.37 130.30 129.12 128.34 125.30 123.02 120.62 117.02 115.32 115.15 86.74 38.88 23.58
126 MHz, CDCl3, 25 oC
O
F
92
00 190 180 170 160 150 140 130 120 110 100 90 80 70 60 50 40 30 20 10 0
f1 (ppm)

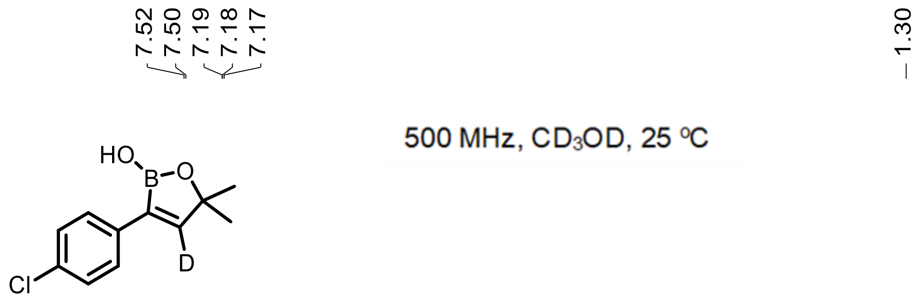

D-11 95%-D

HRMS (ESI): m/z calculated for $[M–H]^{–}$ = 222.0609, found 222.0608.

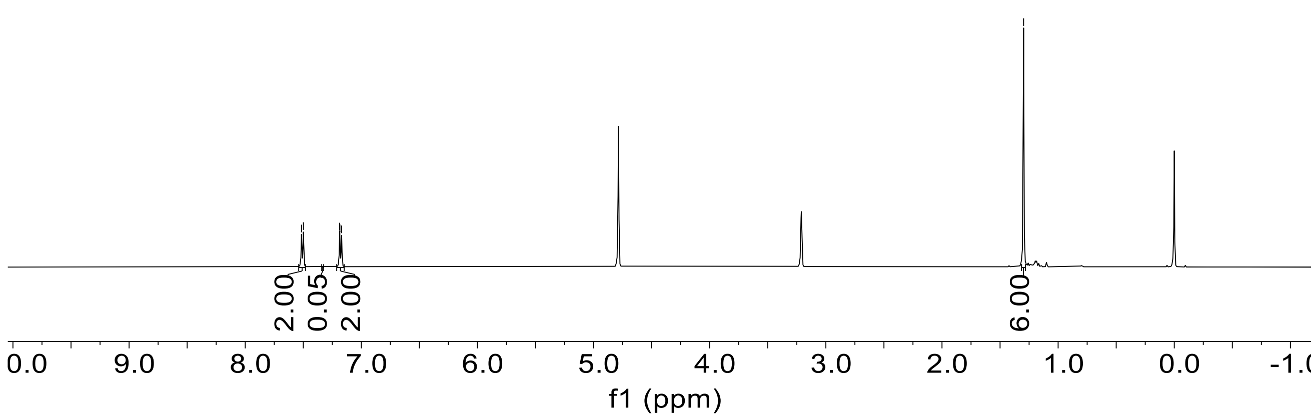

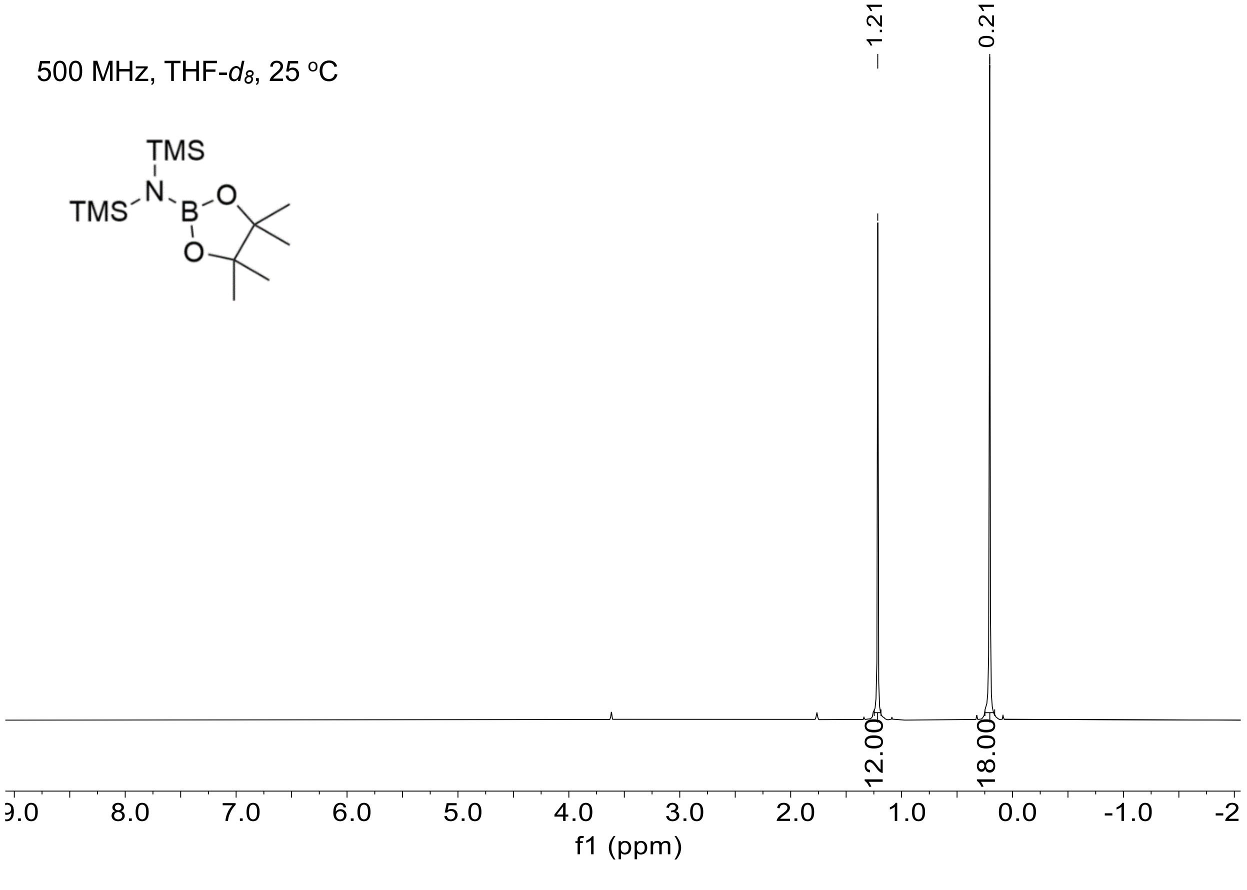

7. Supporting Crystallographic Data

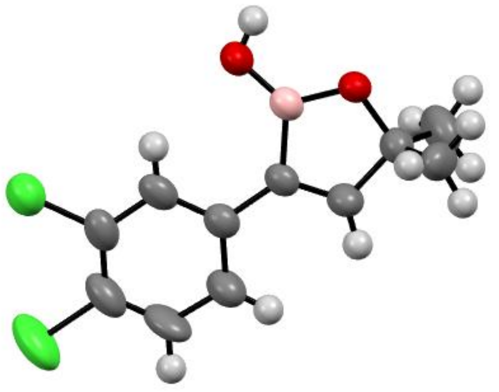

CCDC 2346237

Bond precision:	C-C = 0.0096 A		Wavelength=0.71073
Cell:	a=10.9229(14)	b=9.4760(13)	c=12.3808(18)
	alpha=90	beta=93.918(3)	gamma=90
Temperature:	296 K		

	Calculated	Reported
Volume	1278.5(3)	1278.5(3)
Space group	P 21/c	P 21/c
Hall group	-P 2ybc	-P 2ybc
Moiety formula	C11 H11 B Cl2 O2	?
Sum formula	C11 H11 B Cl2 O2	C11 H11 B Cl2 O2
Mr	256.91	256.91
Dx,g cm-3	1.335	1.335
Z	4	4
Mu (mm-1)	0.488	0.488
F000	528.0	528.0
F000'	529.38	
h,k,lmax	12,11,14	12,11,14
Nref	2248	2246
Tmin,Tmax	0.943,0.962	
Tmin'	0.943	

Correction method= Not given

Data completeness= 0.999 Theta(max)= 24.999

R(reflections)= 0.1039(1134) wR2(reflections)= 0.3301(2246)

S = 1.108 Npar= 152

8. References

1. Köllhofer, A. & Plenio, H. A convenient high activity catalyst for the Sonogashira coupling of aryl bromides. *Adv. Synth. Catal.* **347**, 1295–1300 (2005).
2. Cheng, J., Sun, Y., Wang, F., Guo, M., Xu, J.-H., Pan, Y. & Zhang, Z. A copper- and amine-free Sonogashira reaction employing aminophosphines as ligands. *J. Org. Chem*. **69**, 5428–5432 (2004).
3. Chen, X., Li, M., Liu, Z., Yang, C., Xie, H., Hu, X., Su, S.-J., Jiang, H. & Zeng, W. Bimetal cooperatively catalyzed arylalkynylation of alkynylsilanes. *Org. Lett*. **23**, 6724–6728 (2021).
4. Jin, H., Fürstner, A. Regioselective *trans*-carboboration of propargyl alcohols. *Org. Lett.* **21**, 3446–3450 (2019).
5. Jin, H. & Fürstner, A. Modular synthesis of furans with up to four different substituents by a *trans*-carboboration strategy. *Angew. Chem. Int. Ed*. **59**, 13618–13622 (2020).
6. Nagashima, Y., Hirano, K., Takita, R. & Uchiyama, M. *Trans*-diborylation of alkynes: *pseudo*-intramolecular strategy utilizing a propargylic alcohol unit. *J. Am. Chem. Soc*. **136**, 8532−8535 (2014).
7. Nogami, M., Hirano, K., Kanai, M., Wang, C., Saito, T., Miyamoto, K., Muranaka, A. & Uchiyama, M. Transition metal-free *trans*-selective alkynylboration of alkynes. *J. Am. Chem. Soc*. **139**, 12358–12361 (2017).
8. Zhang, X., Liu, J., Sun, X. & Du, Y. An efficient *cis*-reduction of alkyne to alkene in the presence of a vinyl iodide: stereoselective synthesis of the C22-C31 fragment of leiodolide A. *Tetrahedron* **69**, 1553–1558 (2013).
9. The preparation of DBpin according to this literature: Hamdaoui, M., Liu, F., Cornaton, Y., Lu, X., Shi, X., Zhang, H., Liu, J., Spingler, B., Djukic, J.-P. & Duttwyler, S. An iridium-stabilized borenium intermediate. *J. Am. Chem. Soc.* **144**, 18359–18374 (2022).
10. Banfi,L., Narisano, E. & Riva, R. Potassium triisopropoxyborohydride. Encyclopedia of Reagents for Organic Synthesis. Wiley (2001).
11. Bettinger, H. F., Filthaus, M., Bornemann, H. & Oppel, I. M. Metal-free conversion of methane and cycloalkanes to amines and amides by employing a borylnitrene. *Angew. Chem. Int. Ed.* **47**, 4744 (2008).
12. Cui, Y., Li, W., Sato, T., Yamashita, Y. & Kobayashi, S. Catalytic use of zinc amide for transmetalation with allylboronates: general and efficient catalytic allylation of carbonyl compounds, imines, and hydrazones. *Adv. Synth. Catal.* **355**, 1193–1205 (2013).
13. Guo, S.-R. & Yuan, Y.-Q. Copper-catalyzed alkenylation of alcohols with β-nitrostyrenes via a radical addition–elimination process. *Synlett* **26**, 1961–1968 (2015).
14. Tang, Y., Fan, Y., Zhang, Y., Li, X. & Xu, X. Metal-free *tert*-Butyl hydrogenperoxide (TBHP) mediated radical alkylation of enol acetates with alcohols: a new route to β-hydroxy ketones. Synlett 27, 1860–1863 (2016).
15. Frisch, M. J., Trucks, G. W., Schlegel, H. B., Scuseria, G. E., Robb, M. A., Cheeseman, J. R., Scalmani, G., Barone, V., Petersson, G. A., Nakatsuji, H., Li, X., Caricato, M., Marenich, A. V., Bloino, J., Janesko, B. G., Gomperts, R., Mennucci, B., Hratchian, H. P., Ortiz, J. V., Izmaylov, A. F., Sonnenberg, J. L., Williams-Young, D., Ding, F., Lipparini, F., Egidi, F., Goings, J., Peng, B., Petrone, A., Henderson, T., Ranasinghe, D., Zakrzewski, V. G., Gao, J., Rega, N., Zheng, G., Liang, W., Hada, M., Ehara, M., Toyota, K., Fukuda, R., Hasegawa, J., Ishida, M., Nakajima, T., Honda, Y., Kitao, O., Nakai, H., Vreven, T., Throssell, K., Montgomery, J. A., Peralta, Jr., J. E., Ogliaro, F., Bearpark, M. J., Heyd, J. J., Brothers, E. N., Kudin, K. N., Staroverov, V. N., Keith, T. A., Kobayashi, R., Normand, J., Raghavachari, K.,

Rendell, A. P., Burant, J. C., Iyengar, S. S., Tomasi, J., Cossi, M., Millam, J. M., Klene, M., Adamo, C., Cammi, R., Ochterski, J. W., Martin, R. L., Morokuma, K., Farkas, O., Foresman, J. B. & Fox, D. J. Gaussian, Inc., Wallingford CT, 2009.

16. Becke, A. D. Density-functional thermochemistry. III. The role of exact exchange. *J. Chem. Phys*. **98**, 5648–5652 (1993).
17. Grimme, S., Ehrlich, S. & Goerigk, L. Effect of the damping function in dispersion corrected density functional theory. *J. Comput. Chem*. **32**, 1456–1465 (2011).
18. Weigend, F. & Ahlrichs, R. Balanced basis sets of split valence, triple zeta valence and quadruple zeta valence quality for H to Rn: Design and assessment of accuracy. *Phys. Chem. Chem. Phys.* **7**, 3297–3305 (2005).
19. Fukui, K. Formulation of the reaction coordinate. *J. Phys. Chem*. **74**, 4161–4163 (1970).
20. Fukui, K. The path of chemical reactions - the IRC approach. *Acc. Chem. Res*. **14**, 363–368 (1981).
21. Marenich, A. V., Cramer, C. J. & Truhlar, D. G. Universal Solvation Model Based on Solute Electron Density and on a Continuum Model of the Solvent Defined by the Bulk Dielectric Constant and Atomic Surface Tensions. *J. Phys. Chem. B* **113**, 6378–6396 (2009).
22. Zhao, Y. & Truhlar, D. G. The M06 suite of density functionals for main group thermochemistry, thermochemical kinetics, noncovalent interactions, excited states, and transition elements: two new functionals and systematic testing of four M06-class functionals and 12 other functionals. *Theor. Chem. Acc.* **120**, 215–241 (2008).
23. Pritchard, B. P., Altarawy, D., Didier, B., Gibsom, T. D. & Windus, T. L. A new basis set exchange: an open, up-to-date resource for the molecular sciences community. *J. Chem. Inf. Model* **59**, 4814–4820 (2019).
24. Weigend, F. & Ahlrichs, R. Balanced basis sets of split valence, triple zeta valence and quadruple zeta valence quality for H to Rn: design and assessment of accuracy. *Phys. Chem. Chem. Phys.* **7**, 3297 (2005).
25. Schuchardt, K. L. et al. Basis set exchange: a community database for computational sciences. *J. Chem. Inf. Model* **47**, 1045–1052 (2007).
26. Rappoport, D. & Furche, F. Property-optimized Gaussian basis sets for molecular response calculations. *J. Chem. Phys.* **133**, 134105 (2010).
27. Feller, D. The role of databases in support of computational chemistry calculations. *J. Comput. Chem.* **17**, 1571–1586 (1996).
28. CYLview, 1.0b; C. Y. Legault, Université de Sherbrooke, 2009 (http://www.cylview.org).
29. Lu, T. & Chen, F. Multiwfn-Multiwfn: A multifunctional wavefunction analyzer. *J. Comput. Chem*. **33**, 580–592 (2012).
30. Pang, Y., Liu, G., Huang, C., Yuan, X.-A., Li, W. & Xie, J. A highly efficient dimeric manganese-catalyzed selective hydroarylation of internal alkynes. *Angew. Chem. Int. Ed*. **59**, 12789-12794 (2020).
